# Checklist of the Diptera (Insecta) of Finland: an introduction and a summary of results

**DOI:** 10.3897/zookeys.441.7620

**Published:** 2014-09-19

**Authors:** Jere Kahanpää

**Affiliations:** 1 Finnish Museum of Natural History, Zoology Unit, P.O. Box 17, FI-00014 University of Helsinki, Finland

**Keywords:** Finland, Diptera, flies, biodiversity, faunist

## Abstract

Nearly thirty-five years have passed since Hackman published his “Check list of the Finnish Diptera” (1980). The number of true flies (Diptera) known from Finland has increased by more than two thousand species since then. At the same time, hundreds of erroneous records have been recognized and purged from the checklist.

ZooKeys issue 441 provides a new checklist of the Diptera species of the Republic of Finland. This introductory paper presents the rationale behind the project, provides technical documentation on the checklist format and sources used, and summarizes the results. The remaining papers in this issue cover one or more Diptera families in detail.

Two electronic appendices are provided: supporting data (additional references to first published records and the previous checklist) and a complete list of Finnish Diptera taxa in Darwin Core compliant format for easy computer access and processing.

The new checklist records 6920 fly species from Finland, 2932 belonging to the nematoceran or lower flies and 3989 to the suborder Brachycera. The changes since 1980 are most prominent in the Lower Diptera. For example, more than 400 non-biting midges (Chironomidae) have been added since 1980, and the number of moth flies (Psychodidae) known from Finland has more than tripled. Among the larger families, large increases in known Finnish species are also seen in Cecidomyiidae (161% increase), Pipunculidae (98%), and Chironomidae (90%).

## Introduction

The Diptera is a large order of holometabolous insects commonly known as flies and midges. With some 150,000 described extant species (Pape et al. 2011) and many more still undescribed, the Diptera is one of the most successful groups of animals by any measure. Flies and midges are also an important part of food webs in most land and freshwater ecosystems, particularly so in the arctic and alpine zones (Mani 1968, Pape 2009a). The economic relevance of flies is also considerable (see Pape 2009b for a recent summary): they are key pollinators and biological control agents, but also – less beneficially – important vectors of human and domestic animal diseases.

### Why a new checklist?

The history of Finnish Diptera catalogs starts with Evert J. Bonsdorff (1861, 1866), who reviewed a part of the brachyceran fauna known from the country at the time, totaling 657 species. Systematic maintenance and publishing of faunistic data began with the start of Wolter Hellén’s amazing series of summary papers covering new country records (and deletions) of all Insecta and running uninterrupted for six decades (Hellén 1922, 1926, 1931, 1936, 1941, 1946, 1952, 1956, 1961, 1966, 1971, 1976). Richard Frey, Lauri Tiensuu, and Ragnar Storå (1941) published the first complete list of Diptera recorded from Finland. It included 3824 species.

The era of Hellén culminated in a revised list of Finnish Diptera by Walter Hackman (1980a, 1980b, 1980c) in cooperation with Bernhard Lindeberg and Rauno Väisänen. The number of species had increased to 4852. Hans Silfverberg succeeded Hellén as the author of regular updates on additions and deletions to the Finnish insect fauna (Silfverberg 1981, 1986, 1991, 1996, 2007, 2012).

Nearly thirty-five years have passed since Hackman’s checklist. The number of Diptera species known from Finland has increased by more than two thousand species since 1980 (an increase of more than 40%). Several hundred erroneous records have been recognized during the same period. Many new names have been introduced and others found invalid, incorrectly used, or synonymized. The Diptera fauna of the whole Palaearctic Region has been catalogued (Soós and Papp 1984–1993) and many new local, regional, or global checklists have been published.

At this point, an updated checklist is urgently needed to provide a current reference to the Diptera fauna of Finland as a fresh starting point for further studies into the taxonomy, ecology, and other aspects of flies in Northern Europe.

## Methods and format

### Definition of a checklist

There is no universally accepted definition for an entomological checklist. The word is often used for lists of species with more information than merely valid names, but without comprehensive details on nomenclature or distribution and incomplete literature references. This new checklist of Finnish Diptera falls into category 5 on the comprehensiveness scale of Thompson and Knutson (1987). It does include full names with authorships and some but not all synonyms. Some literature references are provided in an Suppl. material 2.

National insect checklists have traditionally included the species recorded at least once from the country. Exotic imports without locally reproducing populations are usually excluded, but vagrants are included. This is by no means the only possible approach: the latest Danish list (Petersen and Meier 2001) use a predictive approach: it lists not only the species actually recorded from the country, but also those that are likely to be present based on the fauna of neighboring countries. The latter method works best when the fauna of the surrounding areas is at least as well known as the study area.

### Taxonomic, geographical, and temporal limits

This checklist covers the Diptera fauna of the Republic of Finland. Only species recorded at least once within the current (*i.e.* post-1944) borders are included.

Many species were originally reported from Finland on the basis of specimens collected from areas ceded to Russia in 1944. If no reliable records (preferably voucher specimens) from within the current borders of the country exist, species were excluded from the checklist. In some cases – *e.g.* the muscid *Coenosia comita* (Huckett, 1936) – it has been impossible to determine whether the purported Finnish collecting localities fall within the current borders of the country. These species are indicated with a question mark on the checklist and are usually accompanied by a comment in the Notes section of the relevant paper.

No fossil or subfossil records were considered during the preparation of the checklist. Nationally extinct species are included. In practice, the oldest collected Diptera specimens from Finland are from the early 19^th^ century, so all of the species in this checklist have been found in the country at least once during the last two hundred years.

### Data sources and validation

Draft checklists for each family were created in the latter part of 2012 by combining data from the most recent checklist of the Finnish fauna of the family in question, the Palaearctic Catalog (Soós and Papp 1984–1993), Fauna Europaea (Beuk and Pape 2012), Silfverberg’s updates to Hackman’s checklist and references therein, other relevant scientific literature, and the Finnish Insect Record Database (Finnish Museum of Natural History 2013). From here onwards, the individual authors of each checklist paper were responsible for data validation.

For a great majority of families, the next step was examining the major Diptera collections in Finland (see the Acknowledgments). Fortunately, these collections have voucher specimens for most Diptera species reported from the country. If no recently identified specimens from the country could be found, the reliability of the record was judged by section authors based on details provided in the literature. For example, the record of *Alliopsis longiceps* (Ringdahl, 1935) from Finland was rejected. While Ringdahl correctly recorded this species from Finland in 1935, the type location (“Kuusamo bei Paanajärvi”) is now Russian territory, and no later observations have been published.

Exceptions to the procedure include Trichoceridae, Ceratopogonidae, Cecidomyiidae (subfamily Cecidomyiinae), and Phoridae (genus *Megaselia* Rondani, 1856), for which the checklists are largely based on a critical literature review. For more details on sources and validation, see the introductions and notes of individual checklist papers.

### Checklist structure and presentation

The new checklist of the Diptera of Finland is presented in three formats: a series of papers each covering one or more families, a comma separated values (CSV) file with the full taxon list, and a PDF (portable document format) file with literature references for species records. The latter two are included as electronic appendices to this paper.

All checklist papers follow the same general format. An introduction to the families covered is followed by a table or list of number of species recorded from the world, Europe, and Finland. An estimate of the faunistic level of knowledge is also given on a simple three-step scale (poor–average–good). This estimate is admittedly very subjective, being based on individual authors’ judgment. The following factors were considered: the number of species known from Finland in comparison with neighboring countries, taking into account known habitat preferences, etc., of absent species; the number of specialists who have worked with Finnish fauna; the number of publications on the Finnish fauna; and the quantity and quality of identified material collected from Finland.

The Checklist section of each paper starts with the systematic position of the family or families treated. As an example, the checklist of lauxanoid flies starts with:

suborder Brachycera Macquart, 1834

clade Eremoneura Lameere, 1906

clade Cyclorrhapha Brauer, 1863

infraorder Schizophora Becher, 1882

clade Muscaria Enderlein, 1936

parvorder Acalyptratae Macquart, 1835

superfamily Lauxanoidea Macquart, 1835

The family-level classification used follows mostly Marshall (2012), with exceptions noted in the Introductions of individual papers. The presentation order of subfamilies, tribes, genera, and subgenera can be either alphabetical or systematic depending on the author’s preference. Species are always listed alphabetically within a genus or subgenus.

Each species record starts with the name of the taxon, the author’s name, and the year of description. Doubtful records are indicated with a question mark (?) before the species name. The valid name may be followed by one or more additional names used for the same taxon. These names can be younger synonyms, preoccupied names, misidentifications, or common misuses in Finnish or international literature. As an example, the following entry lists three additional names used for *Rhamphomyia trilineata*.

*Rhamphomyia trilineata* Zetterstedt, 1859

= *sulcatina* Collin, 1926

= *tibialis* auct. nec Meigen, 1822

= *propinqua* misid.

*Rhamphomyia sulcatina* Collin is a younger synonym of *Rhamphomyia trilineata*. The name *Rhamphomyia tibialis* Meigen was erroneously used for this species by Frey (1956) and others; the genuine *Rhamphomyia tibialis* of Meigen has not been found in the Nordic countries. Frey et al. (1941) misidentified this species as *Rhamphomyia propinqua* de Meijere (in reality a junior synonym name for *Rhamphomyia sulcata* Meigen).

Table 1 lists abbreviations used in the checklist papers.

**Table 1.** Common abbreviations used in the checklist papers.

Abbreviation	Word or term	Interpretation
aff.	*affinis*	affined to, near
auct. nec	*auctorum*, *nec*	wrong interpretation, literally ‘of authors, not’
cf.	*confer*	compare with (may be identical with)
coll.	collective	collective name for sister species not easily separable by morphology
emend.	emendation	an intentional alternative spelling
misid.	misidentified	
nom. dubium	*nomen dubium*	a name with uncertain meaning
nom. nudum	*nomen nudum*	a name without a proper scientific definition
pr.	*prope*	near
preocc.	preoccupied	preoccupied by an older homonymous name
sg.	subgenus	
sp.	species	
suppr.	suppressed	a name made unavailable by an ICZN decision
var.	variety	a described variety

The checklist section is followed by a list of species not included on the checklist for various reasons. Excluded species comprise species recorded only from areas ceded to Russia before 1945, exotic species occasionally imported to Finland by man without locally reproducing populations, records based on misidentifications, etc. The Notes section presents authors’ comments on individual taxa.

## Discussion

Table 2 presents a summary of the results for each family of Diptera recorded from Finland, which includes the number of species known from the country now; in the previous checklist (Hackman 1980a, 1980b, 1980c); and the difference between the two checklists in species counts and as a fraction of the fauna known in 1980.

**Table 2.** Number of species recorded from Finland for each Diptera family. Systematic order follows Marshall (2012).

		Family	Finland 2014	Hackman (1980)	increase in species #	
			species # with and without doubtful records	species # including doubtful records	spp.	%
LOWER DIPTERA (NEMATOCERAN FLIES)						
Tipulomorpha						
	Tipuloidea					
		Cylindrotomidae	7	5	2	40%
		Limoniidae	196	144	52	36%
		Pediciidae	19	13	6	46%
		Tipulidae	114–115	98	16	16%
		Trichoceridae	15–17	13	2	15%
Psychodomorpha						
		Pscyhodidae	61–63	14	47	336%
Ptychopteromorpha						
		Ptychopteridae	7	6	1	17%
Culicomorpha						
	Culicoidea					
		Chaoboridae	8	8	0	0%
		Culicidae	38	37	1	3%
		Dixidae	16	7	9	129%
	Chironomoidea					
		Ceratopogonidae	97	69	28	41%
		Chironomidae	780	411	369	90%
		Simuliidae	56	35	21	60%
		Thaumaleidae	1	0	1	–
Bibionomorpha						
	Anisopodoidea					
		Anisopodidae	7	5	2	40%
	Bibionoidea					
		Bibionidae	17	13	4	31%
		Canthyloscelidae	3	3	0	0%
		Mycetobiidae	1	1	0	0%
		Pachyneuridae	1	1	0	0%
		Scatopsidae	30	26	4	15%
	Sciaroidea					
		Bolitophilidae	21	17	4	24%
		Cecidomyiidae	355–356	136	219	161%
		Diadocidiidae	5	3	2	67%
		Ditomyiidae	2	1	1	100%
		Keroplatidae	46–47	37	9	24%
		Mycetophilidae	691–692	431	260	60%
		Sciaridae	337	207	130	63%
		Sciarosoma	1	0	1	–
BRACHYCERA						
lower Brachycera						
Tabanomorpha						
	Xylophagoidea					
		Xylophagidae	5	4	1	25%
	Rhagionoidea					
		Athericidae	1	1	0	0%
		Rhagionidae	16–17	14	2	14%
	Tabanoidea					
		Tabanidae	38–39	37	1	3%
	Stratiomyioidea					
		Stratiomyidae	29	28	1	4%
		Xylomyidae	1	1	0	0%
Asilomorpha						
	Asiloidea					
		Asilidae	35	35	0	0%
		Bombyliidae	18–19	22	-4	-18%
		Mythicomyiidae	1	1	0	0%
		Scenopinidae	3	2	1	50%
		Therevidae	17	20	-3	-15%
	unplaced in Asilomorpha					
		Acroceridae	5	5	0	0%
	Empidoidea					
		Atelestidae	2	1	1	100%
		Brachystomatidae	4	4	0	0%
		Dolichopodidae	260	219	41	19%
		Hybotidae	143–144	132	11	8%
		Empididae	172	154	18	12%
		Iteaphila group	3	4		-25%
higher Brachycera, Cyclorrhapha						
	Phoroidea					
		Opetiidae	1	1	0	0%
		Lonchopteridae	8	3	5	167%
		Phoridae	224–234	202	22	11%
		Platypezidae	39	20	19	95%
	Syrphoidea					
		Pipunculidae	107	54	53	98%
		Syrphidae	362	271	91	34%
	Conopoidea					
		Conopidae	19	19	0	0%
	Diopsoidea					
		Psilidae	29	26	3	12%
		Tanypezidae	1	1	0	0%
		Strongylophthalmyiidae	2	2	0	0%
		Megamerinidae	1	1	0	0%
	Nerioidea					
		Micropezidae	6	5	1	20%
		Pseudopomyzidae	1	1	0	0%
	Tephritoidea					
		Eurygnathomyiidae	1	1	0	0%
		Lonchaeidae	41–44	37	4	11%
		Neottiophilidae	2	0	2	–
		Pallopteridae	13	10	3	30%
		Piophilidae	15	13	2	15%
		Platystomatidae	2	2	0	0%
		Tephritidae	69	61	8	13%
		Ulidiidae	16	14	2	14%
	Lauxanoidea					
		Chamaemyiidae	27–28	16	11	69%
		Lauxaniidae	45	42	3	7%
	Sciomyzoidea					
		Coelopidae	1	1	0	0%
		Dryomyzidae	5	3	2	67%
		Heterocheilidae	1	1	0	0%
		Phaeomyiidae	2	2	0	0%
		Sciomyzidae	73–74	68	7	10%
		Sepsidae	32	24	9	38%
	Opomyzoidea					
		Agromyzidae	280–281	221	58	26%
		Anthomyzidae	15	12	3	25%
		Asteiidae	6	5	1	20%
		Aulacigastridae	2	1	1	100%
		Clusiidae	12	10	2	20%
		Odiniidae	5	5	0	0%
		Opomyzidae	16	13	3	23%
		Periscelididae	4	3	1	33%
	Carnoidea					
		Acartophthalmidae	3	3	0	0%
		Canacidae	1	1	0	0%
		Carnidae	13	12	1	8%
		Chloropidae	150	91	59	65%
		Milichiidae	12–13	11	2	18%
	Sphaeroceroidea					
		Chyromyidae	4	4	0	0%
		Heleomyzidae	61	53	8	15%
		Sphaeroceridae	118	97	21	22%
	Ephydroidea					
		Braulidae	1	1	0	0%
		Camillidae	3	3	0	0%
		Diastatidae	10	5	5	100%
		Drosophilidae	67	51	16	31%
		Ephydridae	112	106	6	6%
	Hippoboscoidea					
		Hippoboscidae s. lat. incl. Nycteribiidae	12	12	0	0%
	Muscoidea					
		Anthomyiidae	289	177	112	63%
		Fanniidae	61	40	22	55%
		Muscidae	307–309	253	54	21%
		Scathophagidae	85–86	83	2	2%
	Oestroidea					
		Calliphoridae	45	36	9	25%
		Oestridae	8–9	8	0	0%
		Rhiniidae	0–1	1	0	0%
		Rhinophoridae	5	4	1	25%
		Sarcophagidae	64	58	6	10%
		Tachinidae	319	201	118	59%
Nematoceran, total			2932–2942	1741	1191	67%
Brachycera, total			3989–4015	3166	823	26%
Diptera, total			6920–6956	4907	2013	41%

The Diptera has traditionally been split into two suborders, Nematocera and Brachycera. It is now generally agreed that while Brachycera is a monophyletic lineage, Nematocera is not (see Yeates et al. 2007 and references therein). The Brachycera may have evolved from a bibionomorphan ancestor, but this has so far proved difficult to confirm (Lambkin et al. 2013). The systematic order of Table 2 follows Marshall (2012), with one exception: Conopoidea is given superfamily status. Families are listed alphabetically within each superfamily.

Two nematocerous families dominate by absolute numbers of species: the non-biting midges (Chironomidae) with 780 species, and the true fungus gnats (Mycetophilidae, 691 spp.). These two families also show the largest number of new species reported since 1980 (369 and 260 species respectively). Hoverflies (Syrphidae, 362 spp.) is the largest brachyceran family, followed closely by tachinid parasitic flies (Tachinidae, 319 spp.). The largest absolute increases are seen in Tachinidae (118 spp.) and Anthomyiidae (112 spp.). The relative number of moth fly (Psychodidae) species has more than tripled since Hackman’s checklist, mostly due to the work of Jukka Salmela. Among the larger families, major increases are also seen in the Pipunculidae (98% increase), Cecidomyiidae (161%), and Chironomidae (90%).

Most Diptera families show a decreasing trend in the number of species with latitude in Europe, but some are genuinely more diverse in the boreal zone (see Kjærandsen et al. 2007). The number of species known from Finland compares favorably with the results from neighboring countries (see Table 3). With 6920 species, Finland has the highest reported Diptera diversity among the Nordic countries. Only Germany (9544 species, Schumann 2009) and the Czech Republic (7917 spp., Jedlička et al. 2009) of all the North and Central European countries report significantly higher national Diptera faunas. This must, however, be at least partially attributed to differences between surveying intensity and the history of various countries. Most countries have only a few (if any) active dipterologists. Up until the last decade, access to the taxonomic literature required for Diptera identification was restricted to those working in close cooperation with major taxonomic institutes.

**Table 3.** Recent Diptera checklists from north and central European countries. The species numbers of nematoceran, brachyceran, and all Diptera are listed for each country. Species of doubtful occurrence are not included in the counts.

Country	Reference	# of species recorded		
		nematoceran	brachyceran	all Diptera
*Nordic countries*				
Finland		2932	3989	6920
Sweden	Cederberg et al. (2010)	2260	4410	6670
Norway	Gammelmo et al. (2010)	1936	3116	5052
Denmark	Petersen and Meier (2001)	1327	3034	4361
*Baltic countries*				
Latvia	Karpa (2008)	–	–	1654
Lithuania	Pakalniškis et al. (2006)	–	–	3311
*Western and Central Europe*				
Poland	Zatwarnicki (2001)	–	–	6721
Great Britain	Chandler (1998, 2013a)	2844	4210	7054
Ireland	Chandler et al. (2008), Chandler (2013b)	1479	1907	3386
the Netherlands	Beuk (2002)	1640	3324	4964
Belgium	Grootaert et al. (1991)	–	–	4474
Germany	Schumann et al. (1999), Schumann (2009)	–	–	9544
Switzerland	Merz et al. (1998, 2006)	–	–	6813
Czech Republic	Jedlička et al. (2009)	5162	2755	7917
Slovakia	Jedlička et al. (2009)	4460	2380	6840
Hungary	Papp et al. (2001)	~1460	~4090	~5550

**Figure 1.**
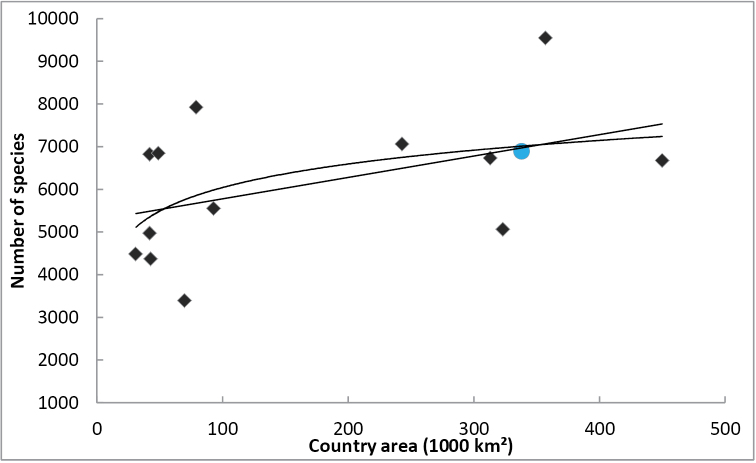
The species-area curve for some Northern, Western and Central European countries based on Table 3, excluding the Baltic states (see also Fig. 1 in Petersen and Meier 2001). Data for Finland is marked by a circle, other countries by rectangles. The two lines show linear and logarithmic least-squares fit models for the data.

**Figure 2.**
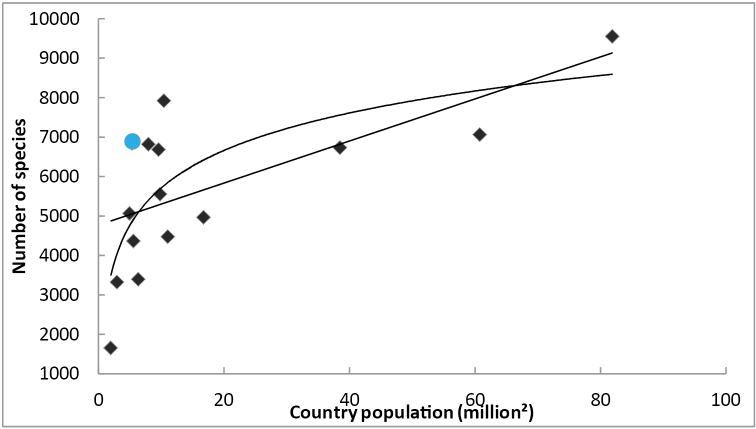
The species-population curve for some Northern, Western and Central European countries based on Table 3. Data for Finland is marked by a circle, other countries by rectangles. The two lines show linear and logarithmic least-squares fit models for the data.

The number of species present in an area does usually increase with the size of the area (see Ulrich and Buszko 2003 for an example involving European insects). Peterson and Meier (2001) presented a species-area curve (of type IV, see Scheiner 2003) for Diptera species of European countries. Figure 1, based on Table 3, is an updated version of their figure. Two simple models (linear and logarithmic) were fitted to the data using least squares fits. One should, however, not draw too many conclusions from these models: the true number of Diptera species present in each country is likely to be significantly larger than the known number of species. Furthermore, large – or at least populous countries—countries are more likely to harbor dipterists, who contribute faunistic records (see Figure 2).

**Figure 3.**
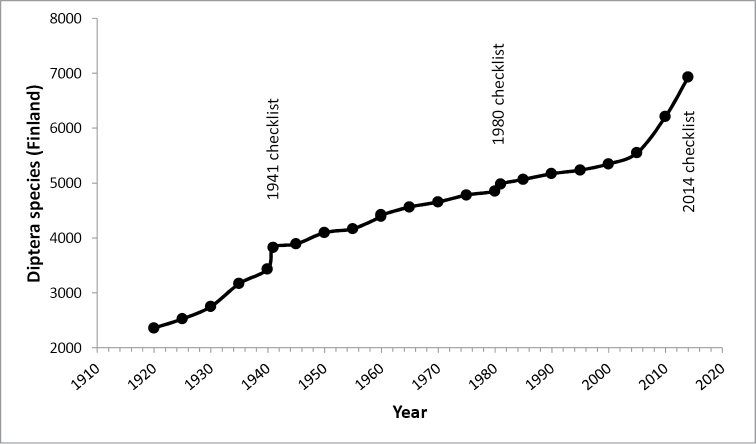
Total number of Diptera species known from Finland over time based on the 5-year summaries by Hellén and Silfverberg and the three checklists of Finnish Diptera. The publishing years of the three checklists are noted.

From an accumulation curve of new records over the last century (Figure 3), one can immediately see that the Finnish fauna is far from completely known. If almost all species present in the country had already been found, one would expect the rate of new records to diminish (but not fall to zero as genuine expansive species would still occasionally arrive). What has actually happened is the opposite; the rate of new discoveries has increased in the last decade. The number of species found as new to Finland during each five-year period seems to reflect the number of active dipterologists in the country. To a degree, this may represent a backlog from the 1980s and 1990s, when the number of dipterists collecting in Finland and publishing new faunistic records was smaller than during the previous 70 years, or in the 21^st^ century. Still, one could predict that the true number of Diptera species present in Finland may be well over eight thousand species, including hundreds of still unknown and undescribed species (Fontaine et al. 2012).

## Supplementary materials

Supplementary material 1 Electronic appendix 1: CVS taxon file Data type: (measurement/occurence/multimedia/etc.) Explanation note: This file is encoded as UTF-8 text with a semicolon as the field separator. In Excel, the CSV file is best opened using the Data/From Text functionality. The data is formatted as Darwin Core (Darwin Core Task Group 2009) compliant. The following fields are provided: taxonID [Fauna Europaea URN if available], scientificName, taxonRank, higherClassification, order, family, genus, subgenus, specificEpithet, scientificNameAuthorship, identificationQualifier, identificationRemarks, bibliographicCitation, taxonConceptID. This dataset is made available under the Open Database License (http://opendatacommons.org/licenses/odbl/1.0/). The Open Database License (ODbL) is a license agreement intended to allow users to freely share, modify, and use this Dataset while maintaining this same freedom for others, provided that the original source and author(s) are credited.Jere Kahanpää

Supplementary material 2 Electronic appendix 2: PDF literature references file Data type: References list. Explanation note: A PDF file with additional references to first published records is also provided. Cross-references to Hackman’s 1980 checklist are included. This file includes an index to genus- and species-level names. This dataset is made available under the Open Database License (http://opendatacommons.org/licenses/odbl/1.0/). The Open Database License (ODbL) is a license agreement intended to allow users to freely share, modify, and use this Dataset while maintaining this same freedom for others, provided that the original source and author(s) are credited.Jere Kahanpää
