# Supplementary material for: Checklist of the Diptera (Insecta) of Finland: an introduction and a summary of results
Source: Zookeys. 2014 Sep 19;(441):1–20. doi: 10.3897/zookeys.441.7620 (PMC4200444; doi:10.3897/zookeys.441.7620)
Supplement: Supplementary material 2 — Electronic appendix 2: PDF literature references file [file zookeys-441-001-s002.pdf]

Checklist of the Diptera of Finland (Insecta). A bibliographic appendix

Jere Kahanpää (editor)

August 29, 2014

An appendix to: *Kahanpää J, Salmela J (Eds) 2014: A Checklist of the Diptera of Finland. ZooKeys XX*

Format: full taxon name [faunistic references]

This appendix provides some references to the previous checklist of Finnish Diptera (ie. Hackman 1980) and published scientific literature relevant for the Finnish fauna from a faunistic point of view. It is definitely NOT a comprehensive catalogue of references for the Finnish Diptera. References are made to

- The previous checklist (Hackman 1980)
- Publications reporting species new for the fauna (∼1965 onwards)
- Publications with corrections to older erroneous records (∼1965 onwards)
- Other papers relevant to understanding the use of a species name in Finland.

TRICHOCERIDAE Rondani, 1841

Supporting references for *Salmela J, Petrašiunas A (2014) Checklist of the infraorder Tipulomorpha (Trichoceridae, Tipuloidea) (Diptera) of Finland. In: Kahanpää J, Salmela J (Eds) Checklist of the Diptera of Finland. ZooKeys @@: @-@. doi: 10.3897/ zookeys.??.7533*

CLADONEURA Scudder, 1894
= ***Diazosma*** Bergroth, 1913
? *Cladoneura hirtipenne* (Siebke, 1863) [Hackman 1980]
TRICHOCERA Meigen, 1803
sg. ***Metatrichocera*** Dahl, 1966
Trichocera gigantea (Dahl, 1967) [Dahl 1968, Hackman 1980]
Trichocera lutea Becher, 1886 [Dahl and Alexander 1976]
Trichocera mackenziei (Dahl, 1967) [Dahl 1968, Hackman 1980]
sg. ***Saltrichocera*** Krzeminska, 2002
Trichocera annulata Meigen, 1818 [Dahl and Alexander 1976]
? *Trichocera arctica* Lundström, 1915 [Dahl 1968, Hackman 1980]
Trichocera implicata Dahl, 1976 [Dahl and Alexander 1976]
Trichocera maculipennis Meigen, 1818 [Frey et al. 1941, Hackman 1980]
Trichocera parva Meigen, 1804 [Frey et al. 1941, Hackman 1980]
Trichocera regelationis (Linnaeus, 1758) [Frey et al. 1941, Hackman 1980]
Trichocera rufescens Edwards, 1921 [Frey et al. 1941]
Trichocera saltator (Harris, 1776) [Hackman 1980]
= *T. fuscata* Meigen, 1818 [Frey et al. 1941]
Trichocera sparsa Starý & Martinovský, 1996 [Krzemiska 2013]
sg. ***Trichocera*** Meigen, 1803
Trichocera hiemalis (De Geer, 1776) [Frey et al. 1941, Hackman 1980]
Trichocera inexplorata (Dahl, 1967) [Dahl 1968, Hackman 1980]
Trichocera major Edwards, 1921 [Frey et al. 1941, Hackman 1980]
Trichocera sibirica Edwards, 1920 [Dahl 1968, Hackman 1980]

Excluded species

Trichocera japonica Matsumura, 1915 [Dahl 1968, Hackman 1980, Dahl 1992]

PEDICIIDAE Osten Sacken, 1860

Supporting references for *Salmela J, Petrašiunas A (2014) Checklist of the infraorder Tipulomorpha (Trichoceridae, Tipuloidea) (Diptera) of Finland. In: Kahanpää J, Salmela J (Eds) Checklist of the Diptera of Finland. ZooKeys @@: @-@. doi: 10.3897/ zookeys.??.7533*

PEDICIINAE Osten Sacken, 1860
DICRANOTA Zetterstedt, 1838
sg. ***Dicranota*** Zetterstedt, 1838
Dicranota bimaculata (Schummel, 1829) [Hackman 1980]
Dicranota crassicauda Tjeder, 1972 [Hackman 1980]
Dicranota guerini Zetterstedt, 1838 [Hackman 1980]
sg. ***Paradicranota*** Alexander, 1934
Dicranota gracilipes Wahlgren, 1905 [Salmela 2001b]
Dicranota pavida (Haliday, 1833) [Salmela 2001a]
Dicranota robusta Lundström, 1912 [Hackman 1980]
Dicranota subtilis Loew, 1871 [Hackman 1980]
sg. ***Rhaphidolabis*** Osten Sacken, 1869
Dicranota exclusa (Walker, 1848) [Hackman 1980]
PEDICIA Latreille, 1809
sg. ***Crunobia*** Kolenati, 1859
Pedicia straminea (Meigen, 1838) [Mannheims 1964, Hackman 1980]
sg. ***Pedicia*** Latreille, 1809
Pedicia rivosa (Linnaeus, 1758) [Hackman 1980]
TRICYPHONA Zetterstedt, 1837
sg. ***Tricyphona*** Zetterstedt, 1837
Tricyphona immaculata (Meigen, 1804) [Hackman 1980]
Tricyphona livida Madarassy, 1881 [Siitonen 1983a, Salmela 2011]
Tricyphona schummeli Edwards, 1921 [Hackman 1980]
Tricyphona unicolor (Schummel, 1829) [Hackman 1980]
ULINAE Alexander, 1920
ULA Haliday, 1833
sg. ***Ula*** Haliday, 1833
Ula bolitophila Loew, 1869 [Rautio 1985, Salmela 2011]
Ula kiushiuensis Loew, 1869 [Salmela and Piirainen 2003]
Ula mixta Starý, 1983 [Starý and Salmela 2004]
Ula mollissima Haliday, 1833 [Hackman 1980]
= *U. inconclusa* (Walker, 1856) [Hackman 1968b]
Ula sylvatica (Meigen, 1818) [Hackman 1980]

Excluded species

Pedicia arctica Frey, 1921 [Frey 1921]

LIMONIIDAE Speiser, 1909

Supporting references for *Salmela J, Petrašiunas A (2014) Checklist of the infraorder Tipulomorpha (Trichoceridae, Tipuloidea) (Diptera) of Finland. In: Kahanpää J, Salmela J (Eds) Checklist of the Diptera of Finland. ZooKeys @@: @-@. doi: 10.3897/ zookeys.??.7533*

CHIONEINAE Rondani, 1841
ARCTOCONOPA Alexander, 1955
Arctoconopa forcipata (Lundström, 1915) [Hackman 1980]
Arctoconopa obscuripes (Zetterstedt, 1851) [Hackman 1980]
Arctoconopa zonata (Zetterstedt, 1851) [Hackman 1980]
CHEILOTRICHIA Rossi, 1848
sg. ***Cheilotrichia*** Rossi, 1848
Cheilotrichia imbuta (Meigen, 1818) [Hackman 1980]
sg. ***Empeda*** Osten Sacken, 1869
Cheilotrichia areolata (Lundström, 1912) [Hackman 1980]
Cheilotrichia cinerascens (Meigen, 1804) [Hackman 1980]
= *C. nubila* (Schummel, 1829) [Hackman 1980]
Cheilotrichia neglecta (Lackschewitz, 1927) [Rautio 1987, Salmela 2011]
CHIONEA Dalman, 1816
sg. ***Chionea*** Dalman, 1816
Chionea araneoides Dalman, 1816 [Hackman 1980]
Chionea crassipes Boheman, 1846 [Hackman 1980]
= *C. brevirostris* Tahvonen, 1932
= *C. minuta* Tahvonen, 1932
sg. ***Sphaeconophilus*** Becker, 1912
Chionea lutescens Lundström, 1907 [Hackman 1980]
CRYPTERIA Bergroth, 1913
Crypteria limnophiloides Bergroth, 1913 [Hackman 1980]
ERIOCONOPA Stary, 1976
Erioconopa diuturna (Walker, 1848) [Mannheims 1965, Hackman 1980]
Erioconopa trivialis (Meigen, 1818) [Hackman 1980]
ERIOPTERA Meigen, 1803
sg. ***Erioptera*** Meigen, 1803
Erioptera beckeri Kuntze, 1914 [Hackman 1980]
= *E. fuscipennis* misid. [Hackman 1980]
Erioptera divisa (Walker, 1848) [Hackman 1980]
Erioptera flavata (Westhoff, 1882) [Salmela 2011]
= *E. gemina* Tjeder, 1967 [Hackman 1980]
Erioptera griseipennis Meigen, 1838 [Hackman 1980]
Erioptera lutea Meigen, 1804 [Hackman 1980]
Erioptera nielseni Meijere, 1921 [Hackman 1980]
Erioptera pederi Tjeder, 1969 [Salmela 2001a]
Erioptera sordida Zetterstedt, 1838 [Hackman 1980]
Erioptera squalida Loew, 1871 [Hackman 1980]
Erioptera tordi Tjeder, 1973 [Salmela 2011]
GNOPHOMYIA Osten Sacken, 1860
Gnophomyia acheron Alexander, 1950 [Starý and Salmela 2004]
Gnophomyia lugubris (Zetterstedt, 1838) [Rautio 1985]

Gnophomyia viridipennis (Gimmerthal, 1847) [Hancock 2008]
GONEMPEDA Alexander, 1924
Gonempeda flava (Schummel, 1829)
GONOMYIA Meigen, 1818
sg. ***Gonomyia*** Meigen, 1818
Gonomyia abscondita Lackschewitz, 1935 [Salmela 2011]
= *G. lucidula* misid.
Gonomyia bifida Tonnoir, 1920 [Mannheims 1965, Hackman 1980]
Gonomyia dentata Meijere, 1920 [Hackman 1980]
Gonomyia simplex Tonnoir, 1920 [Hackman 1980]
Gonomyia stackelbergi Lackschewitz, 1935 [Hackman 1980]
Gonomyia tenella (Meigen, 1818) [Hackman 1980]
sg. ***Teuchogonomyia*** Alexander, 1968
Gonomyia edwardsi Lackschewitz, 1925 [Mannheims 1965, Hackman 1980]
HOPLOLABIS Osten Sacken, 1869
sg. ***Parilisia*** Savchenko, 1976
Hoplolabis areolata (Siebke, 1872) [Hackman 1980]
Hoplolabis vicina (Tonnoir, 1920) [Mannheims 1965, Hackman 1980]
MOLOPHILUS Curtis, 1833
sg. ***Molophilus*** Curtis, 1833
Molophilus appendiculatus (Staeger, 1840) [Hackman 1980]
Molophilus ater (Meigen, 1804) [Hackman 1980]
Molophilus bifidus Goetghebuer, 1920 [Salmela 2001a]
Molophilus bihamatus Meijere, 1918 [Salmela 2001a]
Molophilus cinereifrons Meijere, 1920 [Mannheims 1965, Hackman 1980]
Molophilus corniger Meijere, 1920 [Salmela 2001a]
Molophilus crassipygus Meijere, 1918 [Salmela 2011]
= *M. ochrescens* Edwards, 1938 [Siitonen 1983b]
Molophilus flavus Goetghebuer, 1920 [Hackman 1980]
Molophilus griseus (Meigen, 1804) [Hackman 1980]
Molophilus medius Meijere, 1918 [Mannheims 1965, Hackman 1980]
Molophilus obscurus (Meigen, 1818) [Hackman 1980]
Molophilus occultus Meijere, 1918 [Salmela 2011]
Molophilus ochraceus (Meigen, 1818) [Hackman 1980]
Molophilus propinquus (Egger, 1863) [Hackman 1980]
Molophilus pullus Lackschewitz, 1927 [Salmela et al. 2007a]
NEOLIMNOPHILA Alexander, 1920
Neolimnophila carteri (Tonnoir, 1921) [Hackman 1980]
Neolimnophila placida (Meigen, 1830) [Hackman 1980]
ORMOSIA Rondani, 1856
sg. ***Oreophila*** Lackschewitz, 1935
Ormosia sootryeni (Lackschewitz, 1935)
sg. ***Ormosia*** Rondani, 1856
Ormosia brevinervis (Lundström, 1907) [Hackman 1980]
Ormosia clavata (Tonnoir, 1920) [Hackman 1980]
Ormosia depilata Edwards, 1938 [Hackman 1980]
Ormosia fascipennis (Zetterstedt, 1838) [Hackman 1980]
Ormosia hederae (Curtis, 1835)
Ormosia lineata (Meigen, 1804) [Mee 1961a, Hackman 1980]
Ormosia loxia Starý, 1983 [Salmela et al. 2007a]

Ormosia pseudosimilis (Lundström, 1912) [Mee 1961a, Hackman 1980]
Ormosia ruficauda (Zetterstedt, 1838) [Tjeder 1972, Hackman 1980]
Ormosia staegeriana Alexander, 1953 [Hackman 1980]
PHYLLOLABIS Osten Sacken, 1877
Phyllolabis macroura (Siebke, 1863) [Hackman 1980]
RHABDOMASTIX Skuse, 1890
Rhabdomastix borealis Alexander, 1924 [Salmela 2011]
Rhabdomastix laeta (Loew, 1873) [Hackman 1980]
Rhabdomastix parva (Siebke, 1863) [Hackman 1968b, 1980]
RHYPHOLOPHUS Kolenati, 1860
Rhypholophus haemorrhoidalis (Zetterstedt, 1838) [Hackman 1980]
Rhypholophus varius (Meigen, 1818) [Mannheims 1965, Hackman 1980]
SCLEROPROCTA Edwards, 1938
Scleroprocta pentagonalis (Loew, 1973) [Salmela 2011]
Scleroprocta sororcula (Zetterstedt, 1851) [Hackman 1980]
= *S. danica* (Nielsen, 1923) [Hac 1969]
SYMPLECTA Meigen, 1830
sg. ***Psiloconopa*** Zetterstedt, 1838
Symplecta lindrothi (Tjeder, 1955) [Salmela 2011]
Symplecta meigeni (Zetterstedt, 1838) [Hackman 1980]
Symplecta stictica (Meigen, 1818) [Hackman 1980]
sg. ***Symplecta*** Meigen, 1830
Symplecta chosenensis (Alexander, 1940) [Kahanpää and Salmela 2007]
Symplecta hybrida (Meigen, 1804) [Hackman 1980]
Symplecta mabelana (Alexander, 1955) [Salmela 2011]
Symplecta scotica (Edwards, 1938) [Salmela 2011]
sg. ***Trimicra*** Osten Sacken, 1861
Symplecta pilipes (Fabricius, 1787) [Hackman 1980]
TASIOCERA Skuse, 1890
sg. ***Dasymolophilus*** Goetghebuer, 1920
Tasiocera exigua Savchenko, 1973 [Salmela et al. 2007a]
Tasiocera fuscescens (Lackschewitz, 1940) [Salmela 2011]
Tasiocera murina (Meigen, 1818) [Hackman 1980]
LIMNOPHILINAE Bigot, 1854
ADELPHOMYIA Bergroth, 1891
Adelphomyia punctum (Meigen, 1818) [Salmela and Härmä 2004]
AUSTROLIMNOPHILA Alexander, 1920
sg. ***Archilimnophila*** Alexander, 1934
Austrolimnophila harperi (Alexander, 1926) [Salmela 2011]
Austrolimnophila unica (Osten Sacken, 1869) [Hackman 1980]
DICRANOPHRAGMA Osten Sacken, 1860
sg. ***Brachylimnophila*** Alexander, 1966
Dicranophragma adjunctum (Walker, 1848)
Dicranophragma separatum (Walker, 1848) [Salmela 2011]
= *D. nemorale* misid. [Hackman 1980]
= *D. leucophaea* misid. [Hackman 1980]
ELOEOPHILA Rondani, 1856
Eloeophila maculata (Meigen, 1804) [Hackman 1980]
Eloeophila mundata (Loew, 1971)
Eloeophila submarmorata (Verrall, 1887) [Hackman 1980]

Eloeophila trimaculata (Zetterstedt, 1838) [Hackman 1980]
Eloeophila verralli (Bergroth, 1912) [Mannheims 1965, Hackman 1980]
EPIPHRAGMA Osten Sacken, 1860
sg. ***Epiphragma*** Osten Sacken, 1860
Epiphragma ocellare (Linnaeus, 1761) [Hackman 1980]
EUPHYLIDOREA Alexander, 1972
Euphylidorea dispar (Meigen, 1818) [Hackman 1980]
= *E. lineola* misid. [Hackman 1980]
Euphylidorea meigeni (Verrall, 1886) [Hackman 1980]
Euphylidorea phaeostigma (Schummel, 1829) [Hackman 1980]
EUTONIA van der Wulp, 1874
Eutonia barbipes (Meigen, 1804) [Hackman 1980]
HEXATOMA Latreille, 1809
sg. ***Hexatoma*** Latreille, 1809
Hexatoma fuscipennis (Curtis, 1836) [Hackman 1980]
= *H. nubeculosa* misid. [Hackman 1980]
IDIOPTERA Macquart, 1834
Idioptera linnei Oosterbroek, 1992 [Salmela 2011]
= *I. fasciata* (Linnaeus, 1767) preocc.[Hackman 1980]
Idioptera pulchella (Meigen, 1830) [Hackman 1980]
= *I. pulchella* var. macropteryx (Tjeder, 1955) [Mannheims 1965, Hackman 1980]
LIMNOPHILA Macquart, 1834
sg. ***Limnophila*** Macquart, 1834
Limnophila pictipennis (Meigen, 1818) [Mannheims 1965, Salmela 2011]
Limnophila schranki Oosterbroek, 1992 [Salmela 2011]
= *L. punctata* (Schrank, 1781) [Hackman 1980]
NEOLIMNOMYIA Seguy, 1937
sg. ***Neolimnomyia*** Seguy, 1937
Neolimnomyia batava (Edwards, 1938) [Salmela et al. 2007a]
PARADELPHOMYIA Alexander, 1936
sg. ***Oxyrhiza*** de Meijere, 1946
Paradelphomyia fuscula (Loew, 1873) [Kro 1936, Salmela 2001a]
Paradelphomyia nigrina (Lackschewitz, 1940) [Salmela 2008]
PHYLIDOREA Bigot, 1854
sg. ***Macrolabina*** Savchenko, 1986
Phylidorea nigronotata (Siebke, 1870) [Hackman 1980]
sg. ***Paraphylidorea*** Savchenko, 1986
Phylidorea fulvonervosa (Schummel, 1829) [Hackman 1980]
= *Euphylidorea lineola* misid. [Hackman 1980]
sg. ***Phylidorea*** Bigot, 1854
Phylidorea abdominalis (Staeger, 1840) [Hackman 1980]
Phylidorea bicolor (Meigen, 1804) [Hackman 1980]
Phylidorea ferruginea (Meigen, 1818) [Hackman 1980]
Phylidorea heterogyna (Bergroth, 1913) [Mannheims 1965, Hackman 1980]
Phylidorea longicornis (Schummel, 1829) [Salmela 2011]
= *P. glabricula* (Meigen, 1830) [Hackman 1980]
Phylidorea nervosa (Schummel, 1829) [Salmela 2011]
= *P. nigricollis* (Meigen, 1830) [Mannheims 1965, Hackman 1980]
Phylidorea squalens (Zetterstedt, 1838) [Hackman 1980]
Phylidorea umbrarum (Krogerus, 1937) [Hackman 1980]

PILARIA Sintenis, 1889
Pilaria decolor (Zetterstedt, 1851) [Mannheims 1965, Hackman 1980]
Pilaria discicollis (Meigen, 1818) [Hackman 1980]
Pilaria meridiana (Staeger, 1840) [Hackman 1980]
Pilaria nigropunctata (Agrell, 1945) [Salmela 2011]
= *P. fuscipennis* misid. [Mannheims 1965, Hackman 1980]
Pilaria scutellata (Staeger, 1840) [Mannheims 1965, Hackman 1980]
PSEUDOLIMNOPHILA Alexander, 1919
sg. ***Pseudolimnophila*** Alexander, 1919
Pseudolimnophila lucorum (Meigen, 1818) [Hackman 1980]
LIMONIINAE Speiser, 1909
ACHYROLIMONIA Alexander, 1965
Achyrolimonia decemmaculata (Loew, 1873) [Hackman 1980]
ANTOCHA Osten Sacken, 1860
sg. ***Antocha*** Osten Sacken, 1860
Antocha vitripennis (Meigen, 1830) [Salmela 2011]
ATYPOPHTHALMUS Brunetti, 1911
sg. ***Atypophthalmus*** Brunetti, 1911
Atypophthalmus inustus (Meigen, 1818) [Hackman 1980]
DICRANOMYIA Stephens, 1829
sg. ***Dicranomyia*** Stephens, 1829
Dicranomyia aperta Wahlgren, 1904 [Salmela 2008]
Dicranomyia autumnalis (Staeger, 1840) [Hackman 1980]
Dicranomyia consimilis (Zetterstedt, 1838) [Hackman 1980]
Dicranomyia didyma (Meigen, 1804) [Hackman 1980]
Dicranomyia distendens Lundström, 1912 [Hackman 1980]
Dicranomyia frontalis (Staeger, 1840) [Hackman 1980]
Dicranomyia halterata Osten Sacken, 1869 [Hackman 1980]
Dicranomyia handlirschi Lackschewitz, 1928 [Salmela 2001b]
Dicranomyia hyalinata (Zetterstedt, 1851) [Hackman 1980]
Dicranomyia longipennis (Schummel, 1829) [Hackman 1980]
Dicranomyia mitis (Meigen, 1830) [Hackman 1980]
Dicranomyia modesta (Meigen, 1818) [Hackman 1980]
Dicranomyia moniliformis Doane, 1900 [Salmela 2008]
Dicranomyia omissinervis Meijere, 1918 [Salmela 2008]
Dicranomyia patens Lundström, 1907 [Hackman 1980]
Dicranomyia radegasti Starý, 1993 [Salmela 2011]
Dicranomyia sera (Walker, 1848) [Hackman 1968b, 1980]
Dicranomyia terraenovae Alexander, 1920 [Salmela 2011]
Dicranomyia ventralis (Schummel, 1829) [Hackman 1980]
Dicranomyia zernyi Lackschewitz, 1928 [Salmela 2011]
sg. ***Glochina*** Meigen, 1830
Dicranomyia liberta Osten Sacken, 1860 [Salmela 2011]
Dicranomyia tristis (Schummel, 1829) [Hackman 1980]
= *D. subtristis* Alexander, 1924 [Hackman 1980]
= *D. schineri* misid.
= *D. schineriana* misid.[Geiger 1985]
sg. ***Idiopyga*** Savchenko, 1987
Dicranomyia danica Kuntze, 1919 [Hackman 1980]
Dicranomyia esbeni (Nielsen, 1940) [Salmela 2011]

Dicranomyia halterella Edwards, 1921 [Hackman 1980]
Dicranomyia intricata Alexander, 1927 [Salmela 2011]
Dicranomyia klefbecki (Tjeder, 1941)
Dicranomyia lulensis (Tjeder, 1969) [Salmela 2008]
Dicranomyia magnicauda Lundström, 1912 [Hackman 1980]
Dicranomyia murina (Zetterstedt, 1851) [Salmela 2011]
Dicranomyia ponojensis Lundström, 1912 [Hackman 1980]
Dicranomyia stigmatica (Meigen, 1830) [Hackman 1980]
sg. ***Melanolimonia*** Alexander, 1965
Dicranomyia caledonica Edwards, 1926 [Hackman 1980]
Dicranomyia morio (Fabricius, 1787) [Hackman 1980]
Dicranomyia occidua Edwards, 1926 [Viramo 1992]
Dicranomyia rufiventris (Strobl, 1901) [Hackman 1980]
Dicranomyia stylifera Lackschewitz, 1928 [Hackman 1980]
sg. ***Numantia*** Bigot, 1854
Dicranomyia fusca (Meigen, 1804) [Salmela 2001a]
DICRANOPTYCHA Osten Sacken, 1860
Dicranoptycha cinerascens (Meigen, 1818) [Hackman 1980]
Dicranoptycha fuscescens (Schummel, 1829) [Hackman 1980]
DISCOBOLA Osten Sacken, 1865
Discobola annulata (Linnaeus, 1758) [Hackman 1980]
Discobola caesarea (Osten Sacken, 1854) [Hackman 1980]
ELEPHANTOMYIA Osten Sacken, 1860
sg. ***Elephantomyia*** Osten Sacken, 1860
Elephantomyia edwardsi Lackschewitz, 1932 [Valkeila 1959, Hackman 1982, Salmela 2011]
Elephantomyia krivosheinae Savchenko, 1976 [Polevoi 2001a]
HELIUS Lepeletier & Serville, 1828
sg. ***Helius*** Lepeletier & Serville, 1828
Helius flavus (Walker, 1856) [Salmela 2002]
Helius longirostris (Meigen, 1818) [Salmela et al. 2007a]
Helius pallirostris Edwards, 1921 [Salmela 2011]
LIBNOTES Westwood, 1876
sg. ***Afrolimonia*** Alexander, 1965
Libnotes ladogensis (Lackschewitz, 1940) [Polevoi 2001a]
LIMONIA Meigen, 1803
sg. ***Limnobia*** Meigen, 18018
Limonia badia (Walker, 1848) [Starý and Salmela 2004]
Limonia flavipes (Fabricius, 1787) [Hackman 1980]
Limonia macrostigma (Schummel, 1829) [Hackman 1980]
Limonia maculicosta (Coquillett, 1905) [Siitonen 1983a, Salmela 2011]
Limonia messaurea Mendl, 1971 [Salmela 2011]
Limonia nubeculosa Meigen, 1804 [Hackman 1968b, 1980]
Limonia phragmitidis (Schrank, 1781) [Salmela 2011]
= *L. tripunctata* Fabricius, 1781 [Hackman 1980]
Limonia stigma (Meigen, 1818) [Mannheims 1965, Hackman 1980]
Limonia sylvicola (Schummel, 1829) [Hackman 1980]
Limonia trivittata (Schummel, 1829) [Hackman 1980]
LIPSOTHRIX Loew, 1873
Lipsothrix ecucullata Edwards, 1938 [Starý and Salmela 2004]

Lipsothrix errans (Walker, 1848) [Salmela 2011]
METALIMNOBIA Matsumura, 1911
sg. ***Metalimnobia*** Matsumura, 1911
Metalimnobia bifasciata (Schrank, 1781) [Hackman 1980]
Metalimnobia charlesi Salmela & Starý, 2008 [Salmela and Starý 2009]
Metalimnobia quadrimaculata (Linnaeus, 1761) [Hackman 1980]
Metalimnobia quadrinotata (Meigen, 1818) [Hackman 1980]
Metalimnobia tenua Savchenko, 1976 [Salmela et al. 2007a]
Metalimnobia zetterstedti (Tjeder, 1968) [Hackman 1980]
= *M. elegans* Zetterstedt, 1838 preocc.[Mannheims 1964]
NEOLIMONIA Alexander, 1964
Neolimonia dumetorum (Meigen, 1804) [Hackman 1980]
ORIMARGA Osten Sacken, 1869
sg. ***Orimarga*** Osten Sacken, 1869
Orimarga attenuata (Walker, 1848) [Hackman 1980]
Orimarga juvenilis (Zetterstedt, 1851) [Salmela 2011]
RHIPIDIA Meigen, 1818
sg. ***Rhipidia*** Meigen, 1818
Rhipidia maculata Meigen, 1818 [Salmela 2011]
= *R. duplicata* (Doane, 1900) [Hackman 1980]
Rhipidia uniseriata Schiner, 1864 [Hackman 1980]

Excluded species

Arctoconopa quadrivittata (Siebke, 1872) [Savchenko et al. 1992, Salmela 2011]

Dicranomyia chorea (Meigen, 1818) [Hackman 1980]
Dicranomyia ornata (Meigen, 1818) [Geiger 1986, Oosterbroek 2011]
Ormosia nodulosa (Macquart, 1826) [Krogerus 1960, Salmela 2011]

CYLINDROTOMIDAE Schiner, 1863

Supporting references for *Salmela J, Petrašiunas A (2014) Checklist of the infraorder Tipulomorpha (Trichoceridae, Tipuloidea) (Diptera) of Finland. In: Kahanpää J, Salmela J (Eds) Checklist of the Diptera of Finland. ZooKeys @@: @-@. doi: 10.3897/ zookeys.??.7533*

CYLINDROTOMA Macquart, 1834
Cylindrotoma borealis Peus, 1952 [Salmela and Autio 2007b]
Cylindrotoma distinctissima (Meigen, 1818) [Hackman 1980]
Cylindrotoma nigriventris Loew, 1849 [Hackman 1980]
DIOGMA Edwards, 1938
Diogma caudata Takahashi, 1960 [Siitonen 1984]
Diogma glabrata (Meigen, 1818) [Hackman 1980]
PHALACROCERA Schiner, 1863
Phalacrocera replicata (Linnaeus, 1758) [Hackman 1980]
TRIOGMA Schiner, 1863
Triogma trisulcata (Schummel, 1829) [Hackman 1980]

TIPULIDAE Latreille, 1802

Supporting references for *Salmela J, Petrašiunas A (2014) Checklist of the infraorder Tipulomorpha (Trichoceridae, Tipuloidea) (Diptera) of Finland. In: Kahanpää J, Salmela J (Eds) Checklist of the Diptera of Finland. ZooKeys @@: @-@. doi: 10.3897/ zookeys.??.7533*

CTENOPHORINAE Osten Sacken, 1887
tribe Ctenophorini Osten Sacken, 1887
CTENOPHORA Meigen, 1803
sg. ***Ctenophora*** Meigen, 1803
Ctenophora flaveolata (Fabricius, 1794) [Hackman 1980]
Ctenophora guttata Meigen, 1818 [Hackman 1980]
Ctenophora nigriceps (Tjeder, 1949) [Salmela 2011]
Ctenophora pectinicornis (Linnaeus, 1758) [Hackman 1980]
DICTENIDIA Brulle, 1833
Dictenidia bimaculata (Linnaeus, 1761) [Hackman 1980]
PHOROCTENIA Coquillett, 1910
Phoroctenia vittata (Meigen, 1830) [Hackman 1980]
tribe Tanypterini Savchenko, 1966
TANYPTERA Latreille, 1804
sg. ***Tanyptera*** Latreille, 1804
Tanyptera atrata (Linnaeus, 1758) [Hackman 1980]
Tanyptera nigricornis (Meigen, 1818) [Hackman 1980]
DOLICHOPEZINAE Osten Sacken, 1887
DOLICHOPEZA Curtis, 1825
sg. ***Dolichopeza*** Curtis, 1825
Dolichopeza albipes (Strom, 1768) [Hackman 1980]
Dolichopeza bifida Osterbrook & Lantsov, 2011 [Salmela 2011]
= *D. nitida* of authors[Salmela 2008]
TIPULINAE Latreille, 1802
tribe Prionocerini Savchenko, 1966
PRIONOCERA Loew, 1844
Prionocera abscondita Lackschewitz, 1933 [Salmela 2008]
Prionocera chosenicola Alexander, 1945 [Salmela 2011]
= *P. dimidiata* misid.
= *P. absentiva* misid. [Rautio 1985]
Prionocera pubescens Loew, 1844 [Hackman 1980]
Prionocera recta Tjeder, 1948 [Mannheims 1967, Hackman 1980]
= *P. lapponica* Tjeder, 1948 [Rautio 1985]
= *P. lackschewitzi* Mannheims, 1951 [Hackman 1980, Brodo 1987]
Prionocera ringdahli Tjeder, 1948 [Hackman 1980]
Prionocera serricornis (Zetterstedt, 1838) [Hackman 1980]
Prionocera subserricornis (Zetterstedt, 1851) [Hackman 1980]
= *P. proxima* Lackschewitz, 1933 [Hackman 1980, Brodo 1987]
Prionocera turcica (Fabricius, 1787) [Hackman 1980]
Prionocera woodoorum Brodo, 1987 [Salmela 2008]
tribe Tipulini Latreille, 1802
ANGAROTIPULA Savchenko, 1961
Angarotipula tumidicornis (Lundström, 1907) [Hackman 1980]

NEPHROTOMA Meigen, 1803
Nephrotoma aculeata (Loew, 1871) [Hackman 1980]
Nephrotoma analis (Schummel, 1833) [Hackman 1980]
Nephrotoma appendiculata (Pierre, 1919) [Hackman 1980]
Nephrotoma cornicina (Linnaeus, 1758) [Hackman 1980]
Nephrotoma crocata (Linnaeus, 1758) [Hackman 1980]
Nephrotoma dorsalis (Fabricius, 1781) [Hackman 1980]
Nephrotoma flavescens (Linnaeus, 1758) [Hackman 1980]
Nephrotoma lundbecki (Nielsen, 1907) [Salmela 2011]
Nephrotoma lunulicornis (Schummel, 1833) [Hackman 1980]
Nephrotoma pratensis (Linnaeus, 1758) [Hackman 1980]
Nephrotoma quadristriata (Schummel, 1833) [Hackman 1980]
Nephrotoma relicta (Savchenko, 1973) [Rautio 1986, Salmela 2011]
Nephrotoma scurra (Meigen, 1818) [Hackman 1980]
Nephrotoma submaculosa Edwards, 1928 [Rautio 1987, Salmela 2011]
Nephrotoma tenuipes (Riedel, 1910) [Hackman 1980]
NIGROTIPULA Hudson & Vane-Wright, 1969
Nigrotipula nigra (Linnaeus, 1758) [Hackman 1980]
TIPULA Linnaeus, 1758
sg. ***Acutipula*** Alexander, 1924
Tipula fulvipennis De Geer, 1776 [Hackman 1980]
Tipula maxima Poda, 1761 [Hackman 1980]
sg. ***Arctotipula*** Alexander, 1934
Tipula salicetorum Siebke, 1870 [Hackman 1980]
= *T. nigricornis* Zetterstedt, 1851
sg. ***Beringotipula*** Savchenko, 1961
Tipula unca Wiedemann, 1817 [Hackman 1980]
= *T. hortensis* Meigen, 1818
sg. ***Dendrotipula*** Savchenko, 1964
Tipula flavolineata Meigen, 1804 [Hackman 1980]
sg. ***Emodotipula*** Alexander, 1966
Tipula obscuriventris Strobl, 1900 [Salmela 2011]
sg. ***Lindnerina*** Mannheims, 1965
Tipula bistilata Lundström, 1907 [Hackman 1980]
Tipula subexcisa Lundström, 1907 [Hackman 1980]
sg. ***Lunatipula*** Edwards, 1931
Tipula affinis Schummel, 1833 [Hackman 1980]
Tipula circumdata Siebke, 1863 [Hackman 1980]
= *T. livida* misid. [Hackman 1980]
Tipula fascipennis Meigen, 1818 [Hackman 1980]
Tipula humilis Staeger, 1840 [Hackman 1980]
Tipula laetabilis Zetterstedt, 1838 [Salmela 2011]
= *T. dilatata* Schummel, 1833 [Hackman 1980]
Tipula limitata Schummel, 1833 [Hackman 1980]
Tipula lunata Linnaeus, 1758 [Hackman 1980]
= *T. luna* mistake[Autio and Salmela 2010]
? *Tipula peliostigma* Schummel, 1833 [Salmela 2011]
Tipula recticornis Schummel, 1833 [Hackman 1980]
Tipula selene Meigen, 1830 [Hackman 1980]
Tipula trispinosa Lundström, 1907 [Hackman 1980]

Tipula vernalis Meigen, 1804 [Hackman 1980]
sg. ***Odonatisca*** Savchenko, 1956
Tipula nodicornis Meigen, 1818
= *T. juncea* Meigen, 1818 [Hackman 1980]
sg. ***Platytipula*** Matsumura, 1916
Tipula luteipennis Meigen, 1830 [Hackman 1980]
Tipula melanoceros Schummel, 1833 [Hackman 1980]
sg. ***Pterelachisus*** Rondani, 1842
Tipula cinereocincta Lundström, 1907 [Hackman 1980]
Tipula crassicornis Zetterstedt, 1838 [Hackman 1980]
Tipula irrorata Macquart, 1826 [Hackman 1980]
Tipula jutlandica Nielsen, 1947 [Salmela 2009]
Tipula kaisilai Mannheims, 1954 [Kangas 1952, Hackman 1980]
Tipula laetibasis Alexander, 1934 [Salmela et al. 2007b]
Tipula luridorostris Schummel, 1833 [Hackman 1980]
Tipula matsumuriana Alexander, 1924 [Salmela 2011]
= *T. pseudohortensis* Lackschewitz, 1932 [Hackman 1980]
Tipula mutila Wahlgren, 1905 [Hackman 1980]
Tipula octomaculata Savchenko, 1964 [Salmela and Autio 2007a]
Tipula pabulina Meigen, 1818 [Salmela 2009]
Tipula pauli Mannheims, 1964
Tipula pseudoirrorata Goetghebuer, 1921 [Siitonen 1983b, Salmela 2011]
Tipula recondita Pilipenko & Salmela, 2012 [Pilipenko et al. 2012]
Tipula stenostyla Savchenko, 1964 [Salmela 2009]
Tipula submarmorata Schummel, 1833
= *T. meigeni* Mannheims, 1966 [Hackman 1980]
Tipula truncorum Meigen, 1830 [Hackman 1980]
Tipula varipennis Meigen, 1818 [Hackman 1980]
= *T. pseudovariipennis* misid. [Mannheims 1963b, Hackman 1980]
Tipula wahlgreni Lackschewitz, 1925 [Starý and Salmela 2004, Salmela 2011]
Tipula winthemi Lackschewitz, 1932 [Hackman 1980]
sg. ***Savtshenkia*** Alexander, 1965
Tipula alpium Bergroth, 1888 [Rautio 1986, Salmela 2011]
Tipula benesignata Mannheims, 1954 [Siitonen 1982, Salmela 2011]
Tipula confusa van der Wulp, 1883 [Salmela 2011]
= *T. marmorata* Meigen, 1818 [Hackman 1980]
Tipula gimmerthali Lackschewitz, 1925 [Hac 1964, Karppinen 1964a, Hackman 1980, Salmela 2011]
Tipula grisescens Zetterstedt, 1851 [Hackman 1980]
Tipula interserta Riedel, 1913 [Hackman 1980]
Tipula invenusta Riedel, 1919 [Salmela 2008]
Tipula limbata Zetterstedt, 1838 [Hackman 1980]
Tipula obsoleta Meigen, 1818 [Hackman 1980]
Tipula pagana Meigen, 1818 [Hackman 1980]
Tipula signata Staeger, 1840 [Hackman 1980]
Tipula subnodicornis Zetterstedt, 1838 [Hackman 1980]
sg. ***Schummelia*** Edwards, 1931
Tipula variicornis Schummel, 1833 [Hackman 1980]
sg. ***Tipula*** Linnaeus, 1758
Tipula paludosa Meigen, 1830 [Hackman 1980]

= *T. oleracea* misid. [Hackman 1980, Brodo 1994]
Tipula subcunctans Alexander, 1921 [Salmela 2011]
= *T. czizeki* de Jong, 1925 [Hackman 1980]
sg. ***Vestiplex*** Bezzi, 1924
Tipula excisa Schummel, 1833 [Hackman 1980]
Tipula hortorum Linnaeus, 1758 [Hackman 1980]
Tipula laccata Lundström & Frey, 1916 [Viramo 1992]
Tipula montana Curtis, 1834 [Mannheims 1963b, Hackman 1980]
Tipula nubeculosa Meigen, 1804 [Hackman 1980]
Tipula pallidicosta Pierre, 1924 [Hackman 1980]
Tipula scripta Meigen, 1830 [Hackman 1980]
Tipula sintenisi Lackschewitz, 1933 [Hackman 1980]
Tipula tchukchi Alexander, 1934 [Hackman 1980]
= *T. bo* Mannheims, 1967 [Mannheims 1967]
sg. ***Yamatotipula*** Matsumura, 1916
Tipula chonsaniana Alexander, 1945 [Salmela and Autio 2009]
Tipula coerulescens Lackschewitz, 1923 [Hackman 1980]
Tipula couckei Tonnoir, 1921 [Hackman 1980]
Tipula fendleri Mannheims, 1963 [Mannheims 1963a, Hackman 1980]
Tipula freyana Lackschewitz, 1936 [Hackman 1980]
Tipula lateralis Meigen, 1804 [Hackman 1980]
Tipula marginella Theowald, 1980
= *T. marginata* Meigen, 1818 [Hackman 1980]
Tipula moesta Riedel, 1919 [Hackman 1980]
Tipula montium Egger, 1863 [Hackman 1980]
Tipula pierrei Tonnoir, 1921 [Salmela 2011]
= *T. solstitialis* Westhoff, 1879 [Hackman 1980]
Tipula pruinosa Wiedemann, 1817 [Hackman 1980]
Tipula quadrivittata Staeger, 1840 [Hackman 1980]

Excluded species

Tipula fenestrella Theowald, 1980 [Oosterbroek and Theowald 1992, Salmela 2011]
Tipula middendorffi Lackschewitz, 1936 [Oosterbroek and Theowald 1992, Salmela 2011]
Tipula postposita Riedel, 1919 [Oosterbroek and Theowald 1992, Salmela 2011]

Tipula pseudovariipennis Czizek, 1912 [Mannheims 1963b, Hackman 1980, Salmela 2011]
Tipula vittata Meigen, 1804 [Mannheims 1963a, Salmela 2011]

Nematocera Dumeril, 1805
infraorder Psychodomorpha Hennig, 1968
PSYCHODIDAE Newman, 1834

Supporting references for *Salmela J, Paasivirta L, Kvifte GM (2014) Checklist of the familes Chaoboridae, Dixidae, Thaumaleidae Psychodidae and Ptychopteridae (Diptera) of Finland. In: Kahanpää J, Salmela J (Eds) Checklist of the Diptera of Finland. ZooKeys @@: @-@. doi: 10.3897/zookeys.??.7532*

PSYCHODINAE Newman, 1834
tribe Pericomaini Enderlein, 1935 preocc.
BERDENIELLA Vaillant, 1976
Berdeniella freyi (Berdén, 1954) [Hackman 1980]
CLYTOCERUS Eaton, 1904
sg. ***Boreoclytocerus*** Duckhouse, 1978
Clytocerus ocellaris (Meigen, 1818) [Hackman 1980]
Clytocerus rivosus (Tonnoir, 1919) [Salmela 2002]
Clytocerus tetracorniculatus Wagner, 1977 [Salmela 2003a]
PARABAZARELLA Vaillant, 1983
Parabazarella subneglecta (Tonnoir, 1922) [Salmela 2003a]
PERICOMA Walker, 1856
sg. ***Pachypericoma*** Vaillant, 1978
Pericoma blandula Eaton, 1893 [Salmela 2003a]
Pericoma nielseni Kvifte, 2010
= *Pericoma formosa* Nielsen, 1964 preocc.[Salmela 2003a]
sg. ***Pericoma*** Walker, 1856
Pericoma rivularis Berdén, 1954 [Hackman 1980]
PNEUMIA Enderlein, 1935
= ***Satchelliella*** Vaillant, 1979
Pneumia borealis (Berdén, 1954) [Hackman 1980]
Pneumia bucegica Vaillant, 1981 [Salmela 2002]
Pneumia mutua (Eaton, 1893) [Hackman 1980]
? *Pneumia nubila* (Meigen, 1818) [Hackman 1980]
? *Pneumia palustris* (Meigen, 1818) [Hackman 1980]
Pneumia pilularia Tonnoir, 1940 [Salmela 2008]
Pneumia stammeri (Jung, 1954) [Salmela 2003a]
Pneumia trivialis (Eaton, 1893) [Salmela 2003a]
Pneumia ussurica (Wagner, 1994) [Salmela 2004]
ULOMYIA Walker, 1856
Ulomyia cognata (Eaton, 1893) [Salmela 2003a]
Ulomyia fuliginosa (Meigen, 1804) [Hackman 1980, Salmela 2006]
tribe Psychodini Newman, 1834
PHILOSEPEDON Eaton, 1904
Philosepedon balkanicus Krek, 1970 [Salmela 2003a]
Philosepedon humeralis (Meigen, 1818) [Salmela 2006]
Philosepedon soljani Krek, 1971
PSYCHODA Latreille, 1796
Psychoda albipennis Zetterstedt, 1850 [Salmela 2002]
= *P. parthenogenetica* Tonnoir, 1940
Psychoda alternata Say, 1824 [Hackman 1980]

Psychoda brevicornis Tonnoir, 1940
Psychoda buxtoni Withers, 1988 [Salmela 2006]
Psychoda cinerea Banks, 1894 [Salmela 2003a]
Psychoda crassipenis Tonnoir, 1940
Psychoda cultella Salmela, Kvifte & More, 2012
Psychoda erminea Eaton, 1898 [Salmela 2005]
Psychoda gemina (Eaton, 1904) [Salmela 2003a]
Psychoda grisescens Tonnoir, 1922 [Salmela 2008]
Psychoda itoco Tokunaga & Komyo, 1955 [Salmela et al. 2007a]
Psychoda lativentris Berdén, 1952 [Salmela et al. 2007a]
Psychoda lobata Tonnoir, 1940 [Hackman 1980, Salmela 2006]
Psychoda minuta Banks, 1894 [Salmela 2003a]
Psychoda phalaenoides Linnaeus, 1758 [Hackman 1980]
Psychoda satchelli Quate, 1955 [Salmela 2004, 2005]
Psychoda setigera Tonnoir, 1922
Psychoda trinodulosa Tonnoir, 1922 [Salmela 2006]
Psychoda uniformata Haseman, 1907
THRETICUS Eaton, 1904
Threticus tridactilis (Kincaid, 1899)
TRICHOPSYCHODA Tonnoir, 1922
Trichopsychoda hirtella (Tonnoir, 1919) [Salmela and Halme 2007]
tribe Maruinini Enderlein, 1937
LOBULOSA Szabo, 1960
Lobulosa pollex (Berdén, 1954)
TONNOIRIELLA Vaillant, 1971
Tonnoiriella nigricauda (Tonnoir, 1919) [Salmela 2002]
tribe Mormiini Enderlein, 1937
MORMIA Enderlein, 1935
Mormia niesiolowskii Wagner, 1985 [Salmela 2003a]
Mormia strobli (Ježek, 1986) [Salmela and Halme 2007]
tribe Paramormiini Enderlein, 1937
PARAJUNGIELLA Vaillant, 1972
Parajungiella consors (Eaton, 1893) [Salmela 2002]
Parajungiella ellisi (Withers, 1987) [Salmela 2002]
Parajungiella pseudolongicornis Wagner, 1975 [Salmela 2002]
LEPISEODINA Enderlein, 1937
Lepiseodina rothschildi (Eaton, 1912) [Salmela 2010]
PANIMERUS Eaton, 1913
sg. ***Panimerus*** Eaton, 1913
Panimerus albomaculatus (Wahlgren, 1904)
Panimerus notabilis (Eaton, 1893) [Hackman 1980]
sg. ***Psycmera*** Ježek, 1984
Panimerus albifacies (Tonnoir, 1919)
Panimerus intellegus (Jung, 1956) [Salmela 2005]
Panimerus przhiboroi Wagner, 2005
PARAMORMIA Enderlein, 1935
Paramormia polyscoidea (Krek, 1970) [Salmela 2003a]
Paramormia ustulata (Walker, 1856)
PERIPSYCHODA Enderlein, 1935
Peripsychoda auriculata (Curtis, 1839) [Salmela et al. 2007a]

Peripsychoda fusca (Macquart, 1826) [Hackman 1980]
TELMATOSCOPUS Eaton, 1904
Telmatoscopus advena (Eaton, 1893) [Salmela 2005]
Telmatoscopus similis Tonnoir, 1922 [Hackman 1980]
SYCORACINAE Rondani, 1856
SYCORAX Haliday *in* Curtis, 1839
Sycorax silacea Haliday *in* Curtis, 1839 [Salmela 2003a]

CERATOPOGONIDAE Newman, 1834

Supporting references for *Huldén L, Huldén L (2014) Checklist of the family Ceratopogonidae (Diptera) of Finland. In: Kahanpää J, Salmela J (Eds) Checklist of the Diptera of Finland. ZooKeys @@: @-@. doi:*

FORCIPOMYIINAE Lenz, 1934
ATRICHOPOGON Kieffer, 1906
sg. ***Atrichopogon*** Kieffer, 1906
Atrichopogon brunnipes (Meigen, 1804) [Hackman 1980]
Atrichopogon fuscus (Meigen, 1804) [Hackman 1980]
= *A. fossicola* (Kieffer, 1922)
Atrichopogon longicalcar Remm, 1961
Atrichopogon minutus (Meigen, 1830) [Hackman 1980]
Atrichopogon pavidus (Winnertz, 1852) [Hackman 1980]
sg. ***Meloehelea*** Wirth, 1956
Atrichopogon lucorum (Meigen, 1818) [Hackman 1980]
Atrichopogon meloesugans Kieffer, 1922 [Hackman 1980]
= *A. winnertzi* auct. nec Goetghebuer, 1922
Atrichopogon oedemerarum Storå, 1939 [Storå 1939, Hackman 1980]
sg. ***Psilokempia*** Enderlein, 1936
Atrichopogon appendiculatus (Goetghebuer, 1920)
Atrichopogon forcipatus (Winnertz, 1852) [Hackman 1980]
Atrichopogon maculatus (Lundström, 1910) [Lundström 1910]
Atrichopogon rostratus (Winnertz, 1852)
FORCIPOMYIA Meigen, 1818
sg. ***Caloforcipomyia*** Saunders, 1957
Forcipomyia glauca Macfie, 1934 [Clastrier 1991]
sg. ***Forcipomyia*** Meigen, 1818
Forcipomyia bipunctata (Linnaeus, 1767) [Hackman 1980]
Forcipomyia ciliata (Winnertz, 1852) [Hackman 1980]
Forcipomyia costata (Zetterstedt, 1838)
= *F. picea* (Winnertz, 1852) [Hackman 1980]
Forcipomyia kaltenbachi (Winnertz, 1852) [Hackman 1980]
Forcipomyia myrmecophila (Egger, 1863) [Hackman 1980]
Forcipomyia nigra (Winnertz, 1852) [Hackman 1980]
sg. ***Microhelea*** Kieffer, 1917
Forcipomyia fuliginosa (Meigen, 1818) [Hackman 1980]
= *F. brevimana* Lundström, 1910
sg. ***Thyridomyia*** Saunders, 1925
Forcipomyia monilicornis (Coquillett, 1905)
= *F. hirta* (Lundström, 1910) [Hackman 1980]
sg. ***Trichohelea*** Goetghebuer, 1920
Forcipomyia eques (Johanssen, 1908) [Hackman 1980]
DASYHELEINAE Lenz, 1934
DASYHELEA Kieffer, 1911
sg. ***Dasyhelea*** Kieffer, 1911
Dasyhelea bensoni Edwards, 1933
= *D. vernalis* Remm, 1979
Dasyhelea bilineata Goetghebuer, 1920

= *D. saxicola* (Edwards, 1929)
= *D. obscura* auct. nec (Winnertz, 1852)
= *D. versicolor* auct. nec (Winnertz, 1852)
Dasyhelea pallidiventris (Goetghebuer, 1931)
sg. ***Dicryptoscena*** Enderlein, 1936
Dasyhelea modesta (Winnertz, 1852) [Remm 1988]
= *D. aestiva* (Winnertz, 1852) [Hackman 1980, Remm 1988]
= *D. longipalpis* Kieffer, 1913 [Hackman 1980, Remm 1988]
Dasyhelea notata Goetghebuer, 1920
sg. ***Pseudoculicoides*** Malloch, 1915
Dasyhelea corinneae Gosseries, 1991
= *D. scutellata* (Meigen, 1804) preocc.
Dasyhelea flavoscutellata (Zetterstedt, 1850)
= *D. egens* (Winnertz, 1852)
sg. ***Sebessia*** Remm, 1979
Dasyhelea holosericea (Meigen, 1804) [Storå 1939, Hackman 1980]
CERATOPOGONINAE Newman, 1834
tribe Culicoidini Kieffer, 1911
CULICOIDES Latreille, 1809
sg. ***Avaritia*** Fox, 1955
sg. ***Beltranmyia*** Vargas, 1953
sg. ***Culicoides*** Latreille, 1809
sg. ***Monoculicoides*** Khalaf, 1954
sg. ***Oecacta*** Poey, 1853
sg. ***Silvaticulicoides*** Glukhova, 1977
sg. ***Wirthomyia*** Vargas, 1973
Culicoides achrayi Kettle & Lawson, 1955
Culicoides albicans (Winnertz, 1852)
= *C. vexans* auct. nec (Staeger, 1839) [Hackman 1980, Delécolle et al. 1983]
Culicoides chiopterus (Meigen, 1830)
Culicoides circumscriptus Kieffer, 1918
Culicoides dewulfi Goetghebuer, 1936 [Hildén et al. 2010]
Culicoides fascipennis (Staeger, 1839)
Culicoides festivipennis Kieffer, 1914
= *C. odiblis* Austen, 1921
Culicoides grisescens Edwards, 1939
Culicoides impunctatus Goetghebuer, 1920
Culicoides kibunensis Tokunaga, 1937
= *C. cubitalis* Edwards in Edwards et al., 1939
Culicoides nubeculosus (Meigen, 1830) [Hackman 1980]
Culicoides obsoletus (Meigen, 1818)
= *C. varius* (Winnertz, 1852)
Culicoides pallidicornis (Kieffer, 1919)
Culicoides pictipennis (Staeger, 1839) [Hackman 1980]
Culicoides pseudoheliophilus Callot & Kremer, 1961 [Huldén et al. 2008]
Culicoides pulicaris (Linnaeus, 1758) [Hackman 1980]
Culicoides punctatus (Meigen, 1804) [Delécolle et al. 1983]
Culicoides riethi Kieffer, 1914
Culicoides riouxi Callot & Kremer, 1961
Culicoides salinarius Kieffer, 1914

Culicoides scoticus Downes & Kettle, 1952
Culicoides segnis Campbell & Pelham-Clinton, 1960
Culicoides sphagnumensis Williams, 1955 [Huldén et al. 2008]
 = *C. carjalaensis* Glukhova, 1957
Culicoides stigma (Meigen, 1818) [Hackman 1980]
Culicoides subfasciipennis Kieffer, 1919 [Huldén et al. 2008]
 tribe Ceratopogonini Newman, 1834
ALLUAUDOMYIA Kieffer, 1913
Alluaudomyia needhami Thomsen, 1935
 = *A. pentaspila* Remm & Glukhova, 1971
Alluaudomyia quadripunctata (Goetghebuer, 1934) [Hackman 1980]
BRACHYPOGON Kieffer, 1899
 sg. ***Isohelea*** Kieffer, 1917
Brachypogon aquilonalis (Clastrier, 1961) [Hackman 1980]
Brachypogon hyperboreus (Clastrier, 1961) [Hackman 1980]
Brachypogon incompletus (Kieffer, 1925)
 = *B. lapiae* Clastrier, 1961 [Hackman 1980]
Brachypogon nitidulus (Edwards, 1921)
 = *B. finniae* (Clastrier, 1961) [Hackman 1980]
Brachypogon sociabilis (Goetghebuer, 1920)
CERATOPOGON Meigen, 1803
Ceratopogon lacteipennis Zetterstedt, 1838
Ceratopogon niveipennis Meigen, 1818
 = *C. candidatus* Winnertz, 1852
SCHIZOHELEA Kieffer, 1917
Schizohelea leucopeza (Meigen, 1804) [Hackman 1980]
 = *S. copiosa* Winnertz, 1852
 = *S. xanthopeza* (Clastrier, 1963)
SERROMYIA Meigen, 1818
Serromyia atra (Meigen, 1818)
Serromyia femorata (Meigen, 1804) [Hackman 1980]
Serromyia subinermis Kieffer, 1919 [Borkent and Bissett 1990]
 tribe Heteromyiini Wirth, 1962
CLINOHELEA Kieffer, 1917
Clinohelea unimaculata (Macquart, 1826) [Hackman 1980]
 = *C. variegata* (Winnertz, 1852)
 tribe Sphaeromiini Newman, 1834
MALLOCHOHELEA Wirth, 1962
Mallochohelea inermis (Kieffer, 1909)
Mallochohelea munda (Loew, 1864)
 = *M. dentata* (Kieffer, 1909)
Mallochohelea nitida (Macquart, 1826) [Hackman 1980]
Mallochohelea setigera (Loew, 1864)
Mallochohelea vernalis Remm, 1965
PROBEZZIA Kieffer, 1906
 = ***Dicrobezzia*** Kieffer, 1919
Probezzia concinna (Meigen, 1818)
Probezzia seminigra (Panzer, 1798) [Hackman 1980]
 = *P. venusta* (Meigen, 1818)
 = *P. borealis* (Clastrier, 1962) [Hackman 1980, Remm 1988]

SPHAEROMIAS Curtis, 1829
Sphaeromias fasciatus (Meigen, 1804)
Sphaeromias pictus (Meigen, 1818) [Hackman 1980]
= *S. candidatus* (Loew, 1856) [Storå 1939]
tribe Palpomyiini Enderlein, 1936
BEZZIA Kieffer, 1899
Bezzia annulipes (Meigen, 1830) [Storå 1939, Hackman 1980]
Bezzia bicolor (Meigen, 1804) [Hackman 1980]
Bezzia circumdata (Staeger, 1839)
= *B. solstitialis* (Winnertz, 1852) [Hackman 1980]
Bezzia coracina (Zetterstedt, 1850) [Hackman 1980]
= *B. albipes* (Winnertz, 1852) [Hackman 1980]
Bezzia fascispinosa Clastrier, 1962
Bezzia flavicornis (Staeger, 1839)
= *B. flavipalpis* (Winnertz, 1852)
Bezzia fuscifemoris Remm, 1971
Bezzia leucogaster (Zetterstedt, 1850) [Hackman 1980]
= *B. xanthocephala* Goetghebuer, 1911 [Storå 1939, Hackman 1980]
Bezzia nigritula (Zetterstedt, 1838) [Storå 1939, Hackman 1980]
Bezzia nobilis (Winnertz, 1852)
= *B. punctata* Lundström, 1910
Bezzia winnertziana Kieffer, 1919
= *B. gracilis* (Winnertz, 1852) preocc.[Hackman 1980]
PALPOMYIA Meigen, 1818
Palpomyia armipes (Meigen, 1838) [Hackman 1980]
= *P. rufipecta* (Winnertz, 1852)
Palpomyia distincta (Haliday, 1833) [Storå 1939, Hackman 1980]
Palpomyia flavipes (Meigen, 1804) [Hackman 1980]
= *P. hortulana* Meigen, 1818
Palpomyia flavitarsis (Meigen, 1838)
Palpomyia lineata (Meigen, 1804) [Hackman 1980]
= *P. stagnalis* Clastrier, 1962
Palpomyia lundstroemi Remm, 1981
= *P. bispinosa* Lundström, 1916 preocc.[Hackman 1980]
Palpomyia serripes (Meigen, 1818) [Hackman 1980]
= *P. transfuga* (Staeger, 1839)
Palpomyia tibialis (Meigen, 1818) [Hackman 1980]

Excluded species

Culicoides neglectus (Winnertz, 1852) [Hackman 1980, Remm 1988]
Palpomyia aterrima Goetghebuer, 1921 [Storå 1939, Hackman 1980]
Palpomyia spinipes (Meigen in Panzer, 1806) [Hackman 1980]

CHIRONOMIDAE Newman, 1834

Supporting references for *Paasivirta L (2014) Checklist of the family Chironomidae (Diptera) of Finland. In: Kahanpää J, Salmela J (Eds) Checklist of the Diptera of Finland. ZooKeys @@: @-@. doi:*

PODONOMINAE Thienemann & Edwards, 1937
tribe Boreochlini Brundin, 1966
BOREOCHLUS Edwards, 1938
Boreochlus thienemanni Edwards, 1938 [Tuiskunen and Lindeberg 1986]
LASIODIAMESA Kieffer, 1924
Lasiodiamesa armata Brundin, 1966 [Hackman 1980]
Lasiodiamesa gracilis (Kieffer, 1924) [Hackman 1980]
Lasiodiamesa sphagnicola (Kieffer, 1925) [Hackman 1980]
tribe Podonomini Thienemann & Edwards, 1937
PAROCHLUS Enderlein, 1912
Parochlus kiefferi (Garrett, 1925) [Hackman 1980]
TRICHOTANYPUS Kieffer, 1906
Trichotanypus mariae Wirth & Sublette, 1970 [Paasivirta 2009]
Trichotanypus posticalis (Lundbeck, 1898) [Hackman 1980]
TANYPODINAE Skuse, 1889
tribe Anatopyniini Fittkau, 1962
ANATOPYNIA Johannsen, 1905
Anatopynia plumipes (Fries, 1823) [Paasivirta 2012]
tribe Coelotanypodini Coffman, 1978
CLINOTANYPUS Kieffer, 1913
sg. ***Clinotanypus*** Kieffer, 1913
Clinotanypus nervosus (Meigen, 1818) [Hackman 1980] [Hackman 1980]
tribe Macropelopiini Zavřel, 1929
APSECTROTANYPUS Fittkau, 1962
Apsectrotanypus trifascipennis [Hackman 1980] (Zetterstedt, 1838)
MACROPELOPIA Thienemann, 1916
Macropelopia adaucta Kieffer, 1916
= *M. goetghebueri* (Kieffer, 1918) [Hackman 1980]
Macropelopia nebulosa (Meigen, 1804) [Hackman 1980]
Macropelopia notata (Meigen, 1818) [Bergman and Jansson 1983]
PSECTROTANYPUS Kieffer, 1909
Psectrotanypus varius (Fabricius, 1787) [Hackman 1980]
tribe Natarsiini Roback & Moss, 1978
NATARSIA Fittkau, 1962
Natarsia nugax (Walker, 1856) [Paasivirta 2009]
Natarsia punctata (Fabricius, 1805) [Hackman 1980]
tribe Pentaneurini Hennig, 1950
ABLABESMYIA Johannsen, 1905
Ablabesmyia longistyla Fittkau, 1962 [Hackman 1980]
Ablabesmyia monilis (Linnaeus, 1758) [Hackman 1980]
Ablabesmyia phatta (Egger, 1864) [Hackman 1980]
ARCTOPELOPIA Fittkau, 1962
Arctopelopia barbitarsis (Zetterstedt, 1850) [Hackman 1980]
Arctopelopia griseipennis (van der Wulp, 1859) [Hackman 1980]

Arctopelopia melanosoma (Goetghebuer, 1933) [Tuiskunen and Lindeberg 1986]
CONCHAPELOPIA Fittkau, 1957
Conchapelopia aagaardi Murray, 1987 [Paasivirta 2009]
Conchapelopia hittmairorum Michiels & Spies, 2002 [Paasivirta 2009]
Conchapelopia intermedia Fittkau, 1962 [Tuiskunen and Lindeberg 1986]
Conchapelopia melanops (Meigen, 1818) [Hackman 1980]
Conchapelopia pallidula (Meigen, 1818) [Hackman 1980]
GUTTIPELOPIA Fittkau, 1962
Guttipelopia guttipennis (van der Wulp, 1861) [Hackman 1980]
HAYESOMYIA Murray & Fittkau, 1986
Hayesomyia tripunctata (Goetghebuer, 1922) [Paasivirta 2009]
KRENOPELOPIA Fittkau, 1962
Krenopelopia binotata (Wiedemann, 1817) [Krogerus 1960]
Krenopelopia nigropunctata (Staeger, 1839) [Paasivirta 2009]
LABRUNDINIA Fittkau, 1962
Labrundinia longipalpis (Goetghebuer, 1921) [Hackman 1980]
LARSIA Fittkau, 1962
Larsia atrocincta (Goetghebuer, 1942) [Paasivirta 2009]
MONOPELOPIA Fittkau, 1962
Monopelopia tenuicalcar (Kieffer, 1918) [Hackman 1980]
NILOTANYPUS Kieffer, 1923
Nilotanypus dubius (Meigen, 1804) [Hackman 1980]
PARAMERINA Fittkau, 1962
Paramerina cingulata (Walker, 1856) [Hackman 1980]
Paramerina divisa (Walker, 1856) [Bergman and Jansson 1983]
PENTANEURELLA Fittkau & Murray, 1983
Pentaneurella katterjokki Fittkau & Murray, 1983 [Paasivirta 2009]
RHEOPELOPIA Fittkau, 1962
Rheopelopia maculipennis (Zetterstedt, 1838) [Paasivirta 2009]
Rheopelopia ornata (Meigen, 1838) [Hackman 1980]
TELMATOPELOPIA Fittkau, 1962
Telmatopelopia nemorum (Goetghebuer, 1921) [Hackman 1980]
TELOPELOPIA Roback, 1971
Telopelopia fascigera (Verneaux, 1970) [Paasivirta 2009]
THIENEMANNIMYIA Fittkau, 1957
Thienemannimyia carnea (Fabricius, 1805) [Hackman 1980]
Thienemannimyia fusciceps (Edwards, 1929) [Hackman 1980]
Thienemannimyia laeta (Meigen, 1818) [Paasivirta 2009]
Thienemannimyia lentiginosa (Fries, 1823) [Hackman 1980]
Thienemannimyia pseudocarnea Murray, 1976 [Murray 1976, Hackman 1980]
Thienemannimyia vitellina (Kieffer, 1916) [Paasivirta 2009]
TRISSOPELOPIA Kieffer, 1923
Trissopelopia longimana (Staeger, 1839) [Bergman and Jansson 1983]
XENOPELOPIA Fittkau, 1962
Xenopelopia falcigera (Kieffer, 1911) [Paasivirta 2009]
Xenopelopia nigricans (Goetghebuer, 1927) [Hackman 1980]
ZAVRELIMYIA Fittkau, 1962
Zavrelimyia barbatipes (Kieffer, 1911) [Bergman and Jansson 1983]
Zavrelimyia hirtimana (Kieffer, 1918) [Hirvenoja and Michailova 1991]
Zavrelimyia melanura (Meigen, 1804) [Hackman 1980]

tribe Procladiini Roback, 1971
PROCLADIUS Skuse, 1889
sg. ***Holotanypus*** Roback, 1982
Procladius appropinquatus (Lundström, 1917) [Paasivirta 2012]
Procladius choreus (Meigen, 1804) [Hackman 1980]
Procladius culiciformis (Linnaeus, 1767) [Hackman 1980]
= *P. crassinervis* (Zetterstedt, 1838) [Paasivirta 2009]
Procladius fimbriatus Wülker, 1959 [Wülker 1959, Hackman 1980]
Procladius fuscus Brundin, 1949 [Paasivirta 2009]
? *Procladius islandicus* (Goetghebuer, 1931) [Paasivirta 2012]
Procladius nudipennis Brundin, 1947 [Hackman 1980]
Procladius pectinatus (Kieffer, 1909) [Hackman 1980]
Procladius signatus (Zetterstedt, 1850) [Hackman 1980]
Procladius simplicistylus Freeman, 1948 [Hackman 1980]
Procladius suecicus Brundin, 1949 [Paasivirta 2009]
Procladius cf.*vesus* Roback, 1971 [Paasivirta 2012]
Procladius sp. 1 "Valassaaret" [Paasivirta 2012]
Procladius sp. 2 "Palsa" [Paasivirta 2012]
Procladius sp. 3 "Kaldoaivi" [Paasivirta 2012]
sg. ***Psilotanypus*** Kieffer, 1906
Procladius flavifrons Edwards, 1929 [Hackman 1980]
Procladius imicola Kieffer, 1922 [Paasivirta 2012]
Procladius lugens Kieffer, 1915 [Hackman 1980]
Procladius rufovittatus (van der Wulp, 1874) [Paasivirta 2009]
tribe Tanypodini Skuse, 1889
TANYPUS Meigen, 1803
sg. ***Tanypus*** Meigen, 1803
Tanypus kraatzi (Kieffer, 1912) [Hackman 1980]
Tanypus punctipennis Meigen, 1818 [Hackman 1980]
Tanypus vilipennis (Kieffer, 1918) [Paasivirta 1979, Hackman 1980]
DIAMESINAE Kieffer, 1922
tribe Diamesini Kieffer, 1922
DIAMESA Meigen, 1835
sg. ***Diamesa*** Meigen, 1835
Diamesa aberrata Lundbeck, 1898 [Hackman 1980]
Diamesa arctica (Boheman, 1865) [Paasivirta 2012]
Diamesa bertrami Edwards, 1935 [Tuiskunen and Lindeberg 1986]
Diamesa bohemani Goetghebuer, 1932 [Hackman 1980]
Diamesa hyperborea Holmgren, 1869 [Paasivirta 2009]
Diamesa incallida (Walker, 1856) [Tuiskunen and Lindeberg 1986]
Diamesa insignipes Kieffer, 1908 [Paasivirta 2009]
Diamesa latitarsis (Goetghebuer, 1921) [Tuiskunen and Lindeberg 1986]
Diamesa permacra (Walker, 1856) [Hackman 1980]
Diamesa serratosioi Willassen, 1986 [Tuiskunen and Lindeberg 1986]
Diamesa tonsa (Haliday, 1856)
= *D. thienemanni* Kieffer, 1909 [Tuiskunen and Lindeberg 1986]
POTTHASTIA Kieffer, 1922
Potthastia gaedii (Meigen, 1838) [Hackman 1980]
Potthastia longimanus Kieffer, 1922 [Hackman 1980]
Potthastia pastoris (Edwards, 1933) [Tuiskunen and Lindeberg 1986]

PSEUDODIAMESA Goetghebuer, 1939
sg. ***Pseudodiamesa*** Goetghebuer, 1939
Pseudodiamesa branickii (Nowicki, 1873) [Hackman 1980]
Pseudodiamesa nivosa (Goetghebuer, 1928) [Paasivirta 2012]
PSEUDOKIEFFERIELLA Zavřel, 1941
= ***Diplomesa*** Zavřel, 1941
Pseudokiefferiella parva (Edwards, 1932) [Hackman 1980]
SYMPOTTHASTIA Pagast, 1947
Sympotthastia fulva (Johannsen, 1921) [Paasivirta 2009]
Sympotthastia huldeni Tuiskunen, 1986 [Tuiskunen 1986b]
SYNDIAMESA Kieffer, 1918
Syndiamesa sp. indet. larva
tribe Protanypini Brundin, 1956
PROTANYPUS Kieffer, 1906
Protanypus caudatus Edwards, 1924 [Hackman 1980]
Protanypus morio (Zetterstedt, 1838) [Hackman 1980]
TELMATOGETONINAE Wirth, 1949
TELMATOGETON Schiner, 1866
Telmatogeton japonicus Tokunaga, 1933 [Raunio et al. 2009]
PRODIAMESINAE Sæther, 1976
MONODIAMESA Kieffer, 1922
Monodiamesa bathyphila (Kieffer, 1918) [Hackman 1980]
Monodiamesa ekmani (Brundin, 1949) [Paasivirta 1983]
ODONTOMESA Pagast, 1947
Odontomesa fulva (Kieffer, 1919) [Paasivirta 2009]
PRODIAMESA Kieffer, 1906
Prodiamesa olivacea (Meigen, 1818) [Hackman 1980]
ORTHOCLADIINAE Kieffer, 1911
AAGAARDIA Sæther, 2000
Aagaardia protensa Sæther, 2000 [Paasivirta 2009]
Aagaardia sivertseni (Aagaard, 1979) [Tuiskunen and Lindeberg 1986]
Aagaardia sp. 1
ABISKOMYIA Edwards, 1937
Abiskomyia paravirgo Goetghebuer, 1940 [Paasivirta 2009]
Abiskomyia virgo Edwards, 1937 [Lindeberg 1974, Hackman 1980]
ACAMPTOCLADIUS Brundin, 1956
Acamptocladius reissi Cranston & Sæther, 1982 [Tuiskunen and Lindeberg 1986]
Acamptocladius submontanus (Edwards, 1932) [Tuiskunen and Lindeberg 1986]
ACRICOTOPUS Kieffer, 1921
= ***Lindebergia*** Tuiskunen, 1984
Acricotopus lucens (Zetterstedt, 1850) [Hackman 1980]
ALLOCLADIUS Kieffer, 1913
Allocladius bothnicus (Tuiskunen, 1984) [Tuiskunen 1984]
BOREOSMITTIA Tuiskunen, 1986
Boreosmittia inariensis Tuiskunen, 1986 [Tuiskunen and Lindeberg 1986]
Boreosmittia karelioborealis Tuiskunen, 1986 [Tuiskunen and Lindeberg 1986]
BRILLIA Kieffer, 1913
Brillia bifida (Kieffer, 1909)
= *B. modesta* (Meigen, 1830) preocc.[Hackman 1980]
Brillia longifurca Kieffer, 1921 [Hackman 1980]

BRYOPHAENOCLADIUS Thienemann, 1934
Bryophaenocladius aestivus (Brundin, 1947) [Hackman 1980]
Bryophaenocladius dentatus (Karl, 1937) [Tuiskunen and Lindeberg 1986]
Bryophaenocladius flexidens (Brundin, 1947) [Hackman 1980]
Bryophaenocladius ictericus (Meigen, 1830) [Hackman 1980]
Bryophaenocladius illimbatus (Edwards, 1929) [Paasivirta 2002]
Bryophaenocladius cf. *impectinus* Sæther, 1976 [Paasivirta 2012]
Bryophaenocladius inconstans (Brundin, 1947) [Paasivirta 2009]
Bryophaenocladius cf. *laticaudus* Sæther, 1973 [Paasivirta 2009]
Bryophaenocladius muscicola (Kieffer, 1906) [Paasivirta 2009]
Bryophaenocladius nidorum (Edwards, 1929) [Paasivirta and Koskenniemi 1984]
Bryophaenocladius nigrus Albu, 1974 [Paasivirta 2009]
Bryophaenocladius nitidicollis (Goetghebuer, 1913) [Paasivirta and Koskenniemi 1984]
Bryophaenocladius pectinatus Albu, 1974 [Paasivirta 2012]
Bryophaenocladius psilacrus Sæther, 1982 [Tuiskunen and Lindeberg 1986]
Bryophaenocladius saanae Tuiskunen, 1986 [Tuiskunen and Lindeberg 1986]
Bryophaenocladius scanicus (Brundin, 1947) [Hackman 1980]
Bryophaenocladius cf. *sclerus* Wang, Liu & Epler, 2004 [Paasivirta 2012]
Bryophaenocladius subparallelus (Malloch, 1915) [Paasivirta 2009]
Bryophaenocladius subvernalis (Edwards, 1929) [Paasivirta and Koskenniemi 1984]
Bryophaenocladius tuberculatus (Edwards, 1929) [Tuiskunen and Lindeberg 1986]
Bryophaenocladius vernalis (Goetghebuer, 1921) [Paasivirta 2009]
Bryophaenocladius xanthogyne (Edwards, 1929) [Hackman 1980]
Bryophaenocladius sp., pr. *xanthogyne*
Bryophaenocladius sp. 1 ”Syte”
Bryophaenocladius sp. 2 ”Syte”
Bryophaenocladius sp. 3 ”Malla”
Bryophaenocladius sp. 4 ”Tsarmi”
Bryophaenocladius sp. 5 ”Pallas”
Bryophaenocladius sp. 6 ”Rommas”
CAMPTOCLADIUS van der Wulp, 1874
Camptocladius stercorarius (De Geer, 1776) [Hackman 1980]
CARDIOCLADIUS Kieffer, 1912
Cardiocladius capucinus (Zetterstedt, 1850) [Hackman 1980]
Cardiocladius fuscus Kieffer, 1924 [Paasivirta 2009]
CHAETOCLADIUS Kieffer, 1911
sg. ***Chaetocladius*** Kieffer, 1911
Chaetocladius acuminatus Brundin, 1956 [Tuiskunen and Lindeberg 1986]
Chaetocladius binotatus (Lundström, 1915) [Paasivirta 2009]
Chaetocladius britae Säwedal, 1976 [Hackman 1980]
Chaetocladius crassisaetosus Tuiskunen, 1986 [Tuiskunen and Lindeberg 1986]
Chaetocladius dentiforceps (Edwards, 1929) [Paasivirta 1982]
Chaetocladius dissipatus (Edwards, 1929) [Paasivirta 2009]
Chaetocladius glacialis (Lundström, 1915) [Paasivirta 2012]
Chaetocladius gracilis Brundin, 1956 [Tuiskunen and Lindeberg 1986]
Chaetocladius grandilobus Brundin, 1956 [Paasivirta 2009]
Chaetocladius laminatus Brundin, 1947 [Hackman 1980]
Chaetocladius maeaeri Brundin, 1947 [Tuiskunen and Lindeberg 1986]
Chaetocladius sp. pr. *melaleucus* (Meigen, 1818) [Paasivirta 2009]
Chaetocladius muliebris Tuiskunen, 1986 [Paasivirta 2012]

Chaetocladius perennis (Meigen, 1830) [Hackman 1980]
Chaetocladius piger (Goetghebuer, 1913) [Hackman 1980]
Chaetocladius cf. *rusticus* (Goetghebuer, 1932) [Paasivirta 2012]
Chaetocladius suecicus (Kieffer, 1916) [Hackman 1980]
Chaetocladius tenuistylus Brundin, 1947 [Tuiskunen and Lindeberg 1986]
CLUNIO Haliday, 1855
Clunio balticus Heimbach, 1978 [Hackman 1980]
CORYNONEURA Winnertz, 1846
Corynoneura arctica Kieffer, 1923 [Hirvenoja and Hirvenoja 1988]
Corynoneura brundini Hirvenoja & Hirvenoja, 1988 [Hirvenoja and Hirvenoja 1988]
Corynoneura carriana Edwards, 1924 [Hackman 1980]
Corynoneura celeripes Winnertz, 1852 [Hackman 1980]
Corynoneura celtica Edwards, 1924 [Paasivirta 2009]
Corynoneura coronata Edwards, 1924 [Hackman 1980]
Corynoneura edwardsi Brundin, 1949 [Hackman 1980]
Corynoneura fittkaui Schlee, 1968 [Hackman 1980]
Corynoneura gratias Schlee, 1968 [Hirvenoja and Hirvenoja 1988]
Corynoneura gynocera Tuiskunen, 1983 [Tuiskunen 1983]
Corynoneura lacustris Edwards, 1924 [Hackman 1980]
Corynoneura lobata Edwards, 1924 [Hackman 1980]
Corynoneura magna Brundin, 1949 [Tuiskunen and Lindeberg 1986]
Corynoneura scutellata Winnertz, 1846 [Hackman 1980]
= *C. longipennis* auct. nec Tokunaga, 1936 [Hirvenoja and Hirvenoja 1988]
Corynoneura sp. 1 "Tvrminne"
Corynoneura sp. 2
CORYNONEURELLA Brundin, 1949
Corynoneurella paludosa Brundin, 1949 [Paasivirta 2012]
CRICOTOPUS van der Wulp, 1874
sg. ***Cricotopus*** van der Wulp, 1874
Cricotopus albiforceps (Kieffer, 1916) [Hirvenoja 1973, Hackman 1980]
Cricotopus annulator Goetghebuer, 1927 [Hirvenoja 1973, Hackman 1980]
Cricotopus bicinctus (Meigen, 1818) [Hackman 1980]
Cricotopus caducus Hirvenoja, 1973 [Hirvenoja 1973, Hackman 1980]
Cricotopus coronatus Hirvenoja, 1973 [Hirvenoja 1973, Hackman 1980]
Cricotopus cumulatus Hirvenoja, 1973 [Hackman 1980, Tuiskunen and Lindeberg 1986]

Cricotopus cylindraceus (Kieffer, 1908) [Hirvenoja 1973, Hackman 1980]
Cricotopus ephippium (Zetterstedt, 1838) [Hackman 1980]
Cricotopus festivellus (Kieffer, 1906) [Bagge et al. 1980, Hackman 1980]
Cricotopus flavocinctus (Kieffer, 1924) [Hirvenoja 1973, Hackman 1980]
Cricotopus fuscus (Kieffer, 1909) [Paasivirta 2009]
Cricotopus magus Hirvenoja, 1973 [Hirvenoja 1973, Hackman 1980]
Cricotopus pallidipes Edwards, 1929 [Paasivirta 2009]
Cricotopus patens Hirvenoja, 1973 [Hirvenoja 1973, Hackman 1980]
Cricotopus pilidorsum Hirvenoja, 1973 [Hirvenoja 1973, Hackman 1980]
Cricotopus pilosellus Brundin, 1956 [Hackman 1980]
Cricotopus pirifer Hirvenoja, 1973 [Tuiskunen and Lindeberg 1986]
Cricotopus polaris Kieffer, 1926 [Hirvenoja 1973, Hackman 1980]
Cricotopus sp., pr. *polaris*
Cricotopus pulchripes Verrall, 1912 [Tuiskunen and Lindeberg 1986]

Cricotopus septentrionalis Hirvenoja, 1973 [Hirvenoja 1973, Hackman 1980]
Cricotopus similis Goetghebuer, 1921 [Hirvenoja 1973, Hackman 1980]
Cricotopus slossonae Malloch, 1915
= *C. claripes* Hirvenoja, 1973 [Hirvenoja 1973, Hackman 1980]
Cricotopus tibialis (Meigen, 1804) [Hackman 1980]
Cricotopus tremulus (Linnaeus, 1758) [Hackman 1980]
Cricotopus triannulatus (Macquart, 1826) [Hirvenoja 1973, Hackman 1980]
Cricotopus trifascia Edwards, 1929 [Hirvenoja 1973, Hackman 1980]
Cricotopus tristis Hirvenoja, 1973 [Hirvenoja 1973, Hackman 1980]
Cricotopus vierriensis Goetghebuer, 1935 [Paasivirta 2012]
Cricotopus villosus Hirvenoja, 1973 [Hirvenoja 1973, Hackman 1980]
sg. ***Isocladius*** Kieffer, 1909
Cricotopus arcuatus Hirvenoja, 1973 [Hirvenoja 1973, Hackman 1980]
Cricotopus brevipalpis Kieffer, 1909 [Hirvenoja 1973, Hackman 1980]
Cricotopus intersectus (Staeger, 1839) [Hirvenoja 1973, Hackman 1980]
Cricotopus laetus Hirvenoja, 1973 [Hirvenoja 1973, Hackman 1980]
Cricotopus laricomalis Edwards, 1932 [Hirvenoja 1973, Hackman 1980]
Cricotopus maurii Spies & Sæther, 2004
= *C. polychaetus* Hirvenoja, 1989 preocc.[Hirvenoja 1989, Paasivirta 2002]
Cricotopus obnixus (Walker, 1856) [Hackman 1980]
Cricotopus sp. cf. *obnixus* Hirvenoja, 1973
Cricotopus obtusus Hirvenoja, 1973 [Hirvenoja 1973, Hackman 1980]
Cricotopus ornatus (Meigen, 1818) [Hirvenoja 1973, Hackman 1980]
Cricotopus perniger (Zetterstedt, 1850) [Hirvenoja 1973, Hackman 1980]
Cricotopus pilicauda Hirvenoja, 1973 [Hirvenoja 1973, Hackman 1980]
Cricotopus pilitarsis (Zetterstedt, 1850) [Hackman 1980]
Cricotopus reductus Hirvenoja, 1973 [Hirvenoja 1973, Hackman 1980]
Cricotopus relucens Hirvenoja, 1973 [Hirvenoja 1973, Hackman 1980]
Cricotopus reversus Hirvenoja, 1973 [Hirvenoja 1973, Hackman 1980]
Cricotopus suspiciosus Hirvenoja, 1973 [Hirvenoja 1973, Hackman 1980]
Cricotopus sylvestris (Fabricius, 1794) [Hackman 1980]
= *C. saxicola* Kieffer, 1916 [Hirvenoja 1973]
= *C. motitator* misid.[Hirvenoja 1973]
? = *C. annulipes* misid.[Hirvenoja 1973]
Cricotopus tricinctus (Meigen, 1818) [Hackman 1980]
Cricotopus trifasciatus (Meigen, 1810) [Hackman 1980]
sg. ***Nostococladius*** Ashe & Murray, 1980
Cricotopus lygropis Edwards, 1929 [Paasivirta 2009]
sg. ***?***
Corynoneura (?) sp. 1 ”Tapionaho”
DIPLOCLADIUS Kieffer, 1908
Diplocladius cultriger Kieffer, 1908 [Hackman 1980]
DONCRICOTOPUS Sæther, 1981
Doncricotopus dentatus Tuiskunen, 1985 [Tuiskunen 1986a]
DRATNALIA Sæther & Halvorsen, 1981
Dratnalia potamophylaxi (Fittkau & Lellak, 1971) [Paasivirta 2009]
EPOICOCLADIUS Sulc & Zavřel, 1924
Epoicocladius ephemerae (Kieffer, 1924)
= *E. flavens* auct. nec (Malloch, 1915) [Hackman 1980]
EUKIEFFERIELLA Thienemann, 1926

Eukiefferiella boevrensis Brundin, 1956 [Tuiskunen and Lindeberg 1986]
Eukiefferiella brevicalcar (Kieffer, 1911) [Tuiskunen and Lindeberg 1986]
Eukiefferiella claripennis (Lundbeck, 1898)
= *E. hospita* (Edwards, 1929) [Hackman 1980]
Eukiefferiella clypeata (Thienemann, 1919) [Paasivirta 2009]
Eukiefferiella devonica (Edwards, 1929) [Tuiskunen and Lindeberg 1986]
Eukiefferiella dittmari Lehmann, 1972 [Paasivirta 2009]
Eukiefferiella gracei (Edwards, 1929) [Paasivirta 2009]
Eukiefferiella sp. pr. *gracei*
Eukiefferiella ilkleyensis (Edwards, 1929) [Paasivirta 2012]
Eukiefferiella minor (Edwards, 1929) [Paasivirta 2009]
EURYCNEMUS van der Wulp, 1874
Eurycnemus crassipes (Meigen, 1810) [Paasivirta 2009]
GEORTHOCLADIUS Strenzke, 1941
sg. ***Georthocladius*** Strenzke, 1941
Georthocladius luteicornis (Goetghebuer, 1941) [Raunio and Langton 2005]
Georthocladius platystylus Sæther & Sublette, 1983 [Paasivirta 2012]
? = *Parachaetocladius retezati Albu, 1972*
GYMNOMETRIOCNEMUS Edwards, 1932
sg. ***Gymnometriocnemus*** Edwards, 1932
Gymnometriocnemus subnudus (Edwards, 1929) [Paasivirta 2002]
sg. ***Rhaphidocladius*** Sæther, 1983
Gymnometriocnemus brumalis (Edwards, 1929) [Tuiskunen and Lindeberg 1986]
Gymnometriocnemus volitans (Goetghebuer, 1940) [Hackman 1980]
HALOCLADIUS Hirvenoja, 1973
sg. ***Halocladius*** Hirvenoja, 1973
Halocladius variabilis (Staeger, 1839) [Hackman 1980]
HELENIELLA Gowin, 1943
Heleniella ornaticollis (Edwards, 1929) [Tuiskunen and Lindeberg 1986]
Heleniella serratosioi Ringe, 1976 [Paasivirta 2009]
HETEROTANYTARSUS Spärck, 1923
Heterotanytarsus apicalis (Kieffer, 1921) [Krogerus 1960, Hackman 1980]
Heterotanytarsus brundini Fittkau, 1956 [Tuiskunen and Lindeberg 1986]
Heterotanytarsus sp. 1
HETEROTRISSOCLADIUS Spärck, 1923
Heterotrissocladius brundini Sæther & Schnell, 1988 [Paasivirta 2009]
Heterotrissocladius grimshawi (Edwards, 1929) [Saether 1975, Hackman 1980]
Heterotrissocladius maeaeri Brundin, 1949 [Saether 1975, Hackman 1980]
Heterotrissocladius marcidus (Walker, 1856) [Saether 1975, Hackman 1980]
Heterotrissocladius subpilosus (Kieffer, 1911) [Saether 1975, Hackman 1980]
Heterotrissocladius sp. 1 ”Kolmper”
HYDROBAENUS Fries, 1830
Hydrobaenus conformis (Holmgren, 1869) [Hackman 1980]
Hydrobaenus fusistylus (Goetghebuer, 1933) [Paasivirta 2009]
Hydrobaenus lapponicus (Brundin, 1956) [Paasivirta 2009]
Hydrobaenus martini Sæther, 1976 [Hackman 1980]
Hydrobaenus pilipes (Malloch, 1915) [Bagge et al. 1980, Hackman 1980]
Hydrobaenus spinnatis Sæther, 1976 [Tuiskunen and Lindeberg 1986]
Hydrobaenus ? sp. 1
HYDROSMITTIA Ferrington & Sæther, 2011

Hydrosmittia oxoniana (Edwards, 1922)
= *H. recta* (Edwards, 1929) [Tuiskunen and Lindeberg 1986]
Hydrosmittia ruttneri Strenzke & Thienemann, 1942 [Tuiskunen and Lindeberg 1986]
= *H. brevitarsis* (Brundin, 1947) [Hackman 1980]
KRENOSMITTIA Thienemann & Krüger, 1939
Krenosmittia boreoalpina (Goetghebuer, 1944) [Paasivirta 2009]
Krenosmittia camptophleps (Edwards, 1929) [Paasivirta 2009]
Krenosmittia halvorseni (Cranston & Sæther, 1986) [Tuiskunen and Lindeberg 1986]
LAPPOKIEFFERIELLA Tuiskunen, 1986
Lappokiefferiella platytarsus Tuiskunen, 1986 [Tuiskunen and Lindeberg 1986]
LIMNOPHYES Eaton, 1875
Limnophyes aagaardi Sæther, 1990 [Paasivirta 2009]
Limnophyes angelicae Sæther, 1990 [Paasivirta 2009]
Limnophyes asquamatus Soegaard Andersen, 1937
= *L. smolandicus* Brundin, 1947 [Hackman 1980]
Limnophyes bidumus Sæther, 1990 [Paasivirta 2009]
Limnophyes sp., pr. *bidumus*
Limnophyes brachytomus (Kieffer, 1922) [Paasivirta 2009]
Limnophyes difficilis Brundin, 1947 [Tuiskunen and Lindeberg 1986]
Limnophyes edwardsi Sæther, 1990 [Paasivirta 2009]
Limnophyes er Sæther, 1985 [Saether 1985]
Limnophyes habilis (Walker, 1856)
= *L. truncorum* Goetghebuer, 1921 [Hackman 1980]
Limnophyes margaretae Sæther, 1975 [Paasivirta 2009]
Limnophyes minimus (Meigen, 1818)
= *L. exiguus* (Goetghebuer, 1913) [Hackman 1980]
Limnophyes natalensis (Kieffer, 1914) [Paasivirta 2002]
Limnophyes ninae Sæther, 1975 [Paasivirta 2009]
Limnophyes pentaplastus (Kieffer, 1921) [Paasivirta 2009]
Limnophyes pumilio (Holmgren, 1869)
= *L. globifer* Lundström, 1915 [Hackman 1980]
Limnophyes sp., pr. *pumilio*
Limnophyes schnelli Sæther, 1990 [Paasivirta 2009]
Limnophyes spinigus Sæther, 1990 [Paasivirta 2009]
Limnophyes torulus Sæther, 1990 [Paasivirta 2009]
MESOCRICOTOPUS Brundin, 1956
Mesocricotopus thienemanni (Goetghebuer, 1940) [Hackman 1980]
MESOSMITTIA Brundin, 1956
Mesosmittia flexuella (Edwards, 1929) [Hackman 1980]
METRIOCNEMUS van der Wulp, 1874
sg. ***Metriocnemus*** van der Wulp, 1874
Metriocnemus acutus Sæther, 1995 [Paasivirta 2009]
Metriocnemus albolineatus (Meigen, 1818)
= *M. atratulus* (Zetterstedt, 1850) [Hackman 1980]
Metriocnemus atriclava Kieffer, 1921 [Paasivirta 2002]
Metriocnemus beringensis (Cranston & Oliver, 1988) [Paasivirta 2009]
Metriocnemus caudigus Sæther, 1995 [Paasivirta 2009]
Metriocnemus corticalis Strenzke, 1950 [Paasivirta 2012]
Metriocnemus eurynotus (Holmgren, 1883) [Paasivirta 2009]
= *M. hygropetricus* Kieffer, 1912 [Hackman 1980]

Metriocnemus exilacies Sæther, 1995 [Paasivirta 2009]
Metriocnemus fuscipes (Meigen, 1818) [Hackman 1980]
Metriocnemus intergerivus Sæther, 1995 [Paasivirta 2009]
Metriocnemus picipes (Meigen, 1818) [Paasivirta 2002]
Metriocnemus ursinus (Holmgren, 1869) [Tuiskunen and Lindeberg 1986]
NANOCLADIUS Kieffer, 1913
sg. ***Microcricotopus*** Thienemann & Harnisch, 1932
sg. ***Nanocladius*** Kieffer, 1913
Nanocladius balticus (Palmén, 1959) [Hackman 1980]
Nanocladius dichromus (Kieffer, 1906)
= *N. bicolor* (Zetterstedt, 1838) nom. nudum[Hackman 1980]
Nanocladius parvulus (Kieffer, 1909) [Paasivirta 2009]
Nanocladius rectinervis (Kieffer, 1911) [Tuiskunen and Lindeberg 1986]
ORTHOCLADIUS van der Wulp, 1874
sg. ***Eudactylocladius*** Thienemann, 1935
Orthocladius fuscimanus (Kieffer, 1908) [Paasivirta 2009]
Orthocladius gelidorum (Kieffer, 1923) [Paasivirta 2009]
Orthocladius gelidus Kieffer, 1922 [Paasivirta 2009]
Orthocladius musester Sæther, 2004 [Paasivirta 2009]
Orthocladius olivaceus (Kieffer, 1911) [Tuiskunen and Lindeberg 1986]
= *O. mixtus* auct. nec Holmgren, 1869) in part[Hackman 1980]
Orthocladius priomixtus Sæther, 2004 [Paasivirta 2012]
= *O. mixtus* auct. nec Holmgren, 1869) in part[Hackman 1980]
sg. ***Euorthocladius*** Thienemann, 1935
Orthocladius abiskoensis Thienemann & Krüger, 1937 [Paasivirta 2009]
Orthocladius cf. *annallae* Sæther, 2005 [Paasivirta 2012]
Orthocladius ashei Soponis, 1990 [Paasivirta 2009]
Orthocladius rivicola Kieffer, 1911 [Paasivirta 2009]
Orthocladius rivulorum Kieffer, 1909 [Paasivirta 2009]
Orthocladius saxosus (Tokunaga, 1939) [Paasivirta 2009]
Orthocladius telochaetus Langton, 1985 [Paasivirta 2012]
sg. ***Mesorthocladius*** Sæther, 2005
Orthocladius frigidus (Zetterstedt, 1838) [Krogerus 1960]
sg. ***Orthocladius*** van der Wulp, 1874
Orthocladius decoratus (Holmgren, 1869) [Tuiskunen and Lindeberg 1986]
Orthocladius dentifer Brundin, 1947 [Hackman 1980]
Orthocladius excavatus Brundin, 1947 [Hackman 1980]
Orthocladius lapponicus Goetghebuer, 1940 [Paasivirta 2009]
Orthocladius cf. *nitidoscutellatus* Lundström, 1915 [Paasivirta 2012]
Orthocladius oblidens (Walker, 1856) [Bagge et al. 1980, Hackman 1980]
Orthocladius pedestris Kieffer, 1909 [Paasivirta 2009]
Orthocladius rhyacobius Kieffer, 1911 [Paasivirta 2009]
Orthocladius rubicundus (Meigen, 1818)
= *O. saxicola* Kieffer, 1911 [Tuiskunen and Lindeberg 1986]
Orthocladius wetterensis Brundin, 1956 [Paasivirta 2009]
sg. ***Pogonocladius*** Brundin, 1956
Orthocladius consobrinus (Holmgren, 1869) [Bagge et al. 1980, Hackman 1980]
sg. ***Symposiocladius*** Cranston, 1982
Orthocladius holsatus Goetghebuer, 1937 [Paasivirta 2009]
Orthocladius lignicola Kieffer, 1914 [Hackman 1980]

Orthocladius ruffoi Rossaro & Prato, 1991 [Paasivirta 2009]
Orthocladius schnelli Sæther, 2004 [Paasivirta 2009]
Orthocladius smolandicus Brundin, 1947 [Hackman 1980, Paasivirta 2009]
PARACHAETOCLADIUS Wuelker, 1959
Parachaetocladius abnobaeus (Wülker, 1959) [Paasivirta 2009]
PARACLADIUS Hirvenoja, 1973
Paracladius alpicola (Zetterstedt, 1850) [Tuiskunen and Lindeberg 1986]
Paracladius conversus (Walker, 1856) [Hackman 1980]
Paracladius quadrinodosus Hirvenoja, 1973 [Hackman 1980]
PARACRICOTOPUS Brundin, 1956
Paracricotopus niger (Kieffer, 1913) [Paasivirta 2009]
Paracricotopus uliginosus (Brundin, 1947) [Tuiskunen and Lindeberg 1986]
PARAKIEFFERIELLA Thienemann, 1936
Parakiefferiella bathophila (Kieffer, 1912) [Hackman 1980]
Parakiefferiella bilobata Tuiskunen, 1986 [Tuiskunen 1986d]
Parakiefferiella coronata (Edwards, 1929) [Hackman 1980]
Parakiefferiella fennica Tuiskunen, 1986 [Tuiskunen 1986d]
Parakiefferiella finnmarkica Tuiskunen, 1986 [Paasivirta 2009]
Parakiefferiella gynocera (Edwards, 1937) [Hackman 1980]
Parakiefferiella minuta Tuiskunen, 1986 [Tuiskunen 1986d]
Parakiefferiella nigra Brundin, 1949 [Hackman 1980]
Parakiefferiella scandica Brundin, 1956 [Tuiskunen 1986d]
Parakiefferiella smolandica (Brundin, 1947) [Bagge et al. 1980, Hackman 1980]
Parakiefferiella subaterrima (Malloch, 1915) [Paasivirta 2009]
= *P. torulata* Sæther, 1969
PARALIMNOPHYES Brundin, 1956
Paralimnophyes longiseta (Thienemann, 1919) [Paasivirta 2012]
= *P. hydrophilus* (Goetghebuer, 1921)
PARAMETRIOCNEMUS Goetghebuer, 1932
Parametriocnemus boreoalpinus Gowin & Thienemann, 1942 [Tuiskunen and Lindeberg 1986]
Parametriocnemus stylatus (Spärck, 1923) [Hackman 1980]
Parametriocnemus sp. 1
PARAPHAENOCLADIUS Thienemann, 1924
Paraphaenocladius exagitans (Johannsen, 1905) [Paasivirta 2009]
Paraphaenocladius sp., pr. *exagitans*
Paraphaenocladius impensus (Walker, 1856) [Hackman 1980]
Paraphaenocladius intercedens Brundin, 1947 [Paasivirta 2009]
Paraphaenocladius sp., pr. *intercedens*
Paraphaenocladius irritus (Walker, 1856) [Tuiskunen and Lindeberg 1986]
Paraphaenocladius pseudirritus Strenzke, 1950 [Paasivirta 2009]
Paraphaenocladius triangulus Sæther & Wang, 1995 [Paasivirta 2009]
PARASMITTIA Strenzke, 1950
Parasmittia carinata Strenzke, 1950 [Paasivirta 2009]
Parasmittia sp. 1 ”Luukki”
PARATRICHOCLADIUS Santos Abreu, 1918
Paratrichocladius rufiventris (Meigen, 1830) [Hackman 1980]
Paratrichocladius skirwithensis (Edwards, 1929) [Hackman 1980]
PARATRISSOCLADIUS Zavřel, 1937
Paratrissocladius excerptus (Walker, 1856) [Paasivirta 2009]

PROPSILOCERUS Kieffer, 1923
= ***Synpsilocerus*** Kieffer, 1923
= ***Tokunagayusurika*** Sasa, 1978
Propsilocerus jacuticus (Zvereva, 1950) [Meriläinen 1987]
Propsilocerus komensis (Zvereva, 1950) [Kosonen 1993]
? = *P. paradoxus* (Lundström, 1915)
Propsilocerus saetheri Wang, Liu & Paasivirta, 2007 [Paasivirta 2009]
PROSMITTIA Brundin, 1956
Prosmittia jemtlandica (Brundin, 1947) [Paasivirta and Koskenniemi 1984]
Prosmittia rectangularis Tuiskunen, 1985 [Tuiskunen 1985]
PSECTROCLADIUS Kieffer, 1906
sg. ***Allopsectrocladius*** Wülker, 1956
Psectrocladius conjungens (Brundin, 1947) [Paasivirta 2009]
Psectrocladius obvius (Walker, 1856) [Hackman 1980]
Psectrocladius platypus (Edwards, 1929) [Koskenniemi and Paasivirta 1987]
sg. ***Mesopsectrocladius*** Laville, 1971
Psectrocladius barbatipes Kieffer, 1923 [Hirvenoja 1998b]
sg. ***Monopsectrocladius*** Wülker, 1956
Psectrocladius calcaratus (Edwards, 1929) [Hackman 1980]
sg. ***Psectrocladius*** Kieffer, 1906
Psectrocladius barbimanus (Edwards, 1929) [Hackman 1980]
Psectrocladius bisetus Goetghebuer, 1942 [Tuiskunen and Lindeberg 1986]
Psectrocladius fennicus Storå, 1939 [Hackman 1980]
Psectrocladius limbatellus (Holmgren, 1869) [Bagge et al. 1980, Hackman 1980]
= *P. edwardsi* Brundin, 1949 [Koskenniemi and Paasivirta 1987]
Psectrocladius octomaculatus Wülker, 1956 [Hackman 1980]
Psectrocladius oligosetus Wülker, 1956 [Paasivirta 2009]
Psectrocladius oxyura Langton, 1985 [Paasivirta 2009]
Psectrocladius psilopterus (Kieffer, 1906) [Bagge et al. 1980, Hackman 1980]
Psectrocladius schlienzi Wülker, 1956 [Paasivirta 2009]
Psectrocladius sordidellus (Zetterstedt, 1838) [Hackman 1980]
Psectrocladius ventricosus Kieffer, 1925 [Hackman 1980]
Psectrocladius zetterstedti Brundin, 1949 [Hackman 1980]
PSEUDORTHOCLADIUS Goetghebuer, 1943
Pseudorthocladius curtistylus (Goetghebuer, 1921) [Tuiskunen and Lindeberg 1986]
Pseudorthocladius filiformis (Kieffer, 1908) [Paasivirta 2009]
Pseudorthocladius pilosipennis Brundin, 1956 [Tuiskunen and Lindeberg 1986]
PSEUDOSMITTIA Edwards, 1932
Pseudosmittia albipennis (Goetghebuer, 1921) [Paasivirta 2009]
Pseudosmittia angusta (Edwards, 1929) [Paasivirta 2009]
Pseudosmittia danconai (Marcuzzi, 1947) [Paasivirta 2009]
Pseudosmittia forcipata (Goetghebuer, 1921) [Hackman 1980]
Pseudosmittia sp., pr. *forcipata*
Pseudosmittia gracilis (Goetghebuer, 1913) [Paasivirta 2009]
Pseudosmittia mathildae Albu, 1968 [Paasivirta 2009]
Pseudosmittia obtusa Strenzke, 1960 [Paasivirta 2009]
Pseudosmittia trilobata (Edwards, 1929) [Hackman 1980]
PSILOMETRIOCNEMUS Sæther, 1969
Psilometriocnemus europaeus Tuiskunen, 1985 [Tuiskunen 1986a]
RHEOCRICOTOPUS Brundin, 1956

sg. ***Psilocricotopus*** Sæther, 1986
Rheocricotopus atripes (Kieffer, 1913) [Paasivirta 2009]
= *P. foveatus* (Edwards, 1929) [Lehmann 1969]
Rheocricotopus chalybeatus (Edwards, 1929) [Hackman 1980]
Rheocricotopus chapmani (Edwards, 1935) [Paasivirta 2012]
Rheocricotopus glabricollis (Meigen, 1830) [Paasivirta 2009]
sg. ***Rheocricotopus*** Brundin, 1956
Rheocricotopus effusus (Walker, 1856) [Hackman 1980]
Rheocricotopus fuscipes (Kieffer, 1909) [Hackman 1980]
= *P. dispar* (Goetghebuer, 1913) [Lehmann 1969]
Rheocricotopus reduncus Sæther & Schnell, 1988 [Paasivirta 2009]
Rheocricotopus sp. pr. *unidentatus* Sæther & Schnell, 1988 [Paasivirta 2012]
RHEOSMITTIA Brundin, 1986
Rheosmittia languida (Brundin, 1956) [Paasivirta 2009]
Rheosmittia spinicornis (Brundin, 1956) [Tuiskunen and Lindeberg 1986]
SMITTIA Holmgren, 1869
Smittia alpilonga Rossaro & Lencioni, 2000 [Paasivirta 2012]
Smittia cf. *amoena* Caspers, 1988 [Paasivirta 2012]
Smittia aterrima (Meigen, 1818) [Hackman 1980]
Smittia sp., pr. *aterrima*
Smittia betuletorum Edwards, 1941 [Tuiskunen and Lindeberg 1986]
Smittia contingens (Walker, 1856) [Paasivirta 2012]
Smittia edwardsi Goetghebuer, 1932 [Paasivirta 2009]
Smittia foliacea (Kieffer, 1921) [Paasivirta 2009]
Smittia leucopogon (Meigen, 1804) [Hackman 1980]
Smittia nudipennis (Goetghebuer, 1913) [Paasivirta 2009]
Smittia paranudipennis Brundin, 1947 [Paasivirta 2002]
Smittia pratorum (Goetghebuer, 1927) [Hackman 1980]
Smittia scutellosetosa Caspers, 1988 [Paasivirta 2009]
Smittia stercoraria Rossaro & Lencioni, 2000 [Paasivirta 2009]
Parasmittia sp. 1 1 sensu Tuiskunen
Parasmittia sp., pr. sp. 1
Parasmittia sp. 2 ”Jehkats”
STACKELBERGINA Shilova & Zelentsov, 1978
Stackelbergina praeclara Shilova & Zelentsov, 1978 [Paasivirta 2009]
STILOCLADIUS Rossaro, 1979
Stilocladius intermedius Wang, 1998 [Paasivirta 2012]
SYNORTHOCLADIUS Thienemann, 1935
Synorthocladius semivirens (Kieffer, 1909) [Hackman 1980]
TAVASTIA Tuiskunen, 1985
Tavastia australis Tuiskunen, 1985 [Tuiskunen 1986c]
Tavastia yggdrasilia Brodin, Lundström & Paasivirta, 2008 [Brodin et al. 2008]
THIENEMANNIA Kieffer, 1909
Thienemannia gracilis Kieffer, 1909 [Paasivirta 2009]
Thienemannia paasivirtai Tuiskunen, 1986 [Tuiskunen and Lindeberg 1986]
THIENEMANNIELLA Kieffer, 1911
Thienemanniella acuticornis (Kieffer, 1912) [Paasivirta 2009]
Thienemanniella clavicornis (Kieffer, 1911) [Liljaniemi et al. 2002]
Thienemanniella majuscula (Edwards, 1924) [Paasivirta 2009]
Thienemanniella minuscula (Brundin, 1949) [Hackman 1980]

Thienemanniella obscura Brundin, 1947 [Paasivirta 2009]
Thienemanniella vittata (Edwards, 1924) [Hackman 1980]
Thienemanniella sp., pr. *vittata*
TOKUNAGAIA Sæther, 1973
Tokunagaia excellens (Brundin, 1956) [Paasivirta 2009]
Tokunagaia parexcellens Tuiskunen, 1986 [Tuiskunen and Lindeberg 1986]
Tokunagaia rectangularis (Goetghebuer, 1940) [Tuiskunen and Lindeberg 1986]
Tokunagaia scutellata (Brundin, 1956) [Paasivirta 2009]
Tokunagaia tonollii (Rossaro, 1983) [Paasivirta 2009]
TRISSOCLADIUS Kieffer, 1908
Trissocladius brevipalpis Kieffer, 1908 [Hackman 1980]
TVETENIA Kieffer, 1922
Tvetenia bavarica (Goetghebuer, 1934) [Tuiskunen and Lindeberg 1986]
Tvetenia calvescens (Edwards, 1929) [Tuiskunen and Lindeberg 1986]
Tvetenia discoloripes (Goetghebuer & Thienemann, 1936) [Liljaniemi et al. 2002]
Tvetenia duodenaria Kieffer, 1922
= *T. saanensis* (Wülker, 1959) [Wülker 1959, Hackman 1980]
Tvetenia verralli (Edwards, 1929) [Tuiskunen and Lindeberg 1986]
? = *T. tshernovskii* (Pankratova, 1968)
VIVACRICOTOPUS Schnell & Sæther, 1988
Vivacricotopus ablusus Schnell & Sæther, 1988 [Paasivirta 2009]
ZALUTSCHIA Lipina, 1939
Zalutschia cf. *humphriesiae* Dowling & Murray, 1980 [Paasivirta 2012]
Zalutschia mallae Tuiskunen, 1986 [Tuiskunen and Lindeberg 1986]
Zalutschia mucronata (Brundin, 1949) [Hackman 1980]
Zalutschia tatrica (Pagast, 1935) [Hackman 1980]
Zalutschia tornetraeskensis (Edwards & Thienemann, 1941) [Hackman 1980]
Zalutschia zalutschicola Lipina, 1939 [Hackman 1980]
?
Orthocladiinae sp. 1 ”Outajrvi”
Orthocladiinae sp. 2 ”Pallas”
Orthocladiinae sp. 3 ”Kaldoaivi”
CHIRONOMINAE Newman, 1834
tribe Chironomini Newman, 1834
BENTHALIA Lipina, 1939
Benthalia carbonaria (Meigen, 1804) [Paasivirta 2009]
= *B. dissidens* (Walker, 1856) [Hackman 1980]
CHIRONOMUS Meigen, 1803
sg. ***Chaetolabis*** Townes, 1945
Chironomus macani Freeman, 1948 [Hackman 1980, Wülker 1999]
sg. ***Chironomus*** Meigen, 1803
Chironomus acerbus Hirvenoja, 1962 [Hirvenoja 1962d, Hackman 1980]
Chironomus acutiventris Wülker, Ryser & Scholl, 1983 [Wülker 1999]
Chironomus agilis Shobanov & Dyomin, 1988 [Paasivirta 2012]
Chironomus annularius Meigen, 1818 [Hackman 1980]
Chironomus anthracinus Zetterstedt, 1860 [Hackman 1980]
Chironomus aprilinus Meigen, 1818 [Paasivirta 2012]
Chironomus beljaninae Wülker, 1991 [Wülker 1991]
Chironomus borokensis Kerkis, Filippova, Shobanov, Gunderina & Kiknadze, 1988 [Wülker 1999]

Chironomus brevidentatus Hirvenoja & Michailova, 1998 [Hirvenoja and Michailova 1998]
Chironomus cingulatus Meigen, 1830 [Hackman 1980]
Chironomus clarus Hirvenoja, 1962 [Hirvenoja 1962d, Hackman 1980]
Chironomus coaetaneus Hirvenoja, 1998 [Hirvenoja 1998a]
Chironomus dorsalis Strenzke, 1959 [Hackman 1980]
Chironomus entis Shobanov, 1989 [Paasivirta 2012]
Chironomus esai Wülker, 1997 [Wülker 1997]
Chironomus fraternus Wülker, 1991 [Wülker 1991]
Chironomus heteropilicornis Wülker, 1996 [Wülker 1999]
? *Chironomus hyperboreus* Staeger, 1845 [Hackman 1980]
Chironomus inermifrons Goetghebuer, 1921 [Paasivirta and Koskenniemi 1984]
Chironomus islandicus (Kieffer, 1913) [Paasivirta 2009]
Chironomus jonmartini Lindeberg, 1979 coll. [Hackman 1980]
= *C. neglectus* Lindeberg, 1960 preocc.
Chironomus longistylus Goetghebuer, 1921 [Paasivirta and Koskenniemi 1984]
Chironomus lugubris Zetterstedt, 1850 [Hackman 1980, Wülker et al. 1981]
Chironomus luridus Strenzke, 1959 [Hirvenoja 2002]
Chironomus melanescens Keyl, 1961 [Wülker 1999]
Chironomus melanotus Keyl, 1961 [Paasivirta 1979, Wülker 1999]
Chironomus muratensis Ryser, Scholl & Wülker, 1983 [Wülker 1999]
Chironomus neocorax Wuelker & Butler, 1983 [Wülker and Butler 1983]
Chironomus pallidivittatus Edwards, 1929 [Hackman 1980]
Chironomus piger Strenzke, 1956 [Hackman 1980]
Chironomus pilicornis (Fabricius, 1787) [Hackman 1980]
Chironomus plumosus (Linnaeus, 1758) [Krogerus 1960, Hackman 1980]
Chironomus pseudothummi Strenzke, 1959 coll. [Hirvenoja 1962c, Hackman 1980]
Chironomus riihimakiensis Wülker, 1973 [Wülker 1973, Hackman 1980]
Chironomus riparius Meigen, 1804 [Hirvenoja and Michailova 1991]
Chironomus salinarius Kieffer, 1915 [Paasivirta 2009]
Chironomus saxatilis Wülker, Ryser & Scholl, 1981 coll. [Wülker et al. 1981]
Chironomus sollicitus Hirvenoja, 1962 [Hirvenoja 1962c, Hackman 1980]
Chironomus sororius Wülker, 1973 [Wülker 1999]
? *Chironomus staegeri* Lundbeck, 1898 [Hackman 1980]
Chironomus tentans Fabricius, 1805 [Hackman 1980]
Chironomus tenuistylus Brundin, 1949 [Paasivirta 1979, Hackman 1980]
sg. ***Lobochironomus*** Ryser, Wülker & Scholl, 1985
Chironomus dorsalis Meigen, 1818
= *C. longipes* Staeger, 1839 [Hackman 1980]
Chironomus improvidus Hirvenoja, 1998 [Hirvenoja 1998b]
Chironomus mendax Storå, 1936 [Hackman 1980]
Chironomus storai Goetghebuer, 1954 [Hackman 1980]
= *C. luctuosus* Storå, 1936 preocc.[Hackman 1980]
CLADOPELMA Kieffer, 1921
Cladopelma bicarinatum (Brundin, 1947) [Hackman 1980]
Cladopelma edwardsi (Kruseman, 1933) [Hackman 1980]
Cladopelma goetghebueri Spies & Sæther, 2004 [Paasivirta 2009]
= *C. laterale* (Goetghebuer, 1934) preocc.
Cladopelma virescens (Meigen, 1818) [Bagge et al. 1980]
Cladopelma viridulum (Linnaeus, 1767) [Hackman 1980]

CRYPTOCHIRONOMUS Kieffer, 1918
Cryptochironomus albofasciatus (Staeger, 1839) [Hackman 1980]
Cryptochironomus denticulatus (Goetghebuer, 1921) [Paasivirta 2009]
Cryptochironomus obreptans (Walker, 1856) [Paasivirta 2009]
Cryptochironomus psittacinus (Meigen, 1830) [Hackman 1980]
Cryptochironomus redekei (Kruseman, 1933) [Hackman 1980]
Cryptochironomus rostratus Kieffer, 1921 [Hackman 1980]
Cryptochironomus supplicans (Meigen, 1830) [Hackman 1980]
Cryptochironomus ussouriensis (Goetghebuer, 1933) [Hackman 1980]
= *C. nigridens* Chernovskij, 1949
CRYPTOTENDIPES Beck & Beck, 1969
Cryptotendipes darbyi (Sublette, 1960) [Paasivirta 2009]
Cryptotendipes pflugfelderi Reiss, 1964 [Hackman 1980]
Cryptotendipes pseudotener (Goetghebuer, 1922) [Paasivirta 2009]
Cryptotendipes usmaensis (Pagast, 1931) [Hackman 1980]
DEMEIJEREA Kruseman, 1933
Demeijerea rufipes (Linnaeus, 1761) [Hackman 1980]
DEMICRYPTOCHIRONOMUS Lenz, 1941
sg. ***Demicryptochironomus*** Lenz, 1941
Demicryptochironomus vulneratus (Zetterstedt, 1838) [Hackman 1980]
Demicryptochironomus sp. Pe 1 Langton, 1991
sg. ***Irmakia*** Reiss, 1988
Demicryptochironomus neglectus Reiss, 1988 [Paasivirta 2009]
DICROTENDIPES Kieffer, 1913
= ***Limnochironomus*** Kieffer, 1920
Dicrotendipes lobiger (Kieffer, 1921) [Hackman 1980]
Dicrotendipes nervosus (Staeger, 1839) [Hackman 1980]
Dicrotendipes notatus (Meigen, 1818) [Paasivirta 2009]
Dicrotendipes pulsus (Walker, 1856) [Hackman 1980]
= *D. objectans* (Walker, 1856)
Dicrotendipes tritomus (Kieffer, 1916) [Hackman 1980]
EINFELDIA Kieffer, 1924
Einfeldia pagana (Meigen, 1838) [Hackman 1980]
Einfeldia pectoralis Kieffer, 1924 sensu Hirvenoja, 1998 [Paasivirta 2002]
ENDOCHIRONOMUS Kieffer, 1918
Endochironomus albipennis (Meigen, 1830) [Hackman 1980]
Endochironomus stackelbergi Goetghebuer, 1935 [Paasivirta 2012]
Endochironomus tendens (Fabricius, 1775) [Hackman 1980]
GLYPTOTENDIPES Kieffer, 1913
sg. ***Caulochironomus*** Heyn, 1993
Glyptotendipes aequalis (Kieffer, 1922) [Hirvenoja and Michailova 1991]
Glyptotendipes caulicola (Kieffer, 1913) [Paasivirta 2012]
Glyptotendipes imbecilis (Walker, 1856) [Paasivirta 2012]
= *G. severini* (Goetghebuer, 1923)
Glyptotendipes scirpi (Kieffer, 1915)
= *G. fodiens* (Kieffer, 1924) [Paasivirta 2012]
= *G. mancunianus* (Edwards, 1929)
Glyptotendipes viridis (Macquart, 1834) [Paasivirta 2009]
sg. ***Glyptotendipes*** Kieffer, 1913
Glyptotendipes barbipes (Staeger, 1839) [Hackman 1980]

Glyptotendipes cauliginellus (Kieffer, 1913)
= *G. gripekoveni* (Kieffer, 1913) [Hackman 1980]
Glyptotendipes glaucus (Meigen, 1818) [Paasivirta 2009]
Glyptotendipes pallens (Meigen, 1804) [Hackman 1980]
Glyptotendipes paripes (Edwards, 1929) [Hackman 1980]
sg. ***Heynotendipes*** Spies & Sæther, 2004
Glyptotendipes signatus (Kieffer, 1909) [Hackman 1980]
HARNISCHIA Kieffer, 1921
Harnischia curtilamellata (Malloch, 1915) [Hackman 1980]
Harnischia fuscimana Kieffer, 1921 [Paasivirta 2009]
KIEFFERULUS Goetghebuer, 1922
sg. ***Kiefferulus*** Goetghebuer, 1922
Kiefferulus tendipediformis (Goetghebuer, 1921) [Paasivirta 2009]
KLOOSIA Kruseman, 1933
Kloosia pusilla (Linnaeus, 1767) [Paasivirta 2009]
LAUTERBORNIELLA Thienemann & Bause, 1913
Lauterborniella agrayloides (Kieffer, 1911) [Hackman 1980]
LIPINIELLA Shilova, 1961
? *Lipiniella araenicola* Shilova, 1961 [Paasivirta 2012]
Lipiniella prima Shilova, Kerkis & Kiknadze, 1993 [Paasivirta 2009]
MICROCHIRONOMUS Kieffer, 1918
Microchironomus tener (Kieffer, 1918) [Hackman 1980]
MICROTENDIPES Kieffer, 1915
Microtendipes brevitarsis Brundin, 1947 [Paasivirta 2012]
Microtendipes chloris (Meigen, 1818) [Hackman 1980]
Microtendipes confinis (Meigen, 1830) [Paasivirta 1983]
Microtendipes nigellus Hirvenoja, 1963 [Hirvenoja 1963a, Hackman 1980]
Microtendipes pedellus (De Geer, 1776) [Hackman 1980]
Microtendipes rydalensis (Edwards, 1929) [Paasivirta 2009]
NILOTHAUMA Kieffer, 1921
Nilothauma brayi (Goetghebuer, 1921) [Hackman 1980]
OMISUS Townes, 1945
Omisus caledonicus (Edwards, 1932) [Hackman 1980]
PAGASTIELLA Brundin, 1949
Pagastiella orophila (Edwards, 1929) [Hackman 1980, Tuiskunen and Lindeberg 1986]
PARACHIRONOMUS Lenz, 1921
Parachironomus biannulatus (Staeger, 1839) [Hackman 1980]
Parachironomus digitalis (Edwards, 1929) [Hackman 1980]
Parachironomus frequens (Johannsen, 1905) [Hackman 1980]
Parachironomus gracilior (Kieffer, 1918) [Paasivirta 2012]
= *P. arcuatus* (Goetghebuer, 1919)
Parachironomus monochromus (van der Wulp, 1874) [Hackman 1980]
Parachironomus paradigitalis Brundin, 1949 [Hackman 1980]
Parachironomus parilis (Walker, 1856) [Lehmann 1970b, Hackman 1980]
Parachironomus cf.*pseudovarius* Zorina, 2003
Parachironomus siljanensis Brundin, 1949 [Paasivirta 2009]
Parachironomus subalpinus (Goetghebuer, 1932) [Tuiskunen and Lindeberg 1986]
Parachironomus tenuicaudatus (Malloch, 1915) [Paasivirta 2002]
Parachironomus varus (Goetghebuer, 1921) [Paasivirta 2009]
Parachironomus vitiosus (Goetghebuer, 1921) [Lehmann 1970b, Hackman 1980]

Parachironomus sp. 1 "Iijoki" Zorina, 2003
PARACLADOPELMA Harnisch, 1923
Paracladopelma camptolabis (Kieffer, 1913) [Hackman 1980]
Paracladopelma galaptera (Townes, 1945) [Hackman 1980]
Paracladopelma laminatum (Kieffer, 1921) [Hackman 1980]
Paracladopelma nereis (Townes, 1945)
Paracladopelma nigritulum (Goetghebuer, 1942) [Hackman 1980]
Paracladopelma undine (Townes, 1945) [Paasivirta 2009]
PARALAUTERBORNIELLA Lenz, 1941
Paralauterborniella nigrohalteralis (Malloch, 1915) [Hackman 1980]
PARATENDIPES Kieffer, 1911
Paratendipes albimanus (Meigen, 1818) [Hackman 1980]
Paratendipes subaequalis (Malloch, 1915) [Paasivirta 2012]
Paratendipes conn. no. 3 Lipina, 1926 (larval type)
PHAENOPSECTRA Kieffer, 1921
Phaenopsectra flavipes (Meigen, 1818) [Palmén 1955, Hackman 1980]
Phaenopsectra punctipes (Wiedemann, 1817) [Hackman 1980]
POLYPEDILUM Kieffer, 1912
sg. ***Pentapedilum*** Kieffer, 1913
Polypedilum sordens (van der Wulp, 1874) [Hackman 1980]
Polypedilum tritum (Walker, 1856) [Hackman 1980]
? = *P. uncinatum* (Goetghebuer, 1921) [Hackman 1980]
sg. ***Polypedilum*** Kieffer, 1912
Polypedilum acutum Kieffer, 1915 [Paasivirta 2009]
Polypedilum albicorne (Meigen, 1838) [Tuiskunen and Lindeberg 1986]
Polypedilum amoenum Goetghebuer, 1930 [Paasivirta 2012]
Polypedilum arundineti (Goetghebuer, 1921) [Tuiskunen and Lindeberg 1986]
Polypedilum laetum (Meigen, 1818) [Hackman 1980]
Polypedilum nubeculosum (Meigen, 1804) [Hackman 1980]
Polypedilum pedestre (Meigen, 1830) [Hackman 1980]
Polypedilum trigonus Townes, 1945 [Paasivirta 2009]
sg. ***Tripodura*** Townes, 1945
Polypedilum aegyptium Kieffer, 1925 [Paasivirta 2009]
Polypedilum bicrenatum Kieffer, 1921 [Hirvenoja 1962e, Hackman 1980]
Polypedilum pullum (Zetterstedt, 1838) [Hackman 1980]
Polypedilum quadriguttatum Kieffer, 1921 [Paasivirta 2009]
Polypedilum scalaenum (Schrank, 1803) [Hackman 1980]
Polypedilum tetracrenatum Hirvenoja, 1962 [Hirvenoja 1962e, Hackman 1980]
sg. ***Uresipedilum*** Sasa & Kikuchi, 1995
Polypedilum convictum (Walker, 1856) [Hackman 1980]
Polypedilum cultellatum Goetghebuer, 1931 [Hackman 1980]
ROBACKIA Sæther, 1977
Robackia pilicauda Sæther, 1977
? *Robackia demeijerei* (Kruseman, 1933) [Liljaniemi et al. 2002]
SAETHERIA Jackson, 1977
Saetheria reissi Jackson, 1977 [Paasivirta and Koskenniemi 1984]
SERGENTIA Kieffer, 1922
Sergentia baueri Wülker, Kiknadze, Kerkis & Nevers, 1999 [Paasivirta 2009]
Sergentia coracina (Zetterstedt, 1850) [Hackman 1980]
Sergentia prima Proviz & Proviz, 1997 [Paasivirta 2012]

STENOCHIRONOMUS Kieffer, 1919
sg. ***Petalopholeus*** Borkent, 1984
Stenochironomus fascipennis (Zetterstedt, 1838) [Hackman 1980]
sg. ***Stenochironomus*** Kieffer, 1919
Stenochironomus gibbus (Fabricius, 1794) [Hackman 1980]
Stenochironomus hibernicus (Edwards, 1929) [Hackman 1980]
STICTOCHIRONOMUS Kieffer, 1919
Stictochironomus crassiforceps (Kieffer, 1921) [Hackman 1980]
Stictochironomus maculipennis (Meigen, 1818) [Paasivirta 2009]
Stictochironomus pictulus (Meigen, 1830) [Hackman 1980]
Stictochironomus rosenschoeldi (Zetterstedt, 1838) [Hackman 1980]
Stictochironomus sticticus (Fabricius, 1781) [Hackman 1980]
SYNENDOTENDIPES Grodhaus, 1987
Synendotendipes dispar (Meigen, 1830) [Hackman 1980]
Synendotendipes impar (Walker, 1856) [Krogerus 1960, Hackman 1980]
Synendotendipes lepidus (Meigen, 1830) [Paasivirta and Koskenniemi 1984]
TRIBELOS Townes, 1945
Tribelos cf. *donatoris* (Shilova, 1974)
? = *T. subatrum* Grodhaus, 1987
Tribelos intextum (Walker, 1856) [Hackman 1980]
XENOCHIRONOMUS Kieffer, 1921
Xenochironomus xenolabis (Kieffer, 1916) [Hackman 1980]
ZAVRELIELLA Kieffer, 1920
Zavreliella marmorata (van der Wulp, 1859) [Leppä and Hämäläinen 2002]
tribe Pseudochironomini Sæther, 1977
PSEUDOCHIRONOMUS Malloch, 1915
Pseudochironomus prasinatus (Staeger, 1839) [Hackman 1980]
tribe Tanytarsini Zavřel, 1917
CLADOTANYTARSUS Kieffer, 1921
sg. ***Cladotanytarsus*** Kieffer, 1921
Cladotanytarsus atridorsum Kieffer, 1924 [Palmén 1955, Hackman 1980]
Cladotanytarsus cyrylae Giłka, 2001 [Paasivirta 2009]
Cladotanytarsus difficilis Brundin, 1947 [Hackman 1980]
Cladotanytarsus gedanicus Giłka, 2001 [Paasivirta 2009]
Cladotanytarsus iucundus Hirvenoja, 1962 [Hirvenoja 1962a, Hackman 1980]
Cladotanytarsus lepidocalcar Krüger, 1938 [Hackman 1980]
Cladotanytarsus mancus (Walker, 1856) [Palmén 1955, Hackman 1980]
Cladotanytarsus matthei Giłka, 2001 [Paasivirta 2009]
Cladotanytarsus molestus Hirvenoja, 1962 [Hirvenoja 1962a, Hackman 1980]
Cladotanytarsus nigrovittatus (Goetghebuer, 1922) [Lindeberg 1964, Hackman 1980]
Cladotanytarsus pallidus Kieffer, 1922 [Hackman 1980]
Cladotanytarsus teres Hirvenoja, 1962 [Hirvenoja 1962a, Hackman 1980]
Cladotanytarsus vanderwulpi (Edwards, 1929) [Hackman 1980]
sg. ***Lenziella*** Kieffer, 1922
Cladotanytarsus amandus Hirvenoja, 1962 [Hirvenoja 1962a, Hackman 1980]
Cladotanytarsus bicornutus Kieffer, 1922
= *C. wexionensis* Brundin, 1947 [Hackman 1980]
CONSTEMPELLINA Brundin, 1947
Constempellina brevicosta (Edwards, 1937) [Lindeberg 1970, Hackman 1980]
CORYNOCERA Zetterstedt, 1837

Corynocera ambigua Zetterstedt, 1837 [Hackman 1980]
Corynocera oliveri Lindeberg, 1970 [Lindeberg 1970, Hackman 1980]
MICROPSECTRA Kieffer, 1908
= ***Lauterbornia*** Kieffer, 1911
= ***Lundstroemia*** Kieffer, 1921
= ***Parapsectra*** Reiss, 1969
Micropsectra appendica Stur & Ekrem, 2006 [Stur and Ekrem 2006]
Micropsectra apposita (Walker, 1856) [Säwedal 1976, Hackman 1980]
Micropsectra atrofasciata (Kieffer, 1911) [Lindeberg 1970, Hackman 1980]
Micropsectra attenuata Reiss, 1969 [Paasivirta 2009]
Micropsectra calcifontis Stur & Ekrem, 2006 [Paasivirta 2009]
Micropsectra chionophila (Edwards, 1933) [Paasivirta 2009]
Micropsectra insignilobus Kieffer, 1924 [Säwedal 1976, Hackman 1980]
Micropsectra junci (Meigen, 1818) [Säwedal 1976, Hackman 1980]
Micropsectra klinki Stur & Ekrem, 2006 [Stur and Ekrem 2006]
Micropsectra lacustris Säwedal, 1975 [Paasivirta 2009]
Micropsectra lindebergi Säwedal, 1976 [Säwedal 1976, Hackman 1980]
Micropsectra logani (Johannsen, 1928)
= *M. groenlandica* Soegaard Andersen, 1937 [Lindeberg 1970, Hackman 1980]
Micropsectra malla Giłka & Paasivirta, 2008 [Gika and Paasivirta 2008]
Micropsectra nana (Meigen, 1818) [Lindeberg 1970, Hackman 1980]
Micropsectra notescens (Walker, 1856) [Paasivirta 2009]
Micropsectra pallidula (Meigen, 1830) [Stur and Ekrem 2006]
= *M. bidentata* misid. [Lindeberg 1970, Hackman 1980, Stur and Ekrem 2006]
Micropsectra radialis Goetghebuer, 1939
= *M. coracina* (Kieffer, 1911) [Krogerus 1960, Hackman 1980]
Micropsectra recurvata Goetghebuer, 1928 [Lindeberg 1970, Hackman 1980]
Micropsectra rilensis Giłka, 2001 [Paasivirta 2009]
Micropsectra roseiventris (Kieffer, 1909)
= *M. fusca* auct. nec (Meigen, 1804) [Hackman 1980]
Micropsectra schrankelae Stur & Ekrem, 2006 [Paasivirta 2009]
Micropsectra sofiae Stur & Ekrem, 2006 [Stur and Ekrem 2006]
Micropsectra styriaca Reiss, 1969 [Paasivirta 2012]
NEOZAVRELIA Goetghebuer & Thienemann, 1941
Neozavrelia cuneipennis (Edwards, 1929) [Paasivirta 2009]
PARATANYTARSUS Thienemann & Bause, 1913
Paratanytarsus abiskoensis Reiss & Säwedal, 1981 [Paasivirta 2009]
Paratanytarsus austriacus (Kieffer, 1924) [Lindeberg 1970, Hackman 1980]
Paratanytarsus bituberculatus (Edwards, 1929) [Paasivirta 2009]
Paratanytarsus brevicalcar (Kieffer, 1909)
= *P. intricatus* (Goetghebuer, 1921) [Hackman 1980]
Paratanytarsus dimorphis Reiss, 1965 [Paasivirta 2009]
Paratanytarsus dissimilis (Johannsen, 1905)
= *P. confusus* Palmén, 1960 [Hackman 1980]
Paratanytarsus grimmii (Schneider, 1885)
= *P. boiemicus* Bause, 1913 [Hackman 1980]
Paratanytarsus hyperboreus Brundin, 1949 [Hackman 1980]
Paratanytarsus inopertus (Walker, 1856) [Hackman 1980]
Paratanytarsus laccophilus (Edwards, 1929) [Paasivirta 2012]
Paratanytarsus laetipes (Zetterstedt, 1850) [Bagge et al. 1980, Hackman 1980]

Paratanytarsus lauterborni (Kieffer, 1909) [Lindeberg 1970, Hackman 1980]
Paratanytarsus natvigi (Goetghebuer, 1933) [Hackman 1980]
Paratanytarsus paralaccophilus Giłka & Paasivirta, 2008 [Gika and Paasivirta 2008]
Paratanytarsus penicillatus (Goetghebuer, 1928) [Hackman 1980]
Paratanytarsus tenellulus (Goetghebuer, 1921) [Säwedal and Langton 1977, Hackman 1980]
Paratanytarsus tenuis (Meigen, 1830) [Hackman 1980]
RHEOTANYTARSUS Thienemann & Bause, 1913
Rheotanytarsus curtistylus (Goetghebuer, 1921) [Paasivirta 2009]
Rheotanytarsus muscicola Thienemann, 1929 [Paasivirta 2009]
Rheotanytarsus pellucidus (Walker, 1848)
= *R. distinctissimus* (Brundin, 1947) [Lehmann 1970a, Hackman 1980]
Rheotanytarsus pentapoda (Kieffer, 1909) [Paasivirta 2009]
Rheotanytarsus photophilus (Goetghebuer, 1921) [Kuusela 1979]
Rheotanytarsus ringei Lehmann, 1970 [Lehmann 1970a, Hackman 1980]
STEMPELLINA Thienemann & Bause, 1913
Stempellina almi Brundin, 1947 [Hackman 1980]
Stempellina bausei (Kieffer, 1911) [Hackman 1980]
Stempellina subglabripennis (Brundin, 1947) [Hackman 1980]
Stempellina tervolae Giłka, 2005 [Gika 2005]
STEMPELLINELLA Brundin, 1947
Stempellinella brevis (Edwards, 1929) [Hackman 1980]
Stempellinella edwardsi Spies & Sæther, 2004
= *S. minor* (Edwards, 1929) [Hackman 1980] preocc.
Stempellinella flavidula (Edwards, 1929) [Paasivirta 2009]
Stempellinella saltuum (Goetghebuer, 1921) [Paasivirta 2009]
TANYTARSUS van der Wulp, 1874
Tanytarsus aberrans Lindeberg, 1970 [Hackman 1980]
Tanytarsus aculeatus Brundin, 1949 [Hackman 1980]
Tanytarsus anderseni Reiss & Fittkau, 1971 [Paasivirta 2009]
Tanytarsus bathophilus Kieffer, 1911 [Hackman 1980]
= *T. tripunctatus* Reiss, 1968 [Lindeberg 1970]
Tanytarsus brundini Lindeberg, 1963 [Hirvenoja 1963c, Hackman 1980]
Tanytarsus buchonius Reiss & Fittkau, 1971 [Hackman 1980]
Tanytarsus chinyensis Goetghebuer, 1934 [Hackman 1980]
Tanytarsus curticornis Kieffer, 1911 [Hackman 1980]
Tanytarsus debilis (Meigen, 1830) [Hackman 1980]
Tanytarsus desertor Giłka & Paasivirta, 2007 [Paasivirta 2009]
Tanytarsus dibranchius Kieffer, 1926
= *T. separabilis* Brundin, 1947 [Hackman 1980]
Tanytarsus dispar Lindeberg, 1967 [Lindeberg 1967, Hackman 1980]
Tanytarsus eminulus (Walker, 1856) [Lindeberg 1976, Hackman 1980]
Tanytarsus excavatus Edwards, 1929 [Hackman 1980]
Tanytarsus fennicus Lindeberg, 1970 [Lindeberg 1970, Hackman 1980]
Tanytarsus gibbosiceps Kieffer, 1922 [Paasivirta 2009]
Tanytarsus glabrescens Edwards, 1929 [Hackman 1980]
Tanytarsus gracilentus (Holmgren, 1883) [Hackman 1980]
Tanytarsus gregarius Kieffer, 1909 [Lindeberg 1970, Hackman 1980]
Tanytarsus heusdensis Goetghebuer, 1923 [Paasivirta 2009]
Tanytarsus inaequalis Goetghebuer, 1921 [Bagge et al. 1980, Hackman 1980]

= *T. gregarius* sensu Brundin, 1947 [Lindeberg 1970]
Tanytarsus innarensis Brundin, 1947 [Hackman 1980]
Tanytarsus lactescens Edwards, 1929 [Hackman 1980]
Tanytarsus lapponicus Lindeberg, 1970 [Lindeberg 1970, Hackman 1980]
Tanytarsus latiforceps Edwards, 1941 [Tuiskunen and Lindeberg 1986]
Tanytarsus lestagei Goetghebuer, 1922 [Hackman 1980]
= *T. decipiens* Lindeberg, 1967 [Lindeberg 1967, Hackman 1980]
= *T. palmeni* Lindeberg, 1967 [Lindeberg 1967, Hackman 1980]
Tanytarsus longitarsis Kieffer, 1911 [Hackman 1980]
Tanytarsus lugens (Kieffer, 1916) [Reiss and Fittkau 1971, Hackman 1980]
Tanytarsus mancospinosus Ekrem & Reiss, 1999 [Paasivirta 2009]
Tanytarsus medius Reiss & Fittkau, 1971 [Hackman 1980]
Tanytarsus mendax Kieffer, 1925 [Reiss and Fittkau 1971, Hackman 1980]
= *T. holochlorus* Edwards, 1929
Tanytarsus miriforceps (Kieffer, 1921) [Hackman 1980]
Tanytarsus multipunctatus Brundin, 1947 [Hackman 1980]
Tanytarsus nemorosus Edwards, 1929 [Hackman 1980]
Tanytarsus niger Soegaard Andersen, 1937 [Lindeberg 1970, Hackman 1980]
Tanytarsus norvegicus (Kieffer, 1924) [Lindeberg 1970, Hackman 1980]
Tanytarsus occultus Brundin, 1949 [Bagge et al. 1980, Hackman 1980]
Tanytarsus palettaris Verneaux, 1969 [Hackman 1980, Hirvenoja 2002]
Tanytarsus pallidicornis (Walker, 1856) [Paasivirta 2002]
Tanytarsus paraniger Giłka & Paasivirta, 2008 [Gika and Paasivirta 2008]
Tanytarsus quadridentatus Brundin, 1947 [Lindeberg 1970, Reiss and Fittkau 1971, Hackman 1980]
Tanytarsus recurvatus Brundin, 1947 [Hackman 1980]
Tanytarsus salmelai Giłka & Paasivirta, 2009 [Gika and Paasivirta 2009]
Tanytarsus signatus (van der Wulp, 1859) [Hackman 1980]
Tanytarsus smolandicus Brundin, 1947 [Hackman 1980]
Tanytarsus striatulus Lindeberg, 1976 [Lindeberg 1976, Hackman 1980]
Tanytarsus sylvaticus (van der Wulp, 1859) [Hackman 1980]
= *T. aptus* Hirvenoja, 1963 [Hirvenoja 1963b]
Tanytarsus telmaticus Lindeberg, 1959 [Hackman 1980]
= *T. simulans* Lindeberg, 1967 [Lindeberg 1967, Hackman 1980]
= *T. socialis* Lindeberg, 1967 [Lindeberg 1967, Hackman 1980]
Tanytarsus trux Giłka & Paasivirta, 2007 [Paasivirta 2009]
Tanytarsus usmaensis Pagast, 1931 [Reiss and Fittkau 1971, Hackman 1980]
Tanytarsus verralli Goetghebuer, 1928 [Lindeberg 1970, Reiss and Fittkau 1971, Hackman 1980]
Tanytarsus volgensis Miseiko, 1967 [Hackman 1980]
= *T. fimbriatus* Reiss & Fittkau, 1971
VIRGATANYTARSUS Pinder, 1982
Virgatanytarsus arduennensis (Goetghebuer, 1922) [Reiss and Fittkau 1971, Hackman 1980]
ZAVRELIA Kieffer, Thienemann & Bause, 1913
Zavrelia pentatoma Kieffer & Bause, 1913 [Kuusela 1979]
= *Z. atrofasciata* Kieffer, 1921 [Hackman 1980]

SIMULIIDAE Newman, 1834

Supporting references for *Ilmonen J (2014) Checklist of the family Simuliidae (Diptera) of Finland. In: Kahanpää J, Salmela J (Eds) Checklist of the Diptera of Finland. ZooKeys @@: @-@. doi: 10.3897/zookeys.??.7600*

SIMULIINAE Newman, 1834
tribe Prosimuliini Enderlein, 1921
HELODON Enderlein, 1921
Helodon ferrugineus (Wahlberg, 1844) [Kuusela 1971, Hackman 1980]
PROSIMULIUM Roubaud, 1906
Prosimulium hirtipes (Fries, 1824) [Kuusela 1971, Hackman 1980]
Prosimulium luganicum Rubtsov, 1956 [Crosskey 2004, Ilmonen and Kuusela 2006, Hackman 1980]
Prosimulium macropyga (Lundström, 1911) [Kuusela 1971, Hackman 1980]
Prosimulium ursinum (Edwards, 1935) [Kuusela 1971, Hackman 1980]
tribe Simuliini Newman, 1834
CNEPHIA Enderlein, 1921
Cnephia eremites Shewell, 1952 [Ilmonen and Kuusela 2006]
Cnephia pallipes (Fries, 1824) [Hackman 1980]
= *C. lapponica* (Enderlein, 1921) [Hackman 1980]
GRENIERA Doby & David, 1959
Greniera ivanovae Ivashchenko, 1970 [Ilmonen and Kuusela 2006]
METACNEPHIA Crosskey, 1969
Metacnephia bilineata (Rubtsov, 1940)
= *M. saileri* (Stone, 1952) [Jensen 1997, Ilmonen and Kuusela 2006]
Metacnephia lyra (Lundström, 1911) [Kuusela 1971, Hackman 1980]
= *M. tabescentifrons* (Enderlein, 1929) [Kuusela 1981a]
= *M. trigoniformis* Yankovsky, 2003 [Crosskey 2004, Ilmonen and Kuusela 2006]
Metacnephia tredecimata (Edwards, 1920) [Jensen 1997, Ilmonen and Kuusela 2006]
SIMULIUM Latreille, 1802
sg. ***Boophthora*** Enderlein, 1921
Simulium erythrocephalum (De Geer, 1776) [Hackman 1980]
sg. ***Eusimulium*** Roubaud, 1906
Simulium angustipes Edwards, 1915 [Jensen 1997, Ilmonen and Kuusela 2006]
= *S. securiforme* (Rubtsov, 1956) [Kuusela 1981b]
Simulium aureum Fries, 1824 [Hackman 1980]
Simulium velutinum (Santos Abreu, 1922) [Ilmonen and Adler 2008]
sg. ***Boreosimulium*** Rubzov & Yankovsky, 1982
Simulium annulus (Lundström, 1911) [Kuusela 1971, Hackman 1980]
Simulium baffinense Twinn, 1936 [Ilmonen and Kuusela 2003]
Simulium crassum (Rubtsov, 1956) [Ilmonen and Kuusela 2003]
sg. ***Hellichiella*** Rivosecchi & Cardinali, 1975
Simulium dogieli (Rubtsov, 1956) [Kuusela 1971, Hackman 1980]
Simulium latipes (Meigen, 1804)
Simulium usovae (Golini, 1987) [Ilmonen and Adler 2008]
sg. ***Nevermannia*** Enderlein, 1921
= ***Cnetha*** Enderlein, 1921
Simulium angustitarse (Lundström, 1911) [Hackman 1980]
Simulium beltukovae (Rubtsov, 1956) [Kuusela 1981b]

= *S. carpathicum* (Knoz, 1961) [Ilmonen and Kuusela 2006]
Simulium bicorne Dorogostaisky, Rubtsov & Vlasenko, 1935 [Kuusela 1992]
Simulium cryophilum (Rubtsov, 1959) [Kuusela 1992]
= *S. carthusiense* f. brevicaulis Dorier & Grenier, 1961 [Kuusela and Wotton 1978, Hackman 1980]
Simulium curvans (Rubtsov & Carlsson, 1965) [Crosskey 2004, Ilmonen and Kuusela 2006]
Simulium dendrofilum (Patrusheva, 1962) [Ilmonen and Adler 2008]
Simulium fontinale Radzivilovskaya, 1948 [Kuusela 1971, Hackman 1980]
Simulium juxtacrenobium Bass & Brockhouse, 1990 [Ilmonen and Adler 2008, Ilmonen et al. 2009]
Simulium lundstromi (Enderlein, 1921) [Kuusela 1992]
Simulium silvestre (Rubtsov, 1956) [Crosskey 2004, Ilmonen and Kuusela 2006]
Simulium vernum Macquart, 1826 [Jensen 1997, Ilmonen and Kuusela 2006]
= *S. pritzkowi* (Enderlein, 1926) [Kuusela 1971, Hackman 1980]
sg. ***Schoenbaueria*** Enderlein, 1921
Simulium pusillum Fries, 1824 [Kuusela 1971, Hackman 1980]
Simulium subpusillum Rubtsov, 1940 [Jensen 1997, Ilmonen and Kuusela 2006]
sg. ***Simulium*** Latreille, 1802
Simulium annulitarse Zetterstedt, 1838 [Crosskey 2004, Ilmonen and Kuusela 2006]
Simulium argyreatum Meigen, 1838 [Crosskey 2004, Ilmonen and Kuusela 2006]
Simulium frigidum Rubtsov, 1940 [Kuusela 1971, Hackman 1980]
Simulium intermedium Roubaud, 1906 [Ilmonen and Adler 2008]
Simulium longipalpe Beltyukova, 1955
= *S. curvistylus* Rubtsov, 1957 [Jensen 1997, Ilmonen and Kuusela 2006]
Simulium monticola Friederichs, 1920 [Kuusela 1992]
Simulium morsitans Edwards, 1915 [Kuusela 1971, Hackman 1980]
Simulium murmanum Enderlein, 1935 [Crosskey 2004, Ilmonen and Kuusela 2006]
= *S. forsi* (Carlsson, 1962) [Kuusela 1971, Hackman 1980]
Simulium noelleri Friederichs, 1920 [Kuusela 1992]
Simulium ornatum Meigen, 1818 [Kuusela 1971, Hackman 1980]
Simulium paramorsitans Rubtsov, 1956 [Ilmonen and Kuusela 2006]
Simulium posticatum Meigen, 1838 [Jensen 1997, Ilmonen and Kuusela 2006]
= *S. austeni* Edwards, 1915 [Kuusela 1979, Hackman 1980]
= *S. verecudum* misid. [Kuusela 1971, Hackman 1980]
Simulium reptans (Linnaeus, 1758) [Hackman 1980]
Simulium rostratum (Lundström, 1911) [Jensen 1997, Ilmonen and Kuusela 2006]
= *S. sublacustre* Davies, 1966 [Kuusela 1992]
Simulium rubtzovi Smart, 1945 [Ilmonen and Adler 2008]
Simulium transiens Rubtsov, 1940 [Kuusela 1971, Hackman 1980]
Simulium truncatum (Lundström, 1911) [Hackman 1980]
Simulium tuberosum (Lundström, 1911) [Hackman 1980]
Simulium tumulosum Rubtsov, 1956 [Kuusela and Adler 1994]
Simulium vulgare Dorogostaisky, Rubtsov & Vlasenko, 1935 [Hackman 1980, Kuusela and Adler 1994]
sg. ***Wilhelmia*** Enderlein, 1921
Simulium equinum (Linnaeus, 1758) [Jensen 1997, Ilmonen and Kuusela 2006]
= *S. fuscipes* (Fries, 1824) [Hackman 1980]
= *S. zetlandense* (Davies, 1966) [Kuusela and Wotton 1978, Hackman 1980]
STEGOPTERNA Enderlein, 1930

Stegopterna trigonium (Lundström, 1911) [Hackman 1980]
= *S. richteri* (Enderlein, 1930) [Kuusela 1971]

Excluded species

Simulium lineatum (Meigen, 1804) [Hackman 1980]
Simulium meigeni Rubzov & Carlsson, 1965 [Hackman 1980]
= *S. pygmaeum* of authors[Kuusela 1971]
Simulium tsheburovae (Rubtsov, 1956) [Crosskey 2004, Ilmonen and Kuusela 2006]

CHAOBORIDAE Newman, 1834

Supporting references for *Salmela J, Paasivirta L, Kvifte GM (2014) Checklist of the familes Chaoboridae, Dixidae, Thaumaleidae Psychodidae and Ptychopteridae (Diptera) of Finland. In: Kahanpää J, Salmela J (Eds) Checklist of the Diptera of Finland. ZooKeys @@: @-@. doi: 10.3897/zookeys.??.7532*

CHAOBORUS Lichtenstein, 1800
sg. ***Chaoborus*** Lichtenstein, 1800
Chaoborus crystallinus (De Geer, 1776) [Hackman 1980]
Chaoborus flavicans (Meigen, 1830) [Hirvenoja 1961, Hackman 1980]
Chaoborus obscuripes (van der Wulp, 1859) [Hackman 1980]
sg. ***Peusomyia*** Sæther, 1970
Chaoborus pallidus (Fabricius, 1794) [Hackman 1980]
sg. ***Schadanophasma*** Dyar & Shannon, 1924
Chaoborus nyblaei (Zetterstedt, 1838) [Hirvenoja 1961, Hackman 1980]
CRYOPHILA Edwards, 1930
Cryophila lapponica (Martini, 1928) [Hackman 1980]
MOCHLONYX Loew, 1844
Mochlonyx fuliginosus Felt, 1905 [Hackman 1980]
= *M. martinii* Edwards, 1930
= *M. velutinus* of authors
Mochlonyx velutinus (Ruthe, 1831)
= *M. culiciformis* (De Geer, 1776) preocc.[Hackman 1980]

DIXIDAE Schiner, 1868

Supporting references for *Salmela J, Paasivirta L, Kvifte GM (2014) Checklist of the familes Chaoboridae, Dixidae, Thaumaleidae Psychodidae and Ptychopteridae (Diptera) of Finland. In: Kahanpää J, Salmela J (Eds) Checklist of the Diptera of Finland. ZooKeys @@: @-@. doi: 10.3897/zookeys.??.7532*

DIXA Meigen, 1818
Dixa dilatata Strobl, 1900 [Salmela 2003a]
Dixa nebulosa Meigen, 1830 [Hackman 1980]
Dixa puberula Loew, 1849
Dixa submaculata Edwards, 1920 [Salmela 2003a]
DIXELLA Dyar & Shannon, 1924
Dixella aestivalis (Meigen, 1818) [Hackman 1980]
Dixella amphibia (De Geer, 1776) [Hackman 1980]
Dixella autumnalis (Meigen, 1838)
Dixella borealis (Martini, 1928) [Hackman 1980]
Dixella dyari (Garret, 1924)
Dixella filicornis (Edwards, 1926) [Salmela 2003a]
Dixella hyperborea (Bergroth, 1889) [Hackman 1980]
Dixella laeta (Loew, 1849) [Salmela 2008]
Dixella naevia (Peus, 1934) [Hellén 1952]
Dixella nigra (Staeger, 1840) [Salmela et al. 2007a]
Dixella obscura (Loew, 1849) [Hackman 1980]
Dixella serotina (Meigen, 1818) [Hackman 1980]

THAUMALEIDAE Bezzi, 1913

Supporting references for *Salmela J, Paasivirta L, Kvifte GM (2014) Checklist of the familes Chaoboridae, Dixidae, Thaumaleidae Psychodidae and Ptychopteridae (Diptera) of Finland. In: Kahanpää J, Salmela J (Eds) Checklist of the Diptera of Finland. ZooKeys @@: @-@. doi: 10.3897/zookeys.??.7532*

THAUMALEA Ruthe, 1831
Thaumalea truncata Edwards, 1929 [Salmela 2003b,a]

CULICIDAE Meigen, 1818

Supporting references for *Huldén L, Huldén L (2014) Checklist of the Culicidae (Diptera) in Finland. In: Kahanpää J, Salmela J (Eds) Checklist of the Diptera of Finland. ZooKeys @@: @-@. doi:*

ANOPHELINAE Grassi, 1900
ANOPHELES Meigen, 1818
sg. ***Anopheles*** Meigen, 1818
Anopheles beklemishevi Stegni & Kabanova, 1976 [Utrio 1979, Hackman 1980]
= *A. maculipennis* misid.in part
Anopheles claviger (Meigen, 1804) [Utrio 1975, Hackman 1980]
Anopheles messeae Falleroni, 1926 [Ulmanen and Brummer-Korvenkontio 1971, Hackman 1980]
= *A. claviger* auct. nec (Meigen, 1804)
= *A. maculipennis* misid.in part
CULICINAE Meigen, 1818
AEDES Meigen, 1818
sg. ***Aedes*** Meigen, 1818
Aedes cinereus Meigen, 1818 [Hackman 1980]
sg. ***Aedimorphus*** Theobald, 1903
Aedes vexans (Meigen, 1830) [Hackman 1980]
COQUILLETTIDIA Dyar, 1905
sg. ***Coquillettidia*** Dyar, 1905
Coquillettidia richiardii (Ficalbi, 1889) [Hackman 1980]
CULEX Linnaeus, 1758
sg. ***Culex*** Linnaeus, 1758
Culex pipiens Linnaeus, 1758 [Hackman 1980]
Culex torrentium Martini, 1925 [Hackman 1980]
sg. ***Neoculex*** Dyar, 1905
Culex territans Walker, 1856 [Hackman 1980]
= *C. apicalis* auct. nec Adams, 1903
CULISETA Felt, 1904
THEOBALDIA Neveu-Lemaire, 1902 preocc.
sg. ***Culicella*** Felt, 1904
Culiseta morsitans (Theobald, 1901) [Hackman 1980]
Culiseta ochroptera Peus, 1935 [Utrio 1976, Hackman 1980]
sg. ***Culiseta*** Felt, 1904
Culiseta alaskaensis (Ludlow, 1906) [Hackman 1980]
Culiseta annulata (Schrank, 1776) [Kangas 1952, Hackman 1980]
Culiseta bergrothi (Edwards, 1921) [Hackman 1980]
= *C. glaphyroptera* auct. nec (Shiner, 1864)
Culiseta subochrea (Edwards, 1921) [Hackman 1980]
DAHLIANA Reinert, Harbach & Kitching, 2006
Dahliana geniculata (Olivier, 1791) [Itämies 1981]
OCHLEROTATUS Lynch Arribalzaga, 1881
sg. ***Ochlerotatus*** Lynch Arribalzaga, 1881
Ochlerotatus annulipes (Meigen, 1830) [Utrio 1977, Hackman 1980]
Ochlerotatus cantans (Meigen, 1818) [Hackman 1980]
= *O. waterhousei* (Theobald in Waterhouse, 1905)

= *O. maculatus* auct. nec (Meigen, 1804)
= *O. rusticus* auct. nec (Rossi,1790)
Ochlerotatus caspius (Pallas, 1771) [Hackman 1980]
Ochlerotatus cataphylla Dyar, 1916 [Hackman 1980]
Ochlerotatus communis (De Geer, 1776) [Hackman 1980]
= *O. nemorosus* (Meigen, 1818)
Ochlerotatus cyprius Ludlow, 1920 [Hackman 1980]
= *O. freyi* Edwards, 1921
Ochlerotatus diantaeus Howard, Dyar & Knab, 1912 [Hackman 1980]
Ochlerotatus dorsalis (Meigen, 1830) [Hackman 1980]
Ochlerotatus euedes (Howard, Dyar & Knab, 1913)
= *O. beklemishevi* Denisova, 1955 [Utrio 1975, Hackman 1980]
Ochlerotatus excrucians (Walker, 1848) [Hackman 1980]
Ochlerotatus flavescens (Müller, 1776) [Hackman 1980]
= *O. lutescens* (Fabricius, 1775)
= *O. variegatus* (Schrank, 1781)
Ochlerotatus hexodontus Dyar, 1919 [Brummer-Korvenkontio et al. 1971, Hackman 1980]
Ochlerotatus impiger (Walker, 1848) [Hackman 1980]
= *O. parvulus* (Edwards 1921)
= *O. nearcticus* auct. nec (Dyar, 1922)
Ochlerotatus intrudens Dyar, 1919 [Hackman 1980]
Ochlerotatus leucomelas (Meigen, 1804) [Hackman 1980]
Ochlerotatus nigrinus (Eckstein, 1918) [Hackman 1980]
= *O. sticticus* auct. nec Meigen, 1828 [Frey 1932a, Utrio 1979]
Ochlerotatus nigripes (Zetterstedt, 1838) [Brummer-Korvenkontio et al. 1971, Hackman 1980]
= *O. alpinus* auct. nec (Linnaeus, 1792) (nom. dubium)
Ochlerotatus pionips Dyar, 1919 [Utrio 1975, Hackman 1980]
Ochlerotatus pullatus (Coquillett, 1904) [Hackman 1980]
Ochlerotatus punctodes Dyar, 1922 [Utrio 1976, Hackman 1980]
Ochlerotatus punctor (Kirby, 1837) [Hackman 1980]
Ochlerotatus riparius Dyar & Knab, 1907 [Hirvenoja 1962b, Hackman 1980]

Excluded species

Ochlerotatus behningi Martini, 1926
Ochlerotatus rusticus (Rossi, 1790) [Utrio 1979]

PTYCHOPTERIDAE Osten Sacken, 1862

Supporting references for *Salmela J, Paasivirta L, Kvifte GM (2014) Checklist of the familes Chaoboridae, Dixidae, Thaumaleidae Psychodidae and Ptychopteridae (Diptera) of Finland. In: Kahanpää J, Salmela J (Eds) Checklist of the Diptera of Finland. ZooKeys @@: @-@. doi: 10.3897/zookeys.??.7532*

PTYCHOPTERA Meigen, 1803
sg. ***Paraptychoptera*** Tonnoir, 1919
Ptychoptera lacustris Meigen, 1830 [Hackman 1980]
Ptychoptera paludosa Meigen, 1804 [Hackman 1980]
sg. ***Ptychoptera*** Meigen, 1803
Ptychoptera albimana (Fabricius, 1787) [Hackman 1980]
Ptychoptera contaminata (Linnaeus, 1758) [Hackman 1980]
Ptychoptera hugoi Tjeder, 1968 [Salmela 2001b]
Ptychoptera minuta Tonnoir, 1919 [Hackman 1980]
Ptychoptera scutellaris Meigen, 1818 [Hackman 1980]

BIBIONIDAE Fleming, 1821

Supporting references for *Haarto A (2014) Checklist of the families Anisopodidae, Bibionidae, Canthyloscelididae, Mycetobiidae, Pachyneuridae and Scatopsidae (Diptera) of Finland. In: Kahanpää J, Salmela J (Eds) Checklist of the Diptera of Finland. ZooKeys @@: @-@. doi: 10.3897/zookeys.??.7358*

BIBIONINAE Fleming, 1821
BIBIO Geoffroy, 1762
Bibio brunnipes (Fabricius, 1794)
= *B. fulvipes* (Zetterstedt, 1838) [Hackman 1980]
Bibio clavipes Meigen, 1818 [Hackman 1980]
Bibio ferruginatus (Linnaeus, 1767) [Hackman 1980]
Bibio fulvicollis Gimmerthal, 1842 [Hackman 1980]
= *B. festinans* (Zetterstedt, 1850)
Bibio johannis (Linnaeus, 1767) [Hackman 1980]
Bibio lautaretensis Villeneuve, 1925
= *B. crassipes* Duda, 1930 [Hackman 1980]
Bibio longipes Loew, 1864 [Haarto 2012]
= *B. lepidus* Loew, 1871 [Skartveit 1999]
Bibio marci (Linnaeus, 1758) [Hackman 1980]
Bibio nigriventris Haliday, 1833 [Hackman 1980]
Bibio pomonae (Fabricius, 1775) [Hackman 1980]
Bibio rufipes (Zetterstedt, 1838) [Hackman 1980]
Bibio siebkei Mik, 1887
= *B. femoralis* (Siebke, 1863) preocc.[Hackman 1980]
Bibio varipes Meigen, 1830 [Skartveit 1999]
DILOPHUS Meigen, 1803
Dilophus borealis Skartveit, 1993 [Skartveit 1999]
Dilophus febrilis (Linnaeus, 1758) [Haarto and Winqvist 2014]
Dilophus femoratus Meigen, 1804 [Hackman 1980]
PLECIINAE Fleming, 1821
PENTHETRIA Meigen, 1803
Penthetria funebris Meigen, 1804
= *P. holosericea* Meigen, 1818 [Hackman 1980]

ANISOPODIDAE Knab, 1912

Supporting references for *Haarto A (2014) Checklist of the families Anisopodidae, Bibionidae, Canthyloscelididae, Mycetobiidae, Pachyneuridae and Scatopsidae (Diptera) of Finland. In: Kahanpää J, Salmela J (Eds) Checklist of the Diptera of Finland. ZooKeys @@: @-@. doi: 10.3897/zookeys.??.7358*

SYLVICOLA Harris, 1780
= ***Phryne*** Meigen, 1800 suppr.
= ***Rhyphus*** Latreille, 1804
Sylvicola cinctus (Fabricius, 1787) [Mannheims 1965, Hackman 1980]
Sylvicola fenestralis (Scopoli, 1763) [Hackman 1980]
Sylvicola fuscatoides Michelsen, 1999 [Haarto 2011]
= *S. fuscatus* sensu Andersson 1967 (nec Fabricius, 1775) [Hackman 1980]
Sylvicola fuscatus (Fabricius, 1775) [Haarto 2011]
Sylvicola punctatus (Fabricius, 1787) [Hackman 1980]
Sylvicola stackelbergi Krivosheina & Menzel, 1998 [Krivosheina and Menzel 2002]
Sylvicola zetterstedti (Edwards, 1923) [Hackman 1980]

SCATOPSIDAE Newman, 1834

Supporting references for *Haarto A (2014) Checklist of the families Anisopodidae, Bibionidae, Canthyloscelididae, Mycetobiidae, Pachyneuridae and Scatopsidae (Diptera) of Finland. In: Kahanpää J, Salmela J (Eds) Checklist of the Diptera of Finland. ZooKeys @@: @-@. doi: 10.3897/zookeys.??.7358*

ASPISTINAE Rondani, 1840
ARTHRIA Kirby in Richardson, 1837
Arthria analis (Kirby in Richardson, 1837) [Frey et al. 1941]
ASPISTES Meigen, 1818
Aspistes berolinensis Meigen, 1818 [Hackman 1980]
Aspistes freyi Cook, 1965 [Cook 1965, Hackman 1980]
Aspistes helleni Krivosheina, 2000 [Haarto and Winqvist 2014]
ECTAETIINAE Enderlein, 1936
ECTAETIA Enderlein, 1912
Ectaetia clavipes (Loew, 1846) [Krivosheina 2002]
Ectaetia platyscelis (Loew, 1869) [Hackman 1980]
PSECTROSCIARINAE Cook, 1963
ANAPAUSIS Enderlein, 1912
Anapausis floricola Chandler, 1999 [Haarto and Winqvist 2014]
= *A. soluta* misid.[Hackman 1980, Haarto and Winqvist 2014]
Anapausis rectinervis Duda, 1928 [Hackman 1980]
SCATOPSINAE Newman, 1834
tribe Colobostematini Amorim, 1994
COLOBOSTEMA Enderlein, 1926
Colobostema infumatum (Haliday, 1833) [Krivosheina 2000b]
Colobostema nigripenne (Meigen, 1830) [Hackman 1980]
Colobostema triste (Zetterstedt, 1850) [Hackman 1980]
EFCOOKELLA Haenni, 1998
Efcookella albitarsis (Zetterstedt, 1850) [Hackman 1980]
FERNEIELLA Cook in Freeman, 1985
Ferneiella incompleta (Verrall, 1886) [Hackman 1980]
HOLOPLAGIA Enderlein, 1912
Holoplagia bullata (Edwards, 1925) [Hackman 1980]
Holoplagia transversalis (Loew, 1846) [Hackman 1980]
tribe Rhegmoclematini Cook, 1955
RHEGMOCLEMINA Enderlein, 1936
Rhegmoclemina vaginata (Lundström, 1910) [Hackman 1980]
THRIPOMORPHA Enderlein, 1905
= ***Rhegmoclema*** Enderlein, 1912
Thripomorpha freyi (Cook, 1969) [Cook 1969, Hackman 1980]
Thripomorpha halteratum (Meigen, 1838) [Hackman 1980]
Thripomorpha paludicola Enderlein, 1905 [Krivosheina 2001]
= *T. edwardsi* (Collin, 1954) [Cook 1969, Hackman 1980]
Thripomorpha verralli (Edwards, 1934) [Cook 1969, Hackman 1980]
tribe Scatopsini Newman, 1834
APILOSCATOPSE Cook, 1974
Apiloscatopse flavicollis (Meigen, 1818) [Hackman 1980]
Apiloscatopse flavocincta (Duda, 1928) [Hackman 1980]

REICHERTELLA Enderlein, 1912
Reichertella geniculata (Zetterstedt, 1850) [Hackman 1980]
SCATOPSE Geoffroy, 1762
Scatopse lapponica Duda, 1928 [Hackman 1980]
Scatopse notata (Linnaeus, 1758) [Hackman 1980]
tribe Swammerdamellini Cook, 1972
COBOLDIA Melander, 1916
Coboldia fuscipes (Meigen, 1830) [Hackman 1980]
RHEXOZA Enderlein, 1936
Rhexoza subnitens (Verrall, 1886) [Hackman 1980]
SWAMMERDAMELLA Enderlein, 1912
Swammerdamella adercotris Cook, 1972 [Krivosheina 2001]
Swammerdamella brevicornis (Meigen, 1830) [Hackman 1980]
Swammerdamella genypodis Cook, 1972 [Cook 1972, Hackman 1980]

Excluded species

Swammerdamella acuta Cook, 1956 [Cook 1972, Hackman 1980, Haarto and Winqvist 2014]

CANTHYLOSCELIDIDAE Enderlein, 1912

Supporting references for *Haarto A (2014) Checklist of the families Anisopodidae, Bibionidae, Canthyloscelididae, Mycetobiidae, Pachyneuridae and Scatopsidae (Diptera) of Finland. In: Kahanpää J, Salmela J (Eds) Checklist of the Diptera of Finland. ZooKeys @@: @-@. doi: 10.3897/zookeys.??.7358*

CANTHYLOSCELIDINAE Enderlein, 1912
HYPEROSCELIS Hardy & Nagatomi, 1960
Hyperoscelis eximia (Boheman, 1858) [Hackman 1980]
Hyperoscelis veternosa Mamaev & Krivosheina, 1969 [Hackman 1980]
SYNNEURINAE Enderlein, 1936
SYNNEURON Lundström, 1910
Synneuron annulipes Lundström, 1910 [Hackman 1980]

MYCETOBIIDAE Winnertz, 1863

Supporting references for *Haarto A (2014) Checklist of the families Anisopodidae, Bibionidae, Canthyloscelididae, Mycetobiidae, Pachyneuridae and Scatopsidae (Diptera) of Finland. ZooKeys XX:XX-XX*

MYCETOBIA Meigen, 1818
Mycetobia pallipes Meigen, 1818 [Hackman 1980]

PACHYNEURIDAE Schiner, 1864

Supporting references for *Haarto A (2014) Checklist of the families Anisopodidae, Bibionidae, Canthyloscelididae, Mycetobiidae, Pachyneuridae and Scatopsidae (Diptera) of Finland. In: Kahanpää J, Salmela J (Eds) Checklist of the Diptera of Finland. ZooKeys @@: @-@. doi: 10.3897/zookeys.??.7358*

PACHYNEURA Zetterstedt, 1838
Pachyneura fasciata Zetterstedt, 1838 [Hackman 1980, Väisänen and Biström 1991]

DITOMYIIDAE Keilin, 1919

Supporting references for *Jakovlev J (2014) Checklist of the fungus gnats of Finland: Bolitophilidae, Diadocidiidae, Ditomyiidae, Keroplatidae and Mycetophilidae (Diptera). In: Kahanpää J, Salmela J (Eds) Checklist of the Diptera of Finland. ZooKeys @@: @-@. doi: 10.3897/zookeys.??.7646*

SYMMERUS Walker, 1848
Symmerus annulatus (Meigen, 1830) [Hackman 1980]
Symmerus nobilis Lackschewitz, 1937 [Salmela et al. 2014]

CECIDOMYIIDAE Billberg, 1820

Supporting references for *Jaschhof M, Skuhravá M, Penttinen J (2014) Checklist of the Cecidomyiidae (Diptera) of Finland. In: Kahanpää J, Salmela J (Eds) Checklist of the Diptera of Finland. ZooKeys XX: X-X. doi: 10.3897/zookeys.??.7503*

LESTREMIINAE Rondani, 1840
tribe Lestremiini Rondani, 1840
ALLARETE Pritchard, 1951
Allarete nigra Mamaev, 1994 [Jaschhof and Jaschhof 2009]
ANARETE Haliday, 1833
Anarete sp. 2 [Jaschhof and Jaschhof 2009]
ANARETELLA Enderlein, 1911
Anaretella defecta (Winnertz, 1870) [Ø kland and Mamaev 1997, Jaschhof and Jaschhof 2009]
= *A. magnicornis* Mamaev, 1964 [Ø kland and Mamaev 1997]
Anaretella iola Pritchard, 1951 [Jaschhof and Jaschhof 2009]
LESTREMIA Macquart, 1826
Lestremia cinerea Macquart, 1826 [Jaschhof and Jaschhof 2009]
Lestremia leucophaea (Meigen, 1818) [Hackman 1980, Jaschhof and Jaschhof 2009]
MICROMYINAE Rondani, 1856
tribe Acoenoniini Pritchard, 1960
ACOENONIA Pritchard, 1947
Acoenonia europaea Mamaev, 1964 []
tribe Aprionini Jaschhof, 1998
APRIONUS Kieffer, 1894
Aprionus accipitris Jaschhof, 1997 [Jaschhof and Jaschhof 2009]
Aprionus acutus Edwards, 1938 [Jaschhof and Jaschhof 2009]
Aprionus adventitius Jaschhof, 2009 [Jaschhof and Jaschhof 2009]
Aprionus aquilonius Jaschhof, 2009 [Jaschhof and Jaschhof 2009]
Aprionus arcticus Mamaev, 2001 [Jaschhof and Jaschhof 2009]
Aprionus aviarius Mamaev & Berest, 1990 [Jaschhof and Jaschhof 2009]
Aprionus betulae Jaschhof, 1996 [Jaschhof and Jaschhof 2009]
Aprionus bidentatus (Kieffer, 1894) [Jaschhof and Jaschhof 2009]
Aprionus bifidus Mamaev, 1963 [Jaschhof and Jaschhof 2009]
Aprionus bispinosus Edwards, 1938 [Jaschhof and Jaschhof 2009]
Aprionus brachypterus Edwards, 1938 [Jaschhof and Jaschhof 2009]
Aprionus brevitegminis Jaschhof, 2009 [Jaschhof and Jaschhof 2009]
Aprionus cardiophorus Mamaev, 1963 [Jaschhof and Jaschhof 2009]
Aprionus carinatus Jaschhof, 1996 [Jaschhof and Jaschhof 2009]
Aprionus complicatus Mamaev & Berest, 1986 [Jaschhof and Jaschhof 2009]
Aprionus confusus Mamaev, 1969 [Jaschhof and Jaschhof 2009]
Aprionus corniculatus Mamaev, 1963 [Jaschhof and Jaschhof 2009]
Aprionus cornutus Berest, 1986 [Jaschhof and Jaschhof 2009]
Aprionus dalarnensis Mamaev, 1998 [Jaschhof and Jaschhof 2009]
Aprionus dentifer Mamaev, 1965 [Jaschhof and Jaschhof 2009]
Aprionus dispar Mamaev, 1963 [Jaschhof and Jaschhof 2009]
Aprionus dissectus Mamaev & Berest, 1990 [Jaschhof and Jaschhof 2009]
Aprionus duplicatus Mamaev, 1998 [Jaschhof and Jaschhof 2009]

Aprionus ensiferus Jaschhof, 1996 [Jaschhof and Jaschhof 2009]
Aprionus fennicus Jaschhof, 2009 [Jaschhof and Jaschhof 2009]
Aprionus flavidus (Winnertz, 1870) [Jaschhof and Jaschhof 2009]
Aprionus foliosus Jaschhof, 2009 [Jaschhof and Jaschhof 2009]
Aprionus gladiator Jaschhof, 2009 [Jaschhof and Jaschhof 2009]
Aprionus halteratus (Zetterstedt, 1852) [Jaschhof and Jaschhof 2009]
Aprionus heothinos Jaschhof, 2009 [Jaschhof and Jaschhof 2009]
Aprionus hintelmannorum Jaschhof, 2009 [Jaschhof and Jaschhof 2009]
Aprionus inquisitor Mamaev, 1963 [Jaschhof and Jaschhof 2009]
Aprionus insignis Mamaev, 1963 [Jaschhof and Jaschhof 2009]
Aprionus laevis Mohrig, 1967 [Jaschhof and Jaschhof 2009]
Aprionus lapponicus Jaschhof & Mamaev, 1997 [Jaschhof and Jaschhof 2009]
Aprionus laricis Mamaev & Jaschhof, 1997 [Jaschhof and Jaschhof 2009]
Aprionus latitegminis Jaschhof, 2009 [Jaschhof and Jaschhof 2009]
Aprionus longicollis Mamaev, 1963 [Jaschhof and Jaschhof 2009]
Aprionus longitegminis Yukawa, 1967 [Jaschhof and Jaschhof 2009]
Aprionus marginatus Mamaev, 1963 [Jaschhof and Jaschhof 2009]
Aprionus miki Kieffer, 1895 [Jaschhof and Jaschhof 2009]
Aprionus oligodactylus Jaschhof, 2009 [Jaschhof and Jaschhof 2009]
Aprionus piceae Jaschhof, 1997 [Jaschhof and Jaschhof 2009]
Aprionus praecipuus Jaschhof, 2009 [Jaschhof and Jaschhof 2009]
Aprionus pyxidiifer Mamaev, 1998 [Jaschhof and Jaschhof 2009]
Aprionus reduncus Jaschhof, 2009 [Jaschhof and Jaschhof 2009]
Aprionus separatus Mamaev & Jaschhof, 1997 [Jaschhof and Jaschhof 2009]
Aprionus sievertorum Jaschhof, 2009 [Jaschhof and Jaschhof 2009]
Aprionus similis Mamaev, 1963 [Jaschhof and Jaschhof 2009]
Aprionus spiniferus Mamaev & Berest, 1990 [Jaschhof and Jaschhof 2009]
Aprionus spiniger (Kieffer, 1894) [Jaschhof and Jaschhof 2009]
Aprionus stiktos Jaschhof, 2009 [Jaschhof and Jaschhof 2009]
Aprionus stylifer Mamaev, 1998 [Jaschhof and Jaschhof 2009]
Aprionus styloideus Mamaev & Berest, 1990 [Jaschhof and Jaschhof 2009]
Aprionus subacutus Jaschhof, 1997 [Jaschhof and Jaschhof 2009]
Aprionus svecicus Jaschhof, 1996 [Jaschhof and Jaschhof 2009]
Aprionus taigaensis Jaschhof, 2009 [Jaschhof and Jaschhof 2009]
Aprionus tiliamcorticis Mamaev, 1963 [Jaschhof and Jaschhof 2009]
Aprionus victoriae Jaschhof, 2009 [Jaschhof and Jaschhof 2009]
tribe Bryomyiini Berest, 1993
BRYOMYIA Kieffer, 1895
= ***Tomonomyia*** Berest, 1993
Bryomyia apsectra Edwards, 1938 [Ø kland and Mamaev 1997, Jaschhof and Jaschhof 2009]
Bryomyia bergrothi Kieffer, 1895 [Jaschhof and Jaschhof 2009]
Bryomyia gibbosa (Felt, 1907) [Jaschhof and Jaschhof 2009]
Bryomyia producta (Felt, 1908) [Jaschhof and Jaschhof 2009]
HETEROGENELLA Mamaev, 1963
= ***Cervuatina*** Berest, 1993
Heterogenella finitima Mamaev, 1998 [Jaschhof and Jaschhof 2009]
Heterogenella hybrida Mamaev, 1963 [Jaschhof and Jaschhof 2009]
Heterogenella linearis Yukawa, 1971 [Jaschhof and Jaschhof 2009]
SKUHRAVIANA Mamaev, 1963

Skuhraviana triangulifera Mamaev, 1963 [Jaschhof and Jaschhof 2009]
tribe Campylomyzini Kieffer, 1898
CAMPYLOMYZA Meigen, 1818
Campylomyza abbreviata Jaschhof, 2009 [Jaschhof and Jaschhof 2009]
Campylomyza aemula Mamaev, 1998 [Jaschhof and Jaschhof 2009]
Campylomyza alpina Siebke, 1863 [Jaschhof and Jaschhof 2009]
Campylomyza arcuata Jaschhof, 2009 [Jaschhof and Jaschhof 2009]
Campylomyza armata Mamaev, 1963 [Jaschhof and Jaschhof 2009]
Campylomyza cavitata Mamaev, 1998 [Jaschhof and Jaschhof 2009]
Campylomyza cingulata Jaschhof, 2009 [Jaschhof and Jaschhof 2009]
Campylomyza dilatata Felt, 1907 [Jaschhof and Jaschhof 2009]
Campylomyza falcifera Jaschhof, 2009 [Jaschhof and Jaschhof 2009]
Campylomyza flavipes Meigen, 1818 [Jaschhof and Jaschhof 2009]
= *C. pallipes* Zetterstedt, 1850 [Hackman 1980]
Campylomyza furva Edwards, 1938 [Jaschhof and Jaschhof 2009]
Campylomyza fusca Winnertz, 1870 [Jaschhof and Jaschhof 2009]
Campylomyza inornata Jaschhof, 2009 [Jaschhof and Jaschhof 2009]
Campylomyza insolita Jaschhof, 2009 [Jaschhof and Jaschhof 2009]
Campylomyza ormerodi (Kieffer, 1913) [Jaschhof and Jaschhof 2009]
Campylomyza serrata Jaschhof, 1998 [Jaschhof and Jaschhof 2009]
Campylomyza spatulata Mamaev, 1998 [Jaschhof and Jaschhof 2009]
Campylomyza stegetfore Jaschhof, 2009 [Jaschhof and Jaschhof 2009]
EXCRESCENTIA Mamaev & Berest, 1991
Excrescentia mutuata Mamaev & Berest, 1991 [Jaschhof and Jaschhof 2009]
NEUROLYGA Rondani, 1840
= ***Cordylomyia*** Felt, 1911
Neurolyga acuminata Jaschhof, 2009 [Jaschhof and Jaschhof 2009]
Neurolyga bilobata (Mamaev & Rozhnova, 1982) [Jaschhof and Jaschhof 2009]
Neurolyga excavata (Yukawa, 1967) [Jaschhof and Jaschhof 2009]
Neurolyga interrupta Jaschhof, 2009 [Jaschhof and Jaschhof 2009]
Neurolyga lonsdalensis Jaschhof, 2009 [Jaschhof and Jaschhof 2009]
Neurolyga paludosa Jaschhof, 2009 [Jaschhof and Jaschhof 2009]
Neurolyga sylvestris (Felt, 1907) [Jaschhof and Jaschhof 2009]
Neurolyga verna (Mamaev, 1963) [Jaschhof and Jaschhof 2009]
tribe Catochini Edwards, 1938
CATOCHA Haliday, 1833
Catocha incisa Jaschhof, 2009 [Jaschhof and Jaschhof 2009]
Catocha latipes Haliday, 1833 [Jaschhof and Jaschhof 2009]
tribe Micromyini Rondani, 1856
MICROMYA Rondani, 1840
Micromya lucorum Rondani, 1840 [Jaschhof and Jaschhof 2009]
MONARDIA Kieffer, 1895
= ***Pezomyia*** Kieffer, 1913
sg. ***Monardia*** Kieffer, 1895
Monardia abnormis Mamaev, 1963 [Jaschhof and Jaschhof 2009]
Monardia armata Jaschhof, 2003 [Jaschhof and Jaschhof 2009]
Monardia lignivora (Felt, 1907) [Jaschhof and Jaschhof 2009]
Monardia obsoleta Edwards, 1938 [Jaschhof and Jaschhof 2009]
Monardia pediculata (Mamaev, 1993)
Monardia stirpium Kieffer, 1895 [Jaschhof and Jaschhof 2009]

Monardia yasumatsui Yukawa, 1967 [Jaschhof and Jaschhof 2009]
sg. ***Trichopteromyia*** Williston, 1896
Monardia magnifica Mamaev, 1963 [Jaschhof and Jaschhof 2009]
Monardia relicta Jaschhof, 2009 [Jaschhof and Jaschhof 2009]
sg. ***Xylopriona*** Kieffer, 1913
Monardia adentis Jaschhof, 1998 [Jaschhof and Jaschhof 2009]
Monardia atra (Meigen, 1804) [Jaschhof and Jaschhof 2009]
Monardia monotheca Edwards, 1938 [Jaschhof and Jaschhof 2009]
Monardia radiella Mamaev, 1993 [Jaschhof and Jaschhof 2009]
Monardia toxicodendri (Felt, 1907) [Jaschhof and Jaschhof 2009]
Monardia unguifera Berest & Mamaev, 1997 [Jaschhof and Jaschhof 2009]
POLYARDIS Pritchard, 1947
= ***Campyloneura*** Lengersdorf, 1913 preocc.
Polyardis adela Pritchard, 1947 [Jaschhof and Jaschhof 2009]
Polyardis bispinosa (Mamaev, 1963) [Jaschhof and Jaschhof 2009]
Polyardis silvalis (Rondani, 1840) [Jaschhof and Jaschhof 2009]
tribe Peromyiini Kleesattel, 1979
PEROMYIA Kieffer, 1894
Peromyia abdita Jaschhof, 2009 [Jaschhof and Jaschhof 2009]
Peromyia abnormis Mamaev & Berest, 1990 [Jaschhof and Jaschhof 2009]
Peromyia albicornis (Meigen, 1830) [Jaschhof and Jaschhof 2009]
Peromyia anatina Mamaev & Berest, 1990 [Jaschhof and Jaschhof 2009]
Peromyia angellifera Jaschhof, 1997 [Jaschhof and Jaschhof 2009]
Peromyia anisotoma Mamaev, 1994 [Jaschhof and Jaschhof 2009]
Peromyia apposita Jaschhof, 1997 [Jaschhof and Jaschhof 2009]
Peromyia bicolor (Edwards, 1938) [Jaschhof and Jaschhof 2009]
Peromyia bidentata Berest, 1998 [Jaschhof and Jaschhof 2009]
Peromyia boreophila Jaschhof, 2001 [Jaschhof and Jaschhof 2009]
Peromyia caricis (Kieffer, 1901) [Jaschhof and Jaschhof 2009]
Peromyia concitata Mamaev & Berest, 1994 [Jaschhof and Jaschhof 2009]
Peromyia cornuta (Edwards, 1938) [Jaschhof and Jaschhof 2009]
Peromyia curta Jaschhof, 1997 [Jaschhof and Jaschhof 2009]
Peromyia diadema Mamaev, 1963 [Jaschhof and Jaschhof 2009]
Peromyia edwardsi Berest, 1994 [Jaschhof and Jaschhof 2009]
Peromyia fagiphila Jaschhof, 1997 [Jaschhof and Jaschhof 2009]
Peromyia fungicola (Kieffer, 1901)
Peromyia intermedia (Kieffer, 1895) [Jaschhof and Jaschhof 2009]
Peromyia mitrata Jaschhof, 1997 [Jaschhof and Jaschhof 2009]
Peromyia modesta (Felt, 1907) [Jaschhof and Jaschhof 2009]
Peromyia monilis Mamaev, 1965 [Jaschhof and Jaschhof 2009]
Peromyia muscorum (Kieffer, 1895) [Jaschhof and Jaschhof 2009]
Peromyia ovalis (Edwards, 1938) [Jaschhof and Jaschhof 2009]
Peromyia palustris (Kieffer, 1895) [Jaschhof and Jaschhof 2009]
Peromyia perpusilla (Winnertz, 1870) [Jaschhof and Jaschhof 2009]
Peromyia photophila (Felt, 1907) [Jaschhof and Jaschhof 2009]
Peromyia pumila Jaschhof, 2001 [Jaschhof and Jaschhof 2009]
Peromyia ramosa (Edwards, 1938) [Jaschhof and Jaschhof 2009]
Peromyia ramosoides Jaschhof, 2009 [Jaschhof and Jaschhof 2009]
Peromyia sanguinea (Kieffer, 1894) [Jaschhof and Jaschhof 2009]
Peromyia scirrhosa Jaschhof, 2009 [Jaschhof and Jaschhof 2009]

Peromyia scutellata Mamaev, 1990 [Jaschhof and Jaschhof 2009]
Peromyia semotoides Jaschhof, 2009 [Jaschhof and Jaschhof 2009]
Peromyia subbicolor Jaschhof, 2009 [Jaschhof and Jaschhof 2009]
Peromyia subborealis Jaschhof, 1997 [Jaschhof and Jaschhof 2009]
Peromyia syltefjordensis Jaschhof, 1996 [Jaschhof and Jaschhof 2009]
Peromyia truncata Yukawa, 1967 [Jaschhof and Jaschhof 2009]
Peromyia tundrae Jaschhof, 1996 [Jaschhof and Jaschhof 2009]
Peromyia upupoides Jaschhof, 1997 [Jaschhof and Jaschhof 2009]
WINNERTZIINAE Panelius, 1965
tribe Diallactiini Jaschhof, 2013
DIALLACTIA Gagné, 2004
= ***Dalliactes*** Kieffer, 1894 preocc.
Diallactia crocea (Kieffer, 1894)
= *D. obscuripes* (Spungis, 1985) [Penttinen and Spungis 2007]
SYLVENOMYIA Mamaev & Zaitzev, 1998
Sylvenomyia fennica Penttinen & Jaschhof, 2009 [Penttinen and Jaschhof 2009]
Sylvenomyia spinigera (Spungis, 1985) [Penttinen and Jaschhof 2009]
tribe Heteropezini Schiner, 1868
HETEROPEZA Winnertz, 1846
Heteropeza pygmaea Winnertz, 1846 [Hackman 1980]
LEPTOSYNA Kieffer, 1894
Leptosyna similis Jaschhof, 2013 []
MIASTOR Meinert, 1864
Miastor metraloas Meinert, 1864 [Hackman 1980]
tribe Winnertziini Panelius, 1965
KRONOMYIA Felt, 1911
Kronomyia ovalis (Mamaev, 1964) []
RHIPIDOXYLOMYIA Mamaev, 1964
Rhipidoxylomyia brevicornis Mamaev, 1964 []
WINNERTZIA Rondani, 1860
Winnertzia bulbifera Mamaev, 1963 []
Winnertzia curvata Panelius, 1965 []
Winnertzia fusca Kieffer, 1901 []
Winnertzia globifera Mamaev, 1963 [Penttinen and Spungis 2007]
Winnertzia graduata Spungis, 1992 [Penttinen and Spungis 2007]
Winnertzia nigripennis Kieffer, 1896 [Penttinen and Spungis 2007]
Winnertzia rotundata Spungis, 1992 []
Winnertzia solidaginis Felt, 1907
= *W. calciequina* Felt, 1907 [Hackman 1980]
PORRICONDYLINAE Kieffer, 1913
tribe Asynaptini Rübsaamen & Hedicke, 1926
ASYNAPTA Loew, 1850
Asynapta breviata Spungis, 1988 []
Asynapta inflata Spungis, 1988 []
Asynapta magdalini Panelius, 1965 [Hackman 1980]
Asynapta pectoralis (Winnertz, 1853) [Hackman 1980]
Asynapta rufomaculata Panelius, 1965 [Hackman 1980]
Asynapta saliciperda Felt, 1908
= *A. populnea* Panelius, 1965 [Hackman 1980]
Asynapta strobi (Kieffer, 1920) [Penttinen and Spungis 2007]

CAMPTOMYIA Kieffer, 1894
Camptomyia abnormis Mamaev, 1961 [Panelius 1965]
Camptomyia calcarata Mamaev, 1964 []
Camptomyia corticalis (Loew, 1851) [Panelius 1965]
Camptomyia flavocinerea Panelius, 1965 [Hackman 1980]
Camptomyia gigantea Spungis, 1989 []
Camptomyia pinicola Mamaev, 1961 [Penttinen and Spungis 2007]
Camptomyia piptopori Panelius, 1965 [Hackman 1980]
Camptomyia regia Spungis, 1989 []
Camptomyia salicicola Mamaev, 1961 []
= *C. populicola* Mamaev, 1961 [Hackman 1980]
Camptomyia spinifera Mamaev, 1961 [Hackman 1980]
COLOMYIA Kieffer, 1892
Colomyia clavata Kieffer, 1892 []
PARASYNAPTA Panelius, 1965
Parasynapta intermedia Panelius, 1965 [Hackman 1980]
tribe Dicerurini Mamaev, 1966
DICERURA Kieffer, 1898
Dicerura furculata Mamaev, 1968 [Penttinen and Spungis 2007]
Dicerura mixta Spungis, 1987 [Penttinen and Spungis 2007]
Dicerura rossica (Mamaev, 1960) []
Dicerura triangularis Mamaev, 1966 []
Dicerura unidentata Spungis, 1987 []
DIRHIZA Loew, 1850
Dirhiza lateritia Loew, 1850 []
HILVERSIDIA Mamaev, 1966
Hilversidia autumnalis Mamaev, 1966 []
PARATETRANEUROMYIA Spungis, 1987
Paratetraneuromyia vernalis Spungis, 1987 [Penttinen and Siitonen 2005]
SOLNTSEVIA Mamaev, 1965
Solntsevia nigripes Mamaev, 1965 [Penttinen and Spungis 2007]
TETRANEUROMYIA Mamaev, 1964
Tetraneuromyia lenticularis (Spungis, 1987) []
tribe Porricondylini Kieffer, 1913
ARCTEPIDOSIS Mamaev, 1990
Arctepidosis paneliusi Mamaev & Zaitzev, 1998
CASSIDOIDES Mamaev, 1960
Cassidoides fulvus (Kieffer, 1896)
= *C. pini* Mamaev, 1960 [Hackman 1980]
CLASPETTOMYIA Grover, 1964
= ***Pachylabis*** Panelius, 1965
Claspettomyia hamata (Felt, 1907)
Claspettomyia niveitarsis (Zetterstedt, 1850) [Hackman 1980]
Claspettomyia rossica Mamaev, 1998 []
Claspettomyia ussuriensis Mamaev, 1998 []
COCCOPSILIS Harris, 2004
= ***Coccopsis*** de Meijere, 1901 preocc.
Coccopsilis marginata (de Meijere, 1901) [Penttinen and Spungis 2007]
Coccopsilis obscura (Mamaev, 1964) []
Coccopsilis paneliusi (Yukawa, 1971) [Penttinen and Spungis 2007]

DENDREPIDOSIS Mamaev, 1990
Dendrepidosis longipennis (Spungis, 1981) []
DIVELLEPIDOSIS Fedotova & Sidorenko, 2007
Divellepidosis armilla (Mamaev, 1994) []
Divellepidosis fuscostriata (Panelius, 1965) [Hackman 1980]
Divellepidosis hypoxantha (Panelius, 1965) [Hackman 1980]
Divellepidosis lutescens (Spungis, 1981) [Penttinen and Siitonen 2005]
Divellepidosis pallescens (Panelius, 1965) [Hackman 1980]
Divellepidosis taigacola Jaschhof, 2013 []
JAMALEPIDOSIS Mamaev, 1990
Jamalepidosis spungisi Jaschhof, 2013 []
MONEPIDOSIS Mamaev, 1966
Monepidosis furcata Mamaev, 1966 []
Monepidosis pectinata Mamaev, 1966 [Penttinen and Siitonen 2005]
PANELIUSIA Jaschhof, 2013
Paneliusia albimanoides Jaschhof, 2013 []
Paneliusia aurantiaca (Panelius, 1965) [Hackman 1980]
= *P. modesta* (Spungis, 1981) [Penttinen and Siitonen 2005]
PAREPIDOSIS Kieffer, 1913
Parepidosis arcuata Mamaev, 1964
= *P. longinodis* Panelius, 1965 [Panelius 1965]
Parepidosis venustior Gagné, 2004 [Penttinen and Spungis 2007]
PAURODYLA Jaschhof, 2013
Paurodyla tyresta Jaschhof, 2013 []
PORRICONDYLA Rondani, 1840
= ***Epidosis*** Loew, 1850
Porricondyla fulvescens Panelius, 1965 [Panelius 1965]
Porricondyla neglecta Mamaev *in* Mamaev & Krivosheina, 1965 [Hackman 1980]
Porricondyla nigripennis (Meigen, 1830) [Hackman 1980]
Porricondyla rufescens Panelius, 1965 [Hackman 1980]
PSEUDEPIDOSIS Mamaev, 1966
Pseudepidosis trifida Mamaev, 1966 [Penttinen and Siitonen 2005]
ROSTELLATYLA Jaschhof, 2013
Rostellatyla rostellata (Panelius, 1965) [Hackman 1980]
ROSTRATYLA Jaschhof, 2013
Rostratyla globosa (Spungis, 1981) [Penttinen and Spungis 2007]
SCHISTONEURUS Mamaev, 1964
Schistoneurus impressus Mamaev, 1964 []
Schistoneurus irregularis Mamaev, 1964 [Penttinen and Spungis 2007]
SERRATYLA Jaschhof, 2013
Serratyla furcata (Mamaev, 2001) []
Serratyla pubescens (Walker, 1856) [Hackman 1980]
Serratyla spinosa Jaschhof, 2013 []
SPUNGISOMYIA Mamaev & Zaitzev, 1996
Spungisomyia media (Spungis, 1981) [Penttinen and Spungis 2007]
ZAITZEVIOLA Fedotova & Sidorenko, 2007
Zaitzeviola latistylata Jaschhof, 2013 []
Zaitzeviola pilosistylata Jaschhof, 2013 []
Zaitzeviola rufocinerea (Panelius, 1965) [Panelius 1965]
CECIDOMYIINAE Newman, 1834

tribe unplaced in supertribe Cecidomyiidi
HARMANDIOLA Skuhravá, 1997
= ***Harmandia*** Kieffer, 1896 preocc.
Harmandiola globuli (Rübsaamen, 1889) [Hackman 1980]
Harmandiola tremulae (Winnertz, 1853)
= *H. loewi* (Rübsaamen, 1917) [Hackman 1980]
HYGRODIPLOSIS Kieffer, 1912
Hygrodiplosis vaccinii (Kieffer, 1897) [Hackman 1980]
MASSALONGIA Kieffer, 1897
Massalongia rubra (Kieffer, 1890) [Tiensuu 1963, Hackman 1980]
RESSELIELLA Seitner, 1906
= ***Thomasiniana*** Strand, 1927
Resseliella ribis (Marikovskij, 1956) [Thuneberg 1975, Hackman 1980]
Resseliella theobaldi (Barnes, 1927) [Hackman 1980]
XYLODIPLOSIS Kieffer, 1894
Xylodiplosis nigritarsis (Zetterstedt, 1850) [Hackman 1980]
tribe Aphidoletini Harris, 1966
APHIDOLETES Kieffer, 1904
Aphidoletes aphidimyza (Rondani, 1847) [Markkula et al. 1979, Hackman 1980]
Aphidoletes urticaria (Kieffer, 1895) [Kyrklund 1980]
MONOBREMIA Kieffer, 1912
Monobremia subterranea (Kieffer, 1898) [Kyrklund 1980]
tribe Asphondyliini Rübsaamen & Hedicke, 1925
ASPHONDYLIA Loew, 1850
Asphondylia serpylli Kieffer, 1898
= *A. thymi* Kieffer, 1898 [Hackman 1980]
KIEFFERIA Mik, 1895
Kiefferia pericarpiicola (Bremi, 1847) [Hackman 1980]
SCHIZOMYIA Kieffer, 1889
Schizomyia galiorum Kieffer, 1889 [Hackman 1980]
tribe Cecidomyiini Rübsaamen & Hedicke, 1925
ANISOSTEPHUS Rübsaamen, 1917
Anisostephus betulinus (Kieffer, 1889) [Hackman 1980]
CECIDOMYIA Meigen, 1803
Cecidomyia pini (De Geer, 1776) [Hackman 1980]
CONTARINIA Rondani, 1860
Contarinia coryli (Kaltenbach, 1859) [Hackman 1980]
Contarinia craccae Loew, 1850 [Hackman 1980]
Contarinia gei Kieffer, 1909
= *C. geicola* Rübsaamen, 1917 [Hackman 1980]
Contarinia kanervoi Barnes, 1958 [Barnes 1958, Hackman 1980]
Contarinia lonicerearum (F. Löw, 1877) []
Contarinia loti (De Geer, 1776) [Hackman 1980]
Contarinia merceri Barnes, 1930 [Hackman 1980]
Contarinia petioli (Kieffer, 1898) [Hackman 1980]
Contarinia pisi (Loew, 1850) [Hackman 1980]
Contarinia quercina (Rübsaamen, 1890) [Hackman 1980]
Contarinia sambuci (Kaltenbach, 1873)
= *C. lonicerearum* (F. Löw, 1874) [Hackman 1980]
Contarinia tiliarum (Kieffer, 1890) [Hackman 1980]

Contarinia tritici (Kirby, 1798) [Hackman 1980]
Contarinia vincetoxici Kieffer, 1909 [Widenfalk et al. 2002]
MACRODIPLOSIS Kieffer, 1895
Macrodiplosis pustularis (Bremi, 1847)
= *M. dryobia* (Löw, 1877) [Hackman 1980]
Macrodiplosis roboris (Hardy, 1854)
= *M. volvens* Kieffer, 1895 [Hackman 1980]
PLEMELIELLA Seitner, 1908
Plemeliella abietina Seitner, 1908 [Hackman 1980]
STENODIPLOSIS Reuter, 1895
Stenodiplosis geniculati Reuter, 1895 [Hackman 1980]
THECODIPLOSIS Kieffer, 1895
Thecodiplosis brachyntera (Schwägrichen, 1835) [Hackman 1980]
tribe Clinodiplosini Enderlein, 1936
AMETRODIPLOSIS Rübsaamen, 1910
Ametrodiplosis thalictricola (Rübsaamen, 1895) [Hackman 1980]
CLINODIPLOSIS Kieffer, 1894
Clinodiplosis cilicrus (Kieffer, 1889) []
SITODIPLOSIS Kieffer, 1913
Sitodiplosis mosellana (Géhin, 1857) [Hackman 1980]
tribe Hormomyiini Rübsaamen & Hedicke, 1925
PLANETELLA Westwood, 1840
? *Planetella grandis* (Meigen, 1804) [Hackman 1980]
tribe Lestodiplosini Harris, 1966
LESTODIPLOSIS Kieffer, 1894
Lestodiplosis pallidicornis Kieffer, 1898 [Hackman 1980]
tribe Dasineurini Rübsaamen & Hedicke, 1925
DASINEURA Rondani, 1840
Dasineura affinis (Kieffer, 1886) [Hackman 1980]
Dasineura alopecuri (Reuter, 1895) [Hackman 1980]
Dasineura angelicae Rübsaamen, 1916 [Hackman 1980]
Dasineura cardaminis (Winnertz, 1853) [Hackman 1980]
Dasineura engstfeldi (Rübsaamen, 1889) [Hackman 1980]
Dasineura epilobii (F. Löw, 1889) [Hackman 1980]
Dasineura fraxinea Kieffer, 1907 [Hackman 1980]
Dasineura fraxini (Bremi, 1847) [Hackman 1980]
Dasineura galiicola (F. Löw, 1880) [Hackman 1980]
Dasineura gentneri Pritchard, 1953 [Hackman 1980]
Dasineura hygrophila (Mik, 1883) [Hackman 1980]
Dasineura hyperici (Bremi, 1847) [Hackman 1980]
Dasineura jaapi Rübsaamen, 1914 [Hackman 1980]
Dasineura kiefferiana (Rübsaamen, 1891) [Hackman 1980]
Dasineura leguminicola (Lintner, 1879) [Hackman 1980]
Dasineura lotharingiae (Kieffer, 1888) [Hackman 1980]
Dasineura mali (Kieffer, 1904) [Hackman 1980]
Dasineura napi (Loew, 1850)
= *D. brassicae* (Winnertz, 1853) [Hackman 1980]
Dasineura populeti (Rübsaamen, 1889) [Hackman 1980]
Dasineura pteridicola (Kieffer, 1901) [Hackman 1980]
Dasineura pteridis (Muller, 1871)

= *D. filicina* (Kieffer, 1889) [Hackman 1980]
Dasineura pustulans (Rübsaamen, 1889) [Huldén 2004]
Dasineura pyri (Bouché, 1847) [Hackman 1980]
Dasineura ribis Barnes, 1940 [Hackman 1980]
Dasineura rosae (Bremi, 1847)
= *Wachtliella rosarum* (Hardy, 1854) [Hackman 1980]
Dasineura similis (F. Löw, 1888) [Hackman 1980]
Dasineura sisymbrii (Schrank, 1803) [Tiensuu 1963, Hackman 1980]
Dasineura tetensi (Rübsaamen, 1892) [Vappula 1941, Hackman 1980]
Dasineura thomasiana (Kieffer, 1888) [Hackman 1980]
Dasineura tiliae (Schrank, 1803)
= *D. tiliamvolvens* (Rübsaamen, 1889) [Hackman 1980]
Dasineura trifolii (F. Löw, 1874)
Dasineura ulmaria (Bremi, 1847) [Hackman 1980]
Dasineura urticae (Perris, 1840) [Hackman 1980]
Dasineura viciae (Kieffer, 1888) [Hackman 1980]
Dasineura violae (F. Löw, 1880) [Hackman 1980]
GEOCRYPTA Kieffer, 1913
Geocrypta galii (Loew, 1850) [Hackman 1980]
GEPHYRAULUS Rübsaamen, 1915
Gephyraulus raphanistri (Kieffer, 1886) [Hackman 1980]
GIRAUDIELLA Rübsaamen, 1915
Giraudiella inclusa (Frauenfeld, 1862) [Hackman 1980]
JAAPIELLA Rübsaamen, 1915
Jaapiella loticola (Rübsaamen, 1889)
Jaapiella veronicae (Vallot, 1827) [Hackman 1980]
KALTENBACHIOLA Hedicke, 1938
Kaltenbachiola strobi (Winnertz, 1853) [Hackman 1980]
MACROLABIS Kieffer, 1892
Macrolabis cirsii (Rübsaamen, 1890) [Hackman 1980]
Macrolabis orobi (F. Löw, 1877) [Hackman 1980]
RABDOPHAGA Westwood, 1847
Rabdophaga dubiosa Kieffer, 1913 [Hackman 1980]
Rabdophaga gemmicola (Kieffer, 1896) [Hackman 1980]
Rabdophaga heterobia (Loew, 1850) [Hackman 1980]
Rabdophaga iteobia (Kieffer, 1890) [Hackman 1980]
Rabdophaga marginemtorquens (Bremi, 1847) [Hackman 1980]
Rabdophaga nervorum (Kieffer, 1895) [Hackman 1980]
Rabdophaga rosaria (Loew, 1850) [Hackman 1980]
Rabdophaga salicis (Schrank, 1803) [Hackman 1980]
Rabdophaga terminalis (Loew, 1850) [Hackman 1980]
WACHTLIELLA Rübsaamen, 1915
Wachtliella caricis (Loew, 1850)
= *W. riparia* (Winnertz, 1853)
= *W. muricatae* (Meade, 1886) [Hackman 1980]
Wachtliella persicariae (Linnaeus, 1767) [Hackman 1980]
tribe Lasiopterini Rübsaamen & Hedicke, 1925
LASIOPTERA Meigen, 1818
Lasioptera populnea Wachtl, 1883 [Hackman 1980]
Lasioptera rubi (Schrank, 1803) [Hackman 1980]

tribe Oligotrophini Rübsaamen & Hedicke, 1925
OLIGOTROPHUS Latreille, 1805
Oligotrophus juniperinus (Linnaeus, 1758) [Hackman 1980]
Oligotrophus panteli Kieffer, 1898 [Hackman 1980]
tribe Poomyini Rübsaamen & Hedicke, 1925
MAYETIOLA Kieffer, 1896
Mayetiola avenae (Marchal, 1895) [Hackman 1980]
Mayetiola destructor (Say, 1817) [Hackman 1980]
Mayetiola joannisi Kieffer, 1896 [Hackman 1980]
tribe Rhopalomyiini Harris, 1966
RHOPALOMYIA Rübsaamen, 1892
Rhopalomyia chrysanthemi (Ahlberg, 1939) [Hackman 1980]
Rhopalomyia millefolii (Loew, 1850) [Hackman 1980]
Rhopalomyia ptarmicae (Vallot, 1849) [Hackman 1980]
Rhopalomyia tanaceticola (Karsch, 1879) [Hackman 1980]
Rhopalomyia tubifex (Bouché, 1847) [Hackman 1980]
tribe Unplaced in supertribe Lasiopteridi
CYSTIPHORA Kieffer, 1892
Cystiphora sanguinea (Bremi, 1847) [Hackman 1980]
= *C. hieracii* (F. Löw, 1874)
= *C. pilosellae* Kieffer, 1892
Cystiphora sonchi (Vallot, 1827) [Hackman 1980]
ITEOMYIA Kieffer, 1913
Iteomyia capreae (Winnertz, 1853) [Hackman 1980]
SEMUDOBIA Kieffer, 1913
Semudobia betulae (Winnertz, 1853) [Hackman 1980]

Excluded species

Anarete lacteipennis Kieffer, 1906 [Hackman 1980]
Camptomyia maxima Mamaev, 1961 [Hackman 1980]
Camptomyia piceae Panelius, 1965 [Hackman 1980]
Catarete brevinervis (Zetterstedt, 1851) [Hackman 1980]
Cecidomyia aurora Mannerheim, 1823 [Hackman 1980]
Epidosis flavescens F. Löw, 1874 [Hackman 1980]

BOLITOPHILIDAE Winnertz, 1864

Supporting references for *Jakovlev J (2014) Checklist of the fungus gnats of Finland: Bolitophilidae, Diadocidiidae, Ditomyiidae, Keroplatidae and Mycetophilidae (Diptera). In: Kahanpää J, Salmela J (Eds) Checklist of the Diptera of Finland. ZooKeys @@: @-@. doi: 10.3897/zookeys.??.7646*

BOLITOPHILA Meigen, 1818
sg. ***Bolitophila*** Meigen, 1818
Bolitophila austriaca (Mayer, 1950) [Polevoi 1995a]
Bolitophila basicornis (Mayer, 1951) [Hackman 1980]
Bolitophila caspersi Plassmann, 1956 [Polevoi et al. 2006]
Bolitophila cinerea Meigen, 1818 [Hackman 1980]
Bolitophila saundersii (Curtis, 1836) [Hackman 1980]
Bolitophila tenella Winnertz, 1864 [Hackman 1980]
sg. ***Cliopisa*** Enderlein, 1936
Bolitophila aperta Lundström, 1915 [Hackman 1980]
Bolitophila bimaculata Zetterstedt, 1838 [Hackman 1980]
Bolitophila dubia Siebke, 1863 [Hackman 1980]
= *B. disjuncta* Loew, 1869
Bolitophila fumida Edwards, 1941 [Hackman 1980]
Bolitophila glabrata Loew, 1869 [Hackman 1980]
Bolitophila hybrida (Meigen, 1804) [Hackman 1980]
Bolitophila ingrica Stackelberg, 1969 [Salmela et al. 2014]
Bolitophila limitis Polevoi, 1996 [Polevoi 1995b]
Bolitophila maculipennis Walker, 1836 [Hackman 1980]
= *B. coronata* Mayer, 1951
Bolitophila modesta Lackschewitz, 1937 [Hackman 1980]
= *B. tarsata* Mayer, 1951 [Hackman 1980]
Bolitophila nigrolineata Landrock, 1912 [Polevoi 1995a,b]
Bolitophila obscurior Stackelberg, 1969 [Polevoi 1995a,b]
Bolitophila occlusa Edwards, 1913 [Hackman 1980]
Bolitophila pseudohybrida Landrock, 1912 [Hackman 1980]
Bolitophila rossica Landrock, 1912 [Hackman 1980]

Excluded species

Bolitophila spinigera Edwards, 1925 [Hackman 1980]

DIADOCIDIIDAE Winnertz, 1864

Supporting references for *Jakovlev J (2014) Checklist of the fungus gnats of Finland: Bolitophilidae, Diadocidiidae, Ditomyiidae, Keroplatidae and Mycetophilidae (Diptera). In: Kahanpää J, Salmela J (Eds) Checklist of the Diptera of Finland. ZooKeys @@: @-@. doi: 10.3897/zookeys.??.7646*

DIADOCIDIA Ruthe, 1831
sg. ***Adidocidia*** Laštovka & Matile, 1972
Diadocidia trispinosa Polevoi, 1996 [Polevoi 1995b]
Diadocidia valida Mik, 1874 [Hackman 1979b, 1980]
sg. ***Diadocidia*** Ruthe, 1831
Diadocidia ferruginosa (Meigen, 1830) [Hackman 1980]
Diadocidia fissa Zaitzev, 1994 [Polevoi et al. 2006]
Diadocidia spinosula Tollet, 1948 [Hackman 1979b, 1980]

KEROPLATIDAE Rondani, 1856

Supporting references for *Jakovlev J (2014) Checklist of the fungus gnats of Finland: Bolitophilidae, Diadocidiidae, Ditomyiidae, Keroplatidae and Mycetophilidae (Diptera). In: Kahanpää J, Salmela J (Eds) Checklist of the Diptera of Finland. ZooKeys @@: @-@. doi: 10.3897/zookeys.??.7646*

KEROPLATINAE Rondani, 1856
tribe Keroplatini Rondani, 1856
CEROTELION Rondani, 1856
Cerotelion striatum (Gmelin, 1790)
= *C. lineatum* (Fabricius, 1775) preocc.
KEROPLATUS Bosc, 1792
Keroplatus testaceus Dalman, 1818 [Hackman 1980]
Keroplatus tipuloides Bosc, 1792 [Hackman 1980]
= *K. sesioides* Wahlberg, 1839 [Hackman 1980]
? *Keroplatus tuvensis* Zaitzev, 1991
ROCETELION Matile, 1988
Rocetelion humerale (Zetterstedt, 1850) [Hackman 1980]
tribe Orfeliini Matile, 1990
ISONEUROMYIA Brunetti, 1912
Isoneuromyia semirufa (Meigen, 1818) [Hackman 1980]
MACRORRHYNCHA Winnertz, 1846
Macrorrhyncha flava Winnertz, 1846 [Hackman 1980]
Macrorrhyncha rostrata (Zetterstedt, 1851) [Hackman 1980]
MONOCENTROTA Edwards, 1925
Monocentrota lundstroemi Edwards, 1925 [Hackman 1980]
NEOPLATYURA Malloch, 1928
Neoplatyura flava (Macquart, 1826) [Polevoi 1995a,b]
Neoplatyura modesta (Winnertz, 1864) [Hackman 1980]
ORFELIA Costa, 1857
Orfelia discoloria (Meigen, 1818) [Hackman 1980]
Orfelia falcata Zaitzev, 1994 [Polevoi 1995b]
Orfelia fasciata (Meigen, 1804) [Hackman 1980]
Orfelia krivosheinae Zaitzev, 1994 [Salmela et al. 2014]
Orfelia lugubris (Zetterstedt, 1851) [Salmela et al. 2014]
= *O. tristis* (Lundström, 1911)
Orfelia nemoralis (Meigen, 1818) [Hackman 1980]
Orfelia nigricornis (Fabricius, 1805) [Hackman 1980]
Orfelia ochracea (Meigen, 1818)
= *O. unicolor* (Staeger, 1840) [Hackman 1980]
Orfelia pallida (Staeger, 1840) [Salmela et al. 2014]
PYRATULA Edwards, 1929
Pyratula perpusilla (Edwards, 1913) [Polevoi et al. 2006]
Pyratula subcanariae Chandler, 2001 [Salmela et al. 2014]
Pyratula zonata (Zetterstedt, 1855) [Hackman 1980]
URYTALPA Edwards, 1929
Urytalpa atriceps (Edwards, 1913) [Salmela et al. 2014]
Urytalpa dorsalis (Staeger, 1840)
= *U. ochracea* auct. nec (Meigen, 1818) [Hackman 1980]

Urytalpa galdes Hedmark & Kjærandsen, 2009 [Kjæ randsen 2012, Salmela et al. 2014]
Urytalpa macrocera (Edwards, 1913) [Salmela et al. 2014]
Urytalpa trivittata (Lundström, 1914) [Hackman 1980]
MACROCERINAE Rondani, 1856
tribe Macrocerini Rondani, 1856
MACROCERA Meigen, 1803
Macrocera angulata Meigen, 1818 [Hackman 1980]
Macrocera centralis Meigen, 1818 [Hackman 1980]
Macrocera crassicornis Winnertz, 1864 [Salmela et al. 2014]
Macrocera estonica Landrock, 1924 [Polevoi et al. 2006]
Macrocera fasciata Meigen, 1804 [Hackman 1980]
Macrocera fascipennis Staeger, 1840 [Hackman 1980]
Macrocera grandis Lundström, 1912 [Hackman 1980]
= *M. magna* Landrock, 1917
Macrocera lutea Meigen, 1804 [Hackman 1980]
Macrocera maculata Meigen, 1818 [Hackman 1980]
Macrocera nigricoxa Winnertz, 1864 [Polevoi et al. 2006]
Macrocera parva Lundström, 1914 [Hackman 1980]
Macrocera phalerata Meigen, 1818 [Hackman 1980]
Macrocera pilosa Landrock, 1917 [Hackman 1980]
Macrocera pumilio Loew, 1869 [Hackman 1980]
Macrocera pusilla Meigen, 1830 [Polevoi et al. 2006]
Macrocera stigma Curtis, 1837 [Hackman 1980]
Macrocera stigmoides Edwards, 1925 [Hackman 1980]
Macrocera vittata Meigen, 1830 [Hackman 1980]
Macrocera zetterstedti Lundström, 1914 [Hackman 1980]
= *M. nana* Zetterstedt, 1860

Excluded species

Macrocera nigropicea Lundström, 1906 [Hackman 1980]
Macrocera sudetica Landrock, 1924 females only [Hackman 1980]
Monoclona mikii Kertesz, 1898
Orfelia bicolor (Macquart, 1826) females only
= *O. basalis* (Winnertz, 1864) [Hackman 1980]

MYCETOPHILIDAE Newman, 1834

Supporting references for *Jakovlev J (2014) Checklist of the fungus gnats of Finland: Bolitophilidae, Diadocidiidae, Ditomyiidae, Keroplatidae and Mycetophilidae (Diptera). In: Kahanpää J, Salmela J (Eds) Checklist of the Diptera of Finland. ZooKeys @@: @-@. doi: 10.3897/zookeys.??.7646*

MYCOMYINAE Edwards, 1925
MYCOMYA Rondani, 1856
sg. ***Calomycomya*** Väisänen, 1984
Mycomya avala Väisänen, 1984 [Väisänen 1984]
Mycomya pulchella (Dziedzicki, 1885) [Hackman 1980]
sg. ***Coheromyia*** Väisänen, 1984
Mycomya branderi Väisänen, 1984 [Väisänen 1984]
= *M. tantilla* misid. [Hackman 1980, Väisänen 1984]
sg. ***Cymomya*** Väisänen, 1984
Mycomya circumdata (Staeger, 1840) [Hackman 1980]
sg. ***Lycomya*** Väisänen, 1984 preocc.!
Mycomya pectinifera Edwards, 1924 [Hackman 1980]
sg. ***Mycomya*** Rondani, 1856
Mycomya annulata (Meigen, 1818)
= *M. incisurata* (Zetterstedt, 1838) [Hackman 1980]
Mycomya bialorussica Landrock, 1925 [Hackman 1980]
Mycomya bicolor (Dziedzicki, 1885) [Hackman 1980]
Mycomya bisulca Lackschewitz, 1937 [Hackman 1980]
Mycomya britteni Kidd, 1955 [Hackman 1980]
Mycomya brunnea (Dziedzicki, 1885) [Hackman 1980]
Mycomya cinerascens (Macquart, 1826) [Hackman 1980]
= *M. hyalinata* (Meigen, 1830) [Hackman 1980]
Mycomya collini Edwards, 1941 [Hackman 1980]
Mycomya danielae Matile, 1972 [Hackman 1980]
Mycomya denmax Väisänen, 1979 [Väisänen 1979, Hackman 1980]
Mycomya digitifera Edwards, 1925 [Hackman 1980]
Mycomya disa Väisänen, 1984 [Väisänen 1984]
Mycomya dziedzickii Väisänen, 1984
= *M. fulva* (Dziedzicki, 1885) [Hackman 1980] preocc.
Mycomya egregia (Dziedzicki, 1885) [Hackman 1980]
Mycomya festivalis Väisänen, 1984 [Väisänen 1984]
Mycomya flavicollis (Zetterstedt, 1852) [Hackman 1980]
Mycomya forestaria Plassmann, 1978 [Hackman 1980]
Mycomya fuscata (Winnertz, 1864) [Polevoi et al. 2006]
Mycomya griseovittata (Zetterstedt, 1852)
= *M. fasciata* (Zetterstedt, 1838) [Hackman 1980] preocc.
= *M. clavigera* (Lundström, 1912) [Hackman 1980]
Mycomya hackmani Väisänen, 1984 [Väisänen 1984]
Mycomya heydeni Plassmann, 1970 [Hackman 1980]
Mycomya hians (Lundström, 1912) [Hackman 1980]
Mycomya hiisi Väisänen, 1979 [Väisänen 1979, Hackman 1980]
Mycomya humida Garrett, 1924 [Polevoi 2001a]
= *M. curvata* Fisher, 1937 [Hackman 1980]

Mycomya indistincta Polevoi, 1995 [Zaitzev and Polevoi 1995]
Mycomya insignis (Winnertz, 1864)
= *M. wrzesniowskii* (Dziedzicki, 1885) [Hackman 1980]
Mycomya islandica Väisänen, 1984 [Väisänen 1984]
Mycomya karelica Väisänen, 1979 [Väisänen 1979, Hackman 1980]
Mycomya kaurii Väisänen, 1979 [Väisänen 1979, Hackman 1980]
Mycomya kuusamoensis Väisänen, 1979 [Väisänen 1979, Hackman 1980]
Mycomya lambi Edwards, 1941 [Hackman 1980]
Mycomya leporina Väisänen, 1984 [Väisänen 1984]
Mycomya levis (Dziedzicki, 1885) [Hackman 1980]
Mycomya livida (Dziedzicki, 1885) [Jakovlev et al. 2006]
Mycomya maculata (Meigen, 1804) [Hackman 1980]
Mycomya marginata (Meigen, 1818) [Hackman 1980]
= *M. limbata* (Winnertz, 1864)
Mycomya mituda Väisänen, 1980 [Väisänen 1980]
Mycomya neohyalinata Väisänen, 1984 [Väisänen 1984]
Mycomya nigricornis (Zetterstedt, 1852)
= *M. melanoceras* Edwards, 1924 [Hackman 1980]
Mycomya nitida (Zetterstedt, 1852)
= *M. exigua* (Winnertz, 1864) [Hackman 1980]
Mycomya norna Väisänen, 1984 [Polevoi et al. 2006]
Mycomya occultans (Winnertz, 1864) [Hackman 1980]
Mycomya ornata (Meigen, 1818) [Hackman 1980]
Mycomya parva (Dziedzicki, 1885) [Jakovlev et al. 2006]
Mycomya prominens (Lundström, 1913) [Hackman 1980]
Mycomya pseudoapicalis Landrock, 1925 [Hackman 1980]
Mycomya pseudocurvata Väisänen, 1979 [Väisänen 1979, Hackman 1980]
Mycomya punctata (Meigen, 1804) [Hackman 1980]
Mycomya ruficollis (Zetterstedt, 1852) [Hackman 1980]
Mycomya safena Väisänen, 1984 [Salmela et al. 2014]
Mycomya shermani Garrett, 1924
= *M. kingi* Edwards, 1941 [Hackman 1980]
Mycomya shewelli Väisänen, 1984 [Salmela et al. 2014]
Mycomya siebecki (Landrock, 1912) [Hackman 1980]
Mycomya sieberti Landrock, 1930) [Salmela et al. 2014]
Mycomya sigma Johannsen, 1910
= *M. duplicata* Edwards, 1925 [Hackman 1980]
Mycomya simulans Väisänen, 1984 [Polevoi 2001b]
Mycomya spinicoxa Väisänen, 1979 [Väisänen 1979, Hackman 1980]
Mycomya storai Väisänen, 1979 [Väisänen 1979, Hackman 1980]
Mycomya subarctica Väisänen, 1979 [Väisänen 1979, Hackman 1980]
Mycomya tenuis (Walker, 1856) [Hackman 1980]
Mycomya thula Väisänen, 1984 [Salmela et al. 2014]
Mycomya trivittata (Zetterstedt, 1838) [Hackman 1963, 1980]
Mycomya tumida (Winnertz, 1864) [Hackman 1963, 1980]
Mycomya vittiventris (Zetterstedt, 1852) [Hackman 1980]
= *M. elegans* (Lundström, 1912)
Mycomya wankowiczii (Dziedzicki, 1885) [Hackman 1980]
Mycomya winnertzi (Dziedzicki, 1885) [Hackman 1980]
sg. ***Mycomyopsis*** Väisänen, 1984

Mycomya affinis (Staeger, 1840)
= *M. flava* (Winnertz, 1864) [Hackman 1980]
Mycomya confusa Väisänen, 1977 [Väisänen 1979, Hackman 1980]
Mycomya fennica Väisänen, 1979 [Väisänen 1979, Hackman 1980]
Mycomya maura (Walker, 1856)
Mycomya neolittoralis Väisänen, 1984 [Väisänen 1984]
Mycomya paradentata Väisänen, 1984 [Väisänen 1984]
= *M. dentata* auct. nec Fisher, 1937 [Hackman 1980, Väisänen 1984]
Mycomya penicillata (Dziedzicki, 1885) [Väisänen 1984]
= *M. maura* (Walker, 1856) misid. [Hackman 1980, Väisänen 1984]
Mycomya permixta Väisänen, 1984 [Väisänen 1984]
Mycomya trilineata (Zetterstedt, 1838) [Hackman 1980]
= *M. fusca* (Meigen, 1818) [Hackman 1980, Väisänen 1984]
sg. ***Neomycomya*** Väisänen, 1984
Mycomya fimbriata (Meigen, 1818) [Hackman 1980]
NEOEMPHERIA Osten Sacken, 1878
= ***Empheria*** Winnertz, 1864 preocc.
Neoempheria bimaculata (von Roser, 1840) [Jakovlev et al. 2006]
Neoempheria pictipennis (Haliday, 1833) [Hackman 1980]
Neoempheria striata (Meigen, 1818) [Hackman 1980]
Neoempheria tuomikoskii Väisänen, 1982 [Väisänen 1982]
Neoempheria winnertzi Edwards, 1913 [Polevoi et al. 2006]
SCIOPHILINAE Rondani, 1840
ACNEMIA Winnertz, 1864
Acnemia amoena Winnertz, 1864 [Salmela et al. 2014]
Acnemia angusta Zaitzev, 1982 [Polevoi 2001a,b]
Acnemia falcata Zaitzev, 1982 [Polevoi 2001a,b]
Acnemia longipes Winnertz, 1864 [Polevoi 2001a,b]
Acnemia nitidicollis (Meigen, 1818) [Hackman 1980]
Acnemia trifida Zaitzev, 1982 [Salmela et al. 2014]
ALLOCOTOCERA Mik, 1886
Allocotocera pulchella (Curtis, 1837) [Hackman 1980]
ANACLILEIA Meunier, 1904
Anaclileia dispar (Winnertz, 1864) [Salmela et al. 2014]
Anaclileia dziedzickii (Landrock, 1911) [Salmela et al. 2014]
AZANA Walker, 1856
sg. ***Azana*** Walker, 1856
Azana anomala (Staeger, 1840) [Hackman 1980]
COELOPHTHINIA Edwards, 1941
Coelophthinia thoracica (Winnertz, 1864) [Polevoi 1995a]
EUDICRANA Loew, 1869
Eudicrana nigriceps (Lundström, 1909) [Hackman 1980]
LEPTOMORPHUS Curtis, 1831
sg. ***Leptomorphus*** Curtis, 1831
Leptomorphus forcipatus Landrock, 1918
Leptomorphus subforcipatus Zaitzev & Ševčík, 2002 [Zaitzev and Ševčík 2002]
= *L. quadrimaculatus* auct. nec Matsumura, 1910 [Polevoi 1995a, Zaitzev and Ševčík 2002]
Leptomorphus walkeri Curtis, 1831 [Hackman 1980]
MEGALOPELMA Enderlein, 1911

Megalopelma nigroclavatum (Strobl, 1910) [Hackman 1980]
MONOCLONA Mik, 1886
Monoclona braueri (Strobl, 1895) [Polevoi 2001a,b]
Monoclona rufilatera (Walker, 1837) [Hackman 1980]
Monoclona silvatica Zaitzev, 1983 [Salmela et al. 2014]
NEURATELIA Rondani, 1856
Neuratelia nemoralis (Meigen, 1818) [Hackman 1980]
Neuratelia sintenisi Lackschewitz, 1937 [Polevoi et al. 2006]
PARATINIA Mik, 1874
Paratinia sciarina Mik, 1874 [Polevoi 1995a]
PHTHINIA Winnertz, 1864
Phthinia congenita Plassmann, 1984 [Polevoi 2001a,b]
Phthinia humilis Winnertz, 1864 [Hackman 1980]
Phthinia mira (Ostroverkhova, 1977) [Polevoi and Jakovlev 2004]
= *P. plassmanni* Caspers, 1984
Phthinia setosa Zaitzev, 1994 [Polevoi et al. 2006]
Phthinia winnertzi Mik, 1869 [Jakovlev et al. 2006]
POLYLEPTA Winnertz, 1864
Polylepta borealis Lundström, 1912 [Hackman 1980]
Polylepta guttiventris (Zetterstedt, 1852) [Hackman 1980]
SCIOPHILA Meigen, 1818
Sciophila adamsi Edwards, 1925 [Hackman 1980]
Sciophila antiqua Chandler, 1987
= *S. hebes* auct. nec Johannsen, 1910 [Komonen 2001, Polevoi and Jakovlev 2004]
Sciophila bicuspidata Zaitzev, 1982 [Salmela et al. 2014]
Sciophila buxtoni Freeman, 1956 [Polevoi 1995a]
Sciophila caesarea Chandler, 2001 [Salmela et al. 2014]
Sciophila dziedzickii Edwards, 1925 [Hackman 1971a, 1980]
Sciophila fenestella Curtis, 1837 [Hackman 1980]
Sciophila fuliginosa Holmgren, 1883 [Hackman 1980]
Sciophila geniculata Zetterstedt, 1838 [Hackman 1980]
Sciophila hirta Meigen, 1818
Sciophila karelica Zaitzev, 1982 [Jakovlev et al. 2006]
Sciophila krysheni Polevoi, 2001 [Polevoi 2001b]
Sciophila limbatella Zetterstedt, 1852 [Hackman 1980]
Sciophila lutea Macquart, 1826 [Hackman 1971a, 1980]
Sciophila minuta Zaitzev, 1982 [Jakovlev et al. 2006]
Sciophila modesta Zaitzev, 1982 [Polevoi et al. 2006]
Sciophila nigronitida Landrock, 1925 [Hackman 1980]
Sciophila nonnisilva Hutson, 1979 [Polevoi et al. 2006]
Sciophila persubtilis Polevoi, 2001 [Polevoi et al. 2006]
Sciophila pomacea Chandler, 2006
= *S. ochracea* Stephens in Walker, 1856 preocc.[Hackman 1980]
Sciophila pseudoflexuosa Kurina, 1991 [Jakovlev et al. 2006]
Sciophila plurisetosa Edwards, 1921 []
Sciophila rufa Meigen, 1830 [Hackman 1980]
Sciophila salassea Matile, 1983 [Salmela et al. 2014]
Sciophila setosa Garrett, 1925 [Jakovlev et al. 2006]
Sciophila thoracica Staeger, 1840
= *S. quadriterga* (Hutson, 1979) [Polevoi 1995a]

Sciophila varia (Winnertz, 1864) [Hackman 1980]
Sciophila yakutica Blagoderov, 1992 [Polevoi 2001a,b]
GNORISTINAE Edwards, 1925
ACOMOPTERA Vockeroth, 1980
Acomoptera difficilis (Dziedzicki 1885) [Polevoi 2001a]
Acomoptera spinistylus (Søli, 1993) [Jakovlev et al. 2006]
APOLEPHTHISA Grzegorzek, 1885
Apolephthisa subincana (Curtis, 1837) [Hackman 1980]
BOLETINA Staeger, 1840
Boletina atridentata Polevoi & Hedmark, 2004 [Salmela et al. 2014]
Boletina basalis (Meigen, 1818) [Hackman 1980]
Boletina bidenticulata Sasakawa & Kimura, 1974 [Zaitzev 2006]
Boletina borealis Zetterstedt, 1852 [Hackman 1980]
Boletina brevicornis Zetterstedt, 1852 [Hackman 1980]
Boletina cincticornis (Walker, 1848) [Hackman 1980]
Boletina cordata Polevoi & Hedmark, 2004 [Polevoi and Hedmark 2004]
Boletina cornuta Zaitzev, 1994 [Jakovlev et al. 2006]
Boletina digitata Lundström, 1914 [Polevoi et al. 2006]
Boletina dispecta Dziedzicki, 1885 [Hackman 1980]
Boletina dispectoides Jakovlev & Penttinen, 2007 [Jakovlev and Penttinen 2007]
Boletina dissipata Plassmann, 1986 [Hedmark 1998]
Boletina dubia (Meigen, 1804) [Hackman 1980]
Boletina edwardsi Chandler, 1992 [Chandler 1992b]
Boletina falcata Polevoi & Hedmark, 2004 [Polevoi and Hedmark 2004]
Boletina fennoscandica Polevoi & Hedmark, 2004 [Polevoi and Hedmark 2004]
Boletina gripha Dziedzicki, 1885 [Hackman 1980]
Boletina griphoides Edwards, 1925 [Hac 1962, Hackman 1980]
Boletina groenlandica Staeger, 1845 [Hackman 1980]
Boletina gusakovae Zaitzev, 1994 [Zaitzev 2006]
Boletina hedstroemi Polevoi & Hedmark, 2004 [Polevoi and Hedmark 2004]
Boletina intermedia Lundström, 1915 [Salmela et al. 2014]
Boletina jamalensis Zaitzev, 1994 [Polevoi et al. 2006]
= *B. struthioides* Polevoi & Hedmark, 2004 [Polevoi and Hedmark 2004]
Boletina kivachiana Polevoi & Hedmark, 2004 [Polevoi and Hedmark 2004]
Boletina kurilensis Zaitzev, 1994 [Jakovlev et al. 2006]
Boletina landrocki Edwards, 1924 [Hackman 1980]
Boletina lapponica Polevoi & Hedmark, 2004 [Salmela et al. 2014]
Boletina lundbecki Lundström, 1912 [Polevoi and Jakovlev 2004]
Boletina lundstroemi Landrock, 1912 [Hackman 1980]
Boletina maculata Holmgren, 1870
= *B. apicalis* (Walker, 1848) preocc.[Hackman 1980]
Boletina minuta Polevoi in Zaitzev & Polevoi, 1995 [Jakovlev et al. 2006]
Boletina moravica Landrock, 1912 [Hackman 1980]
Boletina nasuta (Haliday, 1839) [Hackman 1980]
Boletina nigricans Dziedzicki, 1885 [Hackman 1980]
Boletina nigricoxa Staeger, 1840 [Hackman 1980]
Boletina nigrofusca Dziedzicki, 1885 [Hackman 1980]
Boletina nitida Grzegorzek, 1885 [Zaitzev 2006]
Boletina nitiduloides Zaitzev, 1994 [Polevoi 2001a]
Boletina onegensis Polevoi in Zaitzev & Polevoi, 1995 [Polevoi 2001a]

Boletina pallidula Edwards, 1925 [Polevoi et al. 2006]
Boletina palmata Polevoi, 2013 [Salmela et al. 2014]
Boletina pectinunguis Edwards, 1932 [Hackman 1971a, 1980]
Boletina pinusia Maximova, 2001 [Salmela et al. 2014]
Boletina plana Walker, 1856 [Hackman 1980]
Boletina polaris Lundström, 1915 [Salmela et al. 2014]
Boletina populina Polevoi in Zaitzev & Polevoi, 1995 [Polevoi 2001a]
Boletina pseudonitida Zaitzev, 1994 [Salmela et al. 2014]
Boletina rejecta Edwards, 1941 [Hackman 1980]
Boletina sciarina Staeger, 1840 [Hackman 1980]
Boletina silvatica Dziedzicki, 1885 [Hackman 1980]
Boletina subtriangularis Polevoi & Hedmark, 2004 [Polevoi and Hedmark 2004]
Boletina takagii Sasakawa & Kimura, 1974 [Jakovlev et al. 2006]
Boletina tiroliensis Plassmann, 1980 [Polevoi et al. 2006]
Boletina triangularis Polevoi in Zaitzev & Polevoi, 1995 [Jakovlev et al. 2006]
Boletina trispinosa Edwards, 1913 [Hackman 1980]
Boletina trivittata (Meigen, 1818) [Hackman 1980]
Boletina verticillata Stackelberg, 1943 [Hackman 1980]
Boletina villosa Landrock, 1912 [Hackman 1980]
COELOSIA Winnertz, 1864
Coelosia bicornis Stackelberg, 1946 [Sø li 1997]
Coelosia flava (Staeger, 1840) [Hackman 1980]
Coelosia fusca Bezzi, 1892
= *C. silvatica* Landrock, 1918 [Hackman 1980]
Coelosia gracilis Johannsen, 1912 [Salmela et al. 2014]
Coelosia limpida Plassmann, 1986 [Salmela et al. 2014]
Coelosia tenella Zetterstedt, 1852 [Hackman 1980]
Coelosia truncata Lundström, 1909 [Hackman 1980]
DZIEDZICKIA Johannsen, 1909
Dziedzickia marginata (Dziedzicki, 1885) [Hackman 1980]
ECTREPESTHONEURA Enderlein, 1911
Ectrepesthoneura colyeri Chandler, 1980 [Hackman 1980]
Ectrepesthoneura hirta (Winnertz, 1846) [Hackman 1980]
Ectrepesthoneura nigra Zaitzev, 1984 [Salmela et al. 2014]
Ectrepesthoneura ovata Ostroverkhova, 1977
= *E. bucera* Plassmann, 1980 [Hackman 1980]
Ectrepesthoneura pubescens (Zetterstedt, 1860) [Hackman 1980]
Ectrepesthoneura referta Plassmann, 1976 [Polevoi 1995a]
Ectrepesthoneura tori Zaitzev & Økland, 1994 [Polevoi et al. 2006]
GNORISTE Meigen, 1818
Gnoriste apicalis Meigen, 1818 [Hackman 1980]
Gnoriste bilineata Zetterstedt, 1852 [Hac 1963, Karppinen 1964b, Hackman 1980]
GRZEGORZEKIA Edwards, 1941
Grzegorzekia collaris (Meigen, 1818) [Hackman 1980]
HADRONEURA Lundström, 1906
Hadroneura palmeni Lundström, 1906 [Hackman 1980]
IMPLETA Plassmann, 1978
Impleta consorta Plassmann, 1978 [Kurina 2004]
KATATOPYGIA Martinsson & Kjærandsen, 2012
Katatopygia erythropyga (Holmgren, 1883) [Polevoi 2001a]

Katatopygia sahlbergi (Lundström, 1906) [Hackman 1980]
PALAEODOCOSIA Meunier, 1904
Palaeodocosia vittata (Coquillett, 1901)
= *P. janickii* (Dziedzicki, 1923) [Hedmark 1998]
= *P. alpicola* misid. [Hackman 1980]
SAIGUSAIA Vockeroth, 1980
Saigusaia flaviventris (Strobl, 1894) [Hackman 1980]
SPEOLEPTA Edwards, 1925
Speolepta leptogaster (Winnertz, 1864) [Hackman 1980]
SYNAPHA Meigen, 1818
Synapha fasciata Meigen, 1818 [Hackman 1980]
Synapha vitripennis (Meigen, 1818) [Hackman 1980]
SYNTEMNA Winnertz, 1864
Syntemna daisetsuzana Okada, 1938 [Hackman 1980]
Syntemna elegantia Plassmann, 1978 [Polevoi 2003]
Syntemna hungarica (Lundström, 1912) [Hackman 1980]
Syntemna morosa Winnertz, 1864 [Polevoi 2003]
Syntemna nitidula Edwards, 1925 [Hackman 1980]
Syntemna oulankaensis Polevoi, 2003 [Polevoi 2003]
Syntemna penicilla Hutson, 1979 [Hackman 1980]
Syntemna relicta (Lundström, 1912) [Hackman 1980]
Syntemna setigera (Lundström, 1914) [Hackman 1980]
= *S. haagvari* Økland, 1995
Syntemna stylata Hutson, 1979 [Polevoi 2001a]
Syntemna stylatoides Zaitzev, 1994 [Polevoi 2001a]
TETRAGONEURA Winnertz, 1846
Tetragoneura ambigua Grzegorzek, 1885 [Polevoi 2001a]
Tetragoneura obirata Plassmann, 1990 [Polevoi 2001a]
Tetragoneura pudogensis Polevoi & Jakovlev, 2011 [Polevoi and Jakovlev 2011]
Tetragoneura ruuhijarvii Polevoi & Jakovlev, 2011 [Polevoi and Jakovlev 2011]
Tetragoneura sylvatica (Curtis, 1837) [Polevoi et al. 2006]
LEIINAE Edwards, 1925
CLASTOBASIS Skuse, 1890
Clastobasis alternans (Winnertz, 1864) [Hackman 1980]
DOCOSIA Winnertz, 1864
Docosia expectata Laštovka & Ševčík, 2006 [Salmela et al. 2014]
Docosia flavicoxa Strobl, 1900 [Hackman 1980, Salmela et al. 2014]
= *D. pallipes* Edwards, 1941
Docosia gilvipes (Haliday in Walker, 1856) [Mee 1961a, Hackman 1980]
Docosia landrocki Laštovka & Ševčík, 2006 [Salmela et al. 2014]
Docosia muelleri Plassmann, 1986 [Salmela et al. 2014]
Docosia tibialis Laštovka & Ševčík, 2006 [Salmela et al. 2014]
GREENOMYIA Brunetti, 1912
Greenomyia baikalica Zaitzev, 1994 [Polevoi 2001a]
Greenomyia borealis (Winnertz, 1864) [Hackman 1980]
Greenomyia mongolica Laštovka & Matile, 1974 [Salmela et al. 2014]
LEIA Meigen, 1818
Leia bilineata (Winnertz, 1864) [Hackman 1980]
Leia bimaculata (Meigen, 1804) [Hackman 1980]
Leia crucigera Zetterstedt, 1838 [Hedmark 1998]

Leia cylindrica (Winnertz, 1864) [Jakovlev et al. 2006]
Leia fascipennis Meigen, 1818 [Hackman 1980]
Leia longiseta Barendrecht, 1938 [Salmela et al. 2014]
Leia picta Meigen, 1818 [Hackman 1980]
Leia subfasciata (Meigen, 1818) [Hackman 1980]
Leia winthemii Lehmann, 1822 [Hackman 1980]
RONDANIELLA Johannsen, 1909
Rondaniella dimidiata (Meigen, 1804) [Hackman 1980]
MANOTINAE Edwards, 1925
MANOTA Williston, 1896
Manota unifurcata Lundström, 1913 [Jakovlev and Penttinen 2007]
MYCETOPHILINAE Newman, 1834
tribe Exechiini Edwards, 1925
ALLODIA Winnertz, 1864
sg. ***Allodia*** Winnertz, 1864
Allodia anglofennica Edwards, 1921 [Hackman 1980]
Allodia confusa Zaitzev, 2003 [Polevoi 2001a]
= *A. simplex* Zaitzev, 1983 preocc.
Allodia embla Hackman, 1971 [Polevoi 2001a]
Allodia lugens (Wiedemann, 1817) [Hackman 1980]
Allodia lundstroemi Edwards, 1921 [Hackman 1980]
Allodia ornaticollis (Meigen, 1818) [Hackman 1980]
Allodia pyxidiiformis Zaitzev, 1983 [Stå hls 1986]
Allodia septentrionalis Hackman, 1971 [Hackman 1971b, 1980]
Allodia truncata Edwards, 1921 [Hackman 1980]
Allodia tuomikoskii Hackman, 1971 [Hackman 1971b, 1980]
Allodia zaitzevi Kurina, 1998 [Polevoi and Jakovlev 2004]
sg. ***Brachycampta*** Winnertz, 1864
Allodia adunca Zaitzev, 1992 [Polevoi 1995a]
Allodia alternans (Zetterstedt, 1838) [Hackman 1980]
Allodia angulata (Lundström, 1913) [Polevoi et al. 2006]
Allodia barbata (Lundström, 1909) [Hackman 1980]
Allodia bohemica Ševčík, 2004 [Salmela et al. 2014]
Allodia czernyi (Landrock, 1912) [Hackman 1980]
Allodia foliifera (Strobl, 1910)
Allodia grata (Meigen, 1830) [Hackman 1980]
Allodia huggerti Kjærandsen, 2007 [Salmela et al. 2014]
Allodia neglecta Edwards, 1925 [Hackman 1980]
Allodia penicillata (Lundström, 1912) [Hackman 1980]
Allodia pistillata (Lundström, 1911) [Hackman 1980]
Allodia protenta Laštovka & Matile, 1974
= *A. mendli* Plassmann, 1977 [Hackman 1979a, 1980]
Allodia silvatica (Landrock, 1912) [Hackman 1980]
Allodia subpistillata Ševčík, 1999 [Jakovlev et al. 2006]
ALLODIOPSIS Tuomikoski, 1966
Allodiopsis domestica (Meigen, 1830) [Hackman 1980]
Allodiopsis gracai Ševčík& Papp 2003 [Jakovlev et al. 2006]
Allodiopsis korolevi Zaitzev, 1982 [Salmela et al. 2014]
Allodiopsis pseudodomestica (Lackschewitz, 1937) [Jakovlev et al. 2006]
Allodiopsis rustica (Edwards, 1941) [Hackman 1980]

ANATELLA Winnertz, 1864
Anatella ankeli Plassmann, 1977 [Polevoi et al. 2006]
Anatella aquila Zaitzev, 1989 [Polevoi 1995a]
Anatella bremia Chandler, 1994 [Polevoi 2001a]
Anatella ciliata Winnertz, 1864 [Polevoi 1995a]
Anatella crispa Zaitzev, 1994 [Polevoi 2001a]
Anatella dampfi Landrock, 1924 [Jakovlev et al. 2006]
Anatella dentata Zaitzev, 1989 [Jakovlev et al. 2006]
Anatella emergens Caspers, 1987 [Jakovlev et al. 2006]
Anatella flavicauda Winnertz, 1864 [Hackman 1980]
Anatella flavomaculata Edwards, 1925 [Polevoi 1995a]
Anatella gibba Winnertz, 1864 [Kurina 2004]
Anatella lenis Dziedzicki, 1923 [Polevoi 1995a]
Anatella maritima Ostroverkhova, 1979 [Polevoi 1995a]
Anatella minuta (Staeger, 1840) [Hackman 1980]
Anatella novata Dziedzicki, 1923 [Hedmark 2000]
Anatella setigera Edwards, 1921 [Polevoi 1995a]
Anatella simpatica Dziedzicki, 1923 [Hackman 1980]
Anatella turi Dziedzicki, 1923 [Polevoi 1995a]
Anatella unguigera Edwards, 1921 [Jakovlev et al. 2006]
BRACHYPEZA Winnertz, 1864
sg. ***Brachypeza*** Winnertz, 1864
Brachypeza armata Winnertz, 1864 [Polevoi 2001a]
Brachypeza bisignata Winnertz, 1864 [Hackman 1980]
= *B. hilaris* Winnertz, 1864 [Hackman 1980] preocc.
sg. ***Paracordyla*** Tuomikoski, 1966
Brachypeza obscura Winnertz, 1864 [Polevoi et al. 2006]
BREVICORNU Marshall, 1896
Brevicornu arcticoides Caspers, 1985 [Jakovlev et al. 2006]
Brevicornu arcticum (Lundström, 1913) [Salmela et al. 2014]
Brevicornu auriculatum (Edwards, 1925) [Salmela et al. 2014]
Brevicornu beatum (Johannsen, 1912) [Polevoi 2001a]
Brevicornu bellum (Johannsen, 1912) [Polevoi 1995a]
Brevicornu bipartitum Laštovka & Matile, 1974 [Polevoi 2001a]
Brevicornu canescens (Zetterstedt, 1852)
= *B. griseolum* auct. nec (Zetterstedt, 1852) [Hackman 1980]
Brevicornu cognatum Ostroverkhova, 1979 [Salmela et al. 2014]
Brevicornu fasciculatum (Lackschewitz, 1937) [Jakovlev et al. 2006]
Brevicornu fennicum (Landrock, 1927) [Hackman 1980]
Brevicornu fissicauda (Lundström, 1911) [Hackman 1980]
Brevicornu foliatum (Edwards, 1925) [Polevoi 1995a]
Brevicornu fuscipenne (Staeger, 1840) [Hackman 1980]
Brevicornu glandis Laštovka & Matile, 1974 [Salmela et al. 2014]
Brevicornu griseicolle (Staeger, 1840) [Hackman 1980]
Brevicornu griseolum (Zetterstedt, 1852)
= *B. boreale* (Lundström, 1914) [Hackman 1980]
Brevicornu improvisum Zaitzev, 1992 [Polevoi 2001a]
Brevicornu kingi (Edwards, 1925) [Hackman 1980]
Brevicornu luteum (Landrock, 1925) [Hackman 1980]
Brevicornu melanderi Zaitzev, 1988 [Jakovlev et al. 2006]

Brevicornu nigrofuscum (Lundström, 1909) [Hackman 1980]
Brevicornu occidentale Zaitzev, 1988 [Polevoi et al. 2006]
Brevicornu parafennicum Zaitzev in Zaitzev & Polevoi, 1995 [Jakovlev et al. 2006]
Brevicornu proximum (Staeger, 1840) [Hackman 1980]
Brevicornu rosmellitum Chandler, 2001 [Salmela et al. 2014]
Brevicornu ruficorne (Meigen, 1838) [Hackman 1980]
Brevicornu serenum (Winnertz, 1864) [Hackman 1980]
Brevicornu sericoma (Meigen, 1830) [Hackman 1980]
Brevicornu setulosum Zaitzev, 1988 [Polevoi 1995a]
Brevicornu spathulatum (Lundström, 1911) [Salmela et al. 2014]
Brevicornu verralli (Edwards, 1925) [Salmela et al. 2014]
CORDYLA Meigen, 1803
Cordyla brevicornis (Staeger, 1840) [Hackman 1980]
Cordyla crassicornis Meigen, 1818 [Hackman 1980]
Cordyla fasciata Meigen, 1830 [Hackman 1980]
Cordyla fissa Edwards, 1925 [Hackman 1980]
Cordyla flaviceps (Staeger, 1840) [Hackman 1980]
Cordyla fusca Meigen, 1804 [Hackman 1980]
Cordyla insons Laštovka & Matile, 1974 [Jakovlev et al. 2006]
Cordyla murina Winnertz, 1864 [Hackman 1980, Polevoi 2001a, Jakovlev et al. 2006]
Cordyla nitens Winnertz, 1864 [Polevoi 2001a]
Cordyla nitidula Edwards, 1925 [Hackman 1980]
= *C. bergensis* (Barendrecht, 1938) [Hackman 1980]
Cordyla parvipalpis Edwards, 1925 [Hedmark 1998, Polevoi 1995a]
Cordyla pusilla Edwards, 1925 [Ano 1960, Hackman 1980]
= *C. sixi* (Barendrecht, 1938) [Hackman 1980, Polevoi 1995a]
Cordyla semiflava (Staeger, 1840) [Hackman 1980]
EXECHIA Winnertz, 1864
Exechia bicincta (Staeger, 1840) [Jakovlev and Penttinen 2007]
Exechia borealis Lundström, 1912 [Lundström 1912]
Exechia cincta Winnertz, 1864 [Hackman 1980]
Exechia confinis Winnertz, 1864 [Hackman 1980]
Exechia contaminata Winnertz, 1864 [Hackman 1980]
Exechia cornuta Lundström, 1914 [Hackman 1980]
Exechia dentata Lundström, 1916 [Jakovlev et al. 2006]
Exechia dizona Edwards, 1924 [Hackman 1980]
Exechia dorsalis (Staeger, 1840) [Hackman 1980]
Exechia exigua Lundström, 1909 [Hackman 1980]
Exechia festiva Winnertz, 1864 [Hackman 1980]
Exechia frigida (Boheman, 1865) [Hackman 1980]
Exechia fusca (Meigen, 1804) [Hackman 1980]
Exechia lucidula (Zetterstedt, 1838) [Hackman 1980]
Exechia lundstroemi Landrock, 1923 [Hackman 1980]
Exechia macula Chandler, 2001
= *E. maculipennis* (Stannius, 1831) preocc.[Hackman 1980]
Exechia micans Laštovka & Matile, 1974 [Salmela et al. 2014]
Exechia nigra Edwards, 1925 [Hackman 1980]
Exechia nigrofusca Lundström, 1909 [Hackman 1980]
Exechia nigroscutellata Landrock, 1912 [Karppinen 1961, Hackman 1980]
Exechia papyracea Stackelberg, 1948 [Stå hls 1990]

Exechia parva Lundström, 1909 [Hackman 1980]
Exechia parvula (Zetterstedt, 1852) [Hackman 1980]
= *E. nana* Staeger, 1840 preocc.
Exechia pectinivalva Stackelberg, 1948 [Polevoi 2001a]
Exechia pseudocincta Strobl, 1910 [Hackman 1980]
Exechia pseudofestiva Lackschewitz, 1937 [Jakovlev et al. 2006]
Exechia repanda Johannsen, 1912 [Väisänen 1982]
Exechia repandoides Caspers, 1984 [Jakovlev et al. 2006]
Exechia separata Lundström, 1912 [Hackman 1980]
Exechia seriata (Meigen, 1830) [Hackman 1980]
= *E. pallida* Stannius, 1831
Exechia similis Laštovka & Matile, 1974 [Polevoi et al. 2006]
Exechia spinigera Winnertz, 1864 [Hackman 1980] [Hackman 1980]
Exechia spinuligera Lundström, 1912 [Hackman 1980]
Exechia styriaca Strobl, 1898
= *E. sororcula* Lackschewitz, 1937 [Hackman 1980]
Exechia subfrigida Laštovka & Matile, 1974 [Polevoi et al. 2006]
Exechia unifasciata Lackschewitz, 1937 [Hackman 1980]
Exechia unimaculata (Zetterstedt, 1860) [Hackman 1980]
EXECHIOPSIS Tuomikoski, 1966
sg. ***Exechiopsis*** Tuomikoski, 1966
Exechiopsis aemula Plassmann, 1984 [Jakovlev et al. 2006]
Exechiopsis clypeata (Lundström, 1911) [Kurina 2004]
Exechiopsis distendens (Lackschewitz, 1937) [Hackman 1980]
Exechiopsis dumitrescae (Burghele-Balacesco, 1972) [Jakovlev et al. 2006]
Exechiopsis fimbriata (Lundström, 1909) [Hackman 1980]
Exechiopsis forcipata Lackschewitz, 1937) [Jakovlev et al. 2006]
Exechiopsis grassatura (Plassmann, 1978) [Jakovlev et al. 2006]
Exechiopsis hammi (Edwards, 1925) [Hackman 1963, 1980]
Exechiopsis indecisa (Walker, 1856) [Hackman 1963, 1980]
Exechiopsis ingrica Stackelberg, 1948 [Hackman 1980]
Exechiopsis intersecta (Meigen, 1818) [Hackman 1980]
Exechiopsis januarii (Lundström, 1913) [Hackman 1980]
Exechiopsis lackschewitziana (Stackelberg, 1948) [Hackman 1980]
Exechiopsis landrocki (Lundström, 1912) [Hackman 1980]
Exechiopsis ligulata (Lundström, 1913) [Hackman 1980]
Exechiopsis pseudindecisa Laštovka & Matile, 1974 [Polevoi et al. 2006]
Exechiopsis pseudopulchella (Lundström, 1912) [Hackman 1980]
Exechiopsis pulchella (Winnertz, 1864) [Hackman 1980]
Exechiopsis sagittata Laštovka & Matile, 1974 [Polevoi 1995a]
Exechiopsis subulata (Winnertz, 1864) [Hackman 1980]
sg. ***Xenexechia*** Tuomikoski, 1966
Exechiopsis crucigera (Lundström, 1909) [Hackman 1980]
Exechiopsis davatchii Matile, 1969 [Jakovlev et al. 2006]
Exechiopsis leptura (Meigen, 1830) [Hackman 1980]
Exechiopsis membranacea (Lundström, 1912) [Jakovlev et al. 2006]
Exechiopsis perspicua (Johannsen, 1912) [Karppinen 1962, Hackman 1980]
Exechiopsis pollicata (Edwards, 1925) [Hackman 1980]
Exechiopsis praedita (Plassmann, 1976) [Jakovlev et al. 2006]
Exechiopsis seducta (Plassmann, 1976) [Jakovlev et al. 2006]

Exechiopsis stylata Laštovka & Matile, 1974 [Jakovlev et al. 2006]
MYROSIA Tuomikoski, 1966
Myrosia maculosa (Meigen, 1818) [Hackman 1980]
NOTOLOPHA Tuomikoski, 1966
Notolopha brachycera (Zetterstedt, 1852) [Jakovlev et al. 2006]
= *N. tuomikoskii* Zaitzev & Maximova, 2000
Notolopha cristata (Staeger, 1840) [Hackman 1980]
Notolopha sibirica (Zaitzev & Maximova, 2000) [Jakovlev et al. 2006]
PSEUDEXECHIA Tuomikoski, 1966
Pseudexechia aurivernica Chandler, 1978 [Polevoi 1995a]
Pseudexechia canalicula (Johannsen, 1912) [Jakovlev et al. 2006]
Pseudexechia parallela (Edwards, 1925) [Salmela et al. 2014]
Pseudexechia pectinacea (Ostroverkhova, 1979) [Kjæ randsen 2009]
Pseudexechia tristriata Stackelberg, 1969 [Jakovlev et al. 2006]
Pseudexechia trivittata (Staeger, 1840) [Hackman 1980]
PSEUDOBRACHYPEZA Tuomikoski, 1966
Pseudobrachypeza helvetica (Walker, 1856) [Hac 1962, Hackman 1980]
PSEUDORYMOSIA Tuomikoski, 1966
Pseudorymosia fovea (Dziedzicki, 1910) [Jakovlev et al. 2006]
= *P. optiva* misid. [Hackman 1971a, 1980]
RYMOSIA Winnertz, 1864
Rymosia acta Dziedzicki, 1910 [Jakovlev et al. 2006]
Rymosia affinis Winnertz, 1864 [Hackman 1980]
= *R. gracilipes* Dziedzicki, 1910
Rymosia batava (Barendrecht, 1938) [Jakovlev et al. 2006]
Rymosia bifida Edwards, 1925 [Hackman 1980]
Rymosia britteni Edwards, 1925 [Hackman 1980]
Rymosia connexa Winnertz, 1864 [Hackman 1980]
Rymosia fasciata (Meigen, 1804) [Hackman 1980]
Rymosia fraudatrix Dziedzicki, 1910 [Hackman 1980]
? = *R. spiniforceps* Matile, 1963
Rymosia guttata Lundström, 1912 [Hackman 1980]
Rymosia pinnata Ostroverkhova, 1979 [Polevoi et al. 2006]
Rymosia placida Winnertz, 1864 [Hackman 1980]
Rymosia setiger Dziedzicki, 1910 [Hackman 1980]
Rymosia signatipes (van der Wulp, 1859)
= *R. winnertzi* Barendrecht, 1938 [Hackman 1980]
STIGMATOMERIA Tuomikoski, 1966
Stigmatomeria crassicornis (Stannius, 1831) [Hackman 1980]
SYNPLASTA Skuse, 1890
= ***Gymnogonia*** Tuomikoski, 1966
Synplasta bayardi Matile, 1971 [Salmela et al. 2014]
Synplasta dulcia (Dziedzicki, 1910) [Jakovlev et al. 2006]
Synplasta exclusa (Dziedzicki, 1910)
= *S. sintenisi* (Lackschewitz, 1937) [Hackman 1980]
Synplasta gracilis Winnertz, 1864
= *S. excogitata* auct. nec (Dziedzicki, 1910) [Hackman 1980]
Synplasta ingeniosa (Kidd 1969) [Jakovlev et al. 2006]
Synplasta praeformida (Dziedzicki, 1910) [Hackman 1980]
Synplasta pseudingeniosa Zaitzev, 1993 [Salmela et al. 2014]

Synplasta rufilatera (Edwards, 1941) [Hackman 1980]
TARNANIA Tuomikoski, 1966
Tarnania fenestralis (Meigen, 1838) [Hackman 1980]
Tarnania tarnanii (Dziedzicki, 1910) [Hackman 1980]
tribe Mycetophilini Newman, 1834
DYNATOSOMA Winnertz, 1864
Dynatosoma cochleare Strobl, 1895 [Hackman 1980]
Dynatosoma dihaeta Polevoi, 1995 [Polevoi 2001a]
= *D. schachti* Plassman, 1999
Dynatosoma fuscicorne (Meigen, 1818) [Hackman 1980]
Dynatosoma majus Landrock, 1912 [Hackman 1980]
Dynatosoma nigromaculatum Lundström, 1913 [Hackman 1980]
= *D. abdominale* auct. nec (Staeger, 1840)
Dynatosoma nobile Loew, 1873 [Hackman 1980]
Dynatosoma reciprocum (Walker, 1848) [Hackman 1980]
Dynatosoma rufescens (Zetterstedt, 1838) [Hackman 1980]
= *D. bukowskii* Zaitzev, 1986
= *D. lutescens* (Zetterstedt, 1852)
Dynatosoma silesiacum Ševčík, 2001 [Jakovlev et al. 2006]
Dynatosoma thoracicum (Zetterstedt, 1838) [Hackman 1980]
= *D. norwegiense* Zaitzev & Økland, 1994 [Polevoi 2001a]
EPICYPTA Winnertz, 1864
Epicypta aterrima (Zetterstedt, 1852) [Hackman 1980]
Epicypta fumigata (Dziedzicki, 1923) [Jakovlev et al. 2006]
Epicypta limnophila Chandler, 1981 [Salmela et al. 2014]
Epicypta scatophora (Perris, 1849) [Jakovlev et al. 2006]
MYCETOPHILA Meigen, 1803
= ***Fungivora*** Meigen, 1800 suppr.
Mycetophila abbreviata Landrock, 1914 [Hackman 1980]
Mycetophila abiecta (Laštovka, 1963) [Polevoi 2001a]
Mycetophila adumbrata Mik, 1884 [Hackman 1980]
Mycetophila alea Laffoon, 1965 [Hackman 1980]
= *M. guttata* Dziedzicki, 1884
Mycetophila attonsa Laffoon, 1957 [Hackman 1980]
= *M. uncta* Plassmann, 1999
Mycetophila autumnalis Lundström, 1909 [Hackman 1980]
Mycetophila bialorussica Dziedzicki, 1884 [Hackman 1980]
? *Mycetophila biformis* Maximova, 2002 [Salmela et al. 2014]
Mycetophila biusta Meigen, 1818 [Hackman 1980]
Mycetophila blanda Winnertz, 1864 [Hackman 1980]
Mycetophila bohemica (Laštovka, 1963) [Hackman 1980]
Mycetophila boreocruciator Ševčík, 2003 [Salmela et al. 2014]
Mycetophila brevitarsata (Laštovka, 1963) [Laštovka 1963, Polevoi 1995a]
Mycetophila caudata Staeger, 1840 [Hackman 1980]
Mycetophila cingulum Meigen, 1830 [Salmela et al. 2014]
Mycetophila confluens Dziedzicki, 1884 [Hackman 1980]
= *M. fulva* Winnertz, 1864
Mycetophila confusa Dziedzicki, 1884
= *M. affluctata* Edwards, 1941 [Hackman 1980]
Mycetophila curviseta Lundström, 1911 [Polevoi 2001a]

Mycetophila deflexa Chandler, 2001 [Polevoi and Jakovlev 2004]
Mycetophila dentata Lundström, 1915 [Hackman 1980]
Mycetophila devioides Bechev, 1988 [Salmela et al. 2014]
Mycetophila distigma Meigen, 1830 [Salmela et al. 2014]
= *M. w-fuscum* Dziedzicki, 1884
Mycetophila dziedzickii Chandler, 1977 [Hackman 1980]
= *M. obscura* auct. nec Walker, 1848
Mycetophila edwardsi Lundström, 1913
Mycetophila estonica Kurina, 1992 [Polevoi 1995a]
Mycetophila evanida Laštovka, 1972 [Polevoi 2001a]
Mycetophila exstincta Loew, 1869 [Jakovlev et al. 2006]
= *M. mikii* Dziedzicki, 1884
Mycetophila finlandica Edwards, 1913 [Hackman 1980]
Mycetophila flava Winnertz, 1864 [Hackman 1980]
Mycetophila forcipata Lundström, 1913 [Jakovlev and Penttinen 2007]
Mycetophila formosa Lundström, 1911 [Hackman 1980]
Mycetophila freyii Lundström, 1909 [Hackman 1980]
Mycetophila fungorum (De Geer, 1776) [Hackman 1980]
Mycetophila gibbula Edwards, 1925 [Jakovlev et al. 2006]
Mycetophila hetschkoi Landrock, 1918 [Hackman 1980]
Mycetophila ichneumonea Say, 1823 [Meinander 1977, Hackman 1980]
Mycetophila idonea Laštovka, 1972 [Jakovlev et al. 2006]
Mycetophila immaculata (Dziedzicki, 1884) [Hackman 1980]
Mycetophila laeta Walker, 1848
= *M. calva* Lundström, 1912 [Hackman 1980]
Mycetophila lapponica Lundström, 1906 [Hackman 1980]
Mycetophila lobulata Zaitzev, 1999 [Salmela et al. 2014]
Mycetophila lubomirskii Dziedzicki, 1884 [Hackman 1980]
Mycetophila luctuosa Meigen, 1830 [Hackman 1980]
= *M. modesta* Winnertz, 1864
Mycetophila magnicauda Strobl, 1895 [Hackman 1980]
Mycetophila marginata Winnertz, 1864 [Hackman 1980]
Mycetophila mitis (Johannsen, 1912) [Hackman 1980]
Mycetophila mohilevensis Dziedzicki, 1884 [Hackman 1980]
Mycetophila monstera Maximova, 2002 [Salmela et al. 2014]
Mycetophila morata Zaitzev, 1999 [Polevoi et al. 2006]
Mycetophila moravica Landrock, 1925 [Hackman 1980]
Mycetophila morosa Winnertz, 1864 [Hackman 1980]
Mycetophila nigrofusca Dziedzicki, 1884 [Jakovlev et al. 2006]
Mycetophila occultans Lundström, 1913 [Hackman 1980]
= *M. tarsata* Winnertz, 1864 preocc.[Hackman 1980]
Mycetophila ocellus Walker, 1848 [Hackman 1980]
Mycetophila ornata Stephens, 1846 [Hackman 1980]
Mycetophila ostentanea Zaitzev, 1998 [Polevoi et al. 2006]
Mycetophila pecinai (Laštovka, 1963) [Polevoi et al. 2006]
Mycetophila perpallida Chandler, 1993 [Chandler 1993]
= *M. bimaculata* auct. nec Meigen, 1804
Mycetophila pictula Meigen, 1830 [Hackman 1980]
Mycetophila pseudoforcipata Zaitzev, 1998 [Kurina 2004]
Mycetophila pumila Winnertz, 1864 [Hackman 1980]

Mycetophila pyrenaica Matile, 1967 [Salmela et al. 2014]
Mycetophila quadra Lundström, 1909 [Hackman 1980]
Mycetophila ruficollis Meigen, 1818 [Jakovlev et al. 2006]
Mycetophila schnabli (Dziedzicki, 1884) [Hackman 1980]
Mycetophila sigmoides Loew, 1869 [Salmela et al. 2014]
Mycetophila signata Meigen, 1830 [Hackman 1980]
Mycetophila signatoides Dziedzicki, 1884 [Hackman 1980]
= *M. assimilis* Matile, 1967 [Polevoi 1995a]
Mycetophila sinuosa Plassmann & Schacht, 1999 [Salmela et al. 2014]
Mycetophila sordida van der Wulp, 1874 [Hackman 1980]
Mycetophila stolida Walker, 1856 [Jakovlev et al. 2006]
Mycetophila stricklandi (Laffoon, 1957) [Polevoi 1995a]
Mycetophila strigata Staeger, 1840 [Hackman 1980]
Mycetophila strigatoides Landrock, 1927 [Stå hls 1990]
Mycetophila strobli Laštovka, 1972 [Meinander 1977, Hackman 1980]
Mycetophila stylata (Dziedzicki, 1884) [Hackman 1980]
Mycetophila sublunata Zaitzev, 1998 [Kurina 2004]
Mycetophila subsigillata Zaitzev, 1999 [Jakovlev et al. 2006]
Mycetophila sumavica (Laštovka, 1963) [Jakovlev and Penttinen 2007]
Mycetophila triangularis Lundström, 1912 [Jakovlev et al. 2006]
Mycetophila trinotata Staeger, 1840 [Hackman 1980]
Mycetophila uliginosa Chandler, 1988 [Salmela et al. 2014]
Mycetophila unguiculata Lundström, 1913 [Jakovlev et al. 2006]
Mycetophila uninotata Zetterstedt, 1852 [Meinander 1977, Hackman 1980]
Mycetophila unipunctata Meigen, 1818 [Hackman 1980]
Mycetophila xanthopyga Winnertz, 1864 [Jakovlev et al. 2006]
Mycetophila zetterstedtii Lundström, 1906 [Hackman 1980]
PHRONIA Winnertz, 1864
Phronia aviculata Lundström, 1914 [Hackman 1970a, 1980]
Phronia avidoides Jakovlev & Polevoi, 2009 [Jakovlev and Polevoi 2009]
Phronia biarcuata (Becker, 1908) [Hackman 1980]
= *P. johannae* Steenberg, 1924 [Hackman 1970a]
= *P. praecox* Edwards, 1925
Phronia bicolor Dziedzicki, 1889 [Hackman 1980]
= *P. fusciventris* van Duzee, 1928
Phronia borealis Hackman, 1970 [Hackman 1970a, 1980]
Phronia braueri Dziedzicki, 1889 [Hackman 1970a, 1980]
= *P. annulata* Winnertz, 1864
Phronia caliginosa Dziedzicki, 1889 [Hackman 1980]
= *P. trivittata* Dziedzicki, 1889
Phronia cinerascens Winnertz, 1864 [Hackman 1980]
Phronia conformis (Walker, 1856) [Hackman 1980]
= *P. girshneri* Dziedzicki, 1889
Phronia cordata Lundström, 1914 [Hackman 1970a, 1980]
Phronia coritanica Chandler, 1992 [Kurina 2004]
Phronia cornuta Lundström, 1914 [Hackman 1970a, 1980]
Phronia crassitarsus Hackman, 1970 [Hackman 1970a, 1980, Chandler 1992a]
Phronia digitata Hackman, 1970 [Hackman 1970a, 1980]
Phronia disgrega Dziedzicki, 1889 [Hackman 1970a, 1980]
Phronia distincta Hackman, 1970 [Hackman 1970a, 1980]

Phronia dziedzickii Lundström, 1906 [Hackman 1980]
Phronia egregia Dziedzicki, 1889 [Hackman 1980]
Phronia electa Dziedzicki, 1889 [Hac 1964, Hackman 1980]
Phronia elegans Dziedzicki, 1889 [Hackman 1970a, 1980]
Phronia elegantula Hackman, 1970 [Hackman 1970a, 1980]
Phronia exigua (Zetterstedt, 1852) [Hackman 1980]
Phronia fennica Jakovlev & Polevoi, 2009 [Jakovlev and Polevoi 2009]
Phronia flavipes Winnertz, 1864 [Hackman 1980]
Phronia forcipata Winnertz, 1864 [Hackman 1980]
Phronia forcipula Winnertz, 1864 [Hackman 1980]
Phronia gagnei Chandler, 1992 [Polevoi 1995a]
Phronia gracilis Hackman, 1970 [Hackman 1970a, 1980]
Phronia humeralis Winnertz, 1864 [Hac 1967, Hackman 1980]
Phronia interstincta Dziedzicki, 1889 [Hackman 1980]
Phronia longelamellata Strobl, 1898
 = *P. minuta* Landrock, 1928 [Hac 1964, Hackman 1980]
Phronia lutescens Hackman, 1970 [Hackman 1970a, 1980]
Phronia maculata Dziedzicki, 1889 [Hackman 1980]
Phronia mutabilis Dziedzicki, 1889 [Hackman 1980]
Phronia mutila Lundström, 1911 [Salmela et al. 2014]
Phronia nigricornis (Zetterstedt, 1852) [Hackman 1980]
 = *P. dubia* Dziedzicki, 1889
Phronia nigripalpis Lundström, 1909 [Hackman 1980]
Phronia nitidiventris (van der Wulp, 1859) [Hackman 1980]
 = *P. squalida* Winnertz, 1864
 = *P. vitiosa* Winnertz, 1864
Phronia notata Dziedzicki, 1889 [Hackman 1980]
Phronia obscura Dziedzicki, 1889 [Hackman 1970a, 1980]
Phronia obtusa Winnertz, 1864 [Hackman 1980]
Phronia peculiaris Dziedzicki, 1889 [Hac 1964, Hackman 1980]
Phronia persimilis Hackman, 1970 [Hackman 1970a, 1980]
Phronia petulans Dziedzicki, 1889 [Hackman 1970a, 1980]
Phronia portschinskyi Dziedzicki, 1889 [Hackman 1970a, 1980]
Phronia siebeckii Dziedzicki, 1889 [Hackman 1980]
Phronia signata Winnertz, 1864 [Hackman 1980]
 = *P. austriaca* Winnertz, 1864 [Hackman 1980] nom. dubium
Phronia spinigera Hackman, 1970 [Hackman 1970a, 1980]
Phronia strenua Winnertz, 1864 [Hackman 1980]
 = *P. flavicollis* Winnertz, 1864 nom. dubium
Phronia subsilvatica Hackman, 1970 [Hackman 1970a, 1980]
Phronia sudetica Dziedzicki, 1889 [Hac 1964, Hackman 1980]
Phronia sylvatica Dziedzicki, 1889 [Hac 1964, Hackman 1980]
Phronia taczanowskyi Dziedzicki, 1889 [Hackman 1980]
Phronia tenuis Winnertz, 1864 [Hackman 1970a, 1980]
Phronia tiefii Dziedzicki, 1889 [Hackman 1980]
 = *P. marginata* Dziedzicki, 1889
Phronia triangularis Winnertz, 1864 [Hackman 1980]
Phronia unica Dziedzicki, 1889 [Hackman 1980]
Phronia vitrea Plassmann, 1999
 = *P. carli* Chandler, 2001 [Chandler 2001a]

Phronia willistoni Dziedzicki, 1889 [Hackman 1980]
PLATUROCYPTA Enderlein, 1910
Platurocypta punctum (Stannius, 1831) [Hackman 1980]
Platurocypta testata (Edwards, 1925) [Hackman 1980]
SCEPTONIA Winnertz, 1864
Sceptonia concolor Winnertz, 1864 [Hackman 1980]
Sceptonia costata (van der Wulp, 1859) [Polevoi 2001a]
Sceptonia demeijerei Bechev, 1997 [Jakovlev et al. 2006]
Sceptonia flavipuncta Edwards, 1925 [Salmela et al. 2014]
Sceptonia fumipes Edwards, 1925 [Polevoi 2001a]
Sceptonia fuscipalpis Edwards, 1925 [Polevoi 2001a]
Sceptonia hamata Ševčík, 2004 [Jakovlev et al. 2006]
Sceptonia longisetosa Ševčík, 2004 [Jakovlev et al. 2006]
Sceptonia membranacea Edwards, 1925 [Polevoi 2001a]
Sceptonia nigra (Meigen, 1804) [Hackman 1980]
Sceptonia pughi Chandler, 1991 [Jakovlev et al. 2006]
Sceptonia regni Chandler, 1991 [Jakovlev et al. 2006]
Sceptonia tenuis Edwards, 1925
Sceptonia thaya Ševčík, 2004 [Jakovlev et al. 2006]
TRICHONTA Winnertz, 1864
Trichonta apicalis Strobl, 1898 [Polevoi et al. 2006]
Trichonta atricauda (Zetterstedt, 1852) [Hackman 1980]
= *T. adunca* Edwards, 1925 [Hackman 1980]
Trichonta beata Gagné, 1981 [Hackman 1980, Gagné 1981]
Trichonta bicolor Landrock, 1912 [Hackman 1980]
Trichonta bifida Lundström, 1909 [Hackman 1980]
Trichonta brevicauda Lundström, 1909 [Hackman 1980]
Trichonta canora Gagné, 1981 [Hackman 1980, Gagné 1981]
Trichonta clara Gagné, 1981 [Hackman 1980, Gagné 1981]
Trichonta comica Gagné, 1981 [Hackman 1980, Gagné 1981]
Trichonta comis Gagné, 1981 [Hackman 1980, Gagné 1981]
Trichonta concinna Gagné, 1981 [Hackman 1980, Gagné 1981]
Trichonta conjungens Lundström, 1909 [Hackman 1980]
Trichonta delicata Gagné, 1981 [Hackman 1980, Gagné 1981]
Trichonta eximia Gagné, 1981 [Polevoi et al. 2006]
Trichonta facilis Gagné, 1981 [Hackman 1980, Gagné 1981]
Trichonta falcata Lundström, 1911 [Hackman 1980]
Trichonta fissicauda (Zetterstedt, 1852) [Hackman 1980]
= *T. claripennis* Lundström, 1914
Trichonta flavicauda Lundström, 1914 [Hackman 1980]
Trichonta fragilis Gagné, 1981 [Hackman 1980, Gagné 1981]
Trichonta fusca Landrock, 1918 [Hackman 1980]
Trichonta generosa Gagné, 1981 [Salmela et al. 2014]
Trichonta girschneri Landrock, 1912 [Hackman 1980]
Trichonta hamata Mik, 1880 [Hackman 1980]
Trichonta icenica Edwards, 1925 [Hackman 1980]
Trichonta melanura (Staeger, 1840) [Hackman 1980]
Trichonta nigritula Edwards, 1925 [Polevoi et al. 2006]
Trichonta palustris Maximova, 2002 [Salmela et al. 2014]
Trichonta patens Johannsen, 1912 [Hackman 1980]

Trichonta perspicua van der Wulp, 1881
= *T. mediastinalis* (Lundström, 1909) [Hackman 1980]
Trichonta subfusca Lundström, 1909 [Hackman 1980]
Trichonta submaculata (Staeger, 1840) [Hackman 1980]
Trichonta subterminalis Zaitzev & Menzel, 1996 [Polevoi et al. 2006]
Trichonta terminalis (Walker, 1856) [Hackman 1980]
Trichonta tristis (Strobl, 1898) [Jakovlev and Penttinen 2007]
Trichonta trivittata Lundström, 1916 [Hackman 1980]
Trichonta venosa (Staeger, 1840) [Hackman 1980]
Trichonta vitta (Meigen, 1830) [Hackman 1980]
Trichonta vulcani (Dziedzicki, 1889) [Hackman 1980]
= *T. appropinquata* (Strobl, 1900) [Hackman 1980]
? = *T. trifida* Lundström, 1909
Trichonta vulgaris Loew, 1869 [Hackman 1980]
= *T. nigricauda* Lundström, 1906
ZYGOMYIA Winnertz, 1864
Zygomyia angusta Plassmann, 1977 [Jakovlev et al. 2006]
Zygomyia humeralis (Wiedemann, 1817) [Hackman 1980]
Zygomyia kiddi Chandler, 1991 [Polevoi 2001a]
Zygomyia notata (Stannius, 1831) [Jakovlev et al. 2006]
Zygomyia pictipennis (Staeger, 1840) [Hackman 1980]
Zygomyia pseudohumeralis Caspers, 1980 [Polevoi 1995a]
Zygomyia semifusca (Meigen, 1818) [Hackman 1980]
Zygomyia valida Winnertz, 1864 [Hackman 1980]
Zygomyia vara (Staeger, 1840) [Hackman 1980]
Zygomyia zaitzevi Chandler, 1991 [Jakovlev et al. 2006]

Excluded species

Allodia triangularis (Strobl, 1895) [Hackman 1980]
Exechiopsis unguiculata (Lundström, 1911) mistake [Hackman 1980]
Leptomorphus quadrimaculatus Matsumura, 1916 [Polevoi 1995a, Zaitzev and Ševčík 2002]
Mycetophila gratiosa Winnertz, 1864 [Hackman 1980]
Mycetophila lineola Mik, 1898 [Meinander 1977]
Mycetophila lunata Meigen, 1804 [Hackman 1980, Polevoi 1995a]
Mycetophila sigillata Dziedzicki, 1884
Mycetophila vittipes Zetterstedt, 1852 [Hackman 1980]
Palaeodocosia alpicola (Strobl, 1895) [Hackman 1980]
Phronia austriaca Winnertz, 1864 [Hackman 1980]
Pseudexechia trisignata (Edwards, 1913) [Hackman 1980]
Pseudorymosia optiva (Dziedzicki, 1910) [Hackman 1971a, 1980]
Sciophila hebes Johannsen, 1910 [Komonen 2001, Polevoi and Jakovlev 2004]
Synplasta excogitata (Dziedzicki, 1910) [Hackman 1980]
Trichonta aberrans Lundström, 1911 mistake
Trichonta fidelis Gagné, 1981 mistake [Hackman 1980, 1982]

SCIARIDAE Billberg, 1820

Supporting references for *Vilkamaa P (2014) Checklist of the family Sciaridae (Diptera) of Finland. ZooKeys @@: @-@. doi: 10.3897/zookeys.??.7381*

BAEOSCIARA Tuomikoski, 1960
Baeosciara discolor (Lengersdorf, 1928)
= *B. pusillima* (Frey, 1942) [Hackman 1980]
Baeosciara scotica (Edwards, 1925) [Valkeila 1959, Hackman 1980]
Baeosciara sinuata Menzel & Mohrig, 1997 [Vilkamaa and Komonen 2001]
BRADYSIA Winnertz, 1867
Bradysia affinis (Zetterstedt, 1838) [Hackman 1980]
= *B. pratincola* Tuomikoski, 1960 [Hackman 1980]
Bradysia albanensis (Lengersdorf, 1926) [Hackman 1980]
Bradysia alpicola (Winnertz, 1867)
= *B. mutabilis* (Lengersdorf, 1926) [Hackman 1980]
= *B. morio* auct. nec (Fabricius, 1794) [Hackman 1980]
Bradysia angustata Tuomikoski, 1960 [Hackman 1980]
Bradysia angustostylata Menzel, 2005 [Vilkamaa et al. 2013a]
Bradysia aprica (Winnertz, 1867) [Hackman 1980]
Bradysia arcana Menzel & Mohrig, 1998
= *B. fenestralis* auct. nec (Zetterstedt, 1838) [Hackman 1980]
Bradysia arcula Vilkamaa, Salmela & Hippa, 2007 [Vilkamaa et al. 2007]
Bradysia ascenda Rudzinski, 1994 [Vilkamaa et al. 2013a]
Bradysia bicolor (Meigen, 1818) [Hackman 1980]
Bradysia bispinifera Mohrig & Krivosheina, 1983 [Vilkamaa et al. 2013a]
Bradysia brevispina Tuomikoski, 1960 [Hackman 1980]
Bradysia browni (Shaw, 1935)
= *B. diversiabdominalis* (Lengersdorf, 1941)
= *B. laurencei* Menzel & Mohrig, 2000
= *B. betuleti* misid. [Tuomikoski 1960a]
Bradysia cinerascens (Grzegorzek, 1884)
= *B. lanicauda* Tuomikoski, 1960 [Hackman 1980]
Bradysia confinis (Winnertz, 1867) [Hackman 1980]
Bradysia excelsa Menzel & Mohrig, 1998 [Vilkamaa et al. 2007]
Bradysia flavipila Tuomikoski, 1960 [Hackman 1980]
Bradysia forcipulata (Lundbeck, 1898) [Hackman 1980]
Bradysia forficulata (Bezzi, 1914)
= *B. nocturna* Tuomikoski, 1960 [Hackman 1980]
Bradysia fungicola (Winnertz, 1867) [Tuomikoski 1960b]
Bradysia giraudii (Egger, 1862) [Hackman 1980]
Bradysia globulifera (Lengersdorf, 1934) [Hackman 1980]
Bradysia hilariformis Tuomikoski, 1960 [Hackman 1980]
Bradysia hilaris (Winnertz, 1867) [Hackman 1980]
Bradysia holsatica Heller, 2004 [Vilkamaa et al. 2013a]
Bradysia hortensis Heller, 2000 [Vilkamaa et al. 2013a]
Bradysia impatiens (Johannsen, 1912)
= *B. difformis* Frey, 1948 [Frey 1948]
= *B. paupera* Tuomikoski, 1960 [Hackman 1980]
Bradysia inusitata Tuomikoski, 1960 [Hackman 1980]

Bradysia iridipennis (Zetterstedt, 1838) [Hackman 1980]
Bradysia lapponica (Lengersdorf, 1926) [Hackman 1980]
Bradysia latiterga Tuomikoski, 1960 [Hackman 1980]
Bradysia leptoptera Tuomikoski, 1960 [Hackman 1980]
Bradysia lilienthalae Mohrig & Menzel, 1990 [Vilkamaa et al. 2013a]
Bradysia lobulifera Frey, 1948 [Hackman 1980]
Bradysia longicauda Mohrig & Menzel, 1990 [Vilkamaa et al. 2013a]
Bradysia longicubitalis (Lengersdorf, 1924)
= *B. cinereovittata* Frey, 1948 [Hackman 1980]
Bradysia minima Mohrig & Mamaev, 1989 [Vilkamaa et al. 2013a]
Bradysia moesta Frey, 1948 [Hackman 1980]
= *B. albosetosa* Frey, 1948 [Hackman 1980, Mohrig and Menzel 1993]
Bradysia moestula Tuomikoski, 1960 [Hackman 1980, Mohrig and Menzel 1993, Heller et al. 2009]
Bradysia nervosa (Meigen, 1818) [Hackman 1980]
Bradysia nitidicollis (Meigen, 1818) [Hackman 1980]
= *B. atroparva* Frey, 1948 [Hackman 1980]
Bradysia normalis Frey, 1948 [Hackman 1980]
Bradysia ocellaris (Comstock, 1882) [Hackman 1980]
Bradysia pallipes (Fabricius, 1787)
= *B. brunnipes* (Meigen, 1804) [Hackman 1980]
= *B. picipes* (Zetterstedt, 1838) [Hackman 1980, Mohrig and Menzel 1993]
Bradysia pauperata (Winnertz, 1867) []
Bradysia peraffinis Tuomikoski, 1960 [Hackman 1980]
Bradysia pilistriata Frey, 1948 [Hackman 1980]
Bradysia placida (Winnertz, 1867)
= *B. fimbricauda* Tuomikoski, 1960 [Hackman 1980]
Bradysia polonica (Lengersdorf, 1929) [Hackman 1980]
Bradysia praecox (Meigen, 1818) [Hackman 1980]
Bradysia rectinervis Frey, 1948 [Hackman 1980]
Bradysia reflexa Tuomikoski, 1960 [Hackman 1980]
Bradysia regularis (Lengersdorf, 1934)
= *B. subnervosa* Frey, 1948 [Hackman 1980]
Bradysia rufescens (Zetterstedt, 1852) [Hackman 1980]
Bradysia scabricornis Tuomikoski, 1960 [Hackman 1980]
= *B. subscabricornis* Mohrig & Menzel, 199 [Salmela and Vilkamaa 2005]
Bradysia siberica Komarova, 2001 []
Bradysia spinostyla Mohrig & Menzel, 1990 [Vilkamaa et al. 2013a]
Bradysia strigata (Staeger, 1840) [Hackman 1980]
Bradysia subalpina Frey, 1948 [Hackman 1980]
Bradysia subamoena Mohrig & Krivosheina, 1989 [Vilkamaa et al. 2013a]
Bradysia submoesta Mohrig & Krivosheina, 1989 [Salmela and Vilkamaa 2005]
Bradysia tilicola (Loew, 1850)
= *B. amoena* (Winnertz, 1867) [Hackman 1980]
= *B. cellarum* Frey, 1948 [Hackman 1980]
Bradysia trivittata (Staeger, 1840) [Hackman 1980]
Bradysia vagans (Winnertz, 1868)
= *B. callicera* Frey, 1948 [Hackman 1980]
Bradysia vernalis (Zetterstedt, 1851) [Hackman 1980]
Bradysia zonata Rudzinski, 1993 [Vilkamaa et al. 2013a]

BRADYSIOPSIS Tuomikoski, 1960
Bradysiopsis vittata (Meigen, 1830)
= *B. leucotricha* (Tuomikoski, 1960) [Hackman 1980]
Bradysiopsis vittigera (Zetterstedt, 1851) [Hackman 1980]
CAMPTOCHAETA Hippa & Vilkamaa, 1994
Camptochaeta austriaca Heller, 2012 [Vilkamaa et al. 2013a]
Camptochaeta bournei (Shaw, 1941) [Haarto 2009]
Camptochaeta camptochaeta (Tuomikoski, 1960) [Hackman 1980]
Camptochaeta consimilis (Holmgren, 1869) [Hippa and Vilkamaa 1994]
Camptochaeta delicata (Lengersdorf, 1935) [Hippa and Vilkamaa 1994]
Camptochaeta duplicata Hippa & Vilkamaa, 1994 [Hippa and Vilkamaa 1994]
Camptochaeta fallax Hippa & Vilkamaa, 1994 [Vilkamaa et al. 2007]
Camptochaeta hirtula (Lengersdorf, 1934)
= *C. fulvicollis* (Tuomikoski, 1960) [Hackman 1980]
Camptochaeta propria Hippa & Vilkamaa, 1994 [Hippa and Vilkamaa 1994]
Camptochaeta scanica Hippa & Vilkamaa, 1994 [Hippa and Vilkamaa 1994]
Camptochaeta sicilicula Hippa & Vilkamaa, 1994 [Hippa and Vilkamaa 1994]
Camptochaeta simulator Hippa & Vilkamaa, 1994 [Hippa and Vilkamaa 1994]
Camptochaeta stammeri (Lengersdorf, 1940) [Hackman 1980]
Camptochaeta tenuipalpalis (Mohrig & Antonova, 1978) [Hippa and Vilkamaa 1994]
Camptochaeta uniformis (Mohrig & Menzel, 1990) [Hippa and Vilkamaa 1994]
Camptochaeta vivax (Frey, 1948) [Hackman 1980]
Camptochaeta xystica Hippa & Vilkamaa, 1994 [Hippa and Vilkamaa 1994]
CHAETOSCIARA Frey, 1942
Chaetosciara estlandica (Lengersdorf, 1929) [Hackman 1980]
CLAUSTROPYGA Hippa, Vilkamaa & Mohrig, 2003
Claustropyga acanthostyla (Tuomikoski, 1960) [Hackman 1980]
Claustropyga brevichaeta (Mohrig & Antonova, 1978) [Vilkamaa et al. 2013a]
Claustropyga clausa (Tuomikoski, 1960) [Hackman 1980]
Claustropyga corticis (Mohrig & Antonova, 1978) [Hippa et al. 2003]
Claustropyga ctenophora Hippa, Vilkamaa & Mohrig, 2003 [Vilkamaa et al. 2013a]
Claustropyga heteroclausa (Rudzinski, 1991) [Hippa et al. 2003]
Claustropyga refrigerata (Lengersdorf, 1930) [Hackman 1980]
Claustropyga subcorticis (Mohrig & Krivosheina, 1985) [Hippa et al. 2003]
CORYNOPTERA Winnertz, 1867
Corynoptera barbata Tuomikoski, 1960 [Hackman 1980]
Corynoptera bicuspidata (Lengersdorf, 1926)
= *C. gymnops* Tuomikoski, 1960 [Hackman 1980]
Corynoptera bipartita Mohrig & Krivosheina, 1985 [Hippa et al. 2010]
Corynoptera blanda (Winnertz, 1867) [Hackman 1980]
Corynoptera boletiphaga (Lengersdorf, 1940) [Hackman 1980]
= *C. geogenia* Tuomikoski, 1960 [Hackman 1980, Gerbachevskaja-Pavluchenko 1986]
Corynoptera breviformis Mohrig & Krivosheina, 1983 [Hippa et al. 2010]
Corynoptera cracentis Vilkamaa, Hippa & Heller, 2013 [Vilkamaa et al. 2013b]
Corynoptera cuniculata (Lengersdorf, 1942)
= *C. caldariorum* Tuomikoski, 1960 [Hackman 1980]
Corynoptera defecta (Frey, 1948)
Corynoptera dentata (Bukowski & Lengersdorf, 1936) [Vilkamaa et al. 2013a]
Corynoptera deserta Heller & Menzel, 2006
= *C. minutula* (Bukowski & Lengersdorf, 1936) [Hippa and Vilkamaa 1994]

Corynoptera dubitata Tuomikoski, 1960 [Hackman 1980]
Corynoptera fera Mohrig & Heller, 1992 [Salmela and Vilkamaa 2005]
Corynoptera flavicauda (Zetterstedt, 1855) [Hackman 1980]
Corynoptera forcipata (Winnertz, 1867) [Hackman 1980]
Corynoptera furcifera Mohrig & Mamaev, 1987 [Salmela and Vilkamaa 2005]
Corynoptera globiformis (Frey, 1945) []
Corynoptera hypopygialis (Lengersdorf, 1926)
= *C. piniphila* (Lengersdorf, 1940) [Hackman 1980]
Corynoptera inari Vilkamaa, Hippa & Heller, 2013 [Vilkamaa et al. 2013b]
Corynoptera inexspectata Tuomikoski, 1960 [Hackman 1980]
Corynoptera irmgardis (Lengersdorf, 1930) [Hackman 1980]
Corynoptera levis Tuomikoski, 1960 [Hackman 1980]
Corynoptera luteofusca (Bukowski & Lengersdorf, 1936) [Hackman 1980]
Corynoptera marinae Mohrig & Krivosheina, 1986 [Hippa et al. 2010]
Corynoptera melanochaeta Mohrig & Menzel, 1992 [Hippa et al. 2010]
Corynoptera membranigera (Kieffer, 1903)
= *C. trispina* Tuomikoski, 1960 [Hackman 1980]
Corynoptera montana (Winnertz, 1869) [Hackman 1980]
Corynoptera ninae Hippa, Vilkamaa & Heller, 2010 [Hippa et al. 2010]
Corynoptera obscuripila Tuomikoski, 1960 [Hackman 1980]
Corynoptera parvula (Winnertz, 1867) [Hackman 1980]
= *C. uncinata* (Hippa & Vilkamaa, 1994) [Hippa and Vilkamaa 1994]
Corynoptera parvulaformis Mohrig, 1985 [Hippa and Vilkamaa 1994]
Corynoptera penna (Pettey, 1918)
= *C. alneti* Hippa, Vilkamaa & Heller, 2010 [Hippa et al. 2010]
Corynoptera perochaeta (Mohrig & Menzel, 1990) []
Corynoptera plusiochaeta Hippa, Vilkamaa & Heller, 2010 [Hippa et al. 2010]
Corynoptera polana Rudzinski, 2009 [Hippa et al. 2010]
Corynoptera postforcipata Rudzinski, 1993 [Vilkamaa et al. 2007]
Corynoptera postglobiformis Mohrig, 1993 []
Corynoptera praeforcipata Mohrig & Mamaev, 1987 [Vilkamaa et al. 2013a]
Corynoptera quantula (Hippa & Vilkamaa, 1994) [Hippa and Vilkamaa 1994]
Corynoptera saccata Tuomikoski, 1960 [Hackman 1980]
Corynoptera saetistyla Mohrig & Krivosheina, 1985 [Salmela and Vilkamaa 2005]
Corynoptera salmelai Vilkamaa, Hippa & Heller, 2013 [Vilkamaa et al. 2013b]
Corynoptera sphenoptera Tuomikoski, 1960 [Hackman 1980]
Corynoptera spiciforceps Vilkamaa, Hippa & Heller, 2013 [Vilkamaa et al. 2013b]
Corynoptera spinifera Tuomikoski, 1960 [Hackman 1980]
Corynoptera subblanda Tuomikoski, 1960 [Hackman 1980]
Corynoptera subdentata Mohrig, 1985 [Hippa and Vilkamaa 1994]
Corynoptera subparvula Tuomikoski, 1960 [Hackman 1980]
Corynoptera subsedula Mohrig & Mamaev, 1987 [Salmela and Vilkamaa 2005]
Corynoptera subtetrachaeta Komarova, 1995 [Hippa et al. 2010]
Corynoptera subtilis (Lengersdorf, 1929)
= *C. longicornis* (Bukowski & Lengersdorf, 1936) [Hackman 1980]
Corynoptera subvariegata Rudzinski, 1992 [Vilkamaa et al. 2013b]
Corynoptera tetrachaeta Tuomikoski, 1960 [Hackman 1980]
Corynoptera trepida (Winnertz, 1867)
= *C. clinochaeta* Tuomikoski, 1960 [Hackman 1980]
Corynoptera triacantha Tuomikoski, 1960 [Hackman 1980]

Corynoptera tumidula Hippa, Vilkamaa & Heller, 2010 [Hippa et al. 2010]
Corynoptera tuomikoskii Hippa, Vilkamaa & Heller, 2013 [Vilkamaa et al. 2013b]
Corynoptera unidentata (Hippa & Vilkamaa, 1994) [Hippa and Vilkamaa 1994]
Corynoptera vagula Tuomikoski, 1960 [Hackman 1980]
Corynoptera verrucifera (Lengersdorf, 1952) [Hippa and Vilkamaa 1994]
Corynoptera voluptuosa Mohrig & Mamaev, 1987 [Hippa et al. 2010]
Corynoptera waltraudis Mohrig & Mamaev, 1987 [Hippa et al. 2010]
Corynoptera winnertzi Mohrig, 1993 [Mohrig 1993]
COSMOSCIARA Frey, 1942
Cosmosciara perniciosa (Edwards, 1922) [Hackman 1980]
CRATYNA Winnertz, 1867
= ***Plastosciara*** Berg, 1899
= ***Decembrina*** Frey, 1948
= ***Dendrosciara*** Frey, 1948
sg. ***Cratyna*** Winnertz, 1867
Cratyna ambigua (Lengersdorf, 1934)
= *C. latiforceps* (Bukowski & Lengersdorf, 1936) [Hackman 1980]
Cratyna atra Winnertz, 1867
= *C. pictiventris* (Kieffer, 1898) [Hackman 1980]
Cratyna betulae (Mohrig & Mamaev, 1992) [Vilkamaa et al. 2013a]
Cratyna breviflagellata (Mohrig & Mamaev, 1985) [Vilkamaa et al. 2013a]
Cratyna monumenta Rudzinski, 2009 [Vilkamaa et al. 2013a]
Cratyna pernitida (Edwards, 1915) [Hackman 1980]
Cratyna schineri (Winnertz, 1867) [Hackman 1980]
Cratyna sicata Vilkamaa, Hippa & Heller, 2013 [Vilkamaa et al. 2013c]
Cratyna symplecta (Rudzinski, 1991) [Vilkamaa et al. 2013a]
Cratyna uliginosa (Lengersdorf, 1929) [Hackman 1980]
Cratyna vaporariorum (Frey, 1948) [Hackman 1980]
sg. ***Diversicratyna*** Menzel & Mohrig, 1998
Cratyna spiculosa (Rudzinski, 1993) [Salmela and Vilkamaa 2005]
sg. ***Spathobdella*** Frey, 1948
Cratyna colei (Freeman, 1990)
= *C. brachialis* auct. nec (Winnertz, 1867) [Hackman 1980]
Cratyna falcata (Tuomikoski, 1960) [Hackman 1980]
Cratyna falcifera (Lengersdorf, 1933) [Hackman 1980]
Cratyna longispina (Pettey, 1918)
= *C. tuberculata* (Tuomikoski, 1960) [Hackman 1980]
Cratyna nobilis (Winnertz, 1867) [Hackman 1980]
= *C. brachialis* (Winnertz, 1867)
Cratyna perplexa (Winnertz, 1867)
= *C. socialis* (Winnertz, 1867) [Hackman 1980]
= *C. brevicornis* (Tuomikoski, 1957) [Tuomikoski 1957]
CTENOSCIARA Tuomikoski, 1960
Ctenosciara exigua Salmela & Vilkamaa, 2005 [Salmela and Vilkamaa 2005]
Ctenosciara hyalipennis (Meigen, 1804) [Hackman 1980]
DICHOPYGINA Vilkamaa, Hippa & Komarova, 2004
Dichopygina aculeata Vilkamaa, Hippa & Komarova, 2004 [Vilkamaa et al. 2004]
Dichopygina intermedia (Mohrig & Krivosheina, 1982) [Vilkamaa et al. 2004]
Dichopygina nigrohalteralis (Frey, 1948) [Hackman 1980]
Dichopygina ramosa Vilkamaa, Hippa & Komarova, 2004 [Vilkamaa et al. 2004]

DOLICHOSCIARA Tuomikoski, 1960
Dolichosciara flavipes (Meigen, 1804) [Hackman 1980]
Dolichosciara hippai Komarova & Vilkamaa, 2006 [Vilkamaa et al. 2013a]
Dolichosciara nigrovittata (Strobl, 1910) [Vilkamaa et al. 2013a]
Dolichosciara orcina Tuomikoski, 1960 [Tuomikoski 1960b]
Dolichosciara ornata (Winnertz, 1867) [Tuomikoski 1960b]
Dolichosciara saetosa (Lengersdorf, 1929) [Tuomikoski 1960b]
Dolichosciara spissispina Vilkamaa, Hippa & Heller, 2013 [Vilkamaa et al. 2013c]
EPIDAPUS Haliday, 1851
sg. ***Epidapus*** Haliday, 1851
Epidapus alnicola (Tuomikoski, 1957) [Hackman 1980]
Epidapus atomarius (De Geer, 1778) [Vilkamaa et al. 2007]
Epidapus gracilis (Walker, 1848) [Hackman 1980]
Epidapus ignotus (Lengersdorf, 1942)
= *E. gracilior* (Tuomikoski, 1960) [Hackman 1980]
Epidapus microthorax (Börner, 1903)
= *E. gracilicornis* (Lengersdorf, 1926) [Hackman 1980]
Epidapus schillei (Börner, 1903)
= *E. titan* Frey, 1948 [Hackman 1980]
= *E. intermittens* Tuomikoski, 1959
sg. ***Pseudoaptanogyna*** Vimmer, 1926
Epidapus abieticola Frey, 1948 [Hackman 1980]
Epidapus bispinulosus Mohrig & Kauschke, 1994 [Vilkamaa et al. 2013a]
Epidapus echinatum Mohrig & Kozánek, 1992 [Vilkamaa et al. 2013a]
Epidapus ignavus (Lengersdorf, 1941) [Vilkamaa et al. 2013a]
KEILBACHIA Mohrig, 1987
Keilbachia ferrata (Hippa & Vilkamaa, 1994) [Hippa and Vilkamaa 1994]
LEPTOSCIARELLA Tuomikoski, 1960
sg. ***Hirtipennia*** Mohrig & Menzel, 1997
Leptosciarella hirtipennis (Zetterstedt, 1838) [Hackman 1980]
Leptosciarella holotricha Mohrig & Menzel, 1997 [Vilkamaa et al. 2013a]
sg. ***Leptosciarella*** Tuomikoski, 1960
Leptosciarella brevior (Tuomikoski, 1960) [Hackman 1980]
Leptosciarella brevipalpa (Mohrig & Menzel, 1992) [Vilkamaa et al. 2013a]
Leptosciarella claviforceps (Tuomikoski, 1960) [Hackman 1980]
Leptosciarella dimera (Tuomikoski, 1960) [Hackman 1980]
Leptosciarella fuscipalpa (Mohrig & Mamaev, 1979) [Mohrig and Menzel 1997]
Leptosciarella helvetica (Rudzinski, 1992) [Salmela and Vilkamaa 2005]
Leptosciarella hispida (Winnertz, 1871)
Leptosciarella ignis Heller, 2012 [Heller 2012]
= *L. juniperi* misid. [Vilkamaa et al. 2007]
Leptosciarella krille Heller, 2012 [Heller 2012]
Leptosciarella melanoma (Mohrig & Menzel, 1990) [Vilkamaa et al. 2013a]
Leptosciarella nudinervis (Tuomikoski, 1960) [Hackman 1980]
Leptosciarella pilosa (Staeger, 1840) [Hackman 1980]
Leptosciarella reducta Heller & Menzel, 2013 [Heller and Menzel 2013]
Leptosciarella rejecta (Winnertz, 1867) [Vilkamaa et al. 2007]
Leptosciarella scutellata (Staeger, 1840) [Hackman 1980]
= *L. elegans* (Winnertz, 1867) [Hackman 1980]
Leptosciarella subcoarctata Mohrig & Menzel, 1997 [Vilkamaa et al. 2007]

Leptosciarella subpilosa (Edwards, 1925) [Hackman 1980]
Leptosciarella subspinulosa (Edwards, 1925) [Vilkamaa et al. 2013a]
Leptosciarella subviatica Mohrig & Menzel, 1997 [Vilkamaa et al. 2013a]
Leptosciarella trochanterata (Zetterstedt, 1851)
= *L. coarctata* (Winnertz, 1867) [Hackman 1980]
Leptosciarella truncata (Tuomikoski, 1960) [Hackman 1980]
Leptosciarella viatica (Winnertz, 1867) [Hackman 1980]
Leptosciarella viaticella Mohrig & Krivosheina, 1979 [Vilkamaa et al. 2013a]
Leptosciarella yerburyi (Freeman, 1983) [Vilkamaa et al. 2007]
sg. ***Leptospina*** Mohrig & Menzel, 1997
Leptosciarella atricha (Tuomikoski, 1960) [Hackman 1980]
sg. ***Trichosiopsis*** Tuomikoski, 1960
Leptosciarella tuberculigera (Tuomikoski, 1960) [Hackman 1980]
LYCORIELLA Frey, 1942
sg. ***Coelostylina*** Tuomikoski, 1960
Lycoriella eflagellata Tuomikoski, 1960 [Hackman 1980]
Lycoriella freyi Tuomikoski, 1960 [Hackman 1980]
sg. ***Hemineurina*** Tuomikoski, 1960
Lycoriella algida (Frey, 1948) [Hackman 1980]
Lycoriella cochleata (Rübsaamen, 1898) [Hackman 1980]
Lycoriella conspicua (Winnertz, 1867) [Hackman 1980]
Lycoriella inflata (Winnertz, 1867)
= *L. venosa* auct. nec (Staeger, 1840) [Hackman 1980]
Lycoriella modesta (Staeger, 1840) [Hackman 1980]
Lycoriella piristylata Vilkamaa, Hippa & Heller, 2013 [Vilkamaa et al. 2013c]
Lycoriella thuringiensis Menzel & Mohrig, 1991 [Vilkamaa et al. 2013a]
Lycoriella vitticollis (Holmgren, 1883)
= *L. permutata* (Lundbeck, 1900) [Valkeila 1959, Hackman 1980]
sg. ***Lycoriella*** Frey, 1942
Lycoriella aberrans Tuomikoski, 1960 [Hackman 1980]
Lycoriella acutostylia Mohrig & Menzel, 1990 [Mukkala et al. 2005]
Lycoriella agraria (Felt, 1897)
= *L. cellaris* (Lengersdorf, 1934) [Hackman 1980]
Lycoriella approximatonervis (Frey, 1948) [Hackman 1980]
Lycoriella brevipila Tuomikoski, 1960 [Hackman 1980, Menzel and Heller 2007]
Lycoriella inconspicua Tuomikoski, 1960 [Hackman 1980]
Lycoriella ingenua (Dufour, 1839) [Menzel and Mohrig 1999]
= *L. solani* (Winnertz, 1871) [Hackman 1980]
Lycoriella latilobata Menzel & Mohrig, 2000
Lycoriella lundstromi (Frey, 1948) [Hackman 1980]
Lycoriella micria Mohrig & Menzel, 1990 [Salmela and Vilkamaa 2005]
Lycoriella minutula Mohrig & Krivosheina, 1987 [Vilkamaa et al. 2013a]
Lycoriella pallidior Tuomikoski, 1960 [Hackman 1980]
Lycoriella parva (Holmgren, 1869)
= *L. obscuratipes* (Frey, 1948) [Hackman 1980]
= *L. curvispina* Tuomikoski, 1960 [Hackman 1980]
Lycoriella sativae (Johannsen, 1912)
= *L. castanescens* (Lengersdorf, 1940)
= *L. fucorum* (Frey, 1948) [Hackman 1980]
Lycoriella subterranea (Märkel, 1844)

= *L. vanderwieli* (Schmitz, 1920) [Hackman 1980]
Lycoriella tenera Vilkamaa, Hippa & Heller, 2013 [Vilkamaa et al. 2013c]
Lycoriella weberi Menzel & Heller, 2013 [Heller and Menzel 2013]
MOUFFETINA Frey, 1942
Mouffetina expolita (Coquillett, 1900) [Vilkamaa et al. 2013a]
Mouffetina pulchricornis (Edwards, 1925) [Hackman 1980]
Mouffetina silvestris (Mohrig & Antonova, 1978) [Vilkamaa et al. 2013a]
PEYERIMHOFFIA Kieffer, 1903
Peyerimhoffia crassistylata (Frey, 1948) [Hackman 1980]
Peyerimhoffia infera Vilkamaa & Hippa, 2005 [Vilkamaa and Hippa 2005]
Peyerimhoffia menzeli Vilkamaa & Hippa, 2005 [Vilkamaa and Hippa 2005]
Peyerimhoffia quadrifera Vilkamaa, Hippa & Heller, 2013 [Vilkamaa et al. 2013c]
Peyerimhoffia sepei Hippa & Vilkamaa, 2005 [Hippa and Vilkamaa 2005]
Peyerimhoffia thula Vilkamaa & Hippa, 2005 [Vilkamaa and Hippa 2005]
Peyerimhoffia vagabunda (Winnertz, 1867)
= *P. brachyptera* Kieffer, 1903 [Hackman 1980]
PHYTOSCIARA Frey, 1942
Phytosciara halterata (Lengersdorf, 1926) [Hackman 1980]
Phytosciara macrotricha (Lengersdorf, 1926) [Vilkamaa et al. 2007]
PNYXIA Johannsen, 1912
Pnyxia scabiei (Hopkins, 1895) [Hackman 1980]
PNYXIOPSIS Tuomikoski, 1960
Pnyxiopsis aliger Tuomikoski, 1960 [Hackman 1980]
Pnyxiopsis degener (Tuomikoski, 1957) [Hackman 1980]
PROSCIARA Frey, 1942
Prosciara furtiva Vilkamaa & Hippa, 1996 [Vilkamaa et al. 2013a]
Prosciara plusiochaeta Hippa & Vilkamaa, 1991 [Hippa and Vilkamaa 1991]
Prosciara porrecta (Lengersdorf, 1929) [Hackman 1980]
Prosciara producta Tuomikoski, 1960 [Hackman 1980]
Prosciara prosciaroides (Tuomikoski, 1960) [Hackman 1980]
Prosciara ungulata (Winnertz, 1867) [Hackman 1980]
PSEUDOLYCORIELLA Menzel & Mohrig, 1998
Pseudolycoriella brunnea (Bukowski & Lengersdorf, 1936) [Salmela and Vilkamaa 2005]
Pseudolycoriella japonensis (Mohrig & Menzel, 1992) [Vilkamaa et al. 2013a]
Pseudolycoriella koreensis (Mohrig & Menzel, 1992) [Vilkamaa et al. 2013a]
Pseudolycoriella monticula (Mohrig & Menzel, 1992) [Vilkamaa et al. 2013a]
Pseudolycoriella nodulosa (Mohrig & Krivosheina, 1985) [Salmela and Vilkamaa 2005]
Pseudolycoriella paludum (Frey, 1948) [Hackman 1980]
Pseudolycoriella subbruckii (Mohrig & Hövemeyer, 1992) [Vilkamaa et al. 2013a]
SCATOPSCIARA Edwards, 1927
sg. **Scatopsciara** Edwards, 1927
Scatopsciara atomaria (Zetterstedt, 1851)
= *S. vivida* (Winnertz, 1867) [Hackman 1980]
Scatopsciara bucera Rudzinski, 1994 [Vilkamaa et al. 2013a]
Scatopsciara calamophila Frey, 1948 [Hackman 1980]
Scatopsciara curviforceps (Bukowski & Lengersdorf, 1936)
= *S. myrmecophila* Frey, 1948 [Frey 1948]
Scatopsciara edwardsi Freeman, 1983 [Vilkamaa et al. 2013a]
Scatopsciara fluviatilis (Lengersdorf, 1940) [Hackman 1980]

Scatopsciara geophila (Tuomikoski, 1960) [Hackman 1980]
Scatopsciara multispina (Bukowski & Lengersdorf, 1936) [Hackman 1980]
Scatopsciara neglecta Menzel & Mohrig, 1998 [Salmela and Vilkamaa 2005]
Scatopsciara pusilla (Meigen, 1818) [Hackman 1980]
Scatopsciara subcalamophila Menzel & Mohrig, 1991 [Vilkamaa et al. 2013a]
Scatopsciara subciliata Tuomikoski, 1960 [Hackman 1980]
Scatopsciara tricuspidata (Winnertz, 1867) [Hackman 1980]
= *S. degenerans* (Frey, 1948) [Hackman 1980]
Scatopsciara vitripennis (Meigen, 1818) [Hackman 1980]
sg. ***Xenopygina*** Frey, 1948
Scatopsciara gabyae (Heller, 1998) [Vilkamaa et al. 2013a]
Scatopsciara obliqua Vilkamaa, Hippa & Heller, 2013 [Vilkamaa et al. 2013c]
Scatopsciara paradoxa (Frey, 1948) [Vilkamaa et al. 2013a]
Scatopsciara simillima (Tuomikoski, 1960) [Hackman 1980]
SCHWENCKFELDINA Frey, 1942
Schwenckfeldina carbonaria (Meigen, 1830) [Hackman 1980]
Schwenckfeldina pectinea Menzel & Mohrig, 1991 [Menzel and Mohrig 1999]
SCIARA Meigen, 1803
Sciara flavimana Zetterstedt, 1851 [Hackman 1980]
Sciara hebes (Loew, 1869)
= *S. mendax* Tuomikoski, 1960 [Hackman 1980]
= *S. nursei* Freeman, 1983 [Mukkala et al. 2005]
= *S. ulrichi* Menzel & Mohrig, 1998 [Mukkala et al. 2005]
Sciara helvola Winnertz, 1867 [Hackman 1980]
Sciara hemerobioides (Scopoli, 1763)
= *S. thomae* Linnaeus, 1767 [Hackman 1980]
Sciara humeralis Zetterstedt, 1851 [Hackman 1980]
Sciara lackschewitzi (Lengersdorf, 1934)
SCYTHROPOCHROA Enderlein, 1911
Scythropochroa quercicola (Winnertz, 1869) [Hackman 1980]
Scythropochroa radialis Lengersdorf, 1926 [Hackman 1980]
TRICHOSIA Winnertz, 1867
= ***Leptosciara*** Frey, 1942
= ***Lestremioides*** Frey, 1942
sg. ***Trichosia*** Winnertz, 1867
Trichosia acrotricha Tuomikoski, 1960 [Hackman 1980]
Trichosia borealis (Frey, 1942) [Hackman 1980]
Trichosia confusa Menzel & Mohrig, 1997 [Vilkamaa et al. 2007]
Trichosia diota (Garrett, 1925) [Mohrig et al. 2013]
Trichosia flavicoxa Tuomikoski, 1960 [Hackman 1980]
Trichosia glabra (Meigen, 1830) [Salmela and Vilkamaa 2005]
Trichosia habilis (Johannsen, 1912) []
= *T. edwardsi* (Lengersdorf, 1930)
Trichosia morio (Fabricius, 1794)
= *T. caudata* (Walker, 1848) [Hackman 1980]
Trichosia splendens Winnertz, 1867 [Hackman 1980]
Trichosia ussurica Mohrig & Antonova, 1978 [Vilkamaa et al. 2013a]
XYLOSCIARA Tuomikoski, 1957
sg. ***Protoxylosciara*** Tuomikoski, 1960
Xylosciara longiforceps (Bukowski & Lengersdorf, 1936) [Hackman 1980]

sg. ***Xylosciara*** Tuomikoski, 1957
Xylosciara heptacantha Tuomikoski, 1957 [Hackman 1980]
Xylosciara lignicola (Winnertz, 1867) [Hackman 1980]
Xylosciara microdon (Frey, 1948) [Hackman 1980]
Xylosciara misella (Frey, 1948) [Hackman 1980]
Xylosciara phryganophila (Frey, 1948) [Hackman 1980]
Xylosciara senta Vilkamaa, Hippa & Heller, 2013 [Vilkamaa et al. 2013c]
Xylosciara spinata (Pettey, 1918)
= *X. betulae* Tuomikoski, 1960 [Hackman 1980]
Xylosciara steleocera Tuomikoski, 1960 [Hackman 1980]
Xylosciara trimera Tuomikoski, 1960 [Hackman 1980]
Xylosciara validinervis Tuomikoski, 1960 [Hackman 1980]
ZYGONEURA Meigen, 1830
sg. ***Allozygoneura*** Menzel & Mohrig, 1998
Zygoneura calthae Tuomikoski, 1960 [Hackman 1980]
sg. ***Zygoneura*** Meigen, 1830
Zygoneura sciarina Meigen, 1830 [Hackman 1980]

Excluded species

Bradysia betuleti (Lengersdorf, 1940) [Tuomikoski 1960a, Hackman 1980]
Bradysia subbetuleti Mohrig & Krivosheina, 1989 [Salmela and Vilkamaa 2005]
Corynoptera concinna (Winnertz, 1867) [Tuomikoski 1960a, Hackman 1980]
Corynoptera bistrispina (Bukowski & Lengersdorf, 1936) [Tuomikoski 1960a]
Lycoriella auripila (Winnertz, 1867) [Hackman 1980]
Trichosia trochanterata (Zetterstedt, 1851) [Tuomikoski 1960a, Hackman 1980]

UNPLACED IN SCIAROIDEA ***SCIAROSOMA*** Chandler, 2002

Sciarosoma nigriclava (Strobl, 1898)

= *S. borealis* Chandler, 2002 [Chandler 2002]

XYLOPHAGIDAE Fallén, 1810

Supporting references for *Kahanpää J, Winqvist K, Zeegers T (2014) Checklist of the lower Brachycera of Finland: Tabanomorpha, Asilomorpha and associated families (Diptera). In: Kahanpää J, Salmela J (Eds) Checklist of the Diptera of Finland. ZooKeys @@: @-@. doi: 10.3897/zookeys.??.7198*

XYLOPHAGUS Meigen, 1803
= ***Erinna*** Meigen, 1800 suppr.
Xylophagus ater Meigen, 1804
= *X. compeditus* Wiedemann, 1820 [Hackman 1980]
Xylophagus cinctus (De Geer, 1776) [Hackman 1980]
Xylophagus inermis Krivosheina & Krivosheina, 2000
= *X. matsumurae* Miyatake, 1965 misid.[Kahanpää and Winqvist 2005]
Xylophagus junki (Szilády, 1932) [Hackman 1980]
Xylophagus kowarzi (Pleske, 1925)
= *X. ater* auct. nec Meigen, 1804 [Hackman 1980]

Excluded species

Xylophagus matsumurae Miyatake, 1965 [Kahanpää and Winqvist 2005, Woodley 2011b]

RHAGIONIDAE Latreille, 1802
Supporting references for *Kahanpää J, Winqvist K, Zeegers T (2014) Checklist of the lower Brachycera of Finland: Tabanomorpha, Asilomorpha and associated families (Diptera). In: Kahanpää J, Salmela J (Eds) Checklist of the Diptera of Finland. ZooKeys @@: @-@. doi: 10.3897/zookeys.??.7198*

RHAGIONINAE Latreille, 1802
RHAGIO Fabricius, 1775
Rhagio annulatus (De Geer, 1776) [Hackman 1980]
Rhagio lineola Fabricius, 1794 [Hackman 1980]
Rhagio maculatus (De Geer, 1776) [Hackman 1980]
Rhagio notatus (Meigen, 1820) [Frey et al. 1941, Kahanpää and Winqvist 2005]
Rhagio scolopaceus (Linnaeus, 1758) [Hackman 1980]
Rhagio tringarius (Linnaeus, 1758) [Hackman 1980]
CHRYSOPILINAE Bezzi, 1903
CHRYSOPILUS Macquart, 1826
Chrysopilus auratus (Fabricius, 1805) [Hackman 1980]
? = *C. cristatus* (Fabricius, 1775) nom. dubium
Chrysopilus luteolus (Fallén, 1814) [Hackman 1980]
Chrysopilus nubecula (Fallén, 1814) [Hackman 1980]
? *Chrysopilus suomianus* (Szilády, 1934) [Szilády 1934, Kahanpää and Winqvist 2005]
SPANIINAE Rondani, 1856
OMPHALOPHORA Becker, 1900
Omphalophora oculata Becker, 1900
= *O. lapponica* Frey, 1911 [Hackman 1980]

PTIOLINA Zetterstedt, 1842
Ptiolina nigra Zetterstedt, 1842 [Nartshuk 1995]
Ptiolina nigrina Wahlgren, 1854 [Hackman 1980]
Ptiolina nitida Wahlgren, 1854 [Hackman 1980]
Ptiolina obscura (Fallén, 1814)[Hackman 1980]
SPANIA Meigen, 1830
Spania nigra Meigen, 1830 [Hackman 1980]
SYMPHOROMYIA Frauenfeld, 1867
sg. ***Paraphoromyia*** Becker, 1921
Symphoromyia crassicornis (Panzer, 1806) [Hackman 1980]

ATHERICIDAE Nowicki, 1873

Supporting references for *Kahanpää J, Winqvist K, Zeegers T (2014) Checklist of the lower Brachycera of Finland: Tabanomorpha, Asilomorpha and associated families (Diptera). In: Kahanpää J, Salmela J (Eds) Checklist of the Diptera of Finland. ZooKeys @@: @-@. doi: 10.3897/zookeys.??.7198*

ATHERIX Meigen, 1803

Atherix ibis (Fabricius, 1798) [Hackman 1980]

TABANIDAE Latreille, 1802

Supporting references for *Kahanpää J, Winqvist K, Zeegers T (2014) Checklist of the lower Brachycera of Finland: Tabanomorpha, Asilomorpha and associated families (Diptera). In: Kahanpää J, Salmela J (Eds) Checklist of the Diptera of Finland. ZooKeys @@: @-@. doi: 10.3897/zookeys.??.7198*

CHRYSOPSINAE Lutz, 1905
tribe Chrysopsini Lutz, 1905
CHRYSOPS Meigen, 1803
sg. ***Chrysops*** Meigen, 1803
Chrysops caecutiens (Linnaeus, 1758) [Hackman 1980]
Chrysops divaricatus Loew, 1858 [Hackman 1980]
Chrysops nigripes Zetterstedt, 1838 [Hackman 1980]
= *C. lapponicus* Loew, 1858 [Frey et al. 1941, Karvonen 1969]
Chrysops relictus Meigen, 1820 [Hackman 1980]
= *C. melanopleurus* Wahlberg, 1848 [Frey et al. 1941]
Chrysops rufipes Meigen, 1820 [Hackman 1980]
Chrysops sepulcralis (Fabricius, 1794) [Hackman 1980]
Chrysops viduatus (Fabricius, 1794) [Hackman 1980]
= *C. pictus* Meigen, 1820 [Frey et al. 1941]
TABANINAE Latreille, 1802
tribe Haematopotini Enderlein, 1922
HAEMATOPOTA Meigen, 1803
Haematopota italica Meigen, 1804 [Kahanpää 2013d]
Haematopota crassicornis Wahlberg, 1848 [Silfverberg 1981, Vuorimies 1984]
Haematopota pluvialis (Linnaeus, 1758) [Hackman 1980]
= *H. italica* misid. [Vuorimies 1984]
? *Haematopota subcylindrica* Pandellé, 1883 [Vuorimies 1984]
HEPTATOMA Meigen, 1803
Heptatoma pellucens (Fabricius, 1776) [Hackman 1980]
tribe Tabanini Latreille, 1802
ATYLOTUS Osten Sacken, 1876
Atylotus fulvus (Meigen, 1820) [Hackman 1980]
Atylotus plebeius (Fallén, 1817) [Hackman 1980]
Atylotus rusticus (Linnaeus, 1767) [Hackman 1980]
Atylotus sublunaticornis (Zetterstedt, 1842) [Kauri 1964, Hackman 1980]
HYBOMITRA Enderlein, 1922
Hybomitra arpadi (Szilády, 1923) [Hackman 1980]
Hybomitra astuta (Osten Sacken, 1876)
= *H. polaris* (Frey, 1915) [Hackman 1980]
Hybomitra auripila (Meigen, 1820) [Karvonen 1976, Hackman 1980]
= *H. aterrima* (Meigen, 1820)
Hybomitra bimaculata (Macquart, 1826) [Mee 1961b, Hackman 1980]
= *H. tropica* misid.[Karvonen 1969]
? = *H. solstitialis* (Meigen, 1820) [Frey et al. 1941, Mee 1961b, Hackman 1980]
Hybomitra borealis (Fabricius, 1781) [Hackman 1980]
= *H. lapponicus* (Wahlberg, 1848) [Frey et al. 1941]
Hybomitra ciureai (Séguy, 1937) [Hackman 1980]
= *H. schineri* Lyneborg, 1959 [Mee 1961b]

Hybomitra distinguenda (Verrall, 1909) [Hackman 1980]
Hybomitra kaurii Chvála & Lyneborg, 1970 [Hackman 1980]
= *H. borealis* misid.[Frey et al. 1941]
Hybomitra lundbecki Lyneborg, 1959 [Hackman 1980]
= *H. fulvicornis* misid.[Mee 1961b, Karvonen 1969]
Hybomitra lurida (Fallén, 1817) [Hackman 1980]
Hybomitra montana (Meigen, 1820) [Hackman 1980]
Hybomitra muehlfeldi (Brauer, 1880) [Hackman 1980]
= *H. flaviceps* (Zetterstedt, 1842)
Hybomitra nigricornis (Zetterstedt, 1842) [Hackman 1980]
Hybomitra nitidifrons (Szilády, 1914) [Hackman 1980]
= *H. confinis* misid.[Frey et al. 1941]
Hybomitra sexfasciata (Hine, 1923) [Hackman 1980]
= *H. borealis anderi* Kauri, 1951 [Karvonen 1969]
Hybomitra tarandina (Linnaeus, 1758) [Hackman 1980]
Hybomitra tropica (Linnaeus, 1758) [Kauri 1969, Kahanpää and Winqvist 2005]
TABANUS Linnaeus, 1758
Tabanus autumnalis Linnaeus, 1761 [Hackman 1980]
Tabanus bovinus Linnaeus, 1758 [Hackman 1980]
Tabanus bromius Linnaeus, 1758 [Hackman 1980]
Tabanus cordiger Meigen, 1820 [Hackman 1980]
Tabanus maculicornis Zetterstedt, 1842 [Hackman 1980]
Tabanus sudeticus Zeller, 1842 [Hackman 1980]

Excluded species

Tabanus miki Brauer, 1880 [Kauri 1969, Hackman 1980, Haarto and Winqvist 2014]

STRATIOMYIDAE Latreille, 1802

Supporting references for *Kahanpää J, Winqvist K, Zeegers T (2014) Checklist of the lower Brachycera of Finland: Tabanomorpha, Asilomorpha and associated families (Diptera). In: Kahanpää J, Salmela J (Eds) Checklist of the Diptera of Finland. ZooKeys @@: @-@. doi: 10.3897/zookeys.??.7198*

BERIDINAE Westwood, 1838
BERIS Latreille, 1802
Beris chalybata (Forster, 1771) [Hackman 1980]
Beris clavipes (Linnaeus, 1767) [Hackman 1980]
Beris fuscipes Meigen, 1820 [Hackman 1980]
Beris hauseri Stuke, 2004 [Woodley 2011a]
 = *B. strobli* auct. nec [Hackman 1972b, 1980]
Beris morrisii Dale, 1841 [Hackman 1980]
NEMOTELINAE Kertész, 1912
NEMOTELUS Geoffroy, 1762
 sg. ***Camptopelta*** Williston, 1917
Nemotelus nigrinus Fallén, 1817 [Hackman 1980]
 sg. ***Nemotelus*** Geoffroy, 1762
Nemotelus infortunatus Kahanpää, 2010 [Kahanpää 2010]
Nemotelus notatus Zetterstedt, 1842 [Hackman 1980]
Nemotelus uliginosus (Linnaeus, 1767) [Hackman 1980]
PACHYGASTRINAE Loew, 1856
BERKSHIRIA Johnson, 1914
 = ***Pseudowallacea*** Kertész, 1921
Berkshiria hungarica (Kertesz, 1921)
 = *B. albistylum* misid.[Hackman 1980]
 = *B. barovskii* misid.[Frey et al. 1941]
NEOPACHYGASTER Austen, 1901
Neopachygaster meromelas (Dufour, 1841) [Hackman 1980]
 = *N. orbitalis* (Wahlberg, 1854) [Frey et al. 1941]
ZABRACHIA Coquillett, 1901
Zabrachia minutissima (Zetterstedt, 1838) [Hackman 1980]
Zabrachia tenella (Jaennicke, 1866) [Krivosheina and Rozkošný 1990]
SARGINAE Walker, 1834
CHLOROMYIA Duncan, 1837
Chloromyia formosa (Scopoli, 1763) [Hackman 1980]
MICROCHRYSA Loew, 1855
Microchrysa cyaneiventris (Zetterstedt, 1842) [Hackman 1980]
Microchrysa flavicornis (Meigen, 1822) [Hackman 1980]
Microchrysa polita (Linnaeus, 1758) [Hackman 1980]
SARGUS Fabricius, 1798
Sargus cuprarius (Linnaeus, 1758) [Hackman 1980]
Sargus flavipes Meigen, 1822
 = *S. nigripes* Zetterstedt, 1842
 = *S. splendens* of authors[Hedström 1969, Hackman 1980]
Sargus iridatus (Scopoli, 1763) [Hackman 1980]
Sargus rufipes Wahlberg, 1854 [Hackman 1980]
STRATIOMYINAE Latreille, 1802

tribe Oxycerini Enderlein, 1914

OXYCERA Meigen, 1803

Oxycera centralis Loew, 1863

= *O. centralis* Frey, 1911 preocc.

= *O. freyi* Lindner, 1938 [Hackman 1980]

Oxycera dives Loew, 1845 [Fre 1935, Hackman 1980]

Oxycera trilineata (Linnaeus, 1767) [Hackman 1980]

tribe Stratiomyini Latreille, 1802

ODONTOMYIA Meigen, 1803

Odontomyia angulata (Panzer, 1798) [Hackman 1980]

Odontomyia argentata (Fabricius, 1794) [Mee 1961a, Hackman 1980]

Odontomyia microleon (Linnaeus, 1758) [Hackman 1980]

OPLODONTHA Rondani, 1863

Oplodontha viridula (Fabricius, 1775) [Hackman 1980]

STRATIOMYS Geoffroy, 1762

Stratiomys singularior (Harris, 1776)

= *S. furcata* Fabricius, 1794 [Hackman 1980]

Excluded species

Beris geniculata Curtis, 1830

Odontomyia hydroleon (Linnaeus, 1758) [Hackman 1980, Kahanpää and Winqvist 2005]

XYLOMYIDAE Verrall, 1901

Supporting references for *Kahanpää J, Winqvist K, Zeegers T (2014) Checklist of the lower Brachycera of Finland: Tabanomorpha, Asilomorpha and associated families (Diptera). In: Kahanpää J, Salmela J (Eds) Checklist of the Diptera of Finland. ZooKeys @@: @-@. doi: 10.3897/zookeys.??.7198*

XYLOMYA Rondani, 1861
Xylomya czekanovskii Pleske, 1925
= *X. interrupta* auct. nec (Pleske, 1926) [Rozkošný 1973, Hackman 1980]
= *X. maculata* auct. nec (Meigen, 1804) [Frey et al. 1941, Rozkošný 1973]

ASILIDAE Latreille, 1802

Supporting references for *Kahanpää J, Winqvist K, Zeegers T (2014) Checklist of the lower Brachycera of Finland: Tabanomorpha, Asilomorpha and associated families (Diptera). In: Kahanpää J, Salmela J (Eds) Checklist of the Diptera of Finland. ZooKeys @@: @-@. doi: 10.3897/zookeys.??.7198*

ASILINAE Latreille, 1802
ASILUS Linnaeus, 1758
Asilus crabroniformis Linnaeus, 1758 [Hackman 1980]
DIDYSMACHUS Lehr, 1996
Didysmachus picipes (Meigen, 1820) [Hackman 1980]
DYSMACHUS Loew, 1860
Dysmachus trigonus (Meigen, 1804) [Hackman 1980]
MACHIMUS Loew, 1849
Machimus setibarbis Loew, 1849 [Hackman 1980]
NEOITAMUS Osten Sacken, 1878
Neoitamus cothurnatus (Meigen, 1820) [Hackman 1980]
Neoitamus cyanurus (Loew, 1849) [Hackman 1980]
Neoitamus socius (Loew, 1871) [Hackman 1980]
NEOMOCHTHERUS Osten Sacken, 1878
Neomochtherus pallipes (Meigen, 1820) [Hackman 1980]
PAMPONERUS Loew, 1849
Pamponerus germanicus (Linnaeus, 1758) [Hackman 1980]
PHILONICUS Loew, 1849
Philonicus albiceps (Meigen, 1820) [Hackman 1980]
RHADIURGUS Loew, 1849
Rhadiurgus variabilis (Zetterstedt, 1838) [Hackman 1980]
TOLMERUS Loew, 1849
Tolmerus atricapillus (Fallén, 1814) [Hackman 1980]
Tolmerus pyragra (Zeller, 1840) [Kahanpää and Winqvist 2005]
LAPHRINAE Macquart, 1838
tribe Andrenosomatini Hull, 1962
ANDRENOSOMA Rondani, 1856
Andrenosoma albibarbe (Meigen, 1820) [Hackman 1980]
tribe Laphrini Macquart, 1838 Macquart, 1838
CHOERADES Walker, 1851
Choerades fuliginosus (Panzer, 1798) [Hackman 1980]
Choerades gilvus (Linnaeus, 1758) [Hackman 1980]
Choerades igneus (Meigen, 1820) [Hackman 1980]
Choerades lapponicus (Zetterstedt, 1842) [Hackman 1980]
Choerades marginatus (Linnaeus, 1758) [Hackman 1980]
LAPHRIA Meigen, 1803
Laphria flava (Linnaeus, 1761) [Hackman 1980]
Laphria gibbosa (Linnaeus, 1758) [Hackman 1980]
LEPTOGASTRINAE Schiner, 1862
LEPTOGASTER Meigen, 1803
Leptogaster cylindrica (De Geer, 1776) [Hackman 1980]
Leptogaster guttiventris Zetterstedt, 1842 [Hackman 1980]
STENOPOGONINAE Hull, 1962

tribe Dioctriini Hendel, 1936
DIOCTRIA Meigen, 1803
Dioctria atricapilla Meigen, 1804 [Hackman 1980]
Dioctria cothurnata Meigen, 1820 [Hackman 1980]
Dioctria hyalipennis (Fabricius, 1794) [Hackman 1980]
Dioctria oelandica (Linnaeus, 1758) [Hackman 1980]
Dioctria rufipes (De Geer, 1776) [Hackman 1980]
tribe Stegopogonini Hull, 1962
CYRTOPOGON Loew, 1847
Cyrtopogon flavimanus (Meigen, 1820) [Hackman 1980]
Cyrtopogon lapponicus (Zetterstedt, 1838) [Hackman 1980]
Cyrtopogon lateralis (Fallén, 1814) [Hackman 1980]
Cyrtopogon luteicornis (Zetterstedt, 1842) [Hackman 1980]
= *C. luteicornis* var. pollinosus Frey, 1911
Cyrtopogon pulchripes Loew, 1871 [Hackman 1980]
tribe Stichopogonini Hardy, 1930
LASIOPOGON Loew, 1847
Lasiopogon cinctus (Fabricius, 1781) [Hackman 1980]
Lasiopogon septentrionalis Lehr, 1984 [Cannings and Kahanpää 2013]

Excluded species

Choerades fimbriata (Meigen, 1820) [Lehr 1988, Kahanpää and Grichanov 2004]
Choerades ursulus (Loew, 1851) [Hackman 1980, Kahanpää and Grichanov 2004]
Cyrtopogon maculipennis (Macquart, 1834) [Hackman 1980, Kahanpää and Winqvist 2005]
Epitriptus arthriticus (Zeller, 1840) [Lehr 1988, Kahanpää and Grichanov 2004]
Machimus gonatistes (Zeller, 1840) [Kahanpää and Winqvist 2005]
Tolmerus cingulatus (Fabricius, 1781) [Lehr 1988, Kahanpää and Grichanov 2004]

BOMBYLIIDAE Latreille, 1802

Supporting references for *Kahanpää J, Winqvist K, Zeegers T (2014) Checklist of the lower Brachycera of Finland: Tabanomorpha, Asilomorpha and associated families (Diptera). In: Kahanpää J, Salmela J (Eds) Checklist of the Diptera of Finland. ZooKeys @@: @-@. doi: 10.3897/zookeys.??.7198*

PHTHIRIINAE Becker, 1913
tribe Phthiriini Becker, 1913
PHTHIRIA Meigen, 1803
Phthiria pulicaria (Mikan, 1796) [Hackman 1980]
BOMBYLIINAE Latreille, 1802
tribe Bombyliini Latreille, 1802
BOMBYLIUS Linnaeus, 1758
sg. ***Bombylius*** Linnaeus, 1758
Bombylius discolor Mikan, 1796 [Hackman 1980]
Bombylius major Linnaeus, 1758 [Hackman 1980]
Bombylius minor Linnaeus, 1758 [Hackman 1980]
= *B. allibarbis* Zetterstedt, 1842
= *B. albibarbis* misspelling[Hackman 1980]
SYSTOECHUS Loew, 1855
Systoechus ctenopterus (Mikan, 1796)
= *S. sulphureus* (Mikan, 1796) [Hackman 1980]
Systoechus gradatus (Wiedemann, 1820) [Hackman 1980]
ANTHRACINAE Latreille, 1804
tribe Anthracini Latreille, 1804
ANTHRAX Scopoli, 1763
Anthrax anthrax (Schrank, 1781) [Hackman 1980]
Anthrax trifasciatus Meigen, 1804
= *A. leucogaster* Wiedemann, 1820 [Hackman 1980]
Anthrax varius Fabricius, 1794 [Hackman 1980]
tribe Exoprosopini Becker, 1913
EXOPROSOPA Macquart, 1840
Exoprosopa capucina (Fabricius, 1781) [Hackman 1980]
MICOMITRA Bowden, 1964
Micomitra stupida (Rossi, 1790) [Hackman 1980]
tribe Villini Hull, 1973
HEMIPENTHES Loew, 1869
Hemipenthes maura (Linnaeus, 1758) [Hackman 1980]
Hemipenthes morio (Linnaeus, 1758) [Hackman 1980]
THYRIDANTHRAX Osten Sacken, 1886
Thyridanthrax fenestratus (Fallén, 1814) [Hackman 1980]
VILLA Lioy, 1864
Villa cingulata (Meigen, 1804) [Hackman 1980]
? *Villa halteralis* (Kowarz, 1883) [Hedström 1969, Hackman 1980]
Villa hottentotta (Linnaeus, 1758) [Hackman 1980]
Villa modesta (Meigen, 1820) [Kahanpää and Winqvist 2005]
Villa occulta (Wiedemann, 1820) [Hackman 1980]

Excluded species

Anastoechus nitidulus (Fabricius, 1794) [Hackman 1980, Kahanpää and Winqvist 2005]
Phthiria canescens Loew, 1846 [Zaitzev 1989, Kahanpää and Winqvist 2005]
Villa fasciata (Meigen, 1804) [Kahanpää and Winqvist 2005]
 = *V. circumdata* (Meigen, 1820) [Hackman 1980]
 = *V. venusta* (Meigen, 1820)
Villa longicornis Lyneborg, 1965 [Lyneborg 1965a, Kahanpää and Winqvist 2005]
Villa panisca (Rossi, 1790) [Hackman 1980, Kahanpää and Winqvist 2005]
 = *V. circumdata* auct. nec (Meigen, 1820)

MYTHICOMYIIDAE Melander, 1902

Supporting references for *Kahanpää J, Winqvist K, Zeegers T (2014) Checklist of the lower Brachycera of Finland: Tabanomorpha, Asilomorpha and associated families (Diptera). In: Kahanpää J, Salmela J (Eds) Checklist of the Diptera of Finland. ZooKeys @@: @-@. doi: 10.3897/zookeys.??.7198*

GLABELLULINAE Cockerell, 1914
GLABELLULA Bezzi, 1902
Glabellula arctica (Zetterstedt, 1838) [Hackman 1980]

SCENOPINIDAE Burmeister, 1835

Supporting references for *Kahanpää J, Winqvist K, Zeegers T (2014) Checklist of the lower Brachycera of Finland: Tabanomorpha, Asilomorpha and associated families (Diptera). In: Kahanpää J, Salmela J (Eds) Checklist of the Diptera of Finland. ZooKeys @@: @-@. doi: 10.3897/zookeys.??.7198*

SCENOPINUS Latreille, 1802
Scenopinus fenestralis (Linnaeus, 1758) [Hackman 1980]
Scenopinus niger (De Geer, 1776) [Hackman 1980]
Scenopinus sp. A
= *S. vitripennis* misid. [Haarto 2000]

THEREVIDAE Newman, 1834

Supporting references for *Kahanpää J, Winqvist K, Zeegers T (2014) Checklist of the lower Brachycera of Finland: Tabanomorpha, Asilomorpha and associated families (Diptera). In: Kahanpää J, Salmela J (Eds) Checklist of the Diptera of Finland. ZooKeys @@: @-@. doi: 10.3897/zookeys.??.7198*

THEREVINAE Newman, 1834
ACROSATHE Irwin & Lyneborg, 1981
Acrosathe annulata (Fabricius, 1805) [Hackman 1980]
DIALINEURA Rondani, 1856
Dialineura anilis (Linnaeus, 1761) [Hackman 1980]
DICHOGLENA Irwin & Lyneborg, 1981
Dichoglena nigripennis (Ruthe, 1831) [Hackman 1980]
PANDIVIRILIA Irwin & Lyneborg, 1981
Pandivirilia eximia (Meigen, 1820) [Hackman 1980]
PSILOCEPHALA Zetterstedt, 1838
Psilocephala imberbis (Fallén, 1814) [Hackman 1980]
SPIRIVERPA Irwin & Lyneborg, 1981
Spiriverpa lunulata (Zetterstedt, 1838) [Hackman 1980]
= *S. clausa* (Frey, 1911)
THEREVA Latreille, 1796
Thereva cinifera Meigen, 1830
= *T. subfasciata* Schummel, 1830 [Hackman 1980]
Thereva fuscinervis Zetterstedt, 1838 [Hackman 1980]
Thereva handlirschi Kröber, 1912 [Lyneborg 1968, Hackman 1980]
= *T. praestans* Collin, 1948 [Karppinen 1969a]
Thereva inornata Verrall, 1909 [Lyneborg 1968, Hackman 1980]
Thereva lanata Zetterstedt, 1838 [Hackman 1980]
Thereva microcephala Loew, 1847 [Karppinen 1969a, Hackman 1980]
Thereva nobilitata (Fabricius, 1775) [Hackman 1980]
Thereva plebeja (Linnaeus, 1758) [Hackman 1980]
Thereva strigata (Fabricius, 1794) [Karppinen 1969a, Hackman 1980]
Thereva unica (Harris, 1780)
= *T. bipunctata* Meigen, 1820 [Karppinen 1969a, Hackman 1980]
Thereva valida Loew, 1847 [Karppinen 1969a, Hackman 1980]
= *T. circumscripta* auct. nec Loew, 1847 [Hackman 1980, Kahanpää and Winqvist 2005]

Excluded species

Cliorismia ardea (Fabricius, 1794) [Hackman 1980, Kahanpää and Winqvist 2005]
Cliorismia rustica (Panzer, 1804) [Hackman 1980, Kahanpää and Winqvist 2005]
Pandivirilia nigroanalis (Kröber, 1928)

ACROCERIDAE Leach, 1815

Supporting references for *Kahanpää J, Winqvist K, Zeegers T (2014) Checklist of the lower Brachycera of Finland: Tabanomorpha, Asilomorpha and associated families (Diptera). In: Kahanpää J, Salmela J (Eds) Checklist of the Diptera of Finland. ZooKeys @@: @-@. doi: 10.3897/zookeys.??.7198*

ACROCERA Meigen, 1803
= ***Paracrocera*** Mik, 1886
sg. ***Acrocera*** Meigen, 1803
Acrocera orbiculus (Fabricius, 1787) [Frey et al. 1941, Hackman 1980]
= *A. globulus* (Panzer, 1804) [Frey et al. 1941, Hac 1970]
= *A. borealis* Zetterstedt, 1838 [Frey et al. 1941, Hac 1970]
OGCODES Latreille, 1796
sg. ***Ogcodes*** Latreille, 1796
Ogcodes borealis Cole, 1919 [Hac 1970, Hackman 1980]
Ogcodes gibbosus (Linnaeus, 1758) [Frey et al. 1941, Hackman 1980]
Ogcodes nigripes (Zetterstedt, 1838) [Frey et al. 1941, Hackman 1980]
Ogcodes pallipes Latreille in Olivier, 1812 [Frey et al. 1941, Hackman 1980]

UNCERTAIN FAMILY POSITION WITHIN EMPIDOIDEA Uncertain family position within Empidoidea (incertae familiae)

Supporting references for *Kahanpää J (2014) Checklist of the Empidoidea of Finland (Insecta, Diptera). In: Kahanpää J, Salmela J (Eds) Checklist of the Diptera of Finland. ZooKeys XX: X-X. doi: 10.3897/zookeys.??.7154*

ITEAPHILA Zetterstedt, 1838
Iteaphila macquarti Zetterstedt, 1838 [Hackman 1980]
Iteaphila nitidula Zetterstedt, 1838 [Mee 1927, Hackman 1980]
= *I. obscura* Zetterstedt, 1849
ANTHEPISCOPUS Becker, 1891
Anthepiscopus oedalinus (Zetterstedt, 1838) [Hackman 1980]

Excluded species

Iteaphila furcata (Zetterstedt, 1842) [Mee 1927, Hackman 1980]
EMPIDIDAE Latreille, 1809

Supporting references for *Kahanpää J (2014) Checklist of the Empidoidea of Finland (Insecta, Diptera). In: Kahanpää J, Salmela J (Eds) Checklist of the Diptera of Finland. ZooKeys @@: @-@. doi: 10.3897/zookeys.??.7154*

HEMERODROMIINAE Schiner, 1862
tribe Chelipodini Hendel, 1936
CHELIPODA Macquart, 1823
Chelipoda albiseta (Zetterstedt, 1838) [Hackman 1980]
Chelipoda inexspectata Tuomikoski, 1966 [Tuomikoski 1966, Hackman 1980]
Chelipoda vocatoria (Fallén, 1816) [Hackman 1980]
PHYLLODROMIA Zetterstedt, 1837
Phyllodromia melanocephala (Fabricius, 1794) [Hackman 1980]

HEMERODROMIINI Schiner, 1862
CHELIFERA Macquart, 1823
Chelifera concinnicauda Collin, 1927 [Hackman 1980]
= *C. lapponica* Frey, 1950
= *C. stigmatica* misid. [Hackman 1980]
Chelifera flavella (Zetterstedt, 1838) [Hackman 1980]
Chelifera frigelii (Zetterstedt, 1838) [Hackman 1980]
Chelifera precabunda Collin, 1961 [Hackman 1980]
Chelifera precatoria (Fallén, 1816) [Hackman 1980]
Chelifera subangusta Collin, 1961 [Haarto and Winqvist 2014]
Chelifera trapezina (Zetterstedt, 1838) [Hackman 1980]
HEMERODROMIA Meigen, 1822
Hemerodromia adulatoria Collin, 1927 [Kahanpää and Winqvist 2005]
Hemerodromia oratoria (Fallén, 1816) [Hackman 1980]
Hemerodromia raptoria Meigen, 1830 [Hackman 1980]
EMPIDINAE Latreille, 1809
tribe Empidini Latreille, 1809
EMPIS Linnaeus, 1758
sg. ***Anacrostichus*** Bezzi, 1909
Empis lucida Zetterstedt, 1838 [Hackman 1980]
sg. ***Coptophlebia*** Bezzi, 1909
Empis hyalipennis Fallén, 1816 [Hackman 1980]
sg. ***Empis*** Linnaeus, 1758
Empis acinerea Chvala, 1985
= *E. cinerea* Zetterstedt, 1855 [Hackman 1980]
Empis bicuspidata Collin, 1927 [Hackman 1980]
Empis caudatula Loew, 1867[Winqvist 2011]
Empis chioptera Meigen, 1804 [Hackman 1980]
Empis laminata Collin, 1927 [Chvála 1991]
Empis nigripes Fabricius, 1794
= *E. pennaria* Fallén, 1816 [Hackman 1980]
= *E. vernalis* Meigen, 1822
Empis pennipes Linnaeus, 1758 [Hackman 1980]
Empis prodromus Loew, 1867 [Hackman 1980]
Empis staegeri Collin, 1963 [Chvála 1991]
= *E. planetica* misid.[Hackman 1980, Chvála 1991]
Empis syrovatkai Chvála, 1985
= *E. plumipes* Zetterstedt, 1842 preocc.[Hackman 1980]
sg. ***Euempis*** Frey, 1953
Empis picipes Meigen, 1804 [Hackman 1980]
= *E. maculipes* Zetterstedt, 1842
Empis tessellata Fabricius, 1794 [Hackman 1980]
sg. ***Kritempis*** Collin, 1926
Empis livida Linnaeus, 1758 [Hackman 1980]
sg. ***Leptempis*** Collin, 1926
Empis grisea Fallén, 1816 [Flinck and Kahanpää 2013]
sg. ***Platyptera*** Meigen, 1803
Empis borealis Linnaeus, 1758 [Hackman 1980]
sg. ***Xanthempis*** Bezzi, 1909
Empis aemula Loew, 1873

Empis digramma Meigen in Gistl, 1835 [Hackman 1980]
Empis laetabilis Collin, 1926 [Hackman 1980]
Empis stercorea Linnaeus, 1761 [Hackman 1980]
Empis univittata Loew, 1867 [Hackman 1980, Fre 1935]
RHAMPHOMYIA Meigen, 1822
sg. ***Aclonempis*** Collin, 1926
Rhamphomyia galactoptera Strobl, 1893 [Kahanpää and Winqvist 2005]
sg. ***Amydroneura*** Collin, 1926
Rhamphomyia gibba (Fallén, 1816) [Hackman 1980]
sg. ***Holoclera*** Schiner, 1860
Rhamphomyia bohemica Barták & Kubík, 2012 [Barták and Kubík 2012]
= *R. caliginosa* misid. [Hackman 1980]
= *R. trigemina* misid. [Kahanpää and Winqvist 2005]
Rhamphomyia culicina (Fallén, 1816) [Hackman 1980]
Rhamphomyia nigripennis (Fabricius, 1794) [Hackman 1980]
Rhamphomyia sciarina (Fallén, 1816) [Hackman 1980]
Rhamphomyia umbripennis Meigen, 1822 [Hackman 1980]
Rhamphomyia variabilis (Fallén, 1816)
= *R. tenuirostris* (Fallén, 1816) [Hackman 1980]
sg. ***Lundstroemiella*** Frey, 1922
Rhamphomyia dudai Oldenberg, 1927 [Hackman 1980]
Rhamphomyia hybotina Zetterstedt, 1838 [Hackman 1980]
sg. ***Megacyttarus*** Bigot, 1880
Rhamphomyia anomalina Zetterstedt, 1838 [Hackman 1980]
Rhamphomyia anomalipennis Meigen, 1822 [Hackman 1980]
= *R. anomala* misid.[Fre 1930, Hackman 1980]
Rhamphomyia crassirostris (Fallén, 1816)
= *R. nigripes* Bonsdorff, 1861 misid.[Hackman 1980]
Rhamphomyia gufitar Frey, 1922 [Hackman 1980]
Rhamphomyia maculipennis Zetterstedt, 1842 [Hackman 1980]
= *R. tephraea* misid.[Hackman 1980, Kahanpää and Winqvist 2005]
= *R. poissoni* misid.[Kahanpää and Winqvist 2005]
Rhamphomyia nodipes (Fallén, 1816) [Hackman 1980]
= *R. spissirostris* (Fallén, 1816)
Rhamphomyia paradoxa Wahlberg, 1844 [Hackman 1980]
sg. ***Pararhamphomyia*** Frey, 1922
Rhamphomyia albidiventris Strobl, 1898 [Hackman 1980]
= *R. woldstedti* Frey, 1913
Rhamphomyia albipennis (Fallén, 1816) [Hackman 1980]
Rhamphomyia albitarsis Collin, 1926 [Flinck and Kahanpää 2013]
Rhamphomyia albissima Frey, 1913 [Hackman 1980]
Rhamphomyia alpina Zetterstedt, 1838 [Hackman 1980]
Rhamphomyia amoena Loew, 1840 [Hackman 1980]
Rhamphomyia angulifera Frey, 1913 [Hackman 1980]
Rhamphomyia breviventris Frey, 1913 [Hackman 1980]
Rhamphomyia caesia Meigen, 1822
= *R. filata* Zetterstedt, 1842 [Hackman 1980]
Rhamphomyia caudata Zetterstedt, 1838 [Hackman 1980]
= *R. aethiops* Zetterstedt, 1838 [Kahanpää and Winqvist 2005]
Rhamphomyia chibinensis Frey, 1922 [Hackman 1980]

Rhamphomyia cribrata Oldenberg, 1927 [Hackman 1980]
Rhamphomyia curvula Frey, 1913 [Hackman 1980]
Rhamphomyia dentata Oldenberg, 1910 [Hackman 1980]
Rhamphomyia fascipennis Zetterstedt, 1838 [Hackman 1980]
Rhamphomyia filicaudula Frey, 1949 [Hackman 1980]
Rhamphomyia fuscipennis Zetterstedt, 1838 [Hackman 1980]
Rhamphomyia fuscula Zetterstedt, 1838 [Hackman 1980]
Rhamphomyia geniculata Meigen, 1830 [Hackman 1980]
= *R. plumipes* Pipping, 1858 misid.
Rhamphomyia griseola (Zetterstedt, 1838)
= *R. dispar* Zetterstedt, 1838 [Hackman 1980]
= *R. aperta* Zetterstedt, 1859 [Fre 1930, Hackman 1980]
Rhamphomyia helleni Frey, 1922 [Kahanpää and Winqvist 2005]
Rhamphomyia lividiventris Zetterstedt, 1838 [Hackman 1980]
Rhamphomyia longestylata Frey, 1916 [Frey 1927, Hackman 1980]
= *R. caudata* auct. nec Zetterstedt, 1838
Rhamphomyia lucidula Zetterstedt, 1842 [Hackman 1980]
Rhamphomyia marginata (Fabricius, 1787) [Hackman 1968a, 1980]
Rhamphomyia modesta Wahlberg, 1844 [Hackman 1980]
Rhamphomyia murina Collin, 1926 [Winqvist 2011]
Rhamphomyia niveipennis Zetterstedt, 1838 [Hackman 1980]
Rhamphomyia obscura Zetterstedt, 1838 [Hackman 1980]
Rhamphomyia obscuripennis Meigen, 1830
= *R. nitidicollis* Frey, 1913 [Hackman 1980]
Rhamphomyia physoprocta Frey, 1913 [Hackman 1980]
Rhamphomyia pilifer Meigen, 1838
= *R. dentipes* Zetterstedt, 1842 [Hackman 1980]
= *R. glaucella* Zetterstedt, 1842
= *R. intermedia* Frey, 1922 [Hackman 1980]
Rhamphomyia plumifera Zetterstedt, 1838 [Hackman 1980]
Rhamphomyia poplitea Wahlberg, 1844 [Hackman 1980]
Rhamphomyia praestans Frey, 1913 [Hackman 1980]
Rhamphomyia pusilla Zetterstedt, 1838 [Hackman 1980]
Rhamphomyia rufipes Zetterstedt, 1838
= *R. lapponica* Frey, 1955 [Hackman 1980]
Rhamphomyia simplex Zetterstedt, 1849 [Hackman 1980]
Rhamphomyia subglaucella Frey, 1922 [Hackman 1980]
Rhamphomyia tibiella Zetterstedt, 1842 [Hackman 1980]
Rhamphomyia tipularia (Fallén, 1816) [Hackman 1980]
Rhamphomyia unguiculata Frey, 1913 [Hackman 1980]
Rhamphomyia sp. aff.*albipennis* [Winqvist 2011]
sg. ***Rhamphomyia*** Meigen, 1822
Rhamphomyia albosegmentata Zetterstedt, 1838 [Hackman 1980]
Rhamphomyia cinerascens (Meigen, 1804) [Kahanpää and Winqvist 2005]
Rhamphomyia coracina Zetterstedt, 1849 [Hackman 1980]
Rhamphomyia dorsata Becker, 1915 [Hackman 1980]
= *R. phanerostigma* Frey, 1918
Rhamphomyia hambergi Frey, 1916 [Haarto and Winqvist 2014]
Rhamphomyia ignobilis Zetterstedt, 1859 [Hackman 1980]
= *R. attenuata* Frey, 1913

Rhamphomyia laevipes (Fallén, 1816) [Frey et al. 1941, Kahanpää and Winqvist 2005]
= *R. tephraea* Meigen, 1822
Rhamphomyia latifrons Frey, 1913 [Hackman 1980]
Rhamphomyia morio Zetterstedt, 1838 [Hackman 1980]
Rhamphomyia nitidula Zetterstedt, 1842 [Hackman 1980]
Rhamphomyia palmeni Frey, 1913 [Hackman 1980]
Rhamphomyia plumipes (Meigen, 1804) [Hackman 1980]
= *R. vespertilio* Zetterstedt, 1842
Rhamphomyia reflexa Zetterstedt, 1838 [Fre 1930, Hackman 1980]
Rhamphomyia spinipes (Fallén, 1816) [Hackman 1980]
Rhamphomyia stigmosa Macquart, 1827 [Hackman 1980]
= *R. conformis* misid.
Rhamphomyia sulcata (Meigen, 1804) [Hackman 1980]
= *R. tibialis* misid.
Rhamphomyia trilineata Zetterstedt, 1859 [Hackman 1980]
= *R. sulcatina* Collin, 1926 [Hackman 1980]
= *R. tibialis* misid.
= *R. propinqua* misid.
Rhamphomyia vesiculosa (Fallén, 1816) [Hackman 1980]
tribe Hilarini Collin, 1961
HILARA Meigen, 1822
Hilara abdominalis Zetterstedt, 1838
= *H. obscuritarsis* Zetterstedt, 1859 [Hackman 1980]
Hilara albitarsis von Roser, 1840 [Chvála 2005, Kahanpää and Winqvist 2005]
Hilara anglodanica Lundbeck, 1913
Hilara barbipes Frey, 1908 [Frey 1908, Hackman 1980]
Hilara beckeri Strobl, 1892 [Hackman 1980]
Hilara biseta Collin, 1927 [Chvála 2005, Kahanpää and Winqvist 2005]
Hilara bistriata Zetterstedt, 1842 [Hackman 1980]
Hilara brevistyla Collin, 1927 [Hackman 1980]
Hilara campinosensis Niesiolowski, 1986 [Chvála 2005, Kahanpää and Winqvist 2005]
Hilara canescens Zetterstedt, 1849 [Hackman 1980]
Hilara chorica (Fallén, 1816) [Hackman 1980]
Hilara clavipes (Harris, 1776)
= *H. spinimana* Zetterstedt, 1838 [Hackman 1980]
Hilara clypeata Meigen, 1822 [Hackman 1980]
Hilara coracina Oldenberg, 1916 [Chvála 2002b]
= *H. quadrifaria* Strobl, 1892 [Hackman 1980, Chvála 2002b]
Hilara cornicula Loew, 1873 [Hackman 1980]
Hilara discoidalis Lundbeck, 1910 [Hackman 1980]
Hilara diversipes Strobl, 1892
= *H. germanica* Engel, 1941 [Hackman 1980]
Hilara eviana Straka, 1976 [Chvála 2005]
Hilara femorella Zetterstedt, 1842 [Fre 1930, Chvála 1996]
Hilara gallica (Meigen, 1804) [Hackman 1980]
Hilara griseola Zetterstedt, 1838 [Hackman 1980]
Hilara hirta Strobl, 1892 [Chvála 2002a]
= *H. hirtella* misid.[Karppinen 1967, Hackman 1980, Chvála 2002a]
Hilara hybrida Collin, 1961 [Chvála 1996]
Hilara hyposeta Straka, 1976 [Chvála 2005, Kahanpää and Winqvist 2005]

Hilara implicata Collin, 1927 [Chvála 2005, Kahanpää and Winqvist 2005]
Hilara intermedia (Fallén, 1816)
= *H. pubipes* Loew, 1873 [Hackman 1980]
Hilara interstincta (Fallén, 1816) [Hackman 1980]
Hilara monedula Collin, 1927
= *H. longifurca* auct. nec Strobl, 1892 [Chvála 2005, Kahanpää and Winqvist 2005]
Hilara lapponica Chvála, 2002 [Chvála 2002b]
Hilara litorea (Fallén, 1816) [Hackman 1980]
Hilara longivittata Zetterstedt, 1842 [Hackman 1980]
Hilara lurida (Fallén, 1816) [Hackman 1980]
Hilara nigritarsis Zetterstedt, 1838
= *H. infans* Zetterstedt, 1842 [Hackman 1980]
Hilara nitidula Zetterstedt, 1838 [Hackman 1980]
Hilara pilipes Zetterstedt, 1838 [Hackman 1980]
Hilara pseudochorica Strobl, 1892 [Hackman 1980]
Hilara pulchripes Frey, 1913 [Hackman 1980]
Hilara quadrifasciata Chvála, 2002 [Chvála 2002a]
= *H. quadrivittata* auct. nec Meigen, 1822 [Hackman 1980, Chvála 2002a]
Hilara sturmii Meigen, 1822 [Chvála 2005]
= *H. cingulata* misid.[Hackman 1980]
Hilara submaura Collin, 1927 [Chvála 1996]
Hilara tanythrix Frey, 1913 [Hackman 1980]
Hilara tenuinervis Zetterstedt, 1838 [Hackman 1980]
Hilara woodiella Chvála, 1999 [Chvála 2005, Kahanpää and Winqvist 2005]
CLINOCERINAE Collin, 1928
CLINOCERA Meigen, 1803
Clinocera appendiculata (Zetterstedt, 1838) [Hackman 1980, Sinclair 1999]
= *C. simplicinervis* Frey, 1913
Clinocera aucta (Zetterstedt, 1849) [Sinclair 1999]
Clinocera nivalis (Zetterstedt, 1838) [Hackman 1980]
Clinocera stagnalis (Haliday, 1833) [Hackman 1980]
Clinocera wesmaeli (Macquart, 1835) [Hackman 1980]
DOLICHOCEPHALA Macquart, 1823
Dolichocephala guttata (Haliday, 1833) [Kahanpää 2013d]
Dolichocephala irrorata (Fallén, 1815) [Hackman 1980]
Dolichocephala thomasi Wagner, 1983
= *D. ocellata* misid.[Hackman 1980]
WIEDEMANNIA Zetterstedt, 1838
sg. ***Eucelidia*** Mik, 1881
Wiedemannia zetterstedti (Fallén, 1826) [Hackman 1980]
sg. ***Philolutra*** Mik, 1881
Wiedemannia bohemani (Zetterstedt, 1838) [Hackman 1980]
Wiedemannia simplex (Loew, 1862)
= *W. fallaciosa* misid.[Hackman 1980]
sg. ***Wiedemannia*** Zetterstedt, 1838
Wiedemannia bistigma (Curtis, 1834) [Hackman 1980]
Uncertain subfamily position within Empididea (incertae sedis)
HORMOPEZA Zetterstedt, 1838
Hormopeza copulifera Melander, 1927 [Hackman 1980, Tuomikoski 1960a]
Hormopeza obliterata Zetterstedt, 1838 [Hackman 1980, Tuomikoski 1960a]

RAGAS Walker, 1837
Ragas unica Walker, 1837 [Hackman 1980]

Excluded species

Chelifera astigma Collin, 1927 [Hackman 1980, Kahanpää and Winqvist 2005]
Clinocera nigra Meigen, 1804 [Hackman 1980, Kahanpää and Winqvist 2005]
Empis punctata Meigen, 1804 [Chvála 1994, Kahanpää and Winqvist 2005]
Hilara flavipes Meigen, 1822
Hilara maura (Fabricius, 1776) [Hackman 1980]
Hilara scrobiculata Loew, 1873 [Hackman 1980, Kahanpää and Winqvist 2005]
Hilara tetragramma Loew, 1873 [Hedström 1964, Hackman 1980, Kahanpää and Winqvist 2005]
Rhamphomyia tibialis Meigen, 1822 [Hackman 1980, Kahanpää and Winqvist 2005]
Trichoclinocera lapponica (Ringdahl, 1933) [Chvála and Wagner 1989]

ATELESTIDAE Hennig, 1970

Supporting references for *Kahanpää J (2014) Checklist of the Empidoidea of Finland (Insecta, Diptera). In: Kahanpää J, Salmela J (Eds) Checklist of the Diptera of Finland. ZooKeys @@: @-@. doi: 10.3897/zookeys.??.7154*

ATELESTINAE Hennig, 1970
ATELESTUS Walker, 1837
Atelestus pulicarius (Fallén, 1816) [Hackman 1980]
MEGHYPERUS Loew, 1850
Meghyperus sudeticus Loew, 1850 [Chvála 1983]

HYBOTIDAE Meigen, 1820

Supporting references for *Kahanpää J (2014) Checklist of the Empidoidea of Finland (Insecta, Diptera). In: Kahanpää J, Salmela J (Eds) Checklist of the Diptera of Finland. ZooKeys @@: @-@. doi: 10.3897/zookeys.??.7154*

TRICHININAE Chvála, 1983
TRICHINA Meigen, 1830
Trichina bilobata Collin, 1926 [Hackman 1980, Tuomikoski 1935]
Trichina clavipes Meigen, 1830 [Hackman 1980]
Trichina elongata Haliday, 1833 [Hackman 1980]
Trichina opaca Loew, 1864 [Hackman 1980]
= *T. picipes* Tuomikoski, 1935
Trichina pallipes (Zetterstedt, 1838) [Hackman 1980, Tuomikoski 1935]
TRICHINOMYIA Tuomikoski, 1959 [Tuomikoski 1959]
Trichinomyia flavipes (Meigen, 1830) [Hackman 1980]
Trichinomyia fuscipes (Zetterstedt, 1838) [Hackman 1980, Tuomikoski 1935]
OCYDROMIINAE Schiner, 1862
tribe Ocydromiini Schiner, 1862
CHVALAEA Papp & Földvári, 2001
Chvalaea sopianae Papp & Földvári, 2001 [Kahanpää 2013b]
LEPTODROMIELLA Tuomikoski, 1936
Leptodromiella crassiseta Tuomikoski, 1936 [Tuomikoski 1936, Hackman 1980]
LEPTOPEZA Macquart, 1834
Leptopeza borealis Zetterstedt, 1842 [Hackman 1980]
Leptopeza flavipes (Meigen, 1820) [Hackman 1980]
OCYDROMIA Meigen, 1820
Ocydromia glabricula (Fallén, 1816) [Hackman 1980, Tuomikoski 1937]
Ocydromia melanopleura Loew, 1840 [Hackman 1980, Tuomikoski 1937]
OEDALEINAE Chvála, 1983
ALLANTHALIA Melander, 1927
Allanthalia pallida (Zetterstedt, 1838) [Mee 1927, Hackman 1980]
ANTHALIA Zetterstedt, 1838
Anthalia schoenherri Zetterstedt, 1838 [Hackman 1980]
EUTHYNEURA Macquart, 1836
Euthyneura albipennis (Zetterstedt, 1842) [Hackman 1980]
Euthyneura gyllenhali (Zetterstedt, 1838) [Hackman 1980]
Euthyneura myrtilli Macquart, 1836 [Hackman 1980]
= *E. myricae* Haliday, 1851 misid.[Hackman 1980, Chvála 1983]
OEDALEA Meigen, 1820
Oedalea freyi Chvála, 1983 [Chvála 1983]
Oedalea holmgreni Zetterstedt, 1852 [Hackman 1980]
Oedalea hybotina (Fallén, 1816) [Hackman 1980]
Oedalea stigmatella Zetterstedt, 1842 [Hackman 1980]
Oedalea tibialis Macquart, 1827 [Flinck and Kahanpää 2013]
Oedalea zetterstedti Collin, 1926 [Chvála 1983]
TACHYDROMIINAE Meigen, 1822
tribe Symballophthalmini Sinclair & Cumming, 2006
SYMBALLOPHTHALMUS Mecker, 1889
Symballophthalmus dissimilis (Fallén, 1815) [Hackman 1980]

Symballophthalmus fuscitarsis (Zetterstedt, 1859) [Hackman 1980]
= *S. scapularis* Collin, 1961
tribe Tachydromiini Meigen, 1822
PLATYPALPUS Macquart, 1827
Platypalpus agilis (Meigen, 1822) [Hackman 1980]
Platypalpus albicornis (Zetterstedt, 1842) [Chvála 1975]
Platypalpus albiseta (Panzer, 1806) [Hackman 1980]
Platypalpus albocapillatus (Fallén, 1815) [Hackman 1980]
Platypalpus alpinus Chvála, 1971 [Chvála 1971, Hackman 1980]
Platypalpus alter (Collin, 1961) [Chvála 1975, Hackman 1980]
Platypalpus annulatus (Fallén, 1815) [Hackman 1980]
= *P. fulvipes* (Meigen, 1822)
Platypalpus annulipes (Meigen, 1822) [Kahanpää and Winqvist 2003]
Platypalpus articulatoides (Frey, 1918) [Hackman 1980]
? *Platypalpus articulatus* Macquart, 1827 [Hackman 1980]
Platypalpus ater (Wahlberg, 1844) [Hackman 1980]
= *P. atra* misspelling
Platypalpus boreoalpinus Frey, 1943 [Hackman 1980]
Platypalpus brachystylus (Bezzi, 1892)
= *P. brunneitibia* (Strobl, 1899) [Hackman 1980]
Platypalpus brevicornis (Zetterstedt, 1842) [Hackman 1980]
Platypalpus calceatus (Meigen, 1822) [Hackman 1980]
Platypalpus candicans (Fallén, 1815) [Hackman 1980]
Platypalpus ciliaris (Fallén, 1816) [Hackman 1980]
Platypalpus confiformis Chvála, 1971 [Chvála 1971, Hackman 1980]
Platypalpus confinis (Zetterstedt, 1842) [Hackman 1980]
Platypalpus cothurnatus Macquart, 1827 [Hackman 1980]
Platypalpus cryptospina (Frey, 1909) [Hackman 1980]
= *P. tantulus* (Collin, 1961)
Platypalpus cursitans (Fabricius, 1775) [Hackman 1980]
= *P. bicolor* (Meigen, 1804)
Platypalpus ecalceatus (Zetterstedt, 1838) [Hackman 1980]
Platypalpus excavatus Yang & Yao, 2007
= *P. excisus* (Becker, 1907) preocc.
Platypalpus exilis (Meigen, 1822) [Hackman 1980]
Platypalpus fenestella Kovalev, 1971 [Chvála 1975, Hackman 1980]
Platypalpus flavicornis (Meigen, 1822) [Hackman 1980]
Platypalpus fuscicornis (Zetterstedt, 1842) [Kahanpää and Winqvist 2005]
Platypalpus hackmani Chvála, 1972 [Chvála 1972, Hackman 1980]
Platypalpus infectus (Collin, 1926) [Hackman 1980]
Platypalpus interstinctus (Collin, 1926) [Hackman 1980]
Platypalpus laestadianorum (Frey, 1913) [Hackman 1980]
Platypalpus lapponicus Frey, 1943 [Hackman 1980]
Platypalpus longicornis (Meigen, 1822) [Hackman 1980]
Platypalpus longiseta (Zetterstedt, 1842) [Chvála 1975, Hackman 1980]
= *P. extricatus* (Collin, 1926)
Platypalpus luteicornis (Meigen, 1838)
= *P. difficilis* (Frey, 1907) [Hackman 1980]
= *P. interjectus* (Lundbeck, 1910)
Platypalpus luteus (Meigen, 1804) [Hackman 1980]

Platypalpus maculus (Zetterstedt, 1842) [Hackman 1980]
Platypalpus maculimanus (Zetterstedt, 1842)
Platypalpus maculipes (Meigen, 1822) [Hackman 1980]
Platypalpus major (Zetterstedt, 1842) [Hackman 1980]
Platypalpus melancholicus (Collin, 1961) [Chvála 1975, Hackman 1980]
Platypalpus minutus (Meigen, 1804) [Hackman 1980]
Platypalpus nigricoxa (Mik, 1884) [Hackman 1980]
Platypalpus nigritarsis (Fallén, 1816) [Hackman 1980]
Platypalpus nigrosetosus (Strobl, 1893) [Hackman 1980]
Platypalpus nonstriatus Strobl, 1901 [Hackman 1980]
Platypalpus notatus (Meigen, 1822) [Hackman 1980]
Platypalpus pallidicornis (Collin, 1926) [Chvála 1975, Hackman 1980]
Platypalpus pallidicoxa (Frey, 1913) [Hackman 1980]
Platypalpus pallidiventris (Meigen, 1822) [Hackman 1980]
= *P. flavipes* (Fabricius, 1794)
Platypalpus pallipes (Fallén, 1815) [Hackman 1980]
Platypalpus pectoralis (Fallén, 1815) [Hackman 1980]
Platypalpus pseudofulvipes (Frey, 1909)
= *P. coarctatus* (Collin, 1926) [Hackman 1980]
Platypalpus pseudorapidus Kovalev, 1971 [Chvála 1975, Hackman 1980]
Platypalpus pulicarius (Meigen, 1830) [Hackman 1980]
Platypalpus rapidus (Meigen, 1822) [Hackman 1980]
Platypalpus sahlbergi (Frey, 1909) [Hackman 1980]
Platypalpus scandinavicus Chvála, 1972 [Mukkala et al. 2005, Kahanpää and Winqvist 2005]
Platypalpus sordidus (Zetterstedt, 1838) [Hackman 1980]
Platypalpus stabilis (Collin, 1961) [Chvála 1975, Hackman 1980]
Platypalpus stackelbergi Kovalev, 1971 [Chvála 1989]
Platypalpus stigmatellus (Zetterstedt, 1842) [Hackman 1980]
Platypalpus strigifrons (Zetterstedt, 1849) [Hackman 1980]
Platypalpus subbrevis (Frey, 1913) [Hackman 1980]
Platypalpus subtilis (Collin, 1926)
Platypalpus sylvicola (Collin, 1926) [Hackman 1980]
Platypalpus tuomikoskii Chvála, 1972 [Chvála 1972, Hackman 1980]
Platypalpus unguiculatus (Zetterstedt, 1838) [Hackman 1980]
Platypalpus verralli (Collin, 1926) [Hackman 1980]
Platypalpus vividus (Meigen, 1838)
= *P. albisetoides* Chvála, 1973 [Chvála 1973, Hackman 1980]
Platypalpus zetterstedti Chvála, 1971 [Hackman 1980]
TACHYDROMIA Meigen, 1803
Tachydromia aemula (Loew, 1864) [Chvála 1975, Hackman 1980]
Tachydromia arrogans (Linnaeus, 1761) [Hackman 1980]
Tachydromia connexa Meigen, 1822 [Kahanpää 2013d]
Tachydromia incompleta (Becker, 1900) [Chvála 1975, Hackman 1980]
Tachydromia lundstroemi (Frey, 1913) [Hackman 1980]
Tachydromia morio (Zetterstedt, 1838) [Chvála 1975, Hackman 1980]
Tachydromia punctifera (Becker, 1900) [Chvála 1975, Hackman 1980]
Tachydromia sabulosa Meigen, 1830 [Hackman 1980]
Tachydromia terricola Zetterstedt, 1819 [Hackman 1980]
Tachydromia umbrarum Haliday, 1833 [Hackman 1980]

TACHYPEZA Meigen, 1830
Tachypeza fennica Tuomikoski, 1932 [Hackman 1980]
Tachypeza fuscipennis (Fallén, 1815) [Hackman 1980]
Tachypeza heeri (Zetterstedt, 1838) [Hackman 1980]
Tachypeza nubila (Meigen, 1804) [Hackman 1980]
Tachypeza truncorum (Fallén, 1815) [Hackman 1980]
Tachypeza winthemi (Zetterstedt, 1838) [Hackman 1980]
tribe Drapetini Collin, 1961
CHERSODROMIA Haliday, 1851
Chersodromia arenaria (Haliday, 1833) [Hackman 1980]
Chersodromia cursitans (Zetterstedt, 1819) [Hackman 1980]
CROSSOPALPUS Bigot, 1857
Crossopalpus curvinervis (Zetterstedt, 1842) [Hackman 1980]
Crossopalpus curvipes (Meigen, 1822) [Chvála 1975, Hackman 1980]
Crossopalpus humilis (Frey, 1913) [Hackman 1980]
Crossopalpus nigritellus (Zetterstedt, 1842) [Hackman 1980]
Crossopalpus setiger (Loew, 1859) [Hackman 1980]
DRAPETIS Meigen, 1822
Drapetis arcuata Loew, 1859 [Hackman 1980]
Drapetis assimilis (Fallén, 1815) [Hackman 1980]
Drapetis exilis Meigen, 1822 [Hackman 1980]
Drapetis infitialis Collin, 1961 [Chvála 1975, Hackman 1980]
Drapetis ingrica Kovalev, 1972 [Chvála 1975, Hackman 1980]
Drapetis parilis Collin, 1926 [Hackman 1980]
Drapetis pusilla Loew, 1859 [Hackman 1980]
Drapetis simulans Collin, 1961 [Chvála 1975, Hackman 1980]
ELAPHROPEZA Macquart, 1827
Elaphropeza ephippiata (Fallén, 1815) [Hackman 1980]
STILPON Loew, 1859
Stilpon graminum (Fallén, 1815) [Hackman 1980]
HYBOTINAE Meigen, 1820
tribe Bicellariini Sinclair & Cumming, 2006
BICELLARIA Macquart, 1823
= ***Cyrtoma*** Meigen, 1824
Bicellaria austriaca Tuomikoski, 1955 [Chvála 1983, Chvála and Biström 1991]
Bicellaria intermedia Lundbeck, 1910 [Hackman 1980]
Bicellaria nigra (Meigen, 1824) [Hackman 1980]
Bicellaria pilosa Lundbeck, 1910 [Hackman 1980]
Bicellaria simplicipes (Zetterstedt, 1842) [Hackman 1980]
Bicellaria spuria (Fallén, 1816) [Hackman 1980]
Bicellaria subpilosa Collin, 1926 [Hackman 1980]
Bicellaria sulcata (Zetterstedt, 1842) [Hackman 1980]
Bicellaria uvens Melander, 1928
= *B. bisetosa* Tuomikoski, 1936 [Hackman 1980]
tribe Hybotini Meigen, 1820
HYBOS Meigen, 1803
Hybos culiciformis (Fabricius, 1775) [Hackman 1980]
Hybos femoratus (Müller, 1776)
= *H. femoralis* misspelling [Hackman 1980]
Hybos grossipes (Linnaeus, 1767) [Hackman 1980]

SYNDYAS Loew, 1857
Syndyas nigripes (Zetterstedt, 1842) [Hackman 1980]

Excluded species

Crossopalpus abditus Kovalev, 1972 [Chvála 1975, Kahanpää and Winqvist 2005]
Drapetis incompleta Collin, 1926 [Chvála and Kovalev 1989, Kahanpää and Winqvist 2005]
Oedalea flavipes Zetterstedt, 1842 [Mee 1927, Chvála 1983]

BRACHYSTOMATIDAE Melander, 1908

Supporting references for *Kahanpää J (2014) Checklist of the Empidoidea of Finland (Insecta, Diptera). In: Kahanpää J, Salmela J (Eds) Checklist of the Diptera of Finland. ZooKeys @@: @-@. doi: 10.3897/zookeys.??.7154*

TRICHOPEZINAE Vaillant, 1981
GLOMA Meigen, 1822
Gloma fuscipennis Meigen, 1822 [Hackman 1980]
HELEODROMIA Haliday, 1833
sg. ***Heleodromia*** Haliday, 1833
Heleodromia immaculata Haliday, 1833 [Hackman 1980]
TRICHOPEZA Rondani, 1856
Trichopeza albocincta (Boheman, 1863) [Hackman 1980]
= *T. albicincta* Frey, 1913
Trichopeza longicornis (Meigen, 1822) [Hackman 1980]

DOLICHOPODIDAE Latreille, 1809

Supporting references for *Kahanpää J (2014) Checklist of the Empidoidea of Finland (Insecta, Diptera). In: Kahanpää J, Salmela J (Eds) Checklist of the Diptera of Finland. ZooKeys @@: @-@. doi: 10.3897/zookeys.??.7154*

MICROPHORINAE Collin, 1960
MICROPHOR Macquart, 1827
Microphor anomalus (Meigen, 1824) [Kahanpää and Winqvist 2005]
Microphor crassipes Macquart, 1827 [Chvála 1983]
= *M. anomalus* misid.[Frey 1913, Hackman 1980]
Microphor holosericeus (Meigen, 1804)
= *M. velutinus* Macquart, 1827 [Hackman 1980]
PARATHALASSIINAE Chvála, 1981
MICROPHORELLA Becker, 1909
Microphorella praecox (Loew, 1864) [Hackman 1980, Kahanpää 2013a]
DOLICHOPODINAE Latreille, 1809
DOLICHOPUS Latreille, 1796
= ***Hygroceleuthus*** Loew, 1857
sg. ***Dolichopus*** Latreille, 1796
Dolichopus acuticornis Wiedemann, 1817 [Hackman 1980]
Dolichopus annulitarsis Ringdahl, 1920 [Kahanpää 2013a, Haarto and Winqvist 2014]
Dolichopus apicalis Zetterstedt, 1849 [Kahanpää and Grichanov 2006]
Dolichopus argyrotarsis Wahlberg, 1850 [Frey 1915, Hackman 1980]
Dolichopus armillatus Wahlberg, 1850 [Frey 1915, Hackman 1980]
= *D. stenhammari* var. b Zetterstedt, 1843
Dolichopus atripes Meigen, 1824 [Frey 1915, Hackman 1980]
Dolichopus austriacus Parent, 1927 [Haarto 2002b]
Dolichopus bonsdorffi Frey, 1915 [Frey 1915, Hackman 1980]
Dolichopus brevipennis Meigen, 1824 [Hackman 1980]
Dolichopus caligatus Wahlberg, 1850 [Hackman 1980]
= *D. flavipes* misid.
= *D. albifrons* misid.[Kahanpää and Grichanov 2006, Kahanpää 2006]
Dolichopus calinotus Loew, 1871 [Kahanpää and Grichanov 2006]
Dolichopus campestris Meigen, 1824 [Frey 1915, Hackman 1980]
Dolichopus cilifemoratus Macquart, 1827 [Kahanpää 2013a]
= *D. pseudocilifemoratus* Stackelberg, 1930
Dolichopus cinctipes Wahlberg, 1850 [Kahanpää and Grichanov 2006]
Dolichopus claviger Stannius, 1831 [Hackman 1980]
Dolichopus clavipes Haliday, 1832 [Kahanpää and Grichanov 2006]
Dolichopus costalis Frey, 1915 [Kahanpää 2013a]
Dolichopus discifer Stannius, 1831 [Hackman 1980]
? = *D. nigricornis* Meigen, 1824
Dolichopus discimanus Wahlberg, 1851 [Frey 1915, Hackman 1980]
Dolichopus fraterculus Zetterstedt, 1843 [Frey 1915, Hackman 1980]
Dolichopus griseipennis Stannius, 1831 [Hackman 1980]
Dolichopus gubernator Mik, 1878 [Hackman 1980, Fre 1935]
Dolichopus hilaris Loew, 1862 [Kahanpää 2006]
Dolichopus lancearius Hedström, 1966 [Kahanpää 2013a]
Dolichopus latilimbatus Macquart, 1827 [Kahanpää and Grichanov 2006]

Dolichopus latipennis Fallén, 1823 [Frey 1915, Hackman 1980]
Dolichopus lepidus Staeger, 1842 [Hackman 1980]
= *D. cruralis* Wahlberg, 1850 [Frey 1915, Hackman 1980, Kahanpää 2008]
= *D. lapponicus* Becker, 1917
Dolichopus linearis Meigen, 1824 [Frey 1915, Hackman 1980]
Dolichopus lineatocornis Zetterstedt, 1843 [Hackman 1980]
Dolichopus longicornis Stannius, 1831 [Hackman 1980]
Dolichopus longitarsis Stannius, 1831 [Hackman 1980]
Dolichopus maculipennis Zetterstedt, 1843 [Hackman 1980]
Dolichopus mannerheimi Zetterstedt, 1838 [Hackman 1980]
Dolichopus migrans Zetterstedt, 1843 [Hackman 1980]
Dolichopus nigripes Fallén, 1823 [Kahanpää 2013a]
Dolichopus nitidus Fallén, 1823 [Hackman 1980]
Dolichopus notatus Staeger, 1842 [Hackman 1980]
= *D. notabilis* Zetterstedt, 1843
Dolichopus nubilus Meigen, 1824 [Hackman 1980]
Dolichopus pennatus Meigen, 1824 [Hackman 1980]
Dolichopus picipes Meigen, 1824 [Hackman 1980]
= *D. consimilis* Wahlberg, 1850 [Hackman 1980, Kahanpää 2008]
Dolichopus planitarsis Fallén, 1823 [Hackman 1980]
Dolichopus plumipes (Scopoli, 1763) [Hackman 1980]
= *D. parvicaudatus* Zetterstedt, 1843 [Frey 1915, Hackman 1980, Kahanpää 2008]
= *D. pectinitarsis* Stenhammar, 1851 [Hackman 1980, Kahanpää 2008]
Dolichopus popularis Wiedemann, 1817 [Frey 1915, Hackman 1980]
Dolichopus pseudomigrans Ringdahl, 1928 [Hackman 1980]
Dolichopus punctum Meigen, 1824 [Hackman 1980]
Dolichopus remipes Wahlberg, 1839 [Hackman 1980]
Dolichopus rupestris Haliday, 1833 [Frey 1915, Hackman 1980]
Dolichopus ruthei Loew, 1847 [Hackman 1980]
Dolichopus sabinus Haliday, 1838 [Frey 1915, Hackman 1980]
Dolichopus setiger Negrobov, 1973 [Kahanpää and Negrobov 2007]
Dolichopus signatus Meigen, 1824 [Hackman 1980]
Dolichopus signifer Haliday, 1832 [Kahanpää and Grichanov 2006]
Dolichopus simplex Meigen, 1824 [Hackman 1980]
Dolichopus stenhammari Zetterstedt, 1843
= *D. annulipes* Zetterstedt, 1838 [Hackman 1980] invalidated
Dolichopus subpennatus d'Assis-Fonseca, 1976 [Kahanpää and Grichanov 2006]
Dolichopus trivialis Haliday, 1832
= *D. cilifemoratus* auct. nec Macquart, 1827 [Hackman 1980]
Dolichopus ungulatus (Linnaeus, 1758) [Hackman 1980]
Dolichopus urbanus Meigen, 1824 [Hackman 1980]
Dolichopus vitripennis Meigen, 1824 [Hackman 1980]
Dolichopus wahlbergi Zetterstedt, 1843 [Hackman 1980]
Dolichopus zetterstedti Stenhammar, 1852 [Frey 1915, Hackman 1980]
sg. ***Macrodolichopus*** Stackelberg, 1933
Dolichopus diadema Haliday, 1832 [Frey 1915, Hackman 1980]
ETHIROMYIA Brooks, 2005 [Brooks and Wheeler 2005]
Ethiromyia chalybeus (Wiedemann, 1817) [Frey 1915, Hackman 1980]
GYMNOPTERNUS Loew, 1857
Gymnopternus aerosus (Fallén, 1823) [Hackman 1980]

Gymnopternus angustifrons (Staeger, 1842) [Hackman 1980]
Gymnopternus brevicornis (Staeger, 1842) [Frey 1915, Hackman 1980]
Gymnopternus celer (Meigen, 1824) [Frey 1915, Hackman 1980]
Gymnopternus metallicus (Stannius, 1831) [Hackman 1980]
HERCOSTOMUS Loew, 1857
Hercostomus germanus (Wiedemann, 1817) [Hackman 1980]
Hercostomus nigrilamellatus (Macquart, 1827) [Winqvist 2011]
Hercostomus nigriplantis (Stannius, 1831) [Kahanpää 2006]
Hercostomus sahlbergi (Zetterstedt, 1838) [Frey 1915, Hackman 1980]
SYBISTROMA Meigen, 1824
Sybistroma discipes (Germar, 1817) [Kahanpää 2013a]
Sybistroma obscurellum (Fallén, 1823) [Kahanpää 2006]
TACHYTRECHUS Haliday, 1851
= ***Ammobates*** Stannius, 18312 preocc.
Tachytrechus ammobates (Haliday, 1851)
= *T. plumipes* (Fallén, 1823) preocc.[Hackman 1980]
Tachytrechus hamatus Loew, 1871 [Kahanpää and Grichanov 2006]
Tachytrechus notatus (Stannius, 1831) [Frey 1915, Hackman 1980]
SCIAPODINAE Becker, 1917
SCIAPUS Zeller, 1842
Sciapus albifrons (Meigen, 1830) [Kahanpää and Grichanov 2006]
Sciapus basilicus Meuffels & Grootaert, 1990 [Kahanpää and Grichanov 2006]
Sciapus lobipes (Meigen, 1824) [Kahanpää and Grichanov 2006]
Sciapus longulus (Fallén, 1823) [Frey 1915, Hackman 1980]
Sciapus maritimus Becker, 1918 [Hackman 1980]
= *S. flavomaculatus* Ringdahl, 1949
Sciapus platypterus (Fabricius, 1805) [Hackman 1980]
Sciapus wiedemanni (Fallén, 1823)
Sciapus zonatulus (Zetterstedt, 1843) [Kahanpää and Grichanov 2006]
= *S. contristans* misid.[Haarto 1997]
SYMPYCNINAE Aldrich, 1905
CAMPSICNEMUS Haliday, 1851
= ***Ectomus*** Mik, 1878
Campsicnemus alpinus (Haliday, 1833) [Hackman 1980]
Campsicnemus armatus (Zetterstedt, 1849) [Hackman 1980]
Campsicnemus articulatellus (Zetterstedt, 1843)
= *C. pilosellus* (Zetterstedt, 1843) [Frey 1915, Hackman 1980]
= *C. dasycnemus* Loew, 1857 [Hackman 1980]
Campsicnemus compeditus Loew, 1857 [Frey 1915, Hackman 1980]
Campsicnemus curvipes (Fallén, 1823) [Hackman 1980]
Campsicnemus femoratus Ringdahl, 1949 [Kahanpää 2013a]
Campsicnemus loripes (Haliday, 1832) [Hackman 1980]
Campsicnemus lumbatus Loew, 1857 [Hellén 1946]
Campsicnemus marginatus Loew, 1857 [Hackman 1980]
Campsicnemus paradoxus (Wahlberg, 1844) [Frey 1915, Hackman 1980]
Campsicnemus picticornis (Zetterstedt, 1843) [Frey 1915, Hackman 1980]
Campsicnemus pumilio (Zetterstedt, 1843) [Hackman 1980]
= *C. pectinulatus* Loew, 1864 [Hackman 1980]
Campsicnemus pusillus (Meigen, 1824) [Frey 1915, Hackman 1980]
Campsicnemus scambus (Fallén, 1823) [Hackman 1980]

LAMPROCHROMUS Mik, 1878
Lamprochromus strobli Parent, 1925 [Kahanpää and Grichanov 2006]
SYMPYCNUS Loew, 1857
Sympycnus aeneicoxa (Meigen, 1824) [Frey 1915, Hackman 1980]
Sympycnus pulicarius (Fallén, 1823)
? = *S. annulipes* (Meigen, 1824) [Hackman 1980]
? = *S. desoutteri* Parent, 1925
SYNTORMON Loew, 1857
= ***Bathycranium*** Strobl, 1892
Syntormon bicolorellus (Zetterstedt, 1843) [Frey 1915, Hackman 1980]
Syntormon filiger Verrall, 1912 [Ano 1960, Hackman 1980]
= *S. rufipes* misid.
= *S. simplicipes* Frey, 1915 [Frey 1915]
Syntormon freymuthae Loew, 1873 [Kahanpää 2006]
= *S. denticulatus* misid.[Frey 1915, Haarto et al. 2002, Haarto 2002b, Kahanpää 2006]
Syntormon metathesis (Loew, 1850) [Hackman 1980]
Syntormon pallipes (Fabricius, 1794) [Hackman 1980]
Syntormon pumilus (Meigen, 1824) [Frey 1915, Hackman 1980]
Syntormon tarsatus (Fallén, 1823) [Hackman 1980]
= *S. aulicus* misid. [Kahanpää and Grichanov 2006, Kahanpää 2006]
TELMATURGUS Mik, 1874
Telmaturgus tumidulus (Raddatz, 1873) [Fre 1935, Kahanpää and Grichanov 2006]
TEUCHOPHORUS Loew, 1857
= ***Teucophorus*** misspelling
Teuchophorus monacanthus Loew, 1859 [Kahanpää and Salmela 2007]
Teuchophorus nigricosta (von Roser, 1840)
= *T. pectinifer* Kowarz, 1868 [Frey 1915, Hackman 1980]
= *T. signatus* Zetterstedt, 1849
Teuchophorus spinigerellus (Zetterstedt, 1843) [Hackman 1980]
DIAPHORINAE Schiner, 1864
ARGYRA Macquart, 1834
= ***Leucostola*** Loew, 1857
Argyra argentina (Meigen, 1824) [Hackman 1980]
Argyra argyria (Meigen, 1824) [Hackman 1980]
Argyra auricollis (Meigen, 1824) [Frey 1915, Hackman 1980]
Argyra diaphana (Fabricius, 1775) [Hackman 1980]
Argyra elongata (Zetterstedt, 1843) [Frey 1915, Hackman 1980]
Argyra ilonae Geoffries, 1989 [Kahanpää and Grichanov 2006]
= *A. confinis* (Zetterstedt, 1849) preocc.
Argyra leucocephala (Meigen, 1824) [Hackman 1980]
Argyra magnicornis (Zetterstedt, 1838) [Hackman 1980]
Argyra setulipes Becker, 1918 [Kahanpää 2013a]
Argyra setimana Loew, 1859 [Kahanpää and Grichanov 2006]
= *A. subarctica* misid.[Hackman 1980]
Argyra spoliata Kowarz, 1879 [Frey 1915, Hackman 1980]
Argyra vestita (Wiedemann, 1817) [Frey 1915, Hackman 1980]
ASYNDETUS Loew, 1869
Asyndetus latifrons (Loew, 1857) [Kahanpää 2013a]
CHRYSOTUS Meigen, 1824
Chrysotus angulicornis Kowarz, 1874 [Kahanpää and Grichanov 2006]

Chrysotus cilipes Meigen, 1824 [Frey 1915, Hackman 1980]
Chrysotus cupreus (Macquart, 1827) [Kahanpää 2013a]
Chrysotus femoratus Zetterstedt, 1843 [Frey 1915]
Chrysotus gramineus (Fallén, 1823) [Hackman 1980]
= *C. microcerus* Kowarz, 1874
= *C. varians* Kowarz, 1874
Chrysotus laesus (Wiedemann, 1817) [Hackman 1980]
= *C. amplicornis* Zetterstedt, 1849 [Hackman 1980]
Chrysotus neglectus (Wiedemann, 1817) [Hackman 1980]
Chrysotus obscuripes Zetterstedt, 1838
= *C. kowarzi* Lundbeck, 1912 [Frey 1915, Hackman 1980]
Chrysotus pulchellus Kowarz, 1874 [Frey 1915, Hackman 1980]
Chrysotus suavis Loew, 1857 [Frey 1927, Hackman 1980]
DIAPHORUS Meigen, 1824
Diaphorus hoffmannseggi Meigen, 1830 [Frey 1915, Kahanpää and Grichanov 2006]
Diaphorus nigricans Meigen, 1824 [Hackman 1980]
Diaphorus oculatus (Fallén, 1823) [Hackman 1980]
MELANOSTOLUS Kowarz, 1884
Melanostolus melancholicus (Loew, 1869) [Kahanpää 2006]
MEDETERINAE Lioy, 1864
DOLICHOPHORUS Lichtward, 1902
Dolichophorus kerteszi Lichtward, 1902 [Kahanpää and Winqvist 2003]
MEDETERA Fischer von Waldheim, 1819
Medetera abstrusa Thuneberg, 1955 [Hackman 1980]
Medetera acanthura Negrobov & Thuneberg, 1970 [Negrobov and Thuneberg 1970, Hackman 1980]
Medetera adjaniae Gosseries, 1989
= *M. breviseta* Parent, 1927 preocc.
Medetera ambigua (Zetterstedt, 1843) [Hackman 1980]
Medetera apicalis (Zetterstedt, 1843) [Hackman 1980]
Medetera belgica Parent, 1936 sensu Negrobov & Stackelberg, 1972 [Kahanpää 2013a]
Medetera betulae Ringdahl, 1949 [Hackman 1980]
Medetera borealis Thuneberg, 1955 [Hackman 1980]
Medetera cuspidata Collin, 1941 [Kangas 1949, Hackman 1980]
Medetera dichrocera Kowarz, 1877 [Hackman 1980]
Medetera excellens Frey, 1909 [Hackman 1980]
= *M. thunebergi* Negrobov, 1967 [Thuneberg 1975]
Medetera fasciata Frey, 1915 [Frey 1915, Hackman 1980]
Medetera freyi Thuneberg, 1955 [Hackman 1980]
Medetera fumida Negrobov, 1967 [Kahanpää 2013a]
Medetera impigra Collin, 1941 [Hackman 1980]
Medetera incrassata Frey, 1909 [Hackman 1980]
Medetera infumata Loew, 1857 [Hackman 1980]
Medetera inspissata Collin, 1952 [Hackman 1980]
Medetera jacula (Fallén, 1823) [Hackman 1980]
Medetera jugalis Collin, 1941 [Karppinen 1969b, Hackman 1980]
Medetera melancholica Lundbeck, 1912 [Frey 1915, Hackman 1980]
Medetera muralis Meigen, 1824 [Hackman 1980]
Medetera nitida (Macquart, 1834) [Frey 1915, Hackman 1980]
= *M. stackelbergi* Parent, 1927

Medetera obscura (Zetterstedt, 1838) [Hackman 1980]
Medetera pallipes (Zetterstedt, 1843) [Hackman 1980]
Medetera parenti Stackelberg, 1925 [Hackman 1980]
= *M. collini* Thuneberg, 1955 [Kangas 1949]
Medetera pinicola Kowarz, 1877 [Hackman 1980]
= *M. nuortevai* Thuneberg, 1955
Medetera plumbella Meigen, 1824 [Hackman 1980]
Medetera prjachinae Negrobov & Stackelberg, 1974 [Kahanpää 2013a]
Medetera protuberans Negrobov, 1967 [Karppinen 1969b, Hackman 1980]
Medetera pseudoapicalis Thuneberg, 1955 [Hackman 1980]
Medetera seguyi Parent, 1926 [Kahanpää 2013a]
Medetera senicula Kowarz, 1877 [Hackman 1980]
Medetera setiventris Thuneberg, 1955 [Hackman 1980]
Medetera signaticornis Loew, 1857 [Frey 1915, Hackman 1980]
Medetera striata Parent, 1927 [Kangas 1949, Hackman 1980]
Medetera tristis (Zetterstedt, 1838) [Hackman 1980]
Medetera vagans Becker, 1917 [Hackman 1980]
= *M. fennica* Thuneberg, 1955
Medetera veles Loew, 1861
= *M. bilineata* Frey, 1915 [Frey 1915, Hackman 1980]
Medetera zinovjevi Negrobov, 1967 [Kahanpää 2013a]
SYSTENUS Loew, 1857
Systenus bipartitus (Loew, 1850) [Frey 1915, Hackman 1980]
Systenus pallipes (von Roser, 1840) [Hackman 1980]
= *S. adpropinquus* (Loew, 1857) [Frey 1915]
Systenus scholtzi (Loew, 1850) [Kahanpää and Grichanov 2006]
THRYPTICUS Gerstäcker, 1864
Thrypticus atomus Frey, 1915 [Frey 1915, Hackman 1980]
Thrypticus bellus Loew, 1869 [Frey 1915, Hackman 1980]
Thrypticus cuneatus (Becker, 1917) [Hackman 1980]
Thrypticus divisus Strobl, 1880 [Kahanpää and Grichanov 2004]
= *T. fennicus* Becker, 1917 [Hackman 1980]
Thrypticus intercedens Negrobov, 1967 [Kahanpää and Grichanov 2004]
Thrypticus laetus Verrall, 1912 [Hackman 1980]
Thrypticus nigricauda Wood, 1913 [Kahanpää and Grichanov 2004]
Thrypticus pollinosus Verrall, 1912 [Ano 1956, Hackman 1980]
Thrypticus pruinosus Parent, 1932 [Hackman 1980]
Thrypticus smaragdinus Gerstäcker, 1864 [Hackman 1980]
Thrypticus tarsalis Parent, 1932 [Kahanpää 2006]
Thrypticus sp. A
HYDROPHORINAE Lioy, 1864
HYDROPHORUS Fallén, 1823
Hydrophorus albiceps Frey, 1915 [Frey 1915, Hackman 1980]
Hydrophorus alpinus Wahlberg, 1844 [Frey 1915, Hackman 1980]
Hydrophorus altivagus Aldrich, 1911
= *H. wahlgreni* Frey, 1915 [Frey 1915, Hackman 1980]
Hydrophorus bipunctatus (Lehmann, 1822) [Frey 1915, Hackman 1980]
Hydrophorus borealis Loew, 1857 [Hackman 1980]
Hydrophorus brunnicosus Loew, 1857 [Frey 1915, Hackman 1980]
Hydrophorus callosoma Frey, 1915 [Frey 1947, Kahanpää 2013a]

= *H. albosignatus* Ringdahl, 1919
Hydrophorus freyi Storå, 1954 [Storå 1954, Hackman 1980]
Hydrophorus geminus Frey, 1915 [Frey 1915, Hackman 1980]
Hydrophorus litoreus Fallén, 1823 [Hackman 1980]
Hydrophorus nebulosus Fallén, 1823 [Hackman 1980]
Hydrophorus norvegicus Ringdahl, 1928 [Hackman 1980]
Hydrophorus pectinatus Gerstäcker, 1864 [Hackman 1980]
= *H. forcipatus* Frey, 1915 [Frey 1915]
Hydrophorus pilipes Frey, 1915 [Frey 1915, Hackman 1980]
Hydrophorus praecox (Lehmann, 1822) [Hackman 1980]
Hydrophorus rufibarbis Gerstäcker, 1864 [Hackman 1980]
= *H. micans* Frey, 1915 [Frey 1915]
Hydrophorus signiferus Coquillett, 1899
= *H. magnicornis* Frey, 1915 [Frey 1915, Hackman 1980]
Hydrophorus viridis (Meigen, 1824) [Frey 1915, Hackman 1980]
LIANCALUS Loew, 1857
Liancalus virens (Scopoli, 1763) [Frey 1915, Hackman 1980]
PEODES Loew, 1857
Peodes petsamoensis Frey, 1930 [Frey 1930, Kahanpää 2006]
SCELLUS Loew, 1857
Scellus spinimanus (Zetterstedt, 1843) [Hackman 1980]
THINOPHILUS Wahlberg, 1844
sg. ***Thinophilus*** Wahlberg, 1844
Thinophilus flavipalpis (Zetterstedt, 1843) [Frey 1915, Hackman 1980]
Thinophilus ruficornis (Haliday, 1838) [Frey 1915, Hackman 1980]
NEURIGONINAE Aldrich, 1905
NEURIGONA Rondani, 1856
Neurigona abdominalis (Fallén, 1823) [Frey 1915, Hackman 1980]
Neurigona pallida (Fallén, 1823) [Hackman 1980]
Neurigona quadrifasciata (Fabricius, 1781) [Hackman 1980]
Neurigona suturalis (Fallén, 1823) [Frey 1915, Hackman 1980]
Neurigona sp. A
RHAPHIINAE Bigot, 1852
RHAPHIUM Meigen, 1803
= ***Xiphandrium*** Loew, 1857
Rhaphium albifrons (Zetterstedt, 1843) [Kahanpää and Grichanov 2006]
Rhaphium appendiculatum (Zetterstedt, 1849) [Hackman 1980]
= *R. macrocerum* auct. nec Meigen, 1824
Rhaphium basale Loew, 1850 [Hackman 1980]
Rhaphium caliginosum Meigen, 1824 [Hackman 1980]
= *R. zetterstedti* (Parent, 1925) [Haarto et al. 2002, Haarto 2002b]
Rhaphium commune (Meigen, 1824) [Hackman 1980]
= *R. spinicoxa* Loew, 1850 [Frey 1915]
Rhaphium confine Zetterstedt, 1843 [Frey 1915, Hackman 1980]
Rhaphium crassipes (Meigen, 1824) [Hackman 1980]
Rhaphium discolor Zetterstedt, 1838
= *R. consobrinum* Zetterstedt, 1843 [Frey 1915]
= *R. riparium* auct. nec Meigen, 1824 [Hackman 1980]
Rhaphium elegantulum (Meigen, 1824) [Hackman 1980]
Rhaphium fasciatum Meigen, 1824 [Frey 1915, Hackman 1980]

Rhaphium fascipes (Meigen, 1824) [Hackman 1980]
Rhaphium fissum Loew, 1850 [Kahanpää 2006]
Rhaphium glaciale (Ringdahl, 1920) [Hellén 1946]
Rhaphium holmgreni (Mik, 1878) [Hackman 1980]
= *R. luteipenne* (Frey, 1915) [Frey 1915, Hackman 1980]
Rhaphium lanceolatum Loew, 1850 [Hackman 1980]
= *R. caliginosum* auct. nec Meigen, 1824
Rhaphium laticorne (Fallén, 1823) [Hackman 1980]
= *R. nemorum* Meigen, 1830 [Frey 1915]
Rhaphium latimanum Kahanpää, 2007 [Kahanpää 2007b]
Rhaphium longicorne (Fallén, 1823) [Hackman 1980]
Rhaphium micans (Meigen, 1824) [Frey 1915, Hackman 1980]
Rhaphium monotrichum Loew, 1850 [Frey 1915, Hackman 1980]
= *R. auctum* misid.[Kahanpää and Grichanov 2006, Kahanpää 2006]
Rhaphium nasutum (Fallén, 1823) [Hackman 1980]
Rhaphium nigribarbatum (Becker, 1900) [Frey 1915, Hackman 1980]
Rhaphium patulum (Raddatz, 1873) [Hackman 1980]
= *R. antennatum* misid. [Frey 1915, Hackman 1980]
Rhaphium penicillatum Loew, 1850 [Frey 1915, Hackman 1980]
Rhaphium riparium (Meigen, 1824)
= *R. praerosum* Loew, 1850 [Hackman 1980]
Rhaphium rivale (Loew, 1869) [Frey 1915, Hackman 1980]
Rhaphium tridactylum (Frey, 1915) [Frey 1915, Hackman 1980]
Rhaphium umbripenne (Frey, 1915) [Frey 1915, Hackman 1980]
ACHALCINAE Grootaert & Meuffels, 1997
ACHALCUS Haliday, 1857
Achalcus cinereus (Haliday, 1851) [Frey 1915, Hackman 1980]
Achalcus flavicollis (Meigen, 1824) [Frey 1915, Hackman 1980]
Achalcus nigropunctatus Pollét & Brunhues, 1996 [Kahanpää 2013a]
Achalcus vaillanti Brunhues, 1987 [Kahanpää and Grichanov 2006]
PELOROPEODINAE Robinson, 1970
CHRYSOTIMUS Loew, 1857
Chrysotimus molliculus (Fallén, 1823) [Kahanpää and Grichanov 2006]
MICROMORPHUS Mik, 1878
Micromorphus claripennis (Strobl, 1899) [Kahanpää 2013a]
XANTHOCHLORINAE Aldrich, 1905
XANTHOCHLORUS Loew, 1857
Xanthochlorus ornatus (Haliday, 1832) [Frey 1915, Hackman 1980]
Xanthochlorus tenellus (Wiedemann, 1817) [Hackman 1980]

Excluded species

Chrysotus arcticus Frey, 1915 [Hackman 1980]
Chrysotus longipalpus Aldrich, 1896
= *C. pallidipalpus* van Duzee, 1933 [Frey 1927, Hackman 1980]
Chrysotus ringdahli Parent, 1929 [Hackman 1980]
Dolichopus angustipennis Kertesz, 1901
= *D. adustus* Frey, 1915 [Frey 1915]
Dolichopus grandicornis Wahlberg, 1850 [Hackman 1980, Kahanpää and

Grichanov 2004]
Dolichopus mediicornis Verrall, 1875
Dolichopus plumitarsis Fallén, 1823
Dolichopus propinquus Zetterstedt, 1852 [Hackman 1980, Kahanpää and Grichanov 2004]
Gymnopternus assimilis (Stæger, 1842)
Hydrophorus balticus (Meigen, 1824) [Frey 1927, Kahanpää and Grichanov 2004]
Hydrophorus ponojensis Frey, 1915 [Frey 1915]
Medetera annulitarsus von Roser, 1840
Medetera glauca Loew, 1869
Medetera feminina Negrobov, 1967 [Thuneberg 1975, Kahanpää and Grichanov 2004]
Medetera truncorum Meigen, 1824
Parathalassius krogerusi Frey, 1927 [Mee 1927]
Rhaphium antennatum (Carlier, 1835) [Kahanpää and Grichanov 2004]
Rhaphium obscuripes Zetterstedt, 1849 [Hackman 1980, Kahanpää and Grichanov 2004]
Rhaphium suave (Loew, 1859) [Kahanpää and Grichanov 2004]
Tachytrechus insignis (Stannius, 1831)

OPETIIDAE Rondani, 1856

Supporting references for *Ståhls G. (2014) Checklist of the families Opetiidae and Platypezidae (Diptera) of Finland. In: Kahanpää J, Salmela J (Eds) Checklist of the Diptera of Finland. ZooKeys @@: @-@. doi: 10.3897/zookeys.??.7639*

OPETIA Meigen, 1830
Opetia nigra Meigen, 1830 [Hackman 1980]

PLATYPEZIDAE Fallén, 1815

Supporting references for *Ståhls G. (2014) Checklist of the families Opetiidae and Platypezidae (Diptera) of Finland. In: Kahanpää J, Salmela J (Eds) Checklist of the Diptera of Finland. ZooKeys @@: @-@. doi: 10.3897/zookeys.??.7639*

CALLOMYIINAE Rondani, 1841
AGATHOMYIA Verrall, 1901
Agathomyia alneti Ståhls & Rättel, in prep.
Agathomyia antennata (Zetterstedt, 1819) [Hackman 1980]
Agathomyia boreella (Zetterstedt, 1838) [Frey et al. 1941]
Agathomyia cinerea (Zetterstedt, 1852) [Hackman 1980]
Agathomyia elegantula (Fallén, 1815) [Hackman 1980]
= *A. dahlbomi* (Zetterstedt, 1838) [Frey et al. 1941]
Agathomyia falleni (Zetterstedt, 1819) [Winqvist 2011]
Agathomyia lundbecki Chandler *in* Shatalkin, 1985 [Stå hls and Kahanpää 2006]
Agathomyia scutellaris (Zetterstedt, 1838)
Agathomyia sexmaculata (von Roser, 1840)
Agathomyia shatalkini Ståhls in prep.
Agathomyia unicolor Oldenberg, 1928 [Stå hls and Kahanpää 2006]
Agathomyia vernalis Shatalkin, 1981 [Stå hls and Kahanpää 2006]
Agathomyia viduella (Zetterstedt, 1838) [Hackman 1980]
Agathomyia wankowiczii (Schnabl, 1884) [Stå hls and Kahanpää 2006]
Agathomyia woodella Chandler in Shatalkin, 1985 [Hackman 1980]
= *A. elegantula* auct. nec (Fallén, 1815) [Frey et al. 1941]
Agathomyia zetterstedti (Wahlberg *in* Zetterstedt, 1844) [Stå hls and Kahanpää 2006]
CALLOMYIA Meigen, 1804
Callomyia amoena Meigen, 1824 [Hackman 1980]
Callomyia elegans Meigen, 1804 [Hackman 1980]
= *C. leptiformis* (Fallén, 1810)
Callomyia krivosheinae Shatalkin, 1982
Callomyia speciosa Meigen, 1824 [Hackman 1980]
= *C. humeralis* Loew, 1869 [Fre 1935]
MICROSANIINAE Enderlein, 1936
MICROSANIA Zetterstedt, 1837
Microsania capnophila Shatalkin, 1985 [Stå hls and Rättel 2013]
Microsania collarti Chandler, 2001 [Stå hls and Rättel 2013]
Microsania pallipes (Meigen, 1830) [Hackman 1980]
Microsania pectipennis (Meigen, 1830) [Hackman 1980]
Microsania straeleni Collart, 1954 [Hackman 1980]
Microsania vrydaghi Collart, 1954 [Stå hls and Rättel 2013]
PLATYPEZINAE Fallén, 1815
BOLOPUS Enderlein, 1932
Bolopus furcatus (Fallén, 1826) [Stå hls et al. 2012]
PARAPLATYPEZA Kessel & Maggioncalda, 1968
Paraplatypeza atra (Meigen, 1804) [Hackman 1980]
Paraplatypeza bicincta (Szilády, 1941) [Chandler 2001b]
PLATYPEZA Meigen, 1803
Platypeza aterrima Walker, 1836 [Hackman 1980]

Platypeza consobrina Zetterstedt, 1844 [Stå hls and Kahanpää 2006]
Platypeza fasciata Meigen, 1804 [Hackman 1980]
Platypeza hirticeps Verrall, 1901 [Stå hls and Kahanpää 2006]
PLATYPEZINA Wahlgren, 1910
Platypezina connexa (Bohemann, 1858) [Hackman 1980]
POLYPORIVORA Kessel & Maggioncalda, 1968
Polyporivora boletina (Fallén, 1815) [Hackman 1980]
Polyporivora ornata (Meigen, 1838) [Hackman 1980]
= *P. infumata* (Haliday, 1838) [Frey et al. 1941]
Polyporivora picta (Meigen, 1830) [Hackman 1980]
PROTOCLYTHIA Kessel, 1950
Protoclythia modesta (Zetterstedt, 1844) [Chandler 2001b]
SERI Kessel & Maggioncalda, 1966
Seri obscuripennis (Oldenberg, 1916) [Hackman 1980]

LONCHOPTERIDAE Macquart, 1835

Supporting references for *Kahanpää J (2014) Checklist of the families Lonchopteridae and Phoridae of Finland (Insecta, Diptera). In: Kahanpää J, Salmela J (Eds) Checklist of the Diptera of Finland. ZooKeys @@: @-@. doi: 10.3897/zookeys.??.7197*

LONCHOPTERA Meigen, 1803
= ***Dipsa*** Fallén, 1810
Lonchoptera bifurcata (Fallén, 1810)
= *L. furcata* (Fallén, 1823) [Hackman 1980]
Lonchoptera fallax de Meijere, 1906 [Hackman 1980]
Lonchoptera impicta Zetterstedt, 1848 [Andersson 1991]
Lonchoptera lutea Panzer, 1809 [Hackman 1980]
Lonchoptera meijerei Collin, 1938 [Kahanpää 2013d]
Lonchoptera nigrociliata Duda, 1927 [Andersson 1991]
Lonchoptera nitidifrons Strobl, 1898 [Andersson 1991]
Lonchoptera scutellata Stein, 1890 [Kahanpää 2013d]

PHORIDAE Curtis, 1833

Supporting references for *Kahanpää J (2014) Checklist of the families Lonchopteridae and Phoridae of Finland (Insecta, Diptera). In: Kahanpää J, Salmela J (Eds) Checklist of the Diptera of Finland. ZooKeys @@: @-@. doi: 10.3897/zookeys.??.7197*

AENIGMATIAS Meinert, 1890
Aenigmatias lubbockii (Verrall, 1877) [Haarto 1998]
ANEVRINA Lioy, 1864
Anevrina thoracica (Meigen, 1804) [Hackman 1980]
Anevrina unispinosa (Zetterstedt, 1860) [Hackman 1980]
= *A. fennica* (Becker, 1901)
Anevrina urbana (Meigen, 1830) [Hackman 1980]
BOROPHAGA Enderlein, 1924
Borophaga agilis (Meigen, 1830) [Hackman 1980]
Borophaga carinifrons (Zetterstedt, 1848) [Hackman 1980]
Borophaga femorata (Meigen, 1830) [Hackman 1980]
Borophaga incrassata (Meigen, 1830) [Kahanpää 2013d]
Borophaga subsultans (Linnaeus, 1767) [Frey et al. 1941]
= *B. o'kellyi* Schmitz, 1937 [Hackman 1980]
CHAETOPLEUROPHORA Schmitz, 1922
Chaetopleurophora erythronota (Strobl, 1892) [Hackman 1980]
Chaetopleurophora spinosissima (Strobl, 1892) [Kahanpää 2013d]
CONICERA Meigen, 1830
Conicera dauci (Meigen, 1830) [Hackman 1980]
= *C. atra* Meigen, 1830 [Frey et al. 1941]
Conicera floricola Schmitz, 1938 [Mee 1961a, Hackman 1980]
= *C. similis* auct. nec (Haliday, 1833) [Frey et al. 1941]
= *C. minuscula* Schmitz, 1953 [Hackman 1980, Disney 1980]
Conicera schnittmanni Schmitz, 1926
Conicera similis (Haliday, 1833)
= *C. pauxilla* Schmitz, 1920 [Hackman 1980]
? *Conicera tarsalis* Schmitz, 1920 [Hackman 1980]
Conicera tibialis Schmitz, 1925 [Hackman 1980]
= *C. fallens* Schmitz, 1948 [Hackman 1980, Disney 1981]
DIPLONEVRA Lioy, 1864
= ***Diploneura*** misspelling
Diplonevra florescens (Turton, 1801)
= *D. florea* (Fabricius, 1794) preocc. [Hackman 1980]
= *D. abdominalis* (Fallén, 1823) [Hackman 1980]
Diplonevra concinna (Meigen, 1830) [Kahanpää and Winqvist 2003]
Diplonevra freyi Schmitz, 1927 [Hackman 1980]
Diplonevra funebris (Meigen, 1830) [Hackman 1980]
= *D. rostralis* (Schmitz, 1918)
Diplonevra glabra Schmitz, 1927 [Hackman 1980]
= *D. parcepilosa* Schmitz, 1927
Diplonevra nitidula (Meigen, 1830) [Hackman 1980]
Diplonevra oldenbergi Schmitz, 1920 [Hackman 1980]
DOHRNIPHORA Dahl, 1898

Dohrniphora cornuta (Bigot, 1857) [Hackman 1977, 1980]
GYMNOPHORA Macquart, 1835
Gymnophora arcuata (Meigen, 1830) [Hackman 1980]
Gymnophora healeyae Disney, 1980
Gymnophora nigripennis Schmitz, 1926 [Haarto and Winqvist 2014]
Gymnophora quartomollis Schmitz, 1920 [Hackman 1980]
HYPOCERA Lioy, 1864
Hypocera mordellaria (Fallén, 1823) [Hackman 1980]
MEGASELIA Rondani, 1856
 = ***Aphiochaeta*** Brues, 1903
 = ***Plastophora*** Brues, 1905
Megaselia aequalis (Wood, 1909) [Hackman 1980]
Megaselia affinis (Wood, 1909) [Schmitz 1958, Hackman 1980]
 = *M. proxima* (Lundbeck, 1920)
Megaselia albicaudata (Wood, 1910) [Hackman 1980]
Megaselia albiclava (Schmitz, 1926) [Hackman 1980]
Megaselia altifrons (Wood, 1909) [Hackman 1980]
Megaselia analis (Lundbeck, 1920) [Hackman 1980]
? *Megaselia angelicae* (Wood, 1910) [Hackman 1980]
Megaselia angularis (Schmitz, 1924) [Hackman 1980]
Megaselia angusta (Wood, 1909)
 = *M. angustata* misspelling[Hackman 1980, Disney 1989]
 = *M. pulicaria* auct. nec (Fallén, 1923) [Hackman 1980, Disney 1989]
Megaselia annulipes (Schmitz, 1921)
Megaselia armata (Wood, 1909) [Hackman 1980]
Megaselia atrosericea Schmitz, 1927 [Hackman 1980]
Megaselia auricoma Schmitz, 1927 [Hackman 1980]
Megaselia baltica (Schmitz, 1924) [Hackman 1980]
Megaselia barbulata (Wood, 1909) [Hackman 1963, 1980]
 = *M. depilata* (Lundbeck, 1921)
? *Megaselia basispinata* (Lundbeck, 1920) [Hackman 1980]
Megaselia basiveluta Schmitz, 1935 [Hackman 1980]
Megaselia beckeri (Wood, 1909)
 = *M. laticrus* Schmitz, 1927 [Hackman 1980]
Megaselia berndseni (Schmitz, 1919)
 = *M. pygmaeoides* (Lundbeck, 1921) [Hackman 1980]
Megaselia bovista (Gimmerthal, 1848) [Hackman 1980]
Megaselia brevicostalis (Wood, 1910) [Hackman 1980]
Megaselia breviseta (Wood, 1912) [Disney 2012]
Megaselia breviterga (Lundbeck, 1920) [Hackman 1980]
 = *M. similata* (Lundbeck, 1921)
Megaselia campestris (Wood, 1908) [Hackman 1980]
Megaselia ciliata (Zetterstedt, 1848) [Hackman 1980]
Megaselia cinereifrons (Strobl, 1910) [Hackman 1980]
Megaselia clara (Schmitz, 1921) [Hackman 1980]
Megaselia coaetanea Schmitz, 1929 [Hackman 1980]
Megaselia coccyx Schmitz, 1965 [Schmitz and Beyer 1965a, Hackman 1980]
? *Megaselia collini* (Wood, 1909) [Hackman 1980]
Megaselia conformis (Wood, 1909) [Hackman 1980]
Megaselia costalis (von Roser, 1840) [Hackman 1980]

Megaselia curvivenia Schmitz, 1928 [Hackman 1980]
Megaselia dahli (Becker, 1901) [Hackman 1980, Haarto and Winqvist 2014]
Megaselia discreta (Wood, 1909) [Hackman 1980]
= *M. nudiventris* (Wood, 1909) [Hackman 1980, Disney 1985]
Megaselia diversa (Wood, 1909) [Hackman 1980]
Megaselia dubitalis (Wood, 1908) [Hackman 1980]
Megaselia eccoptomera Schmitz, 1927 [Hackman 1980]
Megaselia eisfelderae Schmitz, 1948 [Hackman 1980]
Megaselia elongata (Wood, 1914)
Megaselia erecta (Wood, 1910)
Megaselia exarcuata Schmitz, 1927 [Hackman 1980]
Megaselia excavata Schmitz, 1927 [Hackman 1980]
Megaselia excorticata Disney, 2009 [Disney 2009]
Megaselia fennicola Beyer, 1958 [Beyer 1959, Hackman 1980]
Megaselia flammula Schmitz, 1928 [Hackman 1980]
Megaselia flava (Fallén, 1823) [Hackman 1980]
Megaselia flavicoxa (Zetterstedt, 1848) [Hackman 1980]
Megaselia fumata (Malloch, 1909) [Hackman 1980]
Megaselia funeralis Schmitz, 1928 [Hackman 1980]
Megaselia fungivora (Wood, 1909) [Hackman 1963]
Megaselia fusca (Wood, 1909) [Hackman 1980]
Megaselia fuscipalpis (Lundbeck, 1920) [Hackman 1980]
Megaselia fuscoides Schmitz, 1934 [Hackman 1980]
Megaselia fuscovariana Schmitz, 1933
Megaselia giraudii (Egger, 1862) [Hackman 1980]
Megaselia glabrifrons (Wood, 1909) [Hackman 1980]
Megaselia groenlandica (Lundbeck, 1901) [Hackman 1980]
Megaselia halterata (Wood, 1910)
= *M. plurispinosa* (Lundbeck, 1920) [Hackman 1980]
Megaselia hectochaeta Schmitz, 1974 [Schmitz and Beyer 1974]
Megaselia hilaris Schmitz, 1927 [Hackman 1980]
Megaselia hirsuta (Wood, 1910) [Hackman 1980]
Megaselia hirticaudata (Wood, 1910) [Hackman 1980]
Megaselia hirticrus (Schmitz, 1918) [Hackman 1980]
Megaselia humeralis (Zetterstedt, 1838) [Hackman 1980]
= *M. cubitalis* (Becker, 1901)
Megaselia hyalipennis (Wood, 1912) [Hackman 1980]
Megaselia infraposita (Wood, 1909)
Megaselia ignobilis (Schmitz, 1919) [Hackman 1980]
Megaselia intonsa Schmitz, 1948 [Schmitz 1957, Hackman 1980]
Megaselia involuta (Wood, 1910) [Hackman 1980]
Megaselia latifemorata (Becker, 1901) [Hackman 1980]
Megaselia limburgensis (Schmitz, 1918) [Hackman 1980]
Megaselia longicostalis (Wood, 1912) [Hackman 1980]
Megaselia longifurca (Lundbeck, 1921) [Disney 2009]
= *M. spinolabella* Disney, 1989
Megaselia longipalpis (Wood, 1910) [Hackman 1980]
Megaselia longiseta (Wood, 1909) [Hackman 1980]
Megaselia lucifrons (Schmitz, 1918) [Hackman 1980]
= *M. subnitida* (Lundbeck, 1920) [Hackman 1980, Disney 1988]

Megaselia luminifrons (Schmitz, 1926) [Hackman 1980]
Megaselia lutea (Meigen, 1830) [Hackman 1980]
Megaselia major (Wood, 1912) [Hackman 1980]
Megaselia mallochi (Wood, 1909) [Hackman 1980]
Megaselia manicata (Wood, 1910) [Hackman 1980]
Megaselia maura (Wood, 1910) [Hackman 1980]
Megaselia meconicera (Speiser, 1925) [Hackman 1963, 1980]
Megaselia meigeni (Becker, 1901) [Haarto and Winqvist 2014]
? *Megaselia miki* Schmitz, 1929 [Hackman 1980]
Megaselia minor (Zetterstedt, 1848) [Hackman 1980]
= *M. angustifrons* (Wood, 1912) [Hackman 1980, Disney 1984]
Megaselia minuta (Aldrich, 1892)
= *M. luminosa* Schmitz, 1952 [Hackman 1980]
Megaselia mixta (Schmitz, 1918) [Hackman 1980]
Megaselia nasoni (Malloch, 1914) [Hackman 1980]
= *M. coaequalis* (Schmitz, 1919) [Hackman 1963]
Megaselia nigra (Meigen, 1830) [Hackman 1980]
= *M. albidihalteralis* (Felt, 1896)
Megaselia nigriceps (Loew, 1866) [Hackman 1980]
= *M. projecta* (Becker, 1901)
Megaselia nigripalpis (Lundbeck, 1920) [Hackman 1980]
Megaselia nudipleura (Beyer, 1958) [Beyer 1959, Hackman 1980]
Megaselia obscuripennis (Wood, 1909) [Hackman 1980]
Megaselia offuscata (Schmitz, 1921) [Hackman 1980]
Megaselia opacicornis Schmitz, 1949 [Gross et al. 2004]
Megaselia parnassia Disney, 1986 [Disney 2012]
Megaselia parva (Wood, 1909) [Hackman 1980]
Megaselia pectoralis (Wood, 1910) [Hackman 1980]
Megaselia pectunculata Schmitz, 1927 [Hackman 1980]
Megaselia picta (Lehmann, 1822) [Hackman 1980]
Megaselia pleuralis (Wood, 1909) [Hackman 1980]
Megaselia plurispinulosa (Zetterstedt, 1860) [Hackman 1980]
Megaselia posticata (Strobl, 1898)
Megaselia praeacuta (Schmitz, 1919)
Megaselia prodroma (Lundbeck, 1921) [Disney 2011]
Megaselia producta (Schmitz, 1921) [Hackman 1980]
= *M. sordescens* auct. nec
Megaselia propinqua (Wood, 1909) [Hackman 1980]
Megaselia pubecula Schmitz, 1927 [Hackman 1980]
Megaselia pulicaria (Fallén, 1923) [Hackman 1980]
= *M. sinuata* Schmitz, 1926 [Hackman 1980]
Megaselia pumila (Meigen, 1830) [Hackman 1980]
= *M. atripes* (Brues, 1915)
Megaselia pusilla (Meigen, 1830) [Hackman 1980]
Megaselia pygmaea (Zetterstedt, 1848) [Hackman 1980]
Megaselia quadriseta (Schmitz, 1918)
= *M. badia* Schmitz, 1938
? *Megaselia raetica* Schmitz, 1934 [Hackman 1980]
Megaselia robertsoni Disney, 2008 [Disney 2009]
Megaselia robusta Schmitz, 1928 [Hackman 1980]

Megaselia rubricornis (Schmitz, 1919) [Hackman 1980]
Megaselia rudis (Wood, 1909) [Hackman 1980]
Megaselia ruficornis (Meigen, 1830) [Hackman 1980]
Megaselia rufipes (Meigen, 1804) [Hackman 1980]
Megaselia scalaris (Loew, 1866)
Megaselia scutellaris (Wood, 1909) [Hackman 1980]
= *M. scutellariformis* (Schmitz, 1926) [Hackman 1980, Disney 1985]
Megaselia sepulchralis (Lundbeck, 1920) [Hackman 1980]
Megaselia setulipalpis Schmitz, 1938 [Disney 2009]
Megaselia sordida (Zetterstedt, 1838) [Hackman 1980]
= *M. eminens* Schmitz, 1953 [Schmitz and Beyer 1965a, Hackman 1980, Disney 1985]
= *M. semiscaura* Schmitz, 1927 [Hackman 1980, Disney 1985]
? *Megaselia specularis* Schmitz, 1935 [Hackman 1963]
Megaselia spinicincta (Wood, 1910) [Hackman 1980]
Megaselia spinigera (Wood, 1908) [Hackman 1980]
Megaselia styloprocta (Schmitz, 1921) [Hackman 1980]
Megaselia subcarpalis (Lundbeck, 1920) [Schmitz and Beyer 1965b, Hackman 1980]
Megaselia subpalpalis (Lundbeck, 1920) [Hackman 1980]
Megaselia subpleuralis (Wood, 1909) [Hackman 1980]
Megaselia subtumida (Wood, 1909) [Hackman 1980]
Megaselia sulphuripes (Meigen, 1830) [Hackman 1980]
Megaselia superciliata (Wood, 1910) [Hackman 1980]
Megaselia superfurcata Schmitz, 1928 [Hackman 1980]
Megaselia sylvatica (Wood, 1910)
= *M. impolluta* (Schmitz, 1920) [Hackman 1980]
Megaselia tarsalis (Wood, 1910) [Hackman 1980]
Megaselia tenuiventris Schmitz, 1927 [Hackman 1980]
Megaselia tignorum Disney, 2009 [Disney 2009]
Megaselia tumida (Wood, 1909) [Hackman 1980]
= *M. setifer* Lundbeck, 1920 [Hackman 1980]
Megaselia unicolor (Schmitz, 1919) [Hackman 1963, 1980]
Megaselia valvata Schmitz, 1935 [Schmitz and Beyer 1965b, Hackman 1980]
Megaselia variana Schmitz, 1926 [Hackman 1980]
Megaselia vestita (Wood, 1914) [Hackman 1980]
Megaselia winqvisti Disney, 2011 [Disney 2011]
Megaselia woodi (Lundbeck, 1922) [Hackman 1980]
Megaselia xanthozona (Strobl, 1892) [Hackman 1980]
= *M. euryprocta* Schmitz, 1957 [Schmitz 1958, Hackman 1980]
Megaselia zonata (Zetterstedt, 1838) [Hackman 1980]
MENOZZIOLA Schmitz, 1927
Menozziola obscuripes (Schmitz, 1927) [Hackman 1980]
METOPINA Macquart, 1835
Metopina galeata (Haliday, 1833) [Hackman 1980]
= *M. inaequalis* Schmitz, 1927 [Frey et al. 1941]
Metopina oligoneura (Mik, 1867)
Metopina pileata Schmitz, 1936
MICROSELIA Schmitz, 1934
Microselia forsiusi (Schmitz, 1927) [Hackman 1980]
PHALACROTOPHORA Enderlein, 1912
Phalacrotophora berolinensis Schmitz, 1920 [Hackman 1980]

Phalacrotophora beuki Disney in Disney & Beuk, 1997
Phalacrotophora fasciata (Fallén, 1823) [Hackman 1980]
PHORA Latreille, 1796
= ***Trineura*** Meigen, 1803
Phora artifrons Schmitz, 1920 [Hackman 1980]
Phora atra (Meigen, 1804)
= *P. aterrima* (Fabricius, 1794) preocc.[Hackman 1980]
Phora bullata Schmitz, 1927 [Hackman 1980]
Phora convallium Schmitz, 1928 [Kahanpää 2013d]
Phora convergens Schmitz, 1920 [Kahanpää 2013d]
Phora dubia (Zetterstedt, 1848) [Hackman 1980]
= *P. schineri* (Becker, 1901) [Frey et al. 1941]
Phora edentata Schmitz, 1920 [Hackman 1980]
Phora hamata Schmitz, 1927 [Kahanpää 2013d]
Phora holosericea Schmitz, 1920 [Hackman 1980]
Phora hyperborea Schmitz, 1927 [Hackman 1980]
Phora obscura (Zetterstedt, 1848) [Hackman 1980]
Phora occidentata Malloch, 1912 [Hackman 1980]
= *P. zetterstedti* Schmitz, 1927 [Frey et al. 1941]
Phora penicillata Schmitz, 1920 [Hackman 1980]
Phora praepandens Schmitz, 1927 [Hackman 1980]
Phora pubipes Schmitz, 1920 [Hackman 1980]
Phora stictica Meigen, 1830
Phora tincta Schmitz, 1920 [Hackman 1980]
PLECTANOCNEMA Schmitz, 1926
Plectanocnema nudipes (Becker, 1901) [Kahanpää 2013d]
PSEUDACTEON Coquillett, 1907
Pseudacteon fennicus Schmitz, 1927 [Hackman 1980]
Pseudacteon formicarum (Verrall, 1877) [Haarto and Winqvist 2014]
SPINIPHORA Malloch, 1909
Spiniphora bergenstammi Mik, 1864
Spiniphora dorsalis (Becker, 1901) [Flinck and Kahanpää 2013]
Spiniphora excisa (Becker, 1901) [Haarto and Winqvist 2014]
Spiniphora maculata (Meigen, 1830) [Kahanpää and Winqvist 2003]
= *S. helicivora* (Dufour, 1841)
TRIPHLEBA Rondani, 1856
= ***Citrago*** Schmitz, 1924
Triphleba admirabilis Schmitz, 1927 [Hackman 1980]
? *Triphleba aequalis* (Schmitz, 1919) [Hackman 1980]
? *Triphleba autumnalis* (Becker, 1901) [Disney 1991]
Triphleba bicornuta (Strobl, 1910) [Hackman 1980]
= *T. uncinata* (Schmitz, 1918) [Frey et al. 1941]
Triphleba citreiformis (Becker, 1901) [Hackman 1980]
Triphleba distinguenda (Strobl, 1892) [Schmitz 1943, Hackman 1980]
= *T. unicalcarata* (Becker, 1901) [Frey et al. 1941]
Triphleba excisa (Lundbeck, 1921) [Hackman 1980]
Triphleba gracilis (Wood, 1907) [Hackman 1980]
Triphleba hyalinata (Meigen, 1830) [Haarto and Winqvist 2014]
? *Triphleba inaequalis* Schmitz, 1943 [Disney 1991]
Triphleba intermedia (Malloch, 1908) [Hackman 1980]

Triphleba lugubris (Meigen, 1830) [Hackman 1980]
Triphleba luteifemorata (Wood, 1906) [Haarto and Winqvist 2014]
Triphleba nudipalpis (Becker, 1901) [Hackman 1980]
Triphleba opaca (Meigen, 1830) [Hackman 1980]
Triphleba pachyneurella (Schmitz, 1919) [Hackman 1980]
Triphleba palposa (Zetterstedt, 1848) [Hackman 1980]
Triphleba renidens Schmitz, 1927 [Hackman 1980]
Triphleba subcompleta Schmitz, 1927 [Hackman 1980]
Triphleba trinervis (Becker, 1901) [Hackman 1980]
VERUANUS Schmitz, 1927
Veruanus oldenbergi Schmitz, 1919
= *V. memorabilis* Schmitz, 1927 [Hackman 1980]

Excluded species

Megaselia cirriventris Schmitz, 1929 [Hackman 1980] mistake
Megaselia crassipes (Wood, 1909) [Hackman 1980]
Megaselia digitalis Schmitz, 1957 [Disney 1991]
Megaselia indifferens (Lundbeck, 1920) [Disney 1991]
Megaselia lapponica Schmitz, 1928 [Schmitz and Beyer 1965a]
Megaselia luteipes (Schmitz, 1918) [Hackman 1980]
Megaselia palmeni (Becker, 1901) [Disney 1991]
Megaselia septentrionalis (Schmitz, 1919) [Hackman 1980]
Megaselia simulans (Wood, 1912) [Schmitz 1957]
Triphleba gilvipes Schmitz, 1943 [Disney 1991]

PIPUNCULIDAE Walker, 1834

Supporting references for *Kehlmaier C (2014) Checklist of the family Pipunculidae (Diptera) of Finland. In: Kahanpää J, Salmela J (Eds) Checklist of the Diptera of Finland. ZooKeys @@: @-@. doi:10.3897/zookeys.??.7278*

CHALARINAE Aczél, 1939
CHALARUS Walker, 1834
Chalarus basalis Loew, 1873 [Jervis 1992]
Chalarus brevicaudis Jervis, 1992 [Jervis 1992]
Chalarus decorus Jervis, 1992 [Jervis 1992]
Chalarus elegantulus Jervis, 1992 [Jervis 1992]
= *C. absconditus* Kehlmaier in Kehlmaier & Assmann, 2008
Chalarus fimbriatus Coe, 1966 [Jervis 1992]
Chalarus gynocephalus Jervis, 1992 [Jervis 1992]
Chalarus holosericeus (Meigen, 1824)
= *C. perplexus* Jervis, 1992 [Jervis 1992]
Chalarus immanis Kehlmaier, 2008 [Kehlmaier and Assmann 2008]
Chalarus indistinctus Jervis, 1992 [Jervis 1992]
Chalarus juliae Jervis, 1992 [Jervis 1992]
Chalarus latifrons Hardy, 1943 [Jervis 1992]
Chalarus pughi Coe, 1966 [Jervis 1992]
Chalarus spurius (Fallén, 1816) [Hackman 1980]
= *C. obscurus* (Zetterstedt, 1838)
= *C. argenteus* misid. [Kehlmaier and Stå hls 2008]
JASSIDOPHAGA Aczél, 1939
Jassidophaga beatricis (Coe, 1966) [Kehlmaier and Stå hls 2008]
Jassidophaga fasciata (von Roser, 1840) [Kehlmaier 2006]
= *J. setosa* (Verrall, 1901) [Albrecht 1979c, Hackman 1980]
Jassidophaga pilosa (Zetterstedt, 1838) [Hackman 1980]
Jassidophaga villosa (von Roser, 1840) [Hackman 1980]
Jassidophaga spec.A [Kehlmaier and Stå hls 2008]
VERRALLIA Mik, 1899
Verrallia aucta (Fallén, 1817) [Hackman 1980]
NEPHROCERINAE Aczél, 1939
NEPHROCERUS Zetterstedt, 1838
Nephrocerus flavicornis Zetterstedt, 1844 [Albrecht 1979c, Hackman 1980]
Nephrocerus lapponicus Zetterstedt, 1838 [Hackman 1980]
Nephrocerus scutellatus (Macquart, 1834) [Hackman 1980]
PIPUNCULINAE Walker, 1834
tribe Cephalopsini Macquart, 1834
CEPHALOPS Fallén, 1810
sg. ***Cephalops*** Fallén, 1810
Cephalops aeneus Fallén, 1810 [Hackman 1980]
Cephalops vittipes (Zetterstedt, 1844) [Hackman 1980]
= *C. annulipes* (Zetterstedt, 1838) in part
sg. ***Parabeckerias*** De Meyer, 1994
Cephalops obtusinervis (Zetterstedt, 1844) [Hackman 1980]
sg. ***Semicephalops*** De Meyer, 1994
Cephalops carinatus (Verrall, 1901) [Albrecht 1979c, Hackman 1980]

Cephalops straminipes (Becker, 1900) [Kehlmaier and Stå hls 2008]
= *C. chlorionae* (Frey, 1945) [Hackman 1980]
Cephalops subultimus Collin, 1956 [De Meyer and Backeljau 1990]
Cephalops varipes (Meigen, 1824) [Kehlmaier 2008]
= *C. semifumosus* (Kowarz, 1887) [De Meyer and Backeljau 1990]
CEPHALOSPHAERA Enderlein, 1936
Cephalosphaera furcata (Egger, 1860) [Hackman 1980]
Cephalosphaera germanica Aczél, 1940 [De Meyer and Backeljau 1990, Kehlmaier and Stå hls 2008]
tribe Eudorylini Rafael & De Meyer, 1992
CLISTOABDOMINALIS Skevington, 2001
Clistoabdominalis doczkali Kehlmaier, 2005 [Kehlmaier and Stå hls 2008]
EUDORYLAS Aczél, 1940
Eudorylas angustimembranus Kozánek & Kwon, 1991 [Kehlmaier and Stå hls 2008]
= *E. kozaneki* De Meyer, 1993
Eudorylas arcanus Coe, 1966 [Kehlmaier and Stå hls 2008]
Eudorylas barkalovi Kuznetzov, 1990 [Kehlmaier and Stå hls 2008]
Eudorylas carpathicus Kozánek, 1993 [Kehlmaier and Stå hls 2008]
Eudorylas coloratus (Becker, 1897) [Kehlmaier 2005]
Eudorylas elephas (Becker, 1897) [Hackman 1980]
Eudorylas fascipes (Zetterstedt, 1844) [Hackman 1980, Kehlmaier and Stå hls 2008]
Eudorylas furvulus Collin, 1956 [Albrecht 1979c, Hackman 1980]
Eudorylas fuscipes (Zetterstedt, 1844) [Hackman 1980]
= *E. roseri* misid.[Hackman 1980]
= *E. trochanteratus* misid.[Hackman 1980]
Eudorylas fusculus (Zetterstedt, 1844) [Hackman 1980]
Eudorylas goennersdorfensis Dempewolf & Dunk, 1996 [Kehlmaier 2005]
Eudorylas inferus Collin, 1956 [Kehlmaier and Stå hls 2008]
Eudorylas jenkinsoni Coe, 1966 [Kehlmaier and Stå hls 2008]
Eudorylas johnenae Dempewolf, 1996 [Kehlmaier and Stå hls 2008]
Eudorylas kowarzi (Becker, 1897) [Kehlmaier and Stå hls 2008]
Eudorylas montium (Becker, 1897) [Kehlmaier and Stå hls 2008]
Eudorylas obscurus Coe, 1966 [Kehlmaier and Stå hls 2008]
Eudorylas restrictus Coe, 1966 [Kehlmaier and Stå hls 2008]
= *E. pannonicus* misid.[Hackman 1980]
Eudorylas slovacus Kozánek, 1993 [Kehlmaier and Stå hls 2008]
Eudorylas stackelbergi Kuznetzov, 1990 [Kehlmaier and Stå hls 2008]
Eudorylas subfascipes Collin, 1956 [Kehlmaier and Stå hls 2008]
Eudorylas subterminalis Collin, 1956 [Kehlmaier and Stå hls 2008]
Eudorylas terminalis (Thomson, 1870) [Hackman 1980]
Eudorylas unicolor (Zetterstedt, 1844) [Hackman 1980]
Eudorylas vonderdunki Dempewolf, 1998 [Kehlmaier and Stå hls 2008]
Eudorylas zermattensis (Becker, 1897) [Kehlmaier and Stå hls 2008]
Eudorylas zonatus (Zetterstedt, 1849) [Hackman 1980]
Eudorylas zonellus Collin, 1956 [Kehlmaier and Stå hls 2008]
tribe Microcephalopsini Rafael & De Meyer, 1991
MICROCEPHALOPS De Meyer, 1989
Microcephalops opacus (Fallén, 1816) [Albrecht 1979c, Hackman 1980]
= *M. vestitus* (Becker, 1900)
tribe Pipunculini Walker, 1834

PIPUNCULUS Latreille, 1802
Pipunculus calceatus von Roser, 1840 [Kehlmaier and Stå hls 2008, Kehlmaier 2008]
Pipunculus campestris Latreille, 1802 [Hackman 1980]
= *P. ater* Meigen, 1824 [Hackman 1980]
= *P. spinipes* Meigen, 1830
= *P. thomsoni* Becker, 1897
Pipunculus dimi Kuznetzov, 1991 [Kehlmaier and Stå hls 2008, Kehlmaier 2008]
Pipunculus elegans Egger, 1860 [Kehlmaier and Stå hls 2008, Kehlmaier 2008]
= *P. spinipes* auct. nec Meigen, 1830 [Hackman 1980]
Pipunculus fonsecai Coe, 1966 [Kehlmaier and Stå hls 2008, Kehlmaier 2008]
Pipunculus lenis Kuznetsov, 1991 [Kehlmaier and Stå hls 2008, Kehlmaier 2008]
= *P. thomsoni* auct. nec Becker, 1897 [Hackman 1980]
Pipunculus lichtwardti Kozanek, 1981 [Kehlmaier and Stå hls 2008, Kehlmaier 2008]
Pipunculus oldenbergi Collin, 1956 [Kehlmaier and Stå hls 2008, Kehlmaier 2008]
Pipunculus omissinervis Becker, 1889 [Kehlmaier and Stå hls 2008, Kehlmaier 2008]
Pipunculus tenuirostris Kozanek, 1981 [Kehlmaier and Stå hls 2008, Kehlmaier 2008]
= *P. balticus* Kuznetzov, 1991
Pipunculus violovitshi Kuznetzov, 1991 [Kehlmaier 2008]
= *P. varipes* auct. nec Meigen, 1824 [Hackman 1980]
Pipunculus zugmayeriae Kowarz, 1887 [Kahanpää and Winqvist 2003]
tribe Tomosvaryellini Hardy, 1943
DORYLOMORPHA Aczél, 1939
sg. ***Dorylomorpha*** Aczél, 1939
Dorylomorpha aczeli (Hardy, 1947) [Albrecht 1990, Kehlmaier and Stå hls 2008]
Dorylomorpha confusa (Verrall, 1901) [Albrecht 1979c, Hackman 1980]
Dorylomorpha extricata (Collin, 1937) [Kehlmaier and Stå hls 2008]
Dorylomorpha imparata (Collin, 1937) [Albrecht 1979c, Hackman 1980]
Dorylomorpha rufipes (Meigen, 1824) [Hackman 1980]
= *D. xanthoceroides* (Aczél, 1939) [Albrecht 1979c, Hackman 1980]
Dorylomorpha spinosa Albrecht, 1979 [Albrecht 1979a, Hackman 1980]
sg. ***Dorylomyia*** Albrecht, 1990
Dorylomorpha beckeri (Aczél, 1939) [Albrecht 1979c, Hackman 1980]
Dorylomorpha xanthocera (Kowarz, 1887) [Hackman 1980, Albrecht 1981]
sg. ***Dorylomyza*** Albrecht, 1990
Dorylomorpha albitarsis (Zetterstedt, 1844) [Albrecht 1979c, Hackman 1980]
Dorylomorpha anderssoni Albrecht, 1979 [Albrecht 1983]
Dorylomorpha canadensis Hardy, 1943 [Albrecht 1979c, Hackman 1980]
Dorylomorpha clavata Albrecht, 1979 [Albrecht 1979a, Hackman 1980]
Dorylomorpha clavifemora Coe, 1966 [Albrecht 1981]
Dorylomorpha fennica Albrecht, 1979 [Albrecht 1979b, Hackman 1980]
Dorylomorpha hackmani Albrecht, 1979 [Albrecht 1979a, Hackman 1980]
Dorylomorpha haemorrhoidalis (Zetterstedt, 1838) [Hackman 1980]
Dorylomorpha infirmata (Collin, 1937) [Albrecht 1979c, Hackman 1980]
Dorylomorpha lautereri Albrecht, 1990 [Albrecht 1990]
Dorylomorpha occidens (Hardy, 1939) [Albrecht 1979c, Hackman 1980]
Dorylomorpha onegensis Albrecht, 1990 [Albrecht 1990]
Dorylomorpha platystylis Albrecht, 1979 [Albrecht 1979a, Hackman 1980]
Dorylomorpha praetermissa Albrecht, 1979 [Albrecht 1979a, Hackman 1980]
Dorylomorpha xanthopus (Thomson, 1870) [Hackman 1980]
sg. ***Pipunculina*** Albrecht, 1990

Dorylomorpha borealis (Wahlgren, 1910) [Hackman 1980]
Dorylomorpha maculata (Walker, 1834) [Hackman 1980]
TOMOSVARYELLA Aczél, 1939
= ***Alloneura*** Rondani, 1856 nom. nudum
Tomosvaryella cilitarsis (Strobl, 1910) [Hackman 1980]
= *T. forsiusi* (Frey, 1932) [Frey 1932b]
Tomosvaryella coquilletti (Kertész, 1907) [Kehlmaier and Ståhls 2008]
Tomosvaryella geniculata (Meigen, 1824) [Hackman 1980]
= *T. nigritula* (Zetterstedt, 1844)
Tomosvaryella kalevala Kehlmaier, 2008 [Kehlmaier 2008]
Tomosvaryella kuthyi Aczél, 1944 [Kehlmaier and Ståhls 2008]
Tomosvaryella minuscula (Collin, 1956) [Kehlmaier and Ståhls 2008]
= *T. magyarica* Földvári & De Meyer, 1999
Tomosvaryella palliditarsis (Collin, 1931) [Kehlmaier and Ståhls 2008]
Tomosvaryella rossica Kuznetzov, 1993 [Kehlmaier 2008, Kehlmaier and Ståhls 2008]
Tomosvaryella sylvatica (Meigen, 1824) [Hackman 1980]

Excluded species

Claraeola halterata (Meigen, 1838) [Hackman 1980, Kehlmaier and Ståhls 2008]
Dorylomorpha incognita (Verrall, 1901) [Albrecht 1979c]
Clistoabdominalis trochanteratus (Becker, 1900 [Hackman 1980]
Dasydorylas roseri (Becker, 1897) [Hackman 1980]
Eudorylas pannonicus (Becker, 1897) [Hackman 1980]

SYRPHIDAE Latreille, 1802

Supporting references for *Haarto A, Kerppola S (2014) Checklist of the family Syrphidae (Diptera) of Finland. In: Kahanpää J, Salmela J (Eds) Checklist of the Diptera of Finland. ZooKeys @@: @-@. doi: 10.3897/zookeys.??.7251*

SYRPHINAE Latreille, 1802
tribe Bacchini Bigot, 1883
BACCHA Fabricius, 1805
Baccha elongata (Fabricius, 1775) [Hackman 1980]
= *B. obscuripennis* Meigen, 1822 [Hackman 1980]
tribe Melanostomini Williston, 1885
MELANOSTOMA Schiner, 1860
Melanostoma dubium (Zetterstedt, 1837) [Hackman 1980]
= *M. freyi* (Hellen, 1950) [Ano 1956]
Melanostoma mellinum (Linnaeus, 1758) [Hackman 1980]
Melanostoma scalare (Fabricius, 1794) [Hackman 1980]
PLATYCHEIRUS Le Peletier & Serville, 1828
sg. ***Platycheirus*** Le Peletier & Serville, 1828
Platycheirus aeratus Coquillet, 1900
= *P. angustitarsis* Kanervo, 1934 [Hackman 1980]
Platycheirus albimanus (Fabricius, 1781) [Hackman 1980]
Platycheirus ambiguus (Fallén, 1817) [Hackman 1980]
Platycheirus amplus Curran, 1927 [Haarto 1997]
Platycheirus angustatus (Zetterstedt, 1843) [Hackman 1980]
Platycheirus aurolateralis (Stubbs, 2002) [Haarto and Kerppola 2007b,a]
Platycheirus brunnifrons Nielsen, 2004 [Nielsen 2004]
Platycheirus carinatus (Curran, 1927)
? = *P. hirtipes* Kanervo, 1938 [Kanervo 1938, Hackman 1980]
Platycheirus clypeatus (Meigen, 1822) [Hackman 1980]
Platycheirus discimanus Loew, 1871 [Hackman 1980]
Platycheirus europaeus Goeldlin, Maibach & Speight, 1990 [Haarto and Kerppola 2004]
Platycheirus fulviventris (Macquart, 1829) [Hackman 1980]
Platycheirus goeldlini Nielsen, 2004 [Nielsen 2004]
Platycheirus groenlandicus Curran, 1927
= *P. boreomontanus* Nielsen, 1981 [Hackman 1980]
= *P. monticolus* Nielsen, 1972 preocc.[Nielsen 1972]
Platycheirus holarcticus Vockeroth, 1990 [van Steenis and Goeldlin de Tiefenau 1998]
Platycheirus hyperboreus (Staeger, 1844) [Hackman 1980]
Platycheirus immarginatus (Zetterstedt, 1849) [Hackman 1980]
Platycheirus jaerensis Nielsen, 1971 [Hippa 1972, Hackman 1980]
Platycheirus kittilaensis Dušek & Láska, 1982 [Dušek and Láska 1982]
= *P. complicatus* misid.[Haarto 1995, Haarto and Kerppola 2004]
Platycheirus laskai Nielsen, 1999 [Haarto and Kerppola 2004]
Platycheirus latimanus (Wahlberg, 1844) [Hackman 1980]
Platycheirus lundbecki (Collin, 1931) [Haarto and Kerppola 2004]
Platycheirus magadanensis Mutin, 1999 [Haarto and Kerppola 2007b,a]
Platycheirus manicatus (Meigen, 1822) [Hackman 1980]
Platycheirus modestus Ide, 1926
Platycheirus nielseni Vockeroth, 1990 [Haarto 1997]

Platycheirus nigrofemoratus Kanervo, 1934 [Haarto 2000]
Platycheirus occultus Goeldlin, Maibach & Speight, 1990 [Haarto 1997]
Platycheirus parmatus Rondani, 1857 [Haarto and Kerppola 2004]
Platycheirus peltatus (Meigen, 1822) [Hackman 1980]
Platycheirus perpallidus Verral, 1901 [Haarto and Kerppola 2004]
Platycheirus podagratus (Zetterstedt, 1838) [Hackman 1980]
Platycheirus ramsarensis Goeldlin, Maibach & Speight, 1990 [Haarto 2002a,b]
Platycheirus scambus (Staeger, 1843) [Hackman 1980]
Platycheirus scutatus (Meigen, 1822) [Hackman 1980]
Platycheirus splendidus Rotheray, 1998 [Haarto and Kerppola 2004]
Platycheirus sticticus (Meigen, 1822) [Hackman 1980]
Platycheirus subordinatus Becker, 1915 [Haarto 2002a,b]
Platycheirus tarsalis (Schummel, 1836) [Haarto and Kerppola 2004]
Platycheirus transfugus (Zetterstedt, 1838) [Hackman 1980]
Platycheirus urakawensis (Matsumura, 1919) [Haarto and Kerppola 2007a]
Platycheirus varipes Curran, 1923 [Haarto and Kerppola 2004]
sg. ***Pyrophaena*** Schiner, 1860
Platycheirus granditarsus (Forster, 1771) [Hackman 1980]
Platycheirus rosarum (Fabricius, 1787) [Hackman 1980]
XANTHANDRUS Verrall, 1901
Xanthandrus comtus (Harris, 1780) [Hackman 1980]
tribe Paragini Glumac, 1961
PARAGUS Latreille, 1804
Paragus constrictus Šimić, 1986 [Kerppola 2013]
Paragus finitimus Goeldlin, 1971 [Hippa 1972, Hackman 1980]
Paragus haemorrhous Meigen, 1822
= *P. sigillatus* Curtis, 1836 [Hippa 1972, Hackman 1980]
Paragus pecchiolii Rondani, 1857 [Haarto and Kerppola 2007a]
= *P. majoranae* auct. nec Rondani, 1857 [Stå hls 1995]
= *P. albifrons* misid.[Hackman 1980, Stå hls 1995]
Paragus tibialis (Fallén, 1817) [Hackman 1980]
tribe Chrysotoxini Newman, 1834
CHRYSOTOXUM Meigen, 1803
Chrysotoxum arcuatum (Linnaeus, 1758) [Hackman 1980]
= *C. fasciatum* (Müller, 1764)
Chrysotoxum bicinctum (Linnaeus, 1758) [Hackman 1980]
Chrysotoxum cautum (Harris, 1776) [Hackman 1980]
Chrysotoxum fasciolatum (De Geer, 1776) [Hackman 1980]
Chrysotoxum festivum (Linnaeus, 1758) [Hackman 1980]
= *C. arcuatum* auct. nec (Linnaeus, 1758)
Chrysotoxum octomaculatum Curtis, 1937 [Haarto and Kerppola 2004]
= *C. elegans* misid.[Haarto and Kerppola 2004]
Chrysotoxum vernale Loew, 1841 [Hackman 1980]
tribe Syrphini Latreille, 1802
DASYSYRPHUS Enderlein, 1838
Dasysyrphus albostriatus (Fallén, 1817) [Hackman 1980]
Dasysyrphus friuliensis (van der Goot, 1960) [Haarto and Kerppola 2004]
= *D. claviger* (Frey, 1930) [Frey 1930]
= *D. carpathicus* Stys & Moucha, 1962
Dasysyrphus hilaris (Zetterstedt, 1843) [Bartsch 2000]

Dasysyrphus nigricornis (Verrall, 1873) [Hackman 1980]
= *D. obscuratus* (Ringdahl, 1928) [Hackman 1980]
Dasysyrphus pauxillus (Williston, 1887)
Dasysyrphus pinastri (De Geer, 1776)
= *D. lunulatus* (Meigen, 1822) [Hackman 1980]
Dasysyrphus postclaviger Stys & Moucha, 1962 [Hackman 1980]
= *D. claviger* (Frey, 1930) preocc.
Dasysyrphus tricinctus (Fallén, 1817) [Hackman 1980]
Dasysyrphus venustus (Meigen, 1822)
= *D. arcuatus* (Fallén, 1817) [Hackman 1980]
DIDEA Macquart, 1834
Didea alneti (Fallén, 1817) [Hackman 1980]
Didea fasciata Macquart, 1834 [Hackman 1980]
Didea intermedia Loew, 1854 [Hackman 1980]
DOROS Meigen, 1803
Doros profuges (Harris, 1780)
= *D. conopseus* auct. nec (Linnaeus, 1758) [Hackman 1980]
EPISTROPHE Walker, 1852
Epistrophe annulitarsis Stackelberg, 1918 [Piirainen 2001]
Epistrophe cryptica Doczkal & Schmid, 1994 [Haarto 1997]
Epistrophe diaphana (Zetterstedt, 1843) [Haarto and Kerppola 2004]
Epistrophe eligans (Harris, 1780) [Bartsch 2000]
= *E. bifasciata* (Fabricius, 1794) preocc.
Epistrophe flava Doczkal & Schmid, 1994 [Stå hls 1995]
= *E. melanostomoides* misid.[Hackman 1980]
Epistrophe grossulariae (Meigen, 1822) [Hackman 1980]
Epistrophe melanostoma (Zetterstedt, 1843) [Hackman 1980]
= *E. melanostomoides* Strobl, 1910 [Stå hls 1995]
Epistrophe nitidicollis (Meigen, 1822) [Hackman 1980]
Epistrophe obscuripes (Strobl, 1910)
= *E. similis* Doczkal & Schmid, 1994 [Stå hls 1995]
Epistrophe ochrostoma (Zetterstedt, 1849) [Hackman 1980]
Epistrophe olgae Mutin, 1990 [Haarto and Kerppola 2007b,a]
EPISTROPHELLA Dušek & Láska, 1967
Epistrophella euchroma (Kowarz, 1885) [Hackman 1980, Stå hls 1995, Haarto 1997]
EPISYRPHUS Matsumura & Adachi, 1917
Episyrphus balteatus (De Geer, 1776) [Hackman 1980]
ERIOZONA Schiner, 1860
Eriozona syrphoides (Fallén, 1817) [Hackman 1980]
EUPEODES Osten Sacken, 1877
= ***Metasyrphus*** Matsumura, 1917
Eupeodes abiskoensis (Dušek & Láska, 1973) [Haarto 1995]
Eupeodes biciki (Nielsen, 2003) [Haarto and Kerppola 2007b,a]
Eupeodes bucculatus (Rondani, 1857)
= *E. latilunulatus* (Collin, 1931) [Haarto 1997]
Eupeodes corollae (Fabricius, 1794) [Hackman 1980]
Eupeodes curtus (Hine, 1922) [Haarto and Kerppola 2004]
Eupeodes duseki Mazánek, Láska & Bicik, 1999 [Haarto and Kerppola 2007b,a]
Eupeodes goeldlini Mazánek, Láska & Bicik, 1999 [Haarto and Kerppola 2007b,a]
Eupeodes latifasciatus (Macquart, 1829) [Hackman 1980]

Eupeodes lundbecki (Soot-Ryen, 1946) [Hackman 1980]
Eupeodes luniger (Meigen, 1822) [Hackman 1980]
Eupeodes nielseni (Dušek & Láska, 1976) [Dušek and Láska 1976, Hackman 1980]
Eupeodes nitens (Zetterstedt, 1843) [Hackman 1980]
Eupeodes punctifer (Frey, 1934) [Hackman 1980]
= *E. borealis* misid.[Haarto 1997]
Eupeodes tirolensis (Dušek & Láska, 1973) [Haarto and Kerppola 2007b,a]
FAGISYRPHUS Dušek & Láska, 1967
Fagisyrphus cinctus (Fallén, 1817) [Hippa 1972, Hackman 1980]
sg. ***Lapposyrphus*** Dušek & Láska, 1967
Lapposyrphus lapponicus (Zetterstedt, 1838) [Hackman 1980]
LEUCOZONA Schiner, 1860
sg. ***Leucozona*** Schiner, 1860
Leucozona inopinata Doczkal, 2000 [Haarto and Kerppola 2004]
Leucozona lucorum (Linnaeus, 1758) [Hackman 1980]
sg. ***Ischyrosyrphus*** Bigot, 1882
Leucozona glaucia (Linnaeus, 1758) [Hackman 1980]
Leucozona laternaria (Müller, 1776) [Hackman 1980]
MEGASYRPHUS Dušek & Láska, 1967
Megasyrphus erraticus (Linnaeus, 1758)
= *M. annulipes* (Zetterstedt, 1838) [Hackman 1980]
MELANGYNA Verrall, 1901
Melangyna arctica (Zetterstedt, 1838) [Hackman 1980]
Melangyna barbifrons (Fallén, 1817) [Hackman 1980]
Melangyna coei Nielsen, 1971 [Hippa 1972, Hackman 1980]
Melangyna compositarum (Verrall, 1873) [Hackman 1980]
Melangyna lasiophthalma (Zetterstedt, 1843) [Hackman 1980]
Melangyna lucifera Nielsen, 1980 [Hedström 1990]
Melangyna quadrimaculata (Verrall, 1873) [Hackman 1980]
Melangyna umbellatarum (Fabricius, 1794) [Hackman 1980]
MELIGRAMMA Frey, 1946
Meligramma guttata (Fallén, 1817) [Hackman 1980]
Meligramma triangulifera (Zetterstedt, 1843) [Hackman 1980]
MELISCAEVA Frey, 1946
Meliscaeva auricollis (Meigen, 1822) [Hackman 1980]
Meliscaeva cinctella (Zetterstedt, 1843) [Hackman 1980]
PARASYRPHUS Matsumura, 1917
Parasyrphus annulatus (Zetterstedt, 1838) [Hackman 1980]
Parasyrphus groenlandicus (Nielsen, 1910) [Haarto 2002a,b]
Parasyrphus lineolus (Zetterstedt, 1843) [Hackman 1980]
Parasyrphus macularis (Zetterstedt, 1843) [Hackman 1980]
Parasyrphus malinellus (Collin, 1952) [Hippa 1972, Hackman 1980]
Parasyrphus nigritarsis (Zetterstedt, 1843) [Hackman 1980]
Parasyrphus proximus Mutin, 1990 [Haarto and Kerppola 2004]
Parasyrphus punctulatus (Verrall, 1873) [Haarto and Kerppola 2004]
Parasyrphus tarsatus (Zetterstedt, 1838) [Hackman 1980]
? = *P. dryadis* (Holmgren, 1869)
Parasyrphus vittiger (Zetterstedt, 1843) [Hackman 1980]
SCAEVA Fabricius, 1805
Scaeva pyrastri (Linnaeus, 1758) [Hackman 1980]

Scaeva selenitica (Meigen, 1822) [Hackman 1980]
SPHAEROPHORIA Lepeletier & Serville, 1828
Sphaerophoria abbreviata Zetterstedt, 1859 [Haarto and Kerppola 2004]
Sphaerophoria bankowskae Goeldlin, 1989 [Haarto 1995, Stå hls 1995]
Sphaerophoria batava Goeldlin, 1974 [Stå hls 1995]
Sphaerophoria boreoalpina Goeldlin, 1989 [Stå hls 1995]
Sphaerophoria chongjini Bankowska, 1964 [Decleer 1989]
Sphaerophoria fatarum Goeldlin, 1989 [Hedström 1991, Haarto 1995, Stå hls 1995]
Sphaerophoria interrupta (Fabricius, 1805)
= *S. menthastri* (Linnaeus, 1758) [Hackman 1980]
Sphaerophoria kaa Violovitsh, 1960 [Haarto and Kerppola 2007b,a]
Sphaerophoria laurae Goeldlin, 1989 [Stå hls 1995]
Sphaerophoria loewi Zetterstedt, 1843 [Hackman 1980]
Sphaerophoria pallidula Mutin, 1999 [Haarto and Kerppola 2009]
Sphaerophoria philantha (Meigen, 1822) [Hippa 1972, Hackman 1980]
= *S. sarmatica* Bankowska, 1964 [Hippa 1972]
Sphaerophoria rueppelli (Wiedemann, 1830) [Hackman 1980]
Sphaerophoria scripta (Linnaeus, 1758) [Hackman 1980]
Sphaerophoria taeniata (Meigen, 1822) [Hackman 1980]
Sphaerophoria virgata Goeldlin, 1974 [Haarto 1995]
SYRPHUS Fabricius, 1775
Syrphus admirandus Goeldlin, 1996 [Haarto 1999]
Syrphus attenuatus Hine, 1922
= *S. pilisquamus* Ringdahl, 1928 [Hackman 1980]
Syrphus ribesii (Linnaeus, 1758) [Hackman 1980]
= *S. brevicinctus* Kanervo, 1938 [Kanervo 1938]
Syrphus sexmaculatus Zetterstedt, 1838 [Hackman 1980]
Syrphus torvus Osten Sacken, 1875 [Hackman 1980]
Syrphus vitripennis Meigen, 1822 [Hackman 1980]
= *S. rectus bretolensis* Goeldlin, 1996 [Haarto and Kerppola 2004]
XANTHOGRAMMA Schiner, 1860
Xanthogramma citrofasciatum (De Geer, 1776) [Hackman 1980]
= *X. festivum* auct. nec (Linnaeus, 1758)
Xanthogramma pedissequum (Harris, 1776) [Hackman 1980]
Xanthogramma stackelbergi Violovitsh, 1975 [Haarto and Kerppola 2007a]
MILESIINAE Rondani, 1845
tribe Cerioidini Wahlgren, 1909
CERIANA Rafinesque, 1815
Ceriana conopsoides (Linnaeus, 1758) [Hackman 1980]
SPHIXIMORPHA Rondani, 1850
Sphiximorpha subsessilis (Illiger *in* Rossi, 1807) [Haarto and Kerppola 2007b,a]
tribe Cheilosini Williston, 1885
CHEILOSIA Meigen, 1822
Cheilosia alba Vujić & Claussen, 2000 [Haarto and Kerppola 2004]
Cheilosia albipila Meigen, 1838 [Hackman 1980]
Cheilosia albitarsis (Meigen, 1822) [Hackman 1980]
Cheilosia alpina (Zetterstedt, 1838) [Hackman 1980]
Cheilosia angustigenis Becker, 1894 [Hackman 1980]
Cheilosia barbata Loew, 1857 [Hackman 1980]
= *C. honesta* Rondani, 1868

Cheilosia barovskii (Stackelberg, 1930)
Cheilosia carbonaria Egger, 1860 [Hackman 1980]
Cheilosia chrysocoma (Meigen, 1822) [Hackman 1980]
Cheilosia cynocephala Loew, 1840 [Hackman 1980]
Cheilosia flavipes (Panzer, 1798) [Hackman 1980]
Cheilosia flavissima Becker, 1894 [Haarto and Kerppola 2007a]
= *C. pallipes* auct. nec Loew, 1863 [Hackman 1980]
Cheilosia fraterna (Meigen, 1830) [Hackman 1980]
Cheilosia frontalis Loew, 1857 [Hackman 1980]
Cheilosia gigantea (Zetterstedt, 1838) [Hackman 1980]
= *C. gracilis* Hellén, 1914 [Hackman 1980]
Cheilosia grossa (Fallén, 1817) [Hackman 1980]
Cheilosia illustrata (Harris, 1780)
Cheilosia impressa Loew, 1840 [Haarto and Kerppola 2004]
Cheilosia ingerae Nielsen & Claussen, 2001 [Nielsen and Claussen 2001]
Cheilosia lasiopa Kowarz, 1885 [Haarto and Kerppola 2004]
= *C. honesta* auct. nec Rondani, 1868 [Hackman 1980]
Cheilosia latifrons (Zetterstedt, 1843)
= *C. intonsa* Loew, 1857 [Hackman 1980]
Cheilosia longula (Zetterstedt, 1838) [Hackman 1980]
= *C. plumulifera* Loew, 1857 [Haarto and Kerppola 2007b]
= *C. soror* misid.[Hackman 1980]
Cheilosia melanopa (Zetterstedt, 1843) [Hackman 1980]
Cheilosia morio (Zetterstedt, 1838) [Haarto and Kerppola 2004]
Cheilosia mutabilis (Fallén, 1817) [Hackman 1980]
= *C. ruralis* (Meigen, 1822)
Cheilosia naruska Haarto & Kerppola, 2007 [Haarto et al. 2007, Haarto and Kerppola 2007a]
Cheilosia nebulosa Verrall, 1871
= *C. langhofferi* Becker, 1894 [Hackman 1980]
Cheilosia nigripes (Meigen, 1822) [Hackman 1980]
Cheilosia pagana (Meigen, 1822) [Hackman 1980]
Cheilosia proxima (Zetterstedt, 1843) [Hackman 1980]
Cheilosia psilophthalma Becker, 1894 [Haarto and Kerppola 2004]
Cheilosia pubera (Zetterstedt, 1838) [Hackman 1980]
Cheilosia reniformis Hellén, 1930 [Haarto and Kerppola 2007b,a]
Cheilosia rufimana Becker, 1894 [Stå hls 1995]
Cheilosia sahlbergi Becker, 1894 [Val 1929, Hackman 1980]
Cheilosia scutellata (Fallén, 1817) [Hackman 1980]
Cheilosia semifasciata Becker, 1894 [Hackman 1980]
Cheilosia sootryeni Nielsen, 1970 [Haarto 2000]
Cheilosia urbana (Meigen, 1822)
= *C. ruralis* (Meigen, 1822) [Hackman 1980]
= *C. praecox* (Zetterstedt, 1843)
= *C. globulipes* Becker, 1894
Cheilosia uviformis Becker, 1894
= *C. argentifrons* Hellen, 1914 [Hackman 1980]
Cheilosia variabilis (Panzer, 1798) [Hackman 1980]
Cheilosia velutina Loew, 1840 [Hackman 1980]
Cheilosia vernalis (Fallén, 1817) [Hackman 1980]

= *C. rotundicornis* Hellen, 1914 [Hackman 1980]
= *C. rotundiventris* Becker, 1894 [Ano 1956, Stå hls et al. 2008]
= *C. ruficollis* Becker, 1894
Cheilosia vicina (Zetterstedt, 1849) [Hackman 1980]
= *C. nasutula* Becker, 1894 [Lucas et al. 1995, Hackman 1980]
Cheilosia vulpina (Meigen, 1822) [Haarto and Kerppola 2007b,a]
Cheilosia sp. A Haarto & Kerppola, 2007 [Haarto and Kerppola 2007b,a]
FERDINANDEA Rondani, 1844
Ferdinandea cuprea (Scopoli, 1763) [Hackman 1980]
RHINGIA Scopoli, 1763
Rhingia borealis Ringdahl, 1928
= *R. austriaca* auct. nec Meigen, 1830 [Hackman 1980]
Rhingia campestris Meigen, 1822 [Hackman 1980]
tribe Chrysogasterini Shannon, 1922
BRACHYOPA Meigen, 1822
Brachyopa cinerea Wahlberg, 1844 [Hackman 1980]
Brachyopa dorsata Zetterstedt, 1837 [Hackman 1980]
Brachyopa obscura Thompson & Torp, 1982 [Haarto and Kerppola 2004]
Brachyopa pilosa Collin, 1939 [Hippa 1972, Hackman 1980]
= *B. bicolor* misid.[Stå hls 1995]
Brachyopa testacea (Fallén, 1817)
= *B. conica* misid.[Hackman 1980, Thompson 1980]
Brachyopa vittata Zetterstedt, 1843 [Haarto and Kerppola 2007b,a]
Brachyopa zhelochovtsevi Mutin, 1998 [Haarto and Kerppola 2009]
CHRYSOGASTER Meigen, 1803
Chrysogaster coemiteriorum (Linnaeus, 1758)
= *C. cemiteriorum* misspelling
= *C. chalybeata* Meigen, 1822 [Hackman 1980]
Chrysogaster solstitialis (Fallén, 1817) [Hackman 1980]
Chrysogaster virescens Loew, 1854 [Haarto and Kerppola 2004]
CHRYSOSYRPHUS Sedman, 1965
= ***Helleniola*** Stackelberg, 1965
Chrysosyrphus nasutus (Zetterstedt, 1838) [Haarto and Kerppola 2004]
Chrysosyrphus niger (Zetterstedt, 1843) [Haarto and Kerppola 2004]
= *Myolepta nigra* mistake[Hackman 1980, 1982]
HAMMERSCHMIDTIA Schummel, 1834
Hammerschmidtia ferruginea (Fallén, 1817) [Hackman 1980]
Hammerschmidtia ingrica Stackelberg, 1952 [Kerppola 2011]
LEJOGASTER Rondani, 1857
Lejogaster metallina (Fabricius, 1781) [Hackman 1980]
Lejogaster tarsata (Meigen, 1822)
= *L. splendida* (Meigen, 1822) [Hackman 1980, Valle 1938]
MELANOGASTER Rondani, 1857
Melanogaster aerosa (Loew, 1843)
= *M. macquarti* (Loew, 1843) [Hackman 1980]
= *M. viduata* misid.[Hackman 1980, Haarto and Kerppola 2004]
Melanogaster parumplicata (Loew, 1840) [Haarto and Kerppola 2009]
NEOASCIA Williston, 1886
sg. ***Neoascia*** Williston, 1886
Neoascia podagrica (Fabricius, 1775) [Hackman 1980]

Neoascia tenur (Harris, 1780)
= *N. dispar* (Meigen, 1822) [Hackman 1980]
= *N. lapponica* Kanervo, 1934
= *N. splendida* Kanervo, 1934
sg. ***Neoasciella*** Stackelberg, 1970
Neoascia geniculata (Meigen, 1822) [Hackman 1980]
Neoascia interrupta (Meigen, 1822) [Hackman 1980]
Neoascia meticulosa (Scopoli, 1763)
= *N. aenea* (Meigen, 1822) [Hippa 1967, Hackman 1980]
Neoascia obliqua Coe, 1940 [Haarto and Kerppola 2004]
Neoascia subchalybea Curran, 1925
= *N. petsamoensis* Kanervo, 1934 [Hackman 1982]
ORTHONEVRA Macquart, 1829
Orthonevra elegans (Meigen, 1822) [Hackman 1980]
Orthonevra erythrogona (Malm, 1863) [Hackman 1980]
Orthonevra geniculata (Meigen, 1830) [Hackman 1980]
= *O. linnaniemii* Kanervo, 1934
Orthonevra intermedia Lundbeck, 1916 [Hackman 1980, Thompson and Torp 1982]
= *O. rossica* Stackelberg, 1953
Orthonevra nobilis (Fallén, 1817) [Hackman 1980]
Orthonevra plumbago (Loew, 1840) [Haarto and Kerppola 2004]
= *O. brevicornis* misid.[Hackman 1980]
Orthonevra stackelbergi Thompson & Torp, 1982 [Haarto and Kerppola 2004]
= *O. intermedia* misid.
SPHEGINA Meigen, 1822
sg. ***Sphegina*** Meigen, 1822
Sphegina clunipes (Fallén, 1816) [Hackman 1980]
Sphegina elegans Schummel, 1843 [Polevoi and Stå hls 1994]
= *S. kimakowiczi* misid.[Hellén 1940, Hackman 1980]
Sphegina montana Becker, 1921 [Hackman 1982]
= *S. violovitshi* misid.[Hac 1970, Hackman 1980, 1982]
= *S. verecunda* misid.[Hac 1970]
Sphegina spheginea (Zetterstedt, 1838) [Hackman 1980]
sg. ***Asiosphegina*** Stackelberg, 1975
Sphegina sibirica Stackelberg, 1953 [Hac 1970, Hippa 1972, Hackman 1980]
tribe Eristalini Newman, 1834
ANASIMYIA Schiner, 1864
= ***Eurimyia*** Bigot, 1883
Anasimyia contracta Claussen & Torp, 1980 [Claussen and Torp 1980]
Anasimyia interpuncta (Harris, 1776) [Bartsch 2000]
Anasimyia lineata (Fabricius, 1787) [Hackman 1980]
Anasimyia lunulata (Meigen, 1822) [Hackman 1980]
Anasimyia transfuga (Linnaeus, 1758) [Hackman 1980]
ERISTALINUS Rondani, 1845
Eristalinus aeneus (Scopoli, 1763) [Haarto 1999]
Eristalinus sepulchralis (Linnaeus, 1758) [Hackman 1980]
ERISTALIS Latreille, 1804
Eristalis abusiva Collin, 1931 [Hackman 1980]
Eristalis alpina (Panzer, 1798) [Hippa et al. 2001]
Eristalis anthophorina (Fallén, 1817) [Hackman 1980]

Eristalis arbustorum (Linnaeus, 1758) [Hackman 1980]
= *E. nemorum* auct. nec (Linnaeus, 1758)
Eristalis cryptarum (Fabricius, 1794) [Hackman 1980]
Eristalis fratercula Zetterstedt, 1838 [Hackman 1980]
= *E. vallei* Kanervo, 1934 [Hackman 1980]
Eristalis gomojunovae Violovitsh, 1977 [Nielsen 1995] modified19.4.2007
= *E. fratercula* auct. nec Zetterstedt, 1838 [Hackman 1980]
Eristalis hirta Loew, 1866
= *E. tundrarum* Frey, 1932 [Frey 1932b, Hackman 1980]
Eristalis horticola (De Geer, 1776) [Hackman 1980]
= *E. lineata* (Harris, 1776)
Eristalis intricaria (Linnaeus, 1758) [Hackman 1980]
Eristalis nemorum (Linnaeus, 1758) [Hackman 1980]
= *E. interrupta* (Poda, 1761)
Eristalis obscura Loew, 1866
= *E. pseudorupium* Kanervo, 1938 [Hippa et al. 2001]
= *E. vitripennis* Strobl, 1893 [Val 1929, Hackman 1980]
Eristalis oestracea (Linnaeus, 1758) [Hackman 1980]
Eristalis pertinax (Scopoli, 1763) [Brander 1971, Hackman 1980]
Eristalis picea (Fallén, 1817) [Val 1929, Hackman 1980]
Eristalis rupium (Fabricius, 1805) [Hackman 1980]
Eristalis similis (Fallén, 1817)
= *E. pratorum* (Meigen, 1822) [Hackman 1980]
Eristalis tenax (Linnaeus, 1758) [Hackman 1980]
HELOPHILUS Meigen, 1822
Helophilus affinis Wahlberg, 1844 [Hackman 1980]
Helophilus bottnicus Wahlberg, 1844 [Haarto and Kerppola 2004]
Helophilus groenlandicus (Fabricius, 1780) [Hackman 1980]
Helophilus hybridus Loew, 1846 [Hackman 1980]
Helophilus lapponicus Wahlberg, 1844 [Hackman 1980]
= *H. borealis* Staeger, 1845
Helophilus pendulus (Linnaeus, 1758) [Hackman 1980]
Helophilus trivittatus (Fabricius, 1805)
= *H. parallelus* (Harris, 1776) [Hackman 1980]
MALLOTA Meigen, 1822
Mallota megilliformis (Fallén, 1817) [Hackman 1980]
Mallota tricolor Loew, 1871
MYATHROPA Rondani, 1845
Myathropa florea (Linnaeus, 1758) [Hackman 1980]
PARHELOPHILUS Girschner, 1897
Parhelophilus consimilis (Malm, 1863) [Hackman 1980]
Parhelophilus frutetorum (Fabricius, 1775) [Hackman 1980]
Parhelophilus versicolor (Fabricius, 1794) [Hackman 1980]
tribe Eumerini Smirnov, 1924
EUMERUS Meigen, 1822
Eumerus flavitarsis Zetterstedt, 1843 [Hackman 1980]
Eumerus funeralis Meigen, 1822
= *E. tuberculatus* Rondani, 1857 [Hackman 1980, Kanervo 1935]
Eumerus grandis Meigen, 1822
= *E. annulatus* (Panzer, 1798) [Hackman 1980]

Eumerus ruficornis Meigen, 1822 [Hackman 1980]
Eumerus sabulonum (Fallén, 1817) [Hackman 1980]
Eumerus strigatus (Fallén, 1817) [Hackman 1980]
tribe Merodontini Edwards, 1915
MERODON Meigen, 1803
Merodon equestris (Fabricius, 1794) [Hackman 1980]
tribe Pelecocerini Williston, 1887
PELECOCERA Meigen, 1822
= ***Chamaesyrphus*** Mik, 1895
Pelecocera caledonica Collin, 1940 [Stå hls 1995]
Pelecocera lusitanica Mik, 1898 [Hackman 1980]
Pelecocera scaevoides (Fallén, 1817) [Hackman 1980]
Pelecocera tricincta Meigen, 1822 [Val 1929, Hackman 1980]
tribe Pipizini Williston, 1885
CRYPTOPIPIZA Mutin, 1998
Cryptopipiza notabila (Violovitsh, 1985) [Stå hls and Vujić 2009]
HERINGIA Rondani, 1856
sg. ***Heringia*** Rondani, 1856
Heringia heringi (Zetterstedt, 1843) [Hackman 1980]
NEOCNEMODON Goffe, 1944
= ***Cnemodon*** Egger, 1865 preocc.
Neocnemodon fulvimanus (Zetterstedt, 1843) [Haarto and Kerppola 2007b]
Neocnemodon larusi (Vujić, 1999) [Haarto and Kerppola 2007b,a]
= *N. fulvimanus* auct. nec (Zetterstedt, 1843) [Hackman 1980]
Neocnemodon latitarsis (Egger, 1865) [Haarto and Kerppola 2009]
Neocnemodon pubescens Delucchi & Pschorn-Walcher, 1955 [Hippa 1972, Hackman 1980]
Neocnemodon verrucula (Collin, 1931) [Hippa 1972, Hackman 1980]
Neocnemodon vitripennis (Meigen, 1822) [Hackman 1980]
PIPIZA Fallén, 1810
Pipiza accola Violovitsh, 1985 [Haarto and Kerppola 2007b,a]
Pipiza austriaca Meigen, 1822 [Hackman 1980]
Pipiza fasciata Meigen, 1822 [Kerppola 2013]
Pipiza festiva Meigen, 1822 [Haarto and Kerppola 2004]
Pipiza lugubris (Fabricius, 1775) [Hackman 1980, Haarto and Winqvist 2014]
Pipiza luteitarsis Zetterstedt, 1843 [Hippa 1972, Hackman 1980]
Pipiza noctiluca (Linnaeus, 1758) [Hackman 1980]
Pipiza notata Meigen, 1822 [Hackman 1980]
= *P. bimaculata* Meigen, 1822 [Hackman 1980]
Pipiza quadrimaculata (Panzer, 1804) [Hackman 1980]
PIPIZELLA Rondani, 1856
Pipizella certa Violovitsh, 1981 [Haarto and Kerppola 2004]
= *P. brevis* auct. nec Lucas, 1976 [Kuznetzov 1987, Haarto and Kerppola 2004]
Pipizella obscura Van Steenis & Lucas, 2011 [van Steenis and Lucas 2011]
Pipizella viduata (Linnaeus, 1758) [Haarto 1995]
= *P. virens* misid.[Hackman 1980, Haarto and Kerppola 2004]
TRICHOPSOMYIA Williston, 1888
Trichopsomyia flavitarsis (Meigen, 1822)
Trichopsomyia joratensis Goeldlin, 1997 [Haarto and Kerppola 2004]
TRIGLYPHUS Loew, 1840

Triglyphus primus Loew, 1840 [Haarto and Kerppola 2004]
tribe Sericomyiini Rondani, 1845
SERICOMYIA Meigen, 1803
Sericomyia arctica Schirmer, 1913 [Hackman 1980]
Sericomyia jakutica (Stackelberg, 1927) [Haarto and Kerppola 2004]
Sericomyia lappona (Linnaeus, 1758) [Hackman 1980]
Sericomyia nigra Portschinsky, 1872 [Hackman 1980]
Sericomyia silentis (Harris, 1776) [Hackman 1980]
tribe Volucellini Newman, 1834
VOLUCELLA Geoffroy, 1762
Volucella bombylans (Linnaeus, 1758) [Hackman 1980]
Volucella inanis (Linnaeus, 1758) [Hackman 1980]
Volucella pellucens (Linnaeus, 1758) [Hackman 1980]
tribe Xylotini Bigot, 1883
BLERA Billberg, 1820
= ***Cynorhina*** Williston, 1887
Blera eoa (Stackelberg 1928) [Kerppola and Raekunnas 2012]
Blera fallax (Linnaeus, 1758) [Hackman 1980]
BRACHYPALPOIDES Hippa, 1978
Brachypalpoides lentus (Meigen, 1822) [Hackman 1980]
BRACHYPALPUS Macquart, 1834
Brachypalpus laphriformis (Fallén, 1816) [Hackman 1980]
CHALCOSYRPHUS Curran, 1925
Chalcosyrphus jacobsoni (Stackelberg, 1921) [Hackman 1980, Kangas 1946]
Chalcosyrphus nemorum (Fabricius, 1805) [Hackman 1980]
Chalcosyrphus nigripes (Zetterstedt, 1838) [Hackman 1980]
Chalcosyrphus piger (Fabricius, 1794) [Hackman 1980]
Chalcosyrphus rufipes (Loew, 1873) [Bartsch et al. 2010]
Chalcosyrphus valgus (Gmelin, 1790)
= *C. femoratus* auct. nec Linnaeus, 1758 [Hackman 1980]
CRIORHINA Meigen, 1822
Criorhina asilica (Fallén, 1816) [Hackman 1980]
LEJOTA Rondani, 1857
Lejota ruficornis (Zetterstedt, 1843) [Hackman 1980]
SPHECOMYIA Latreille, 1829
Sphecomyia vespiformis Gorski, 1852 [Hackman 1980]
SPILOMYIA Meigen, 1803
Spilomyia diophthalma (Linnaeus, 1758) [Hackman 1980]
SYRITTA Lepeletier & Serville, 1828
Syritta pipiens (Linnaeus, 1758) [Hackman 1980]
TEMNOSTOMA Lepeletier & Serville, 1828
Temnostoma angustistriatum Krivosheina, 2002 [Krivosheina and Stå hls 2003]
= *T. bombylans* auct. nec (Fabricius, 1805) [Hackman 1980, Krivosheina and Stå hls 2003]
Temnostoma apiforme (Fabricius, 1794) [Hackman 1980]
Temnostoma carens Gaunitz, 1936 [Krivosheina 2003]
Temnostoma sericomyiaeforme (Portschinsky, 1886) [Krivosheina 2004b, Haarto and Kerppola 2004]
Temnostoma vespiforme (Linnaeus, 1758) [Hackman 1980]
TROPIDIA Meigen, 1822

Tropidia fasciata Meigen, 1822 [Hackman 1980]
Tropidia scita (Harris, 1780) [Hackman 1980]
XYLOTA Meigen, 1822
Xylota caeruleiventris Zetterstedt, 1838 [Hackman 1980]
Xylota florum (Fabricius, 1805) [Hackman 1980]
Xylota ignava (Panzer, 1798) [Hackman 1980]
Xylota jakutorum Bagatshanova, 1980 [Bartsch et al. 2002]
= *X. caeruleiventris* auct. nec Zetterstedt, 1838
Xylota meigeniana Stackelberg, 1964 [Hippa 1972, Hackman 1980]
Xylota segnis (Linnaeus, 1758) [Hackman 1980]
Xylota suecica (Ringdahl, 1943) [Hippa 1972, Hackman 1980]
Xylota sylvarum (Linnaeus, 1758) [Hackman 1980]
Xylota tarda Meigen, 1822 [Hackman 1980]
Xylota triangularis Zetterstedt, 1838 [Hackman 1980]
Xylota xanthocnema Collin, 1939 [Haarto and Kerppola 2007a]
MICRODONTINAE Rondani, 1845
tribe Microdontini Rondani, 1845
MICRODON Meigen, 1803
Microdon analis (Macquart, 1842)
= *M. eggeri* Mik, 1897 [Hackman 1980]
Microdon miki Doczkal & Schmid, 1999
= *M. latifrons* auct. nec (Loew, 1856) [Hackman 1980]
Microdon mutabilis (Linnaeus, 1758) [Hackman 1980]

Excluded species

Microdon devius (Linnaeus, 1761) [Hackman 1980, Stå hls 1995]
Microdon devius (Linnaeus, 1761)
Neoascia annexa (Müller, 1776)
Paragus punctulatus Zetterstedt, 1838
Parasyrphus relictus (Zetterstedt, 1838) [Hackman 1980, Haarto and Kerppola 2007a]
Sphaerophoria sp. B [Haarto and Kerppola 2007b]
Temnostoma meridionale Krivosheina & Mamayev, 1962

CONOPIDAE Latreille, 1802

Supporting references for *Kahanpää J, Stuke J-H (2014) Checklist of the superfamiles Conopoidea, Diopsoidea and Nerioidea of Finland (Insecta: Diptera). In: Kahanpää J, Salmela (Eds) J. Checklist of the Diptera of Finland. ZooKeys @@: @-@*

CONOPINAE Latreille, 1802
CONOPS Linnaeus, 1758
sg. ***Conops*** Linnaeus, 1758
Conops quadrifasciatus De Geer, 1776 [Hackman 1980]
Conops strigatus Wiedemann, 1824 [Kaisila 1960, Hackman 1980]
Conops vesicularis Linnaeus, 1761 [Hackman 1980]
PHYSOCEPHALA Schiner, 1861
Physocephala nigra (De Geer, 1776) [Hackman 1980]
ZODIONINAE Rondani, 1856
ZODION Latreille, 1796
Zodion cinereum (Fabricius, 1794) [Hackman 1980]
= *Z. notatum* (Meigen, 1804) [Hackman 1980, Mei and Stuke 2008]
SICINAE Zimina, 1960
SICUS Scopoli, 1763
Sicus ferrugineus (Linnaeus, 1761) [Hackman 1980]
MYOPINAE Macquart, 1834
MYOPA Fabricius, 1775
Myopa buccata (Linnaeus, 1758) [Hackman 1980]
Myopa fasciata Meigen, 1804 [Hackman 1980]
Myopa hirsuta Stuke & Clements 2008 [Stuke and Clements 2008]
= *M. strandi* auct. nec Duda, 1940 [Hackman 1980]
Myopa occulta Wiedemann, 1824 [Hackman 1980]
= *M. nigrifrons* von Bonsdorff, 1866
Myopa tessellatipennis Motschulsky, 1859
= *M. polystigma* auct. nec Rondani, 1857 [Hackman 1980]
Myopa testacea (Linnaeus, 1767) [Hackman 1980, Clements et al. 2008]
Myopa vicaria Walker, 1849
= *M. villosa* Ringdahl, 1945
= *M. strandi* Duda, 1940
MYOPOTTA Zimina, 1969
Myopotta pallipes (Wiedemann, 1824) [Hackman 1980]
THECOPHORA Rondani, 1845
= ***Occemyia*** Robineau-Desvoidy, 1853
Thecophora cinerascens (Meigen, 1804)
= *T pusilla* (Meigen, 1824) [Kahanpää and Winqvist 2003]
= *T. atra* misid.[Hackman 1980]
Thecophora distincta (Wiedemann, 1824) [Hackman 1980]
= *T. melanopa* misid.[Kaisila 1960, Hackman 1980]
Thecophora fulvipes (Robineau-Desvoidy, 1830) [Hackman 1980]
= *T. sundewalli* (Zetterstedt, 1844)
Thecophora jakutica Zimina, 1974 [Kahanpää 2007a]
= *T. atra* misid.[Hackman 1980]
DALMANNIINAE Hendel, 1916

DALMANNIA Robineau-Desvoidy, 1830
Dalmannia dorsalis (Fabricius, 1794) [Kanervo 1928]
= *D. punctata* misid.[Hackman 1980]

Excluded species

Dalmannia marginata (Meigen, 1824)
Dalmannia punctata (Fabricius, 1794) [Hackman 1980]

MICROPEZIDAE Blanchard, 1840

Supporting references for *Kahanpää J, Stuke J-H (2014) Checklist of the superfamiles Conopoidea, Diopsoidea and Nerioidea of Finland (Insecta: Diptera). In: Kahanpää J, Salmela (Eds) J. Checklist of the Diptera of Finland. ZooKeys @@: @-@*

CALOBATINAE Bigot, 1853
CALOBATA Meigen, 1803
Calobata petronella (Linnaeus, 1761) [Hackman 1980]
NERIA Robineau-Desvoidy, 1830
Neria cibaria (Linnaeus, 1761) [Hackman 1980]
Neria commutata (Czerny, 1930)
= *N. nigricornis* auct. nec (Zetterstedt, 1838) [Hackman 1980]
Neria ephippium (Fabricius, 1794) [Hackman 1980]
Neria nigricornis (Zetterstedt, 1838)
= *N. helleni* (Frey, 1918)
= *N. nitidicollis* (Frey, 1947)
MICROPEZINAE Blanchard, 1840
MICROPEZA Meigen, 1803
Micropeza corrigiolata (Linnaeus, 1767) [Hackman 1980]

PSEUDOPOMYZIDAE McAlpine, 1966

Supporting references for *Kahanpää J, Stuke J-H (2014) Checklist of the superfamiles Conopoidea, Diopsoidea and Nerioidea of Finland (Insecta: Diptera). In: Kahanpää J, Salmela (Eds) J. Checklist of the Diptera of Finland. ZooKeys @@: @-@*

PSEUDOPOMYZA Strobl, 1893
Pseudopomyza atrimana (Meigen, 1830) [Frey 1952, Hackman 1980]

MEGAMERINIDAE Hendel, 1913

Supporting references for *Kahanpää J, Stuke J-H (2014) Checklist of the superfamiles Conopoidea, Diopsoidea and Nerioidea of Finland (Insecta: Diptera). In: Kahanpää J, Salmela (Eds) J. Checklist of the Diptera of Finland. ZooKeys @@: @-@*

MEGAMERINA Rondani, 1861
= ***Lissa*** Meigen, 1826 preocc.
Megamerina dolium (Fabricius, 1805) [Hackman 1980]
= *M. loxocerina* (Fallén, 1820)

PSILIDAE Macquart, 1835

Supporting references for *Kahanpää J, Stuke J-H (2014) Checklist of the superfamiles Conopoidea, Diopsoidea and Nerioidea of Finland (Insecta: Diptera). In: Kahanpää J, Salmela (Eds) J. Checklist of the Diptera of Finland. ZooKeys @@: @-@*

CHYLIZINAE Rondani, 1856
CHYLIZA Fallén, 1820
sg. ***Chyliza*** Fallén, 1820
Chyliza annulipes Macquart, 1835
= *C. fuscipennis* auct. nec (Robineau-Desvoidy, 1830) [Hackman 1980]
Chyliza leptogaster (Panzer, 1798)
= *C. scutellata* (Fabricius, 1798) [Hackman 1980]
Chyliza nova Collin, 1944 [Haarto and Winqvist 2014]
Chyliza vittata Meigen, 1826 [Hackman 1980]
PSILINAE Macquart, 1835
tribe Loxocerini Macquart, 1835
IMANTIMYIA Frey, 1925
Imantimyia albiseta (Schrank, 1803) [Kahanpää 2013d]
= *L. ichneumonea* (Linnaeus, 1758) nom. dubium
Imantimyia fulviventris (Meigen, 1826) [Hackman 1980]
Imantimyia nigrifrons (Macquart, 1835) [Hackman 1980]
Imantimyia sylvatica (Meigen, 1826) [Hackman 1980]
LOXOCERA Meigen, 1803
sg. ***Loxocera*** Meigen, 1803
Loxocera aristata (Panzer, 1801)
= *L. ichneumonea* (Linnaeus, 1758) nom. dubiumHackman [1980]
tribe Psilini Macquart, 1835
CHAMAEPSILA Hendel, 1917
sg. ***Chamaepsila*** Hendel, 1917
Chamaepsila atra (Meigen, 1826) [Hackman 1980]
Chamaepsila bicolor (Meigen, 1826) [Hackman 1980]
= *C. nigromaculata* (Strobl, 1909) [Hackman 1980, Carles-Tolrá 1993]
Chamaepsila buccata (Fallén, 1826) [Hackman 1980]
= *C. gracilis* (Meigen, 1826) [Hackman 1980]
Chamaepsila humeralis (Zetterstedt, 1847) [Hackman 1980]
Chamaepsila limbatella (Zetterstedt, 1847) [Hackman 1980]
Chamaepsila morio (Zetterstedt, 1835) [Hackman 1980]
Chamaepsila nigra (Fallén, 1820) [Hackman 1980]
Chamaepsila nigricornis (Meigen, 1826) [Hellén 1946, Hackman 1980]
Chamaepsila nigrosetosa Frey, 1925
Chamaepsila obscuritarsis (Loew, 1856) [Hackman 1980]
Chamaepsila pallida (Fallén, 1820) [Hackman 1980]
Chamaepsila pectoralis (Meigen, 1826) [Hackman 1980]
Chamaepsila rosae (Fabricius, 1794) [Hackman 1980]
Chamaepsila rufa (Meigen, 1826) [Hackman 1980]
Chamaepsila unilineata (Zetterstedt, 1847)
PSILA Meigen, 1803
sg. ***Freyopsila*** Shatalkin, 1986

Psila sibirica (Frey, 1925) [Kahanpää 2013d]
sg. ***Psila*** Meigen, 1803
Psila fimetaria (Linnaeus, 1761) [Hackman 1980]
Psila merdaria Collin, 1944 [Tiensuu 1964, Hackman 1980]
PSILOSOMA Zetterstedt, 1860
Psilosoma audouini (Zetterstedt, 1835) [Hackman 1980]
Psilosoma lefebvrei (Zetterstedt, 1835) [Hackman 1980]

STRONGYLOPHTHALMYIIDAE Hendel, 1917

Supporting references for *Kahanpää J, Stuke J-H (2014) Checklist of the superfamiles Conopoidea, Diopsoidea and Nerioidea of Finland (Insecta: Diptera). In: Kahanpää J, Salmela (Eds) J. Checklist of the Diptera of Finland. ZooKeys @@: @-@*

STRONGYLOPHTHALMYIA Heller, 1902
Strongylophthalmyia pictipes Frey, 1935 [Frey 1935, 1956, Hackman 1982]
Strongylophthalmyia ustulata (Zetterstedt, 1847) [Hackman 1980]

TANYPEZIDAE Rondani, 1856

Supporting references for *Kahanpää J, Stuke J-H (2014) Checklist of the superfamiles Conopoidea, Diopsoidea and Nerioidea of Finland (Insecta: Diptera). In: Kahanpää J, Salmela (Eds) J. Checklist of the Diptera of Finland. ZooKeys @@: @-@*

TANYPEZA Fallén, 1820
Tanypeza longimana Fallén, 1820 [Hackman 1980]

EURYGNATHOMYIIDAE Czerny, 1934

Supporting references for *Kahanpää J, Winqvist K (2014) Checklist of the Diptera superfamilies Tephritoidea and Sciomyzoidea of Finland (Insecta). In: Kahanpää J, Salmela J (Eds) Checklist of the Diptera of Finland. ZooKeys @@: @-@. doi: 10.3897/zookeys.??.7143*

EURYGNATHOMYIA Czerny, 1904
Eurygnathomyia bicolor (Zetterstedt, 1837) [Hackman 1980, Morge 1967]

LONCHAEIDAE Rondani, 1856

Supporting references for *Kahanpää J, Winqvist K (2014) Checklist of the Diptera superfamilies Tephritoidea and Sciomyzoidea of Finland (Insecta). In: Kahanpää J, Salmela J (Eds) Checklist of the Diptera of Finland. ZooKeys @@: @-@. doi: 10.3897/zookeys.??.7143*

DASIOPINAE Morge, 1963
tribe Dasiopini Morge, 1963
DASIOPS Rondani, 1856
Dasiops appendiculus Morge, 1959 [Hackman 1980]
Dasiops facialis Collin, 1953 [Hackman 1956, 1980]
Dasiops mucronatus Morge, 1959
= *D. hennigi* misid.[Hackman 1980]
= *D. latiterebra* misid.[Hackman 1956]
Dasiops occultus Collin, 1953 [Winqvist and Kahanpää 2007]
= *D. albiceps* (Frey, 1930) preocc.[Frey 1930]
Dasiops perpropinquus Morge, 1959 [Hackman 1980]
Dasiops spatiosus (Becker, 1895) [Hackman 1956, 1980]
Dasiops trichosternalis Morge, 1959 [Hackman 1980]
EAROMYIINAE Morge, 1963
CHAETOLONCHAEA Czerny, 1934
Chaetolonchaea pallipennis (Zetterstedt, 1855) [Winqvist and Kahanpää 2007]
= *C. dasyops* misid.[Hackman 1956, 1980]
EAROMYIA Zetterstedt, 1842
Earomyia lonchaeoides Zetterstedt, 1848 [Hackman 1956, 1980]
Earomyia schistopyga Collin, 1953 [Hackman 1956, 1980]
Earomyia viridana (Meigen, 1826) [Hackman 1956, 1980]
PROTEAROMYIA McAlpine, 1962
= ***Priscoearomyia*** Morge, 1963
Protearomyia nigra (Meigen, 1826) [Hackman 1956, 1980]
LONCHAEINAE Rondani, 1856
tribe Lonchaeini Rondani, 1856
LONCHAEA Fallén, 1820
Lonchaea affinis Malloch, 1920
= *L. laxa* auct. necCollin, 1953 [Hackman 1956, 1980]
Lonchaea albigena Collin, 1953 [Hackman 1956]
Lonchaea albitarsis Zetterstedt, 1838 [Hackman 1956, 1980]
? *Lonchaea bruggeri* Morge, 1967 [Kovalev 1981]
Lonchaea bukowskii Czerny, 1934 [MacGowan and Rotheray 2008]
Lonchaea caledonica MacGowan & Rotheray, 2000 [MacGowan and Rotheray 2000]
= *L. laticornis* auct. necMeigen, 1826 [Hackman 1956, 1980, MacGowan and Rotheray 2000]
Lonchaea carpathica Kovalev, 1974 [Haarto and Winqvist 2014]
Lonchaea chorea (Fabricius, 1781) [Hackman 1956, 1980]
Lonchaea collini Hackman, 1956 [Hackman 1956, 1980]
? *Lonchaea contigua* Collin, 1953 [Hackman 1956, 1980]
Lonchaea corusca Czerny, 1934 [Hackman 1980, Nuorteva 1967]
= *L. alni* Ringdahl, 1947 [Hackman 1956, 1980]
= *L. lauta* Collin, 1953

= *L. britteni* Collin, 1953 [Hackman 1956, 1980]
Lonchaea defecta McAlpine, 1964 [Nuorteva 1967, Hackman 1980]
Lonchaea deutschi Zetterstedt, 1838 [Hackman 1956, 1980]
= *L. sarekensis* Frey, 1916 [Hackman 1956, 1980]
? *Lonchaea difficilis* Hackman, 1956 [Hackman 1956, 1980]
Lonchaea fraxina MacGowan & Rotheray, 2000 [MacGowan and Rotheray 2000]
Lonchaea freyi Czerny, 1934 [Hackman 1956, 1980]
Lonchaea fugax Becker, 1895 [Hackman 1980]
= *L. cariecola* Czerny, 1934 [Hackman 1956]
Lonchaea hackmani Kovalev, 1981 [Kovalev 1981]
= *L. peregrina* auct. nec Becker, 1895 [Hackman 1956, 1980, Kovalev 1981]
Lonchaea ipsiphaga McAlpine, 1964 [Kovalev 1981]
= *L. maniola* misid.[Nuorteva 1967, Hackman 1980, Kovalev 1984]
Lonchaea limatula Collin, 1953 [Hackman 1956, Winqvist and Kahanpää 2007]
= *L. flavidipennis* auct. nec Zetterstedt, 1847 [Kaisila 1952, Hackman 1956, 1980]
Lonchaea nitens (Bigot, 1885) [Hackman 1956, 1980]
= *L. krogerusi* Czerny, 1934
Lonchaea palposa Zetterstedt, 1847 [Hackman 1956, 1980]
Lonchaea patens Collin, 1953 [Hackman 1956, 1980]
Lonchaea ragnari Hackman, 1956 [Hackman 1956, 1980]
Lonchaea scutellaris Rondani, 1874 [Winqvist and Kahanpää 2007]
Lonchaea sororcula Hackman, 1956 [Hackman 1956, 1980]
Lonchaea stackelbergi Czerny, 1934 [Hackman 1956, 1980]
Lonchaea subneatosa Kovalenko, 1974 [Kovalev 1981]
Lonchaea sylvatica Beling, 1873 [Hackman 1980]
= *L. lucidiventris* Becker, 1895 [Hackman 1956]
Lonchaea tarsata Fallen, 1820 [Hackman 1956, Winqvist and Kahanpää 2007]
Lonchaea ultima Collin, 1953 [Winqvist and Kahanpää 2007]
Lonchaea zetterstedti Becker, 1902 [Frey et al. 1941, Hackman 1956]

Excluded species

Earomyia crystallophila (Becker, 1895) [Winqvist and Kahanpää 2007]
= *E. nigroviolacea* (Frey in Lundström & Frey, 1913)
Lonchaea hirticeps Zetterstedt, 1838 [Hackman 1956, 1980, Winqvist and Kahanpää 2007]

NEOTTIOPHILIDAE Hendel, 1916

Supporting references for *Kahanpää J, Winqvist K (2014) Checklist of the Diptera superfamilies Tephritoidea and Sciomyzoidea of Finland (Insecta). In: Kahanpää J, Salmela J (Eds) Checklist of the Diptera of Finland. ZooKeys @@: @-@. doi: 10.3897/zookeys.??.7143*

ACTENOPTERA Czerny, 1904
Actenoptera hilarella (Zetterstedt, 1847) [Haarto and Winqvist 2014]
NEOTTIOPHILUM Frauenfeld, 1868
Neottiophilum praeustum (Meigen, 1826) [Haarto and Winqvist 2014]

PALLOPTERIDAE Loew, 1862

Supporting references for *Kahanpää J, Winqvist K (2014) Checklist of the Diptera superfamilies Tephritoidea and Sciomyzoidea of Finland (Insecta). In: Kahanpää J, Salmela J (Eds) Checklist of the Diptera of Finland. ZooKeys @@: @-@. doi: 10.3897/zookeys.??.7143*

PALLOPTERA Fallén, 1820
Palloptera anderssoni Rotheray & MacGowan, 1999 [Rotheray and McGowan 1999]
Palloptera formosa Frey, 1930 [Frey 1930, Hackman 1980]
Palloptera marginata (Meigen, 1826) [Haarto et al. 2002, Haarto 2002b]
= *P. costalis* Loew, 1873
Palloptera umbellatarum (Fabricius, 1775)
= *P. arcuata* auct. nec(Fabricius, 1781) [Hackman 1980]
Palloptera ustulata Fallén, 1820 [Hackman 1980]
TEMNOSIRA Enderlein, 1936
Temnosira ambusta (Meigen, 1826) [Hackman 1980]
Temnosira saltuum (Linnaeus, 1758) [Hackman 1980]
TOXONEURA Macquart, 1835
Toxoneura ephippium Zetterstedt, 1860 [Hackman 1980]
Toxoneura laetabilis Loew, 1873 [Hackman 1980]
Toxoneura modesta (Meigen, 1830) [Winqvist and Kahanpää 2007]
= *T. umbellatarum* auct. nec(Fabricius, 1775) [Hackman 1980]
Toxoneura trimacula (Meigen, 1826) [Hackman 1980]
Toxoneura usta (Meigen, 1826) [Hackman 1980]
Toxoneura venusta Loew, 1858 Polevoi [2001a]
= *T. atriventris* (Ringdahl, 1947)

PIOPHILIDAE Macquart, 1835

Supporting references for *Kahanpää J, Winqvist K (2014) Checklist of the Diptera superfamilies Tephritoidea and Sciomyzoidea of Finland (Insecta). In: Kahanpää J, Salmela J (Eds) Checklist of the Diptera of Finland. ZooKeys @@: @-@. doi: 10.3897/zookeys.??.7143*

PIOPHILINAE Macquart, 1835
ALLOPIOPHILA Hendel, 1917
= ***Arctopiophila*** Duda, 1924
= ***Boreopiophila*** Frey, 1930
= ***Parapiophila*** McAlpine, 1977
Allopiophila calceata (Duda, 1924) [Hackman 1980]
Allopiophila flavipes (Zetterstedt, 1847) [Frey et al. 1941]
Allopiophila lonchaeoides (Zetterstedt, 1838) [Frey et al. 1941]
Allopiophila luteata (Haliday, 1833) [Hackman 1980]
Allopiophila pectiniventris (Duda, 1924) [Hackman 1980]
Allopiophila tomentosa Frey, 1930 [Frey 1930, Hackman 1980]
Allopiophila vulgaris (Fallén, 1820) [Hackman 1980]
Allopiophila sp. A [Winqvist and Kahanpää 2007]
AMPHIPOGON Wahlberg, 1845
Amphipogon flavus (Zetterstedt, 1838) [Hackman 1980]
= *A. spectrum* Wahlberg, 1845 [Hackman 1980]
MYCETAULUS Loew, 1845
Mycetaulus bipunctatus (Fallén, 1823) [Hackman 1980]
PIOPHILA Fallén, 1810
Piophila casei (Linnaeus, 1758) [Hackman 1980]
PROCHYLIZA Walker, 1849
= ***Liopiophila*** Duda, 1924
Prochyliza nigrimana (Meigen, 1826) [Hackman 1980]
Prochyliza varipes (Meigen, 1830) [Frey et al. 1941]
= *P. lundbecki* misid.[Hackman 1980]
PSEUDOSEPS Becker, 1902
Pseudoseps signata (Fallén, 1820) [Hackman 1980]
STEARIBIA Lioy, 1864
Stearibia nigriceps (Meigen, 1826)
= *S. foveolata* (Meigen, 1826) [Hackman 1980]
= *S. coerulescens* (Zetterstedt, 1847)

Excluded species

Allopiophila dudai (Frey, 1930) [Frey 1930, Hackman 1980]

PLATYSTOMATIDAE Schiner, 1862

Supporting references for *Kahanpää J, Winqvist K (2014) Checklist of the Diptera superfamilies Tephritoidea and Sciomyzoidea of Finland (Insecta). In: Kahanpää J, Salmela J (Eds) Checklist of the Diptera of Finland. ZooKeys @@: @-@. doi: 10.3897/zookeys.??.7143*

PLATYSTOMA Meigen, 1803
Platystoma seminationis (Fabricius, 1775) [Hackman 1980]
RIVELLIA Robineau-Desvoidy, 1830
Rivellia syngenesiae (Fabricius, 1781) [Hackman 1980]

TEPHRITIDAE Newman, 1834

Supporting references for *Kahanpää J, Winqvist K (2014) Checklist of the Diptera superfamilies Tephritoidea and Sciomyzoidea of Finland (Insecta). In: Kahanpää J, Salmela J (Eds) Checklist of the Diptera of Finland. ZooKeys @@: @-@. doi: 10.3897/zookeys.??.7143*

TRYPETINAE Loew, 1861 *sensu lato*
tribe Adramini Hendel, 1914
EUPHRANTA Loew, 1862
sg. ***Euphranta*** Loew, 1862
Euphranta connexa (Fabricius, 1794) [Hackman 1980]
sg. ***Rhacochlaena*** Loew, 1862
Euphranta toxoneura (Loew, 1846) [Söderman et al. 2007, Winqvist and Kahanpää 2007]
tribe Trypetini Loew, 1861
ACIDIA Robineau-Desvoidy, 1830
Acidia cognata (Wiedemann, 1817) [Hackman 1980]
ANOMOIA Walker, 1835
= ***Phagocarpus*** Rondani, 1870
Anomoia purmunda (Harris, 1776) [Nordman 1963, Hackman 1980]
CHETOSTOMA Rondani, 1856
Chetostoma stackelbergi (Rohdendorf, 1955) [Winqvist and Kahanpää 2007]
CORNUTRYPETA Han, Wang & Kim, 1993
Cornutrypeta spinifrons (Schroeder, 1913) [Ano 1956, Hackman 1980, 1981]
Cornutrypeta superciliata (Frey, 1935) [Frey 1935, Winqvist and Kahanpää 2007]
EULEIA Walker, 1835
= ***Cryptaciura*** Hendel, 1927
Euleia heraclei (Linnaeus, 1758) [Hackman 1980]
Euleia rotundiventris (Fallén, 1814) [Hackman 1980]
MYOLEJA Rondani, 1856
Myoleja lucida (Fallén, 1826) [Hackman 1980]
PHILOPHYLLA Rondani, 1870
Philophylla caesio (Harris, 1776) [Hackman 1980]
RHAGOLETIS Loew, 1862
Rhagoletis alternata (Fallén, 1814) [Hackman 1980]
Rhagoletis cerasi (Linnaeus, 1758) [Hackman 1980]
Rhagoletis meigenii (Loew, 1844) [Flinck and Kahanpää 2013]
TRYPETA Meigen, 1803
Trypeta artemisiae (Fabricius, 1794) [Hackman 1980]
Trypeta immaculata (Macquart, 1835) [Hackman 1980]
= *T. hamifera* Loew, 1846
Trypeta zoe Meigen, 1826 [Hackman 1980]
TEPHRITINAE Newman, 1834
tribe Terellini Hendel, 1927
CHAETORELLIA Hendel, 1927
Chaetorellia jaceae (Robineau-Desvoidy, 1830) [Hackman 1980]
CHAETOSTOMELLA Hendel, 1927
Chaetostomella cylindrica (Robineau-Desvoidy, 1830)
= *C. onotrophes* (Loew, 1848) [Hackman 1980]

ORELLIA Robineau-Desvoidy, 1830
Orellia falcata (Scopoli, 1763) [Hackman 1980]
TERELLIA Robineau-Desvoidy, 1830
sg. ***Cerajocera*** Rondani, 1856
Terellia ceratocera (Hendel, 1913) [Hackman 1980]
Terellia plagiata (Dahlbom, 1850) [Söderman et al. 2007, Winqvist and Kahanpää 2007]
Terellia tussilaginis (Fabricius, 1775) [Hackman 1980]
sg. ***Terellia*** Robineau-Desvoidy, 1830
Terellia colon (Meigen, 1826) [Söderman et al. 2007, Winqvist and Kahanpää 2007]
Terellia ruficauda (Fabricius, 1794) [Hackman 1980]
Terellia serratulae (Linnaeus, 1758) [Hackman 1980]
Terellia winthemi (Meigen, 1826) [Hackman 1980]
tribe Xyphosiini Hendel, 1927
XYPHOSIA Robineau-Desvoidy, 1830
Xyphosia miliaria (Schrank, 1781) [Hackman 1980]
tribe Noeetini Norrbom & Korneyev, 1999
ENSINA Robineau-Desvoidy, 1830
Ensina sonchi (Linnaeus, 1767) [Hackman 1980]
NOEETA Robineau-Desvoidy, 1830
= ***Oplocheta*** Rondani, 1856
Noeeta pupillata (Fallén, 1814) [Hackman 1980]
tribe Myopitini Bezzi, 1910
EURASIMONA Korneyev & White, 1991
Eurasimona stigma (Loew, 1840) [Hackman 1980]
UROPHORA Robineau-Desvoidy, 1830
Urophora aprica (Fallén, 1820) [Hackman 1980]
Urophora cardui (Linnaeus, 1758) [Jansson and Lindeberg 1982]
Urophora cuspidata (Meigen, 1826) [Hackman 1980]
Urophora jaceana (Hering, 1935) [Tiensuu 1963, Hackman 1980]
Urophora solstitialis (Linnaeus, 1758) [Hackman 1980]
= *U. sonderupi* (Hering, 1940)
Urophora stylata (Fabricius, 1775) [Hackman 1980]
tribe Dithrycini Hendel, 1927
DITHRYCA Rondani, 1856
Dithryca guttularis (Meigen, 1826) [Hackman 1980]
tribe Eutretini Munro, 1952
XANTHOMYIA Phillips, 1923
= ***Paracarphotrichia*** Hendel, 1927
Xanthomyia alpestris (Pokorny, 1887)
= *X. pseudoradiata* (Becker, 1900) [Hackman 1980]
tribe Tephritini Newman, 1834
CAMPIGLOSSA Rondani, 1870
Campiglossa absinthii (Fabricius, 1805) [Hackman 1980]
= *C. parvula* (Loew, 1862) [Frey et al. 1941]
Campiglossa argyrocephala (Loew, 1844) [Hackman 1980]
Campiglossa difficilis (Hendel, 1927) [Hackman 1980]
= *C. tessellata* misid.[Frey et al. 1941, Hackman 1980]
Campiglossa grandinata (Rondani, 1870) [Frey et al. 1941]
= *C. borealis* Portschincky, 1875 [Hackman 1980]
Campiglossa guttella (Rondani, 1870) [Winqvist and Kahanpää 2007]

= *C. achyrophori* misid.[Hackman 1980]
= *C. producta* misid.[Söderman et al. 2007]
Campiglossa loewiana (Hendel, 1927) [Hackman 1980]
Campiglossa plantaginis (Haliday, 1833) [Hackman 1980]
Campiglossa punctella (Fallén, 1814) [Hackman 1980]
Campiglossa solidaginis (White, 1986) [Winqvist and Kahanpää 2007]
Campiglossa sp. A
DIOXYNA Frey, 1945
Dioxyna bidentis (Robineau-Desvoidy, 1830) [Hackman 1980]
= *D. sororcula* auct. nec (Wiedemann, 18340)
HERINGINA Aczél, 1940
Heringina guttata (Fallén, 1814) [Hackman 1980]
OXYNA Robineau-Desvoidy, 1830
Oxyna flavipennis (Loew, 1844) [Hackman 1980]
Oxyna nebulosa (Wiedemann, 1817) [Winqvist and Kahanpää 2007]
Oxyna parietina (Linnaeus, 1758) [Hackman 1980]
SPHENELLA Robineau-Desvoidy, 1830
Sphenella marginata (Fallén, 1814) [Hackman 1980]
TEPHRITIS Latreille, 1804
Tephritis angustipennis (Loew, 1844) [Hackman 1980]
Tephritis bardanae (Schrank, 1803) [Hackman 1980]
Tephritis cometa (Loew, 1840) [Hackman 1980]
Tephritis conura (Loew, 1844) [Hackman 1980]
Tephritis dilacerata (Loew, 1846) [Hackman 1980]
Tephritis fallax (Loew, 1844) [Frey et al. 1941]
Tephritis hyoscyami (Linnaeus, 1758) [Hackman 1980]
Tephritis leontodontis (De Geer, 1776) [Hackman 1980]
Tephritis mutabilis Merz, 1992 [Söderman et al. 2007, Winqvist and Kahanpää 2007]
Tephritis neesii (Meigen, 1830)
= *T. nesii* misspelling[Hackman 1980]
Tephritis ruralis (Loew, 1844) [Hackman 1980]
Tephritis sp. cf.*rydeni* Hering, 1956 [Söderman et al. 2007, Winqvist and Kahanpää 2007]
= *T. dioscurea* misid.[Hedström 1964, Hackman 1980]
TRUPANEA Schrank, 1795
Trupanea stellata (Fuessly, 1775) [Hackman 1980]
tribe unplaced in Tephritinae (incertae sedis)
ACINIA Robineau-Desvoidy, 1830
Acinia corniculata (Zetterstedt, 1819) [Hackman 1980]

Excluded species

Bactrocera sp. aff. *dorsalis*
Campiglossa irrorata (Fallén, 1814) [Hackman 1980, Winqvist and Kahanpää 2007]
Ceratitis capitata (Wiedemann, 1824) [Hackman 1980]
Tephritis nigricauda (Loew, 1856) [Hellén 1946, Merz 1994]

ULIDIIDAE Macquart, 1835

Supporting references for *Kahanpää J, Winqvist K (2014) Checklist of the Diptera superfamilies Tephritoidea and Sciomyzoidea of Finland (Insecta). In: Kahanpää J, Salmela J (Eds) Checklist of the Diptera of Finland. ZooKeys @@: @-@. doi: 10.3897/zookeys.??.7143*

OTITINAE Aldrich, 1932
tribe Myennidini Kameneva & Korneyev, 2006
PSEUDOTEPHRITIS Johnson, 1802
Pseudotephritis trypetoptera (Hennig, 1939) [Winqvist and Kahanpää 2007]
= *P. corticalis* auct. nec (Loew, 1873)
tribe Otitini Aldrich, 1932
CEROXYS Macquart, 1835
Ceroxys urticae (Linnaeus, 1758) [Hackman 1980]
HERINA Robineau-Desvoidy, 1830
Herina frondescentiae (Linnaeus, 1758) [Hackman 1980]
Herina paludum (Fallén, 1820) [Hackman 1981]
Herina palustris (Meigen, 1826) [Hackman 1981]
MELIERIA Robineau-Desvoidy, 1830
sg. ***Melieria*** Robineau-Desvoidy, 1830
Melieria crassipennis (Fabricius, 1794) [Hackman 1980]
Melieria omissa (Meigen, 1826) [Winqvist and Kahanpää 2007]
= *M. obscuripes* auct. nec (Loew, 1873) [Hackman 1980]
TETANOPS Fallén, 1820
sg. ***Eurycephalomyia*** Hendel, 1907
Tetanops sintenisi Becker, 1909 [Hackman 1980]
sg. ***Tetanops*** Fallén, 1820
Tetanops myopinus Fallén, 1820 [Hackman 1980]
ULIDIINAE Macquart, 1835
tribe Seiopterini Kameneva & Korneyev, 1994
HOMALOCEPHALA Zetterstedt, 1838
Homalocephala albitarsis Zetterstedt, 1838
= *H. bipunctata* (Loew, 1854) [Hackman 1980]
Homalocephala angustata (Wahlberg, 1839) [Hackman 1980]
Homalocephala apicalis (Wahlberg, 1839) [Hackman 1980]
= *H. biseta* Frey, 1908 [Hackman 1980, Kameneva 2008]
Homalocephala bimaculata (Wahlberg, 1839) [Hackman 1980]
Homalocephala biumbrata (Wahlberg, 1838)
= *H. albitarsis* auct. nec Zetterstedt, 1838 [Hackman 1980]
SEIOPTERA Kirby, 1817
Seioptera vibrans (Linnaeus, 1758) [Hackman 1980]
tribe Ulidiini Macquart, 1835
PHYSIPHORA Fallén, 1810
Physiphora alceae (Preyssler, 1791)
= *P. demandata* (Fabricius, 1798) [Hackman 1980]

CHAMAEMYIIDAE Hendel, 1910

Supporting references for *Kahanpää J (2014) Checklist of the fly families Chamaemyiidae and Lauxaniidae of Finland (Insecta, Diptera). In: Kahanpää J, Salmela J (Eds) Checklist of the Diptera of Finland. ZooKeys @@: @-@. doi: 10.3897/zookeys.??.7506*

CHAMAEMYIINAE Hendel, 1910
tribe Chamaemyiini Hendel, 1910
ACROMETOPIA Schiner, 1862
Acrometopia wahlbergi (Zetterstedt, 1846) [Hackman 1980]
CHAMAEMYIA Meigen, 1803
Chamaemyia aestiva Tanasijtshuk, 1970
Chamaemyia aridella (Fallén, 1823) [Hackman 1980]
Chamaemyia elegans (Panzer, 1809) [Hackman 1980]
Chamaemyia emiliae Tanasijtshuk, 1970
Chamaemyia flavipalpis (Haliday, 1838)
Chamaemyia geniculata (Zetterstedt, 1838) [Hackman 1980]
? *Chamaemyia herbarum* (Robineau-Desvoidy, 1830) sensu Coe, 1943 [Hackman 1980]
Chamaemyia juncorum (Fallén, 1823) [Hackman 1980]
Chamaemyia paludosa Collin, 1966
Chamaemyia polystigma (Meigen, 1830) [Krogerus 1960]
Chamaemyia sylvatica Collin, 1966
PAROCHTHIPHILA Czerny, 1904
sg. ***Euestelia*** Enderlein, 1927
Parochthiphila coronata (Loew, 1858) [Hackman 1980, Fre 1935]
sg. ***Parochthiphila*** Czerny, 1904
Parochthiphila spectabilis (Loew, 1858) [Hackman 1980, Fre 1935]
tribe Leucopini Hendel, 1928
ANCHIOLEUCOPIS Tanasijtshuk 1997
Anchioleucopis geniculata (Zetterstedt, 1855) [Frey et al. 1941]
LEUCOPIS Meigen, 1830
Leucopis annulipes Zetterstedt, 1848 [McAlpine 1967, Hackman 1980]
Leucopis argentata Heeger, 1848
= *L. conciliata* McAlpine & Tanasijtshuk, 1972
= *L. argenticollis* of authors [Hackman 1980]
Leucopis atritarsis Tanasijtshuk, 1958
Leucopis glyphinivora Tanasijtshuk, 1958
Leucopis griseola (Fallén, 1823) [Hackman 1980]
Leucopis sorbi Tanasijtshuk, 1986
Leucopis sp. cf. *szepligetii* Aczél, 1937
NEOLEUCOPIS Malloch, 1921
Neoleucopis atratula (Ratzeburg, 1844) [Frey et al. 1941, McAlpine 1971]
Neoleucopis freyi (McAlpine, 1971) [McAlpine 1971, Hackman 1980]
Neoleucopis obscura (Haliday, 1833) [McAlpine 1971]
Neoleucopis orbiseta (McAlpine, 1971) [McAlpine 1971, Hackman 1980]
LEUCOPOMYIA Malloch, 1921
Leucopomyia silesiaca (Egger, 1862) [Tiensuu 1951]
= *L. alticeps* misid.[Frey et al. 1941]
LIPOLEUCOPIS de Meijere, 1928

Lipoleucopis praecox de Meijere, 1928 [Hackman 1980]

Excluded species

Leucopis impunctata von Roser, 1840 [Hackman 1980]
Leucopis puncticornis Meigen, 1830 [Hackman 1980]

LAUXANIIDAE Macquart, 1835

Supporting references for *Kahanpää J (2014) Checklist of the fly families Chamaemyiidae and Lauxaniidae of Finland (Insecta, Diptera). In: Kahanpää J, Salmela J (Eds) Checklist of the Diptera of Finland. ZooKeys @@: @-@. doi: 10.3897/zookeys.??.7506*

HOMONEURINAE Stuckenberg, 1971
HOMONEURA van der Wulp, 1891
Homoneura biumbrata (Loew, 1873) [Kahanpää 2013c]
 = *H. tesquae* misid.[Hackman 1980]
Homoneura lamellata (Becker, 1895) [Hackman 1980]
Homoneura mediospinosa Merz, 2003 [Kahanpää 2013c]
 = *H. interstincta* auct. nec (Fallén, 1820) [Hackman 1980]
Homoneura tenera (Loew, 1846) [Hackman 1980]
LAUXANIINAE Macquart, 1835
AULOGASTROMYIA Hendel, 1925
Aulogastromyia anisodactyla (Loew, 1845) [Hackman 1980]
CALLIOPUM Strand, 1928
Calliopum aeneum (Fallén, 1820) [Hackman 1980]
Calliopum elisae (Meigen, 1826) [Hackman 1980]
 = *C. nitens* auct. nec (Loew, 1858)
CNEMACANTHA Macquart, 1835
Cnemacantha muscaria (Fallén, 1823) [Hackman 1980]
LAUXANIA Latreille, 1804
 sg. ***Czernushka*** Shatalkin, 2000
Lauxania albomaculata Strobl, 1909 [Kahanpää 2013c] added7.8.2013
 sg. ***Lauxania*** Latreille, 1804
Lauxania cylindricornis (Fabricius, 1794) [Hackman 1980]
MEIOSIMYZA Hendel, 1925
 = ***Lycia*** Robineau-Desvoidy, 1830 preocc.
 = ***Lyciella*** Collin, 1948
Meiosimyza affinis (Zetterstedt, 1847) [Hackman 1980]
Meiosimyza decempunctata (Fallén, 1820) [Hackman 1980]
Meiosimyza decipiens (Loew, 1847) [Hackman 1980]
Meiosimyza illota (Loew, 1847) [Hackman 1980]
Meiosimyza laeta (Zetterstedt, 1838) [Hackman 1980]
Meiosimyza platycephala (Loew, 1847) [Hackman 1980]
Meiosimyza rorida (Fallén, 1820) [Hackman 1980]
Meiosimyza subfasciata (Zetterstedt, 1838) [Hackman 1980]
MINETTIA Robineau-Desvoidy, 1830
 sg. ***Frendelia*** Collin, 1948
Minettia longipennis (Fabricius, 1794) [Hackman 1980]
 sg. ***Minettia*** Robineau-Desvoidy, 1830
Minettia desmometopa (de Meijere, 1907) [Hackman 1980]
Minettia lupulina (Fabricius, 1787) [Hackman 1980]
 sg. ***Plesiominettia*** Shatalkin, 2000
Minettia filia (Becker, 1895) [Kahanpää 2013c]
Minettia helvola (Becker, 1895) [Kahanpää 2013c]
Minettia loewi (Schiner, 1864) [Hackman 1980]

sg. ***unplaced in Minettia***
Minettia styriaca (Strobl, 1892) [Hackman 1980]
PACHYCERINA Macquart, 1835
Pachycerina pulchra (Loew, 1850) [Kahanpää 2013c]
Pachycerina seticornis (Fallén, 1820) [Hackman 1980]
PEPLOMYZA Haliday, 1837
Peplomyza discoidea (Meigen, 1830) [Hackman 1980]
POECILOLYCIA Shewell, 1986
Poecilolycia vittata (Walker, 1849)
= *P. quadrivittata* (Loew, 1861) [Hackman 1980]
PSEUDOLYCIELLA Shatalkin, 2000
Pseudolyciella pallidiventris (Fallén, 1820) [Hackman 1980]
Pseudolyciella stylata (Papp, 1978) [Kahanpää 2013c]
SAPROMYZA Fallén, 1810
sg. ***Nannomyza*** Frey, 1941
Sapromyza basalis Zetterstedt, 1847 [Hackman 1980]
sg. ***Sapromyza*** Fallén, 1810
Sapromyza albiceps (Fallén, 1820) [Kahanpää 2013c]
Sapromyza amabilis Frey, 1930 [Frey 1930, Hackman 1980]
Sapromyza apicalis Loew, 1847 [Kahanpää 2013c]
= *S. obsoleta* misid.[Hackman 1980]
Sapromyza opaca Becker, 1895 [Hedström 1964, Hackman 1980]
= *S. imitatrix* Czerny, 1932 [Hackman 1980, Papp 1984]
= *S. leningradensis* Czerny, 1932 misid.[Hackman 1980]
Sapromyza schnabli Papp, 1987 [Kahanpää 2013c]
Sapromyza setiventris Zetterstedt, 1847 [Hackman 1980]
Sapromyza sexpunctata Meigen, 1826 [Hackman 1980]
= *S. atechna* Becker, 1895 [Hackman 1980]
= *S. pellucida* Becker, 1895 [Hackman 1980]
Sapromyza simplicior Hendel, 1908 [Kahanpää 2013c]
Sapromyza zetterstedti Hendel, 1908 [Hackman 1980]
sg. ***Schumannimyia*** Papp, 1978
Sapromyza hyalinata (Meigen, 1826) [Hackman 1980]
SAPROMYZOSOMA Lioy, 1864
Sapromyzosoma quadripunctata (Linnaeus, 1767) [Hackman 1980]
TRICHOLAUXANIA Hendel, 1925
Tricholauxania praeusta (Fallén, 1820) [Hackman 1980]
TRIGONOMETOPUS Macquart, 1835
Trigonometopus frontalis (Meigen, 1830) [Kahanpää 2013c]

Excluded species

Homoneura dilecta (Rondani, 1868)
Sapromyza thoracica Becker, 1895 [Hackman 1980]
Neoparoecus signatipes (Loew, 1856) [Hackman 1980]

COELOPIDAE Hendel, 1910

Supporting references for *Kahanpää J, Winqvist K (2014) Checklist of the Diptera superfamilies Tephritoidea and Sciomyzoidea of Finland (Insecta). In: Kahanpää J, Salmela J (Eds) Checklist of the Diptera of Finland. ZooKeys @@: @-@. doi: 10.3897/zookeys.??.7143*

COELOPA Meigen, 1830
  sg. ***Fucomyia*** Haliday, 1838
Coelopa frigida (Fabricius, 1805) [Hackman 1980]

DRYOMYZIDAE Schiner, 1862

Supporting references for *Kahanpää J, Winqvist K (2014) Checklist of the Diptera superfamilies Tephritoidea and Sciomyzoidea of Finland (Insecta). In: Kahanpää J, Salmela J (Eds) Checklist of the Diptera of Finland. ZooKeys @@: @-@. doi: 10.3897/zookeys.??.7143*

DRYOMYZA Fallén, 1820
= ***Neuroctena*** Rondani, 1868
Dryomyza anilis Fallén, 1820 [Hackman 1980]
DRYOPE Robineau-Desvoidy, 1830
= ***Dryomyza*** of authors
Dryope decrepita (Zetterstedt, 1838) [Winqvist and Kahanpää 2007]
Dryope flaveola (Fabricius, 1794) [Hackman 1980]
PARADRYOMYZA Ozerov, 1987
Paradryomyza spinigera Ozerov, 1987 [Haarto and Winqvist 2014]
PSEUDONEUROCTENA Ozerov, 1987
Pseudoneuroctena senilis (Zetterstedt, 1838) [Hackman 1980]

HETEROCHEILIDAE McAlpine, 1991

Supporting references for *Kahanpää J, Winqvist K (2014) Checklist of the Diptera superfamilies Tephritoidea and Sciomyzoidea of Finland (Insecta). In: Kahanpää J, Salmela J (Eds) Checklist of the Diptera of Finland. ZooKeys @@: @-@. doi: 10.3897/zookeys.??.7143*

HETEROCHEILA Rondani, 1857
Heterocheila buccata (Fallén, 1820) [Hackman 1980]

PHAEOMYIIDAE Verbeke, 1950

Supporting references for *Kahanpää J, Winqvist K (2014) Checklist of the Diptera superfamilies Tephritoidea and Sciomyzoidea of Finland (Insecta). In: Kahanpää J, Salmela J (Eds) Checklist of the Diptera of Finland. ZooKeys @@: @-@. doi: 10.3897/zookeys.??.7143*

PELIDNOPTERA Rondani, 1856
Pelidnoptera fuscipennis (Meigen, 1830) [Hackman 1980]
= *P. fumipennis* (Zetterstedt, 1846)
Pelidnoptera nigripennis (Fabricius, 1794) [Hackman 1980]

SCIOMYZIDAE Fallén, 1820

Supporting references for *Kahanpää J, Winqvist K (2014) Checklist of the Diptera superfamilies Tephritoidea and Sciomyzoidea of Finland (Insecta). In: Kahanpää J, Salmela J (Eds) Checklist of the Diptera of Finland. ZooKeys @@: @-@. doi: 10.3897/zookeys.??.7143*

SCIOMYZINAE Fallén, 1820
tribe Sciomyzini Fallén, 1820
COLOBAEA Zetterstedt, 1837
Colobaea bifasciella (Fallén, 1820) [Hackman 1980]
Colobaea distincta (Meigen, 1830) [Hackman 1980]
Colobaea nigroaristata Rozkošný, 1984 [Rozkošný 1984]
Colobaea pectoralis (Zetterstedt, 1847) [Hackman 1980]
Colobaea punctata Lundbeck, 1923 [Hackman 1980]
DITAENIELLA Sack, 1939
Ditaeniella grisescens (Meigen, 1830) [Hackman 1980]
PHERBELLIA Robineau-Desvoidy, 1830
Pherbellia albocostata (Fallén, 1820) [Hackman 1980]
Pherbellia alpina (Frey, 1930) [Frey 1930, Hackman 1980]
Pherbellia argyra Verbeke, 1967 [Hackman 1980]
Pherbellia brunnipes (Meigen, 1838) [Hackman 1980]
Pherbellia cinerella (Fallén, 1820) [Hackman 1980]
Pherbellia dubia (Fallén, 1820) [Hackman 1980]
Pherbellia goberti (Pandellé, 1902)
= *P. stylifera* Rozkošný, 1982 [Rozkošný 1982]
Pherbellia griseicollis (Becker, 1900) [Hackman 1980]
= *P. lapponica* (Ringdahl, 1948) [Hackman 1980]
Pherbellia griseola (Fallén, 1820) [Hackman 1980]
Pherbellia hackmani Rozkošný, 1982 [Rozkošný 1982]
Pherbellia nana (Fallén, 1820) [Hackman 1980]
Pherbellia obscura (Ringdahl, 1948) [Rozkošný 1981]
Pherbellia obtusa (Fallén, 1820) [Hackman 1980, Rozkošný 1981]
Pherbellia pallidiventris (Fallén, 1820) [Hackman 1980]
Pherbellia rozkosnyi Verbeke, 1967 [Rozkošný 1981]
= *P. scutellaris* misid.[Hackman 1980, Rozkošný 1981]
Pherbellia schoenherri (Fallén, 1826) [Hackman 1980]
Pherbellia sordida (Hendel, 1902) [Hackman 1980]
Pherbellia stackelbergi Elberg, 1965 [Rozkošný 1981]
Pherbellia ventralis (Fallén, 1820) [Hackman 1980]
PTEROMICRA Lioy, 1864
Pteromicra angustipennis (Staeger, 1845) [Hackman 1980]
Pteromicra glabricula (Fallén, 1820) [Hackman 1980]
Pteromicra leucopeza (Meigen, 1838) [Hackman 1980]
Pteromicra oldenbergi (Hendel, 1902) [Kahanpää 2013d]
Pteromicra pectorosa (Hendel, 1902) [Winqvist and Kahanpää 2007]
SCIOMYZA Fallén, 1820
Sciomyza dryomyzina Zetterstedt, 1846 [Hackman 1980]
Sciomyza sebezhica Przhiboro, 2001 [Winqvist and Kahanpää 2007]
Sciomyza simplex Fallén, 1820 [Hackman 1980]

TETANURA Fallén, 1820
Tetanura pallidiventris Fallén, 1820 [Hackman 1980]
tribe Tetanocerini Newman, 1834
ANTICHETA Haliday, 1839
Anticheta analis (Meigen, 1830) [Hackman 1980]
Anticheta atriseta (Loew, 1849) [Hackman 1980]
Anticheta brevipennis (Zetterstedt, 1846) [Hackman 1980]
Anticheta nigra Karl, 1921 [Hackman 1980]
= *A. nigroaenea* Frey, 1935 [Frey 1935]
COREMACERA Rondani, 1856
Coremacera marginata (Fabricius, 1775)
= *C. tristis* (Harris, 1780) preocc.[Hackman 1980]
DICHETOPHORA Rondani, 1868
Dichetophora finlandica Verbeke, 1964 [Verbeke 1964, Hackman 1980]
DICTYA Meigen, 1803
Dictya umbrarum (Linnaeus, 1758) [Hackman 1980]
ECTINOCERA Zetterstedt, 1838
Ectinocera borealis Zetterstedt, 1838 [Hackman 1980]
ELGIVA Meigen, 1838
Elgiva cucularia (Linnaeus, 1767) [Hackman 1980]
Elgiva divisa (Loew, 1845) [Hackman 1980]
Elgiva solicita (Harris, 1780)
= *E. sundewalli* Kloet & Hincks, 1945 [Hackman 1980]
EUTHYCERA Latreille, 1829
Euthycera chaerophylli (Fabricius, 1798) [Hackman 1980]
Euthycera fumigata (Scopoli, 1763) [Flinck and Kahanpää 2013]
HYDROMYA Robineau-Desvoidy, 1830
Hydromya dorsalis (Fabricius, 1775) [Hackman 1980]
ILIONE Haliday in Curtis, 1837
sg. ***Ilione*** Haliday in Curtis, 1837
Ilione lineata (Fallén, 1820) [Hackman 1980]
sg. ***Knutsonia*** Verbeke, 1964
Ilione albiseta (Scopoli, 1763) [Hackman 1980]
LIMNIA Robineau-Desvoidy, 1830
Limnia paludicola Elberg, 1965 [Rozkošný 1981]
Limnia unguicornis (Scopoli, 1763) [Hackman 1980]
PHERBINA Robineau-Desvoidy, 1830
Pherbina coryleti (Scopoli, 1763) [Hackman 1980]
PSACADINA Enderlein, 1939
Psacadina zernyi (Mayer, 1953) [Hac 1963, Hackman 1980]
= *P. punctata* misid.[Hackman 1980, Rozkošný 1981]
RENOCERA Hendel, 1900
Renocera pallida (Fallén, 1820) [Hackman 1980]
Renocera striata (Meigen, 1830) [Hackman 1980]
Renocera stroblii Hendel, 1900 [Rozkošný 1984]
= *R. fuscinervis* auct. nec (Zetterstedt, 1838) [Hackman 1980]
SEPEDON Latreille, 1804
sg. ***Sepedon*** Latreille, 1804
Sepedon sphegea (Fabricius, 1775) [Hackman 1980]
Sepedon spinipes (Scopoli, 1763) [Hackman 1980]

TETANOCERA Duméril, 1800
sg. ***Chaetotetanocera*** Mayer, 1953
Tetanocera robusta Loew, 1847 [Hackman 1980]
sg. ***Tetanocera*** Duméril, 1800
? *Tetanocera amurensis* Hendel, 1909
Tetanocera arrogans Meigen, 1830 [Hackman 1980]
Tetanocera elata (Fabricius, 1781) [Hackman 1980]
Tetanocera ferruginea Fallén, 1820 [Hackman 1980]
= *T. brunnipennis* Frey, 1924 [Hackman 1980]
Tetanocera freyi Stackelberg, 1963 [Rozkošný 1981]
Tetanocera fuscinervis (Zetterstedt, 1838)
= *T. unicolor* Loew, 1847 [Hackman 1980]
Tetanocera hyalipennis von Roser, 1840 [Hackman 1980]
Tetanocera kerteszi Hendel, 1901
= *T. griseicollis* Frey, 1924
= *T. ornatifrons* Frey, 1924 [Hackman 1980]
Tetanocera lapponica Frey, 1924 [Hackman 1980]
Tetanocera latifibula Frey, 1924 [Hackman 1980]
Tetanocera montana Day, 1881 [Hackman 1980]
= *T. borealis* Frey, 1924
Tetanocera phyllophora Melander, 1920 [Hackman 1980]
= *T. nigricosta* misid.[Hac 1963, Hackman 1980, Rozkošný 1981]
Tetanocera silvatica Meigen, 1830 [Hackman 1980]
TRYPETOPTERA Hendel, 1900
Trypetoptera punctulata (Scopoli, 1763) [Hackman 1980]

Excluded species

Pherbellia dorsata (Zetterstedt, 1846) [Hackman 1980, Rozkošný 1981]

SEPSIDAE Walker, 1833

Supporting references for *Kahanpää J, Winqvist K (2014) Checklist of the Diptera superfamilies Tephritoidea and Sciomyzoidea of Finland (Insecta). In: Kahanpää J, Salmela J (Eds) Checklist of the Diptera of Finland. ZooKeys @@: @-@. doi: 10.3897/zookeys.??.7143*

ORYGMATINAE Frey, 1921
ORTALISCHEMA Frey, 1925
Ortalischema albitarse (Zetterstedt, 1847) [Pont 1990]
SEPSINAE Walker, 1833
THEMIRA Robineau-Desvoidy, 1830
Themira annulipes (Meigen, 1826) [Hackman 1980]
Themira arctica (Becker, 1915) [Hackman 1980]
Themira biloba Andersson, 1975 [Pont 1990]
Themira germanica Duda, 1926 [Pont 1990]
Themira gracilis (Zetterstedt, 1847) [Hackman 1980]
Themira leachi (Meigen, 1826) [Hackman 1980]
Themira lucida (Staeger, 1844) [Pont 1990]
Themira malformans Melander & Spuler, 1917 [Pont 1990]
Themira minor (Haliday, 1833) [Hackman 1980]
Themira nigricornis (Meigen, 1826) [Hackman 1980]
Themira paludosa Elberg, 1963 [Pont 1990]
Themira pusilla (Zetterstedt, 1847) [Hackman 1980]
Themira putris (Linnaeus, 1758) [Hackman 1980]
Themira superba (Haliday, 1833) [Hackman 1980]
SALTELLA Robineau-Desvoidy, 1830
Saltella sphondylii (Schrank, 1803) [Hackman 1980]
NEMOPODA Robineau-Desvoidy, 1830
Nemopoda nitidula (Fallén, 1820) [Hackman 1980]
= *N. cylindrica* (Fabricius, 1794) preocc.
Nemopoda pectinulata Loew, 1873 [Hackman 1980]
Nemopoda speiseri (Duda, 1926) [Hedström 1964, Hackman 1980]
MEROPLIUS Rondani, 1874
Meroplius fukuharai (Iwasa, 1984) [Haarto and Winqvist 2014]
Meroplius minutus (Wiedemann, 1830)
= *M. stercorarius* (Robineau-Desvoidy, 1830) [Hackman 1980]
SEPSIS Fallén, 1810
Sepsis biflexuosa Strobl, 1893 [Pont 1990]
Sepsis cynipsea (Linnaeus, 1758) [Hackman 1980]
Sepsis duplicata Haliday, 1838 [Hackman 1980]
= *S. pilipes* van der Wulp, 1871
Sepsis flavimana Meigen, 1826 [Hackman 1980]
= *S. borealis* Frey, 1825
Sepsis fulgens Meigen, 1826 [Hackman 1980]
= *S. communis* Frey, 1925
Sepsis luteipes Melander & Spuler, 1917 [Pont and Meier 2002]
= *S. lamellifera* Frey, 1925
Sepsis nigripes Meigen, 1826 [Hackman 1980]
Sepsis orthocnemis Frey, 1908 [Hackman 1980]

Sepsis punctum (Fabricius, 1794) [Hackman 1980]
= *S. luteipes* misid.[Hackman 1980, Pont 1990]
Sepsis thoracica (Robineau-Desvoidy, 1830) [Pont 1990]
Sepsis violacea Meigen, 1826

Excluded species

Orygma luctuosum Meigen, 1830 [Hackman 1980, Pont and Meier 2002]

AGROMYZIDAE Fallén, 1823

Supporting references for *Kahanpää J (2014) Checklist of the leaf-mining flies (Diptera, Agromyzidae) of Finland. In: Kahanpää J, Salmela J (Eds) Checklist of the Diptera of Finland. ZooKeys @@: @-@. doi: 10.3897/zookeys.??.7586*

AGROMYZINAE Fallén, 1823
AGROMYZA Fallén, 1810
Agromyza abiens Zetterstedt, 1848 [Spencer 1976, Hackman 1980]
Agromyza albipennis Meigen, 1830 [Hackman 1980]
Agromyza albitarsis Meigen, 1830 [Hackman 1980]
Agromyza alnibetulae Hendel, 1931 [Hackman 1980]
Agromyza alnivora Spencer, 1969 [Spencer 1976, Hackman 1980]
Agromyza alunulata (Hendel, 1931) [Hackman 1980]
Agromyza ambigua Fallén, 1823 [Hackman 1980]
Agromyza anthracina Meigen, 1830 [Hackman 1980]
Agromyza bicaudata Hendel, 1931 [Spencer 1976]
Agromyza cinerascens Macquart, 1835 [Hackman 1980]
Agromyza demeijerei Hendel, 1920 [Vikberg 1986]
Agromyza erythrocephala Hendel, 1920 [Karppinen 1964b, Hackman 1980]
Agromyza flaviceps Fallén, 1823 [Hackman 1980]
Agromyza graminicola Hendel, 1931 [Hackman 1980]
Agromyza idaeiana Hardy, 1853
= *A. potentillae* (Kaltenbach, 1864) [Hackman 1980]
= *A. stackelbergi* (Frey, 1946)
Agromyza igniceps Hendel, 1920 [Hackman 1980]
= *A. lathyri* misid.[Kaisila 1960, Karppinen 1964b]
Agromyza lapponica Hendel, 1931 [Hackman 1980]
Agromyza lucida Hendel, 1920 [Hackman 1980]
Agromyza luteitarsis (Rondani, 1875) [Spencer 1976, Hackman 1980]
= *A. intermittens* misid.[Spencer 1976]
Agromyza marionae Griffiths, 1963
= *A. alandensis* Spencer, 1976 [Spencer 1976, Hackman 1980]
Agromyza mobilis Meigen, 1830 [Hackman 1980]
Agromyza nana Meigen, 1830 [Hackman 1980]
Agromyza nigrella (Rondani, 1875) [Spencer 1976, Hackman 1980]
Agromyza nigrescens Hendel, 1920 [Hackman 1980]
Agromyza nigripes Meigen, 1830 [Hackman 1980]
Agromyza nigrociliata Hendel, 1931 [Kaisila 1960]
Agromyza orobi Hendel, 1920 [Hackman 1980]
Agromyza phragmitidis Hendel, 1922 [Kaisila 1960, Hackman 1980]
Agromyza pseudoreptans Nowakowski, 1967 [Thuneberg 1975, Hackman 1980]
= *A. urticae* Nowakowski, 1964 preocc.[Karppinen 1964b]
Agromyza quadriseta Zlobin, 2001 [Zlobin 2001]
Agromyza reptans Fallén, 1823 [Winqvist 2007]
Agromyza rufipes (Meigen, 1830) [Haarto and Winqvist 2014]
Agromyza salicina Hendel, 1922 [Hackman 1980]
Agromyza sulfuriceps Strobl, 1898 [Hackman 1980]
Agromyza vicifoliae Hering, 1932 [Hackman 1980]
HEXOMYZA Enderlein, 1936

Hexomyza schineri (Giraud, 1861) [Vikberg 1978]
Hexomyza simplicoides Hendel, 1920 [Hackman 1980]
MELANAGROMYZA Hendel, 1920
Melanagromyza aenea (Meigen, 1830) [Spencer 1976, Hackman 1980]
Melanagromyza aeneoventris (Fallén, 1823) [Hackman 1980]
Melanagromyza angeliciphaga Spencer, 1969 [Winqvist 2007]
Melanagromyza chaerophylli Spencer, 1969 [Haarto and Winqvist 2014]
Melanagromyza lappae (Loew, 1850) [Hackman 1980]
Melanagromyza nigrissima Spencer, 1976 [Spencer 1976, Hackman 1980]
Melanagromyza oligophaga Spender, 1990 [Vikberg and Malinen 2012]
Melanagromyza pubescens Hendel, 1923 [Hackman 1980]
Melanagromyza submetallescens Spencer, 1966 [Spencer 1976, Hackman 1980]
OPHIOMYIA Braschnikov, 1897
Ophiomyia beckeri (Hendel, 1923) [Hackman 1980]
= *O. goniaea* (Hendel, 1931)
Ophiomyia cunctata (Hendel, 1920) [Hackman 1980]
Ophiomyia curvipalpis (Zetterstedt, 1848)
Ophiomyia fennoniensis Spencer, 1976 [Spencer 1976, Hackman 1980]
? = *O. eucodonus* Hering, 1960
= *O. melandryi* misid.[Frey 1946, Spencer 1976]
Ophiomyia heringi Starý, 1930 [Spencer 1976, Hackman 1980]
= *O. penicillata* misid.[Frey 1937, Spencer 1976]
Ophiomyia labiatarum Hering, 1937 [Spencer 1976, Hackman 1980]
Ophiomyia longilingua (Hendel, 1920) [Hackman 1980]
= *O. rostrata* misid.[Kaisila 1960, Spencer 1976]
Ophiomyia maura (Meigen, 1838) [Hackman 1980]
Ophiomyia nasuta (Melander, 1913) [Hackman 1980]
Ophiomyia orbiculata (Hendel, 1931) [Hackman 1980]
= *O. nostradamus* (Hering, 1933) [Kaisila 1960]
= *O. paracelsus* (Hering, 1933) [Kaisila 1960]
Ophiomyia pinguis (Fallén, 1820) [Hackman 1980]
Ophiomyia pulicaria (Meigen, 1830) [Hackman 1980]
Ophiomyia ranunculicaulis Hering, 1949 [Winqvist 2007]
Ophiomyia vitiosa Spencer, 1964
? = *O. alliariae* Hering, 1954
PHYTOMYZINAE Fallén, 1823
AMAUROMYZA Hendel, 1931
sg. ***Amauromyza*** Hendel, 1931
Amauromyza morionella (Zetterstedt, 1848) [Hackman 1980]
sg. ***Cephalomyza*** Hendel, 1931
= ***Trilobomyza*** Hendel, 1931
Amauromyza chenopodivora Spencer, 1971 [Spencer 1976, Hackman 1980]
= *A. abnormalis* auct. nec (Malloch, 1913) [Frey 1937, 1946, Spencer 1976]
Amauromyza flavifrons (Meigen, 1830) [Hackman 1980]
Amauromyza gyrans (Fallén, 1823) [Hackman 1980]
Amauromyza karli (Hendel, 1927) [Haarto and Winqvist 2014]
= *Dizygomyza facialis* Frey, 1941 nom. nudum[Frey et al. 1941]
Amauromyza labiatarum (Hendel, 1920) [Hackman 1980]
Amauromyza luteiceps (Hendel, 1920) [Hackman 1980]
Amauromyza monfalconensis (Strobl, 1909) [Kaisila 1960, Kahanpää 2013d]

AULAGROMYZA Enderlein, 1936
= ***Paraphytomyza*** Enderlein, 1936
Aulagromyza buhri (de Meijere, 1938) [Spencer 1976, Hackman 1980]
Aulagromyza fulvicornis (Hendel, 1935) [Hackman 1980]
Aulagromyza hendeliana (Hering, 1926) [Hackman 1980]
Aulagromyza heringii (Hengel, 1920) [Kahanpää 2013d]
Aulagromyza lucens (de Meijere, 1924) [Karppinen 1964b, Hackman 1980]
Aulagromyza luteoscutellata (de Meijere, 1924) [Spencer 1976, Hackman 1980]
= *A. xylostei* auct. nec (Robineau-Desvoidy, 1851) [Spencer 1976]
Aulagromyza similis (Brischke, 1880) [Hackman 1980]
Aulagromyza tremulae (Hering 1957) [Kahanpää 2013d]
Aulagromyza tridentata (Loew, 1858) [Hackman 1980]
Aulagromyza trivittata (Loew, 1873) [Haarto and Winqvist 2014] [Spencer 1976]
CALYCOMYZA Hendel, 1931
Calycomyza artemisiae (Kaltenbach, 1856) [Hackman 1980]
Calycomyza subapproximata (Sasakawa, 1955) [Zlobin and Pakalniškis 1993]
CERODONTHA Rondani, 1861
sg. ***Butomomyza*** Nowakowski, 1967
Cerodontha caricivora (Groschke, 1954) [Nowakowski 1973, Hackman 1980]
Cerodontha eucaricis Nowakowski, 1967 [Haarto and Winqvist 2014]
Cerodontha rohdendorfi Nowakowski, 1967 [Nowakowski 1973, Hackman 1980]
Cerodontha scirpi (Karl, 1926) [Spencer 1976, Hackman 1980]
= *C. scutellaris* misid.[Spencer 1976]
= *C. semiposticata* misid.[Spencer 1976]
Cerodontha staryi (Starý, 1930) [Kahanpää 2013d]
sg. ***Cerodontha*** Rondani, 1861
Cerodontha affinis (Fallén, 1823) [Hackman 1980]
Cerodontha denticornis (Panzer, 1806) [Hackman 1980]
Cerodontha fulvipes (Meigen, 1830) [Hackman 1980]
Cerodontha hennigi Nowakowski, 1967 [Hackman 1980]
= *C. lateralis* (Zetterstedt, 1848) preocc.
Cerodontha stackelbergi Nowakowski, 1972 [Nowakowski 1973, Hackman 1980]
sg. ***Dizygomyza*** Hendel, 1920
Cerodontha bimaculata (Meigen, 1830) [Hackman 1980]
Cerodontha fasciata (Strobl, 1880) [Hackman 1980]
Cerodontha ireos (Robineau-Desvoidy, 1851) [Hackman 1980]
Cerodontha luctuosa (Meigen, 1830) [Hackman 1980]
Cerodontha morosa (Meigen, 1830) [Hackman 1980]
sg. ***Icteromyza*** Hendel, 1931
Cerodontha bohemani (Rydén, 1951) [Kahanpää 2013d]
Cerodontha capitata (Zetterstedt, 1848) [Hackman 1980]
Cerodontha churchillensis Spencer, 1969 [Haarto and Winqvist 2014]
Cerodontha geniculata (Fallén, 1823) [Hackman 1980]
Cerodontha lineella (Zetterstedt, 1838) [Hackman 1980]
sg. ***Phytagromyza*** Hendel, 1920
Cerodontha flavocingulata (Strobl, 1909) [Hackman 1980]
= *C. storai* (Frey, 1946)
sg. ***Poemyza*** Hendel, 1931
Cerodontha atra (Meigen, 1830) [Hackman 1980]
Cerodontha calamagrostidis Nowakowski, 1967

= *C. spenceri* Nowakowski, 1967 [Spencer 1976]
= *C. tschirnhausi* Nowakowski, 1973
Cerodontha calosoma (Hendel, 1931) [Hackman 1980]
Cerodontha imbuta (Meigen, 1838)
= *C. deschampsiae* (Spencer, 1957) [Karppinen 1964b, Hackman 1980]
Cerodontha incisa (Meigen, 1830) [Hackman 1980]
Cerodontha lateralis (Macquart, 1835) [Hackman 1980]
Cerodontha muscina (Meigen, 1830) [Hackman 1980]
Cerodontha phragmitidis Nowakowski, 1967 [Hackman 1980]
Cerodontha pygmaea (Meigen, 1830) [Hackman 1980]
Cerodontha pygmella (Hendel, 1931)
= *C. lapplandica* (Rydén, 1956) [Spencer 1976]
Cerodontha spencerae Zlobin, 1993 [Zlobin 1995]
= *C. inconspicua* auct. nec (Malloch, 1914) [Nowakowski 1973]
Cerodontha thunebergi Nowakowski, 1967 [Nowakowski 1967, Hackman 1980]
sg. ***Xenophytomyza*** Frey, 1946
Cerodontha atronitens (Hendel, 1920) [Hackman 1980]
Cerodontha biseta (Hendel, 1920) [Hackman 1980]
= *C. crassinervis* (Frey, 1946)
Cerodontha venturii Nowakowski, 1967 [Nowakowski 1973, Hackman 1980]
CHROMATOMYIA Hardy, 1849
Chromatomyia asteris (Hendel, 1934) [Winqvist 2007]
Chromatomyia ciliata (Hendel, 1935) [Hackman 1980]
Chromatomyia farfarella (Hendel, 1935) [Hackman 1980]
Chromatomyia fuscula (Zetterstedt, 1838) [Hackman 1980]
Chromatomyia glacialis (Griffiths, 1964) [Griffiths 1964, 1980]
Chromatomyia horticola (Goureau, 1851) [Griffiths 1967, Hackman 1980]
Chromatomyia isicae (Hering, 1962) [Griffiths 1980]
Chromatomyia linnaeae Griffiths, 1974 [Itämies 1995]
Chromatomyia lonicerae (Robineau-Desvoidy, 1851) [Hackman 1980]
= *C. xylostei* (Kaltenbach, 1862) preocc.
Chromatomyia luzulae (Hering, 1924) [Frey 1940, Hackman 1980]
Chromatomyia milii (Kaltenbach, 1864) [Hackman 1980]
Chromatomyia nigra (Meigen, 1830) [Hackman 1980]
Chromatomyia norwegica (Rydén, 1957) [Griffiths 1980]
Chromatomyia ochracea (Hendel, 1920) [Zlobin 1993]
Chromatomyia opacella (Hendel, 1935) [Hackman 1980]
Chromatomyia periclymeni (Hendel, 1922) [Hackman 1980]
Chromatomyia primulae (Robineau-Desvoidy, 1851) [Kaisila 1960, Hackman 1980]
Chromatomyia ramosa (Hendel, 1923) [Hackman 1980]
= *C. nigriventris* (Hendel, 1935)
Chromatomyia styriaca Griffiths, 1980 [Griffiths 1980]
Chromatomyia syngenesiae Hardy, 1849 [Spencer 1976, Hackman 1980]
GALIOMYZA Spencer, 1981
Galiomyza morio (Brischke, 1880) [Hackman 1980]
LIRIOMYZA Mik, 1894
Liriomyza amoena (Meigen, 1830) [Hackman 1980]
Liriomyza angulicornis (Malloch, 1918) [Hackman 1980]
Liriomyza approximata (Hendel, 1920) [Hackman 1980]
Liriomyza artemisicola de Meijere, 1924 [Hackman 1980]

Liriomyza bryoniae (Kaltenbach, 1858) [Hackman 1980]
Liriomyza buhri Hering, 1937 [Karppinen 1964a, Hackman 1980]
= *L. adolescens* Frey, 1946
Liriomyza bulbata Hendel, 1931 [Hackman 1980]
Liriomyza canescens Spencer, 1976 [Hackman 1980]
= *L. graminicola* misid.[Spencer 1976]
Liriomyza cannabis Hendel, 1931 [Hackman 1980]
Liriomyza congesta (Becker, 1903) [Hackman 1980]
Liriomyza demeijerei Hering, 1930 [Hackman 1980]
Liriomyza equiseti de Meijere, 1924 [Haarto and Winqvist 2014]
Liriomyza eupatorii (Kaltenbach, 1873) [Hackman 1980]
Liriomyza flaveola (Fallén, 1823) [Hackman 1980]
Liriomyza flavopicta Hendel, 1931 [Hackman 1980]
Liriomyza freyella Spencer, 1976 [Spencer 1976, Hackman 1980]
Liriomyza hieracii (Kaltenbach, 1862) [Spencer 1976, Hackman 1980]
Liriomyza infuscata Hering, 1926 [Spencer 1976, Hackman 1980]
Liriomyza lutea (Meigen, 1830) [Hackman 1980]
Liriomyza occipitalis Hendel, 1931 [Hackman 1980]
Liriomyza orbona (Meigen, 1830) [Hackman 1980]
Liriomyza pedestris Hendel, 1931 [Hackman 1980]
Liriomyza pseudopygmina (Hering, 1933) [Spencer 1976, Hackman 1980]
Liriomyza ptarmicae de Meijere, 1925 [Spencer 1976, Hackman 1980]
Liriomyza pusilla (Meigen, 1830) [Hackman 1980]
= *L. fasciola* (Meigen, 1838) [Kaisila 1960]
Liriomyza pusio (Meigen, 1830) [Hackman 1980]
= *L. breviseta* Frey, 1946
Liriomyza richteri Hering, 1927 [Hackman 1980]
Liriomyza sonchi Hendel, 1931 [Hackman 1980]
Liriomyza strigata (Meigen, 1830) [Hackman 1980]
Liriomyza tanaceti de Meijere, 1924 [Hackman 1980]
Liriomyza taraxaci Hering, 1927
Liriomyza valerianae Hendel, 1932 [Hackman 1980, Kaisila 1958, Spencer 1976]
Liriomyza virgo (Zetterstedt, 1838) [Hackman 1980]
Liriomyza virgula Frey, 1946 [Hackman 1980]
Liriomyza wachtlii Hendel, 1920 [Hackman 1980]
METOPOMYZA Enderlein, 1936
Metopomyza flavonotata (Haliday, 1833) [Hackman 1980]
Metopomyza interfrontalis (Melander, 1913)
= *M. xanthaspida* (Hendel, 1920) [Hackman 1980]
Metopomyza scutellata (Fallén, 1823) [Spencer 1976, Hackman 1980]
= *M. flavoscutellaris* auct. nec (Zetterstedt, 1838) [Spencer 1976]
Metopomyza xanthaspioides (Frey, 1946) [Hackman 1980]
Metopomyza xanthaspis (Loew, 1858) [Hackman 1980]
NAPOMYZA Westwood, 1840
Napomyza achilleanella von Tschirnhaus, 1992 [Zlobin 1994b]
Napomyza bellidis Griffiths, 1967 [Zlobin 1994b]
Napomyza carotae (Spencer, 1966 [Zlobin 1994b]
Napomyza cichorii (Spencer, 1966 [Zlobin 1994b]
Napomyza elegans (Meigen, 1830) [Hackman 1980]
Napomyza hirticornis Hendel, 1932 [Zlobin 1994b]

Napomyza lateralis (Fallén, 1823) [Hackman 1980]
Napomyza maritima von Tschirnhaus, 1981 [Zlobin 1994b]
Napomyza merita Zlobin, 1993 [Zlobin 1994b]
Napomyza plumea Spencer, 1969 [Spencer 1976, Hackman 1980]
NEMORIMYZA Frey, 1946
Nemorimyza posticata (Meigen, 1830) [Hackman 1980]
PHYTOBIA Lioy, 1864
= ***Dendromyza*** Hendel, 1931
Phytobia aucupariae (Kangas, 1949) [Hackman 1980]
Phytobia cambii (Hendel, 1931) [Hackman 1980]
= *P. betulae* (Kangas, 1935) [Kangas 1935, Hackman 1980, von Tschirnhaus 1992]
= *P. tremulae* (Kangas, 1949)
Phytobia mallochi (Hendel, 1924) [Kangas 1935, Hackman 1980]
PHYTOLIRIOMYZA Hendel, 1931
Phytoliriomyza arctica (Lundbeck, 1901) [Spencer 1976, Hackman 1980]
Phytoliriomyza dorsata (Siebke, 1863) [Hackman 1980]
= *Liriomyza fasciata* misid.[Frey 1946, Spencer 1976]
Phytoliriomyza hilarella (Zetterstedt, 1848) [Hackman 1980]
Phytoliriomyza melampyga (Loew, 1869) [Hackman 1980]
Phytoliriomyza mikii (Strobl, 1898) [Hackman 1980]
Phytoliriomyza ornata (Meigen, 1830) [Hackman 1980]
= *P. elegantula* (Zetterstedt, 1848)
Phytoliriomyza perpusilla (Meigen, 1830) [Hackman 1980]
PHYTOMYZA Fallén, 1810
Phytomyza abdominalis Zetterstedt, 1848 [Hackman 1980]
= *P. gentianae* misid.[Karppinen 1964b]
Phytomyza aconitophila Hendel, 1927 [Hackman 1980]
Phytomyza actaeae Hendel, 1922 [Spencer 1976, Hackman 1980]
Phytomyza adjuncta Hering, 1928 [Hackman 1980]
Phytomyza albiceps Meigen, 1830 [Hackman 1980]
Phytomyza albipennis Fallén, 1823 [Hackman 1980]
Phytomyza anemones Hering, 1925 [Kangas 1952, Hackman 1980]
Phytomyza angelicae Kaltenbach, 1872 [Hackman 1980]
= *P. laserpitii* Hendel, 1924 [Spencer 1976]
Phytomyza angelicastri Hering, 1932 [Hackman 1980]
Phytomyza aquilegiae Hardy, 1849 [Hackman 1980]
Phytomyza aquilonia Frey, 1946 [Hackman 1980]
Phytomyza artemisivora Spencer, 1971 [Spencer 1976, Hackman 1980]
Phytomyza buhriella Spencer, 1969
Phytomyza calthophila Hering, 1931 [Hackman 1980]
Phytomyza campanulae Hendel, 1920 [Hackman 1980]
Phytomyza chaerophylli Kaltenbach, 1856 [Kangas 1952, Hackman 1980]
Phytomyza cirsii Hendel, 1923 [Hackman 1980]
Phytomyza continua Hendel, 1920 [Hackman 1980]
= *P. polyarthrocera* Frey, 1946
Phytomyza crassiseta Zetterstedt, 1860 [Hackman 1980]
Phytomyza dasyops Hendel, 1920 [Hackman 1980]
Phytomyza diversicornis Hendel, 1927 [Kahanpää 2013d]
Phytomyza enigmoides Hering, 1937 [Zlobin 1994a]
= *P. enigma* Hering, 1936 preocc.

Phytomyza erigerophila Hering, 1927 [Hackman 1980]
Phytomyza eumorpha Frey, 1946 [Hackman 1980]
Phytomyza evanescens Hendel, 1920 [Spencer 1976, Hackman 1980]
= *P. calthivora* misid.[Frey 1937, Spencer 1976]
= *P. opaca* misid.[Frey 1937, Spencer 1976]
Phytomyza fallaciosa Brischke, 1880 [Hackman 1980]
Phytomyza flavicornis Fallén, 1823 [Hackman 1980]
Phytomyza flavofemorata Strobl, 1893 [Hackman 1980]
= *P. distantipila* Frey, 1950
= *P. aristata* misid.[Frey 1946, Spencer 1976]
Phytomyza glabra Hendel, 1935 [Hackman 1980]
Phytomyza glechomae Kaltenbach, 1862 [Hackman 1980]
? *Phytomyza gymnostoma* Loew, 1858
Phytomyza hellebori Kaltenbach, 1872 [Hackman 1980]
Phytomyza hendeli Hering, 1923 [Kangas 1952, Hackman 1980]
Phytomyza heracleana Hering, 1937 [Kangas 1952, Winqvist 2007]
Phytomyza hirsuta Spencer, 1976 [Spencer 1976, Hackman 1980]
Phytomyza isais Hering, 1936 [Winqvist 2007]
Phytomyza krygeri Hering, 1949 [Ano 1956, Spencer 1976, Hackman 1980]
Phytomyza lappae Goureau, 1851 [Hackman 1980]
Phytomyza linguae Lundqvist, 1947 [Kaisila 1960]
Phytomyza marginella Fallén, 1823 [Hackman 1980]
Phytomyza minuscula Goureau, 1851 [Hackman 1980]
Phytomyza murina Hendel, 1935 [Kaisila 1960, Hackman 1980]
Phytomyza mylini Hering, 1954 [Winqvist 2007]
Phytomyza nigrifemur Hering, 1934 [Hackman 1980]
= *P. semitenella* Hendel, 1935 [Kaisila 1960]
Phytomyza nigripennis Fallén, 1823 [Hackman 1980]
Phytomyza nigritula Zetterstedt, 1838 [Hackman 1980]
Phytomyza notata Meigen, 1830 [Spencer 1976, Hackman 1980]
Phytomyza obscurella Fallén, 1823 [Hackman 1980]
Phytomyza pauliloewii Hendel, 1920 [Hackman 1980]
Phytomyza pimpinellae Hendel, 1924 [Kangas 1952, Winqvist 2007]
Phytomyza plantaginis Robineau-Desvoidy, 1851 [Hackman 1980]
Phytomyza ptarmicae Hering, 1937 [Hackman 1980]
Phytomyza pubicornis Hendel, 1920 [Kahanpää and Winqvist 2003]
Phytomyza pullula Zetterstedt, 1848 [Hackman 1980]
Phytomyza ranunculi (Schrank, 1803) [Hackman 1980]
Phytomyza ranunculivora Hering, 1932 [Hackman 1980]
Phytomyza rapunculi Hendel, 1927 [Haarto and Winqvist 2014]
= *P. campanulivora* Spencer, 1971
Phytomyza rhabdophora Griffiths, 1964 [Spencer 1976, Hackman 1980]
Phytomyza rostrata Hering, 1934 [Hackman 1980]
Phytomyza rufescens von Roser, 1840 [Spencer 1976, Hackman 1980]
Phytomyza rufipes Meigen, 1830 [Hackman 1980]
Phytomyza rydeni Hering, 1934 [Winqvist 2007]
Phytomyza sedicola Hering, 1924 [Kangas 1952, Hackman 1980]
Phytomyza selini Hering, 1922 [Winqvist 2007]
Phytomyza socia Brischke, 1880 [Kahanpää 2013d]
Phytomyza soenderupi Hering, 1941 [Winqvist 2007]

Phytomyza solidaginis Hendel, 1920 [Hackman 1980]
Phytomyza spinaciae Hendel, 1935 [Spencer 1976, Hackman 1980]
Phytomyza spoliata Strobl, 1906 [Hackman 1980]
Phytomyza spondylii Robineau-Desvoidy, 1851 [Hackman 1980]
= *P. pastinacae* misid.[Frey 1946, Spencer 1976]
Phytomyza subrostrata Frey, 1946 [Hackman 1980]
Phytomyza tanaceti Hendel, 1923 [Hackman 1980]
Phytomyza tenella Meigen, 1830 [Hackman 1980]
Phytomyza thysselini Hendel, 1923 [Winqvist 2007]
Phytomyza trollii Hering, 1930 [Hackman 1980]
Phytomyza tussilaginis Hendel, 1925 [Hackman 1980]
Phytomyza varipes Macquart, 1835 [Hackman 1980]
Phytomyza virgaureae Hering, 1926 [Hackman 1980]
Phytomyza virosae Pakalniškis, 2000 [Winqvist 2007]
Phytomyza wahlgreni Rydén, 1944 [Spencer 1976, Hackman 1980]
= *P. robustella* misid.[Frey 1937, Spencer 1976]
PSEUDONAPOMYZA Hendel, 1920
Pseudonapomyza atra (Meigen, 1830) [Hackman 1980]
Pseudonapomyza europaea Spencer, 1973 [Černý and Merz 2006]
SELACHOPS Wahlberg, 1844
Selachops flavocinctus Wahlberg, 1844 [Hedström 1964, Hackman 1980]

Excluded species

Cerodontha silvatica (Groschke, 1957) [Nowakowski 1973]
Dizygomyza facialis Frey, 1941 [Frey et al. 1941, Spencer 1976]
Liriomyza huidobrensis (Blanchard, 1926) [Valtonen 1993]
Liriomyza sativae Blanchard, 1938 [Brax 1991]
Liriomyza trifolii (Burgess in Comstock, 1880) [Tuovinen and Aapro 1981]
Phytomyza atricornis Meigen, 1838 [Griffiths 1967]
Phytomyza brischkei Hendel, 1922 [Hackman 1980, Spencer 1976]

ANTHOMYZIDAE Czerny, 1903

Supporting references for *Kahanpää J (2014) Checklist of the smaller families of Opomyzoidea, Anthomyzidae, Asteiidae, Aulacigastridae, Clusiidae, Odiniidae, Opomyzidae and Periscelididae (Diptera) of Finland. In: Kahanpää J, Salmela J (Eds) Checklist of the Diptera of Finland ZooKeys @@: @-@. doi: 10.3897/zookeys.??.7141*

ANTHOMYZA Fallén, 1810
Anthomyza collini Andersson, 1976 [Roháček 2006]
Anthomyza dissors Collin, 1944 [Hackman 1980, Andersson 1984]
Anthomyza elbergi Andersson, 1976 [Andersson 1976]
= *A. sordidella* auct. nec(Zetterstedt, 1848) [Hackman 1980]
Anthomyza gracilis Fallén, 1823 [Andersson 1976, Hackman 1980]
Anthomyza macra Czerny, 1928 [Roháček 2006]
Anthomyza pallida (Zetterstedt, 1838) [Andersson 1984, Hackman 1980]
= *A. unguicella* (Zetterstedt, 1838) [Hackman 1980]
Anthomyza paraneglecta Elberg, 1968 [Roháček 2006]
Anthomyza pleuralis Czerny, 1928 [Hackman 1980]
ARGANTHOMYZA Roháček, 2009
Arganthomyza socculata (Zetterstedt, 1847) [Andersson 1976, Hackman 1980]
= *A. ungulata* (Loew, 1873) [Hedström 1964, Hackman 1980]
CAREXOMYZA Roháček, 2009
Carexomyza caricis (Roháček, 1999) [Roháček 2013]
FUNGOMYZA Roháček, 1999
Fungomyza albimana (Meigen, 1830) [Roháček 2013]
PARANTHOMYZA Czerny, 1902
Paranthomyza nitida (Meigen, 1838) [Hackman 1980]
STIPHROSOMA Czerny, 1928
= ***Ptenotaenia*** Enderlein, 1936
Stiphrosoma laetum (Meigen, 1830) [Hackman 1980, Roháček 1996]
= *S. cingulatum* misid.[Hackman 1980, Roháček 1996]
Stiphrosoma sabulosum (Haliday, 1837) [Hackman 1980]
TYPHAMYZA Roháček, 1992
Typhamyza bifasciata (Wood, 1911) [Hackman 1980]

ASTEIIDAE Rondani, 1856

Supporting references for *Kahanpää J (2014) Checklist of the smaller families of Opomyzoidea, Anthomyzidae, Asteiidae, Aulacigastridae, Clusiidae, Odiniidae, Opomyzidae and Periscelididae (Diptera) of Finland. In: Kahanpää J, Salmela J (Eds) Checklist of the Diptera of Finland ZooKeys @@: @-@. doi: 10.3897/zookeys.??.7141*

ASTEIINAE Rondani, 1856
ASTEIA Meigen, 1830
sg. ***Asteia*** Meigen, 1830
Asteia amoena Meigen, 1830 [Flinck and Kahanpää 2013]
Asteia concinna Meigen, 1830 [Hackman 1980]
Asteia elegantula Zetterstedt, 1847 [Hackman 1980]
SIGALOESSINAE Sabrosky, 1956
LEIOMYZA Macquart, 1835
Leiomyza dudai Sabrosky, 1956 [Hackman 1980]
Leiomyza laevigata (Meigen, 1830) [Hackman 1980]
Leiomyza scatophagina (Fallén, 1823) [Val 1929, Hackman 1980]

AULACIGASTRIDAE Duda, 1924

Supporting references for *Kahanpää J (2014) Checklist of the smaller families of Opomyzoidea, Anthomyzidae, Asteiidae, Aulacigastridae, Clusiidae, Odiniidae, Opomyzidae and Periscelididae (Diptera) of Finland. In: Kahanpää J, Salmela J (Eds) Checklist of the Diptera of Finland ZooKeys @@: @-@. doi: 10.3897/zookeys.??.7141*

AULACIGASTER Macquart, 1835
= ***Aulacogaster*** misspelling
Aulacigaster leucopeza (Meigen, 1830) [Haarto and Winqvist 2014]
Aulacigaster pappi Kassebeer, 2001 [Haarto and Winqvist 2014]
= *A. leucopeza* misid.[Hackman 1980]

CLUSIIDAE Handlirsch, 1884

Supporting references for *Kahanpää J (2014) Checklist of the smaller families of Opomyzoidea, Anthomyzidae, Asteiidae, Aulacigastridae, Clusiidae, Odiniidae, Opomyzidae and Periscelididae (Diptera) of Finland. In: Kahanpää J, Salmela J (Eds) Checklist of the Diptera of Finland ZooKeys @@: @-@. doi: 10.3897/zookeys.??.7141*

CLUSIINAE Handlirsch, 1884
CLUSIA Haliday, 1838
= ***Paraclusia*** Czerny, 1903
Clusia flava (Meigen, 1830) [Hackman 1980]
Clusia tigrina (Fallén, 1820) [Hackman 1980]
CLUSIODINAE Johnson, 1913
CLUSIODES Coquillett, 1904
= ***Heteroneura*** Falln, 1823 preocc.
Clusiodes albimanus (Meigen, 1830) [Hackman 1980]
Clusiodes apicalis (Zetterstedt, 1848) [Hackman 1980]
Clusiodes caledonicus (Collin, 1912) [Hackman 1980]
= *C. gentilis* misid.[Frey 1928]
Clusiodes freyi Tuomikoski, 1933 [Tuomikoski 1933, Hackman 1980]
Clusiodes geomyzinus (Fallén, 1823) [Hackman 1980]
Clusiodes microcercus Stackelberg, 1955 [Polevoi 2001a]
Clusiodes pictipes (Zetterstedt, 1855) [Hackman 1980]
Clusiodes ruficollis (Meigen, 1830) [Hackman 1980]
Clusiodes verticalis (Collin, 1912) [Lonsdale and Marshall 2007]
HENDELIA Czerny, 1903
Hendelia beckeri Czerny, 1903 [Hackman 1980]

ODINIIDAE Hendel, 1920

Supporting references for *Kahanpää J (2014) Checklist of the smaller families of Opomyzoidea, Anthomyzidae, Asteiidae, Aulacigastridae, Clusiidae, Odiniidae, Opomyzidae and Periscelididae (Diptera) of Finland. In: Kahanpää J, Salmela J (Eds) Checklist of the Diptera of Finland ZooKeys @@: @-@. doi: 10.3897/zookeys.??.7141*

ODINIINAE Hendel, 1920
NEOALTICOMERUS Hendel, 1903
Neoalticomerus formosus (Loew, 1844) [Hackman 1980]
ODINIA Robineau-Desvoidy, 1830
Odinia boletina (Zetterstedt, 1848) [Nordman 1940, Hackman 1980]
Odinia czernyi Collin, 1952 [MacGowan and Horsfield 2002]
= *O. meijerei* misid.[Ano 1960, Hackman 1980]
Odinia ornata (Zetterstedt, 1838) [Hackman 1980]
Odinia xanthocera Collin, 1952 [Ano 1960, Hackman 1980]

OPOMYZIDAE Fallén, 1820

Supporting references for *ZooKeys @@: @-@*

ANOMALOCHAETA Frey, 1921
Anomalochaeta guttipennis (Zetterstedt, 1838) [Hackman 1980]
GEOMYZA Fallén, 1810
Geomyza angustipennis Zetterstedt, 1847 [Hackman 1980]
Geomyza apicalis (Meigen, 1830) [Hackman 1980]
Geomyza consobrina Zetterstedt, 1847 [Hackman 1980]
Geomyza hackmani Nartshuk, 1984
= *G. combinata* auct. nec(Linnaeus, 1767) [Hackman 1980]
= *G. balachowskyi* misid.
Geomyza majuscula (Loew, 1864)
= *G. breviforceps* Hackman, 1958 [Hackman 1980]
Geomyza martineki Drake, 1992 [Kahanpää 2013d]
Geomyza paganettii (Strobl, 1909) [Flinck and Kahanpää 2013]
Geomyza pilosula Czerny, 1928 [Ano 1960, Hackman 1980]
Geomyza tripunctata Fallén, 1823 [Hackman 1980]
OPOMYZA Fallén, 1820
Opomyza florum (Fabricius, 1794) [Hackman 1980]
Opomyza germinationis (Linnaeus, 1758) [Hackman 1980]
Opomyza lineatopunctata von Roser, 1840 [Kahanpää 2013d]
Opomyza nigriventris Loew, 1865 [Hackman 1980]
Opomyza punctata Haliday, 1833 [Hackman 1980]
Opomyza punctella Fallén, 1820 [Hackman 1980]

Excluded species

Geomyza balachowskyi Mesnil, 1934

PERISCELIDIDAE Oldenberg, 1914

Supporting references for *Kahanpää J (2014) Checklist of the smaller families of Opomyzoidea, Anthomyzidae, Asteiidae, Aulacigastridae, Clusiidae, Odiniidae, Opomyzidae and Periscelididae (Diptera) of Finland. In: Kahanpää J, Salmela J (Eds) Checklist of the Diptera of Finland ZooKeys @@: @-@. doi: 10.3897/zookeys.??.7141*

PERISCELIDINAE Oldenberg, 1914
MYODRIS Lioy, 1864
= ***Microperiscelis*** Oldenberg, 1914
Myodris annulata (Fallén, 1813) [Nordman 1940, Hackman 1980]
PERISCELIS Loew, 1858
Periscelis annulipes Loew, 1858 [Hackman 1980]
Periscelis nigra (Zetterstedt, 1860) [Hackman 1980]
Periscelis winnertzii Egger, 1862 [Haarto and Winqvist 2014]

CHLOROPIDAE Rondani, 1856

Supporting references for *Nartshuk E, Kahanpää J (2014) Checklist of the family Chloropidae (Diptera) of Finland. In: Kahanpää J, Salmela J (Eds) Checklist of the Diptera of Finland. ZooKeys @@: @-@. doi: 10.3897/zookeys.??.7505*

CHLOROPINAE Rondani, 1856
CENTORISOMA Hendel, 1907
Centorisoma elegantulum Becker, 1910
CETEMA Hendel, 1907
  = ***Centor*** Loew, 1866 preocc.
  sg. ***Cetema*** Hendel, 1907
Cetema cereris (Fallén, 1820) [Hackman 1980]
Cetema elongatum (Meigen, 1830) [Hackman 1980]
Cetema myopinum (Loew, 1866) [Hackman 1980]
Cetema neglectum Tonnoir, 1921 [Savage and Wheeler 1999, Nartshuk 1999]
Cetema simile Ismay, 1985 [Savage and Wheeler 1999, Nartshuk 1999]
CHLOROPS Meigen, 1803
  = ***Oscinis*** Latreille, 1804
  sg. ***Chlorops*** Meigen, 1803
Chlorops anthracophagoides Strobl, 1900 [Nartshuk 1998]
Chlorops calceatus Meigen, 1830 [Hackman 1980]
Chlorops centromaculatus Duda, 1933 [Nartshuk 1998]
Chlorops geminatus Meigen, 1830 [Haarto and Winqvist 2014]
Chlorops gracilis Meigen, 1830 [Hackman 1980]
Chlorops hypostigma Meigen, 1830 [Hackman 1980]
Chlorops kirigaminensis Kanmiya, 1978 [Nartshuk 2002]
  = *C. zonulatus* misid.[Hackman 1980, Nartshuk 2002]
Chlorops laetus Meigen, 1830 [Nartshuk and Andersson 2013]
Chlorops meigenii Loew, 1866 [Hackman 1980]
Chlorops nigripalpis (Duda, 1933)
  = *C. crassipalpis* Smirnov, 1958 [Nartshuk 1998]
Chlorops obscurellus (Zetterstedt, 1838) [Hackman 1980]
  = *C. brunnipes* auct. nec (Zetterstedt, 1848) [Andersson 1966]
Chlorops palpatus Smirnov, 1959 [Haarto and Winqvist 2014]
Chlorops pannonicus Strobl, 1893 [Haarto and Winqvist 2014]
Chlorops planifrons (Loew, 1866) [Hackman 1980]
  = *C. triangularis* Becker, 1910 [Hackman 1980, Nartshuk 1998]
Chlorops pumilionis (Bjerkander, 1778) [Hackman 1980]
Chlorops ringens Loew, 1866 [Hackman 1980]
Chlorops riparius Smirnov, 1958 [Haarto and Winqvist 2014]
Chlorops rossicus Smirnov, 1955 [Nartshuk 1998]
Chlorops rufinus (Zetterstedt, 1848) [Hackman 1980]
  = *C. citrinellus* (Zetterstedt, 1848) [Hackman 1980]
Chlorops scalaris Meigen, 1830 [Hackman 1980]
Chlorops scutellaris (Zetterstedt, 1838)
  = *C. laevicollis* Becker, 1910 [Hellén 1946]
  = *C. freyi* Duda, 1933 [Hackman 1980]
Chlorops serenus Loew, 1866 [Nartshuk 1998]
Chlorops speciosus Meigen, 1830 [Hackman 1980]

Chlorops troglodytes (Zetterstedt, 1848) [Hackman 1980]
sg. ***Sclerophallus*** Beschovski, 1978
Chlorops limbatus Meigen, 1830
= *C. brevimanus* Loew, 1866 [Hackman 1980]
Chlorops varsoviensis Becker, 1910 [Hackman 1980]
CHLOROPSINA Becker, 1911
Chloropsina distinguenda (Frey, 1909) [Hackman 1980]
CRYPTONEVRA Lioy, 1864
Cryptonevra diadema (Meigen, 1830) [Hackman 1980]
Cryptonevra flavitarsis (Meigen, 1830) [Frey et al. 1941, Nartshuk 1999]
DIPLOTOXA Loew, 1863
Diplotoxa messoria (Fallén, 1820) [Hackman 1980]
DIPLOTOXOIDES Andersson, 1977 22.3.2012)
Diplotoxoides dalmatina (Strobl, 1900) [Hackman 1980]
EPICHLOROPS Becker, 1910
Epichlorops puncticollis (Zetterstedt, 1848) [Hackman 1980]
EUTROPHA Loew, 1866
Eutropha fulvifrons (Haliday, 1833) [Nartshuk 1999]
Eutropha variegata Loew, 1866 [Hackman 1980]
LASIOSINA Becker, 1910
Lasiosina albipila (Loew, 1866) [Nartshuk 1999]
Lasiosina brevisurstylata Dely-Draskovits, 1977 [Nartshuk 1999]
Lasiosina herpini (Guérin-Méneville, 1843)
= *L. cinctipes* auct. nec (Meigen, 1830) [Hackman 1980]
Lasiosina parvipennis Duda, 1933 [Hackman 1980]
MELANUM Becker, 1910
Melanum laterale (Haliday, 1833) [Hackman 1980]
MEROMYZA Meigen, 1830
sg. ***Meromyza*** Meigen, 1830
Meromyza curvinervis (Zetterstedt, 1848) [Nartshuk 1992]
= *M. hybrida* Péterfi, 1961
Meromyza elbergi Fedoseeva, 1979 [Nartshuk 1992]
Meromyza ingrica Nartshuk, 1992 [Nartshuk 1992]
Meromyza mosquensis Fedoseeva, 1960 [Nartshuk 1992]
Meromyza nigriseta Fedoseeva, 1960 [Nartshuk 1992]
Meromyza nigriventris Macquart, 1835 [Hackman 1980]
= *M. rostrata* Hubicka, 1966 [Nartshuk 1992]
Meromyza ornata (Wiedemann, 1817)
= *M. sororcula* Fedoseeva, 1962 [Nartshuk 1992]
Meromyza palposa Fedoseeva, 1960 [Nartshuk 1992]
Meromyza pluriseta Péterfi, 1961 [Nartshuk 1992]
Meromyza pratorum Meigen, 1830 [Hackman 1980]
Meromyza rohdendorfi Fedoseeva, 1974 [Nartshuk 1992]
Meromyza saltatrix (Linnaeus, 1761) [Hackman 1980]
Meromyza sibirica Fedoseeva, 1961 [Nartshuk and Andersson 2013]
Meromyza triangulina Fedoseeva, 1960 [Nartshuk 1992]
Meromyza variegata Meigen, 1830
= *M. lidiae* Nartshuk, 1992 [Nartshuk 1992]
Meromyza zimzerla Nartshuk, 1992 [Nartshuk 1992]
NEOHAPLEGIS Beschovski, 1981

Neohaplegis tarsata (Fallén, 1820) [Hackman 1980]
PLATYCEPHALA Fallén, 1820
Platycephala planifrons (Fabricius, 1798) [Hackman 1980]
Platycephala umbraculata (Fabricius, 1794) [Hackman 1980]
PSEUDOPACHYCHAETA Strobl, 1902
Pseudopachychaeta approximatonervis (Zetterstedt, 1848) [Hackman 1980]
Pseudopachychaeta oscinina (Fallén, 1813)
= *P. heleocharis* (Nartshuk, 1964) [Nartshuk 1999]
Pseudopachychaeta ruficeps (Zetterstedt, 1838) [Nartshuk 1999]
THAUMATOMYIA Zenker, 1833
Thaumatomyia glabra (Meigen, 1830) [Hackman 1980]
Thaumatomyia hallandica Andersson, 1966 [Hackman 1980]
Thaumatomyia notata (Meigen, 1830) [Hackman 1980]
Thaumatomyia rufa (Macquart, 1835) [Hackman 1980]
Thaumatomyia trifasciata (Zetterstedt, 1848) [Hackman 1980]
TRICHIEURINA Duda, 1933
Trichieurina pubescens (Meigen, 1830) [Kontkanen 1935, Hackman 1980]
OSCINELLINAE Becker, 1910
APHANOTRIGONUM Duda, 1932
Aphanotrigonum cinctellum (Zetterstedt, 1848) [Nartshuk and Andersson 2013]
= *A. fasciella* (Zetterstedt, 1855)
Aphanotrigonum hungaricum Dely-Draskovits, 1981 [Nartshuk and Andersson 2013]
Aphanotrigonum nigripes (Zetterstedt, 1848) [Hackman 1980]
Aphanotrigonum trilineatum (Meigen, 1830) [Hackman 1980]
= *A. beschovskii* Dely-Draskovits, 1981
ASPISTYLA Duda, 1933
Aspistyla plumiger (Meigen, 1830) [Nartshuk and Andersson 2013]
CALAMONCOSIS Enderlein, 1911
sg. ***Calamoncosis*** Enderlein, 1911
Calamoncosis aprica (Meigen, 1830) [Hackman 1980]
Calamoncosis duinensis (Strobl, 1909) [Hackman 1980]
Calamoncosis minima (Strobl, 1893) [Hackman 1980]
Calamoncosis oscinella (Becker, 1910) [Hackman 1980]
sg. ***Rhaphiopyga*** Nartshuk, 1971
Calamoncosis glyceriae Nartshuk, 1958 [Hackman 1980]
COLLINIELLA Nartshuk & Andersson, 2013
Colliniella meijerei (Duda, 1932) [Kahanpää 2013d]
CONIOSCINELLA Duda, 1929
Conioscinella frontella (Fallén, 1820) [Hackman 1980]
Conioscinella gallarum (Duda, 1933) [Hackman 1980]
Conioscinella livida Nartshuk, 1970 [Nartshuk and Andersson 2013]
Conioscinella mimula Collin, 1946 [Nartshuk and Andersson 2013]
Conioscinella sordidella (Zetterstedt, 1848) [Hackman 1980]
Conioscinella zetterstedti Andersson, 1966 [Hackman 1980]
DICRAEUS Loew, 1873
sg. ***Dicraeus*** Loew, 1873
Dicraeus tibialis (Macquart, 1835) [Nartshuk and Andersson 2013]
sg. ***Oedesiella*** Becker, 1910
Dicraeus fennicus Duda, 1932 [Hackman 1980]
Dicraeus rossicus Stackelberg, 1955 [Nartshuk and Andersson 2013]

sg. ***Paroedesiella*** Anonymous in Imperial Institute of Entomology, 1937
Dicraeus nitidus Wahlgren, 1913 [Nartshuk and Andersson 2013]
= *D. napaeus* Collin, 1946
Dicraeus styriacus (Strobl, 1898) [Hackman 1980]
= *D. vallaris* Collin, 1946 [Hackman 1980]
Dicraeus vagans (Meigen, 1838) [Kahanpää and Winqvist 2003]
ELACHIPTERA Macquart, 1835
Elachiptera cornuta (Fallén, 1820) [Hackman 1980]
Elachiptera diastema Collin, 1946 [Kahanpää and Winqvist 2003]
Elachiptera scrobiculata (Strobl, 1901) [Hackman 1980]
= *E. tenuiseta* (Frey, 1947)
Elachiptera tuberculifera (Corti, 1909) [Hackman 1980]
ERIBOLUS Becker, 1910
Eribolus gracilior (de Meijere, 1918) [Nartshuk and Andersson 2013]
Eribolus hungaricus Becker, 1910 [Hackman 1980]
Eribolus nana (Zetterstedt, 1838) [Hackman 1980]
= *E. sudeticus* Becker, 1910
Eribolus slesvicensis Becker, 1910 [Hackman 1980]
GAMPSOCERA Schiner, 1862
Gampsocera numerata (Heeger, 1858) [Hackman 1980]
GAURAX Loew, 1863
Gaurax borealis (Duda, 1933)
Gaurax dubius (Macquart, 1835) [Hackman 1980]
Gaurax ephippium (Zetterstedt, 1848) [Nartshuk and Andersson 2013]
= *G. strobilum* Karps, 1981
Gaurax fascipes Becker, 1910 [Nartshuk and Andersson 2013]
Gaurax macrocerus (Nartshuk, 1962) [Nartshuk and Andersson 2013]
Gaurax maculipennis (Zetterstedt, 1848) [Hackman 1980]
HAPLEGINELLA Duda, 1933
Hapleginella laevifrons (Loew, 1858) [Hackman 1980]
INCERTELLA Sabrosky, 1980
Incertella albipalpis (Meigen, 1830) [Hackman 1980]
Incertella kerteszi (Becker, 1910) [Hackman 1980]
Incertella nigrifrons (Duda, 1933) [Hackman 1980]
Incertella scotica (Collin, 1946) [Nartshuk and Andersson 2013]
Incertella zuercheri (Duda, 1933) [Hackman 1980]
LASIAMBIA Anonymous in Imperial Institute of Entomology, 1937
= ***Goniopsita*** Duda, 1930
Lasiambia brevibucca (Duda, 1932) [Hackman 1980]
Lasiambia coxalis (von Roser, 1840) [Nartshuk and Andersson 2013]
= *L. oophila* (Hennig, 1941)
Lasiambia palposa (Fallén, 1820) [Hackman 1980]
LIPARA Meigen, 1830
Lipara lucens Meigen, 1830 [Valle 1939, Hackman 1980]
Lipara pullitarsis Doskočil & Chvála, 1971 [Koponen 1976, Hackman 1980]
= *L. rufitarsis* misid.[Koponen 1976]
MICROCERCIS Beschovski, 1978
Microcercis trigonella (Duda, 1933) [Hackman 1980]
OSCINELLA Becker, 1909
sg. ***Oscinella*** Becker, 1909

Oscinella angularis Collin, 1946 [Nartshuk and Andersson 2013]
Oscinella cariciphila Collin, 1946 [Nartshuk and Andersson 2013]
Oscinella frit (Linnaeus, 1758) [Hackman 1980]
Oscinella maura (Fallén, 1820) [Hackman 1980]
= *O. albiseta* (Meigen, 1830)
Oscinella nigerrima (Macquart, 1835) [Nartshuk and Andersson 2013]
Oscinella nitidissima (Meigen, 1838) [Nartshuk and Andersson 2013]
Oscinella pusilla (Meigen, 1830) [Nartshuk and Andersson 2013]
Oscinella ventricosi Nartshuk, 1956 [Nartshuk and Andersson 2013]
Oscinella vindicata (Meigen, 1830) [Nartshuk and Andersson 2013]
= *O. hortensis* Collin, 1946
OSCINIMORPHA Lioy, 1864
Oscinimorpha minutissima (Strobl, 1900) [Hackman 1980]
Oscinimorpha sordidissima (Strobl, 1893) [Hackman 1980]
OSCINISOMA Lioy, 1864
Oscinisoma cognatum (Meigen, 1830) [Hackman 1980]
POLYODASPIS Duda, 1933
Polyodaspis ruficornis (Macquart, 1835) [Hackman 1980]
Polyodaspis sulcicollis (Meigen, 1838) [Nartshuk and Andersson 2013]
RHOPALOPTERUM Duda, 1929
Rhopalopterum anthracinum (Meigen, 1930) [Hackman 1980]
Rhopalopterum atricillum (Zetterstedt, 1838) [Hackman 1980]
= *R. atripes* (Duda, 1933)
Rhopalopterum atricorne (Zetterstedt, 1838) [Nartshuk and Andersson 2013]
= *R. platythorax* (Nartshuk, 1958)
Rhopalopterum brunneipenne Beschovski & Lansbury, 1987 [Nartshuk and Andersson 2013]
Rhopalopterum fasciolum (Meigen, 1830) [Nartshuk and Andersson 2013]
Rhopalopterum femorale (Collin, 1946) [Nartshuk and Andersson 2013]
SIPHONELLA Macquart, 1835
Siphonella oscinina (Fallén, 1820) [Hackman 1980]
SIPHUNCULINA Rondani, 1856
Siphunculina aenea (Macquart, 1835) [Hackman 1980]
SPECCAFRONS Sabrosky, 1980
Speccafrons halophila (Duda, 1933) [Hackman 1980]
TRACHYSIPHONELLA Enderlein, 1936
Trachysiphonella ruficeps (Macquart, 1835) [Nartshuk and Andersson 2013]
= *T. pygmaea* (Meigen, 1838)
Trachysiphonella scutellata (von Roser, 1840) [Hackman 1980]
TRICIMBA Lioy, 1864
sg. ***Nartshukiella*** Beschovski, 1981
Tricimba cincta (Meigen, 1830) [Hackman 1980]
= *T. sulcella* (Zetterstedt, 1848)
sg. ***Tricimba*** Lioy, 1864
Tricimba lineella (Fallén, 1820) [Nartshuk and Andersson 2013]

Excluded species

Lasiosina intermedia Dely-Draskovits, 1977
Chlorops figuratus (Zetterstedt, 1848) [Hackman 1980]

Camarota curvipennis (Latreille, 1805)
Eurina lurida Meigen, 1830
Lasiambia baliola (Collin, 1946)

ACARTOPHTHALMIDAE Czerny, 1928

Supporting references for *Kahanpää J (2014) Checklist of the Diptera families Acartophthalmidae, Canacidae (including Tethinidae), Carnidae and Milichiidae of Finland (Insecta). ZooKeys @@: @-@. doi: 10.3897/zookeys.??.7144*

ACARTOPHTHALMUS Czerny, 1902
Acartophthalmus bicolor Oldenberg, 1910 [Hackman 1980]
Acartophthalmus nigrinus (Zetterstedt, 1848) [Hackman 1980]
Acartophthalmus pusio Frey, 1947 [Hackman 1980]

CANACIDAE Jones, 1906

Supporting references for *Kahanpää J (2014) Checklist of the Diptera families Acartophthalmidae, Canacidae (including Tethinidae), Carnidae and Milichiidae of Finland (Insecta). ZooKeys @@: @-@. doi: 10.3897/zookeys.??.7144*

PELOMYIINAE Foster, 1976
PELOMYIELLA Hendel, 1934
Pelomyiella cinerella (Haliday, 1837) [Hackman 1980]

Excluded species

Tethina grisea (Fallén, 1823) [Krogerus 1932]
Tethina illota (Haliday, 1838) [Munari and Mathis 2010]

CARNIDAE Newman, 1834

Supporting references for *Kahanpää J (2014) Checklist of the Diptera families Acartophthalmidae, Canacidae (including Tethinidae), Carnidae and Milichiidae of Finland (Insecta). ZooKeys @@: @-@. doi: 10.3897/zookeys.??.7144*

CARNUS Nitzsch, 1818
Carnus hemapterus Nitzsch, 1818 [Hackman 1980]
MEONEURA Rondani, 1856
Meoneura anceps Frey, 1935 [Frey 1935, Hackman 1980]
Meoneura elongella (Zetterstedt, 1838) [Ano 1960, Hackman 1980]
Meoneura flavifacies Collin, 1930 [Hackman 1980]
Meoneura glaberrima Becker, 1907
= *M. neglecta* Collin, 1930 [Ano 1960, Hackman 1980]
Meoneura lacteipennis (Fallén, 1823) [Hackman 1980]
Meoneura lamellata Collin, 1930 [Hackman 1980]
Meoneura minutissima (Zetterstedt, 1860) [Kahanpää 2013d]
Meoneura neottiophila Collin, 1930 [Hackman 1980]
Meoneura obscurella (Fallén, 1823) [Hackman 1980]
Meoneura prima (Becker, 1903)
= *M. seducta* Collin, 1937 [Ano 1960, Hackman 1980]
Meoneura triangularis Collin, 1930 [Hackman 1980]
Meoneura vagans (Fallén, 1823) [Hackman 1980]

MILICHIIDAE Schiner, 1862

Supporting references for *Kahanpää J (2014) Checklist of the Diptera families Acartophthalmidae, Canacidae (including Tethinidae), Carnidae and Milichiidae of Finland (Insecta). ZooKeys @@: @-@. doi: 10.3897/zookeys.??.7144*

MADIZINAE Czerny, 1909
DESMOMETOPA Loew, 1866
Desmometopa m-nigrum (Zetterstedt, 1848) [Hackman 1980]
Desmometopa sordida (Fallén, 1820) [Hackman 1980]
Desmometopa varipalpis Malloch, 1927 [Haarto and Winqvist 2014]
LEPTOMETOPA Becker, 1903
Leptometopa latipes (Meigen, 1830) [Hackman 1980]
MADIZA Fallén, 1810
Madiza glabra Fallén, 1820 [Hackman 1980]
MILICHIINAE Schiner, 1862
MILICHIA Meigen, 1830
Milichia ludens (Wahlberg, 1847) [Hackman 1980]
PHYLLOMYZINAE Curran, 1934
NEOPHYLLOMYZA Melander, 1913
Neophyllomyza acyglossa (Villeneuve, 1920) [Hackman 1980]
PHYLLOMYZA Fallén, 1810
? *Phyllomyza equitans* (Hendel, 1919) [Hackman 1980]
Phyllomyza formicae Schmitz, 1923 [Hackman 1980]
Phyllomyza longipalpis (Schmitz, 1924)
Phyllomyza rubricornis Schmitz, 1923
Phyllomyza securicornis Fallén, 1823 [Hackman 1980]
Phyllomyza silesiaca (Duda, 1935) [Hackman 1980]

Excluded species

Phyllomyza tetragona Hendel, 1924 [Hackman 1980]

CHYROMYIDAE Hendel, 1916

Supporting references for *Kahanpää J (2014) Checklist of the fly families Chyromyidae and Heleomyzidae (Diptera) of Finland. In: Kahanpää J, Salmela J (Eds) Checklist of the Diptera of Finland. ZooKeys @@: @-@. doi: 10.3897/zookeys.??.7507*

CHYROMYINAE Hendel, 1916
CHYROMYA Robineau-Desvoidy, 1830 134900
Chyromya flava (Linnaeus, 1758) [Hackman 1980]
Chyromya oppidana (Scopoli, 1763) [Hackman 1980]
GYMNOCHIROMYIA Hendel, 1933
Gymnochiromyia flavella (Zetterstedt, 1848)
= *G. minima* (Becker, 1904) [Hackman 1980]
Gymnochiromyia inermis (Collin, 1933) [Ano 1961, Hackman 1980]

HELEOMYZIDAE Westwood, 1840

Supporting references for *Kahanpää J (2014) Checklist of the fly families Chyromyidae and Heleomyzidae (Diptera) of Finland. In: Kahanpää J, Salmela J (Eds) Checklist of the Diptera of Finland. ZooKeys @@: @-@. doi: 10.3897/zookeys.??.7507*

BORBOROPSINAE Griffiths, 1972
BORBOROPSIS Czerny, 1902
Borboropsis puberula (Zetterstedt, 1838) [Hackman 1980]
= *B. fulviceps* (Strobl, 1898)
CHIROPTEROMYZINAE Frey, 1952
CHIROPTEROMYZA Frey, 1952
Chiropteromyza broersei (de Meijere, 1946)
= *C. wegelii* Frey, 1952 [Frey 1952, Hackman 1980]
NEOSSOS Malloch, 1927
= ***Ornitholeria*** Frey, 1930
Neossos nidicola (Frey, 1930) [Frey 1930, Hackman 1980]
HETEROMYZINAE Fallén, 1820 ***HETEROMYZA*** Fallén, 1820
Heteromyza atricornis Meigen, 1830
Heteromyza oculata Fallén, 1820 [Hackman 1980]
Heteromyza rotundicornis (Zetterstedt, 1846) [Kahanpää and Salmela 2007]
TEPHROCHLAMYS Loew, 1862
Tephrochlamys flavipes (Zetterstedt, 1838) [Hackman 1980]
Tephrochlamys rufiventris (Meigen, 1830) [Hackman 1980]
= *T. lapponica* (Czerny, 1924) [Storå 1958]
Tephrochlamys steniusi Frey, 1930 [Frey 1930, Hackman 1980]
Tephrochlamys tarsalis (Zetterstedt, 1847) [Hackman 1980]
HELEOMYZINAE Westwood, 1840
tribe Heleomyzini Westwood, 1840
GYMNOMUS Loew, 1863
Gymnomus amplicornis (Czerny, 1924) [Hackman 1980]
HELEOMYZA Fallén, 1810
= ***Helomyza*** Fallén, 1820 misspelling
= ***Leria*** Robineau-Desvoidy, 1830
sg. **Heleomyza** Fallén, 1810
Heleomyza borealis Boheman, 1865 [Hackman 1968b, 1980]
= *H. czernyi* Collart, 1933
= *H. modesta* misid.[Hackman 1968b]
Heleomyza hackmani Frey, 1950 [Frey 1950, Hackman 1980]
Heleomyza pleuralis (Becker, 1907) [Hackman 1980]
Heleomyza serrata (Linnaeus, 1758) [Hackman 1980]
MORPHOLERIA Garrett, 1921
sg. **Spanoparea** Czerny, 1924
Morpholeria dudai (Czerny, 1924) [Gorodkov 1984]
Morpholeria kerteszii Czerny, 1924 [Hackman 1980]
Morpholeria obscuriventris (Zetterstedt, 1847) [Hackman 1980]
Morpholeria ruficornis (Meigen, 1830) [Hackman 1980]
NEOLERIA Malloch, 1919
Neoleria inscripta (Meigen, 1830) [Hackman 1980]

= *N. minuta* (Zetterstedt, 1838)
Neoleria prominens (Becker, 1897) [Hackman 1968b, 1980]
= *N. tibialis* misid.[Hackman 1968b]
Neoleria ruficauda (Zetterstedt, 1847) [Hackman 1980]
Neoleria ruficeps (Zetterstedt, 1838) [Hackman 1980]
SCOLIOCENTRA Loew, 1862
sg. ***Chaetomus*** Czerny, 1924
Scoliocentra confusa (Wahlgren, 1918) [Hackman 1980]
Scoliocentra flavotestacea (Zetterstedt, 1838) [Hackman 1980]
sg. ***Leriola*** Gorodkov, 1962
Scoliocentra brachypterna (Loew, 1873) [Hackman 1980]
Scoliocentra nigrinervis (Wahlgren, 1918) [Hackman 1980]
sg. ***Scoliocentra*** Loew, 1862
Scoliocentra dupliciseta (Strobl, 1894) [Haarto and Winqvist 2014]
Scoliocentra scutellaris (Zetterstedt, 1838) [Hedström 1964, Hackman 1980]
Scoliocentra villosa [Haarto and Winqvist 2014] (Meigen, 1830)
tribe Oecotheini Gorodkov, 1972
ECCOPTOMERA Loew, 1862
Eccoptomera infuscata Wahlgren, 1918 [Hackman 1963, Storå 1958, Hackman 1980]
Eccoptomera longiseta (Meigen, 1830) [Hackman 1980]
Eccoptomera marginicornis Czerny, 1924 [Ano 1961, Hackman 1980]
Eccoptomera microps (Meigen, 1830) [Ano 1961, Hackman 1980]
Eccoptomera obscura (Meigen, 1830) [Ano 1961, Hackman 1980]
Eccoptomera ornata Loew, 1862 [Ano 1960, Hackman 1980]
Eccoptomera pallescens (Meigen, 1830) [Hackman 1980]
OECOTHEA Haliday, 1837
Oecothea fenestralis (Fallén, 1820) [Hackman 1980]
tribe Orbelliini Gorodkov, 1972
ORBELLIA Robineau-Desvoidy, 1830
Orbellia nivicola Frey, 1913 [Storå 1958, Hackman 1980]
SUILLIINAE Wahlgren, 1917
SUILLIA Robineau-Desvoidy, 1830
= ***Allophyla*** Loew, 1862
Suillia affinis (Meigen, 1830) [Hackman 1980]
Suillia apicalis (Loew, 1862) [Hackman 1972c, 1980]
Suillia atricornis (Meigen, 1830) [Hackman 1980]
Suillia bicolor (Zetterstedt, 1838) [Hackman 1980]
Suillia femoralis (Loew, 1862)
Suillia flava (Meigen, 1830) [Hackman 1980]
Suillia flavifrons (Zetterstedt, 1838) [Hackman 1980]
= *S. nudipes* (Czerny, 1932)
Suillia fuscicornis (Zetterstedt, 1847) [Hackman 1980]
Suillia humilis (Meigen, 1830)
= *S. inornata* (Loew, 1862) [Hackman 1980]
Suillia laevifrons (Loew, 1862) [Hackman 1980]
Suillia lineitergum (Pandellé, 1901) [Flinck and Kahanpää 2013]
= *S. stroblii* (Czerny, 1904)
Suillia lurida (Meigen, 1830) [Kahanpää 2013d]
Suillia mikii (Pokorny, 1886) [Hackman 1980]
Suillia nemorum (Meigen, 1830) [Hackman 1980]

Suillia pallida (Fallén, 1820) [Hackman 1980]
Suillia parva (Loew, 1862)
  = *S. collini* Hackman, 1972 [Hackman 1972c, 1980]
  = *S. flavifrons* auct. nec (Zetterstedt, 1838)
Suillia quadrilineata Czerny, 1924 [Kahanpää 2013d]
Suillia vaginata (Loew, 1862) [Thuneberg 1975, Hackman 1980]
TRIXOSCELIDINAE Hendel, 1916
TRIXOSCELIS Rondani, 1856
Trixoscelis frontalis (Fallén, 1823) [Hackman 1980]
? = *T. canescens* (Loew, 1865) [Flinck and Kahanpää 2013]
Trixoscelis marginella (Fallén, 1823) [Hackman 1980]
Trixoscelis obscurella (Fallén, 1823) [Hackman 1980]
Trixoscelis similis Hackman, 1970 [Hackman 1980, 1970b]

SPHAEROCERIDAE Macquart, 1835

Supporting references for *Haarto A, Kahanpää J (2014) Checklist of the family Sphaeroceridae (Diptera) of Finland. In: Kahanpää J, Salmela J (Eds) Checklist of the Diptera of Finland. ZooKeys @@: @-@. doi: 10.3897/zookeys.??.7250*

COPROMYZINAE Stenhammar, 1854
ALLOBORBORUS Duda, 1923
Alloborborus pallifrons (Fallén, 1820) [Hackman 1980]
= *A. flavipennis* (Haliday, 1836) [Hac 1963, Karppinen 1963]
BORBORILLUS Duda, 1923
Borborillus uncinatus (Duda, 1923) [Karppinen 1963, Hackman 1980]
Borborillus vitripennis (Meigen, 1830) [Hackman 1980]
= *B. longipennis* (Haliday, 1836)
COPROMYZA Fallén, 1810
Copromyza borealis Zetterstedt, 1847 [Hackman 1980]
Copromyza equina Fallén, 1820 [Hackman 1980]
Copromyza nigrina (Gimmerthal, 1847)
= *C. similis* (Collin, 1930) [Hackman 1980]
Copromyza stercoraria (Meigen, 1830) [Hackman 1980]
CRUMOMYIA Macquart, 1835
= ***Apterina*** Macquart, 1835
Crumomyia fimetaria (Meigen, 1830) [Hackman 1963, 1980]
Crumomyia gelida Hackman, 1965 [Hackman 1965, 1980]
Crumomyia glabrifrons (Meigen, 1830) [Hackman 1980]
Crumomyia nigra (Meigen, 1830) [Hackman 1980]
Crumomyia nitida (Meigen, 1830) [Hackman 1963, 1980]
Crumomyia notabilis (Collin, 1902) [Hackman 1980]
= *C. glacialis* misid.[Hackman 1965]
Crumomyia pedestris (Meigen, 1830 [Haarto and Kahanpää 2013]
Crumomyia pruinosa (Richards, 1932) [Norrbom and Kim 1985]
= *C. annulus* misid.[Hackman 1965, 1980, Norrbom and Kim 1985]
= *C. rufoannulata* misid.[Hac 1963, Hackman 1965]
Crumomyia setitibialis (Spuler, 1925)
= *C. freyi* (Hackman, 1965) [Hackman 1965, 1980]
= *C. annulipes* misid.[Hackman 1965]
= *C. roseri* misid.[Hackman 1965]
LOTOPHILA Lioy, 1864
Lotophila atra (Meigen, 1830) [Hackman 1980]
NORRBOMIA Papp, 1988
Norrbomia costalis (Zetterstedt, 1847) [Hackman 1980]
Norrbomia fumipennis (Stenhammar, 1855) [Hackman 1980]
Norrbomia hispanica (Duda, 1923) [Hackman 1980]
Norrbomia sordida (Zetterstedt, 1847) [Hackman 1980]
SPHAEROCERINAE Macquart, 1835
ISCHIOLEPTA Lioy, 1864
Ischiolepta crenata (Meigen, 1838) [Hackman 1980]
Ischiolepta denticulata (Meigen, 1830)
= *I. paracrenata* (Duda, 1920) [Hackman 1980]
Ischiolepta micropyga (Duda, 1938) [Haarto and Kahanpää 2013]

Ischiolepta nitida (Duda, 1920)
= *I. denticulata* auct. nec (Meigen, 1830) [Hackman 1980]
Ischiolepta pusilla (Fallén, 1820) [Hackman 1980]
Ischiolepta scabricula (Haliday, 1836) [Ano 1960, Hackman 1980]
Ischiolepta vaporariorum (Haliday, 1836) [Hackman 1980]
LOTOBIA Lioy, 1864
Lotobia pallidiventris (Meigen, 1830) [Ano 1960, Hackman 1980]
SPHAEROCERA Latreille, 1804
= ***Cypsela*** Meigen, 1800 suppr.
Sphaerocera curvipes Latreille, 1805 [Hackman 1980]
Sphaerocera monilis Haliday, 1836 [Ano 1960, Hackman 1980]
LIMOSININAE Frey, 1921
APTEROMYIA Vimmer, 1929
Apteromyia claviventris (Strobl, 1909) [Hackman 1980]
BIFRONSINA Roháček, 1983
Bifronsina bifrons (Stenhammar, 1855) [Hackman 1980]
CHAETOPODELLA Duda, 1920
sg. ***Chaetopodella*** Duda, 1920
Chaetopodella scutellaris (Haliday, 1836) [Hackman 1980]
COPROICA Rondani, 1861
Coproica acutangula (Zetterstedt, 1847) [Hackman 1980]
Coproica ferruginata (Stenhammar, 1855) [Hackman 1980]
Coproica hirticula Collin, 1965
Coproica hirtula (Rondani, 1880) [Hackman 1980]
Coproica lugubris (Haliday, 1835) [Hackman 1980]
Coproica pusio (Zetterstedt, 1847)
= *C. pseudolugubris* (Duda, 1924) [Hackman 1980]
Coproica vagans (Haliday, 1833) [Hackman 1980]
ELACHISOMA Rondani, 1880
Elachisoma aterrimum (Haliday, 1833) [Hackman 1980]
EULIMOSINA Roháček, 1983
Eulimosina ochripes (Meigen, 1830) [Hackman 1980]
GIGALIMOSINA Roháček, 1983
Gigalimosina flaviceps Zetterstedt, 1847 [Hackman 1980]
GONIONEURA Rondani, 1880
= ***Halidayina*** Duda, 1918
Gonioneura spinipennis (Haliday, 1836) [Hackman 1980]
HERNIOSINA Roháček, 1983
Herniosina bequaerti (Villeneuve, 1917) [Hackman 1980]
LEPTOCERA Olivier, 1813
= ***Paracollinella*** Duda, 1924
Leptocera caenosa (Rondani, 1880) [Hackman 1963, 1980]
Leptocera finalis (Collin, 1956) [Hackman 1980]
Leptocera fontinalis (Fallén, 1826) [Hackman 1980]
Leptocera nigra Olivier, 1813
LIMOSINA Macquart, 1835
Limosina silvatica (Meigen, 1830) [Hackman 1980]
MINILIMOSINA Roháček, 1983
sg. ***Minilimosina*** Roháček, 1983
Minilimosina baculum Marshall, 1985 [Marshall 1985]

Minilimosina bicuspis Roháček, 1993 [Roháček 1993]
= *M. trogeri* misid.[Roháček 1983]
Minilimosina fungicola (Haliday, 1836) [Hackman 1980]
= *M. exigua* (Rondani, 1880)
Minilimosina parvula Stenhammar, 1855 [Roháček 1981]
Minilimosina tenera Roháček, 1983 [Roháček 1993]
sg. ***Svarciella*** Roháček, 1983
Minilimosina guestphalica (Duda, 1918)
= *M. v-atrum* misid.[Hackman 1980, Roháček 1977]
Minilimosina v-atrum (Villeneuve, 1917) [Haarto and Kahanpää 2013]
= *M. splendens* (Duda, 1928)
Minilimosina vitripennis (Zetterstedt, 1847) [Hackman 1980]
OPACIFRONS Duda, 1918
Opacifrons coxata (Stenhammar, 1855) [Hackman 1980]
OPALIMOSINA Roháček, 1983
sg. ***Dentilimosina*** Roháček, 1983
Opalimosina denticulata (Duda, 1924) [Hackman 1980]
sg. ***Opalimosina*** Roháček, 1983
Opalimosina collini (Richards, 1929) [Hackman 1980]
Opalimosina mirabilis (Collin, 1902) [Hackman 1980]
Opalimosina simplex Richards 1929
sg. ***Pappiella*** Roháček, 1983
Opalimosina liliputana (Rondani, 1880)
= *O. appendiculata* Villeneuve, 1918 [Hackman 1980]
PARALIMOSINA Papp, 1973
Paralimosina kaszabi Papp, 1973 [Haarto and Kahanpää 2013]
PHILOCOPRELLA Richards, 1929
Philocoprella quadrispina (Laurence, 1952) [Hac 1963, Karppinen 1963, Hackman 1980]
PHTHITIA Enderlein, 1938
sg. ***Alimosina*** Roháček, 1983
Phthitia empirica (Hutton, 1901) [Hac 1975, Hackman 1980]
= *P. cadaverina* (Duda, 1918)
sg. ***Collimosina*** Roháček, 1983
Phthitia spinosa Collin, 1930 [Hackman 1980]
sg. ***Kimosina*** Roháček, 1983
Phthitia longisetosa (Dahl, 1909) [Hac 1963, Karppinen 1963, Hackman 1980]
Phthitia plumosula (Rondani, 1880) [Hackman 1980]
PSEUDOCOLLINELLA Duda, 1924
Pseudocollinella flavilabris (Hackman, 1968) [Hackman 1968c, 1980]
Pseudocollinella humida (Haliday, 1836) [Hackman 1980]
Pseudocollinella septentrionalis (Stenhammar, 1855) [Hac 1963, Karppinen 1963, Hackman 1980]
PTEREMIS Rondani, 1856
Pteremis fenestralis (Fallén, 1820) [Hackman 1980]
= *P. nivalis* (Haliday, 1833)
= *P. subaptera* Frey, 1946
PULLIMOSINA Roháček, 1983
sg. ***Dahlimosina*** Roháček, 1983
Pullimosina dahli (Duda, 1918) [Hackman 1980]

sg. ***Pullimosina*** Roháček, 1983
Pullimosina heteroneura (Haliday, 1836) [Hackman 1980]
Pullimosina meijerei (Duda, 1918) [Haarto and Kahanpää 2013]
Pullimosina moesta (Villeneuve, 1918)
= *P. antennata* (Duda, 1918) [Hackman 1980]
Pullimosina pullula (Zetterstedt, 1847) [Hackman 1980]
Pullimosina vulgesta Roháček, 2001 [Roháček 2000]
= *P. moesta* auct. nec (Villeneuve, 1918) [Hackman 1980]
RACHISPODA Lioy, 1864
Rachispoda anceps (Stenhammar, 1855) [Roháček 1991]
Rachispoda breviceps (Stenhammar, 1855) [Hackman 1980]
Rachispoda fuscipennis (Haliday, 1833) [Hackman 1980]
Rachispoda hostica Villeneuve, 1917 [Roháček 1991]
Rachispoda intermedia (Duda, 1918) [Roháček 1991]
Rachispoda limosa (Zetterstedt, 1820)
Rachispoda lugubrina (Zetterstedt, 1847) [Roháček 1991]
Rachispoda lutosa (Stenhammar, 1855) [Hackman 1980]
= *R. palustris* (Collin, 1930)
Rachispoda lutosoidea (Duda, 1938) [Hackman 1980]
= *R. lutosa* auct. nec (Stenhammar, 1855)
SPELOBIA Spuler, 1924
Spelobia belanica Roháček, 1983 [Haarto and Kahanpää 2013]
Spelobia cambrica (Richards, 1929) [Hackman 1980]
Spelobia clunipes (Meigen, 1830) [Hackman 1980]
= *S. crassimana* (Haliday, 1836)
Spelobia ibrida Roháček, 1983 [Roháček 1983]
Spelobia luteilabris (Rondani, 1880) [Hackman 1980]
Spelobia manicata (Richards, 1927) [Hackman 1980]
Spelobia nana (Rondani, 1880) [Hackman 1980]
Spelobia palmata (Richards, 1927) [Hackman 1963, 1980]
Spelobia pappi Roháček, 1983 [Haarto and Kahanpää 2013]
Spelobia parapusio (Dahl, 1909) [Hackman 1980]
Spelobia pseudonivalis (Dahl, 1909) [Marshall et al. 2011]
Spelobia pseudosetaria (Duda, 1918) [Hackman 1980]
= *S. penetralis* (Collin, 1925) [Hac 1963, Karppinen 1963]
Spelobia rufilabris (Stenhammar, 1855) [Hackman 1980]
Spelobia talparum (Richards, 1927) [Hackman 1963, 1980]
Spelobia ulla Roháček, 1983 [Roháček 1983, Hackman 1980]
SPINILIMOSINA Roháček, 1983
Spinilimosina brevicostata (Duda, 1918) [Hackman 1980]
TELOMERINA Roháček, 1983
Telomerina eburnea Roháček, 1983 [Marshall and Roháček 1984]
Telomerina flavipes (Meigen, 1830) [Hackman 1963, 1980]
Telomerina pseudoleucoptera (Duda, 1924) [Hackman 1980]
TERRILIMOSINA Roháček, 1983
Terrilimosina racovitzai (Bezzi, 1911) [Hackman 1980]
Terrilimosina schmitzi (Duda, 1918) [Hackman 1980]
THORACOCHAETA Duda, 1918
Thoracochaeta brachystoma (Stenhammar, 1855) [Hackman 1980]
Thoracochaeta zosterae (Haliday, 1833) [Hackman 1980]

TRACHYOPELLA Duda, 1918
sg. ***Nudopella*** Roháček & Marshall, 1986
Trachyopella leucoptera (Haliday, 1836) [Hac 1963, Karppinen 1963, Hackman 1980]
sg. ***Trachyopella*** Duda, 1918
Trachyopella atomus (Rondani, 1880)[Haarto and Kahanpää 2013]
Trachyopella bovilla Collin, 1954 [Roháček and Marshall 1985]
= *T. coprina* misid.[Hac 1963, Karppinen 1963, Hackman 1980, Roháček and Marshall 1985]
Trachyopella lineafrons (Spuler, 1925) [Roháček and Marshall 1985]
= *T. atomus* misid.[Hackman 1980, Roháček and Marshall 1985]
Trachyopella melania (Haliday, 1836) [Hac 1963, Karppinen 1963, Hackman 1980]
= *T. villeneuvii* (Duda, 1924)

Excluded species

Rachispoda cilifera (Rondani, 1880) [Hackman 1980]
Minilimosina unica (Papp, 1973) [Hackman 1980]
= *M. hackmani* (Roháček, 1977) [Hackman 1980, Roháček 1977]

BRAULIDAE Egger, 1853

Supporting references for *Kahanpää J (2014) Checklist of the Braulidae, Camillidae, Diastatidae and Drosophilidae of Finland (Diptera, Ephydroidea). In: Kahanpää J, Salmela J (Eds) Checklist of the Diptera of Finland. ZooKeys @@: @-@. doi: 10.3897/zookeys.??.7075*

BRAULA Nitzsch, 1818
Braula coeca Nitzsch, 1818 [Hackman 1980]

CAMILLIDAE Frey, 1921

Supporting references for *Kahanpää J (2014) Checklist of the Braulidae, Camillidae, Diastatidae and Drosophilidae of Finland (Diptera, Ephydroidea). In: Kahanpää J, Salmela J (Eds) Checklist of the Diptera of Finland. ZooKeys @@: @-@. doi: 10.3897/zookeys.??.7075*

CAMILLA Haliday, 1838
Camilla atrimana Strobl, 1910 [Hackman 1961]
= *C. atripes* Duda, 1934 [Hackman 1980]
= *C. acutipennis* misid.[Basden 1961]
Camilla flavicauda Duda, 1922
= *C. glabrata* Collin, 1956 [Hackman 1961, 1980]
Camilla glabra (Fallén, 1823) [Hackman 1980]
= *C. subfuscipes* Collin, 1933

DIASTATIDAE Hendel, 1917

Supporting references for *Kahanpää J (2014) Checklist of the Braulidae, Camillidae, Diastatidae and Drosophilidae of Finland (Diptera, Ephydroidea). In: Kahanpää J, Salmela J (Eds) Checklist of the Diptera of Finland. ZooKeys @@: @-@. doi: 10.3897/zookeys.??.7075*

CAMPICHOETINAE Griffiths, 1972
CAMPICHOETA Macquart, 1835
Campichoeta griseola (Zetterstedt, 1855) [Chandler 1987, Hackman 1980]
Campichoeta obscuripennis (Meigen, 1830) [Chandler 1987]
DIASTATINAE Hendel, 1917
DIASTATA Meigen, 1830
Diastata adusta Meigen, 1830 [Chandler 1987]
= *D. unipunctata* Zetterstedt, 1847 [Hackman 1980]
Diastata boreonigra Chandler, 1987 [Chandler 1987]
Diastata costata Meigen, 1830 [Chandler 1987]
Diastata flavicosta Chandler, 1987 [Chandler 1987]
Diastata fuscula (Fallén, 1820) [Chandler 1987, Hackman 1980]
Diastata nebulosa (Fallén, 1823) [Chandler 1987, Hackman 1980]
Diastata ornata Meigen, 1830 [Chandler 1987]
Diastata vagans Loew, 1864 [Chandler 1987, Hackman 1980]

DROSOPHILIDAE Rondani, 1856

Supporting references for *Kahanpää J (2014) Checklist of the Braulidae, Camillidae, Diastatidae and Drosophilidae of Finland (Diptera, Ephydroidea). In: Kahanpää J, Salmela J (Eds) Checklist of the Diptera of Finland. ZooKeys @@: @-@. doi: 10.3897/zookeys.??.7075*

STEGANINAE Hendel, 1917
AMIOTA Loew, 1862
sg. ***Amiota*** Loew, 1862
Amiota albilabris (Roth in Zetterstedt, 1860) [Máca 1980]
Amiota alboguttata (Wahlberg, 1839) [Hackman 1980, Val 1929]
Amiota rufescens (Oldenberg, 1914) [Bächli et al. 2004]
Amiota subtusradiata Duda, 1934 [Hac 1971, Hackman 1980]
CACOXENUS Loew, 1858
sg. ***Paracacoxenus*** Hardy & Wheeler, 1960
Cacoxenus argyreator Frey, 1932 [Frey 1932b, Hackman 1980]
LEUCOPHENGA Mik, 1886
sg. ***Neoleucophenga*** Oldenberg, 1915
Leucophenga quinquemaculata Strobl, 1893 [Hackman 1980, Val 1929]
PHORTICA Schiner, 1862
sg. ***Phortica*** Schiner, 1862
Phortica variegata Fallén, 1823 [Val 1929, Hackman 1980]
STEGANA Meigen, 1830
sg. ***Stegana*** Meigen, 1830
Stegana furta (Linnaeus, 1766) [Hackman 1980]
= *S. curvipennis* (Fallén, 1823)
sg. ***Steganina*** Wheeler, 1960
Stegana baechlii Laštovka & Máca, 1982 [Laštovka and Máca 1982]
Stegana coleoptrata (Scopoli, 1763) [Hackman 1980]
Stegana hypoleuca Meigen, 1830
Stegana longifibula Takada, 1968 [Laštovka and Máca 1982]
Stegana mehadiae Duda, 1924 [Laštovka and Máca 1982]
Stegana nigrithorax Strobl, 1898 [Laštovka and Máca 1982]
Stegana similis Laštovka & Máca, 1982 [Laštovka and Máca 1982]
DROSOPHILINAE Rondani, 1856
CHYMOMYZA Czerny, 1903
Chymomyza amoena (Loew, 1862) [Winqvist 2011]
Chymomyza caudatula Oldenberg, 1914 [Karppinen 1969c, Hackman 1980]
Chymomyza costata (Zetterstedt, 1838) [Hackman 1980]
Chymomyza distincta (Egger, 1862) [Hackman 1980, Val 1929]
Chymomyza fuscimana (Zetterstedt, 1838) [Hackman 1980]
DROSOPHILA Fallén, 1823
sg. ***Dorsilopha*** Sturtevant, 1942
Drosophila busckii Coquillett, 1901 [Hackman 1954, 1980]
sg. ***Drosophila*** Fallén, 1823
Drosophila ezoana Takada & Okada, 1957 [Hackman 1980]
Drosophila funebris (Fabricius, 1787) [Hackman 1954, 1980]
Drosophila histrio Meigen, 1830 [Hackman 1954, 1980]
Drosophila hydei Sturtevant, 1921 [Hackman 1968a, 1980]

= *D. repleta* misid.[Hac 1965]
Drosophila immigrans Sturtevant, 1921 [Karppinen 1967, Hackman 1980]
= *D. tripunctata* Becker, 1908 preocc.
Drosophila limbata von Roser, 1840 [Hackman 1954, 1980]
Drosophila littoralis Meigen, 1830 [Hackman 1954, 1980]
Drosophila lummei Hackman, 1972 [Hackman 1972a, 1980]
Drosophila montana Stone, Griffen & Patterson, 1941 [Hackman 1980]
= *D. ovivororum* Lakovaara & Hackman, 1973 [Lakovaara and Hackman 1973]
Drosophila phalerata Meigen, 1830 [Hackman 1954, 1980]
Drosophila picta Zetterstedt, 1847 [Bergman and Nuorteva 1974, Hackman 1980]
Drosophila repleta Wollaston, 1858 [Lakovaara and Itämies 1994]
Drosophila subarctica Hackman, 1969 [Hackman 1969, 1980]
Drosophila testacea von Roser, 1840 [Hackman 1954, 1980]
Drosophila transversa Fallén, 1823 [Hackman 1954, 1980]
Drosophila vireni Bächli, Vilela & Haring, 2002 [Bachli et al. 2002]
sg. ***Sophophora*** Sturtevant, 1942
Drosophila alpina Burla, 1948 [Hackman 1954, 1980]
Drosophila ambigua Pomini, 1940 [Lakovaara and Saura 1970, Hackman 1980]
Drosophila bifasciata Pomini, 1940 [Hackman 1980]
Drosophila eskoi Lakovaara & Lankinen, 1974 [Lakovaara and Lankinen 1974, Hackman 1980]
Drosophila ingrica Hackman, 1957 [Ano 1961, Hackman 1980]
Drosophila melanogaster Meigen, 1830 [Hackman 1954, 1980]
Drosophila obscura Fallén, 1823 [Hackman 1954, 1980]
Drosophila simulans Sturtevant, 1919 [Kangas and Käpylä 1975, Hackman 1980]
Drosophila subobscura Collin, 1936 [Karppinen 1965b, Hackman 1968a, 1980]
Drosophila subsilvestris Hardy & Kaneshiro, 1968 [Hackman 1980]
= *D. silvestris* Basden, 1954 preocc.[Hackman 1968a]
Drosophila tristis Fallén, 1823 [Haarto and Winqvist 2014]
HIRTODROSOPHILA Duda, 1924
Hirtodrosophila cameraria (Haliday, 1833) [Karppinen 1967, Hackman 1980]
Hirtodrosophila confusa (Staeger, 1844) [Bächli et al. 2004]
Hirtodrosophila lundstroemi (Duda, 1935) [Hackman 1954, 1980]
Hirtodrosophila oldenbergi (Duda, 1924) [Kahanpää and Winqvist 2003]
Hirtodrosophila trivittata (Strobl, 1893)
LORDIPHOSA Basden, 1961
Lordiphosa fenestrarum (Fallén, 1823) [Hackman 1954, 1980]
Lordiphosa nigricolor (Strobl, 1898) [Karppinen 1965a, Hackman 1980]
MICRODROSOPHILA Malloch, 1921
sg. ***Microdrosophila*** Malloch, 1921
Microdrosophila congesta (Zetterstedt, 1847) [Bächli et al. 2004]
sg. ***Oxystyloptera*** Duda,1924
Microdrosophila zetterstedti Wheeler, 1959 [Hackman 1980]
= *M. nigriventris* (Zetterstedt, 1847) preocc.[Hackman 1954, Ano 1961]
SCAPTODROSOPHILA Duda, 1923
Scaptodrosophila deflexa (Duda, 1924) [Hac 1969, Hackman 1980]
= *S. guyenoti* (Burla, 1948)
SCAPTOMYZA Hardy, 1849
sg. ***Hemiscaptomyza*** Hackman, 1959
Scaptomyza trochanterata Collin, 1953 [Hackman 1955, 1980]

Scaptomyza unipunctum (Zetterstedt, 1847) [Val 1929, Hackman 1955, 1980]
sg. ***Parascaptomyza*** Duda, 1924
Scaptomyza pallida (Zetterstedt, 1847) [Hackman 1980]
= *S. disticha* (Duda, 1921) [Hackman 1955]
sg. ***Scaptomyza*** Hardy, 1849
Scaptomyza consimilis Hackman, 1955 [Hackman 1955, 1980]
Scaptomyza flava (Fallén, 1823) [Val 1929, Hackman 1980]
= *S. flaveola* (Meigen, 1830) [Hackman 1955]
= *S. apicalis* Hardy, 1849
Scaptomyza graminum (Fallén, 1823) [Val 1929, Hackman 1955, 1980]
Scaptomyza griseola (Zetterstedt, 1847) [Hackman 1955, 1980]
Scaptomyza montana Wheeler, 1949 [Bächli et al. 2004]
Scaptomyza teinoptera Hackman, 1955 [Hackman 1955, 1980]

Excluded species

Stegana strobli Mik, 1898 [Val 1929, Hackman 1980, Laštovka and Máca 1982]
Lordiphosa hexasticha (Papp, 1971)

EPHYDRIDAE Zetterstedt, 1837

Supporting references for *Zatwarnicki T, Kahanpää J (2014) Checklist of the family Ephydridae of Finland (Insecta, Diptera). In: Kahanpää J, Salmela J (Eds) Checklist of the Diptera of Finland. ZooKeys @@: @-@. doi: 10.3897/zookeys.??.7448*

DISCOMYZINAE Acloque, 1897
tribe Discomyzini Acloque, 1897
DISCOMYZA Meigen, 1830
Discomyza incurva (Fallén, 1823) [Fre 1930, Hackman 1980]
tribe Psilopini Cresson, 1925
PSILOPA Fallén, 1823
Psilopa compta (Meigen, 1830) [Hackman 1980]
Psilopa leucostoma (Meigen, 1830) [Hackman 1980]
Psilopa marginella Fallén, 1823 [Hackman 1980]
Psilopa nigritella Stenhammar, 1844 [Fre 1930, Hackman 1980]
Psilopa nitidifacies Frey, 1958
Psilopa nitidula (Fallén, 1813) [Fre 1930, Hackman 1980]
Psilopa polita (Macquart, 1835) [Fre 1930, Hackman 1980]
TRIMERINA Macquart, 1835
Trimerina madizans (Fallén, 1813) [Fre 1930, Hackman 1980]
Trimerina microchaeta Hendel, 1932
= *T. indistincta* Krivosheina, 2004 [Krivosheina 2004a]
HYDRELLIINAE Robineau-Desvoidy, 1830
tribe Atissini Cresson, 1942
ATISSA Haliday, 1839
Atissa limosina Becker, 1896 [Fre 1930, Hackman 1980]
Atissa pygmaea Haliday, 1839 [Winqvist 2011]
tribe Hydrelliini Robineau-Desvoidy, 1830
HYDRELLIA Robineau-Desvoidy, 1830
Hydrellia albifrons Fallén, 1813
Hydrellia albilabris (Meigen, 1830) [Hackman 1980]
Hydrellia cardamines Haliday, 1839
= *H. baltica* Frey, 1930 [Frey 1930, Hackman 1980]
Hydrellia cochleariae Haliday, 1839
= *H. flavicornis* misid.[Hackman 1980]
Hydrellia flaviceps (Meigen, 1830) [Hackman 1980]
= *H. discors* Collin, 1966
= *H. lapponica* misid.[Hackman 1980]
Hydrellia fulviceps (Stenhammar, 1844)
= *H. chrysostoma* misid.[Hackman 1980]
Hydrellia fusca (Stenhammar, 1844) [Hackman 1980]
Hydrellia griseola (Fallén, 1813) [Hackman 1980]
= *H. chrysostoma* (Meigen, 1830)
Hydrellia laticeps (Stenhammar, 1844)
Hydrellia mutata (Zetterstedt, 1846)
Hydrellia obscura (Meigen, 1830) [Hackman 1980]
= *H. fascitibia* misid. [Hackman 1980]
Hydrellia pilitarsis (Stenhammar, 1844) [Hackman 1980]

Hydrellia subalbiceps Collin, 1966
Hydrellia tarsata Haliday, 1839
Hydrellia thoracica Haliday, 1839 [Hackman 1980]
= *H. modesta* misid. [Hackman 1980]
tribe Notiphilini Bigot, 1853
DICHAETA Meigen, 1830
Dichaeta caudata (Fallén, 1813) [Hackman 1980]
= *D. brevicauda* Loew, 1860 [Fre 1930]
NOTIPHILA Fallén, 1810
sg. ***Agrolimna*** Cresson, 1917
Notiphila uliginosa Haliday, 1839 [Hackman 1980]
sg. ***Notiphila*** Fallén, 1810
Notiphila annulipes Stenhammar, 1844 [Hackman 1980]
Notiphila aquatica Becker, 1896 [Hackman 1980]
Notiphila brunipes (Robineau-Desvoidy, 1830) [Hackman 1980]
Notiphila cinerea Fallén, 1813 [Hackman 1980]
Notiphila dorsata Stenhammar, 1844 [Hackman 1980]
Notiphila graecula Becker, 1926 [Krivosheina 1998]
= *N. maculata* misid.[Hackman 1980]
Notiphila major Stenhammar, 1844 [Kahanpää and Winqvist 2003]
Notiphila pollinosa Krivosheina, 1998 [Krivosheina 1998]
Notiphila riparia Meigen, 1830 [Hackman 1980]
GYMNOMYZINAE Latreille, 1829
tribe Gymnomyzini Latreille, 1829
ATHYROGLOSSA Loew, 1860
sg. ***Athyroglossa*** Loew, 1860
Athyroglossa glabra (Meigen, 1830) [Hackman 1980]
MOSILLUS Latreille, 1804
Mosillus subsultans (Fabricius, 1794) [Hackman 1980]
tribe Lipochaetini Coquillett, 1896
GLENANTHE Haliday, 1839
Glenanthe fuscinervis Becker, 1896 [Hackman 1980, Zatwarnicki and Mathis 2011]
= *G. ripicola* auct. nec Haliday, 1839 [Fre 1930, Hackman 1980, Zatwarnicki and Mathis 2011]
tribe Hecamedini Mathis, 1991
ALLOTRICHOMA Becker, 1896
Allotrichoma bezzii Becker, 1896
= *A. laterale* [Hackman 1980] misid.
tribe Ochtherini Dahl, 1959
OCHTHERA Latreille, 1802
Ochthera manicata (Fabricius, 1794) [Irwin 1985, Hackman 1980]
Ochthera mantis (De Geer, 1776) [Irwin 1985, Hackman 1980]
tribe Discocerinini Cresson, 1925
DISCOCERINA Macquart, 1835
Discocerina obscurella (Fallén, 1813) [Hackman 1980]
DITRICHOPHORA Cresson, 1924
Ditrichophora calceata (Meigen, 1830) [Haarto and Winqvist 2014]
Ditrichophora fuscella (Stenhammar, 1844) [Hackman 1980]
GYMNOCLASIOPA Hendel, 1930
Gymnoclasiopa aurivillii (Becker, 1896) [Fre 1930, Hackman 1980]

Gymnoclasiopa bohemanni (Becker, 1896) [Hackman 1980]
Gymnoclasiopa cinerella (Stenhammar, 1844) [Fre 1930, Hackman 1980]
= *G. pulchella* misid.[Winqvist 2002]
Gymnoclasiopa nigerrima (Strobl, 1893)
Gymnoclasiopa psilopina (Frey, 1933) [Hackman 1980]
HECAMEDOIDES Hendel, 1917
Hecamedoides glaucellus (Stenhammar, 1844) [Hackman 1980]
Hecamedoides unispinosus (Collin, 1943) [Kahanpää 2013d]
POLYTRICHOPHORA Cresson, 1924
Polytrichophora duplosetosa (Becker, 1896) [Fre 1930, Hackman 1980]
ILYTHEINAE Cresson, 1943
tribe Hyadinini Philips *et al.* in Cresson, 1949
AXYSTA Haliday, 1839
Axysta cesta (Haliday, 1833) [Fre 1930, Hackman 1980]
HYADINA Haliday, 1839
Hyadina guttata (Fallén, 1813) [Fre 1930, Hackman 1980]
Hyadina humeralis Becker, 1896 [Fre 1930, Hackman 1980]
Hyadina rufipes (Meigen, 1830)
= *H. nitida* (Macquart, 1835) [Hackman 1980]
Hyadina scutellata (Haliday, 1839) [Hackman 1980]
LYTOGASTER Becker, 1896
Lytogaster abdominalis (Stenhammar, 1844) [Fre 1930, Hackman 1980]
NOSTIMA Coquillett, 1900
Nostima picta (Fallén, 1813) [Fre 1930, Hackman 1980]
PELINA Haliday, 1839
Pelina aenea (Fallén, 1813) [Fre 1930, Hackman 1980]
Pelina aenescens (Stenhammar, 1844) [Fre 1930, Hackman 1980]
PHILYGRIA Stenhammar, 1844
Philygria femorata (Stenhammar, 1844) [Hackman 1980]
= *P. posticata* misid.[Fre 1930, Hackman 1980]
Philygria flavipes (Fallén, 1823) [Hackman 1980]
Philygria interstincta (Fallén, 1813)
= *P. maculipennis* (Robineau-Desvoidy, 1830)
= *P. sexmaculata* Becker, 1896 [Fre 1930, Hackman 1980]
Philygria obtecta Becker, 1896 [Hackman 1980]
Philygria vittipennis (Zetterstedt, 1838) [Cogan 1984]
= *P. nigricauda* Stenhammar, 1844 [Hackman 1980]
= *P. trilineata* De Meijere, 1907
tribe Ilytheini Cresson, 1943
ILYTHEA Haliday, 1839
Ilythea spilota (Haliday in Curtis, 1832) [Fre 1930, Hackman 1980]
EPHYDRINAE Zetterstedt, 1837
tribe Parydrini Wirth & Stone, 1956
EUTAENIONOTUM Oldenberg, 1923
Eutaenionotum guttipenne (Stenhammar, 1843) [Fre 1930, Hackman 1980]
PARYDRA Stenhammar, 1844
= ***Napaea*** Robineau-Desvoidy, 1830 preocc.
sg. ***Chaetoapnaea*** Hendel, 1930
Parydra arctica Clausen, 1971 [Krivosheina 2000a]
Parydra fossarum (Haliday, 1833) [Hackman 1980]

Parydra mitis (Cresson, 1930) [Kahanpää 2013d]
Parydra nigritarsis Strobl, 1893 [Haarto and Winqvist 2014]
Parydra pusilla (Meigen, 1830) [Hackman 1980]
Parydra quadripunctata (Meigen, 1830) [Hackman 1980]
sg. ***Parydra*** Stenhammar, 1844
Parydra aquila (Fallén, 1813) [Hackman 1980]
Parydra coarctata (Fallén, 1813) [Hackman 1980]
Parydra nubecula (Becker, 1896) [Hackman 1980]
tribe Ephydrini Zetterstedt, 1837
CALOCOENIA Mathis, 1975
sg. ***Leptocoenia*** Mathis, 1975
Calocoenia paurosoma (Sturtevant & Wheeler, 1954) [Krivosheina 2000a]
COENIA Robineau-Desvoidy, 1830
Coenia curvicauda (Meigen, 1830) [Winqvist 2002]
Coenia palustris (Fallén, 1823) [Fre 1930, Hackman 1980]
Coenia vulgata Krivosheina, 2001
EPHYDRA Fallén, 1810
Ephydra macellaria Egger, 1862 [Hackman 1980]
Ephydra riparia Fallén, 1813 [Hackman 1980]
Ephydra scholtzi Becker, 1896 [Fre 1930, Hackman 1980]
= *E. krogerusi* Frey, 1930 [Frey 1930]
PARACOENIA Cresson, 1935
Paracoenia fumosa (Stenhammar, 1844) [Fre 1930, Hackman 1980]
SETACERA Cresson, 1930
Setacera aurata (Stenhammar, 1844) [Hackman 1980]
Setacera micans (Haliday, 1833) [Fre 1930, Hackman 1980]
tribe Scatellini Wirth & Stone, 1956
HALOSCATELLA Mathis, 1979 [Olafsson 1991]
Haloscatella dichaeta (Loew, 1860) [Hackman 1980]
LAMPROSCATELLA Hendel, 1917 [Olafsson 1991]
Lamproscatella sibilans (Haliday, 1833) [Hackman 1980]
LIMNELLIA Malloch, 1925
Limnellia fallax (Czerny, 1903) [Hackman 1980]
Limnellia quadrata (Fallén, 1813) [Hackman 1980]
Limnellia stenhammari (Zetterstedt, 1846) [Hackman 1980]
PHILOTELMA Becker, 1896
Philotelma defectum (Haliday, 1833) [Mathis et al. 2009]
Philotelma nigripenne (Meigen, 1930) [Hackman 1980]
SCATELLA Robineau-Desvoidy, 1830 [Olafsson 1991]
sg. ***Neoscatella*** Malloch, 1933
Scatella crassicosta Becker, 1896 [Hackman 1980]
Scatella silacea Loew, 1860 [Hackman 1980]
Scatella subguttata (Meigen, 1830) [Hackman 1980]
sg. ***Scatella*** Robineau-Desvoidy, 1830
Scatella obsoleta Loew, 1861
= *S. callosicosta* Bezzi, 1895 [Hackman 1980]
Scatella paludum (Meigen, 1830) [Hackman 1980]
Scatella stagnalis (Fallén, 1813) [Hackman 1980]
Scatella tenuicosta Collin, 1930 [Hackman 1980]
SCATOPHILA Becker, 1896

Scatophila caviceps (Stenhammar, 1844) [Hackman 1980, Zatwarnicki 1987]
Scatophila contaminata (Stenhammar, 1844) [Hackman 1980, Zatwarnicki 1987]
= *S. halterata* Becker, 1896
Scatophila cribrata (Stenhammar, 1844) [Hackman 1980]
Scatophila despecta (Haliday, 1839) [Hackman 1980, Zatwarnicki 1987]
= *S. hamifera* Becker, 1896
Scatophila iowana Wheeler, 1961
Scatophila mesogramma (Loew, 1869)
Scatophila noctula (Meigen, 1830) [Zatwarnicki 1987]
= *S. flavitarsis* (Zetterstedt, 1846) [Hackman 1980]
= *S. laevigata* (Loew, 1860) [Hellén 1946]
= *S. silesiaca* Becker, 1896
Scatophila quadriguttata (Meigen, 1830) [Zatwarnicki 1987]
= *S. variegata* (Loew, 1860) [Hackman 1980]

Excluded species

Gymnoclasiopa aurifacies (Strobl, 1893) [Fre 1935, Hackman 1980]
Gymnoclasiopa plumosa (Fallén, 1923) [Hackman 1980]
Hyadina nigricornis Frey, 1930 [Frey 1930]
Hydrellia argyrogenis Becker, 1896 [Hackman 1980]
Hydrellia concolor (Stenhammar, 1844) [Hackman 1980]
Hydrellia flavicornis (Stenhammar, 1844) [Hackman 1980]
Hydrellia incana (Stenhammar, 1844) [Hackman 1980]
Hydrellia meigeni Zatwarnicki, 1988
= *H. albiceps* (Meigen, 1830) preocc.[Hackman 1980]
Hydrellia nympheae (Stenhammar, 1844) [Hackman 1980]
Hydrellia tibialis Cresson, 1917
= *H. diadema* Frey, 1930 [Frey 1930, Hackman 1980]
Notiphila stagnicola (Robineau-Desvoidy, 1830) [Hackman 1980]
Psilopa pulicaria (Haliday, 1839) [Hackman 1980]
Scatella lutosa Haliday, 1833 [Hackman 1980]

ANTHOMYIIDAE Robineau-Desvoidy, 1830

Supporting references for *Michelsen V (2014) Checklist of the family Anthomyiidae (Diptera) of Finland. In: Kahanpää J, Salmela J (Eds) Checklist of the Diptera of Finland. ZooKeys @@: @-@. doi: 10.3897/zookeys.??.7527*

ADIA Robineau-Desvoidy, 1830
Adia cinerella (Fallén, 1825) [Hackman 1980]
ALLIOPSIS Schnabl & Dziedzicki, 1911
= ***Prosalpia*** Pokorny, 1893 preocc.
= ***Paraprosalpia*** Villeneuve, 1922
= ***Pseudochirosia*** Ringdahl, 1928
Alliopsis albipennis (Ringdahl, 1928) [Dely-Draskovits 1993, Haarto and Winqvist 2014]
Alliopsis aldrichi (Ringdahl, 1934) [Michelsen 2008a]
Alliopsis atronitens (Strobl, 1893) [Hackman 1980]
Alliopsis benanderi (Ringdahl, 1926) [Hackman 1980]
Alliopsis billbergi (Zetterstedt, 1838) [Hackman 1980]
Alliopsis brunneigena (Schnabl, 1915) [Dely-Draskovits 1993]
= *A. incisa* (Ringdahl, 1926)
Alliopsis conifrons (Zetterstedt, 1845) [Dely-Draskovits 1993, Kahanpää and Winqvist 2003]
Alliopsis denticauda (Zetterstedt, 1838) [Tiensuu 1933]
Alliopsis dentiventris (Ringdahl, 1918) [Dely-Draskovits 1993]
Alliopsis fractiseta (Stein, 1908) [Dely-Draskovits 1993]
Alliopsis glacialis (Zetterstedt, 1845) [Tiensuu 1941]
Alliopsis longipennis (Ringdahl, 1918) [Hackman 1980]
Alliopsis maculifrons (Zetterstedt, 1838) [Michelsen 2008a]
Alliopsis moerens (Zetterstedt, 1838) [Hackman 1980]
Alliopsis sepiella (Zetterstedt, 1845) [Hackman 1980]
Alliopsis silvestris (Fallén, 1824) [Hackman 1980]
Alliopsis teriolensis (Pokorny, 1893) [Hackman 1980]
= *A. borealis* (Stein, 1916)
ANTHOMYIA Meigen, 1803
= ***Craspedochoeta*** Macquart, 1851
= ***Craspedochaeta*** misspelling
= ***Chelisia*** Rondani, 1856
Anthomyia canningsi Griffiths, 2001 [Michelsen 2008a]
Anthomyia confusanea Michelsen *in* Michelsen & Baez, 1985 [Michelsen 2008a]
Anthomyia imbrida Rondani, 1866 [Michelsen 2008a]
Anthomyia liturata (Robineau-Desvoidy, 1830)
= *A. pullula* (Zetterstedt, 1845) [Hackman 1980]
Anthomyia mimetica Malloch, 1918
= *A. angulata* (Tiensuu, 1938) [Hackman 1980]
Anthomyia monilis (Meigen, 1826) [Hackman 1980]
Anthomyia plurinotata Brullé, 1832 [Hackman 1980]
Anthomyia pluvialis (Linnaeus, 1758) [Hackman 1980]
Anthomyia procellaris Rondani, 1866 [Michelsen 1980b]
BOREOPHORBIA Michelsen, 1987
Boreophorbia hirtipes (Stein, 1907)
BOTANOPHILA Lioy, 1864

= ***Pegohylemyia*** Schnabl & Dziedzicki, 1911
= ***Monochrotogaster*** Ringdahl, 1932
= ***Pseudomyopina*** Ringdahl, 1933
Botanophila apiciseta (Ringdahl, 1933) [Hackman 1980]
Botanophila betarum Lintner, 1883
= *B. macra* (Karl, 1940) [Hackman 1980]
Botanophila biciliaris Pandellé, 1900 [Michelsen 2008a]
Botanophila bidens (Ringdahl, 1933) [Haarto and Winqvist 2014]
Botanophila brunneilinea (Zetterstedt, 1845) [Hackman 1980]
Botanophila cognata Suwa, 2011
? = *B. subnitida* Malloch, 1920
Botanophila discreta (Meigen, 1826)
= *B. striolata* of authorsin part
Botanophila dissecta (Meigen, 1826)
Botanophila estonica (Elberg, 1970) [Michelsen 2008a]
= *B. varicolor* of authorsin part
Botanophila fugax (Meigen, 1826) [Hackman 1980]
Botanophila gemmata (Zetterstedt, 1860)
Botanophila gnava (Meigen, 1826) [Dely-Draskovits 1993]
Botanophila hucketti (Ringdahl, 1935) [Ringdahl 1935, Hackman 1980]
Botanophila impudica (Rondani, 1866) [Michelsen 2008a]
= *B. varicolor* of authorsin part
Botanophila laterella (Collin, 1967) [Michelsen 2008a]
Botanophila latifrons (Zetterstedt, 1845)
= *B. humeralis* (Hennig, 1970) [Hackman 1980]
Botanophila lobata (Collin, 1967) [Michelsen 2008a]
Botanophila maculipes (Zetterstedt, 1845)
= *B. pseudomaculipes* (Strobl, 1893) [Hackman 1980]
Botanophila miniatura (Huckett, 1965) [Haarto and Winqvist 2014]
Botanophila nigra Michelsen, 2009 [Michelsen 2009a]
Botanophila petrophila (Ringdahl, 1926) [Michelsen 2008a]
Botanophila phrenione (Séguy, 1937) [Väre and Itämies 1995]
Botanophila profuga (Stein, 1916) [Hackman 1980]
Botanophila relativa (Huckett, 1965) [Haarto and Winqvist 2014]
Botanophila rubrifrons (Ringdahl, 1933) [Dely-Draskovits 1993]
Botanophila rubrigena (Schnabl, 1915) [Hackman 1980]
Botanophila salicis (Ringdahl, 1918) [Hackman 1980]
Botanophila sericea (Malloch, 1920)
= *B. obscura* (Zetterstedt, 1845) preocc.[Hackman 1980]
Botanophila silvatica (Robineau-Desvoidy, 1830) [Hackman 1980]
Botanophila sonchi (Hardy, 1872) [Michelsen 2008a]
Botanophila spinosa (Rondani, 1866) [Hackman 1980]
Botanophila striolata (Fallén, 1824) [Hackman 1980]
Botanophila trapezina (Zetterstedt, 1845)
= *B. varicolor* of authorsin part
Botanophila unicolor (Ringdahl, 1932)
Botanophila varicolor (Meigen, 1826) [Hackman 1980]
Botanophila verticella (Zetterstedt, 1838)
= *B. lineatula* (Karl, 1928) [Hackman 1980]
CALYTHEA Schnabl & Dziedzicki, 1911

Calythea nigricans (Robineau-Desvoidy, 1830) [Hackman 1980]
CHIASTOCHETA Pokorny, 1889
Chiastocheta dentifera Hennig, 1953 [Michelsen 2008a]
Chiastocheta inermella (Zetterstedt, 1838) [Hackman 1980]
= *C. trollii* auct. nec in part[Dely-Draskovits 1993]
Chiastocheta macropyga Hennig, 1953 [Michelsen 2008a]
Chiastocheta trollii (Zetterstedt, 1845) [Hackman 1980]
= *C. schnabli* Collin, 1954
CHIROSIA Rondani, 1856
Chirosia albitarsis (Zetterstedt, 1845) [Hackman 1980]
Chirosia betuleti (Ringdahl, 1935) [Hackman 1980, Ringdahl 1935]
Chirosia bisinuata (Tiensuu, 1939) [Hackman 1980]
Chirosia cinerosa (Zetterstedt, 1845)
Chirosia crassiseta Stein, 1908 [Hackman 1980]
Chirosia flavipennis (Fallén, 1823) [Hackman 1980]
Chirosia griseifrons (Séguy, 1923) [Michelsen 2008a]
Chirosia grossicauda Strobl, 1901
= *C. parvicornis* auct. nec (Zetterstedt, 1845) [Hackman 1980]
Chirosia histricina (Rondani, 1866)
= *C. hystrix* (Brischke, 1880) [Hackman 1980]
Chirosia nigripes Bezzi, 1895 [Michelsen 2008a]
= *C. albifrons* Tiensuu, 1938 [Dely-Draskovits 1993]
Chirosia similata (Tiensuu, 1939) [Hackman 1980]
DELIA Robineau-Desvoidy, 1830
= ***Leptohylemyia*** Schnabl & Dziedzicki, 1911
= ***Chortophilina*** Karl, 1928
Delia abruptiseta (Ringdahl, 1935) [Michelsen 2008a]
Delia albula (Fallén, 1825) [Hackman 1980]
Delia angustaeformis (Ringdahl, 1933) [Hackman 1980]
Delia angustifrons (Meigen, 1826) [Hackman 1980]
Delia antiqua (Meigen, 1826) [Hackman 1980]
Delia brassicaeformis (Ringdahl, 1926) [Hackman 1980]
Delia brunnescens (Zetterstedt, 1845) [Hackman 1980]
Delia cardui (Meigen, 1826) [Hackman 1980]
Delia carduiformis Schnabl *in* Schnabl & Dziedzicki, 1911 [Michelsen 2008a]
Delia coarctata (Fallén, 1825) [Hackman 1980]
Delia coarctoides Michelsen, 2007 [Michelsen 2007c]
Delia cregyoglossa (Huckett, 1965) [Michelsen 2008a]
= *D. turcica* Hennig, 1974
Delia criniventris (Zetterstedt, 1860) [Haarto and Winqvist 2014]
Delia cuneata Tiensuu, 1946 [Dely-Draskovits 1993]
Delia diluta (Stein, 1916) [Haarto and Winqvist 2014]
Delia echinata (Séguy, 1923) [Hackman 1980]
Delia fabricii (Holmgren, 1872) [Hackman 1980]
Delia flavogrisea (Ringdahl, 1926)
Delia floralis (Fallén, 1824) [Hackman 1980]
Delia florilega (Zetterstedt, 1845) [Hackman 1980]
Delia frontella (Zetterstedt, 1838) [Hedström 1964, Hackman 1980]
Delia hirtitibia (Stein, 1916) [Hackman 1980]
Delia judicariae (Pokorny, 1893) [Michelsen 2012]

Delia lamelliseta (Stein, 1900) [Kahanpää 2013d]
Delia linearis (Stein, 1898)
= *D. flabellifera* (Pandellé, 1900) [Hackman 1980]
Delia lineariventris (Zetterstedt, 1845) [Hackman 1980]
Delia longicauda (Strobl, 1898) [Dely-Draskovits 1993]
Delia lophota (Pandellé, 1900)
= *D. nuda* (Strobl, 1901) [Hackman 1980]
Delia martini Griffiths, 1993 [Michelsen 2008a]
Delia nigrescens (Rondani, 1877) [Michelsen 2008a]
Delia nudicosta (Ringdahl, 1949)
Delia pallipennis (Zetterstedt, 1838)
= *D. candens* (Zetterstedt, 1845) [Hackman 1980]
Delia penicilliventris Ackland, 2010
= *D. penicillaris* auct. nec (Rondani, 1866)
Delia pilifemur Ringdahl, 1933 [Hackman 1980]
Delia piliventris (Pokorny, 1889) [Haarto and Winqvist 2014]
Delia planipalpis (Stein, 1898) [Hackman 1980]
Delia platura (Meigen, 1826) [Hackman 1980]
Delia pruinosa (Zetterstedt, 1845) [Michelsen 2012]
= *D. flavifrons* (Zetterstedt, 1860) [Hackman 1980]
Delia radicum (Linnaeus, 1758)
= *D. brassicae* (Wiedemann, 1817) [Hackman 1980]
Delia rimiventris Michelsen, 2007 [Michelsen 2007a]
Delia rondanii (Ringdahl, 1918) [Haarto and Winqvist 2014]
Delia setigera (Stein, 1920) [Hackman 1980]
Delia sileni Michelsen, 2012 [Michelsen 2012]
= *D. flavifrons* of authors in part
Delia subalpina (Ringdahl, 1926) [Kahanpää 2013d]
Delia tarsata (Ringdahl, 1918) [Michelsen 2008a]
Delia tenuiventris (Zetterstedt, 1860)
= *D. angustitarsis* (Malloch, 1920) [Hackman 1980]
Delia tumidula Ringdahl, 1949 [Michelsen 2008a]
Delia uniseriata (Stein, 1914) [Hackman 1980]
EGLE Robineau-Desvoidy, 1830
Egle atomaria (Zetterstedt, 1845) [Michelsen 2009b]
Egle brevicornis (Zetterstedt, 1838) [Hackman 1980]
Egle ciliata (Walker, 1849)
= *E. muscaria* auct. nec (Fabricius, 1794) [Hackman 1980]
Egle concomitans (Pandellé, 1900) [Michelsen 2008a]
Egle ignobilis Michelsen, 2009 [Michelsen 2009b]
Egle inermis Ackland, 1970 [Michelsen 2008a]
Egle lyneborgi Ackland & Griffiths, 2003 [Michelsen 2009b]
Egle minuta (Meigen, 1826) [Hackman 1980]
Egle parva Robineau-Desvoidy, 1830 [Hackman 1980]
Egle parvaeformis Schnabl *in* Schnabl & Dziedzicki, 1911 [Hackman 1980]
Egle pilitibia (Ringdahl, 1918) [Michelsen 2008a]
Egle rhinotmeta (Pandellé, 1900) [Hackman 1980]
Egle steini Schnabl *in* Schnabl & Dziedzicki, 1911 [Michelsen 2009b]
Egle subarctica (Huckett, 1965) [Michelsen 2009b]
EMMESOMYIA Malloch, 1917

Emmesomyia grisea (Robineau-Desvoidy, 1830)
= *E. socia* of authorsin part[Hackman 1980]
Emmesomyia socia (Fallén, 1825) [Dely-Draskovits 1993]
= *E. villica* (Meigen, 1838)
EUSTALOMYIA Kowarz, 1873
Eustalomyia festiva (Zetterstedt, 1845) [Hackman 1980]
Eustalomyia hilaris (Fallén, 1823) [Hackman 1980]
Eustalomyia histrio (Zetterstedt, 1838) [Hackman 1980]
Eustalomyia vittipes (Zetterstedt, 1845) [Hackman 1980]
EUTRICHOTA Kowarz, 1893
= ***Eremomyia*** Stein, 1898
= ***Pegomyza*** Schnabl & Dziezicki, 1911
= ***Arctopegomyia*** Ringdahl, 1938
Eutrichota frigida (Zetterstedt, 1845) [Hackman 1980]
Eutrichota labradorensis (Malloch, 1920)
Eutrichota longimana (Pokorny, 1887) [Hackman 1980]
Eutrichota pilimana (Ringdahl, 1918) [Hackman 1980]
Eutrichota praepotens (Wiedemann, 1817) [Hackman 1980]
Eutrichota schineri (Schnabl, 1910)
= *E. socculata* auct. nec (Zetterstedt, 1845) [Hackman 1980]
Eutrichota socculata (Zetterstedt, 1845)
= *E. consanguinea* Tiensuu, 1938 [Hackman 1980]
Eutrichota tunicata (Zetterstedt, 1846) [Haarto and Winqvist 2014]
FUCELLIA Robineau-Desvoidy, 1842
Fucellia fucorum (Fallén, 1819) [Hackman 1980]
Fucellia griseola (Fallén, 1819) [Hackman 1980]
Fucellia tergina (Zetterstedt, 1845) [Lyneborg 1965b, Hackman 1980]
HETEROSTYLODES Hennig, 1967
Heterostylodes macrura (Schnabl *in* Schnabl & Dziedzicki, 1911)
Heterostylodes nominabilis (Collin, 1947)
= *H. pratensis* of authors in part
Heterostylodes obscura (Macquart, 1835)
= *H. pratensis* of authors in part
Heterostylodes pilifera (Zetterstedt, 1845) [Dely-Draskovits 1993]
= *H. pratensis* of authors in part
Heterostylodes pratensis (Meigen, 1826)
HYDROPHORIA Robineau-Desvoidy, 1830
Hydrophoria lancifer (Harris, 1780)
= *H. conica* (Wiedemann, 1817) [Hackman 1980]
Hydrophoria linogrisea (Meigen, 1826) [Hackman 1980]
Hydrophoria silvicola (Robineau-Desvoidy, 1830)
= *H. annulata* auct. nec (Pandellé, 1899) [Hackman 1980]
HYLEMYA Robineau-Desvoidy, 1830
Hylemya nigrimana (Meigen, 1826) [Hackman 1980]
Hylemya urbica Wulp, 1896
= *H. latifrons* Schnabl in Schnabl & Dziedzicki, 1911 [Hackman 1980]
Hylemya vagans (Panzer, 1798)
= *H. strenua* Robineau-Desvoidy, 1830 [Hackman 1980]
Hylemya variata (Fallén, 1823) [Hackman 1980]
HYLEMYZA Schnabl & Dziedzicki, 1911

Hylemyza partita (Meigen, 1826) [Hackman 1980]
LASIOMMA Stein, 1916
= ***Crinurina*** Karl, 1928
= ***Acrostilpina*** Ringdahl, 1929
Lasiomma anthomyinum (Rondani, 1866) [Kahanpää 2013d]
Lasiomma atricauda (Zetterstedt, 1845) [Hackman 1980]
Lasiomma collini (Ringdahl, 1929) [Hackman 1981]
Lasiomma craspedodontum Hsue, 1980 [Haarto and Winqvist 2014]
Lasiomma cuneicorne (Zetterstedt, 1838) [Hackman 1980]
Lasiomma iwasai Suwa, 1978
Lasiomma japonicum Suwa, 1971 [Michelsen 2008a]
Lasiomma latipenne (Zetterstedt, 1838) [Hackman 1980]
Lasiomma monticola Suh & Kwon, 1985
Lasiomma morionellum (Zetterstedt, 1838) [Hackman 1980]
Lasiomma picipes (Meigen, 1826)
= *L. octoguttata* (Zetterstedt, 1845) [Hackman 1980]
Lasiomma quinquelineatum (Ringdahl, 1926) [Hackman 1980]
Lasiomma seminitidum (Zetterstedt, 1845)
Lasiomma strigilatum (Zetterstedt, 1838)
= *L. nitidicauda* (Zetterstedt, 1855) [Hackman 1980]
LEUCOPHORA Robineau-Desvoidy, 1830
Leucophora cinerea Robineau-Desvoidy, 1830 [Hackman 1980]
Leucophora grisella Hennig, 1967 [Hackman 1980]
Leucophora obtusa (Zetterstedt, 1838) [Hackman 1980]
Leucophora sericea Robineau-Desvoidy, 1830 [Hackman 1980]
Leucophora tavastica (Tiensuu, 1939) [Hackman 1980]
Leucophora unilineata (Zetterstedt, 1838)
Leucophora unistriata (Zetterstedt, 1838) [Hackman 1980]
MYCOPHAGA Rondani, 1856
Mycophaga testacea (Gimmerthal, 1834) [Hackman 1980]
MYOPINA Robineau-Desvoidy, 1830
Myopina myopina (Fallén, 1824) [Tiensuu 1941]
= *M. reflexa* Robineau-Desvoidy, 1830
Myopina scoparia (Zetterstedt, 1845) [Hackman 1980]
PARADELIA Ringdahl, 1933
= ***Pegomyiella*** Ringdahl, 1938
= ***Pseudonupedia*** Ringdahl, 1959 nom. nudum
= ***Pseudonupedia*** Huckett, 1971 [Michelsen 2007b]
Paradelia brunneonigra (Schnabl *in* Schnabl & Dziedzicki, 1911) [Michelsen 2007b]
Paradelia intersecta (Meigen, 1826) [Hackman 1980]
Paradelia lunatifrons (Zetterstedt, 1846) [Hackman 1980]
Paradelia lundbeckii (Ringdahl, 1918) [Michelsen 2007b]
PAREGLE Schnabl, 1911
= ***Chionomyia*** Ringdahl, 1933
Paregle atrisquama (Ringdahl, 1948) [Ackland 1989]
Paregle audacula (Harris, 1780)
= *P. radicum* auct. nec (Linnaeus, 1758) [Hackman 1980]
Paregle vetula (Zetterstedt, 1838) [Hackman 1980]
PEGOMYA Robineau-Desvoidy, 1830
= ***Phoraea*** Robineau-Desvoidy, 1830

= ***Chaetopegomya*** Ringdahl, 1938
Pegomya atricauda Ringdahl, 1944 [Hac 1975, Hackman 1976, 1980]
Pegomya avida Hennig, 1973 [Michelsen 2008a]
Pegomya betae (Curtis, 1847)
= *P. hyoscyami* of authorsin part
Pegomya bicolor (Wiedemann, 1817) [Hackman 1980]
Pegomya caesia Stein, 1906 [Haarto and Winqvist 2014]
Pegomya calyptrata (Zetterstedt, 1846) [Hackman 1980]
Pegomya circumpolaris Ackland & Griffiths, 1983 [Ackland 1989, Griffiths 1983]
Pegomya conformis (Fallén, 1825) [Hackman 1980]
Pegomya cunicularia (Rondani, 1866) [Michelsen 2008a]
= *P. hyoscyami* of authorsin part
Pegomya curviphallis Michelsen, 2006 [Michelsen 2006a]
Pegomya deprimata (Zetterstedt, 1845) [Hackman 1976, 1980]
Pegomya exilis (Meigen, 1826)
= *P. hyoscyami* of authorsin part
Pegomya flavifrons (Walker, 1849) [Hackman 1980]
Pegomya flavoscutellata (Zetterstedt, 1838) [Hackman 1980]
Pegomya fulgens (Meigen, 1826) [Ackland 1989, Hackman 1980]
Pegomya furva Ringdahl, 1938 [Ackland 1989, Hackman 1980]
Pegomya fuscinata Tiensuu, 1939 [Dely-Draskovits 1993]
Pegomya geniculata (Bouché, 1834) [Hackman 1980]
Pegomya grahami Michelsen & Ackland, 2009 [Michelsen and Ackland 2009]
Pegomya haemorrhoum (Zetterstedt, 1838) [Dely-Draskovits 1993]
Pegomya holmgreni (Boheman, 1859) [Flinck and Kahanpää 2013]
Pegomya holosteae (Hering, 1924) [Michelsen 2008a]
Pegomya hyoscyami (Panzer, 1809) [Hackman 1980, Michelsen 1980a]
Pegomya incisiva Stein, 1906 [Hackman 1980]
Pegomya interruptella (Zetterstedt, 1855) [Hackman 1980]
Pegomya lurida (Zetterstedt, 1846) [Hackman 1980]
Pegomya maculata Stein, 1906 [Michelsen 2008a]
= *P. atricauda* of authorsin part
Pegomya meridiana (Villeneuve, 1923)
Pegomya notabilis (Zetterstedt, 1846)
= *P. zonata* auct. nec (Zetterstedt, 1838) [Hackman 1980]
Pegomya pallidoscutellata (Zetterstedt, 1852) [Hackman 1979b, 1980]
Pegomya pulchripes (Loew, 1857) [Dely-Draskovits 1993]
= *P. flavipes* (Fallén, 1825)
Pegomya rubivora (Coquillett in Slingerland, 1897) [Hackman 1980]
Pegomya ruficeps (Zetterstedt, 1838) [Hackman 1980]
Pegomya rufina (Fallén, 1825) [Hackman 1980]
Pegomya scapularis (Zetterstedt, 1846)
= *P. pilosa* Stein, 1900 [Hackman 1980]
Pegomya seitenstettensis (Strobl, 1880) [Hackman 1980]
Pegomya setaria (Meigen, 1826) [Hackman 1980]
Pegomya solennis (Meigen, 1826)
= *P. nigritarsis* (Zetterstedt, 1838) [Hackman 1980]
Pegomya steini Hendel, 1925 [Hackman 1980]
Pegomya tabida (Meigen, 1826) [Hackman 1980]
Pegomya tenera (Zetterstedt, 1838)

Pegomya testacea (De Geer, 1776)
= *P. silacea* (Meigen, 1830) [Hackman 1980]
Pegomya transgressa (Zetterstedt, 1846) [Ackland 1989, Griffiths 1983]
Pegomya vanduzeei Malloch, 1919
= *P. versicolor* auct. nec (Meigen, 1826) [Hackman 1980]
Pegomya vittigera (Zetterstedt, 1838) [Hackman 1980]
Pegomya winthemi (Meigen, 1826) [Hackman 1980]
Pegomya zonata (Zetterstedt, 1838)
= *P. tenera* auct. nec (Zetterstedt, 1838) [Hackman 1980]
PEGOPLATA Schnabl & Dziedzicki, 1911
= ***Nupedia*** Karl, 1930
Pegoplata aestiva (Meigen, 1826) [Hackman 1980]
Pegoplata annulata (Pandellé, 1899)
= *P. virginea* auct. nec (Meigen, 1826) [Dely-Draskovits 1993]
Pegoplata infirma (Meigen, 1826) [Hackman 1980]
Pegoplata nigroscutellata (Stein, 1920) [Michelsen 2008a]
Pegoplata palposa (Stein, 1897) [Haarto and Winqvist 2014]
Pegoplata patellans (Pandellé, 1900) [Hackman 1980]
Pegoplata tundrica (Schnabl, 1915) [Hackman 1980]
PHORBIA Robineau-Desvoidy, 1830
Phorbia atrogrisea Tiensuu, 1936 [Hackman 1980, Tiensuu 1935a]
Phorbia curvicauda (Zetterstedt, 1845) [Hackman 1980, Ackland and Michelsen 1986]
Phorbia fascicularis Tiensuu, 1936 [Hackman 1980, Tiensuu 1935a]
Phorbia fumigata (Meigen, 1826)
= *P. securis* Tiensuu, 1936 [Hackman 1980, Tiensuu 1935a]
Phorbia genitalis (Schnabl *in* Schnabl & Dziedzicki, 1911) [Hackman 1980, Tiensuu 1935b]
Phorbia longipilis (Pandellé, 1900) [Hackman 1980]
Phorbia melania Ackland & Michelsen, 1987
= *P. bartaki* misid.[Kahanpää 2013d]
Phorbia moliniaris (Karl, 1917) [Kahanpää 2013d]
Phorbia penicillaris (Stein, 1916) [Hackman 1980]
Phorbia singularis Tiensuu, 1938 [Kahanpää 2013d]
RINGDAHLIA Michelsen, 2014
Ringdahlia curtigena (Ringdahl, 1935) [Dely-Draskovits 1993]
STROBILOMYIA Michelsen, 1988
Strobilomyia anthracina (Czerny, 1906) [Hackman 1980, Michelsen 1988, Tiensuu 1935a]
Strobilomyia infrequens (Ackland, 1965) [Michelsen 1988]
Strobilomyia laricicola (Karl, 1928) [Michelsen 1988]
Strobilomyia sibirica Michelsen, 1988 [Michelsen 1988]
Strobilomyia svenssoni Michelsen, 1988 [Michelsen 2008a]
SUBHYLEMYIA Ringdahl, 1933
Subhylemyia longula (Fallén, 1824) [Hackman 1980]
ZAPHNE Robineau-Desvoidy, 1830
= ***Acroptena*** Pokorny, 1893
Zaphne ambigua (Fallén, 1823) [Hackman 1980]
Zaphne barbiventris (Zetterstedt, 1845) [Hackman 1980]
Zaphne brunneifrons (Zetterstedt, 1838)
Zaphne caudata (Zetterstedt, 1855) [Hackman 1980]

Zaphne divisa (Meigen, 1826) [Hackman 1980]
Zaphne fasciculata (Schnabl, 1915) [Hackman 1980]
Zaphne frontata (Zetterstedt, 1838) [Hackman 1980]
Zaphne ignobilis (Zetterstedt, 1845) [Hackman 1980]
Zaphne inuncta (Zetterstedt, 1838)
= *Z. hyalipennis* (Zetterstedt, 1855) [Hackman 1980]
Zaphne lineatocollis (Zetterstedt, 1838)
= *Z. laticornis* (Ringdahl, 1916) [Hackman 1980]
Zaphne nuda (Schnabl *in* Schnabl & Dziedzicki, 1911) [Hackman 1980]
Zaphne proxima (Malloch, 1920) [Hackman 1980]
Zaphne verticina (Zetterstedt, 1838) [Michelsen 2008a]
Zaphne wierzejskii (Mik, 1867) [Hackman 1980]
Zaphne zetterstedtii (Ringdahl, 1918) [Hanski and Nuorteva 1975]

Excluded species

Botanophila rotundivalva (Ringdahl, 1937) [Hackman 1980]
Delia angustiventris (Zetterstedt, 1845) [Hackman 1980] *Delia penicillosa* Hering, 1974
Lasiomma multisetosum (Ringdahl, 1926) [Hackman 1980] mistake
Pegomya glabroides Michelsen, 2008 [Michelsen 2008b]
Pegomya laticornis (Fallén, 1825) 06fe41ff-90ef-4ce4-8037-b88bad425ecb:1
= *P. genupuncta* Stein, 1906 [Hackman 1980]
Pegomya nigrisquama (Stein, 1888) [Hackman 1980]
a0113082-7316-42a4-91f2-9fed1e894ada:1
Pegomya sociella (Stein, 1906) [Hackman 1980]
Zaphne subarctica (Ringdahl, 1918) [Dely-Draskovits 1993]
Phorbia bartaki Ackland & Michelsen, 1987 [Kahanpää 2013d]

SCATHOPHAGIDAE Robineau-Desvoidy, 1830

Supporting references for *Kahanää J, Haarto A (2014) Checklist of the familes Scathophagidae, Fanniidae and Muscidae of Finland (Insecta, Diptera). In: Kahanpää J, Salmela J (Eds) Checklist of the Diptera of Finland. ZooKeys @@: @-@. doi: 10.3897/zookeys.??.7142*

DELININAE Séguy, 1952
DELINA Robineau-Desvoidy, 1830
Delina nigrita (Fallén, 1819) [Hackman 1980]
LEPTOPA Zetterstedt, 1838
Leptopa filiformis Zetterstedt, 1838 [Hackman 1980]
MICROPSELAPHA Becker, 1894
Micropselapha filiformis (Zetterstedt, 1846) [Hackman 1980]
PARALLELOMMA Strobl, 1894
= ***Chylizosoma*** Hendel, 1924
Parallelomma medium Becker, 1894 [Hackman 1980]
? *Parallelomma paridis* Hering, 1923 [Hackman 1980]
Parallelomma sellatum (Hackman, 1956) [Hackman 1980]
Parallelomma vittatum (Meigen, 1826) [Hackman 1980]
PHROSIA Robineau-Desvoidy, 1830
Phrosia albilabris (Fabricius, 1805) [Hackman 1980]
SCATHOPHAGINAE Robineau-Desvoidy, 1830
ACANTHOCNEMA Becker, 1894
sg. ***Clinoceroides*** Hendel, 1917
Acanthocnema glaucescens (Loew, 1864) [Hackman 1980]
= *A. nigripes* Ringdahl, 1936
ACEROCNEMA Becker, 1894
Acerocnema macrocera (Meigen, 1826)
= *A. tiefi* Becker, 1894 [Hackman 1980]
= *A. pokornyi* Becker, 1894
ALLOMYELLA Malloch, 1923
Allomyella albipennis (Zetterstedt, 1838) [Hackman 1980]
Allomyella frigida [Haarto and Winqvist 2014] (Holmgren, 1883)
= *A. portenkoi* (Stackelberg, 1952) misid.
BOSTRICHOPYGA Becker, 1894
Bostrichopyga crassipes (Zetterstedt, 1838) [Hackman 1980]
CHAETOSA Coquillett, 1898
Chaetosa punctipes (Meigen, 1826) [Hackman 1980]
CLEIGASTRA Macquart, 1835
= ***Cnemopogon*** Rondani, 1856
Cleigastra apicalis (Meigen, 1826) [Hackman 1980]
CONISTERNUM Strobl, 1894
= ***Coniosternum*** Becker, 1894
Conisternum lapponicum (Ringdahl, 1920) [Šifner 2003]
Conisternum obscurum (Fallén, 1819) [Hackman 1980]
Conisternum tinctinerve (Becker, 1894) [Tiensuu 1946b, Hackman 1980]
CORDILURA Fallén, 1810
= ***Cordylura*** Meigen, 1826 misspelling
sg. ***Cordilura*** Fallén, 1810

Cordilura aberrans Becker, 1894 [Hackman 1980]
Cordilura aemula Collin, 1958 [Kahanpää 2013d]
Cordilura atrata Zetterstedt, 1846 [Hackman 1980]
Cordilura ciliata Meigen, 1826 [Hackman 1980]
Cordilura picipes Meigen, 1826 [Hackman 1980]
Cordilura picticornis Loew, 1864 [Hackman 1980]
Cordilura proboscidea Zetterstedt, 1838 [Hackman 1980]
Cordilura pubera (Linnaeus, 1758) [Hackman 1980]
Cordilura pudica Meigen, 1826 [Hackman 1980]
Cordilura rufimana Meigen, 1826 [Hackman 1980]
Cordilura socialis (Becker, 1894) [Hackman 1980]
= *C. freyi* Hackman, 1956
sg. ***Cordilurina*** James, 1955
= ***Parallelomma*** Becker, 1894 preocc.
Cordilura albipes Fallén, 1819 [Hackman 1980]
Cordilura fuscipes Zetterstedt, 1838 [Hackman 1980]
sg. ***Scoliaphleps*** Becker, 1894
Cordilura ustulata Zetterstedt, 1838 [Hackman 1980]
= *C. melanacra* Loew, 1873 [Hackman 1980]
COSMETOPUS Becker, 1894
Cosmetopus dentimana (Zetterstedt, 1838) [Hackman 1980]
= *C. fulvipes* (Zetterstedt, 1838)
Cosmetopus longa (Walker, 1849) [Gorodkov 1986]
= *C. bergrothi* Becker, 1900 in part
= *C. fulvipes* auct. nec (Zetterstedt, 1838) [Hackman 1980]
Cosmetopus ringdahli Andersson, 1974 [Hackman 1980]
= *C. bergrothi* Becker, 1900 in part
ERNONEURA Becker, 1894
Ernoneura argus (Zetterstedt, 1838) [Hackman 1980]
GONARCTICUS Becker, 1894
Gonarcticus abdominalis (Zetterstedt, 1846) [Hackman 1980]
GONATHERUS Rondani, 1856
Gonatherus planiceps (Fallén, 1826) [Hackman 1980]
GIMNOMERA Rondani, 1866
= ***Gymnomera*** misspelling
= ***Cochliarium*** Becker, 1894
Gimnomera albipila (Zetterstedt, 1846) [Hackman 1980]
Gimnomera cuneiventris (Zetterstedt, 1846) [Hackman 1980]
Gimnomera dorsata (Zetterstedt, 1838) [Hackman 1980]
Gimnomera hirta Hendel, 1930 [Hackman 1980]
Gimnomera tarsea (Fallén, 1819) [Hackman 1980]
HEXAMITOCERA Becker, 1894
Hexamitocera loxoceratum (Fallén, 1826) [Hackman 1980]
HYDROMYZA Fallén, 1823
Hydromyza livens (Fabricius, 1794) [Hackman 1980]
MEGAPHTHALMA Becker, 1894
Megaphthalma pallida (Fallén, 1819) [Hackman 1980]
MEGAPHTHALMOIDES Ringdahl, 1936
Megaphthalmoides unilineatus (Zetterstedt, 1838) [Hackman 1980]
MICROPROSOPA Strobl, 1894

Microprosopa haemorrhoidalis (Meigen, 1826) [Hackman 1980]
Microprosopa lacteipennis Ringdahl, 1920 [Hackman 1980]
Microprosopa lineata (Zetterstedt, 1838) [Hackman 1980]
Microprosopa pallidicauda (Zetterstedt, 1838) [Hackman 1980]
NANNA Strobl, 1894
= ***Amaurosoma*** Becker, 1894
Nanna armillata (Zetterstedt, 1846) [Hackman 1980]
Nanna articulata (Becker, 1894) [Hackman 1980]
Nanna bispinosa (Malloch, 1920) [Hackman 1980]
Nanna brevifrons (Zetterstedt, 1838) [Hackman 1980]
Nanna fasciata (Meigen, 1826) [Hackman 1980]
Nanna flavipes (Fallén, 1819) [Hackman 1980]
= *N. minuta* (Becker, 1894) [Hackman 1980]
? = *N. multisetosa* (Hackman, 1956) [Hackman 1980, Chandler 1998]
Nanna inermis (Becker, 1894) [Hackman 1980]
Nanna leucostoma (Zetterstedt, 1846) [Hackman 1980]
Nanna tibiella (Zetterstedt, 1838) [Hackman 1980]
= *N. nigripes* (Zetterstedt, 1846)
NORELLIA Robineau-Desvoidy, 1830
Norellia tipularia (Fabricius, 1794) [Winqvist 2011]
NORELLISOMA Hendel, 1910
Norellisoma lituratum (Wiedemann, 1826) [Hackman 1980]
Norellisoma spinimanum (Fallén, 1819) [Hackman 1980]
OKENIELLA Hendel, 1907
Okeniella caudata (Zetterstedt, 1838) [Hackman 1980]
Okeniella dasyprocta (Loew, 1864) [Hackman 1980]
ORTHACHETA Becker, 1894
Orthacheta pilosa (Zetterstedt, 1838) [Hackman 1980]
PLEUROCHAETELLA Vockeroth, 1965
Pleurochaetella simplicipes (Becker, 1900) [Haarto and Winqvist 2014]
POGONOTA Zetterstedt, 1860
sg. ***Pogonota*** Zetterstedt, 1860
Pogonota barbata (Zetterstedt, 1838) [Hackman 1980]
sg. ***Lasioscelus*** Becker, 1894
Pogonota immunda (Zetterstedt, 1838) [Hackman 1980]
= *P. clavatus* (Zetterstedt, 1846)
Pogonota sahlbergi Becker, 1900 [Hackman 1980]
SCATHOPHAGA Meigen, 1803
= ***Scatophaga*** Frabricius, 1805 misspelling
= ***Scopeuma*** Meigen, 1800 suppr.
= ***Scatomyza*** Fallén, 1810
Scathophaga apicalis Curtis *in* Ross, 1835 [Haarto and Winqvist 2014]
Scathophaga furcata (Say, 1823) [Hackman 1980]
Scathophaga incola Becker, 1900 [Hackman 1980]
Scathophaga inquinata (Meigen, 1826) [Hackman 1980]
= *S. analis* Meigen, 1826
Scathophaga litorea (Fallén, 1819)
Scathophaga lutaria (Fabricius, 1794) [Hackman 1980]
Scathophaga obscurinervis Becker, 1900 [Hackman 1980]
Scathophaga pictipennis Oldenberg, 1923 [Hackman 1980]

Scathophaga scybalaria (Linnaeus, 1758) [Hackman 1980]
Scathophaga stercoraria (Linnaeus, 1758) [Hackman 1980]
Scathophaga suilla (Fabricius, 1794) [Hackman 1980]
SPAZIPHORA Rondani, 1856
Spaziphora hydromyzina (Fallén, 1819) [Hackman 1980]
= *S. fascipes* (Becker, 1894)
STAEGERIA Rondani, 1856
Staegeria kunzei (Zetterstedt, 1821) [Hackman 1980]
TRICHOPALPUS Rondani, 1856
Trichopalpus fraternus (Meigen, 1826) [Hackman 1980]
Trichopalpus nigribasis Curran, 1927
= *T. pilirostris* (Ringdahl, 1936) [Hackman 1980]
Trichopalpus obscurellus (Zetterstedt, 1846) [Hackman 1980]
= *T. subarcticus* (Ringdahl, 1936)

FANNIIDAE Schnabl & Dziezicki, 1911

Supporting references for *Kahanää J, Haarto A (2014) Checklist of the familes Scathophagidae, Fanniidae and Muscidae of Finland (Insecta, Diptera). In: Kahanpää J, Salmela J (Eds) Checklist of the Diptera of Finland. ZooKeys @@: @-@. doi: 10.3897/zookeys.??.7142*

FANNIA Robineau-Desvoidy, 1830
= ***Homalomyia*** Bouché, 1834
= ***Coelomyia*** Haliday, 1840
Fannia aethiops Malloch, 1913 [Kahanpää and Haarto 2013]
Fannia alpina Pont, 1970 [Kahanpää and Haarto 2013]
Fannia armata (Meigen, 1826) [Hackman 1980]
Fannia atra (Stein, 1895) [Haarto 2009]
Fannia canicularis (Linnaeus, 1761) [Hackman 1980]
Fannia carbonaria (Meigen, 1826) [Hackman 1980]
Fannia carbonella (Stein, 1895) [Kahanpää and Haarto 2013]
Fannia coracina (Loew, 1873) [Hackman 1980]
Fannia corvina (Verrall, 1892) [Hackman 1980]
= *F. halterata* Ringdahl, 1918
Fannia cothurnata (Loew, 1873) [Hackman 1980]
Fannia difficilis (Stein, 1895) [Hackman 1980]
Fannia fuscitibia Stein, 1920 [Kahanpää and Haarto 2013]
Fannia fuscula (Fallén, 1825) [Hackman 1980]
Fannia genualis (Stein, 1895) [Hackman 1980]
Fannia gotlandica Ringdahl, 1926 [Kahanpää and Haarto 2013]
Fannia hirticeps (Stein, 1892) [Hackman 1980]
Fannia hirundinis Ringdahl, 1948 [Nuorteva and Järvinen 1961, Hackman 1980]
Fannia immutica Collin, 1939 [Kahanpää and Haarto 2013]
Fannia incisurata (Zetterstedt, 1838) [Hackman 1980]
Fannia latifrontalis Hennig, 1955 [Hellén 1952, Hackman 1980]
Fannia latipalpis (Stein, 1892) [Kahanpää and Haarto 2013]
Fannia lepida (Wiedemann, 1817)
= *F. mutica* (Zetterstedt, 1845) [Hackman 1980]
Fannia leucosticta (Meigen, 1838) [Tiensuu 1946a, Kahanpää and Haarto 2013]
Fannia limbata (Tiensuu, 1938) [Hackman 1980]
= *F. berolinensis* Hennig, 1955
Fannia lucidula (Zetterstedt, 1860) [Pont 2002]
= *F. glaucescens* auct. nec (Zetterstedt, 1845) [Hackman 1980]
Fannia lugubrina (Zetterstedt, 1838) [Hackman 1980]
Fannia lustrator (Harris, 1780)
= *F. hamata* (Macquart, 1835) [Hackman 1980]
Fannia manicata (Meigen, 1826) [Hackman 1980]
Fannia melania (Dufour, 1839) [Hackman 1980]
= *F. ciliata* (Stein, 1895)
Fannia metallipennis (Zetterstedt, 1838) [Kahanpää and Haarto 2013]
= *F. kowarzi* (Verrall, 1892)
Fannia minutipalpis (Stein, 1895) [Hackman 1980]
Fannia mollissima (Haliday, 1840) [Hackman 1980]
= *F. spathulata* (Zetterstedt, 1845)

Fannia monilis (Haliday, 1838) [Hackman 1980]
Fannia pallitibia (Rondani, 1866) [Hackman 1980]
Fannia parva (Stein, 1895) [Hackman 1980]
Fannia pauli Pont, 1997 [Kahanpää and Haarto 2013]
= *F. nitida* (Stein, 1895) preocc.
Fannia polychaeta (Stein, 1895) [Hackman 1980]
Fannia postica (Stein, 1895) [Hackman 1980]
Fannia posticata (Meigen, 1826)
= *F. pretiosa* (Schiner, 1862) [Hackman 1980, Tiensuu 1935a]
Fannia rabdionata Karl, 1940 [Karl 1940, Hackman 1980]
Fannia ringdahlana Collin, 1939 [Hackman 1980]
= *F. umbrosa* auct. nec (Stein, 1895)
Fannia rondanii (Strobl, 1893) [Hackman 1980]
= *F. carbonaria* (Rondani, 1871) preocc.
= *F. aerea* misid.
Fannia scalaris (Fabricius, 1794) [Hackman 1980]
= *F. subscalaris* Zimin, 1946
Fannia serena (Fallén, 1825) [Hackman 1980]
Fannia similis (Stein, 1895) [Hackman 1980]
Fannia slovaca Gregor & Rozkošný, 2005 [Kahanpää and Haarto 2013]
Fannia sociella (Zetterstedt, 1845) [Hackman 1980]
Fannia spathiophora Malloch 1918
= *F. nodulosa* Ringdahl, 1926 [Hackman 1980]
Fannia speciosa (Villeneuve, 1898) [Mee 1961a, Hackman 1980]
Fannia stigi Rognes, 1982 [Kahanpää and Haarto 2013]
Fannia subatripes d'Assis-Fonseca, 1967 [Kahanpää and Haarto 2013]
Fannia subpellucens (Zetterstedt, 1845) [Hackman 1980]
Fannia subpubescens Collin, 1958 [Hedström 1968, Kahanpää and Haarto 2013]
Fannia tuberculata (Zetterstedt, 1849) [Hackman 1980]
Fannia umbratica Collin, 1939 [Kahanpää and Haarto 2013]
Fannia umbrosa (Stein, 1895) [Kahanpää and Haarto 2013]
= *F. subumbrosa* Ringdahl, 1934
Fannia verrallii (Stein, 1895) [Kahanpää and Haarto 2013]
Fannia vesparia (Meade, 1891) [Kahanpää and Haarto 2013]
Fannia vespertilionis Ringdahl, 1934 [Kahanpää and Haarto 2013]
PIEZURA Rondani, 1844
= ***Platycoenosia*** Strobl, 1894
Piezura graminicola (Zetterstedt, 1846) [Kahanpää and Haarto 2013]
= *P. boletorum* (Rondani, 1866)
= *P. mikii* (Strobl, 1894)
Piezura pardalina (Rondani, 1866) [Kahanpää and Haarto 2013]
= *P. graminicola* auct. nec (Zetterstedt, 1846)

Excluded species

Fannia barbata (Stein, 1892) [Rozkošný et al. 1997]
Fannia lineata (Stein, 1895) [Hackman 1980]
Fannia nigra Malloch, 1910 [Rozkošný et al. 1997]

MUSCIDAE Latreille, 1802

Supporting references for *Kahanää J, Haarto A (2014) Checklist of the familes Scathophagidae, Fanniidae and Muscidae of Finland (Insecta, Diptera). In: Kahanpää J, Salmela J (Eds) Checklist of the Diptera of Finland. ZooKeys @@: @-@. doi: 10.3897/zookeys.??.7142*

ACHANTHIPTERINAE Hennig, 1962
ACHANTHIPTERA Rondani, 1856
Achanthiptera rohrelliformis (Robineau-Desvoidy, 1830) [Hackman 1980]
  = *A. inanis* (Fallén, 1825) preocc.
COENOSIINAE Verrall, 1888
  tribe Coenosiini Verrall, 1888
COENOSIA Meigen, 1826
  = ***Caricea*** Robineau-Desvoidy, 1830
  = ***Oplogaster*** Rondani, 1856
  = ***Dexiopsis*** Pokorny, 1893
Coenosia acuminata Strobl, 1898 [Hackman 1980]
  = *C. annulipes* Ringdahl, 1932 [Ringdahl 1932b]
Coenosia agromyzina (Fallén, 1825) [Winqvist 2011, Flinck and Kahanpää 2013]
Coenosia ambulans Meigen, 1826 [Hackman 1980]
Coenosia bilineella (Zetterstedt, 1838) [Hackman 1980]
Coenosia campestris (Meigen, 1830)
  = *C. sexnotata* auct. nec Meigen, 1826 [Hackman 1980]
? *Coenosia comita* (Huckett, 1936)
  = *C. ovulifera* Tiensuu, 1938 [Hackman 1980]
Coenosia dealbata (Zetterstedt, 1838)
  = *C. fulvicornis* (Zetterstedt, 1845) [Hackman 1980]
Coenosia flavimana (Zetterstedt, 1845)
  = *C. albatella* (Zetterstedt, 1849) [Hackman 1980]
Coenosia humilis Meigen, 1826 [Hackman 1980]
Coenosia intermedia (Fallén, 1825) [Hackman 1980]
Coenosia lacteipennis (Zetterstedt, 1845) [Haarto and Mukkala 2013]
Coenosia lineatipes (Zetterstedt, 1845) [Mukkala et al. 2005, Haarto 2009]
  = *C. albicornis* misid.
Coenosia means Meigen, 1826 [Kahanpää and Winqvist 2003]
Coenosia mollicula (Fallén, 1825) [Hackman 1980]
Coenosia octopunctata (Zetterstedt, 1838) [Hackman 1980]
Coenosia paludis Tiensuu, 1939 [Hackman 1980]
Coenosia pedella (Fallén, 1825) [Hackman 1980]
  = *C. decipiens* Meigen, 1826
Coenosia perpusilla Meigen, 1826 [Hackman 1980]
Coenosia pudorosa Collin, 1953 [Kahanpää 2013d]
Coenosia pulicaria (Zetterstedt, 1845) [Hackman 1980]
Coenosia pumila (Fallén, 1825) [Hackman 1980]
Coenosia pygmaea (Zetterstedt, 1845) [Hackman 1980]
Coenosia ruficornis Macquart, 1835
  = *C. litoralis* (Zetterstedt, 1846) [Hackman 1980]
Coenosia rufipalpis Meigen, 1826 [Hedström 1968]
  = *C. flavicauda* Ringdahl, 1937

Coenosia sallae Tiensuu, 1938 [Kahanpää 2013d]
Coenosia testacea (Robineau-Desvoidy, 1830)
= *C. tricolor* (Zetterstedt, 1845) [Hackman 1980]
= *C. alleni* d'Assis-Fonseca, 1966
Coenosia tigrina (Fabricius, 1775) [Hackman 1980]
Coenosia trilineella (Zetterstedt, 1838)
= *C. trilineata* misspelling[Hackman 1980]
Coenosia verralli Collin, 1953 [Haarto and Mukkala 2013]
= *C. steini* Verrall, 1912 preocc.
LIMNOSPILA Schnabl, 1902
Limnospila albifrons (Zetterstedt, 1849) [Hackman 1980]
LISPOCEPHALA Pokorny, 1893
= ***Caricea***
Lispocephala alma (Meigen, 1826) [Hackman 1980]
Lispocephala erythrocera (Robineau-Desvoidy, 1830) [Hackman 1980]
Lispocephala falculata Collin, 1963 [Haarto and Winqvist 2014]
Lispocephala fuscitibia Ringdahl, 1944 [Kahanpää 2013d]
Lispocephala pallipalpis (Zetterstedt, 1845) [Haarto and Winqvist 2014]
Lispocephala spuria (Zetterstedt, 1838) [Hackman 1980]
= *L. vitripennis* Ringdahl, 1951
Lispocephala verna (Fabricius, 1794) [Hackman 1980]
MACRORCHIS Rondani, 1877
Macrorchis meditata (Fallén, 1825) [Hackman 1980]
PSEUDOCOENOSIA Stein, 1916
Pseudocoenosia abnormis Stein, 1916 [Hackman 1980]
Pseudocoenosia solitaria (Zetterstedt, 1838) [Hackman 1980]
= *P. longicauda* (Zetterstedt, 1860)
SCHOENOMYZA Haliday, 1833
Schoenomyza litorella (Fallén, 1823) [Hackman 1980]
tribe Limnophoriini Villeneuve, 1902
LIMNOPHORA Robineau-Desvoidy, 1830
Limnophora nigripes (Robineau-Desvoidy, 1830) [Hackman 1980]
Limnophora pandellei Séguy 1923 [Kahanpää 2013d]
Limnophora riparia (Fallén, 1824) [Hackman 1980]
Limnophora rotundata Collin, 1930 [Michelsen 2006b]
Limnophora tigrina (Am Stein, 1860) [Hackman 1980]
= *L. notata* (Fallén, 1823) preocc.
Limnophora triangula (Fallén, 1825) [Hackman 1980]
Limnophora uniseta Stein, 1916 [Hackman 1980]
LISPE Latreille, 1796
Lispe consanguinea Loew, 1858 [Hackman 1980]
Lispe hydromyzina Fallén, 1825 [Hackman 1980]
Lispe litorea Fallén, 1825 [Mee 1928, Hackman 1980]
Lispe melaleuca Loew, 1847 [Kahanpää 2013d]
Lispe pygmaea Fallén, 1825 [Hackman 1980]
Lispe tentaculata (De Geer, 1776) [Hackman 1980]
Lispe uliginosa Fallén, 1825 [Hackman 1980]
SPILOGONA Schnabl, 1911
Spilogona aerea (Fallén, 1825) [Hackman 1980]
Spilogona albisquama (Ringdahl, 1932) [Kahanpää 2013d]

Spilogona alpica (Zetterstedt, 1845) [Hackman 1980]
Spilogona arenosa (Ringdahl, 1918) [Hackman 1980]
Spilogona atrisquamula Hennig, 1959 [Kahanpää 2013d]
Spilogona baltica (Ringdahl, 1918) [Hackman 1980]
Spilogona brunneifrons Ringdahl, 1931 [Haarto and Winqvist 2014]
Spilogona brunneisquama (Zetterstedt, 1845) [Hedström and Nuorteva 1971]
Spilogona carbonella (Zetterstedt, 1845) [Hackman 1980]
Spilogona contractifrons (Zetterstedt, 1838) [Hackman 1980]
Spilogona denigrata (Meigen, 1826) [Hackman 1980]
Spilogona depressiuscula (Zetterstedt, 1838) [Hackman 1980]
Spilogona depressula (Zetterstedt, 1845) [Hackman 1980]
Spilogona dispar (Fallén, 1823)
= *S. funeralis* Rondani, 1866 [Hackman 1980]
Spilogona falleni Pont, 1984
= *S. litorea* auct. nec (Fallén, 1823) [Hackman 1980]
Spilogona krogerusi (Ringdahl, 1941) [Hackman 1980]
= *S. micans* misid.[Hackman 1980]
Spilogona leucogaster (Zetterstedt, 1838) [Hackman 1980]
Spilogona malaisei (Ringdahl, 1920) [Kahanpää 2013d]
Spilogona marginifera Hennig, 1959 [Hackman 1980]
= *S. marginalis* (Fallén, 1824) preocc.
Spilogona meadei (Schnabl, 1915) [Hackman 1981]
Spilogona megastoma (Boheman, 1866) [Hackman 1980]
Spilogona nigriventris (Zetterstedt, 1845) [Hackman 1980]
Spilogona nitidicauda (Schnabl, 1911) [Haarto and Winqvist 2014]
Spilogona novemmaculata (Zetterstedt, 1860) [Hackman 1980]
Spilogona opaca (Schnabl, 1915) [Hackman 1980]
Spilogona pacifica (Meigen, 1826)
= *S. vana* (Zetterstedt, 1845) [Hackman 1980]
Spilogona palmeni (Ringdahl, 1935) [Hackman 1980, Ringdahl 1935]
Spilogona pseudodispar (Frey, 1915) [Hackman 1980]
= *S. spinitibia* (Ringdahl, 1918)
Spilogona pusilla (Huckett, 1932) [Kahanpää 2013d]
Spilogona quinquelineata (Zetterstedt, 1838) [Hackman 1980]
Spilogona semiglobosa (Ringdahl, 1916) [Kahanpää 2013d]
Spilogona sororcula (Zetterstedt, 1845) [Hackman 1980]
= *S. zetterstedti* (Ringdahl, 1918) preocc.
Spilogona spectabilis (Tiensuu, 1938) [Kaisila 1952]
Spilogona surda (Zetterstedt, 1845) [Hackman 1980]
Spilogona tenuis Hennig, 1959 [Haarto and Winqvist 2014]
Spilogona tornensis (Ringdahl, 1926) [Hanski and Nuorteva 1975]
Spilogona triangulifera (Zetterstedt, 1838) [Haarto and Winqvist 2014]
Spilogona trianguligera (Zetterstedt, 1838) [Hackman 1980]
= *S. insularis* (Collin, 1921)
Spilogona trigonata (Zetterstedt, 1838) [Kaisila 1960, Hackman 1980]
Spilogona tundrae (Schnabl, 1915) [Hackman 1980, Haarto 2009]
= *S. macropyga* (Frey, 1915)
Spilogona tundrica (Schnabl & Dziedzicki, 1911) [Hackman 1980]
Spilogona varsaviensis (Schnabl & Dziedzicki, 1911) [Hackman 1980]
= *S. glauca* (Stein, 1916)

Spilogona veterrima (Zetterstedt, 1845) [Hackman 1980]
VILLENEUVIA Schnabl & Dziedzicki, 1911
= ***Spilogona*** error
Villeneuvia aestuum (Villeneuve, 1902) [Hackman 1980]
AZELIINAE Robineau-Desvoidy, 1830
tribe Azeliini Robineau-Desvoidy, 1830
AZELIA Robineau-Desvoidy, 1830
Azelia aterrima (Meigen, 1826) [Hackman 1980]
Azelia cilipes (Haliday, 1838) [Hackman 1980]
Azelia gibbera (Meigen, 1826) [Hackman 1980]
Azelia monodactyla Loew, 1874 [Pont 1989]
Azelia nebulosa Robineau-Desvoidy, 1830 [Hackman 1980]
= *A. macquarti* (Staeger in Schiødte, 1843) [Hackman 1980]
Azelia trigonica Hennig, 1956 [Pont 1989]
= *A. nuda* Hennig, 1956
Azelia triquetra (Wiedemann, 1817) [Hackman 1980]
Azelia zetterstedtii Rondani, 1866 [Hackman 1980]
DRYMEIA Meigen, 1826
= ***Pogonomyia*** Rondani, 1871
= ***Trichopticoides*** Ringdahl, 1931
Drymeia hamata (Fallén, 1823) [Hackman 1980]
Drymeia tetra (Meigen, 1826) [Hackman 1980]
Drymeia vicana (Harris, 1780) [Hackman 1980]
= *D. decolor* (Fallén, 1824) [Hackman 1980]
HYDROTAEA Robineau-Desvoidy, 1830
= ***Ophyra*** Robineau-Desvoidy, 1830
= ***Lasiops*** Meigen, 1838
Hydrotaea aenescens (Wiedemann, 1830) [Haarto and Mukkala 2013]
Hydrotaea albipuncta (Zetterstedt, 1845) [Hackman 1980]
Hydrotaea anxia (Zetterstedt, 1838)
= *H. bispinosa* (Zetterstedt, 1845) [Hackman 1980]
Hydrotaea armipes (Fallén, 1825)
= *H. occulta* (Meigen, 1826) [Hackman 1980]
Hydrotaea basdeni Collin, 1939 [Haarto and Winqvist 2014]
Hydrotaea borussica Stein, 1899 [Kahanpää 2013d]
Hydrotaea cyrtoneurina (Zetterstedt, 1845) [Haarto and Winqvist 2014]
Hydrotaea dentipes (Fabricius, 1805) [Hackman 1980]
Hydrotaea diabolus (Harrris, 1780)
= *H. ciliata* (Fabricius, 1794) preocc.
= *H. bimaculata* (Meigen, 1826) [Hackman 1980]
Hydrotaea floccosa Macquart, 1835
= *H. armipes* auct. nec (Fallén, 1825) [Hackman 1980]
Hydrotaea ignava (Harrris, 1780)
= *H. leucostoma* (Wiedemann, 1817) [Hackman 1980]
Hydrotaea irritans (Fallén, 1823) [Hackman 1980]
Hydrotaea meridionalis Portschinsky, 1882
Hydrotaea meteorica (Linnaeus, 1758) [Hackman 1980]
Hydrotaea militaris (Meigen, 1826) [Hackman 1980]
Hydrotaea nidicola Malloch, 1925 [Tiensuu 1935a, Hackman 1980]
Hydrotaea palaestrica (Meigen, 1826) [Hedström 1964, Hackman 1980]

Hydrotaea pandellei Stein, 1899 [Hackman 1980]
Hydrotaea parva Meade, 1889 [Hackman 1980]
Hydrotaea pellucens Portschinsky, 1879 [Hackman 1980]
Hydrotaea pilipes Stein, 1903 [Hackman 1980]
Hydrotaea pilitibia Stein, 1916 [Hackman 1980]
Hydrotaea ringdahli Stein, 1916 [Hackman 1980]
Hydrotaea scambus (Zetterstedt, 1838) [Hackman 1980]
Hydrotaea similis Meade, 1887 [Hackman 1980]
Hydrotaea tuberculata Rondani, 1866 [Hackman 1980]
Hydrotaea velutina Robineau-Desvoidy, 1830 [Hackman 1980]
POTAMIA Robineau-Desvoidy, 1830
= ***Dendrophaonia*** Malloch, 1923
Potamia littoralis Robineau-Desvoidy, 1830
= *P. querceti* (Bouché, 1834) [Hackman 1980]
THRICOPS Rondani, 1856
= ***Alleostylus*** Schnabl, 1888
Thricops aculeipes (Zetterstedt, 1838) [Hackman 1980]
Thricops albibasalis (Zetterstedt, 1849) [Hackman 1980]
= *T. sudeticus* auct. nec (Schnabl, 1888)
Thricops coquilletti (Malloch, 1920) [Hedström and Nuorteva 1971]
Thricops cunctans (Meigen, 1826)
= *T. hirsutulus* auct. nec (Zetterstedt, 1838) [Hackman 1980]
Thricops diaphanus (Wiedemann, 1817) [Hackman 1980]
Thricops foveolatus (Zetterstedt, 1845) [Hackman 1980]
Thricops genarum (Zetterstedt, 1838)
= *T. sundewalli* (Zetterstedt, 1845) [Hackman 1980]
Thricops hirtulus (Zetterstedt, 1838) [Hackman 1980]
= *T. subrostratus* (Zetterstedt, 1845)
Thricops innocuus (Zetterstedt, 1838) [Hackman 1980]
Thricops lividiventris (Zetterstedt, 1845) [Hackman 1980]
Thricops longipes (Zetterstedt, 1845) [Hackman 1980]
Thricops nigrifrons (Robineau-Desvoidy, 1830) [Hackman 1980]
Thricops nigritellus (Zetterstedt, 1838) [Hackman 1980]
Thricops rostratus (Meade, 1882) [Haarto and Winqvist 2014]
Thricops rufisquamus (Schnabl, 1915) [Hackman 1980]
= *T. penicillatus* (Ringdahl, 1926)
Thricops semicinereus (Wiedemann, 1817) [Hackman 1980]
Thricops separ (Zetterstedt, 1845) [Hackman 1980]
Thricops simplex (Wiedemann, 1817) [Hackman 1980]
tribe Reinwardtiini Brauer & Bergenstamm, 1889
MUSCINA Robineau-Desvoidy, 1830
Muscina angustifrons (Loew, 1858) [Flinck and Kahanpää 2013]
Muscina levida (Harris, 1780)
= *M. assimilis* (Fallén, 1823) [Hackman 1980]
Muscina pascuorum (Meigen, 1826) [Hackman 1980]
Muscina prolapsa (Harris, 1780)
= *M. pabulorum* (Fallén, 1817) [Hackman 1980]
Muscina stabulans (Fallén, 1817) [Hackman 1980]
MUSCINAE Latreille, 1802
tribe Muscini Latreille, 1802

EUDASYPHORA Townsend, 1911
= ***Dasyphora*** error
Eudasyphora cyanicolor (Zetterstedt, 1845) [Hackman 1980]
Eudasyphora zimini (Hennig, 1963) [Hackman 1980]
MESEMBRINA Meigen, 1826
= ***Hypodermodes*** Townsend, 1912
Mesembrina intermedia Zetterstedt, 1848? [Hackman 1980]
Mesembrina meridiana (Linnaeus, 1758) [Hackman 1980]
Mesembrina mystacea (Linnaeus, 1758) [Hackman 1980]
Mesembrina resplendens Wahlberg, 1844 [Haarto and Winqvist 2014]
MORELLIA Robineau-Desvoidy, 1830
sg. ***Morellia*** Robineau-Desvoidy, 1830
Morellia aenescens Robineau-Desvoidy, 1830 [Hackman 1980]
Morellia hortorum (Fallén, 1817) [Hackman 1980]
Morellia podagrica (Loew, 1857) [Hackman 1980]
sg. ***Ziminiella*** Nihei & Carvalho, 2007
Morellia simplex (Loew, 1857) [Hackman 1980]
MUSCA Linnaeus, 1758
Musca autumnalis De Geer, 1776 [Hackman 1980]
= *M. corvina* Fabricius, 1781
Musca domestica Linnaeus, 1758 [Hackman 1980]
Musca tempestiva Fallén, 1817 [Hackman 1980]
NEOMYIA Walker, 1859
= ***Orthellia*** Robineau-Desvoidy, 1863
Neomyia cornicina (Fabricius, 1781)
= *N. caesarion* (Meigen, 1826) [Hackman 1980]
= *N. fennica* (Frey, 1909)
Neomyia viridescens (Robineau-Desvoidy, 1830)
= *N. cornicina* auct. nec (Fabricius, 1781) [Hackman 1980]
POLIETES Rondani, 1866
Polietes domitor (Harris, 1780)
= *P. albolineatus* (Fallén, 1823) [Hackman 1980]
Polietes lardarius (Fabricius, 1781) [Hackman 1980]
Polietes nigrolimbatus (von Bonsdorff, 1866) [Hackman 1980]
Polietes steinii (Ringdahl, 1913) [Hedström 1968]
PYRELLIA Robineau-Desvoidy, 1830
Pyrellia vivida Robineau-Desvoidy, 1830
= *P. cadaverina* auct. nec (Linnaeus, 1758) [Hackman 1980]
tribe Stomoxyini Meigen, 1824
HAEMATOBIA Le Peletier & Serville, 1828
= ***Lyperosia*** Rondani, 1856
Haematobia irritans (Linnaeus, 1758) [Hackman 1980]
HAEMATOBOSCA Bezzi, 1907
Haematobosca alcis Snow, 1891
= *H. crassipalpis* (Ringdahl, 1920)
Haematobosca stimulans (Meigen, 1824) [Hackman 1980]
STOMOXYS Geoffroy, 1762
Stomoxys calcitrans (Linnaeus, 1758) [Hackman 1980]
MYDAEINAE Verrall, 1888
GRAPHOMYA Robineau-Desvoidy, 1830

Graphomya maculata (Scopoli, 1763) [Hackman 1980]
Graphomya minor Robineau-Desvoidy, 1830 [Haarto and Mukkala 2013]
GYMNODIA Robineau-Desvoidy, 1863
= ***Brontaea*** Kowarz, 1873
Gymnodia humilis (Zetterstedt, 1860) [Hackman 1980]
HEBECNEMA Schnabl, 1889
Hebecnema fumosa (Meigen, 1826) [Hackman 1980]
Hebecnema nigra (Robineau-Desvoidy, 1830)
= *H. vespertina* auct. nec (Fallén, 1823) [Hackman 1980]
Hebecnema nigricolor (Fallén, 1825) [Hackman 1980]
Hebecnema umbratica (Meigen, 1826) [Hackman 1980]
Hebecnema vespertina (Fallén, 1823)
= *H. affinis* Malloch, 1921 [Hackman 1980]
MYDAEA Robineau-Desvoidy, 1830
Mydaea affinis Meade, 1891
= *M. discimana* Malloch, 1920 [Hackman 1980]
Mydaea ancilla (Meigen, 1826) [Hackman 1980]
Mydaea anicula (Zetterstedt, 1860) [Hackman 1980]
Mydaea corni Scopoli, 1763
= *M. pagana* (Fabricius, 1794) preocc.
= *M. scutellaris* Robineau-Desvoidy, 1830 [Hackman 1980]
Mydaea deserta (Zetterstedt, 1845) [Hackman 1980]
Mydaea detrita (Zetterstedt, 1845)
= *M. electa* (Zetterstedt, 1860) [Hackman 1980]
Mydaea humeralis Robineau-Desvoidy, 1830
= *M. tincta* (Zetterstedt, 1845) [Hackman 1980]
Mydaea nebulosa (Stein, 1893) [Haarto and Winqvist 2014]
Mydaea obscurella Malloch, 1921
= *M. bengtssoni* Ringdahl, 1924 [Hackman 1980]
Mydaea orthonevra (Macquart, 1835)
= *M. detrita* auct. nec (Zetterstedt, 1845) [Hackman 1980]
Mydaea palpalis Stein, 1916 [Hedström and Nuorteva 1971]
Mydaea setifemur Ringdahl, 1924 [Hackman 1980]
Mydaea sootryeni Ringdahl, 1928 [Hackman 1980]
Mydaea urbana (Meigen, 1826) [Hackman 1980]
MYOSPILA Rondani, 1856
Myospila bimaculata (Macquart, 1834) [Pont 1990]
Myospila meditabunda (Fabricius, 1781) [Hackman 1980]
OPSOLASIA Coquillett, 1910
Opsolasia orichalcea (Zetterstedt, 1849) [Hackman 1980]
PHAONIINAE Malloch, 1917
tribe Phaoniini Malloch, 1917
HELINA Robineau-Desvoidy, 1830
Helina abdominalis (Zetterstedt, 1846) [Hackman 1980]
Helina allotalla (Meigen, 1830) [Hackman 1980]
Helina annosa (Zetterstedt, 1838) [Hackman 1980]
Helina atricolor (Fallén, 1825)
= *H. denudata* (Zetterstedt, 1845)
= *H. denutata* misspelling[Hackman 1980]
Helina bohemani (Ringdahl, 1916) [Hackman 1980]

Helina celsa (Harris, 1780)
= *H. quadrimaculata* (Fallén, 1823) preocc.
= *H. quadrimaculella* Hennig, 1957 [Hackman 1980]
? *Helina ciliata* Karl, 1929 [Hackman 1980]
Helina ciliatocosta (Zetterstedt, 1845)
= *H. ciliatocostata* misspelling[Hackman 1980]
Helina cilipes (Schnabl, 1902) [Hackman 1980]
Helina cinerella (van der Wulp, 1867)
= *H. vanderwulpi* (Schnabl, 1888) [Hackman 1980]
= *H. calceata* misid.[Tiensuu 1935a, Hackman 1980]
Helina confinis (Fallén, 1825)
= *H. anceps* (Zetterstedt, 1838) [Hackman 1980]
Helina consimilis (Fallén, 1825) [Hackman 1980]
Helina cothurnata (Rondani, 1866) [Hackman 1980]
= *H. obscuripes* auct. nec (Zetterstedt, 1845)
Helina daicles (Walker, 1849)
= *H. binotata* (Zetterstedt, 1845) [Hackman 1980]
Helina depuncta (Fallén, 1825) [Hackman 1980]
Helina evecta (Harris, 1780)
= *H. lucorum* (Fallén, 1823) preocc.
= *H. laetifica* (Robineau-Desvoidy, 1830) [Hackman 1980]
Helina flavisquama (Zetterstedt, 1849) [Hackman 1980]
Helina fratercula (Zetterstedt, 1845) [Hackman 1980]
Helina fulvisquama (Zetterstedt, 1845) [Hackman 1980]
Helina impuncta (Fallén, 1825) [Hackman 1980]
Helina latitarsis Ringdahl, 1924 [Hackman 1980]
Helina laxifrons (Zetterstedt, 1860) [Hackman 1980]
Helina longicornis (Zetterstedt, 1838) [Hackman 1981]
Helina luteisquama (Zetterstedt, 1845) [Hackman 1980]
Helina maculipennis (Zetterstedt, 1845) [Hackman 1980]
= *H. obscuripes* (Zetterstedt, 1845)
Helina obscurata (Meigen, 1826) [Hackman 1980]
Helina pertusa (Meigen, 1826) [Kahanpää 2013d]
Helina protuberans (Zetterstedt, 1845) [Hackman 1980]
Helina pubiseta (Zetterstedt, 1845) [Hackman 1980]
Helina quadrinotata (Meigen, 1826) [Hackman 1980]
Helina quadrum (Fabricius, 1805) [Hackman 1980]
Helina reversio (Harris, 1780)
= *H. duplicata* (Meigen, 1826) [Hackman 1980]
= *H. duplaris* auct. nec (Zetterstedt, 1845) [Kaisila 1960]
= *H. communis* (Robineau-Desvoidy, 1830)
Helina setiventris Ringdahl, 1924 [Flinck and Kahanpää 2013]
Helina sexmaculata (Preyssler, 1791)
= *H. uliginosa* (Fallén, 1825) preocc.
= *H. punctata* (Robineau-Desvoidy, 1830) [Hackman 1980]
Helina spinicosta (Zetterstedt, 1845) [Hackman 1980]
Helina squalens (Zetterstedt, 1838) [Hackman 1980]
= *H. borealis* (Zetterstedt, 1838)
Helina subvittata (Séguy, 1923)
= *H. rothi* Ringdahl, 1939 [Hackman 1980]

= *H. marmorata* auct. nec (Zetterstedt, 1860)
Helina tetrastigma (Meigen, 1826)
= *H. flagripes* (Rondani, 1866) [Hackman 1980]
Helina trivittata (Zetterstedt, 1860)
= *H. atripes* (Meade, 1889) [Hackman 1980]
Helina veterana (Zetterstedt, 1838) [Hanski and Nuorteva 1975]
Helina vicina (Czerny, 1900) [Hackman 1980]
= *H. suecica* Ringdahl, 1924
LOPHOSCELES Ringdahl, 1922
Lophosceles cinereiventris (Zetterstedt, 1845) [Hackman 1980]
= *L. cristata* (Zetterstedt, 1845)
Lophosceles frenatus (Holmgren, 1872) [Hackman 1980]
Lophosceles hians (Zetterstedt, 1838) [Hackman 1980]
Lophosceles mutatus (Fallén, 1825) [Hackman 1980]
PHAONIA Robineau-Desvoidy, 1830
= ***Wahlgrenia*** Ringdahl, 1929
= ***Dialytina*** Ringdahl, 1945
Phaonia aeneiventris (Zetterstedt, 1845) [Hackman 1980]
= *P. cinctinervis* (Zetterstedt, 1860)
Phaonia alpicola (Zetterstedt, 1845) [Hackman 1980]
Phaonia amabilis (Meigen, 1826) [Haarto 2009]
Phaonia amicula Villeneuve, 1922
Phaonia angelicae (Scopoli, 1763)
= *P. basalis* (Zetterstedt, 1838) [Hackman 1980]
Phaonia angulicornis (Zetterstedt, 1838)
= *P. erinacea* (Fallén, 1824) [Hackman 1980]
Phaonia apicalis Stein, 1914 [Hackman 1980]
Phaonia atriceps (Loew, 1858) [Haarto and Winqvist 2014]
Phaonia atrocyanea Ringdahl, 1916 [Haarto 2009]
Phaonia canescens Stein, 1916 [Kahanpää 2013d]
Phaonia consobrina (Zetterstedt, 1838) [Hackman 1980]
Phaonia czernyi Hennig, 1963 [Hackman 1980]
= *P. steinii* Czerny, 1900 preocc.
Phaonia errans (Meigen, 1826) [Hackman 1980]
= *P. erratica* (Fallén, 1825) preocc.
Phaonia erronea (Schnabl, 1887) [Hackman 1980]
Phaonia falleni (Michelsen, 1977)
= *P. vagans* (Fallén, 1825) preocc.[Hackman 1980]
Phaonia fugax Tiensuu, 1946 [Hackman 1981]
Phaonia fuscata (Fallén, 1825) [Hackman 1980]
Phaonia gobertii (Mik, 1881) [Kahanpää 2013d]
Phaonia gracilis Stein, 1916 [Hackman 1981]
Phaonia grandaeva (Zetterstedt, 1845) [Hackman 1980]
Phaonia halterata (Stein, 1893) [Hackman 1980, Tiensuu 1935a]
Phaonia hybrida (Schnabl, 1888) [Hackman 1980]
Phaonia incana (Wiedemann, 1817) [Hackman 1980]
Phaonia jaroschewskii (Schnabl, 1888)
= *P. crinipes* Stein, 1913 [Hackman 1980]
Phaonia kowarzii (Schnabl, 1886) [Hackman 1980]
= *P. fulvicornis* Tiensuu, 1936 [Tiensuu 1935a]

Phaonia laeta (Fallén, 1823) [Winqvist 2011]
Phaonia latipalpis Schnabl, 1911 [Haarto and Winqvist 2014]
= *P. umbraticola* d'Assis-Fonseca, 1957
Phaonia longicornis Stein, 1916 [Haarto and Winqvist 2014]
Phaonia lugubris (Meigen, 1826)
= *P. morio* (Zetterstedt, 1845) [Hackman 1980]
Phaonia magnicornis (Zetterstedt, 1845) [Hackman 1980]
Phaonia meigeni Pont, 1986
= *P. lugubris* auct. nec (Meigen, 1826) [Hackman 1980]
Phaonia mystica (Meigen, 1826)
= *P. vittifera* (Zetterstedt, 1845) [Hackman 1980]
Phaonia nymphaearum (Robineau-Desvoidy, 1830)
= *P. nitida* (Macquart, 1835) [Hackman 1980]
Phaonia pallida (Fabricius, 1787) [Hackman 1980]
Phaonia pallidisquama (Zetterstedt, 1849) [Hackman 1980]
Phaonia palpata (Stein, 1897) [Tiensuu 1964, Hackman 1980]
Phaonia perdita (Meigen, 1830) [Hackman 1980]
Phaonia pratensis (Robineau-Desvoidy, 1830) [Hackman 1980]
= *P. laeta* auct. nec (Fallén, 1923)
Phaonia rufipalpis (Macquart, 1835) [Hackman 1981]
Phaonia rufiventris (Scopoli, 1763)
= *P. populi* (Meigen, 1826) [Hackman 1980]
Phaonia serva (Meigen, 1826) [Hackman 1980]
Phaonia steinii (Strobl, 1898)
Phaonia subfuscinervis (Zetterstedt, 1838) [Hackman 1980]
Phaonia subventa (Harris, 1780)
= *P. variegata* (Meigen, 1826) [Hackman 1980]
Phaonia taigensis Zinovjev, 1987 [Zinovjev 1987]
Phaonia tiefii Schnabl, 1888 [Hackman 1981]
Phaonia trimaculata (Bouché) [Hackman 1980]
= *P. servaeformis* Ringdahl, 1916
Phaonia tuguriorum (Scopoli, 1763)
= *P. signata* (Meigen, 1826) [Hackman 1980]
Phaonia valida (Harris, 1780)
= *P. viarum* (Robineau-Desvoidy, 1830) [Hackman 1980]
= *P. erratica* auct. nec (Fallén, 1825)
Phaonia villana Robineau-Desvoidy, 1830
= *P. mystica* auct. nec (Meigen, 1826) [Hackman 1980, Tiensuu 1935a]
Phaonia vivida (Rondani, 1870) [Hackman 1980]
= *P. austriaca* (Czerny, 1900)
Phaonia wahlbergi Ringdahl, 1930 [Hackman 1980]
Phaonia zugmayeriae (Schnabl, 1888) [Hackman 1980]
? = *P. humeralis* (Zetterstedt, 1845)
= *P. humerella* (Stein, 1900)

Excluded species

Coenosia femoralis (Robineau-Desvoidy, 1830)
Hydrotaea glabricula (Fallén, 1825)

Limnophora pollinifrons Stein, 1916
Phaonia bitincta (Rondani, 1866) [Tiensuu 1935a, Hackman 1980]
Spilogona acrostichalis (Stein, 1916) [Pont 1986]
Spilogona norvegica (Ringdahl, 1932) [Pont 1986]
Spilogona obscuripennis (Stein, 1916)
Spilogona septemnotata (Zetterstedt, 1845) [Hackman 1980]
Spilogona setigera (Stein, 1907)
Spilogona spininervis (Villeneuve, 1922)

CALLIPHORIDAE Brauer & Bergenstamm, 1889

Supporting references for *Pohjoismäki J, Kahanpää J (2014) Checklist of the superfamilies Oestroidea and Hippoboscoidea of Finland (Insecta, Diptera). In: Kahanpää J, Salmela J (Eds) Checklist of the Diptera of Finland. ZooKeys @@: @-@. doi: 10.3897/zookeys.??.7252*

CALLIPHORINAE Brauer & Bergenstamm, 1889
BELLARDIA Robineau-Desvoidy, 1863
= ***Pseudonesia*** Villeneuve, 1920
Bellardia bayeri (Jacentkovsky, 1937) [Flinck and Kahanpää 2013]
Bellardia pandia (Walker, 1849)
= *B. biseta* (Kramer, 1917) [Hackman 1980]
Bellardia pubicornis (Zetterstedt, 1838)
= *B. puberula* (Zetterstedt, 1838) [Hackman 1980]
Bellardia stricta (Villeneuve, 1926) [Hackman 1980]
= *B. polita* auct. nec (Mik, 1883) [Hackman 1980]
Bellardia viarum (Robineau-Desvoidy, 1830)
= *B. pusilla* (Meigen, 1826) [Hackman 1980]
Bellardia vulgaris (Robineau-Desvoidy, 1830)
= *B. agilis* (Meigen, 1826) preocc.
CALLIPHORA Robineau-Desvoidy, 1830
= ***Steringomyia*** Pokorny, 1889
= ***Acrophaga*** Brauer & Bergenstamm, 1891
= ***Abonesia*** Villeneuve, 1927
= ***Acronesia*** Hall, 1948
Calliphora genarum (Zetterstedt, 1838) [Hackman 1980]
= *C. alpina* (Zetterstedt, 1845) [Nuorteva 1963, Hackman 1980]
Calliphora loewi Enderlein, 1903 [Hackman 1980]
Calliphora stelviana (Brauer & Bergenstamm, 1891)
Calliphora subalpina (Ringdahl, 1931) [Hackman 1980]
Calliphora uralensis Villeneuve, 1922 [Hackman 1980]
Calliphora vicina Robineau-Desvoidy, 1830 [Hackman 1980]
= *C. erythrocephala* (Meigen, 1826) preocc.
Calliphora vomitoria (Linnaeus, 1758) [Hackman 1980]
CYNOMYA Robineau-Desvoidy, 1830
Cynomya mortuorum (Linnaeus, 1761) [Hackman 1980]
ONESIA Robineau-Desvoidy, 1830
Onesia floralis Robineau-Desvoidy, 1830 [Haarto 1995]
CHRYSOMYINAE Shannon, 1923
PROTOCALLIPHORA Hough, 1899
Protocalliphora azurea (Fallén, 1817) [Hackman 1980]
Protocalliphora nuortevai Grunin, 1972 [Grunin 1972, Hackman 1980]
Protocalliphora peusi Gregor & Povolny, 1959
Protocalliphora proxima Grunin, 1966 [Hackman 1980]
Protocalliphora rognesi Thompson & Pont, 1993
= *P. chrysorrhoea* Meigen, 1826 preocc.[Hackman 1980]
PROTOPHORMIA Townsend, 1908
= ***Boreellus*** Aldrich & Schannon, 1923
Protophormia atriceps (Zetterstedt, 1845) [Nuorteva 1964, Hackman 1980]

Protophormia terraenovae (Robineau-Desvoidy, 1830) [Hackman 1980]
TRYPOCALLIPHORA Peus, 1960
Trypocalliphora braueri (Hendel, 1901)
= *T. lindneri* Peus, 1960 [Hackman 1980]
HELICOBOSCINAE Verves, 1980
EURYCHAETA Brauer & Bergenstamm, 1891
= ***Helicobosca*** Bezzi, 1906
Eurychaeta palpalis (Robineau-Desvoidy, 1830) [Hackman 1980]
LUCILIINAE Shannon, 1923
LUCILIA Robineau-Desvoidy, 1830
Lucilia ampullacea Villeneuve, 1922
Lucilia bufonivora Moniez, 1876 [Hackman 1980]
Lucilia caesar (Linnaeus, 1758) [Hackman 1980]
Lucilia illustris (Meigen, 1826) [Hackman 1980]
Lucilia magnicornis (Siebke, 1863)
= *L. fuscipalpis* (Zetterstedt, 1845) [Hackman 1980]
Lucilia richardsi Collin, 1926 [Hackman 1980]
Lucilia sericata (Meigen, 1826) [Hackman 1980]
Lucilia silvarum (Meigen, 1826) [Hackman 1980]
MELANOMYINAE Townsend, 1919
ANGIONEURA Brauer & Bergenstamm, 1893
Angioneura acerba (Meigen, 1838) [Hackman 1980]
MELANOMYA Rondani, 1856
Melanomya nana (Meigen, 1826) [Hackman 1980]
MELINDA Robineau-Desvoidy, 1830
Melinda gentilis Robineau-Desvoidy, 1830 [Haarto 1995]
= *M. coerulea* (Meigen, 1826) preocc.[Valle 1938]
= *M. cognata* (Meigen, 1830) [Hackman 1980]
Melinda viridicyanea (Robineau-Desvoidy, 1830)
POLLENIINAE Brauer & Bergenstamm, 1889
MORINIA Robineau-Desvoidy, 1830
Morinia doronici (Scopoli, 1763)
= *M. melanoptera* (Fallén, 1817) [Hackman 1980]
= *Angioneura fimbriata* misid.[Rognes 1991]
POLLENIA Robineau-Desvoidy, 1830
Pollenia amentaria (Scopoli, 1763)
= *P. vespillo* auct. nec (Fabricius, 1794) [Hackman 1980]
Pollenia angustigena Wainwright, 1940 [Rognes 1991]
Pollenia griseotomentosa (Jacentkowský, 1944) [Rognes 1991]
Pollenia hungarica Rognes, 1987 [Rognes 1991]
Pollenia labialis Robineau-Desvoidy, 1863 [Rognes 1991]
= *P. excarinata* Wainwright, 1940 [Hackman 1963]
= *P. intermedia* misid.[Nuorteva 1960, Hackman 1980]
Pollenia pediculata Macquart, 1834 [Rognes 1991]
Pollenia rudis (Fabricius, 1794) [Hackman 1980]
= *P. varia* (Meigen, 1826) [Hackman 1980]
Pollenia vagabunda (Meigen, 1826) [Hackman 1980]

RHINIIDAE Brauer & Bergenstamm, 1889

Supporting references for *Pohjoismäki J, Kahanpää J (2014) Checklist of the superfamilies Oestroidea and Hippoboscoidea of Finland (Insecta, Diptera). In: Kahanpää J, Salmela J (Eds) Checklist of the Diptera of Finland. ZooKeys @@: @-@. doi: 10.3897/zookeys.??.7252*

STOMORHINA Rondani, 1861
? *Stomorhina lunata* (Fabricius, 1805) [Hackman 1980]

SARCOPHAGIDAE Macquart, 1834

Supporting references for *Pohjoismäki J, Kahanpää J (2014) Checklist of the superfamilies Oestroidea and Hippoboscoidea of Finland (Insecta, Diptera). In: Kahanpää J, Salmela J (Eds) Checklist of the Diptera of Finland. ZooKeys @@: @-@. doi: 10.3897/zookeys.??.7252*

MILTOGRAMMINAE Lioy, 1864
AMOBIA Robineau-Desvoidy, 1830
Amobia oculata (Zetterstedt, 1844) [Pape 1987a]
= *A. signata* misid.[Hackman 1980, Pape 1987a]
Amobia signata (Meigen, 1824) [Pape 1987b, Haarto 2000]
HILARELLA Rondani, 1856
Hilarella hilarella (Zetterstedt, 1844) [Pape 1987a]
MACRONYCHIA Rondani, 1859
= ***Macronichia*** orig. misspelling
sg. ***Macronychia*** Rondani, 1859
Macronychia striginervis (Zetterstedt, 1838) [Hackman 1980]
sg. ***Moschusa*** Robineau-Desvoidy, 1863
Macronychia agrestis (Fallén, 1820) [Hackman 1980]
Macronychia griseola (Fallén, 1820) [Hackman 1980]
Macronychia polyodon (Meigen, 1824) [Hackman 1980]
METOPIA Meigen, 1803
Metopia argentata Macquart 1850
= *M. roserii* Rondani, 1859 [Pape 1987b]
= *M. stackelbergi* Rohdendorf, 1955 [Hackman 1980]
Metopia argyrocephala (Meigen, 1824) [Hackman 1980]
= *M. leucocephala* (Rossi, 1790) preocc.
Metopia campestris (Fallén, 1810) [Hackman 1980]
Metopia grandii Venturi, 1953 [Pape 1987a]
Metopia staegerii Rondani, 1859
= *M. rondaniana* Venturi, 1953 [Hackman 1980]
Metopia tshernovae Rohdendorf, 1955 [Hackman 1980]
MILTOGRAMMA Meigen, 1803
Miltogramma germari Meigen, 1824 [Pape 1987a]
Miltogramma iberica (Villeneuve, 1912) [Hackman 1980]
Miltogramma oestracea (Fallén, 1820) [Hackman 1980]
Miltogramma punctata Meigen, 1824 [Hackman 1980]
Miltogramma villeneuvei Verves, 1982 [Pape 1987a]
OEBALIA Robineau-Desvoidy, 1863
Oebalia cylindrica (Fallén, 1810) [Hackman 1980]
Oebalia minuta (Fallén, 1810) [Pape 1987a]
PHROSINELLA Robineau-Desvoidy, 1863
Phrosinella sannio (Zetterstedt, 1838) [Pape 1987b]
= *P. nasuta* misid.[Mee 1928, Hackman 1980, Pape 1987a]
PTERELLA Robineau-Desvoidy, 1830
Pterella grisea (Meigen, 1824) [Hackman 1980]
SENOTAINIA Macquart, 1846
Senotainia albifrons (Rondani, 1859) [Flinck and Kahanpää 2013]
Senotainia conica (Fallén, 1810) [Hackman 1980]

Senotainia puncticornis (Zetterstedt, 1859) [Pape 1987b]
= *S. imberbis* (Zetterstedt, 1838) preocc.[Hackman 1980]
= *S. crabronum* (Kramer, 1920)
TAXIGRAMMA Perris, 1852
Taxigramma elegantulum (Zetterstedt, 1844) [Hackman 1980]
Taxigramma heteroneurum (Meigen, 1830) [Hackman 1980]
PARAMACRONYCHIINAE Brauer & Bergenstamm, 1889
AGRIA Robineau-Desvoidy, 1830
Agria affinis (Fallén, 1817) [Hackman 1980]
= *A. punctata* Robineau-Desvoidy, 1830 [Pape 1987b]
Agria mamillata (Pandellé, 1896) [Hackman 1980]
ANGIOMETOPA Brauer & Bergenstamm, 1889
Angiometopa falleni Pape, 1986
= *A. ruralis* (Fallén, 1817) preocc.[Hackman 1980]
BRACHICOMA Rondani, 1856
Brachicoma devia (Fallén, 1820) [Hackman 1980]
Brachicoma borealis Ringdahl, 1932 [Ringdahl 1932a, Hackman 1980, Pape 1993]
SARCOPHAGINAE Macquart, 1834
BLAESOXIPHA Loew, 1861
sg. ***Blaesoxipha*** Loew, 1861
Blaesoxipha lapidosa Pape, 1994 [Pape 1994]
= *B. agerestis* auct. nec (Robineau-Desvoidy, 1863) [Pape 1987a]
= *B. campestris* auct. nec (Robineau-Desvoidy, 1863)
Blaesoxipha laticornis (Meigen, 1826) [Pape 1987b, Haarto and Winqvist 2014]
Blaesoxipha plumicornis (Zetterstedt, 1859) [Mee 1928, Pape 1987a]
= *B. laticornis* misid.[Hackman 1980, Pape 1987a]
= *B. gladiatrix* (Pandellé, 1896) [Mee 1928]
sg. ***Servaisia*** Robineau-Desvoidy, 1863
Blaesoxipha erythrura (Meigen, 1826) [Hackman 1980]
RAVINIA Robineau-Desvoidy, 1830
Ravinia pernix (Harris, 1780) [Hackman 1980]
= *R. striata* (Fabricius, 1794) preocc.[Hackman 1980]
SARCOPHAGA Meigen, 1826
sg. ***Bellieriomima*** Rohdendorf, 1937
Sarcophaga subulata Pandellé, 1896 [Hackman 1980]
sg. ***Discacheata*** Enderlein, 1928
Sarcophaga pumila Meigen, 1826 [Hackman 1980]
sg. ***Helicophagella*** Enderlein, 1928
Sarcophaga crassimargo Pandellé, 1896 [Hackman 1980]
Sarcophaga melanura Meigen, 1826 [Hackman 1980]
Sarcophaga rosellei Böttcher, 1912 [Flinck and Kahanpää 2013]
sg. ***Heteronychia*** Brauer & Bergenstamm, 1889
Sarcophaga depressifrons Zetterstedt, 1845 [Pape 1987b]
= *S. offuscata* auct. nec Meigen, 1826 [Hackman 1980]
Sarcophaga haemorrhoa Meigen, 1826 [Hackman 1980]
Sarcophaga proxima (Rondani, 1860) [Pape 1987a]
Sarcophaga vagans (Meigen, 1826) [Hackman 1980]
= *S. frenata* Pandellé, 1896
Sarcophaga vicina (Macquart, 1835) [Pape 1987a]
sg. ***Liosarcophaga*** Enderlein, 1928

Sarcophaga emdeni (Rohdendorff, 1969) [Pape 1987b]
= *S. teretirostris* auct. nec Pandellé, 1896 [Hackman 1980]
Sarcophaga portschinskyi Rohdendorf, 1937 [Hackman 1980]
= *S. tuberosa* misid.[Hackman 1980, Pape 1987a]
sg. ***Mehria*** Enderlein, 1928
Sarcophaga sexpunctata (Fabricius, 1805)
= *S. clathrata* (Meigen, 1826) [Hackman 1980]
Sarcophaga nemoralis Kramer, 1908 [Hackman 1980]
sg. ***Myorhina*** Robineau-Desvoidy, 1830
= ***Pierretia*** Robineau-Desvoidy, 1863
Sarcophaga socrus Rondani, 1860
= *S. rostrata* Pandellé, 1896 [Hackman 1980]
Sarcophaga villeneuvei Böttcher, 1912 [Hackman 1980]
sg. ***Pandelleisca*** Rohdendorf, 1937
Sarcophaga similis Meade, 1876 [Hackman 1980]
sg. ***Parasarcophaga*** Johnston & Tiegs, 1921
Sarcophaga albiceps Meigen, 1826 [Hackman 1980]
= *S. privigna* Rondani, 1860
sg. ***Robineauella*** Enderlein, 1928
Sarcophaga caerulescens Zetterstedt, 1838
= *S. scoparia* Pandellé, 1896 [Hackman 1980]
sg. ***Rosellea*** Rohdendorf, 1937
Sarcophaga aratrix Pandellé, 1896 [Hackman 1980]
sg. ***Sarcophaga*** sensu stricto
Sarcophaga carnaria (Linnaeus, 1758)
= *S. schulzi* Müller, 1922 [Hackman 1980]
= *S. lehmanni* auct. nec Müller, 1922 [Hackman 1980, Pape 1987a]
Sarcophaga lehmanni Müller, 1922 [Haarto and Winqvist 2014]
= *S. lasiostyla* auct. nec Macquart, 1843
= *S. cognata* Rondani, 1860 preocc.
Sarcophaga subvicina Rohdendorf, 1937 [Hackman 1980]
Sarcophaga variegata (Scopoli, 1763)
= *S. carnaria* auct. nec (Linnaeus, 1758) [Hackman 1980]
sg. ***Sarcotachinella*** Townsend, 1892
Sarcophaga sinuata Meigen, 1826 [Hackman 1980]
sg. ***Thyrsocnema*** Enderlein, 1928
Sarcophaga incisilobata Pandellé, 1896 [Hackman 1980]
Sarcophaga kentejana (Rohdendorf, 1937) [Pape 1987a]
= *S. lapponica* (Tiensuu, 1939)

Excluded species

Macronychia alpestris Rondani, 1865 [Pape 1987b, Verves and Khrokalo 2006]
Macronychia conica Robineau-Desvoidy, 1830 [Pape 1987a]
Sarcophaga agnata Rondani, 1860 [Hackman 1980, Pape 1987a] ?
Sarcophaga dissimilis Meigen, 1826 [Hackman 1980, Pape 1987a] ?
Sarcophaga granulata Kramer, 1908 [Verves 1986]
Sarcophila latifrons (Fallén, 1817) [Pape 1987a]

RHINOPHORIDAE Robineau-Desvoidy, 1863

Supporting references for *Pohjoismäki J, Kahanpää J (2014) Checklist of the superfamilies Oestroidea and Hippoboscoidea of Finland (Insecta, Diptera). In: Kahanpää J, Salmela J (Eds) Checklist of the Diptera of Finland. ZooKeys @@: @-@. doi: 10.3897/zookeys.??.7252*

MELANOPHORA Meigen, 1803
Melanophora roralis (Linnaeus, 1758) [Hackman 1980]
PAYKULLIA Robineau-Desvoidy, 1830
Paykullia brevicornis (Zetterstedt, 1844) [Haarto 2000]
Paykullia maculata (Fallén, 1815)
STEVENIA Robineau-Desvoidy, 1830
Stevenia atramentaria (Meigen, 1824) [Hackman 1980]
TRICOGENA Rondani, 1856
= ***Frauenfeldia*** Egger, 1865
Tricogena rubricosa (Meigen, 1824) [Hackman 1980]

TACHINIDAE Robineau-Desvoidy, 1830

Supporting references for *Pohjoismäki J, Kahanpää J (2014) Checklist of the superfamilies Oestroidea and Hippoboscoidea of Finland (Insecta, Diptera). In: Kahanpää J, Salmela J (Eds) Checklist of the Diptera of Finland. ZooKeys @@: @-@. doi: 10.3897/zookeys.??.7252*

EXORISTINAE Robineau-Desvoidy, 1863
tribe Exoristini Robineau-Desvoidy, 1863
EXORISTA Meigen, 1803
sg. ***Adenia*** Robineau-Desvoidy, 1863
Exorista mimula (Meigen, 1824)
Exorista rustica (Fallén, 1810) [Hackman 1980]
Exorista sp. A
sg. ***Exorista*** Meigen, 1803
Exorista fasciata (Fallén, 1820) [Hackman 1980]
Exorista larvarum (Linnaeus, 1758) [Hackman 1980]
sg. ***Podotachina*** Brauer & Bergenstamm, 1891
Exorista grandis (Zetterstedt, 1844) [Hackman 1980]
= *E. sorbillans* auct. nec Wiedemann, 1830
sg. ***Ptilotachina*** Brauer & Bergenstamm 1891
Exorista deligata Pandellé, 1896 [Hackman 1981]
CHETOGENA Rondani, 1856
Chetogena tschorsnigi Ziegler, 1999 [Pohjoismäki 2006]
DIPLOSTICHUS Brauer & Bergenstamm, 1889
Diplostichus janitrix (Hartig, 1838) [Hackman 1980]
PHOROCERA Brauer & Bergenstamm, 1889
Phorocera assimilis (Fallén, 1810)
Phorocera obscura (Fallén, 1810) [Hackman 1980]
PHORINIA Robineau-Desvoidy, 1830
Phorinia aurifrons Robineau-Desvoidy, 1830
BESSA Robineau-Desvoidy, 1830
Bessa selecta (Meigen, 1824) [Hackman 1980]
tribe Blondeliini Robineau-Desvoidy, 1863
BELIDA Robineau-Desvoidy, 1863
= ***Aporotachina*** Meade, 1894
Belida angelicae (Meigen, 1824) [Hackman 1980]
MEIGENIA Robineau-Desvoidy, 1830
Meigenia dorsalis (Meigen, 1824)
Meigenia mutabilis (Fallén, 1810) [Hackman 1980]
= *M. bisignata* (Meigen, 1824)
ZAIRA Robineau-Desvoidy, 1830
= ***Viviania*** Rondani, 1861
Zaira cinerea (Fallén, 1810) [Hackman 1980]
MEDINA Robineau-Desvoidy, 1830
Medina collaris (Fallén, 1820) [Hackman 1980]
Medina luctuosa (Meigen, 1824) [Hackman 1980]
Medina separata (Meigen, 1824)
POLICHETA Rondani, 1856
= ***Perichaeta*** preocc.

Policheta unicolor (Fallén, 1820) [Hackman 1980]
ISTOCHETA Rondany, 1859
= ***Hyperecteina*** Schiner, 1862
Istocheta longicornis (Fallén, 1810) [Hackman 1980]
STAUROCHAETA Brauer & Bergenstamm, 1889
Staurochaeta albocingulata (Fallén, 1820) [Hackman 1980]
ADMONTIA Brauer & Bergenstamm, 1889
= ***Trichopareia*** Brauer & Bergenstamm, 1889
Admontia blanda (Fallén, 1820) [Hackman 1980]
Admontia grandicornis (Zetterstedt, 1849) [Hackman 1980, 1981]
Admontia seria (Meigen, 1824) [Hackman 1980]
OSWALDIA Robineau-Desvoidy, 1863
Oswaldia muscaria (Fallén, 1810) [Hackman 1980]
Oswaldia eggeri (Brauer & Bergenstamm, 1889)
Oswaldia reducta (Villeneuve, 1930)
Oswaldia spectabilis (Meigen, 1824)
= *O. albisquama* (Zetterstedt, 1844) [Hackman 1980]
PARACRASPEDOTHRIX Villeneuve, 1919
Paracraspedothrix montivaga Villeneuve, 1919
LIGERIA Robineau-Desvoidy, 1863
Ligeria angusticornis (Loew, 1847) [Hackman 1980]
= *L. zetterstedti* (Ringdahl, 1945)
LIGERIELLA Mesnil, 1961
Ligeriella aristata (Villeneuce, 1911)
BLONDELIA Robineau-Desvoidy, 1830
Blondelia nigripes (Fallén, 1810) [Hackman 1980]
tribe Acemyini Brauer & Bergenstamm, 1889
ACEMYA Robineau-Desvoidy, 1830
Acemya acuticornis (Meigen, 1824) [Hackman 1980]
Acemya rufitibia (von Roser, 1840)
tribe Ethillini Mesnil, 1944
PARATRYPHERA Brauer & Bergenstamm, 1891
Paratryphera barbatula (Rondani, 1859)
Paratryphera bisetosa (Brauer & Bergenstamm, 1891)
tribe Winthemiini Townsend, 1913
RHAPHIOCHAETA Brauer & Bergenstamm, 1889
Rhaphiochaeta breviseta (Zetterstedt, 1838) [Hackman 1980]
SMIDTIA Robineau-Desvoidy, 1830
= ***Timavia*** Robineau-Desvoidy, 1863
Smidtia amoena (Meigen, 1824) [Hackman 1980]
Smidtia conspersa (Meigen, 1824)
WINTHEMIA Robineau-Desvoidy, 1830
Winthemia cruentata (Rondani, 1859)
Winthemia quadripustulata (Fabricius, 1794) [Hackman 1980]
Winthemia erythrura (Meigen, 1838) [Hackman 1980]
Winthemia venusta (Meigne, 1824)
NEMORILLA Rondani, 1856
Nemorilla floralis (Fallen, 1810)
Nemorilla maculosa (Meigen, 1824) [Haarto et al. 2002]
= *N. floralis* misid.[Hackman 1980]

tribe Eryciini Robineau-Desvoidy, 1830

APLOMYA Robineau-Desvoidy, 1830

Aplomya confinis (Fallén, 1820) [Hackman 1980]

PHEBELLIA Robineau-Desvoidy, 1846

Phebellia clavellariae (Brauer & Bergenstamm, 1891)

Phebellia glauca (Meigen, 1824) [Hackman 1980]

Phebellia glaucoides Herting, 1961

Phebellia glirina (Rondani, 1859)

Phebellia margaretae Bergström, 2005 [Bergström 2005]

Phebellia nigripalpis (Robineau-Desvoidy, 1847)

= *P. fuscipennis* (Robineau-Desvoidy, 1830) [Hackman 1980]

Phebellia pauciseta (Villeneuve, 1908)

Phebellia strigifrons (Zetterstedt, 1838)

Phebellia stulta (Zetterstedt, 1844)

Phebellia triseta (Pandellé, 1896) [Pohjoismäki 2006]

Phebellia vicina Wainwright, 1940

Phebellia villica (Zetterstedt, 1838)

Phebellia sp. A

NILEA Robineau-Desvoidy, 1863

Nilea hortulana (Meigen, 1824) [Hackman 1980]

Nilea innoxia Robineau-Desvoidy, 1863 [Hackman 1981]

Nilea rufiscutellaris (Zetterstedt, 1859)

TLEPHUSA Robineau-Desvoidy, 1863

Tlephusa cincinna (Meigen, 1859) [Tschorsnig and Herting 1994]

= *T. diligens* auct. nec (Zetterstedt, 1844) [Hackman 1980]

EPICAMPOCERA Macquart, 1849

Epicampocera succincta (Meigen, 1824) [Hedström 1964, Hackman 1980]

PHRYXE Robineau-Desvoidy, 1830

Phryxe erythrostoma (Hartig, 1838) [Hackman 1980]

Phryxe magnicornis (Zetterstedt, 1838) [Mattila 2009]

Phryxe nemea (Meigen, 1824) [Hackman 1980]

Phryxe vulgaris (Fallén, 1810) [Hackman 1980]

PERIARCHICLOPS Villeneuve, 1924

Periarchiclops scutellaris (Fallén, 1820) [Hackman 1981]

BACTROMYIA Brauer & Bergenstamm, 1891

Bactromyia aurulenta (Meigen, 1824) [Hackman 1980]

= *Thecocarcelia acutangula* misid.[Hackman 1981]

PSEUDOPERICHAETA Brauer & Bergenstamm, 1899

Pseudoperichaeta nigrolineata (Stephens, 1853) [Haarto 1999]

LYDELLA Robineau-Desvoidy, 1830

Lydella ripae (Brischke, 1885) [Haarto 1997]

= *L. grisescens* misid.[Hackman 1980]

= *L. lepida* misid.[Mee 1928]

Lydella stabulans (Meigen, 1824) [Hackman 1980]

CADURCIELLA Villeneuve, 1927

Cadurciella tritaeniata (Rondani, 1859) [Hackman 1980]

DRINO Robineau-Desvoidy, 1863

Drino bohemica Mesnil, 1949

Drino galii (Brauer & Bergenstamm, 1891)

Drino gilva (Hartig, 1838)

Drino inconspicua (Meigen, 1830) [Hackman 1980]
Drino lota (Meigen, 1824) [Hackman 1981]
Drino vicina (Zetterstedt, 1849) [Hackman 1980]
HUBNERIA Robineau-Desvoidy, 1848
Hubneria affinis (Fallén, 1810) [Hackman 1980]
CARCELIA Robineau-Desvoidy, 1830
sg. ***Carcelia*** Robineau-Desvoidy, 1830
Carcelia atricosta Herting, 1961
= *C. bombylans* misid.[Hackman 1980]
Carcelia bombylans Robineau-Desvoidy, 1830
Carcelia gnava (Meigen, 1824)
= *C. excavata* (Zetterstedt, 1844) [Hackman 1980]
Carcelia laxifrons Villeneuve, 1912 [Hackman 1981]
Carcelia lucorum (Meigen, 1824) [Hackman 1980]
Carcelia rasa (Macquart, 1849)
= *C. amphion* Robineau-Desvoidy, 1863 [Hackman 1980]
Carcelia tibialis (Robineau-Desvoidy, 1863)
SENOMETOPIA Macquart, 1834
Senometopia excisa (Fallén, 1820) [Hackman 1980]
Senometopia intermedia (Herting, 1960)
Senometopia pollinosa Mesnil, 1941 [Hackman 1980]
= *S. rutilla* auct. nec (Villeneuve, 1912)
= *S. obesa* auct. nec (Zetterstedt, 1859)
Senometopia separata (Rondani, 1859)
ERYCIA Robineau-Desvoidy, 1830
Erycia fatua (Meigen, 1824) [Komonen 1997]
= *E. festinans* misid.[Hackman 1980]
Erycia furibunda (Zetterstedt, 1844)
XYLOTACHINA Brauer & Bergenstamm, 1891
Xylotachina diluta (Meigen, 1824) [Hackman 1980]
tribe Goniini Lioy, 1864
PLATYMYA Robineau-Desvoidy, 1830
Platymya fimbriata (Meigen, 1824) [Hackman 1980]
EUMEA Robineau-Desvoidy, 1863
= ***Platymya*** Robineau-Desvoidy, 1830 in part
Eumea linearicornis (Zetterstedt, 1844) [Hackman 1981]
= *E. westermanni* (Zetterstedt, 1844)
Eumea mitis (Meigen, 1824) [Hackman 1980]
MYXEXORISTOPS Townsend, 1911
Myxexoristops abietis Herting, 1964
Myxexoristops arctica (Zetterstedt, 1838)
Myxexoristops bonsdorffi (Zetterstedt, 1859) [Hackman 1980]
Myxexoristops stolida (Stein, 1924) [Hackman 1981]
= *M. blondeli* misid.[Hackman 1980]
ZENILLIA Robineau-Desvoidy, 1830
Zenillia fulva (Fallén, 1820)
= *Z. libatrix* (Panzer, 1798) preocc.[Hackman 1980]
CLEMELIS Robineau-Desvoidy, 1863
Clemelis pullata (Meigen, 1824) [Hackman 1980]
PALES Robineau-Desvoidy, 1830

Pales pavida (Meigen, 1824) [Hackman 1980]
CYZENIS Robineau-Desvoidy, 1863
Cyzenis albicans (Fallén, 1810) [Hackman 1980]
Cyzenis jucunda (Meigen, 1838) [Hackman 1980]
BOTRIA Rondani, 1856
Botria frontosa (Meigen, 1824)
Botria subalpina Villeneuve, 1910 [Hackman 1980]
CEROMASIA Rondani, 1856
Ceromasia rubrifrons (Macquart, 1834)
ALLOPHOROCERA Hendel, 1901
= ***Erycilla*** Mesnil, 1957
Allophorocera ferruginea (Meigen, 1824) [Hackman 1980]
Allophorocera lapponica Wood, 1974 [Wood 1975]
OCYTATA Gistel, 1848
Ocytata pallipes (Fallén, 1820)
ERYNNIA Robineau-Desvoidy, 1830
Erynnia ocypterata (Fallén, 1810) [Hackman 1980]
= *E. nitida* (Robineau-Desvoidy, 1830)
ELODIA Robineau-Desvoidy, 1863
Elodia ambulatoria (Meigen, 1824) [Hackman 1980]
= *E. convexifrons* (Zetterstedt, 1844)
STURMIA Robineau-Desvoidy, 1830
Sturmia bella (Meigen, 1824) [Hackman 1980]
BLEPHARIPA Rondani, 1856
Blepharipa pratensis (Meigen, 1824)
PROSOPEA Rondani, 1861
Prosopea nigricans (Egger, 1861) [Tiensuu 1941, Hackman 1981]
HEBIA Robineau-Desvoidy, 1830
Hebia flavipes Robineau-Desvoidy, 1830 [Hackman 1980]
FRONTINA Meigen, 1838
Frontina laeta (Meigen, 1824) [Hackman 1980]
BRACHICHETA Rondani, 1861
Brachicheta strigata (Meigen, 1824) [Hackman 1980]
GONIA Meigen, 1803
Gonia capitata (De Geer, 1776) [Hackman 1980]
Gonia divisa Meigen, 1826 [Hackman 1980]
Gonia ornata Meigen, 1826 [Hackman 1980]
Gonia picea (Robineau-Desvoidy, 1830) [Hackman 1980]
= *G. sicula* auct. nec (Robineau-Desvoidy, 1830)
ONYCHOGONIA Brauer & Bergenstamm, 1889
Onychogonia cervini (Bigot, 1881)
Onychogonia flaviceps (Zetterstedt, 1838) [Hackman 1980]
= *O. interrupta* (Rondani, 1859) [Hackman 1980]
SPALLANZANIA Robineau-Desvoidy, 1830
Spallanzania hebes (Fallén, 1820) [Hackman 1980]
TACHININAE Robineau-Desvoidy, 1830
tribe Polideini Brauer & Bergenstamm, 1889
LYDINA Robineau-Desvoidy, 1830
Lydina aenea (Meigen, 1824) [Hackman 1980]
LYPHA Robineau-Desvoidy, 1830

Lypha dubia (Fallén, 1810) [Hackman 1980]
Lypha ruficauda (Zetterstedt, 1838) [Hackman 1980]
tribe Tachinini Robineau-Desvoidy, 1830
TACHINA Meigen, 1803
= ***Larvaevora*** Meigen, 1800 suppr.
sg. ***Eudoromyia*** Bezzi, 1906
Tachina fera (Linnaeus, 1761) [Hackman 1980]
Tachina sp. aff. *magnicornis*
Tachina magnicornis (Zetterstedt, 1844) [Hackman 1980]
sg. ***Servillia*** Robineau-Desvoidy, 1830
Tachina ursina (Meigen, 1824) [Hackman 1980]
sg. ***Tachina*** Meigen, 1803
Tachina grossa (Linnaeus, 1758) [Hackman 1980]
NOWICKIA Wachtl, 1894
Nowickia alpina (Zetterstedt, 1844) [Hackman 1980]
Nowickia ferox (Panzer, 1809) [Hackman 1980]
Nowickia marklini (Zetterstedt, 1838) [Hackman 1980]
PELETERIA Robineau-Desvoidy, 1830
= ***Cuphocera*** Macquart, 1845
Peleteria ferina (Zetterstedt, 1844) [Hackman 1980]
Peleteria rubescens Robineau-Desvoidy, 1830 [Hackman 1980]
= *P. nigricornis* (Meigen, 1838)
Peleteria ruficornis (Macquart, 1835) [Haarto 1999]
GERMARIA Robineau-Desvoidy, 1830
= ***Atractochaeta*** Brauer & Bergenstamm, 1889
Germaria angustata (Zetterstedt, 1844) [Hackman 1980]
Germaria ruficeps (Fallén, 1820) [Hackman 1980]
tribe Linnaemyini Townsend, 1919
LINNAEMYA Robineau-Desvoidy, 1830
Linnaemya haemorrhoidalis (Fallén, 1810) [Hackman 1980]
Linnaemya olsufjevi Zimin, 1954
= *L. haemorrhoidalis* misid.
Linnaemya rossica Zimin, 1954
= *L. haemorrhoidalis* misid.
Linnaemya vulpina (Fallén, 1810) [Hackman 1980]
Linnaemya sp. A
CHRYSOSOMOPSIS Townsend, 1916
Chrysosomopsis aurata (Fallén, 1820) [Hackman 1980]
PANZERIA Robineau-Desvoidy, 1830
= ***Ernestia*** Robineau-Desvoidy, 1830
Panzeria puparum (Fabricius, 1794)
Panzeria rudis (Fallén, 1810) [Hackman 1980]
Panzeria vagans (Meigen, 1824)
APPENDICIA Stein, 1924
Appendicia truncata (Zetterstedt, 1838) [Hackman 1980]
EURITHIA Robineau-Desvoidy, 1844
Eurithia anthophila (Robineau-Desvoidy, 1830) [Hackman 1980]
= *E. radicum* auct. nec Linnaeus, 1758
Eurithia caesia (Fallén, 1810) [Hackman 1980]
Eurithia connivens (Zetterstedt, 1844) [Hackman 1980]

Eurithia consobrina (Meigen, 1824) [Hackman 1980]
Eurithia intermedia (Zetterstedt, 1844)
= *E. conjugata* (Zetterstedt, 1855) [Hackman 1980]
Eurithia vivida (Zetterstedt, 1838) [Hackman 1980]
HYALURGUS Brauer & Bergenstamm, 1893
Hyalurgus crucigerus (Zetterstedt, 1838) [Hackman 1980]
Hyalurgus lucidus (Meigen, 1824) [Hackman 1980]
NEMORAEA Robineau-Desvoidy, 1830
Nemoraea pellucida (Meigen, 1824)
GYMNOCHETA Robineau-Desvoidy, 1830
Gymnocheta magna (Fallén, 1810)
Gymnocheta viridis (Fallén, 1810) [Hackman 1980]
ZOPHOMYIA Macquart, 1835
Zophomyia temula (Scopoli, 1763) [Hackman 1980]
CLEONICE Robineau-Desvoidy, 1863
= ***Steiniella*** Berg, 1898 , preocc.
Cleonice callida (Meigen, 1824) [Hackman 1980]
Cleonice keteli Ziegler, 2000
Cleonice nitidiuscula (Zetterstedt, 1859) [Tiensuu 1941]
LOEWIA Egger, 1856
= ***Fortisia*** Rondani, 1861
Loewia erecta Bergström, 2007 [Bergström 2007]
= *L. phaeoptera* misid.[Hackman 1980]
Loewia foeda (Meigen, 1824) [Hackman 1980]
ELOCERIA Robineau-Desvoidy, 1863
Eloceria delecta (Meigen, 1824) [Hackman 1980]
tribe Brachymerini Mesnil, 1939
PSEUDOPACHYSTYLUM Mik, 1891
Pseudopachystylum gonioides (Zetterstedt, 1838) [Mee 1928]
tribe Pelatachinini Mesnil, 1966
PELATACHINA Meade, 1894
Pelatachina tibialis (Fallén, 1810) [Hackman 1980]
tribe Macquartiini Robineau-Desvoidy, 1830
MACQUARTIA Robineau-Desvoidy, 1830
Macquartia dispar (Fallén, 1820) [Hackman 1980]
Macquartia nudigena Mesnil, 1972 [Hackman 1980]
= *M. buccalis* auct. nec Robineau-Desvoidy, 1830
Macquartia tenebricosa (Meigen, 1824) [Hackman 1980]
Macquartia viridana Robineau-Desvoidy, 1863
ANTHOMYIOPSIS Townsend, 1916
= ***Ptilopsina*** Villeneuve, 1920
Anthomyiopsis nigrisquamata (Zetterstedt, 1838) [Hackman 1980]
tribe Triarthriini Belshaw, 1993
TRIARTHRIA Stephens, 1829
Triarthria setipennis (Fallén, 1810) [Hackman 1980]
tribe Neaerini Mesnil, 1966
PHYTOMYPTERA Rondani, 1845
= ***Elfia*** Robineau-Desvoidy, 1850
Phytomyptera bohemica Kramer, 1907 [Hackman 1980]
Phytomyptera cingulata (Robineau-Desvoidy, 1830) [Hackman 1980]

Phytomyptera nigrina (Meigen, 1824) [Hackman 1980]
Phytomyptera minutissima (Zetterstedt, 1844)
Phytomyptera riedeli (Villeneuve, 1930)
Phytomyptera zonella (Zetterstedt, 1844) [Hackman 1980]
GRAPHOGASTER Rondani, 1868
= ***Anurogyna*** Brauer & Bergenstamm, 1889
Graphogaster brunnescens Villeneuve, 1907
Graphogaster buccata Herting, 1971 [Hackman 1980]
Graphogaster dispar (Brauer & Bergenstamm, 1889) [Hackman 1980]
= *G. punctiventris* (Ringdahl, 1942)
Graphogaster nigrescens Herting, 1971 [Winqvist 2011]
tribe Siphonini Rondani, 1844
ENTOMOPHAGA Lioy, 1864
Entomophaga nigrohalterata (Villeneuve, 1921)
Entomophaga sufferta (Villeneuve, 1942) [Andersen 1996]
CEROMYA Robineau-Desvoidy, 1830
Ceromya bicolor (Meigen, 1824) [Hackman 1980]
Ceromya dorsigera Herting, 1967
Ceromya flaviceps Ratzeburg, 1844 [Andersen 1996]
Ceromya silacea (Meigen, 1824) [Hackman 1981]
ACTIA Robineau-Desvoidy, 1830
Actia crassicornis (Meigen, 1824) [Hackman 1980]
Actia infantula (Zetterstedt, 1844)
Actia lamia (Meigen, 1838) [Hackman 1980]
Actia maksymovi Mesnil, 1952 [Andersen 1996]
Actia nigroscutellata Lundbeck, 1927 [Hackman 1980]
Actia pilipennis (Fallén, 1810) [Hackman 1980]
Actia resinellae (Schrank, 1781)
= *A. nudibasis* Stein, 1924 [Hackman 1980]
PERIBAEA Robineau-Desvoidy, 1863
Peribaea hertingi Andersen, 1996 [Andersen 1996]
= *P. fissicornis* misid.[Hackman 1980]
Peribaea longirostris Andersen, 1996
CERANTHIA Robineau-Desvoidy, 1830
Ceranthia abdominalis Robineau-Desvoidy, 1830 [Hackman 1980]
Ceranthia lichtwardtiana (Villeneuve, 1931) [Andersen 1996]
Ceranthia pallida Herting, 1959 [Andersen 1996]
Ceranthia tenuipalpis (Villeneuve, 1921) [Andersen 1996]
Ceranthia tristella Herting, 1966
Ceranthia verneri Andersen, 1996 [Andersen 1996]
APHANTORHAPHOPSIS Townsend, 1926
Aphantorhaphopsis samarensis (Villeneuve, 1921) [Andersen 1996]
Aphantorhaphopsis siphonoides (Strobl, 1898)
Aphantorhaphopsis verralli (Wainwright, 1928) [Hedström 1964, Hackman 1980]
SIPHONA Meigen, 1803
Siphona boreata Mesnil, 1960 [Andersen 1982]
Siphona collini Mesnil, 1960 [Andersen 1996]
Siphona confusa Mesnil, 1961 [Andersen 1996]
= *S. mesnili* Andersen, 1982 [Andersen 1982]
Siphona cristata (Fabricius, 1805) [Hackman 1980]

= *S. geniculata* auct. nec (De Geer, 1776) [Andersen 1996]
Siphona flavifrons (Staeger, 1849) [Hackman 1980]
Siphona geniculata (De Geer, 1776) [Hackman 1980]
= *S. urbana* (Harris, 1776) [Andersen 1996]
Siphona grandistyla Pandellé, 1894
Siphona immaculata Andersen, 1996 [Andersen 1996]
Siphona ingerae Andersen, 1982 [Andersen 1982, 1996]
Siphona hokkaidensis Mesnil, 1957
= *S. silvarum* Herting, 1967 [Andersen 1982]
= *S. martini* Andersen, 1982 [Andersen 1982]
Siphona maculata (Staeger, 1849) [Hackman 1980]
Siphona nigricans (Villeneuve, 1930) [Pohjoismäki 2006]
Siphona paludosa Mesnil, 1960 [Andersen 1982]
Siphona pauciseta Rondani, 1865 [Andersen 1996]
Siphona rossica Mesnil, 1961 [Andersen 1996]
Siphona setosa Mesnil, 1960 [Andersen 1996]
Siphona subarctica Andersen, 1996 [Andersen 1996]
Siphona variata Andersen, 1982
tribe Leskiini Townsend, 1919
APHRIA Robineau-Desvoidy, 1830
Aphria longilingua Rondani, 1861 [Haarto 1999]
Aphria longirostris (Meigen, 1824) [Hackman 1980]
DEMOTICUS Macquart, 1854
Demoticus plebejus (Fallén, 1810) [Hackman 1980]
LESKIA Robineau-Desvoidy, 1830
Leskia aurea (Fallén, 1820) [Hackman 1980]
SOLIERIA Robineau-Desvoidy, 1848
Solieria inanis (Fallén, 1810) [Hackman 1980]
Solieria pacifica (Meigen, 1824) [Hackman 1981]
= *S. tibialis* (von Roser, 1840)
tribe Minthonini Brauer & Bergenstamm, 1889
MINTHO Robineau-Desvoidy, 1830
Mintho rufiventris (Fallén, 1816) [Hackman 1980]
MINTHODES Brauer & Bergenstamm, 1899
Minthodes picta (Zetterstedt, 1844)
tribe Microphthalmini Crosskey, 1976
DEXIOSOMA Rondani, 1856
Dexiosoma caninum (Fabricius, 1794) [Hackman 1980]
DEXIINAE Macquart, 1834
tribe Dexiini Macquart, 1834
TRIXA Meigen, 1824
= ***Murana*** Meigen, 1824
Trixa caerulescens Meigen, 1824 [Zhang and Shima 2005]
= *T. alpina* Meigen, 1824 [Hackman 1980]
Trixa conspersa (Harris, 1776)
= *T. variegata* Meigen, 1824 [Hackman 1980]
= *T. oestroidea* (Robineau-Desvoidy, 1830)
BILLAEA Robineau-Desvoidy, 1830
Billaea irrorata (Meigen, 1826) [Hackman 1980]
Billaea kolomyetzi Mesnil, 1970

Billaea triangulifera (Zetterstedt, 1844) [Hackman 1980]
DINERA Robineau-Desvoidy, 1830
Dinera ferina (Fallén, 1816) [Hackman 1980]
Dinera grisescens (Fallén, 1816) [Hackman 1980]
Dinera carinifrons (Fallén, 1817) [Hackman 1980]
ESTHERIA Robineau-Desvoidy, 1830
Estheria petiolata (Bonsdorff, 1866) [Hackman 1980]
DEXIA Meigen, 1826
Dexia vacua (Fallén, 1816) [Hackman 1980]
PROSENA Le Peletier & Serville, 1828
Prosena siberita (Fabricius, 1775) [Hackman 1980]
tribe Voriini Townsend, 1912
ERIOTHRIX Meigen, 1803
Eriothrix argyreatus (Meigen, 1824)
= *E. apenninus* misid.[Hackman 1980]
Eriothrix prolixa (Meigen, 1824) [Hackman 1980]
Eriothrix rufomaculata (De Geer, 1776) [Hackman 1980]
= *E. monochaeta* (Wainwright, 1928)
TRAFOIA Brauer & Bergenstamm, 1893
Trafoia monticola Brauer & Bergenstamm, 1893
CAMPYLOCHETA Rondani, 1859
= ***Elpe*** Robineau-Desvoidy, 1863
Campylocheta fuscinervis (Stein, 1924)
Campylocheta inepta (Meigen, 1824) [Hackman 1980]
Campylocheta praecox (Meigen, 1824) [Hackman 1980]
BLEPHAROMYIA Brauer & Bergenstamm, 1889
Blepharomyia angustifrons Herting, 1971
Blepharomyia pagana (Meigen, 1824) [Hackman 1980]
= *B. amplicornis* (Zetterstedt, 1844)
Blepharomyia piliceps (Zetterstedt, 1859) [Hackman 1980]
PETEINA Meigen, 1838
Peteina erinaceus (Fabricius, 1794) [Hackman 1980]
RAMONDA Robineau-Desvoidy, 1863
Ramonda prunaria (Rondani, 1861) [Haarto et al. 2002]
= *R. carbonaria* misid.[Hackman 1980]
Ramonda ringdahli (Villeneuve, 1922) [Haarto 1999]
Ramonda spathulata (Fallén, 1820) [Hackman 1980]
WAGNERIA Robineau-Desvoidy, 1830
Wagneria alpina (Villeneuve, 1910) [Hackman 1980]
Wagneria costata (Fallén, 1820) [Hackman 1980]
ATHRYCIA Robineau-Desvoidy, 1830
= ***Blepharigena*** Rondani, 1856
Athrycia curvinervis (Zetterstedt, 1844) [Hackman 1981]
Athrycia impressa (van der Wulp, 1869)
Athrycia trepida (Meigen, 1824) [Hackman 1980]
Athrycia sp. A
VORIA Robineau-Desvoidy, 1830
= ***Plagia*** Meigen, 1838
Voria ruralis (Fallén, 1810) [Hackman 1980]
CYRTOPHLEBA Rondani, 1856

Cyrtophleba ruricola (Meigen, 1824) [Hackman 1980]
Cyrtophleba vernalis (Kramer, 1917)
KLUGIA Robineau-Desvoidy, 1863
Klugia marginata (Meigen, 1824) [Hackman 1980]
CHAETOVORIA Villeneuve, 1920
Chaetovoria antennata Villeneuve, 1920 [Hedström and Nuorteva 1971]
PHYLLOMYA Robineau-Desvoidy, 1830
Phyllomya volvulus (Fabricius, 1794) [Hackman 1980]
THELAIRA Robineau-Desvoidy, 1830
Thelaira nigrina (Fallén, 1817)
= *T. nigripes* (Fabricius, 1794) preocc.[Hackman 1980]
HALIDAYA Egger, 1856
Halidaya aurea Egger, 1856
STOMINA Robineau-Desvoidy, 1830
Stomina tachinoides (Fallén, 1816) [Hackman 1980]
tribe Dufouriini Robineau-Desvoidy, 1830
DUFOURIA Robineau-Desvoidy, 1830
Dufouria chalybeata (Meigen, 1824) [Hackman 1980]
Dufouria nigrita (Fallén, 1810) [Hackman 1980]
RONDANIA Robineau-Desvoidy, 1850
Rondania dimidiata (Meigen, 1824) [Hackman 1980]
Rondania dispar (Dufour, 1851)
Rondania fasciata (Macquart, 1834) [Hackman 1981]
MICROSOMA Macquart, 1855
= ***Campogaster*** Rondani, 1856
Microsoma exiguum (Meigen, 1824) [Hackman 1980]
FRERAEA Robineau-Desvoidy, 1830
Freraea gagatea Robineau-Desvoidy, 1830 [Hackman 1980]
= *F. albipennis* (Zetterstedt, 1838)
PHASIINAE Robineau-Desvoidy, 1830
tribe Phasiini Robineau-Desvoidy, 1830
CLYTIOMYA Rondani, 1861
Clytiomya continua (Panzer, 1798)
ELIOZETA Rondani, 1856
Eliozeta pellucens (Fallén, 1820) [Hackman 1980]
SUBCLYTIA Pandellé, 1894
Subclytia rotundiventris (Fallén, 1820) [Hackman 1981]
GYMNOSOMA Meigen, 1803
Gymnosoma clavatum (Rohdendorf, 1947) [Tschorsnig and Herting 1994]
Gymnosoma dolycoridis Dupuis, 1961
Gymnosoma nudifrons Herting, 1966 [Tschorsnig and Herting 1994]
CISTOGASTER Latreille, 1804
= ***Pallasia*** Robinaeu-Desvoidy, 1830
Cistogaster globosa (Fabricius, 1775) [Hackman 1980]
PHASIA Latreille, 1804
= ***Alophora*** Robineau-Desvoidy, 1830
sg. ***Hyalomya*** Robineau-Desvoidy, 1830
Phasia pusilla (Meigen, 1824) [Hackman 1980]
sg. ***Phasia*** Latreille, 1804
Phasia aurulans (Meigen, 1824) [Hackman 1980]

Phasia barbifrons (Girschner, 1887) [Pohjoismäki 2006]
Phasia hemiptera (Fabricius, 1794) [Hackman 1980]
Phasia obesa (Fabricius, 1798) [Hackman 1980]
Phasia subcoleoptrata (Linnaeus, 1767) [Hackman 1980]
tribe Catharosiini Townsend, 1936
CATHAROSIA Rondani, 1868
Catharosia pygmaea (Fallén, 1815) [Hackman 1980]
tribe Strongygastrini Townsend, 1936
STRONGYGASTER Macquart, 1834
= ***Tamiclea*** Macquart, 1836
Strongygaster celer (Meigen, 1838) [Hackman 1980]
tribe Leucostomatini Townsend, 1908
LEUCOSTOMA Meigen, 1803
Leucostoma simplex (Fallén, 1815) [Hackman 1980]
BRULLAEA Robineau-Desvoidy, 1863
Brullaea ocypteroidea Robineau-Desvoidy, 1863
CINOCHIRA Zetterstedt, 1845
Cinochira atra Zetterstedt, 1945
tribe Cylindromyiini Townsend, 1912
LOPHOSIA Meigen, 1824
sg. ***Lophosia*** Meigen, 1824
Lophosia fasciata Meigen, 1824 [Hackman 1980]
CYLINDROMYIA Meigen, 1803
sg. ***Cylindromyia*** Meigen, 1803
Cylindromyia brassicaria (Fabricius, 1775) [Hackman 1980]
sg. ***Neocyptera*** Townsend, 1916
Cylindromyia interrupta (Meigen, 1824) [Hackman 1980]
sg. ***Ocypterula*** Rondani, 1856
Cylindromyia pusilla (Meigen, 1824) [Hackman 1980]
HEMYDA Robineau-Desvoidy, 1830
Hemyda obscuripennis (Meigen, 1824)
Hemyda vittata (Meigen, 1824)
BESSERIA Robineau-Desvoidy, 1830
Besseria anthophila (Loew, 1871) [Hackman 1980]
Besseria melanura (Meigen, 1824) [Tiensuu 1941]
PHANIA Meigen, 1824
Phania curvicauda (Fallén, 1820) [Hackman 1980]
Phania funesta (Meigen, 1824)
= *P. pseudofunesta* (Villeneuve, 1931) unjustified new name
Phania thoracica Meigen, 1824 [Hackman 1980]

Excluded species

Aphantorhaphopsis selecta (Pandellé, 1894) [Hackman 1981, Andersen 1996]
Baumhaueria goniaeformis (Meigen, 1824) [Tiensuu 1941, Hackman 1980]
Billaea fortis (Rondani, 1862) [Hackman 1980] probably misid.
Bithia geniculata (Zetterstedt, 1844) [Hackman 1980, 1981]
Blepharipoda scutellata (Robineau-Desvoidy, 1830) [Hackman 1980]
Blondelia piniariae (Hartig, 1838)

Dexia rustica (Fallén, 1816) [Lundbeck 1927]   ?
Drino atropivora Robineau-Desvoidy, 1830
Entomophaga exoleta (Meigen, 1824) [Hackman 1981, Andersen 1996]
Graphogaster vestita Rondani, 1868 [Hackman 1980]
Gymnosoma rotundatum (Linnaeus, 1758) [Hackman 1980]
Istocheta subcinerea (Borisova-Zinoveva, 1966) [Herting and Dely-Draskovits 1993]   ?
Leiophora innoxia (Meigen, 1824) [Hackman 1981]   ?
Linnaemya comta (Fallén, 1810) [Hackman 1980]
Lydella grisescens Robineau-Desvoidy, 1830 [Hackman 1980]
Macroprosopa atrata (Fallén, 1810) [Hackman 1980]   ?
Masicera silvatica (Fallén, 1810) [Lundbeck 1927]   ?
Opesia cana (Meigen, 1824) [Zetterstedt 1859]
Pseudogonia rufifrons (Wiedemann, 1830)
Pseudoperichaeta palesoidea (Robineau-Desvoidy, 1830) [Hackman 1980]
Senometopia confundens Rondani, 1859 [Hackman 1980]
Thelymorpha marmorata (Fabricius, 1805) [Hackman 1980]

OESTRIDAE Leach, 1815

Supporting references for *Pohjoismäki J, Kahanpää J (2014) Checklist of the superfamilies Oestroidea and Hippoboscoidea of Finland (Insecta, Diptera). In: Kahanpää J, Salmela J (Eds) Checklist of the Diptera of Finland. ZooKeys @@: @-@. doi: 10.3897/zookeys.??.7252*

HYPODERMATINAE Rondani, 1856
HYPODERMA Latreille, 1818
Hypoderma bovis (Linnaeus, 1758) [Hackman 1980]
Hypoderma lineatum (De Villers, 1789) [Hackman 1980]
Hypoderma tarandi (Linnaeus, 1758) [Hackman 1980]
CEPHENEMYIINAE Patton, 1921
tribe Cephenemyiini Patton, 1921
CEPHENEMYIA Latreille, 1818
Cephenemyia trompe (Modeer, 1786) [Hackman 1980]
Cephenemyia ulrichii Brauer, 1862 [Hackman 1980]
GASTEROPHILINAE Girschner, 1896
GASTEROPHILUS Leach, 1817
Gasterophilus haemorrhoidalis (Linnaeus, 1758) [Hackman 1980]
Gasterophilus intestinalis (De Geer, 1776) [Hackman 1980]
Gasterophilus nasalis (Linnaeus, 1758) [Hackman 1980]

Excluded species

tribe Oestrini Leach, 1815
OESTRUS Linnaeus, 1758
? *Oestrus ovis* Linnaeus, 1758 imported?

HIPPOBOSCIDAE Samouelle, 1819

Supporting references for *Pohjoismäki J, Kahanpää J (2014) Checklist of the superfamilies Oestroidea and Hippoboscoidea of Finland (Insecta, Diptera). In: Kahanpää J, Salmela J (Eds) Checklist of the Diptera of Finland. ZooKeys @@: @-@. doi: 10.3897/zookeys.??.7252*

HIPPOBOSCINAE Samouelle, 1819
tribe Ornithomyini Costa, 1846
CRATAERINA von Olfers, 1816
= ***Stenepteryx*** Leach, 1817
Crataerina hirundinis (Linnaeus, 1758) [Hackman 1980]
Crataerina pallida (Olivier, 1812) [Hackman 1980]
tribe Olfersiini Maa, 1969
OLFERSIA Wiedemann, 1830
= ***Feronia*** Leach, 1817 preocc.
Olfersia fumipennis (Sahlberg, 1886) [Hackman 1980]
ORNITHOMYA Latreille, 1802
Ornithomya avicularia (Linnaeus, 1758) [Hackman 1980]
Ornithomya chloropus Bergroth, 1901 [Hackman 1980]
Ornithomya fringillina Curtis, 1836 [Hackman 1980]
ORNITHOPHILA Rondani, 1879
Ornithophila metallica (Schiner, 1864) [Itämies et al. 1976, Hackman 1980]
tribe Hippoboscini Samouelle, 1819
HIPPOBOSCA Linnaeus, 1758
Hippobosca equina Linnaeus, 1758 [Hackman 1980]
tribe Lipoptenini Speiser, 1908
LIPOPTENA Nitzsch, 1818
Lipoptena cervi (Linnaeus, 1758) [Karppinen 1966, Hackman 1980]
MELOPHAGUS Latreille, 1802
Melophagus ovinus (Linnaeus, 1758) [Hackman 1980]
NYCTERIBIINAE Samouelle, 1819
tribe Nycteribiini Samouelle, 1819
NYCTERIBIA Latreille, 1796
sg. ***Nycteribia*** Latreille, 1796
Nycteribia kolenatii Theodor & Moscona, 1954 [Väisänen 1983]
= *N. pedicularia* auct. nec Latreille, 1805
= *N. latreillei* misid.[Hackman 1980, Väisänen 1983]
= *N. blasii* (Kolenati, 1863) preocc.
PENICILLIDIA Kolenati, 1863
Penicillidia monoceros Speiser, 1900 [Hackman 1980]

References

(1927) Föreningsmeddelanden. - Tietoja yhdistyksistä. Notulae entomologicae 67(2): 55–63.

(1928) Föreningsmeddelanden. - Tietodonantoja yhdistyksistä. Notulae entomologicae 8(2): 50–60.

(1929) Tiedonantoja yhdistyksistä. - Föreningsmeddelanden. Notulae Entomologicae 9(4): 113–129.

(1930) Tiedonantoja yhdistyksistä. Föreningsmeddelanden. Notulae Entomologicae 10(3-4): 106–120.

(1935) Tiedonantoja yhdistyksistä. Föreningsmeddelanden. Notulae entomologicae 15(4): 104–121.

(1936) Föreningsmeddelanden. Tietoja yhdistyksistä. Notulae Entomologicae 16(3): 88–91.

(1956) Kokousselostuksia. Annales Entomologici Fennici 22(2): 95–102.

(1960) Mötesreferat. - Kokousselostuksia. Notulae entomologicae 39[1959](4): 132–141.

(1961a) Mötesreferat - Kokousselostuksia. Notulae Entomologicae 41(1): 25–30.

(1961b) Mötesreferat. - Kokousselostuksia. Notulae Entomologicae 41(3): 101–104.

(1961) Mötesreferat. - Kokousselostuksia. Notulae Entomologicae 40[1960](4): 153–156.

(1962) Mötesreferat. - Kokousselostuksia. Notulae Entomologicae 42(1): 27–32.

(1963) Mötesreferat. - Kokousselostuksia. Notulae Entomologicae 43(4): 165–172.

(1964) Mötesreferat. - Kokousselostuksia. Notulae Entomologicae 44(4): 137–140.

(1965) Mötesreferat Kokousselostuksia. Notulae Entomologicae 45(3): 97–100.

(1967) Mötesreferat. - Kokousselostuksia. Notulae entomologicae 47(4): 156–161.

(1969) Mötesreferat. - Kokousselostuksia. Notulae entomologicae 49(4): 284–288.

(1970) Mötesreferat Kokousselostuksia. Notulae entomologicae 50(4): 131–136.

(1971) Mötesreferat Kokousselostuksia. Notulae Entomologicae 51(4): 132–136.

(1975) Mötesreferat. - Kokousselostuksia. Notulae Entomologicae 55(4): 136–140.

Ackland DM (1989) Anthomyiidae (Dipt.) new to Britain, with a description of a new species of Botanophila Lioy. Entomologist's Monthly Magazine 125: 211–230.

Ackland DM, Michelsen V (1986) The Palearctic species of the Phorbia curvicauda (Zetterstedt) group (Diptera: Anthomyiidae). Entomologica scandinavica 17(4): 423–432 doi:10.1163/187631286X00026. http://www.ingentaconnect.com/content/brill/ise/1986/00000017/00000004/art00002.

Albrecht A (1979a) Descriptions of seven new Dorylomorpha Aczél species from Europe (Diptera: Pipunculidae). Entomologica scandinavica 10(3): 211–218 doi:10.1163/187631279794814986.

Albrecht A (1979b) Dorylomorpha fennica sp.n., a new Pipunculid species (Diptera) from Finland. Notulae entomologicae 59(1): 15–17.

Albrecht A (1979c) För Finlands fauna nya ögonflugor (Pipunculidae). Notulae Entomologicae 59(4): 172.

Albrecht A (1981) Tvåför Finland nya ögonflugor (Pipunculidae). Notulae Entomologicae 61(4): 225.

Albrecht A (1983) Dorylomorpha anderssoni Albrecht ny for Finland. Notulae Entomologicae 63(4): 216.

Albrecht A (1990) Revision, phylogeny and classification of the genus Dorylomorpha (Diptera, Pipunculidae). Acta Zoologica Fennica 188: 1–240.

Andersen S (1982) Revision of European species of Siphona Meigen (Diptera: Tachinidae). Entomologica scandinavica 13(2): 149–172 doi:10.1163/187631282X00066. http://www.ingentaconnect.com/content/brill/ise/1982/00000013/00000002/art00006.

Andersen S (1996) The Siphonini (Diptera: Tachinidae) of Europe. Fauna Entomologica Scandinavica E. J. Brill, Leiden - New York, 148 pp. ISBN 9789004107311.

Andersson H (1966) A revision of the species of Oscinides described by J. W. Zetterstedt (Dipt. Chloropidae). Opuscula Entomologica 31(1-2): 61–76.

Andersson H (1976) Revision of the Anthomyza species of Northwest Europe (Diptera: Anthomyzidae) I. The gracilis group. Entomologica scandinavica 7(1): 41–52 doi:10.1163/187631276X00072.

Andersson H (1984) Revision of the Anthomyza species of Northwest Europe (Diptera: Anthomyzidae) II. The pallida group. Entomologica scandinavica 15(1): 15–24 doi:10.1163/187631284X00037.

Andersson H (1991) Family Lonchopteridae. In: Soós A, Papp L (Eds) Catalogue of Palaearctic Diptera, Volume 7: Dolichopodidae-Platypezidae. Akademiai Kiado, Budapest & Elsevier, Amsterdam, 139–142.

Autio O, Salmela J (2010) The semi-aquatic fly fauna (Diptera) of wetlands of the Åland Islands. Memoranda Societatis pro Fauna et Flora Fennica 86: 43–53. http://ojs.tsv.fi/index.php/msff/article/view/3969.

Bächli G, Vilela C, Escher SA, Saura A (2004) The Drosophilidae (Diptera) of Fennoscandia and Denmark. Fauna Entomologica Scandinavica Brill NV, Leiden & Boston, 362 pp. ISBN 90-04-13265-1. http://www.cabdirect.org/abstracts/20053205535.html.

Bachli G, Vilela C, Haring E (2002) Four new species of West Palaearctic Drosophilidae (Diptera). Mitteilungen der Schweizerischen Entomologischen Gesellschaft 75: 299–333. http://www.dgrc.kit.ac.jp/~jdd/class/030710/03071006.pdf.

Bagge P, Ilus E, Paasivirta L (1980) Emergence of insects especially Diptera, Chironomidae at different depths in the archipelago of Lovisa Gulf of Finland in 1971. Annales Entomologici Fennici 46(4): 89–100.

Barnes HFR (1958) The Gall Midges (Dipt., Cecidomyiidae) of Timothy Grass Inflorences, with the description of one new species. Annales Entomologici Fennici 24(2): 59–68.

Barták M, Kubík v (2012) A review of the Palaearctic species of Rhamphomyia subgenus Holoclera (Diptera: Empididae) with description of 5 new species. Revue Suisse de Zoologie 119(3): 385–407.

Bartsch H (2000) Blomflugor under 20. finländska entomologidagarna i Jomala påÅland (Diptera, Syrphidae). Sahlbergia 5: 41–45.

Bartsch H, Stå hls G, Kerppola S (2010) Rödbent mulmblomfluga Chalcosyrphus valgus och dess dubbelgångare (Diptera, Syrphidae). Fauna & Flora 105(2): 30–35.

Bartsch HD, Nielsen TR, Speight MCD (2002) Reappraisal of Xylota caeruleiventris Zetterstedt, 1838, with remarks on the distribution of this species and X. jakutorum Bagatshanova, 1980 in Europe. Volucella 6: 69–79.

Basden EB (1961) Notes on the Camillidae (Diptera) in Strobl's collection and on the biology of Camilla. Notulae Entomologicae 41(4): 124–129. http://www.dgrc.kit.ac.jp/~jdd/class/030701/03070111.pdf.

Bergman G, Jansson A (1983) Accession to the Zoological Museum, University of Helsinki/Helsingfors 14.5.-31.12.1982. Memoranda Societatis pro Fauna et Flora Fennica 59: 165–166.

Bergman G, Nuorteva P (1974) Accessions to the Zoological Museum, University of Helsinki (Helsingfors), in 1973-1974. Memoranda Societatis pro Fauna et Flora Fennica 50: 143–145.

Bergström C (2005) A new species of the genus Phebellia Robineau-Desvoidy (Diptera: Tachinidae) from Finland. Stuttgarter Beiträge zur Naturkunde A 679: 1–9. http://www.naturkundemuseum-bw.de/sites/default/files/publikationen/serie-a/A679.pdf.

Bergström C (2007) Loewia erecta n. sp.(Diptera: Tachinidae) - a new parasitic fly from Fennoscandia and Poland. Stuttgarter Beiträge zur Naturkunde A 708: 1–16. http://www.naturkundemuseum-bw.de/sites/default/files/publikationen/serie-a/a_708.pdf.

Beyer E (1959) Drei neue Phoriden aus Japan und Finnland. Notulae entomologicae 38[1958](4): 104–108.

Borkent A, Bissett B (1990) A revision of the Holarctic species of Serromyia Meigen (Diptera: Ceratopogonidae). Systematic Entomology 15(2): 153–217 doi:10.1111/j.1365-3113.1990.tb00311.x. http://onlinelibrary.wiley.com/doi/10.1111/j.1365-3113.1990.tb00311.x/abstract.

Brander T (1971) Uusia huomion arvoisia hyönteislöytöjä Lounais-Hämeestä. Lounais-Hämeen Luonto 43: 32–35. http://www.lounaisluonto.net/julkaisut/LHL/LHL43.pdf.

Brax R (1991) Suomeen tulevista leikkokukista löytyi uusi kasvintuhooja, Thrips palmi. Kasvinsuojelulehti 24(1): 12–13.

Brodin Y, Lundström JO, Paasivirta L (2008) Tavastia yggdrasilia, a new orthoclad midge (Diptera: Chironomidae) from Europe. Aquatic Insects 30(4): 261–267 doi:10.1080/01650420802448515. http://urn.kb.se/resolve?urn=urn:nbn:se:uu:diva-107105.

Brodo F (1987) A revision of the genus Prionocera (Diptera: Tipulidae). Evolutionary Monographs 8: 1–93.

Brodo F (1994) The subgenus Tipula (Tipula) in Finland and Estonia. Entomologica fennica 5(1): 49–52. http://ukpmc.ac.uk/abstract/AGR/IND20413545.

Brooks SE, Wheeler TA (2005) Ethiromyia, a new genus of holarctic Dolichopodinae (Diptera: Dolichopodidae). Proceedings of the Entomological Society of Washington 107(3): 489–500. http://cat.inist.fr/?aModele=afficheN\&cpsidt=16926266.

Brummer-Korvenkontio M, Korhonen P, Hämeen-Anttila R (1971) Ecology and phenology of mosquitoes (Dipt., Culicidae) inhabiting small pools in Finland. Acta entomologica fennica 28: 51–73.

Cannings RA, Kahanpää J (2013) Lasiopogon septentrionalis, a robber fly (Diptera: Asilidae) new to the European Fauna. Entomologica fennica 24(2): 113–116. http://ojs.tsv.fi/index.php/entomolfennica/article/download/8347/6317.

Carles-Tolrá M (1993) A new species of Chamaepsila Hendel from the Czech Republic, with a key to the bicolor-group species (Diptera, Psilidae). Graellsia 49: 91–95.

Chandler PJ (1987) The families Diastatidae and Campichoetidae (Diptera, Drosophiloidea) with a revision of Palaearctic and Nepalese species of Diastata Meigen. Entomologica scandinavica 18(1): 1–50 doi:10.1163/187631287X00016.

Chandler PJ (1992a) A review of the British Phronia Winnertz and Trichonta Winnertz (Dipt., Mycetophilidae). Entomologist’s Monthly Magazine 128: 237–254.

Chandler PJ (1992b) New records and nine additions to the British list of fungus gnats of the smaller families and subfamilies (Diptera: Mycetophiloidea). British Journal of Entomology and Natural History 5(5): 107–128.

Chandler PJ (1993) The Holarctic species of the Mycetophila fungorum (De Geer) group (Diptera: Mycetophilidae). British Journal of Entomology and Natural History 6(1): 5–11.

Chandler PJ (1998) Checklists of Insects of the British Isles (New Series) Part 1: Diptera. Handbooks for the Identification of British Insects Royal Entomological Society, London, 1–234 pp. `http://www.dipteristsforum.org.uk/sgb\_check\_intro.php`.

Chandler PJ (2001a) Fungus gnats (Diptera: Sciaroidea) new to Britain. British Journal of Entomology and Natural History 13: 215–243. `http://www.online-keys.net/sciaroidea/2000\_/Chandler\_2001\_Fungus\_gnats\_new\_to\_Brittain.pdf`.

Chandler PJ (2001b) The Flat-footed Flies (Diptera: Opetiidae and Platypezidae) of Europe. Fauna Entomologica Scandinavica E. J. Brill, Leiden, 276 pp.

Chandler PJ (2002) Heterotricha Loew and allied genera (Diptera: Sciaroidea): Offshoots of the stem group of Mycetophilidae and/or Sciaridae? Annales de la Société Entomologique de France 38(1-2): 101–144. `http://www.online-keys.net/sciaroidea/2000\_/Chandler\_2002\_Heterotrichia\_group\_sp\_n.pdf`.

Chvála M (1971) A Revision of the Scandinavian Tachydromiinae (Dipt., Empididae) described by J. W. Zetterstedt. Entomologica scandinavica 2(1): 1–28 doi:10.1163/187631271X00013.

Chvála M (1972) Notes on Scandinavian Platypalpus (Dipt., Empididae) with Description of Four New Species and New Synonymies. Entomologica scandinavica 3(1): 1–11 doi:10.1163/187631272X00012.

Chvála M (1973) European species of the Platypalpus albiseta-group (Deptera, Empididae). Acta entomologica bohemoslovaca 70(2): 117–136.

Chvála M (1975) The Tachydromiinae (Dipt. Empididae) of Fennoscandia and Denmark. Fauna Entomologica Scandinavica Scandinavian Science Press Ltd, Klampenborg, 336 pp.

Chvála M (1983) The Empidoidea (Diptera) of Fennoscandia and Denmark. II. General Part. The Families Hybotidae, Atelestidae and Microphoridae. Fauna Entomologica Scandinavica, vol. 12 Scandinavian Science Press Ltd, Klampenborg, 279 pp. ISBN 8787491079.

Chvála M (1989) Monograph of Northern and Central European Species of Platypalpus (Diptera, Hybotidae), with Data on the Occurrence in Czechoslovakia. Acta Universitatis CarolinaeBiologica 32: 209–376.

Chvála M (1991) Empididae (Diptera) of Finland: The Empis subgenera Empis and Coptophlebia. Entomologica fennica 2(2): 79–86.

Chvála M (1994) The Empidoidea (Diptera) of Fennoscandia and Denmark, Part III: Genus Empis. Fauna Entomologica Scandinavica E.J. Brill, Leiden, New York, Köln, 192 pp. ISBN 9004096639.

Chvála M (1996) A taxonomic revision of the Hilara maura-group (Diptera, Empididae) in Europe. Systematic Zoology 21: 265–294 doi:10.1111/j.1365-3113.1996.tb00601.x.

Chvála M (2002a) Revision of European species of the Hilara ”quadrivittata” group (Diptera: Empididae). Acta Universitatis CarolinaeBiologica 46: 229–276.

Chvála M (2002b) The Hilara species described by L. Oldenburg, with description of a new species from Lapland (Diptera: Empididae). Entomologica fennica 13(2): 65–78.

Chvála M (2005) The Empidoidea (Diptera) of Fennoscandia and Denmark. IV. Genus Hilara. Fauna Entomologica Scandinavica Brill NV, Leiden, 233 pp.

Chvála M, Biström O (1991) Bicellaria austriaca Tuomikoski, new for the Finnish fauna (Diptera, Hybotidae). Entomologica fennica 2(1): 3.

Chvála M, Kovalev VG (1989) Family Hybotidae. In: Soós A, Papp L (Eds) Catalogue of Palaearctic Diptera. Therevidae-Empididae. Volume 6. Akademiai Kiado, Budapest & Elsevier, Amsterdam, 174–227.

Chvála M, Wagner R (1989) Family Empididae. In: Soós A, Papp L (Eds) Catalogue of Palaearctic Diptera. Therevidae-Empididae. Volume 6. Akademiai Kiado, Budapest & Elsevier, Amsterdam, 228–336.

Clastrier J (1991) Description de Forcipomyia (Dycea) madeira n. sp. de l’Ille de Madère, et présence en Finlande de F. (Caloforcipomyia) glauca Macfie (Dipt. Ceratopogonidae). Bulletin de la Societe Entomologique de France 96(3): 261–266.

Claussen C, Torp E (1980) Untersuchungen über vier europäische Arten der Gattung Anasimyia Schiner, 1864 (Insecta, Diptera, Syrphidae). Mitteilungen aus dem Zoologischen Museum der Universitat Kiel 1(4): 1–16.

Clements DK, Stuke JH, Chandler PJ (2008) Conops testacea Linnaeus, 1767 (currently Myopa testacea)(Insecta, Diptera): proposed conservation of usage of the specific name by the designation of. Bulletin of Zoological Nomenclature 65(4): 294–299.

Cogan BH (1984) Family Ephydridae. In: Soós A, Papp L (Eds) Catalogue of Palaearctic Diptera. Clusiidae-Chloropidae. Volume 10. Catalogue of Palaearctic Diptera Akademiai Kiado, Budapest & Elsevier, Amsterdam, 126–176 ISBN 0-444-99601-X.

Cook EF (1965) A Contribution Toward a Monograph of the Scatopsidae. Part IX. The Genera Aspistes and Arthria (Diptera: Scatopsidae: Aspistinae). Annals of the Entomological Society of America 58(5): 713–721.

Cook EF (1969) A synopsis of the Scatopsidae of the Palaearctic Part I. Rhegmoclematini. Journal of Natural History 3(3): 393–407 doi: 10.1080/00222936900770341. `http://www.tandfonline.com/doi/abs/10.1080/00222936900770341`.

Cook EF (1972) A synopsis of the Scatopsidae of the Palaearctic Part II. Swammerdamellini. Journal of Natural History 6(6): 625–634 doi: 10.1080/00222937200770561. `http://www.tandfonline.com/doi/abs/10.1080/00222937200770561`.

Crosskey RW (2004) World Inventory of Simuliidae 2004. `http://blackflies.info/sites/blackflies.info/files/u13/Inventory.pdf`.

Dahl C (1968) Notes on the taxonomy and distribution of Trichoceridae (Dipt. Nemat.) from Finland and adjacent areas of the USSR. Opuscula Entomologica 33(3): 365–370.

Dahl C (1992) Family Trichoceridae (Petauristidae). In: Sóos A, Papp L, Oosterbroek P (Eds) Catalogue of Palaearctic Diptera. Trichoceridae - Nymphomyiidae. Volume 1. Hungarian Natural History Museum, Budapest, 31–37 ISBN 9637093109.

Dahl C, Alexander CP (1976) A world catalogue of Trichoceridae Kertész, 1902 (Diptera). Entomologica scandinavica 7(1): 7–18 doi:10.1163/187631276X00027.

De Meyer M, Backeljau T (1990) Taxonomical and geographical notes on the genus Cephalops Fallén, 1810 (Diptera, Pipunculidae) in Fennoscandia and Denmark. Notulae Entomologicae 69: 199–202.

Decleer K (1989) Sphaerophoria chongjini Bankowska, 1964 (Diptera Syrphidae) recorded for the first time in Belgium and its distribution in Europe. Bulletin et Annales de la Societe Royale Belge d'Entomologie 125: 152–155.

Delécolle JC, Brummer-Korvenkontio M, Kremer M (1983) Culicoides (Diptera, Ceratopogonidae) new to the Finnish fauna light trapped in the Tvärminne area (Hanko, Southern Finland). Notulae Entomologicae 63(2): 88–89.

Dely-Draskovits A (1993) Family Anthomyiidae. In: Soós A, Papp L (Eds) Catalogue of Palaearctic Diptera. Anthomyiidae-Tachinidae. Volume 13. Hungarian Natural History Museum, Budapest, 11–102 ISBN 9637093214.

Disney RHL (1980) What is Conicera minuscula Schmitz. Entomologist's Gazette 31: 202.

Disney RHL (1981) A further synonym in the genus Conicera Meigen with a revised list of the British species. Entomologist's Record and Journal of Variation 93: 126–128.

Disney RHL (1984) The holotype of Megaselia minor (Zett.) (Dipt., Phoridae) and two new synonyms. Entomologist's Monthly Magazine 120: 239–240.

Disney RHL (1985) Additions and amendments to the list of British Megaselia (Dipt., Phoridae). Entomologist's Monthly Magazine 121: 243–248.

Disney RHL (1988) Three new species of Megaselia (Dipt., Phoridae) from the British Isles, plus new synonyms. Entomologist's Monthly Magazine 124(January-April): 7–14.

Disney RHL (1989) Scuttle Flies - Megaselia. Handbooks for the Identification of British Insects, vol. 10, part 8 Royal Entomological Society of London, London, 156 pp.

Disney RHL (1991) Family Phoridae. In: Soós A, Papp L (Eds) Catalogue of Palaearctic Diptera. Dolichopodidae-Platypezidae. Volume 7. Akademiai Kiado, Budapest & Elsevier, Amsterdam, 143–204 ISBN 9630557630.

Disney RHL (2009) Scuttle flies (Diptera: Phoridae) reared from logs in Finland and NW Russia, including two new species. Entomologica fennica 20(4): 257–267. http://www.cabdirect.org/abstracts/20103109900.html.

Disney RHL (2011) A new species of Megaselia Rondani (Dipt., Phoridae) from Finland. Entomologist's Monthly Magazine 147: 49–53.

Disney RHL (2012) Scuttle flies (Diptera: Phoridae) caught at dead mice in SW Finland. Zootaxa 3298: 30–42. http://www.mapress.com/zootaxa/2012/f/z03298p042f.pdf.

Dušek J, Láska P (1976) European species of Metasyrphus: key, descriptions and notes (Diptera, Syrphidae). Acta entomologica bohemoslovaca 73(4): 263–282.

Dušek J, Láska P (1982) European species related to Platycheirus manicatus, with description of two new species (Diptera, Syrphidae). Acta entomologica bohemoslovaca 79(5): 377–392.

Flinck J, Kahanpää J (2013) Suomelle uusia kärpäslajeja (Diptera: Brachycera). Sahlbergia 19(1-2): 53–62.

Frey R (1908) Beiträge zur Kenntnis der Dipteren-Fauna Finlands. Meddelanden af Societas pro Fauna et Flora Fennica 33[1906-7]: 67–69.

Frey R (1913) Zur kenntnis der Dipterenfauna Finlands. II. Empididae. Acta Societatis pro Fauna et Flora Fennica 37(3): 1–88. `http://www.biodiversitylibrary.org/page/5623721`.

Frey R (1915) Zur kenntnis der Dipterenfauna Finlands. III. Dolichopodidae. Acta Societatis pro Fauna et Flora Fennica 40(5): 1–80+3 pl. `http://www.biodiversitylibrary.org/page/5571884`.

Frey R (1921) Beschreibung einer neuen Pedicia-Art aus dem nordlichen Europa (Diptera,Tipulidae). Notulae Entomologicae 1: 110–112.

Frey R (1927) För Finlands fauna nya Dolichopodider och Empidider. Memoranda Societatis pro Fauna et Flora Fennica 1[1924-25]: 38–39.

Frey R (1928) Förteckning över Finlands Clusiider (Dipt., Haplost.). Notulae entomologicae 8: 108–109.

Frey R (1930) Neue Diptera brachycera aus Finnland und angrenzenden Ländern. Notulae entomologicae 10(3-4): 82–94.

Frey R (1932a) Aedes sticticus Meigen, ny för Finland. Notulae entomologicae 12: 64–65.

Frey R (1932b) Neue Diptera brachycera aus Finnland und angrenzenden Ländern. II. Notulae entomologicae 12(4): 81–85.

Frey R (1935) Neue Diptera brachycera aus Finnland und angrenzenden Ländern. III. Notulae entomologicae 15(4): 97–101.

Frey R (1937) Finlands Agromyzider, bestämda av Fr. Hendel. Notulae Entomologicae 17(3): 82–96.

Frey R (1940) Föreningsmeddelanden - Tietoja yhdistyksistä. Notulae entomologicae 20(1): 20.

Frey R (1946) Anteckningar om Finlands agromyzider. Notulae Entomologicae 26: 13–55.

Frey R (1947) Anteckningar om dipterafaunan påKarlö (Hailuoto) sommaren 1947. Memoranda Societatis pro Fauna et Flora Fennica 24: 69–80. `https://helda.helsinki.fi/handle/10138/37244`.

Frey R (1948) Entwurf einer neuen Klassifikation der Mückenfamilie Sciaridae (Lycoriidae). II. Die nordeuropäischen Arten. Notulae entomologicae 27[1947](2-4): 33–112.

Frey R (1950) Dipterfaunan vid Tana älv i Utsjoki sommaren 1949. Notulae entomologicae 30(1-2): 5–18.

Frey R (1952) Über Chiropteromyza n. gen. und Pseudopomyza Strobl. (Diptera Haplostomata). Notulae entomologicae 32: 5–8.

Frey R (1956) Studien über ostasiatische Dipteren V. Psilidae, Megamerinidae. Notulae entomologicae 35[1955]: 122–137.

Frey R, Tiensuu L, StoråR (1941) Enumeratio Insectorum Fenniae. VI. Diptera. Helsingin hyönteisvaihtoyhdistys r.y., Helsinki, 63 pp.

Gagné RJ (1981) A monograph of Trichonta with a model for the distribution of Holarctic Mycetophilidae (Diptera). United States Department of Agriculture Technical Bulletin 1638: 1–64. `http://naldc.nal.usda.gov/download/CAT81766129/PDF`.

Geiger W (1985) Limoniidae (Diptera), sous-famille Limoniinae de Suisse: synonymies nouvelles. Mitteilungen der Schweizerischen Entomologischen Gesellschaft 58(1): 69–76.

Geiger W (1986) Bibliographie raisonnee des Limoniidae (Diptera Nematocera) de la zone Palaearctique ouest. (Publications 19001982). 1. Sous-famille Limoniinae. Bulletin Romand dEntomologie 4: 3–121.

Gerbachevskaja-Pavluchenko AA (1986) Family Sciaridae. In: Soós A, Papp L (Eds) Catalogue of Palaearctic Diptera. Sciaridae-Anisopodidae. Volume 4. Akademiai Kiado, Budapest & Elsevier, Amsterdam, 11–72 ISBN 0-444-99529-3.

Gika W (2005) A Systematic Review of European Stempellina Thienemann et Bause, 1913 (Diptera: Chironomidae) with Description of a New Species from Fennoscandia. Annales Zoologici (Warszawa) 55(3): 413–419.

Gika W, Paasivirta L (2008) On the systematics of the tribe Tanytarsini (Diptera: Chironomidae) - three new species from Finland. Entomologica fennica 19(1): 41–48.

Gika W, Paasivirta L (2009) Evaluation of diagnostic characters of the Tanytarsus chinyensis group (Diptera: Chironomidae), with description of a new species from Lapland. Zootaxa 2197: 31–42.

Gorodkov KB (1984) Family Heleomyzidae (Helomyzidae). In: Soós A, Papp L (Eds) Catalogue of Palaearctic Diptera. Clusiidae-Chloropidae. Volume 10. Akademiai Kiado, Budapest & Elsevier, Amsterdam, 15–45 ISBN 044499601X.

Gorodkov KB (1986) Family Scathophagidae. In: Soós A, Papp L (Eds) Catalogue of Palaearctic Diptera. Scathophagidae-Hypodermatidae. Volume 11. Akademiai Kiado, Budapest & Elsevier, Amsterdam, 11–41.

Griffiths GCD (1964) The agromyzid fauna of Iceland and the Faeroes, with appendices on the Phytomyza milii and robustella groups (Diptera, Agromyzidae). Entomologiske Meddelelser 32(5): 393–450.

Griffiths GCD (1967) Revision of the Phytomyza syngenesiae group (Diptera, Agromyzidae), including species hitherto known as ’Phytomyza atricornis Meigen’. Stuttgarter Beiträge zur Naturkunde A 177: 1–28.

Griffiths GCD (1980) Studies on boreal Agromyzidae (Diptera) XIV. Chromatomyia miners on Monocotyledones. Entomologica scandinavica Supplement 13: 1–61.

Griffiths GCD (1983) Cyclorrhapha II (Schizophora: Calyptratae) Anthomyiidae No. 2. In: Griffiths GCD (Ed) Flies of the Nearctic Region. vol. 8, 2(3) E. Schweizerbart’sche Verlagsbuchhandlung, Stuttgart, 161–228.

Gross J, Fatouros NE, Neuvonen S, Hilker M (2004) The importance of specialist natural enemies for Chrysomela lapponica in pioneering a new host plant. Ecological Entomology 29(5): 584–593 doi:10.1111/j.0307-6946.2004.00632.x.

Grunin KJ (1972) Beschreibung einer ornithoparasitischen Fliege, Protocalliphora nuortevai Grunin, sp. n. (Dipt., Calliphoridae) aust Nord-Finnland. Annales Entomologici Fennici 38(3): 156–158.

Haarto A (1995) Five species of flies (Diptera) new to Finland, with notes on additional rare species. Entomologica fennica 6(1): 5–6.

Haarto A (1997) Short reports, Taxa new for Finland. Entomologica fennica 8(1): 1–2.

Haarto A (1998) Taxa new to Finland - Diptera. Entomologica fennica 9(1): 1.

Haarto A (1999) Short reports. Taxa new to Finland. Diptera. Entomologica fennica 10(1): 1–2.

Haarto A (2000) Short reports. Taxa new for Finland. Diptera. Entomologica fennica 11(1): 1.

Haarto A (2002a) Kaksi kukkakärpästen (Diptera, Syrphidae) keräysretkeä Lappiin Kilpisjärven takatuntureille. Sahlbergia 7(1): 26–32.

Haarto A (2002b) Species new to the Finnish fauna - Diptera. Sahlbergia 7(1): 35–36.

Haarto A (2009) 3.2 Kaksisiipiset (Diptera). In: Hyvärinen E, Sulkava P (Eds) Hyönteiskartoitukset Annjaloanjilla ja Toskaljärven ympäristössä Käsivarren erämaa-alueella 2007 ja 2008. No. 185 In Metsähallituksen luonnonsuojelujulkaisuja, Sarja A Metsähallitus, Helsinki, 21–25.

Haarto A (2011) Suomen ikkunasääsket Finnish window gnats (Diptera, Anisopodidae). w-album 10: 3–13.

Haarto A (2012) Suomen karvasääsket ja maasääsket (Diptera, Bibionidae ja Pleciidae). w-album 12: 3–31.

Haarto A, Kahanpää J (2013) Notes on Finnish Sphaeroceridae (Diptera) with description of female of Minilimosina tenera Rohacek, 1983. Entomologica fennica 24(4): 228–233.

Haarto A, Kerppola S (2004) Checklist of Finnish Hoverflies (Diptera, Syrphidae). Sahlbergia 9(2): 147–164.

Haarto A, Kerppola S (2007a) Muutoksia Suomen kukkakärpäsfaunaan (Diptera, Syrphidae). Sahlbergia 13: 34–40.

Haarto A, Kerppola S (2007b) Suomen kukkakärpäset ja lähialueiden lajeja. Suomen ympäristöministeriö, Helsinki, 647 pp. ISBN 978-952-11-2568-3.

Haarto A, Kerppola S (2009) Brachyopa zhelochovtsevi Mutin, 1998 Euroopasta ja muita lisäyksiä Suomen kukkakärpäsfaunaan (Diptera, Syrphidae). w-album 7: 3–11.

Haarto A, Kerppola S, Stå hls G (2007) Description of Cheilosia naruska Haarto & Kerppola spec. nov. from northern Europe (Diptera, Syrphidae). Volucella 8: 63–72.

Haarto A, Mukkala VM (2013) Korppoon Utön hyönteisistä ja muista eläimistä. w-album 13: 3–35.

Haarto A, Mukkala VM, Koponen S (2002) Tutkimus Rekijokilaakson hyönteisistä ja hämähäkkieläimistä. Lounais-Suomen ympäristökeskuksen monistesarja 2002(5): 1–58.

Haarto A, Winqvist K (2014) Muutoksia Suomen kaksisiipisfaunassa (Insecta: Diptera). w-album 16: 3–19.

Hackman W (1954) Die Drosophila-Arten Finnlands. Notulae entomologicae 34: 130–139.

Hackman W (1955) On the genera Scaptomyza Hardy and Parascaptomyza Duda (Dipt., Drosophilidae). Notulae entomologicae 35(3): 74–91.

Hackman W (1956) The Lonchaeidae (Dipt.) of Eastern Fennoscandia. Notulae entomologicae 36: 89–115.

Hackman W (1961) Addendum [to Basden: Notes on the Camillidae (Diptera)]. Notulae Entomologicae 41(4): 129.

Hackman W (1963) Studies on the dipterous fauna in burrows of voles (Microtus, Clethrionomys) in Finland. Acta Zoologica Fennica 102: 1–64. https://helda.helsinki.fi/bitstream/handle/10138/37756/299947_102_1963.pdf?sequence=1.

Hackman W (1965) On the genus Copromyza Fall. (Dipt., Sphaeroceridae), with special reference to the finnish species. Notulae entomologicae 45: 33–46.

Hackman W (1968a) Kokousselostuksia Sitzungsberichte. Annales Entomologici Fennici 34(2): 109–112.

Hackman W (1968b) Mötesreferat. - Kokousselostuksia. Notulae entomologicae 48(4): 242–248.

Hackman W (1968c) On the subgenus Opacifrons Duda of the genus Leptocera Olivier (Diptera, Sphaeroceridae) with a description of a new species from Northern Fennoscandia. Notulae entomologicae 48: 40–44.

Hackman W (1969) A new Drosophila species from Northern Fennoscandia (Diptera). Notulae Entomologicae 49: 69–72.

Hackman W (1970a) New species of the genus Phronia Winnertz (Diptera, Mycetophilidae) from Eastern Fennoscandia and notes on the synonymies in this genus. Notulae Entomologicae 50: 41–60.

Hackman W (1970b) Trixoscelidae (Diptera) from Southern Spain and Description of a New Trixoscelis Species from Northern Europe. Entomologica scandinavica 1(2): 127–134 doi:10.1163/187631270X00168.

Hackman W (1971a) Sieni- ja surusääskiä (Diptera, Mycetophiloidea) Lounais-Hämeestä. Lounais-Hämeen Luonto 43: 59–61. `http://www.lounaisluonto.net/julkaisut/LHL/LHL43.pdf`.

Hackman W (1971b) Three new species of the Allodia lugens group from northern Europe (Diptera, Mycetophilidae). Annales Entomologici Fennici 37(1): 3–7.

Hackman W (1972a) Drosophila lummei sp. n., a new species close to D. littoralis Meigen (Diptera, Drosophilidae). Notulae Entomologicae 52: 89–92.

Hackman W (1972b) Mötesreferat - Kokousselostuksia. Notulae Entomologicae 52(4): 140–144.

Hackman W (1972c) Phronia matilei n. sp., a new fungus gnat from caves in Switzerland (Diptera, Mycetophilidae). Notulae Entomologicae 52: 39–40.

Hackman W (1976) Communications at the meetings of Societies. Annales Entomologici Fennici 42(3): 146–148.

Hackman W (1977) Brief reports. Diploneura cornuta Bigot (Phoridae) ny för Finland. Notulae Entomologicae 57(2): 63–64.

Hackman W (1979a) Brief reports. Allodia (Brachycampta) mendli Plassmann (Mycetopholidae), ny för Finland. Notulae entomologicae 59(4): 172.

Hackman W (1979b) Brief reports. Pegomya pallidoscutellata (Zetterstedt) ny för Finland (Anthomyiidae). Notulae Entomologicae 59(4): 172–173.

Hackman W (1980) A Check List of the Finnish Diptera. Notulae entomologicae 60: 17–48,117–162.

Hackman W (1981) För Finland nya flugor samt tre övriga tillägg till förtäckningen över Finlands Diptera. Notulae entomologicae 61(4): 225–226.

Hackman W (1982) Tillägg och rättelser till förteckingen över Finlands Diptera. Notulae Entomologicae 62: 158–159.

Hancock EG (2008) Larval habitat preferences in Palaearctic Gnophomyia (Diptera, Limoniidae) with a key to adults. Sahlbergia 14: 13–16.

Hanski I, Nuorteva P (1975) Trap survey of flies and their diel periodicity in the subarctic Kevo Nature Reserve, northern Finland. Annales Entomologici Fennici 41(2): 56–64.

Hedmark K (1998) Svampmyggor nya arter för Sverige och Finland (Diptera: Mycetophilidae s. lat.). Entomologisk Tidskrift 119(1): 1–12.

Hedmark K (2000) Fungus gnats in the taiga new species from Sweden in a Fennoscandian perspective (Diptera: Sciaroidea excl. Sciaridae). Entomologisk Tidskrift 3(121): 73–89.

Hedström L (1964) Lounais-Hämeen kärpäsiä, Brachycera (Diptera). Lounais-Hämeen Luonto 15: 77–89. www.lounaisluonto.net/julkaisut/LHL/LHL27.pdf.

Hedström L (1968) Muscidae s. str. (Diptera) from South-West Häme. Lounais-Hämeen Luonto 27: 1–13. http://www.lounaisluonto.net/julkaisut/LHL/LHL27.pdf.

Hedström L (1969) Diptera Orthorrhapha from South-West Häme Part I. Lounais-Hämeen Luonto 32: 1–6. http://www.lounaisluonto.net/julkaisut/LHL/LHL32.pdf.

Hedström L (1990) Svenska insektfynd rapport 6. Entomologisk Tidskrift 111(4): 133–147.

Hedström L (1991) Svenska insektfynd - rapport 7. Entomologisk Tidskrift 112: 133–146.

Hedström L, Nuorteva P (1971) Zonal distribution of flies on the hill Ailigas in subartic northern Finland. Annales Entomologici Fennici 37(2): 121–125.

Hellén W (1940) Sphegina Kimakoviczi Strobl (Dipt.), ny för Nordeuropa. Notulae Entomologicae 20: 74.

Hellén W (1946) Verzeichnis der in den Jahren 19411945 für die Fauna Finnlands neuhinzugekommenen Insektenarten. Notulae Entomologicae 26: 122–142.

Hellén W (1952) Verzeichnis der in den Jahren 19461950 für die Fauna Finnlands neuhinzugekommenen Insektenarten. Notulae entomologicae 32: 59–80.

Heller K (2012) Fünf neue europäische Arten von Trauermücken (Diptera, Sciaridae) aus dem Artenkreis um Leptosciarella pilosa. Veröffentlichungen des Museums für Naturkunde Chemnitz 36: 91–102.

Heller K, Menzel F (2013) Drei neue Trauermückenarten aus Mitteleuropa (Diptera: Sciaridae). Ferrantia 69: 337–348. http://ps.mnhn.lu/ferrantia/publications/Ferrantia69/Ferrantia69337-348.pdf.

Heller K, Vilkamaa P, Hippa H (2009) An annotated check list of Swedish black fungus gnats (Diptera, Sciaridae). Sahlbergia 15(1): 23–51.

Herting B, Dely-Draskovits A (1993) Family Tachinidae. In: Soós A, Papp L (Eds) Catalogue of Palaearctic Diptera. Anthomyiidae-Tachinidae. Volume 13. Akademiai Kiado, Budapest & Elsevier, Amsterdam, 118–458 ISBN 9637093214.

Hildén M, Silfverberg H, Talman R (2010) Accessions to the Zoological Museum of the Finnish Museum of Natural History, University of Helsinki in 2009. Memoranda Societatis pro Fauna et Flora Fennica 86: 86–89. http://ojs.tsv.fi/index.php/msff/article/view/3973/3727.

Hippa H (1967) A redescription of Neoascia aenea Meig. Annales Entomologici Fennici 33(2): 113–117.

Hippa H (1972) Some species of Syrphidae (Diptera) new to the Finnish fauna. Annales Entomologici Fennici 38(4): 187–189.

Hippa H, Nielsen TR, van Steenis J (2001) The West Palearctic species of the genus Eristalis Latreille (Diptera: Syrphidae). Norwegian Journal of Entomology 48(2): 289–327. http://www.entomologi.no/journals/nje/2001-2/abs/48_289.pdf.

Hippa H, Vilkamaa P (1991) The Genus Prosciara Frey (Diptera, Sciaridae). Entomologica fennica 2(3): 113–155.

Hippa H, Vilkamaa P (1994) The genus Camptochaeta gen.n. (Diptera, Sciaridae). Acta Zoologica Fennica 194: 1–85.

Hippa H, Vilkamaa P (2005) Cladistic analysis finds a placement for an enigmatic species, Peyerimhoffia sepei sp. n. (Diptera: Sciaridae), with a note on its spermatophore. Zootaxa 1044: 49–55.

Hippa H, Vilkamaa P, Heller K (2010) Review of the Holarctic Corynoptera Winnertz, 1867, s. str. (Diptera, Sciaridae). Zootaxa 2695: 1–197.

Hippa H, Vilkamaa P, Mohrig W (2003) Phylogeny of Corynoptera Winnertz and related genera, with the description of Claustropyga gen. nov. (Diptera, Sciaridae). Studia dipterologica 9[2002](2): 469–511.

Hirvenoja M (1961) Weitere Studien über Chaoborinen (Dipt., Culicidae). Beschreibung der Larve und der Puppe von Chaoborus (Schadonophasma) nyblaei Zett. Annales Entomologici Fennici 27(2): 77–83.

Hirvenoja M (1962a) Cladotanytarsus-Arten (Dipt., Chironomidae) aus Finnisch-Lappland. Annales Entomologici Fennici 28(4): 173–181.

Hirvenoja M (1962b) Ein Vergleich der Culiciden-Fauna einiger süd- und nordfinnischen Schmelzwasserlachen. Annales Entomologici Fennici 28(3): 97–107.

Hirvenoja M (1962c) Materialen zur Kenntnis der Gattung Chironomus (Dipt.). Annales Entomologici Fennici 28(2): 63–67.

Hirvenoja M (1962d) Zur Kenntnis der Gattung Chironomus (Dipt., Chironomidae). Annales Entomologici Fennici 28(2): 85–88.

Hirvenoja M (1962e) Zur Kenntnis der Gattung Polypedilum Kieff. (Dipt., Chironomidae). Annales Entomologici Fennici 28(3): 127–136.

Hirvenoja M (1963a) Microtendipes nigellus n.sp. (Dipt., Chironomidae). Annales Entomologici Fennici 29(4): 247–249.

Hirvenoja M (1963b) Tanytarsus aptus n.sp. (Dipt., Chironomidae). Annales Entomologici Fennici 29(1): 50–52.

Hirvenoja M (1963c) Taxonomy, biology and biometry of Tanytarsus curticornis Kieff. and. T. brundini n.sp. (Dipt., Chironomidae). Annales Entomologici Fennici 29(2): 118–130.

Hirvenoja M (1973) Revision der Gattung Cricotopus van der Wulp und ihrer Verwandten (Diptera, Chironomidae). Annales Zoologici Fennici 10(1): 1–363.

Hirvenoja M (1989) Cricotopus (Isocladius) polychaetus, spec. nov. and designations of some other type specimens of the genus Cricotopus v. d. Wulp (Diptera, Chironomidae). Sphixiana 12: 275–278. http://biostor.org/reference/52465.

Hirvenoja M (1998a) Chironomus coaetaneus sp.n. (Diptera, Chironomidae) from the Lokka Reservoir, northern Finland. Oulanka reports 18: 53–61.

Hirvenoja M (1998b) The biodiversity of the fauna in some shore pools of the Lokka Reservoir in northern Finland with descriptions or redescriptions of the species of Chironomus Meigen (Dipt., Chironomidae) found in them. Oulanka reports 18: 37–52.

Hirvenoja M (2002) The fauna in two cold springs and in an epirhithral pool in southern Finland. Sahlbergia 7(1): 7–25. http://poronoro.fi/library/PDF/Hirvenoja_Springs.pdf.

Hirvenoja M, Hirvenoja E (1988) Corynoneura brundini spec. nov. Ein Beitrag zur Systematik der Gattung Corynoneura. Spixiana Supplement 14: 213–238. http://www.biodiversitylibrary.org/page/30082829.

Hirvenoja M, Michailova P (1991) The Karyotype, Morphology and Ecology of Glyptotendipes aequalis Kieffer (Diptera, Chironomidae). Entomologica fennica 2(2): 87–96. http://www.entomologicafennica.org/Volume02/abstracts2_87.htm.

Hirvenoja M, Michailova P (1998) The karyotype and morphology of Chironomus brevidentatus sp. n. (Dipt., Chironomidae). A species with a ”salinarius type” larva from northern Finland. Entomologica fennica 9(4): 225–236.

Huldén L (2004) Brief communications. Diptera. Sahlbergia 8[2003](2): 100.

Huldén L, Huldén L, Lahtinen T (2008) Bluetongue-viruksen vektorilajit Suomessa. Suomen eläinlääkärilehti 114(3): 158–161. http://urn.fi/URN:NBN:fi:ELE-1364876.

Ilmonen J, Adler PH (2008) Updated checklist of the black flies (Diptera: Simuliidae) of Finland. British Simuliid Group Bulletin 29: 9–15.

Ilmonen J, Adler PH, Malmqvist B, Cywinska A (2009) The Simulium vernum group (Diptera: Simuliidae) in Europe: multiple character sets for assessing species status. Zoological Journal of the Linnean Society 156(4): 847–863 doi:10.1111/j.1096-3642.2009.00500.x. http://doi.wiley.com/10.1111/j.1096-3642.2009.00500.x.

Ilmonen J, Kuusela K (2003) Simulium (Hellichiella) baffinense Twinn and Simulium (Hellichiella) crassum (Rubtsov), two new blackfly species for Finland (Diptera, Simuliidae). Sahlbergia 8: 89–91.

Ilmonen J, Kuusela K (2006) The black flies (Diptera, Simuliidae) recorded from Finland. Studia dipterologica Supplement 14: 60–66.

Irwin AG (1985) British Ochthera (Diptera, Ephydridae). Entomologist’s Monthly Magazine 121: 151–154.

Itämies J (1981) Aedes geniculatus Suomelle uusi hyttynen (Diptera, Culicidae). Notulae Entomologicae 61(1): 207–208.

Itämies J (1995) Chromatomyia linnaeae (Diptea, Agromyzidae) new to Finland. Entomologica fennica 6(4): 187–188.

Itämies J, Helle P, Hyytinen L (1976) Ornithophila metallica (Diptera, Hippoboscidae) a new bird-fly species in Fennoscandia. Notulae Entomologicae 56(4): 103–104.

Jakovlev J, Kjæ randsen J, Polevoi A (2006) Seventy species of fungus gnats new to Finland (Diptera: Mycetophilidae). Sahlbergia 11(1): 22–39.

Jakovlev J, Penttinen J (2007) Boletina dispectoides sp.n. and six other species of fungus gnats (Diptera: Mycetophilidae) new to Finland. Entomologica fennica 18(4): 211–217.

Jakovlev J, Polevoi A (2009) Two new species of the genus Phronia Winnertz (Diptera:Mycetophilidae) from Finland and Russian Karelia. Entomologica fennica 19(4): 199–206.

Jansson A, Lindeberg B (1982) A spectacular tephritid fly (Diptera) new to Finland. Notulae entomologicae 62: 151–152.

Jaschhof M, Jaschhof C (2009) The Wood Midges (Diptera: Cecidomyiidae: Lestremiinae) of Fennoscandia and Denmark. Studia dipterologica Supplement Ampyx-Verlag, Halle (Saale), VIII + 333 pp. ISBN 978-3-932795-32-9.

Jensen F (1997) Diptera Simuliidae, blackflies. In: Nilsson A (Ed) Aquatic insects of North Europe. A taxonomic handbook. Volume 2: Odonata - Diptera. Apollo Books, Steenstrup, 209–241.

Jervis MA (1992) A taxonomic revision of the pipunculid fly genus Chalarus Walker, with particular reference to the European fauna. Zoological Journal of the Linnean Society 105: 243–352. `http://onlinelibrary.wiley.com/doi/10.1111/j.1096-3642.1992.tb01232.x/abstract`.

Kahanpää J (2006) First update to the checklist of Finnish Dolichopodidae (Diptera). International Journal of Dipterological Research 17(2): 121–125.

Kahanpää J (2007a) A review of Finnish Thecophora Rondani, 1845 (Diptera: Conopidae). Entomologica fennica 18: 168–173.

Kahanpää J (2007b) Rhaphium latimanum spec. nov., a new long-legged fly from northern Finland, with a redescription of Rhaphium patellitarse (Becker, 1900) (Diptera: Dolichopodidae). International Journal of Dipterological Research 18(4): 227–233.

Kahanpää J (2008) Nematode-induced demasculinisation of Dolichopus males (Diptera, Dolichopodidae). Zootaxa 1689: 51–62.

Kahanpää J (2010) Finnish species of Nemotelus (Diptera: Stratiomyidae), with description of a new species. Zootaxa 2401: 30–40. `http://www.mapress.com/zootaxa/2010/f/z02401p040f.pdf`.

Kahanpää J (2013a) A second update to the checklist of Finnish long-legged flies (Diptera: Dolichopodidae), with a re-evaluation of the status of Hydrophorus callosoma Frey, 1915. Biodiversity Data Journal e976: 1–34 doi:10.3897/BDJ.1.e976. `http://biodiversitydatajournal.com/articles.php?id=976`.

Kahanpää J (2013b) First record of the genus Chvalaea Papp & Földvári from Northern Europe (Diptera: Hybotidae). Zootaxa 3716(4): 592–594.

Kahanpää J (2013c) Muutoksia Suomen kärpästen luetteloon: heimo Lauxaniidae (Diptera). Sahlbergia 19(1-2): 72–78.

Kahanpää J (2013d) Viisikymmentä Suomelle uutta kärpäslajia (Diptera: Brachycera). Sahlbergia 19(1-2): 63–71.

Kahanpää J, Grichanov IY (2004) A check-list of Finnish long-legged flies (Diptera: Dolichopodidae). International Journal of Dipterological Research 15(1): 57–62.

Kahanpää J, Grichanov IY (2006) Dolichopodidae (Diptera) new for the fauna of Finland. Entomologica fennica 17(2): 73–78.

Kahanpää J, Haarto A (2013) Notes on Fanniidae (Diptera) of Finland, with a description of the female of Fannia stigi Rognes, 1982. Entomologica fennica 24: 179–185.

Kahanpää J, Negrobov OP (2007) Dolichopus setiger Negrobov, 1973 (Diptera, Dolichopodidae): A long-legged fly new to Europe. Norwegian Journal of Entomology 54: 27–31. `http://www.entomologi.no/journals/nje/2007-1/pdf/NJE-54-kahanpaa.pdf`.

Kahanpää J, Salmela J (2007) Ohkolanjokilaakson Natura-alueen kaksisiipisistä (Diptera). Sahlbergia 12: 43–49.

Kahanpää J, Winqvist K (2003) Rare flies recorded in Finland during the year 2002 (Diptera). Sahlbergia 8: 92–95.

Kahanpää J, Winqvist K (2005) Check-list of Finnish flies: families Xylophagidae-Microphoridae. Sahlbergia 10(1): 10–27.

Kaisila J (1952) Insects from arctic mountain snows. Annales Entomologici Fennici 18(1): 8–25.

Kaisila J (1958) Kokousselostuksia Sitzungsberichte. Annales Entomologici Fennici 24(2): 89–96.

Kaisila J (1960) Kokousselostuksia Sitzungsberichte. Annales Entomologici Fennici 26(4): 291–299.

Kameneva EP (2008) New and Little-Known Ulidiidae (Diptera, Tephritoidea) from Europe. Vestnik Zoologii 42(5): e45–e72 doi:10.2478/v10058-008-0010-6. `http://versita.metapress.com/openurl.asp?genre=article\&id=doi:10.2478/v10058-008-0010-6`.

Kanervo E (1928) Über die skandinavischen Dalmannia-Arten (Dipt., Conopidae). Notulae entomologicae 8(2): 36–38.

Kanervo V (1935) Eumerus tuberculatus Rond., maalle uusi laji sipulituholaisena. Annales Entomologici Fennici 1(3): 101–106.

Kanervo V (1938) Die Syrphidenfauna Sibiriens. Annales Entomologici Fennici 4(3): 147–170.

Kangas E (1935) Die Braunfleckigkeit des Birkenholzes und ihr urheber Dendromyza (Dizygomyza) betulae n. sp. Vorläufige Mitteilung. Communicationes Instituti forestalia Fenniae 22(1): 1–31.

Kangas E (1946) Kokousselostuksia Sitzungsberichte. Annales Entomologici Fennici 12(1): 31–43.

Kangas E (1949) Kokousselostuksia Sitzungsberichte. Annales Entomologici Fennici 15(4): 177–184.

Kangas E (1952) Kokousselostuksia Sitzungsberichte. Annales Entomologici Fennici 18(3): 147–152.

Kangas E, Käpylä M (1975) Communications at the meetings of Societies. Annales Entomologici Fennici 41(4): 136–142.

Karl O (1940) Neue paläarktische Musciden (Anthomyiiden) (Diptera). Stettiner Entomologische Zeitung 101: 41–47.

Karppinen E (1961) Kokousselostuksia - Sitzungsberichte. Annales Entomologici Fennici 27(4): 213–221.

Karppinen E (1962) Kokousselostuksia - Sitzungsberichte. Annales Entomologici Fennici 28(4): 190–192.

Karppinen E (1963) Kokousselostuksia - Sitzungsberichte. Annales Entomologici Fennici 29(3): 191–207.

Karppinen E (1964a) Kokousselostuksia - Sitzungsberichte. Annales Entomologici Fennici 30(4): 248–252.

Karppinen E (1964b) Kokousselostuksia - Sitzungsberichte. Annales Entomologici Fennici 30(1): 57–64.

Karppinen E (1965a) Kokousselostuksia - Sitzungsberichte. Annales Entomologici Fennici 31(2): 148–153.

Karppinen E (1965b) Kokousselostuksia Sitzungsberichte. Annales Entomologici Fennici 31(2): 148–153.

Karppinen E (1966) Kokousselostuksia - Sitzungsberichte. Annales Entomologici Fennici 32(2): 199–209.

Karppinen E (1967) Kokousselostuksia - Sitzungsberichte. Annales Entomologici Fennici 33(3): 269–283.

Karppinen E (1969a) Kokousselostuksia Sitzungsberichte. Annales Entomologici Fennici 35(4): 238–240.

Karppinen E (1969b) Kokousselostuksia Sitzungsberichte. Annales Entomologici Fennici 35(1): 59–62.

Karppinen E (1969c) Kokousselostuksia Sitzungsberichte. Annales Entomologici Fennici 35(2): 124–127.

Karvonen J (1969) On Finnish Tabanids (Diptera). Annales Entomologici Fennici 35(3): 176–183.

Karvonen J (1976) Communications at the meetings of Societies. Annales Entomologici Fennici 42(3): –146–.

Kauri H (1964) Über Bremsen (Tabanidae, Diptera) von Südwest-Häme, Finnland. Lounais-Hämeen Luonto 15: 39–45. http://www.lounaisluonto.net/julkaisut/LHL/LHL15.pdf.

Kauri H (1969) Über Bremsen (Tabanidae, Diptera) von Südwest-Häme, Finnland. II. Lounais-Hämeen Luonto 30: 1–4. http://www.lounaisluonto.net/julkaisut/LHL/LHL30.pdf.

Kehlmaier C (2005) Taxonomic revision of European Eudorylini (Insecta, Diptera, Pipunculidae). Verh. naturwiss. Ver. hamburg (NF) 42: 45–353.

Kehlmaier C (2006) The West-Palaearctic Species of Jassidophaga Aczél and Verrallia Mik Describe Up to 1966 (Diptera: Pipunculidae). Stuttgarter Beiträge zur Naturkunde A 697: 1–34. http://www.naturkundemuseum-bw.de/sites/default/files/publikationen/serie-a/a_697.pdf.

Kehlmaier C (2008) Finnish Pipunculidae (Diptera) studies Part I: Taxonomic notes on Cephalops Fallén, 1810, Pipunculus Latreille, 1802 and Tomosvaryella Aczél, 1939. Zootaxa 1672: 1–42. http://www.mapress.com/zootaxa/2008/f/z01672p042f.pdf.

Kehlmaier C, Assmann T (2008) The European species of Chalarus Walker, 1834 revisited (Diptera: Pipunculidae). Zootaxa 1936: 1–39. http://mapress.com/zootaxa/2008/f/z01936p039f.pdf.

Kehlmaier C, Stå hls G (2008) Finnish Pipunculidae (Diptera) studies Part II: an annotated checklist of Finnish big-headed flies. Sahlbergia 13: 44–68.

Kerppola S (2011) Hammerschmidtia ingrica Stackelberg, 1952 Inkerinmahlanen Suomesta (Diptera, Syrphidae). Sahlbergia 17(2): 2–4.

Kerppola S (2013) Muutoksia Suomen kukkakärpäsfaunaan (Diptera, Syrphidae). Sahlbergia 19(1-2): 30–38.

Kerppola S, Raekunnas M (2012) Blera eoa (Stackelberg, 1928) Siperiankantopuuhari Suomesta (Diptera, Syrphidae). Sahlbergia 18(1): 2–5.

Kjæ randsen J (2009) The genus Pseudexechia Tuomikoski re-characterized, with a review of European species (Diptera: Mycetophilidae). Zootaxa 2056: 1–45. `http://www.mapress.com/zootaxa/2009/f/z02056p045f.pdf`.

Kjæ randsen J (2012) Checklist of Nordic fungus gnats (Diptera: Bolitophilidae, Diadocidiidae, Ditomyiidae, Keroplatidae, Mycetophilidae and Sciarosoma). Version 1.0 2012.01.05. `http://sciaroidea.info/node/48341`.

Komonen A (1997) The parasitoid complexes attacking Finnish populations of two threatened butterflies, Euphydryas maturna and E. aurinia. Baptria 22(3): 105–109.

Komonen A (2001) Lyhyita tiedonantoja. Suomelle uusia lajeja. Diptera. Sciophila hebes Johansen (Mycetophilidae). Sahlbergia 6: 37. `http://www.online-keys.net/sciaroidea/add01/Komonen\_2001\_Sciophila\_hebes.pdf`.

Kontkanen P (1935) Trichieurina pubescens Meig. in Finnland Gefunden. Annales Entomologici Fennici 1(2): 60–62.

Koponen M (1976) Communications at the meetings of Societies. Annales Entomologici Fennici 42(3): 146.

Koskenniemi E, Paasivirta L (1987) The chironomid (Diptera) fauna in a Finnish reservoir during its first four years. In: Sæ ther OA (Ed) A Conspectus of Contemporary Studies in Chironomidae (Diptera). Contributions from the IXth Symposium on Chironomidae, Bergen, Norway. Entomologica Scandinavica Supplement Entomological Society of Lund, Lund, 239–246.

Kosonen L (1993) Tokunagayusurika jacutica - siperialainen surviaissääski. Diamina 2: 33–35.

Kovalev VG (1981) On European species of the group Lonchaea peregrina (Diptera, Lonchaeidae). Zoologicheskii Zhurnal 60: 221–228.

Kovalev VG (1984) On Finnish and some other species of Lonchaea Fallén (Diptera, Lonchaeidae). Annales Entomologici Fennici 50(1): 17–20.

Krivosheina MG (1998) A revision of the shore-fly genus Notiphila Fallén of Palaearctic (Diptera, Ephydridae). International Journal of Dipterological Research 9(1): 31–63.

Krivosheina MG (2000a) New records of shore flies (Diptera: Ephydridae). International Journal of Dipterological Research 11(4): 171–174.

Krivosheina MG (2004a) New data on shore flies of the genus Trimerina (Diptera, Ephydridae) in the palaearctic region with description of a new species from Finland. Zoologicheskii Zhurnal 83(5): 631–634.

Krivosheina NP (2000b) On the systematics of the species of the genera Colobostema Enderlein, Holoplagia Enderlein, and Cookella Freeman (Diptera, Scatopsidae) from Russia and neighbouring countries. Entomologicheskoe Obozrenie 79(2): 487–498.

Krivosheina NP (2001) Faunistic notes of Scatopsidae species (Diptera) from Russia and neighboring countries. Zoologicheskii Zhurnal 80(5): 545–555.

Krivosheina NP (2002) A Review of Palaearctic Species of the Genus Ectaetia Enderlein,. 1912 (Diptera, Scatopsidae) with Description of New Species. Entomologicheskoe Obozrenie 81(3): 726–736.

Krivosheina NP (2003) Morphology of species of the genus Temnostoma from apiforme and vespiforme groups. Report 1. Zoologicheskii Zhurnal 82(12): 1475–1486.

Krivosheina NP (2004b) Morphology of species of the genus Temnostoma from apiforme and vespiforme groups. Report 2. Zoologicheskii Zhurnal 83(1): 75–92.

Krivosheina NP, Menzel AF (2002) The morphology of females of the xylomycetophilous species of the genus Sylvicola Harris. Entomologicheskoe Obozrenie 81(2): 424–438.

Krivosheina NP, Rozkošný R (1990) Zabrachia stackelbergi sp. n., a new species of xylophilous soldier fly from eastern Asia (Diptera, Stratiomyidae). Acta entomologica bohemoslovaca 87(4): 304–313.

Krivosheina NP, Stå hls G (2003) Taxonomy and distribution of Temnostoma bombylans (Fabricius) and T. angustistriatum Krivosheina (Diptera, Syrphidae) in Northern Europe. Sahlbergia 8(2): 96–99.

Krogerus R (1932) Über die Ökologie und Verbreitung der Arthropoden der Triebsandgebiete an den Küsten Finnlands. Acta Zoologica Fennica 12: 1–308.

Krogerus R (1960) Ökologische Studien über nordische Moorarthropoden. Artenbestand, ökologische Faktoren, Korrelation der Arten. Commentationes Biologicae (Societas Scientiarum Fennicae) 21(3): 1–238.

Krzemiska E (2013) Fauna Europaea: Trichoceridae. http://www.faunaeur.org.

Kurina O (2004) Notes on fungus gnats from the Lemmenlaakso area in southern Finland, including six species new to the Finnish list (Diptera: Sciaroidea excl. Sciaridae). Sahlbergia 8: 84–88.

Kuusela K (1971) Preliminary notes on the blackfly species (Dipt., Simuliidae) of Finland. Annales Entomologici Fennici 37(4): 190–194.

Kuusela K (1979) Early summer ecology end community structure of the macrozoobenthos on stones in the Jäväjänkoski rapids on the river Lestijoki, Finland Ph.D. thesis University of Oulu.

Kuusela K (1981a) Cnephia (Metacnephia) tabescetifrons (Diptera, Simuliidae) from Finland a re-establishment of its status. Notulae Entomologicae 61: 159–160.

Kuusela K (1981b) Records of bloodsucking blackflies (Diptera: Simuliidae) on birds of prey (Aves: Falconiidae). Aquilo, Serie Zoologia 19: 77–80.

Kuusela K (1992) Blackflies (Diptera, Simuliidae) of the biological province of Koillismaa (Ks), NE Finland. Oulanka reports 10: 41–44.

Kuusela K, Adler PH (1994) Cytological identities of Simulium tuberosum and S. vulgare (Diptera: Simuliidae), with notes on other Palearctic members of the S. tuberosum species-group. Entomologica scandinavica 25(4): 439–446 doi:10.1163/187631294X00207.

Kuusela K, Wotton RS (1978) Simulium brevicaule and S. zetlandense, two blackfly species new to Finland (Diptera, Simuliidae). Notulae Entomologicae 58(4): 129–130.

Kuznetzov SY (1987) New data on systematics of Palearctic Syrphidae (Diptera). Entomologicheskoe Obozrenie 67(2): 419–435.

Kyrklund K (1980) Brief reports. Tvånya arter av fam. Cecidomyiidae för Finland. Notulae Entomologicae 60(4): 231.

Lakovaara S, Hackman W (1973) Drosophila ovivororum sp. n., a new species of the Drosophila virilis group (Diptera, Drosophilidae). Notulae Entomologicae 53: 167–169.

Lakovaara S, Itämies J (1994) Drosophila repleta Wollaston (Diptera, Drosophilidae) new to Finland. Entomologica fennica 5(3): 149–150.

Lakovaara S, Lankinen P (1974) Drosophila eskoi sp. n., a new species of the Drosophila obscura group (Diptera, Drosophilidae). Notulae Entomologicae 54(4): 122–124.

Lakovaara S, Saura A (1970) Isoenzymes of alcohol dehydrogenase in the species of the Drosophila obscura group. Annales Academiae Scientiarum Fennicae, Series A-IV (Biologica) 163: 1–10.

Laštovka P (1963) Beitrag zur Kenntnis der europäischen Fungivora-Arten aus der Gruppe vittipes (Zett.) (Dipt., Fungivoridae). Acta Societatis entomologicae cechosloveniae 60: 312–327.

Laštovka P, Máca J (1982) European and north American species of the genus Stegana (Diptera, Drosophilidae). Annotationes Zoologicae et Botanicae (Bratislava) 149: 1–38.

Lehmann J (1969) Die europäischen Arten der Gattung Rheocricotopus Thienemann & Harnisch und drei neue Artvertreter dieser Gattung aus der Orientalis (Diptera, Chironomidae). Archiv für Hydrobiologie 66: 348–381.

Lehmann J (1970a) Revision der europäischen arten (imagines und puppen) der gattung Rheotanytarsus Bause (Diptera, Chironomidae). Zoologischer Anzeiger 185: 345–378.

Lehmann J (1970b) Revision der Europäischen Arten (Imagines) der Gattung Parachironomus Lenz (Diptera, Chironomidae). Hydrobiologia 36(1): 129–158 doi:10.1007/BF00751287.

Lehr PA (1988) Family Asilidae. In: Soós A, Papp L (Eds) Catalogue of Palaearctic Diptera. Athericidae - Asilidae. Volume 5. Akademiai Kiado, Budapest & Elsevier, Amsterdam, 197–326.

Leppä M, Hämäläinen H (2002) Zavreliella marmorata (v.d.Wulp, 1859) (Diptera: Chironomidae) - a chironomid species new to Finland from Lake Pohjalampi, North Karelia. Entomologica fennica 12(1): 44–45.

Liljaniemi P, Vuori KM, Ilyashuk B, Luotonen H (2002) Habitat characteristics and macroinvertebrate assemblages in boreal forest streams: relations to catchment silvicultural activities. Hydrobiologia 474(1-3): 239–251 doi: 10.1023/A:1016552308537. `http://link.springer.com/article/10.1023\%2FA\%3A1016552308537?LI=true`.

Lindeberg B (1964) The swarm of males as a unit for taxonomic recognition in the chironomids (Diptera). Annales Zoologici Fennici 1: 71–76.

Lindeberg B (1967) Sibling species delimitation in the Tanytarsus lestagei aggregate (Diptera, Chironomidae). Annales Zoologici Fennici 4: 45–86.

Lindeberg B (1970) Tanytarsini (Diptera, Chironomidae) from northern Fennoscandia. Annales Zoologici Fennici 7(3): 303–312.

Lindeberg B (1974) Parthenogenetic and normal populations of Abiskomyia virgo Edw. (Diptera, Chironomidae). Entomologisk Tidskrift Supplement 95: 157–161.

Lindeberg B (1976) Taxonomy of some species of the Tanytarsus eminulus group (Dipt., Chironomidae). Annales Entomologici Fennici 42: 47–53.

Lonsdale O, Marshall SA (2007) Redefinition of the genera Clusiodes and Hendelia (Diptera: Clusiidae: Clusiodinae), with a review of Clusiodes. Studia dipterologica 14(1): 117–159.

Lucas JAW, Nielsen TR, Stå hls G (1995) The identity of the Cheilosia complex vicina (Zetterstedt, 1849) - nigripes (Meigen, 1822) (Diptera, Syrphidae). International Journal of Dipterological Research 6: 3–7.

Lundbeck W (1927) [Diptera danica : genera and species of flies hitherto found in Denmark. Part 7.] Platypezidae, Tachinidae. Diptera Danica G.E.C. Gad, Copenhagen, 571 pp. doi:http://dx.doi.org/10.5962/bhl.title.8545. `http://www.biodiversitylibrary.org/item/35321`.

Lundström C (1910) Beiträge zur Kenntnis der Dipteren Finlands VI. Chironomidae. Acta Societatis pro Fauna et Flora Fennica 33(10): 1–47.

Lundström C (1912) Beiträge zur Kenntnis der Dipteren Finlands. VIII. Supplement 2. Mycetophilidæ, Tipulidæ, Cyclindrotomidæund Limnobiidæ. Acta Societatis pro Fauna et Flora Fennica 36(1): 1–70.

Lyneborg L (1965a) A Revised List of Danish Bombyliidae (Diptera), with a Subspecific Division of Villa circumdata Meig. Entomologiske Meddelelser 34: 155–166.

Lyneborg L (1965b) The Fucellia maritima-group in North Europe, with description of a new species (Dipt., Anthomyiidae). Entomologiske Meddelelser 34: 19–29.

Lyneborg L (1968) Notes on British Therevidae (Diptera). Proceedings of the Royal Entomological Society of London (B) 37(11-12): 170–174 doi:10.1111/j.1365-3113.1968.tb00192.x.

Máca J (1980) European species of the subgenus Amiota s. str. (Diptera, Drosophilidae). Acta entomologica bohemoslovaca 77(5): 328–346. `http://www.dgrc.kit.ac.jp/~jdd/class/030704/03070445.pdf`.

MacGowan I, Horsfield D (2002) Odinia czernyi Collin (Diptera, Odiniidae) new to Britain. Dipterists Digest 9: 107–110.

MacGowan I, Rotheray G (2008) British Lonchaeidae: Diptera Cyclorrhapha, Acalyptratae. Handbooks for the Identification of British Insects Royal Entomological Society, London, 142 pp. ISBN 978-0-90154-688-3.

MacGowan I, Rotheray GE (2000) New species, additions and possible deletions to the British Lonchaea Fallen (Diptera, Lonchaeidae). Dipterists Digest 7: 37–49.

Mannheims B (1963a) Eine Tipula der ostasiatischen ”sempiterna”[sic.]-Gruppe in Finnland (Dipt., Tipulidae). Notulae Entomologicae 43: 69–74.

Mannheims B (1963b) Zwei für Fennoskandien neue Tipula-Arten (Dipt. Tipulinae). Notulae Entomologicae 43(1): 37–41.

Mannheims B (1964) Tipuliden und Limoniiden aus Südwest-Häme (Finnland). Lounais-Hämeen Luonto 15: 20–22. `http://www.lounaisluonto.net/julkaisut/LHL/LHL15.pdf`.

Mannheims B (1965) Sechzehn für Finnland neue Limoniiden mit Liste der Tipulidae, Limoniidae, Cylindrotomidae, Liriopeidae, Trichoceridae und Phryneidae (Diptera, Nematocera) Südwest-Hämes. Lounais-Hämeen Luonto 20: 4–9.

Mannheims B (1967) Tipula (Vestiplex) bo sp. n. und andere Tipuliden aus Fennoskandien (Diptera). Notulae entomologicae 47(4): 147–156.

Markkula M, Tittanen K, Hämäläinen N, Forsberg A (1979) The aphid midge Aphidoletes aphidimyza (Diptera, Cecidomyiidae) and its use in biological control of aphids. Annales Entomologici Fennici 45(4): 89–98.

Marshall SA (1985) A revision of the New World species of Minilimosina Roháček (Diptera: Sphaeroceridae). Proceedings of the Entomological Society of Ontario 116: 1–60.

Marshall SA, Roháček J (1984) A revision of the genus Telomerina Roháček (Diptera, Sphaeroceridae). Systematic Entomology 9(2): 127–163 doi:10.1111/j.1365-3113.1984.tb00511.x. `http://doi.wiley.com/10.1111/j.1365-3113.1984.tb00511.x`.

Marshall SA, Roháček J, Dong H, Buck M (2011) The state of Sphaeroceridae (Diptera: Acalyptratae): a world catalog update covering the years 20002010, with new generic synonymy, new combinations, and new distributions. Acta entomologica Musei Nationalis Pragae 51(1): 217–298. `http://www.aemnp.eu/PDF/51\_1/SphaeroceridaeLQ.pdf`.

Mathis WN, Zatwarnicki T, Kubátová-Hiršová H (2009) A revision of the shore-fly genus Philotelma Becker (Diptera: Ephydridae). Insect Systematics & Evolution 40(2): 121–158 doi:http://dx.doi.org/10.1163/187631209X416714. `http://www.ingentaconnect.com/content/brill/ise/2009/00000040/00000002/art00001`.

Mattila K (2009) Hyönteistiedonannot 2009. Diamina 2009: 32–33.

McAlpine JF (1967) The identity of Leucopis annulipes (Diptera: Chamaemyiidae). The Canadian Entomologist 99(6): 599–604.

McAlpine JF (1971) A revision of the subgenus Neoleucopis (Diptera: Chaemaemyiidae). The Canadian Entomologist 103: 1851–1874.

Mei M, Stuke JH (2008) Remarks on Zodion nigritarsis (Strobl, 1902) and other European species of Zodion Latreille, 1796, with a revised key (Diptera, Conopidae). Tijdschrift voor Entomologie 151: 3–10. `http://www.nev.nl/tve/pdf/te0151003.pdf`.

Meinander M (1977) Brief reports. Mycetophila ruficollis Meigen gruppen i Finland. Notulae entomologicae 57(2): 63.

Menzel F, Heller K (2007) Bemerkungen zur Nomenklatur der Sciariden (Diptera, Bibionomorpha: Sciaridae). Studia dipterologica 13[2006](2): 209–229.

Menzel F, Mohrig W (1999) Revision der paläarktischen Trauermücken (Diptera, Sciaridae). Studia dipterologica Supplement 6: 1–761.

Meriläinen JJ (1987) The profundal zoobenthos used as an indigator of the biological condition of Lake Päijänne. In: Lake Päijänne Symposium. Biological Research Reports from the University of Jyväskylä, vol. 10 University of Jyväskyä, Jyväskylä, 87–94 ISBN 9789516797710.

Merz B (1994) Diptera Tephritidae. Fauna Insecta Helvetica Schweizerischen Entomologischen Gesellschaft, Genéve, 198 pp. doi:10.1002/mmnd.19950420119.

Michelsen V (1980a) A revision of the beet leaf-miner complex, Pegomya hyoscyami s.lat. (Diptera: Anthomyiidae). Entomologica scandinavica 11: 297–309.

Michelsen V (1980b) The Anthomyia pluvialis complex in Europe (Diptera, Anthomyiidae). Systematic Entomology 5: 281–290.

Michelsen V (1988) A world revision of Strobilomyia gen. n.: the anthomyiid seed pests of conifers (Diptera: Anthomyiidae). Systematic Entomology 13(3): 271–314 doi:10.1111/j.1365-3113.1988.tb00244.x. `http://onlinelibrary.wiley.com/doi/10.1111/j.1365-3113.1988.tb00244.x/abstract`.

Michelsen V (2006a) A new European species of Pegomya Robineau-Desvoidy (Diptera: Anthomyiidae) near P. flavoscutellata (Zetterstedt). Zootaxa 1257: 49–55.

Michelsen V (2006b) Annotated catalogue of the Anthomyiidae, Fanniidae, Muscidae and Scathophagidae (Diptera: Muscoidea) of Greenland. Steenstrupia 29(2): 105–126.

Michelsen V (2007a) A new European species of Delia Robineau-Desvoidy (Diptera: Anthomyiidae) near the wheat bulb fly, D. coarctata (Fallén). Zootaxa 1412: 61–67.

Michelsen V (2007b) Taxonomic review of Eurasian Paradelia Ringdahl (Diptera: Anthomyiidae). Zootaxa 1592: 1–44.

Michelsen V (2007c) Two new European species of Delia Robineau-Desvoidy (Diptera: Anthomyiidae) with a bipartite male sternite III. Zootaxa 1469: 51–57.

Michelsen V (2008a) Fauna Europaea: Anthomyiidae, version 1.3. `http://www.faunaeur.org/`.

Michelsen V (2008b) The Palaearctic species of the Pegomya terminalis species group (Diptera: Anthomyiidae), with descriptions of two new species. Zootaxa 1781: 31–46.

Michelsen V (2009a) Revision of the Botanophila helviana species group (Diptera: Anthomyiidae) in Europe. Zootaxa 2108: 45–61.

Michelsen V (2009b) Revision of the willow catkin flies, genus Egle Robineau-Desvoidy (Diptera: Anthomyiidae), in Europe and neighbouring areas. Zootaxa 2043: 1–76. `http://www.mapress.com/zootaxa/2009/f/z02043p076f.pdf`.

Michelsen V (2012) Revision of the European Delia pruinosa species group (Diptera: Anthomyiidae) feeding as larvae in seed capsules of Silene L.(Caryophyllaceae). Zootaxa 3434: 31–48. http://mapress.com/zootaxa/2012/f/z03434p048f.pdf.

Michelsen V, Ackland DM (2009) The Pegomya maculata species group (Diptera Anthomyiidae) in Europe, with description of a new species. Zootaxa 2315: 51–65. http://216.92.149.63/zootaxa/2009/f/z02315p065f.pdf.

Mohrig W (1993) Der Artenkreis Corynoptera concinna (Winnertz, 1867) (Diptera, Sciaridae). Bonner Zoologische Beiträge 44(1-2): 47–55. http://www.zfmk.de/BZB/B44_H1_2/BZBB44H5.PDF.

Mohrig W, Heller K, Hippa H, Vilkamaa P, Menzel F (2013) Revision of the Black Fungus Gnats (Diptera: Sciaridae) of North America. Studia dipterologica 19[2012](1): 141–286.

Mohrig W, Menzel F (1993) Revision der paläarktischen Arten der Bradysia brunnipes-Gruppe (Diptera, Sciaridae). Bonner Zoologische Beiträge 44(3.4): 267–291.

Mohrig W, Menzel F (1997) Revision der paläarktischen Arten von Trichosia Winnertz sensu Tuomikoski, 1960 (Diptera, Sciaridae). - Teil II. Gattungen Leptosciarella Tuomikoski, 1960 und Trichodapus gen. nov. Studia dipterologica 4(1): 41–98.

Morge G (1967) Die Lonchaeidae und Pallopteridae Österreichs und der angrenzenden Gebiete 2. Teil: Die Pallopteridae. Naturkundliches Jahrbuch der Stadt Linz .

Mukkala VM, Haarto A, Koponen S, Mukkala L, Rinne V, Salmela J (2005) Ilmajoen Kivistönmäen hyönteisistä, hämähäkkieläimistä ja muista selkärangattomista. w-album 2: 3–34.

Munari L, Mathis WN (2010) World Catalog of the Family Canacidae (including Tethinidae), with keys to the supraspecific taxa. Zootaxa 2471: 1–84.

Murray DA (1976) Thienemannimyia pseudocarnea n.sp., a palaearctic species of the Tanypodinae (Diptera: Chironomidae). Entomologica scandinavica 7(3): 191–194 doi:10.1163/187631276X00315.

Nartshuk EP (1992) Revision of the Species of Meromyza Meigen (Chloropidae) from Finland. Entomologica fennica 3(3): 121–138.

Nartshuk EP (1995) Taxonomic and faunistic data on the Rhagionidae (Diptera, Brachycera) of the northern Palaearctic. Acta Zoologica Fennica 199: 17–24.

Nartshuk EP (1998) A revision of grassflies of the tribe Chloropini (Diptera, Chloropidae) of Finland, Estonia and North-West Russia. Entomologica fennica 9(3): 153–183.

Nartshuk EP (1999) Grassflies on the subfamily Chloropinae, except the tribe Chloropini and the genus Meromyza, of Finland, Karelia and the Kola Peninsula (Diptera, Cyclorrhapha, Chloropidae). Entomologica fennica 10(1): 7–28.

Nartshuk EP (2002) On taxonomy of Palaearctic Chlorops Meigen (Diptera, Cyclorrhapha, Chloropidae). International Journal of Dipterological Research 13(1): 15–21.

Nartshuk EP, Andersson H (2013) The Frit Flies (Chloropidae, Diptera) of Fennoscandia and Denmark. Fauna Entomologica Scandinavica Brill, Leiden, 282 pp.

Negrobov OP, Thuneberg E (1970) Some questions on the systematics of the genus Medetera (Dip., Dolichopodidae) of the Palaearctic region. Annales Entomologici Fennici 36(3): 143–145.

Nielsen TR (1972) Platycheirus monticolus nov. sp., a northern species confused with Platycheirus discimanus Loew (Diptera, Syrphidae). Norsk Entomologisk Tidsskrift 19: 91–98.

Nielsen TR (1995) Studies on some northern Eristalis species (Diptera, Syrphidae). International Journal of Dipterological Research 6(2): 129–133.

Nielsen TR (2004) European species of the Platycheirus ambiguus group (Diptera, Syrphidae), with description of new species. Volucella 7: 1–30.

Nielsen TR, Claussen C (2001) On Cheilosia ingerae spec. nov. (Diptera, Syrphidae) from Northern Fennoscandia. Dipteron 4(1): 43–56.

Nordman AF (1940) Notis om de i veden i strandalarna vid Tvärminne Zoologiska Station levande insekterna. Notulae Entomologicae 20(1): 17–18.

Nordman AF (1963) Die Bohrfliege Phagocarpus permundus (Harris) (Dipt., Trypetidae) in Finnland gefunden. Notulae Entomologicae 43(1): 43–44.

Norrbom AL, Kim KC (1985) Systematics of Crumomyia Macquart and Alloborborus Duda (Diptera: Sphaeroceridae). Systematic Entomology 10(2): 167–225. `http://onlinelibrary.wiley.com/doi/10.1111/j.1365-3113.1985.tb00527.x/abstract`.

Nowakowski JT (1967) Vorläufige Mitteilung zu einer Monographie der europäischen Arten der Gattung Cerodontha Rond. (Diptera, Agromyzidae). Polskie Pismo Entomologiczne 37(4): 633–661.

Nowakowski JT (1973) Monographie der europäischen Arten der Gattung Cerodontha Rond. Annales Zoologici (Warszawa) 31(1): 1–327.

Nuorteva M (1959) Untersuchungen über einige in den Frassbildern der Borkenkäfer lebende Medetera-Arten (Dipt., Dolichopodidae). Annales Entomologici Fennici 25(4): 192–210.

Nuorteva M (1967) On the habitats of some Lonchaea species in Fennoscandia (Dipt., Lonchaeidae). Annales Entomologici Fennici 33(2): 118–121.

Nuorteva P (1960) Liero- ja raatokärpästen esiintymisen jatkuva tarkkailu aloitettu Urjalassa. Lounais-Hämeen Luonto 11: 75–82.

Nuorteva P (1963) Synanthropy of Blowflies (Dipt., Calliphoridae) in Finland. Annales Entomologici Fennici 29(1): 1–49.

Nuorteva P (1964) The zonal distribution of blowflies (Dipt., Calliphoridae) on the arctic hill Ailigas in Finland. Annales Entomologici Fennici 30(4): 218–226.

Nuorteva P, Järvinen U (1961) The insect fauna of the nests of the Sand Martin (Riparia riparia L.) in Finland. Annales Entomologici Fennici 27(4): 197–204.

Ø kland Br, Mamaev BM (1997) Fennoscandian records of Lestremiinae (Diptera: Cecidomyiidae). Fauna Norvegica Series B 44(2): 123–128. http://www.entomologi.no/journals/nje/old/V44/NJE_44_02_1997.pdf.

Olafsson E (1991) Taxonomic revision of western Palaearctic species of the genera Scatella R.-D. and Lamproscatella Hendel, and studies on their phylogenetic positions within the subfamily Ephydrinae. Entomologica scandinavica Supplement 36: 1–100.

Oosterbroek P (2011) Catalogue of the Craneflies of the World (Diptera, Tipuloidea: Pediciidae, Limoniidae, Cylindrotomidae, Tipulidae). http://ip30.eti.uva.nl/ccw/index.php.

Oosterbroek P, Theowald B (1992) Family Tipulidae. In: Soós A, Papp L, Oosterbroek P (Eds) Catalogue of Palaearctic Diptera. Trichoceridae - Nymphomyiidae. Volume 1. Hungarian Natural History Museum, Budapest, 56–178 ISBN 9637093109.

Paasivirta L (1979) The Chironomidae of three Finnish forest lakes with different content of humus and nutrients In: Viitala J (Ed) NCE-symposium Ecology and fishery biology of small forest lakes Lammi 15.17. XI 1978. Jyväskylän yliopiston biologian laitoksen tiedonantoja, vol. 19 41 University of Jyväskylä, Jyväskylä.

Paasivirta L (1982) Saarijärven Pyhä-Häkin kansallispuiston ja sen lähiympäristön metsäjärvien koskikorento-, sudenkorento-, kaislakorento-, verkkosiipis- ja sääskilajisto. In: Viitala J (Ed) Saarijärven Pyhä-Häkin kansallispuiston ja sen lähiympäristön metsäjärvien veden laatu, klorofyllipitoisuus, eläinplankton, pohjaeläimistö sekä vesihyönteis- ja vesipunkkilajisto. Jyväskylän yliopiston biologian laitoksen tiedonantoja, vol. 29 Jyväskylän yliopisto, 40–49 ISBN 9516787231.

Paasivirta L (1983) Konneveden vesihyönteisistä. Jyväskylän yliopiston biologian laitoksen tiedonantoja 34: 25–36.

Paasivirta L (2002) Surviaissääsket, sulkasääsket ja hyttyset. In: Salokannel J (Ed) Iidesjärven hyönteisselvitys. Diamina, vol. 11, 25–26.

Paasivirta L (2009) Chironomidae (Diptera: Nematocera) in the biogeographical provinces of Finland. `http://www.ymparisto.fi/download.asp?contentid=82649`.

Paasivirta L (2012) Finnish Chironomidae. Chironomus 25: 47–70. `http://www.ntnu.no/ojs/index.php/chironomus/article/view/1546/1439`.

Paasivirta L, Koskenniemi E (1984) No Title. In: yliopisto J (Ed) Järvien ja jokien pohjaeläintutkijoiden kokous 13. - 15.10.1983. Jyväskylän yliopiston biologian laitoksen tiedonantoja, vol. 38 Jyväskylän yliopisto, Jyväskylä, –48– ISBN 951-679-151-4.

Palmén E (1955) Diel periodicity of pupal emergence in natural populations of some chironomids (Diptera). Annales Societatis Zoologicae-Botanicae Fenniae Vanamo 17(3): 1–30.

Panelius S (1965) A revision of the European gall midges of the subfamily Porricondylinae (Diptera: Itonididae). Acta Zoologica Fennica 113: 1–157.

Pape T (1987a) An annotated check-list of Finnish flesh-flies (Diptera: Sarcophagidae). Notulae Entomologicae 67: 43–46.

Pape T (1987b) The Sarcophagidae (Diptera) of Fennoscandia and Denmark. Fauna Ent. Scand, vol. 19 E. J. Brill/Scandinavian Science Press Ltd, Leiden-Kobenhagen, 203 pp.

Pape T (1993) The Sarcophagidae described by C. De Geer, J. H. S. Siebke, and O. Ringdahl. Entomologica fennica 4: 143–150.

Pape T (1994) The world Blaesoxipha Loew, 1861 (Diptera: Sarcophagidae). Entomologica scandinavica Supplement 45: 1–247.

Papp L (1984) Family Lauxaniidae. In: Catalogue of Palaearctic Diptera. vol. 9 Akademiai Kiado, Budapest & Elsevier, Amsterdam, 193–217.

Penttinen J, Jaschhof M (2009) On the systematics of Sylvenomyia Mamaev & Zaitzev (Diptera, Cecidomyiidae, Porricondylinae), with the description of a new species from Finland. Zootaxa 2032: 48–54. `http://www.mapress.com/zootaxa/list/2009/zt02032.html`.

Penttinen J, Siitonen J (2005) Five species of log midges (Diptera, Cecidomyiidae: Porricondylinae) new to Finland. Sahlbergia 10: 50–53.

Penttinen J, Spungis V (2007) Additions to the Finnish fauna of log midges (Diptera, Cecidomyiidae: Porricondylinae). Sahlbergia 12: 36–42.

Piirainen T (2001) Maalle uusi kukkakärpäslaji Epistrophe annulitarsis (Stackelberg). Diamina 10: 1–2.

Pilipenko VE, Salmela J, Vesterinen EJ (2012) Description and DNA barcoding of Tipula (Pterelachisus) recondita sp. n. from the Palaearctic region (Diptera, Tipulidae). ZooKeys 192: 51–65. http://www.ncbi.nlm.nih.gov/pmc/articles/PMC3349062/.

Pohjoismäki J (2006) No Title. Diamina 15: 1–.

Polevoi A (2001a) List of Diptera, collected within Koitajoki area during the study period (1993-1996). In: Hokkanen TJ (Ed) Diversity Studies in Koitajoki Area (North Karelian Biosphere Reserve, Ilomantsi, Finland). Metsähallituksen luonnonsuojelujulkaisuja. Sarja A, vol. 131 Metsähallitus, Vantaa, 191–202 ISBN 952-446-325-3. http://julkaisut.metsa.fi/julkaisut/show/34.

Polevoi A, Hedmark K (2004) New species of the genus Boletina Winnertz (Diptera: Mycetophilidae) from Fennoscandia. Entomologica fennica 15(1): 23–33.

Polevoi A, Jakovlev J (2004) Changes in the Finnish fauna of fungus gnats (Diptera: Mycetophilidae s. l.) since Hackmans checklist. Sahlbergia 9(2): 135–140.

Polevoi A, Jakovlev J (2011) A review of the European species of the genus Tetragoneura Winnertz (Diptera: Mycetophilidae). Zootaxa 3062: 1–12. http://mapress.com/zootaxa/2011/f/z03062p012f.pdf.

Polevoi A, Jakovlev J, Zaitzev A (2006) Fungus gnats (Diptera Bolitophilidae, Diadocidiidae, Keroplatidae and Mycetophilidae) new to Finland. Entomologica fennica 17(2): 161–169.

Polevoi AV (1995a) Fungus gnats (Diptera, Mycetophilidae) in Pirhu and Tapionaho (Ilomantsi, Finland). In: Hokkanen TJ, Leshko E (Eds) Karelian Biosphere Reserve Studies. North Karelian biosphere reserve, Ilomantsi, 159–166.

Polevoi AV (1995b) New and poorly known fungus gnats of the families Bolitopholidae, Diadocidiidae and Keroplatidae from Eastern Fennoscandia (Diptera, Nematocera). Zoosystematica Rossica 4(1): 177–182.

Polevoi AV (2001b) New and little known species of the fungus gnat subfamilies Mycomyinae and Sciophilinae Diptera, Mycetophilidae from Eastern Fennoscandia. Entomologicheskoe Obozrenie 80(2): 518–526.

Polevoi AV (2003) Review of the Fennoscandian species of the genus Syntemna Winnertz (Diptera, Mycetophilidae) including the description of a new species. Studia dipterologica 10(1): 133–142.

Polevoi AV, Stå hls G (1994) New records of threatened Diptera in Finland. Sahlbergia 1: –24–.

Pont AC (1986) Family Muscidae. In: Soós A, Papp L (Eds) Catalogue of Palaearctic Diptera. Scathophagidae-Hypodermatidae. Volume 11. Catalogue of Palaearctic Diptera, vol. 11 Akademiai Kiado, Budapest & Elsevier, Amsterdam, 57–215.

Pont AC (1989) Azelia monodactyla Loew, 1874, and Azelia trigonica Hennig, 1956 (Muscidae), new to Finland. Notulae entomologicae 69: 215.

Pont AC (1990) Notes on Finnish Sepsidae and Muscidae (Diptera). Entomologica fennica 1(3): 154.

Pont AC (2002) The Fanniidae (Diptera) described by J. W. Zetterstedt. Insect Systematics & Evolution 33(1): 103–112 doi:http://dx.doi.org/10.1163/187631202X00073.

Pont AC, Meier R (2002) The Sepsidae (Diptera) of Europe. Fauna Entomologica Scandinavica, vol. 37 Brill NV, Leiden, New York, Köln, 221 pp. ISBN 9004124772.

Raunio J, Langton PH (2005) First record of Georthocladius luteicornis (Diptera: Chironomidae) in Finland. Sahlbergia 10: 7–9.

Raunio J, Paasivirta L, Brodin Y (2009) Marine midge Telmatogeton japonicus Tokunaga (Diptera: Chironomidae) exploiting brackish water in Finland. Aquatic Invasions 4(2): 405–408. `http://www.aquaticinvasions.net/2009/AI\_2009\_4\_2\_Raunio\_etal.pdf`.

Rautio O (1985) Suomelle uusia lajeja yläheimosta Tipuloidea. Notulae entomologicae 65: 166.

Rautio O (1986) Brief reports. Suomelle uusi vaaksiainen (Tipulidae). Notulae entomologicae 66(4): 189–190.

Rautio O (1987) Suomelle uusi vaaksiainen (Tipulidae). Notulae entomologicae 67: 216.

Reiss F, Fittkau EJ (1971) Taxonomie und Ökologie europäisch verbreiteter Tanytarsus-Arten (Chironomidae, Diptera). Archiv für Hydrobiologie, Suppl. 40: 75–200.

Remm H (1988) Family Ceratopogonidae. In: Sóos A, Papp L (Eds) Catalogue of Palaearctic Diptera. Ceratopogonidae - Mycetophilidae. Volume 3. Akademiai Kiado, Budapest & Elsevier, Amsterdam, 11–110.

Ringdahl O (1932a) Eine neue Brachycoma-Art. Notulae entomologicae 12(1): 21.

Ringdahl O (1932b) Vier neue Anthomyiden. Notulae entomologicae 12(1): 19–20.

Ringdahl O (1935) Neue fennoskandische Musciden. Notulae entomologicae 15(1-2): 26–31.

Rognes K (1991) Blowflies (Diptera, Calliphoridae) of Fennoscandia and Denmark. Fauna Entomologica Scandinavica, vol. 24 Scandinavian Science Press Ltd, Leiden- New York - København - Köln, 272 pp.

Roháček J (1977) Limosina hackmani sp.n. and its relatives (Diptera, Sphaeroceridae). Notulae entomologicae 57(4): 115–123.

Roháček J (1981) A contribution to the taxonomy of some European species of Limosina Macq. (Diptera: Sphaeroceridae). Folia entomologica Hungarica 42(1): 163–168.

Roháček J (1983) A monograph and re-classification of the previous genus Limosina Macquart (Diptera, Sphaeroceridae) of Europe. Part II. Beiträge zur Entomologie, Berlin 33(1): 3–195.

Roháček J (1991) A monograph of Leptocera (Rachispoda Lioy) of the West Palearctic area. Časopis Slezského zemského muzea Opava (A) 40: 97–288.

Roháček J (1993) Herniosina Roháček and Minilimosina Roháček of Europe: two new species, new records and taxonomic notes (Insecta, Diptera: Sphaeroceridae). Entomologische Abhandlungen Staatliches Museum für Tierkunde Dresden 55(12): 185–203.

Roháček J (1996) Revision of palaearctic Stiphrosoma, including the Anthomyza laeta-group (Diptera: Anthomyzidae). European Journal of Entomology 93(1): 89–120. http://www.eje.cz/pdfarticles/624/eje_093_1_089_Rohacek.pdf.

Roháček J (2000) The type material of Sphaeroceridae described by J. Villeneuve with lectotype designations and nomenclatural and taxonomic notes (Diptera). Bulletin de la Societe Entomologique de France 105(5): 467–478.

Roháček J (2006) A monograph of Palaearctic Anthomyzidae (Diptera) Part 1. Časopis Slezského zemského muzea Opava (A) Supplement 55(1): 1–328.

Roháček J (2013) New host-plant, habitat and distributional records of West Palaearctic Anthomyzidae (Diptera). Časopis Slezského zemského muzea Opava (A) 62(1): 23–43 doi:10.2478/cszma-2013-0002. http://www.degruyter.com/view/j/cszma.2013.62.issue-1/cszma-2013-0002/cszma-2013-0002.xml.

Roháček J, Marshall SA (1985) The genus Trachyopella Duda (Diptera, Sphaeroceridae) of the Holarctic Region. Museo Regionale di Scienze Naturali, Monografie Museo regionale di scienze naturali, Torino, 109 pp.

Rotheray GE, McGowan I (1999) Palloptera anderssoni sp. n. from Scotland and Finland (Diptera: Pallopteridae). British Journal of Entomology and Natural History 11[1998]: 175–179. http://www.diptera.info/downloads/Rotheray-MacGowan-1999.pdf.

Rozkošný R (1973) The Stratiomyioidea (Diptera) of Fennoscandia and Denmark. Fauna Entomologica Scandinavica, vol. 1 Scandinavian Science Press Ltd, Gadstrup, 140 pp.

Rozkošný R (1981) Distribution of the Finnish Sciomyzidae (Diptera), with additions and corrections to the list of Finnish species. Notulae entomologicae 61(4): 209–215.

Rozkošný R (1982) Three new species of Pherbellia Robineau-Desvoidy and new synonyms of Holarctic and Palaearctic Sciomyzidae (Diptera). Annales Entomologici Fennici 48(2): 51–56.

Rozkošný R (1984) The Sciomyzidae (Diptera) of Fennoscandia and Denmark. Fauna Entomologica Scandinavica, vol. 14 Brill, Leiden, 224 pp. ISBN 9789004075924.

Rozkošný R, Gregor F, Pont AC (1997) The European Fanniidae (Diptera). Acta scientiarum naturalium Academiae scientiarum bohemicae, Brno (New Series) 31: 1–80.

Saether OA (1975) Nearctic and Palearctic. Heterotrissocladius (Diptera: Chironomidae). Bulletin of the Fisheries Research Board of Canada 193: 1–67.

Saether OA (1985) Limnophyes er sp. n. (Diptera: Chironomidae) from Finland, with new Nearctic records of previously described species. Entomologica scandinavica 15(4): 540–544 doi:http://dx.doi.org/10.1163/187631284X00398.

Salmela J (2001a) Adult craneflies (Diptera, Nematocera) around springs in southern Finland. Entomologica fennica 12(3): 139–152.

Salmela J (2001b) Kolme maalle uutta kaksisiipistä (Diptera, Nematocera). Diamina 10: 21.

Salmela J (2002) Iidesjärven sääsket. Diamina 11: 30–32.

Salmela J (2003a) New records of the families Psychodidae, Dixidae and Thaumaleidae (Diptera, Nematocera) from Finland. Studia dipterologica 10(1): 143–152.

Salmela J (2003b) Norosääsket (Diptera, Thaumaleidae), uusi suomalainen sääskiheimo. Diamina 12: 11.

Salmela J (2004) Semiaquatic flies (Diptera, Nematocera) of three mires in the southern boreal zone, Finland. Memoranda Societatis pro Fauna et Flora Fennica 80: 1–10.

Salmela J (2005) New moth flies for eastern Fennoscandia (Diptera, Psychodidae). Sahlbergia 10: 1–3.

Salmela J (2006) Ecology, distribution and redlist status of craneflies, phantom midges, mothflies, meniscus midges and trickle midges in Finland (Diptera, Nematocera) Tech. rep.

Salmela J (2008) Semiaquatic fly (Diptera, Nematocera) fauna of fens, springs, headwater streams and alpine wetlands in the northern boreal ecoregion, Finland. w-album 6: 3–63. `http://org.utu.fi/harrastus/TEKS/w-album/608.pdf`.

Salmela J (2009) The subgenus Tipula (Pterelachisus) in Finland (Diptera, Tipulidae) - species and biogeographic analysis. Zoosymposia 3: 245–261. `http://staff.science.uva.nl/~oosterbr/Salmela,2009b.pdf`.

Salmela J (2010) Tahmelan lähteikön aikuiset semiakvaattiset sääsket ja muut vesihyönteiset lajisto ja suojeluarvo. Diamina 19: 6–14. `http://staff.science.uva.nl/~oosterbr/Salmela,2010.pdf`.

Salmela J (2011) Annotated list of Finnish crane flies (Diptera: Tipulidae, Limoniidae, Pediciidae, Cylindrotomidae). Entomologica fennica 22: 219–242. `http://ojs.tsv.fi/index.php/entomolfennica/article/view/5002/4535`.

Salmela J, Autio O (2007a) Redescription of Tipula octomaculata Savchenko, with notes on related holarctic species (Diptera, Tipulidae). Zootaxa 1527: 53–58.

Salmela J, Autio O (2007b) Semiaquatic flies of Kivineva mire, Middle boreal Finland, and redescription of Cylindrotoma borealis Peus, 1952 stat. n. (Diptera, Nematocera). International Journal of Dipterological Research 18: 47–55.

Salmela J, Autio O (2009) Tipula (Yamatotopula) moesta Riedel and related species in Finland (Diptera, Tipulidae). Entomologica fennica 20(1): 49–55.

Salmela J, Autio O, Ilmonen J (2007a) A survey on the nematoceran (Diptera) communities of southern Finnish wetlands. Memoranda Societatis pro Fauna et Flora Fennica 83: 33–47. `http://ojs.tsv.fi/index.php/msff/article/view/411`.

Salmela J, Autio O, Kulmala K (2007b) Tipula laetibasis Alexander, 1934 löydetty Suomesta (Diptera, Tipulidae). Sahlbergia 12: 33–35.

Salmela J, Halme P (2007) Keskisuomalaisen pihapiirin sääskilajistoa. Diamina 16: 4–7.

Salmela J, Härmä O (2004) Adelphomyia punctum (Diptera, Limoniidae) found from Finland. Sahlbergia 9: 141–143.

Salmela J, Jakovlev J, Polevoi A, Penttinen J, Vartija NA (2014) Recent noteworthy findings of fungus gnats from Finland and northwestern Russia (Diptera: Ditomyiidae, Keroplatidae, Bolitophilidae and Mycetophilidae). Biodiversity Data Journal 2: e1068 doi:10.3897/BDJ.2.e1068. `http://bdj.pensoft.net/articles.php?id=1068`.

Salmela J, Piirainen T (2003) Contribution to the taxonomy of the Holarctic Ula Haliday, 1833 (Diptera, Pediciidae). Norwegian Journal of Entomology 50(2): 73–90. http://www.entomologi.no/journals/nje/2003-2/NJE_50_02_2003.pdf.

Salmela J, Starý J (2009) Description of Metalimnobia (Metalimnobia) charlesi sp. n. from Europe (Diptera, Limoniidae). Entomologica fennica 19(19): 268–272. http://staff.science.uva.nl/~oosterbr/SalmelaandStary,2009.pdf.

Salmela J, Vilkamaa P (2005) Sciaridae fauna of Central Finland (Diptera, Nematocera): faunistics and taxonomy. Entomologica fennica 16(4): 287–300.

Savage J, Wheeler TA (1999) Systematics of Cetema Hendel: revision of the Nearctic species and phylogeny and zoogeography of the Holarctic fauna. Entomologica scandinavica 30(3): 249–262 doi:10.1163/187631200X00101.

Savchenko EN, Oosterbroek P, Starý J (1992) Family Limoniidae. In: Soós A, Papp L, Oosterbroek P (Eds) Catalogue of Palaearctic Diptera. Trichoceridae - Nymphomyiidae. Volume 1. vol. 1 Hungarian Natural History Museum, Budapest, 183–374.

Säwedal L (1976) Revision of the notescens-group of the genus Micropsectra Kieffer, 1909 (Diptera: Chironomidae). Entomologica scandinavica 7(2): 109–144 doi:10.1163/187631276X00207.

Säwedal L, Langton PH (1977) Redescription of Paratanytarsus tenellulus (Goetghebuer, 1921) (Diptera: Chironomidae). Entomologica scandinavica 8(3): 167–171 doi:10.1163/187631277X00233.

Schmitz H (1943) 33. Phoridae. Lieferung 147 & 149. In: Die Fliegen der palaearktischen Region 4(7). Die Fliegen der palaearktischen Region, vol. 4(33) E. Schweizerbart'sche Verlagsbuchhandlung, Stuttgart, 129–192.

Schmitz H (1957) 33. Phoridae. Lieferung 196. In: Die Fliegen der palaearktischen Region 4(7). E. Schweizerbart'sche Verlagsbuchhandlung, Stuttgart, 417–464.

Schmitz H (1958) 33. Phoridae. Lieferung 202. In: Die Fliegen der paläarktischen Region 4(7). E. Schweizerbart'sche Verlagsbuchhandlung, Stuttgart, 465–512.

Schmitz H, Beyer E (1965a) 33. Phoridae. Lieferung 258. In: Die Fliegen der palaearktischen Region 4(7). Die Fliegen der palaearktischen Region E. Schweizerbart'sche Verlagsbuchhandlung, Stuttgart, 513–560.

Schmitz H, Beyer E (1965b) 33. Phoridae. Lieferung 260. In: Die Fliegen der paläarktischen Region 4(7). E. Schweizerbart'sche Verlagsbuchhandlung, Stuttgart, 561–608.

Schmitz H, Beyer E (1974) 33. Phoridae. Lieferung 301. In: Die Fliegen der palaearktischen Region 4(7). Die Fliegen der palaearktischen Region E. Schweizerbart'sche Verlagsbuchhandlung, Stuttgart, 609–664.

Siitonen O (1982) Tipula (Savtschenkia) benesignata Mannheims, Suomelle uusi vaaksiaislaji (Tipulidae). Notulae entomologicae 62(4): 159.

Siitonen O (1983a) Kaksi Suomelle uutta pikkuvaaksiaista (Limoniidae). Notulae entomologicae 63(4): 215.

Siitonen O (1983b) Kaksi Suomelle uutta vaaksiaista (Tipulidae). Notulae entomologicae 63(4): 215.

Siitonen O (1984) Diogma caudata Takahashi, 1960, Suomelle uusi vaaksiainen (Cylindrotomidae). Notulae Entomologicae 64: 203.

Silfverberg H (1981) Haematopota crassicornis Wahlb. (Tabanidae) i Finland. Notulae entomologicae 61(4): 225.

Sinclair BJ (1999) Review of the Holarctic Clinocera appendiculata complex. Entomologist's Monthly Magazine 135: 223–232.

Skartveit J (1999) Two species of Bibionidae (Diptera) new to the Finnish fauna. Entomologica fennica 10(2): 115–116.

Sø li GEE (1997) On the morphology and phylogeny of Mycetophilidae, with a revision of Coelosia Winnertz (Diptera, Sciaroidea). Entomologica scandinavica Supplement 50: 1–137.

Söderman G, Winqvist K, Albrecht A, Kahanpää J (2007) Suomen hedelmäkärpästen (Diptera: Tephritidae) biologia, levinneisyys ja uhanalaisuus. Sahlbergia 12: 1–19.

Spencer KA (1976) The Agromyzidae (Diptera) of Fennoscandia and Denmark. Fauna entomologica Scandinavica, vol. 5 Scandinavian Science Press, Klampenborg, 606 pp. ISBN 8787491087.

Stå hls G (1986) Brief reports. Allodia pxyidiiformis Zaizev (Mycetophilidae) i Finland. Notulae entomologicae 66(4): 190.

Stå hls G (1990) Brief reports. Svampmyggfynd i Finland (Mycetophilidae). Notulae entomologicae 69[1989](4): 214.

Stå hls G (1995) Additions and deletions to Syrphidae (Diptera) of Finland. Entomologica fennica 6(1): 7–9.

Stå hls G, Kahanpää J (2006) New data on Platypezidae and Opetiidae (Diptera) of Finland. Sahlbergia 11(1): 1–6.

Stå hls G, Rättel E (2013) Genus Microsania (Platypezidae: Microsaninae) in Finland. Sahlbergia 19(1-2): 50–52.

Stå hls G, Rättel E, Muona J (2012) Bolopus furcatus (Fallén, 1826) (Diptera, Platypezidae) rediscovered in Finland. Sahlbergia 18(2): 29–32.

Stå hls G, Vujić A (2009) Cryptopipiza notabila (Violovitsh, 1985) (Diptera, Syrphidae) new to Finland. Sahlbergia 15(2): 1–2.

Stå hls G, Vujić A, Milankov V (2008) Cheilosia vernalis (Diptera, Syrphidae) Complex: Molecular and Morphological Variability. Annales Zoologici Fennici 45(2): 149–159 doi:10.5735/086.045.0206. www.sekj.org/PDF/anz45-free/anz45-149.pdf.

Starý J, Salmela J (2004) Redescription and biology of Limonia badia (Walker) (Diptera: Limoniidae). Entomologica fennica 15(1): 41–47.

StoråR (1939) Mitteilungen über die Nematoceren Finnlands II. Notulae Entomologicae 19(1-2): 16–30.

StoråR (1954) Eine neue Hydrophorus-Art aus Finnland. (Dipt., Dolichopodidae). Notulae entomologicae 34: 70–74.

StoråR (1958) Über einige Helomyziden-Arten (Diptera) Finnlands. Notulae entomologicae 38(2): 61–63.

Stuke JH, Clements DK (2008) Revision of the Myopa testacea Species-Group in the Palaearctic Region (Diptera: Conopidae). Zootaxa 1713: 1–26. http://www.dce.org.uk/wp-content/uploads/2012/05/Stuke-Clements-2008-Myopa-testacea-group.pdf.

Stur E, Ekrem Tr (2006) A revision of West Palaearctic species of the Micropsectra atrofasciata species group (Diptera: Chironomidae). Zoological Journal of the Linnean Society 146(2): 165–225 doi:10.1111/j.1096-3642.2006.00198.x. http://doi.wiley.com/10.1111/j.1096-3642.2006.00198.x.

Szilády Z (1934) Die palaearktischen Rhagioniden. Annales historico-naturales Musei Nationalis Hungarici 28: 229–277. http://publication.nhmus.hu/pdf/annHNHM/Annals_HNHM_1934_Vol_28_229.pdf.

Thompson FC (1980) The problem of old names as illustrated by Brachyopa ”conica Panzer”, with a synopsis of Palaearctic Brachyopa Meigen (Diptera: Syrphidae). Entomologica scandinavica 11: 209–216. http://www.ingentaconnect.com/content/brill/ise/1980/00000011/00000002/art00008.

Thompson FC, Torp E (1982) Two new palaearctic Syrphidae (Diptera). Entomologica scandinavica 13: 441–444. http://www.ingentaconnect.com/content/brill/ise/1982/00000013/00000004/art00007.

Thuneberg E (1975) Communications at the meetings of the Entomological Society of Finland. Annales Entomologici Fennici 41(1): 37–41.

Tiensuu L (1933) Tietoja Suomessa tavatuista sukakärpäsistä (Muscidae). Notulae entomologicae 13(3-4): 78–80.

Tiensuu L (1935a) Die bisher aus Finnland bekannten Musciden. Acta Societatis pro Fauna et Flora Fennica 58(4): 1–56.

Tiensuu L (1935b) Kokousselostuksia [Suomen Phorbia-lajeja]. Annales Entomologici Fennici 1(4): 149.

Tiensuu L (1941) Brachycera (Muscidae, Tachinidae). In: Enumeratio Insectorum Fenniae. V I Diptera. Helsingin hyönteisvaihtoyhdistys r.y, 32–43.

Tiensuu L (1946a) Beiträge zur Muscidenfauna Nordeuropas (Dipt.). Annales Entomologici Fennici 12(2): 63–68.

Tiensuu L (1946b) Coniosternum tinctinervis Becker (Cordyluridae) von Finnland. Annales Entomologici Fennici 12(1): 6–9.

Tiensuu L (1951) Notes on the smallreed coccid, Eriopeltis lichtensteini Sign. (Hem., Coccoidea, Lecanoidae) and its natural enemies in Finland. Annales Entomologici Fennici 17(1): 3–10.

Tiensuu L (1963) Sata äkämää Kymenlaaksosta. Kymenlaakson Luonto 4: 21–33.

Tiensuu L (1964) Huomion arvoisia kärpäslöytöjä Lounais-Hämeestä. Lounais-Hämeen Luonto 15: 76–77.

Tjeder B (1972) Rhypholophus pseudosimilis Lundström, 1912; lectotype designation (Diptera, Tipulidae). Notulae entomologicae 52(2): 79–80.

Tschorsnig HP, Herting B (1994) Die Raupenfliegen (Diptera: Tachinidae) Mitteleuropas: Bestimmungstabellen und Angaben zur Verbreitung und Ökologie der einzelnen Arten. Stuttgarter Beiträge zur Naturkunde A 506: 1–170. `http://ia700401.us.archive.org/18/items/stuttgarter5065171994staa/stuttgarter5065171994staa.pdf`.

Tuiskunen J (1983) A description of Corynoneura gynocera sp. n. (Diptera, Chironomidae) from Finland. Annales Entomologici Fennici 49(4): 100–102.

Tuiskunen J (1984) Lindebergia bothnica. gen. n., sp. n. (Diptera, Chironomidae) from Finland. Annales Entomologici Fennici 50: 121–122.

Tuiskunen J (1985) A description of Prosmittia rectangularis sp. n. (Diptera, Chironomidae) from northern Finland. Annales Entomologici Fennici 51(2): 55–56.

Tuiskunen J (1986a) A description of Psilometriocnemus europaeus sp.n. and Doncricotopus dentatus sp.n. (Diptera, Chironomidae, Orthocladiinae) from Finland. Annales Entomologici Fennici 51[1985](4): 101–104.

Tuiskunen J (1986b) A new northern species of Sympotthastia (Diptera, Chironomidae, Diamesinae). Annales Entomologici Fennici 52: 78–80.

Tuiskunen J (1986c) Tavastia australis, a new genus and species (Diptera, Chironomidae, Orthocladiinae) from Finland. Annales Entomologici Fennici 51[1985](4): 30–32.

Tuiskunen J (1986d) The Fennoscandian species of Parakiefferiella Thicnemann (Diptera. Chironomidae. Orthocladiinae). Annales Zoologici Fennici 23(2): 175–196. `http://www.sekj.org/PDF/anzf23/anz23-175-196.pdf`.

Tuiskunen J, Lindeberg B (1986) Chironomidae (Diptera) from Fennoscandia north of 68 deg. N, with a description of ten new species and two new genera. Annales Zoologici Fennici 23(4): 361–393. `http://www.sekj.org/PDF/anzf23/anz23-361-393.pdf`.

Tuomikoski R (1933) Notizen über die Clusiiden Finnlands. Notulae entomologicae 13(1): 15–19.

Tuomikoski R (1935) Mitteilungen über die Empididen (Dipt.) Finnlands I. Die Gattung Trichina Meig. Annales Entomologici Fennici 1(1): 95–101.

Tuomikoski R (1936) Mitteilungen über die Empididen (Dipt.) Finnlands III. Die Gattung Leptodromiella n. gen. Annales Entomologici Fennici 2(4): 187–190.

Tuomikoski R (1937) Mitteilungen über die Empididen (Dipt.) Finnlands IV. Die Gattung Ocydromia Meig. Annales Entomologici Fennici 3(1): 17–20.

Tuomikoski R (1957) Beobachtungen über einige Sciariden (Dipt.), deren Larven in faulem Holz oder unter der Rinde abgestorbener Bäume leben. Annales Entomologici Fennici 23(1): 3–35.

Tuomikoski R (1959) Mitteilungen über die Empididen (Dipt.) Finnlands VI. Trichinomyia gen.n., eine neue Ocydromiinengattung. Annales Entomologici Fennici 25(2): 103–110.

Tuomikoski R (1960a) Mitteilungen über die Empididen (Dipt.) Finnlands VII. Die Gattung Hormopeza Zett. Annales Entomologici Fennici 26(1): 99–107.

Tuomikoski R (1960b) Zur Kenntnis der Sciariden (Dipt.) Finnlands. Annales Zoologici Societatis Zoologicae Botanicae Fennicae ”Vanamo” 21(4): 1–164.

Tuomikoski R (1966) Mitteilungen über die Empididen (Dipt.) Finnlands VIII. Die Gattung Chelipoda Macq. Annales Entomologici Fennici 32(4): 321–326.

Tuovinen T, Aapro H (1981) Liriomyza trifolii (Diptera, Agromyzidae) introduced on chrysanthemum into Finland. Notulae Entomologicae 61(4): 173–174.

Ulmanen I, Brummer-Korvenkontio M (1971) Anopheles messeae messeae Falleroni (Dipt., Culicidae) in Finland. Acta entomologica fennica 28: 43–45.

Utrio P (1975) Anopheles claviger (Meig.), Aedes pionips Dyar and A. beklemishevi Den. (Diptera, Culicidae) found in Finland. Notulae Entomologicae 55: 63–64.

Utrio P (1976) Identification key to Finnish Mosquito Larvae (Diptera, Culicidae). Annales Agriculturae Fenniae 15: 128–136.

Utrio P (1977) A checklist of Finnish mosquitoes (Diptera, Culicidae), including Aedes annulipes, new to Finland. Notulae entomologicae 57(4): 130–132.

Utrio P (1979) Geographic distribution of mosquitoes (Diptera, Culicidae) in eastern Fennoscandia. Notulae Entomologicae 59: 105–123. http://www.mosquitocatalog.org/files/pdfs/134700-3.pdf.

Väisänen R (1979) New species of the genus Mycomya (Diptera, Mycetophilidae) from Finland. Annales Entomologici Fennici 45(4): 109–115. http://www.online-keys.net/sciaroidea/add01/Vaisanen_1979_new_Mycomya.pdf.

Väisänen R (1980) Mycomya mituda sp. n. from southern Finland (Diptera, Mycetophilidae). Notulae entomologicae 60: 63–64.

Väisänen R (1982) Genus Neoempheria (Diptera, Mycetophilidae) in Finland, with a description of a new species. Notulae entomologicae 62(1): 1–7.

Väisänen R (1983) The bat-flies (Diptera, Nycteribiidae) of Finland. Notulae entomologicae 63(3): 143–144.

Väisänen R (1984) A monograph of the genus Mycomya Rondani in the Holarctic region (Diptera, Mycetophilidae). Acta Zoologica Fennica 177: 1–346.

Väisänen R, Biström O (1991) Pachyneura fasciata Zetterstedt found at Saarijärvi, Central Finland (Diptera, Pachyneuridae). Entomologica fennica 2(1): 4.

Valkeila E (1959) Suomen Hyönteistieteellisen Seuran kesäretki Kuusamoon v. 1958. Annales Entomologici Fennici 25(1): 49–51.

Valle KJ (1938) Kokousselostuksia Sitzungsberichte. Annales Entomologici Fennici 4(3): 194–200.

Valle KJ (1939) Kokousselostuksia. Annales Entomologici Fennici 5(2): 171–176.

Valtonen R (1993) Suonimiinaajakärpänen Euroopassa ongelmallisempi kuin floridankärpänen. Kasvinsuojelulehti 26(3): 82–85.

van Steenis J, Goeldlin de Tiefenau P (1998) Description of and key to the European females of the Platycheirus peltatus sub-group (Diptera, Syrphidae), with a description of the male and female of P.islandicus Rhingdahl, 1930, stat.n. Mitteilungen der Schweizerischen Entomologischen Gesellschaft 71: 187–199.

van Steenis J, Lucas JAW (2011) Revision of the West-Palaearctic species of Pipizella Rondani, 1856 (Diptera, Syrphidae). Dipterists Digest 18: 127–180.

Vappula NA (1941) Zwei Dasyneura-Arten als Schädlinge der schwarzen Johannisbeere in Finnland. Annales Entomologici Fennici 7(2): 112–117.

Väre H, Itämies J (1995) Phorbia phrenione (Seguy, 1937) (Diptera: Anthomyiidae) in Finland. Sahlbergia 2: 119–124.

Černý M, Merz B (2006) New records of Agromyzidae (Diptera) from the Palaearctic region. Mitteilungen der Entomologischen Gesellschaft Basel 79: 77–106.

Verbeke J (1964) Contribution a l'etude des dipteres malacophages II. Données nouvelles sur la taxonomie et la répartition géographique des Sciomyzidae paléarctiques. Bulletin de l'Institut Royal des Sciences Naturelles de Belgique 40(8): 1–27.

Verves YG (1986) Family Sarcophagidae. In: Soós A, Papp L (Eds) Catalogue of Palaearctic Diptera. Calliphoridae - Sarcophagidae. Volume 12. Akademiai Kiado, Budapest & Elsevier, Amsterdam, 58–193 ISBN 0444995781.

Verves YG, Khrokalo LA (2006) Review of Macronychiinae (Diptera, Sarcophagidae) of the World. Vestnik Zoologii 40(3): 219–239. http://www.nbuv.gov.ua/portal/chem_biol/vzl/2006/pdf/2006/3/03_Verves_khrokalo.pdf.

Vikberg V (1978) Brief reports. Hexomyza schineri (Giraud) (Agromyzidae) tavattu Suomesta. Notulae Entomologicae 58(4): 176.

Vikberg V (1986) Agromyza demeijerei Hendel, 1920 (Agromyzidae) Suomesta. Notulae Entomologicae 66(4): 190.

Vikberg V, Malinen P (2012) Ahdekaunokin varsien hyönteisten kasvatus kevättalvella 2012 Janakkalassa. Sahlbergia 18(2): 17–23.

Vilkamaa P, Heller K, Hippa H (2013a) New species of Sciaridae (Diptera) to the Finnish fauna. Sahlbergia 19(1-2): 20–29.

Vilkamaa P, Hippa H (2005) Phylogeny of Peyerimhoffia Kieffer, with the revision of the species (Diptera: Sciaridae). Insect Systematics & Evolution 35(4): 457–480 doi:http://dx.doi.org/10.1163/187631204788912445.

Vilkamaa P, Hippa H, Heller K (2013b) Notes on the taxonomy of the Holarctic Corynoptera Winnertz sensu lato (Diptera, Sciaridae), with description of six new species. Zootaxa 3710: 322–332.

Vilkamaa P, Hippa H, Heller K (2013c) Taxonomy of the Sciaridae (Diptera) of Northern Europe: description of eight new species. Studia Dipterologica 20(1): 47–58.

Vilkamaa P, Hippa H, Komarova LA (2004) The genus Dichopygina gen. n. (Diptera: Sciaridae). Insect Systematics & Evolution 35(1): 107–120. http://dx.doi.org/10.1163/187631204788964691.

Vilkamaa P, Komonen A (2001) Redescription and biology of Trichosia (Baeosciara) sinuata Menzel & Mohrig (Diptera: Sciaridae). In: Entomologica fennica. vol. 12, 46–49.

Vilkamaa P, Salmela J, Hippa H (2007) Black fungus-gnats in deciduous forest habitat in northern Europe, with the description of Bradysia arcula sp. n. (Diptera: Sciaridae). Entomologica fennica 18(4): 226–231.

Viramo J (1992) Koillismaan (Ks) vaaksiaisista (Diptera, Tipulidae). Oulanka reports 10: 33–40.

von Tschirnhaus M (1992) Minier- und Halmfliegen (Agromyzidae, Chloropidae) und 53 weitere Familien aus Malaise-Fallen in Kiesgruben und einem Vorstadtgarten in Köln. Decheniana - Beihefte 31: 445–497.

Šifner F (2003) The family Scathophagidae (Diptera) of the Czech and Slovak republics (with notes on selected Palaearctic taxa). Acta Musei Nationalis Pragae, Series B, Historia Naturalis 59(1-2): 1–90.

Vuorimies J (1984) Kaksi maalle uutta Haematopota-lajia (Tabanidae). Notulae Entomologicae 64(4): 203.

Widenfalk O, Gyllenstrand N, Sylvén E, Solbreck C (2002) Identity and phylogenetic status of two sibling gall midge species (Diptera: Cecidomyiidae: Contarinia) on the perennial herb Vincetoxicum hirundinaria. Systematic Entomology 27(4): 519–528 doi:10.1046/j.1365-3113.2002.00193.x. http://doi.wiley.com/10.1046/j.1365-3113.2002.00193.x.

Winqvist K (2002) Faunistic notes. Species new to the Finnish fauna. Diptera. Sahlbergia 7: 36–37.

Winqvist K (2007) Lauri Tiensuun dipterologinen perintö ja hänen Agromyzidae-kokoelmastaan löytyneet maalle uudet lajit. Diamina 16[2007]: 25–30.

Winqvist K (2011) Ruissalon kärpäsistä (Diptera, Brachycera). w-album 11: 3–23. http://org.utu.fi/harrastus/TEKS/w-album/w_album_11.pdf.

Winqvist K, Kahanpää J (2007) Checklist of Finnish flies: superfamilies Tephritoidea and Sciomyzoidea (Diptera: Brachycera). Sahlbergia 12(1): 20–32.

Wood DM (1975) Notes on Allophorocera with a description of a new species (Diptera:Tachinidae) from Finland. The Canadian Entomologist 106(6): 667–671.

Woodley NE (2011a) A World Catalog of the Stratiomyidae (Insecta: Diptera): A Supplement with Revisionary Notes and Errata. In: Brake I, Thompson FC (Eds) Contributions to the Systema Dipterorum (Insecta: Diptera). Myia 12:. vol. 12 Pensoft Publishers & North America Dipterists Society, Sofia, Moscow & Washington (D.C.), 379–415.

Woodley NE (2011b) A world catalog of the Xylophagidae (Insecta: Diptera). In: Brake I, Thompson FC (Eds) Contributions to the Systema Dipterorum (Insecta: Diptera). Myia 12:. Pensoft Publishers & North America Dipterists Society, Sofia, Moscow & Washington (D.C.), 455–500.

Wülker W (1959) Drei neue Chironomiden-Arten (Dipt.) und ihre Bedeutung für das Konvergenzproblem bei Imagines und Puppen. Archiv für Hydrobiologie, Suppl. 25: 44–64.

Wülker W (1997) Chironomus esai n. sp. (Diptera: Chironomidae) in lakes and reservoirs of central Fennoscandia. Entomologica fennica 8(3): 171–176.

Wülker W (1999) Fennoscandian Chironomus species (Dipt., Chironomidae) - identified by karyotypes and compared with the Russian and Central European fauna. Studia dipterologica 6(2): 425–436.

Wülker W, Ryser HM, Scholl A (1981) Revision der Gattung Chironomus Meigen (Dipt.). VI. C. holomelas Keyl, C. saxatilis n. sp., C. melanescens Keyl. Revue Suisse de Zoologie 88(4): 903–924.

Wülker WF (1991) Chironomus fraternus sp. n. and C. beljaninae sp. n., Sympatric Sister Species of the Aberratus Group in Fennoscandian Reservoirs. Entomologica fennica 2: 97–109.

Wülker WF, Butler MG (1983) Karyosystematics and morphology of Northern Chironomus (Diptera: Chironomidae): Freshwater species with larvae of the salinarius-type. Entomologica scandinavica 14(2): 121–136 doi:http://dx.doi.org/10.1163/187631283X00010.

Wülker WW (1973) Revision der Gattung Chironomus Meig. III. Europäische Arten des thummi-Komplexes. Archiv für Hydrobiologie 72: 356–374.

Zaitzev AI (2006) Palaearctic species of the Boletina nitida-group (Diptera: Mycetophilidae) including the description of a new species. Studia dipterologica 12[2005](2): 243–253. `http://www.online-keys.net/sciaroidea/2000\_/Zaitzev\_et\_al\_2005\_Boletina\_nitida-group\_n\_sp.pdf`.

Zaitzev AI, Ševčík J (2002) A review of the Palaearctic species of the Leptomorphus quadrimaculatus (Matsumura) group (Diptera: Mycetophilidae). Acta Zoologica Academiae Scientiarum Hungaricae 48(3): 203–211. `http://actazool.nhmus.hu/48/3/ac48\_3.html`.

Zaitzev AO, Polevoi AV (1995) New species of fungus gnats (Diptera: Mycetophilidae) from the Kivach Nature Reserve, Russian Karelia. Entomologica fennica 6(4): 185–195.

Zaitzev VF (1989) Family Bombyliidae. In: Soós A, Papp L (Eds) Catalogue of Palaearctic Diptera. Therevidae-Empididae. Volume 6. Akademiai Kiado, Budapest & Elsevier, Amsterdam, 43–169.

Zatwarnicki T (1987) New synonyms and records of Palearctic Scatophila (Diptera, Ephydridae). Polskie Pismo Entomologiczne 57: 277–298.

Zatwarnicki T, Mathis WN (2011) Heterogeneity in shore fliesThe case of Glenanthe Haliday (Diptera: Ephydridae) in the Old World. Annales de la Société Entomologique de France (nouvelle série) 47(3-4): 418–443. http://www.zoologie.umh.ac.be/asef/pdf/2011_47_03_04$\backslash$Compact$\backslash$Zatwarnicki_\&_Mathis_2011_ASEF_47_3_4_418_443_compact.pdf.

Zetterstedt JW (1859) Diptera Scandinaviae. Disposita et descripta. Vol. 13. Officina Lundbergiana, Lundae [=Lund], xvi+ 4943–6190 pp.

Zhang CT, Shima H (2005) A revision of the genus Trixa Meigen (Diptera: Tachinidae). Insect Science 12(1): 57–71.

Zinovjev AG (1987) On the taxonomy of flies of the genus Phaonia R.-D. (Diptera, Muscidae). Entomologicheskoe Obozrenie 66(2): 436–441.

Zlobin VV (1993) Chromatomyia ochracea (Hendel, 1920) new faunistic records (Diptera: Agromyzidae). International Journal of Dipterological Research 4(3): 171–172.

Zlobin VV (1994a) Review of mining flies of the genus Napomyza Westwood Diptera: Agromyzidae. 5. Phytomyza species currently placed in the genus Napomyza Westwood. International Journal of Dipterological Research 5(4): 289–311.

Zlobin VV (1994b) Review of mining flies of the genus Napomyza Westwood (Diptera: Agromyzidae). IV. Palaearctic species of lateralis-group. International Journal of Dipterological Research 5(1): 39–78.

Zlobin VV (1995) Review of mining flies of the genus Cerodontha. IV. Subgenus Poemyza. Zoosystematica Rossica 1[1992]: 117–141.

Zlobin VV (2001) A new European Agromyza species (Diptera: Agromyzidae). International Journal of Dipterological Research 12(2): 91–95.

Zlobin VV, Pakalniškis S (1993) Calycomyza subapproximata (Sasakawa, 1955) a new combination and faunistic records (Diptera: Agromyzidae). International Journal of Dipterological Research 4(3): 177–179.
